# PM2.5 promotes NSCLC carcinogenesis through translationally and transcriptionally activating DLAT-mediated glycolysis reprograming

**DOI:** 10.1186/s13046-022-02437-8

**Published:** 2022-07-22

**Authors:** Qianqian Chen, Yiling Wang, Lin Yang, Liyuan Sun, Yuxin Wen, Yongyi Huang, Kaiping Gao, Wenhan Yang, Feng Bai, Lijuan Ling, Zizi Zhou, Xiaoming Zhang, Juan Xiong, Rihong Zhai

**Affiliations:** 1 School of Public Health, Shenzhen University Health Science Center, 1066 Xueyuan Ave, Shenzhen, 518055 China; 2 Guangdong Provincial Key Laboratory of Genome Stability and Disease Prevention, International Cancer Center, Shenzhen University Health Science Center, 1066 Xueyuan Ave, Shenzhen, 518055 China; 3 Department of Thoracic Surgery, The People’s Hospital of Shenzhen, 1017 North Dongmen Road, Shenzhen, 518020 China; 4 Department of Thoracic Surgery, Shenzhen University General Hospital, 1098 Xueyuan Ave, Shenzhen, 518055 China

**Keywords:** PM2.5, Glycolysis reprograming, Non-small cell lung cancer (NSCLC), DLAT, eIF4E, Sp1, Translation, Transcription

## Abstract

**Background:**

Airborne fine particulate matter (PM2.5) has been associated with lung cancer development and progression in never smokers. However, the molecular mechanisms underlying PM2.5-induced lung cancer remain largely unknown. The aim of this study was to explore the mechanisms by which PM2.5 regulated the carcinogenesis of non-small cell lung cancer (NSCLC).

**Methods:**

Paralleled ribosome sequencing (Ribo-seq) and RNA sequencing (RNA-seq) were performed to identify PM2.5-associated genes for further study. Quantitative real time-PCR (qRT-PCR), Western blot, and immunohistochemistry (IHC) were used to determine mRNA and protein expression levels in tissues and cells. The biological roles of PM2.5 and PM2.5-dysregulated gene were assessed by gain- and loss-of-function experiments, biochemical analyses, and Seahorse XF glycolysis stress assays. Human tissue microarray analysis and ^18^F-FDG PET/CT scans in patients with NSCLC were used to verify the experimental findings. Polysome fractionation experiments, chromatin immunoprecipitation (ChIP), and dual-luciferase reporter assay were implemented to explore the molecular mechanisms.

**Results:**

We found that PM2.5 induced a translation shift towards glycolysis pathway genes and increased glycolysis metabolism, as evidenced by increased L-lactate and pyruvate concentrations or higher extracellular acidification rate (ECAR) in vitro and in vivo. Particularly, PM2.5 enhanced the expression of glycolytic gene DLAT, which promoted glycolysis but suppressed acetyl-CoA production and enhanced the malignancy of NSCLC cells. Clinically, high expression of DLAT was positively associated with tumor size, poorer prognosis, and SUVmax values of ^18^F-FDG-PET/CT scans in patients with NSCLC. Mechanistically, PM2.5 activated eIF4E, consequently up-regulating the expression level of DLAT in polysomes. PM2.5 also stimulated transcription factor Sp1, which further augmented transcription activity of DLAT promoter.

**Conclusions:**

This study demonstrated that PM2.5-activated overexpression of DLAT and enhancement in glycolysis metabolism contributed to the tumorigenesis of NSCLC, suggesting that DLAT-associated pathway may be a therapeutic target for NSCLC.

**Supplementary Information:**

The online version contains supplementary material available at 10.1186/s13046-022-02437-8.

## Background

The incidence of lung cancer in never-smokers (LCNS) has been rapidly increasing in recent decades, accounting for approximately 25% of lung cancer cases worldwide [1, 2]. In fact, it has been recognized that lung cancer in smokers and never-smokers are two distinct entities, differing not only in epidemiology but also at the levels of molecular profiles and tumoral microenvironments [3, 4]. Among the risk factors that contributed to the development of LCNS, PM2.5, the major air pollutant worldwide, was considered as the major culprit [5, 6]. For instance, studies from the 17 Prospective European Cohort have concluded that a 5 μg/m^3^ increment in annual exposure to PM2.5 was associated with increased hazard ratio of lung cancer incidence by 1.18 [7]. Similarly, the incident rate ratio (IRR) per 10 μg/m^3^ increase in PM2.5 for lung cancer was 1.19 (95% CI: 1.09, 1.30) in the USA [8]. In consistent with these, a nationwide study in 295 Chinese counties has shown that an increase of 10 μg/m^3^ exposure to PM2.5 would result in higher incidence of lung cancer by 4.20% for males and 2.48% for females [9]. Furthermore, it has also been revealed that the increment in PM2.5 concentration was positively correlated with increase in mortality of lung cancer [10–12]. Indeed, outdoor PM2.5 pollution has been defined as a Group 1 human carcinogen by the International Agency for Research on Cancer (IARC) in 2013 [13]. Nevertheless, the molecular mechanisms by which PM2.5 induces lung cancer remain poorly understood.

Cancer is considered a disease that originates from overproduction of oncogenes and reduced amount of tumor suppressors [14]. To investigate genetic variances underlying PM2.5-induced pathological conditions, prior studies have focused primarily on the identification of differentially expressed mRNAs, based on the assumption that mRNA changes must be accompanied by coordinated changes in the protein. However, as the whole transcriptome includes large pools of mRNAs not undergoing active translation, only 40% of the variation in the protein expression can be explained by mRNA levels [15]. In fact, it has been shown that the correlation between mRNA transcripts and protein levels was generally poor [15, 16]; and that translation efficiency, rather than mRNA abundance, contributed most to the final protein amount [17]. Emerging evidence has also demonstrated that in the flow of genetic information, translation initiation is the rate limiting step for protein synthesis, and critical for the activity of oncogenes and tumor suppressor genes [18]. As ribosome sequencing (Ribo-seq) can directly quantify the transcripts of ribosome-protected mRNAs, it has been proposed that Ribo-seq may provide a more accurate and complete picture of gene expression in addition to conventional RNA sequencing (RNA-seq) [19]. Nevertheless, whether PM2.5 could contribute to the tumorigenesis of NSCLC through regulating the translational rate of ribosomes is unknown.

In this study, we present what to our knowledge is the first thorough analysis of transcriptional and translational landscapes in PM2.5-exposed cells. We found that PM2.5 enhanced the translation efficiency of glycolysis pathway genes, with DLAT as the most over-translated one. We showed that DLAT increased glycolysis, promoted proliferation, and suppressed apoptosis in NSCLC cells. Up-regulation of DLAT was associated with poorer outcome in NSCLC patients. We verified that PM2.5 promoted glycolysis metabolism and DLAT expression in vitro and in vivo. We demonstrated that PM2.5 activated transcription factor Sp1, which transcriptionally promoted the expression of DLAT. In addition, we revealed that PM2.5 enhanced the expression of ribosomal eIF4E, which selectively up-regulated DLAT translation in polysomes. Overall, this research revealed a previously unidentified mechanism of PM2.5 for promoting glycolysis metabolism and the malignancy of NSCLC, suggesting that DLAT-associated glycolysis pathway may be a promising target for NSCLC treatment.

## Materials and methods

### PM2.5 samples

Urban particulate matter samples SRM 1648a (encoded as PM2.5) were purchased from National Institute of Standards and Technology (NIST, MD, USA). The PM2.5 was collected in an urban area and all constituents in SRM 1648a were naturally present in the material before processing (NIST Certificate of Analysis, 2020). SRM 1648a includes 25 metals, 21 polycyclic aromatic hydrocarbons (PAH), and 7 polychlorinated biphenyls (PCBs). As SRM 1648a has uniform constituents，it is usually used as a reference to estimate the impact of mixed PM2.5 on health damage and may also be used as the reference material [20]. Before each test, PM2.5 was dissolved in phosphate buffer saline (PBS) for infusion and the samples were sonicated for 20 min before use in each experiment.

We determined the PM2.5 dosage for in vitro experiments on the basis of previous reports in PM-2.5-induced cytotoxicity and on our pilot cell viability experiments. BEAS-2B cells (the normal human bronchial epithelial cell line) were exposed to different levels of PM2.5 for 24 h and the cell viability was tested by CCK-8 methods. No significant changes of cell viability were observed between cells exposed to PM2.5 (25, 50, 100, 200 μg/mL, respectively) and control cells. When the concentration of PM2.5 reached 400 μg/mL, the cell viability significantly (*P* < 0.001) decreased to approximately 50% (≈LC50) (Fig. S1). This effect dose was similar to reports in which the dose of SRM 1648a to reduce cell viability to 50% was about 500 μg/mL [21]. Considering that previous studies have also shown that the optimum doses of PM2.5 to induce apoptosis, inflammatory cytokine release, or DNA damage in cells were between 200 to 500 μg/mL [22–26]. we chose 100 μg/mL, 200 μg/mL, and 400 μg/mL as the working doses of PM2.5 for in vitro experiments in the present study.

The doses of PM2.5 used for animal experiment were estimated according to the World Health Organization (WHO) air quality guidelines (WHO, 2006) and physiological parameters of rats: The respiratory volume of an adult rat (200 g) is 0.86 mL at each breath and the breath rate is 85 times per min, and breathing quantity per day is 0.105 m^3^. According to the WHO annual mean concentration (target-1, 35 μg/m^3^), PM2.5 exposure level was calculated as 3.684 μg/per day. After multiplying by a 100-fold uncertainty factor, the exposure level of PM2.5 was estimated to be 1.8 mg/kg body weight (bw)/per day. Using 1.8 mg/kg bw as the low-dose, a 3-fold (5.4 mg/kg bw) and a 9-fold (16.2 mg/kg bw) doses were considered as moderate- and high-dose, respectively [27, 28].

### Clinical samples

This study was approved by the Medical Ethics Committee of Shenzhen University Health Science Center (Approved no. 2016002). Written informed consents were obtained from all participants. NSCLC subjects were recruited from patients who underwent surgical resection in the Department of Thoracic Surgery at People’s Hospital of Shenzhen, China (Supplementary Table S9). NSCLC was diagnosed according to the criteria of Lung Cancer Stage Classification (The Eighth Edition) [29]. Tissue samples were collected in the operating room and the surgical specimens were fixed in formalin and embedded in paraffin before they were archived.

### Immunohistochemistry (IHC) analysis

The paraffin-embedded tissue slides were deparaffinized and dehydrated in xylene and alcohol, and then incubated with the primary antibodies against DLAT (Sigma, R38114) at 4 °C overnight and then with HPR-labelled secondary antibody (MaxVision TM HRP, 210908S407n) at 37 °C for 1 h. The 3,3-diaminobenzidine (DAB) solution was used for development of brown staining to identify the expression of DLAT. Images were observed and analyzed under microscope (Olympus) with Image J software.

### Cell culture and transfection

BEAS-2B and human NSCLC cell lines (A549, and PC9) were ordered from Shanghai Institute for Biological Science (Shanghai, China). Each cell lines were authenticated by measuring the short-tandem repeat (STR) DNA profiles. No contamination of mycoplasma was found in these cell lines. A549, PC9, and BEAS-2B cells were cultured in Dulbecco’s modified Eagle’s medium (Gibco, NY, USA) containing 10% fetal bovine serum (FBS, Gibco, NY, USA) in a humidified atmosphere with 5% CO_2_ at 37 °C. For in vitro PM2.5 exposure experiments, cells were treated with different concentrations of PM2.5 (0, 200, 400 μg/ml) for 24–48 h.

### Ribosome sequencing (Ribo-seq)

A total of 1×10^7^ BEAS-2B cells were lysed in polysome lysis buffer (20 mM Tris•Cl at pH 7.4, 150 mM NaCl, 5 mM MgCl2, 1 mM DTT, 1% Triton X-100, Turbo DNase I 25 U/ml, 50 mg/mL cycloheximide). Then, cell lysate was treated with unspecific endoribonuclease RNase to digest the free RNAs other than ribosome-protected fragments (RPFs). Isolation of ribosome was performed by size-exclusion chromatography with MicroSpin S-400 HR columns (GE Healthcare, USA). Recovered RPFs were then subjected to rRNA depletion kit to remove rRNA contamination. Small RNA (< 200 bp) was resolved by electrophoresis on a 15% (w/v) polyacrylamide gel (PAGE) with 7 M urea. The RPFs bands (~ 30 nt) were cut for RNA extraction, phosphorylated and ligated with 5′ and 3′ adapters, reverse transcribed and PCR amplified, respectively. Libraries were then sequenced on Illumina Novaseq platform using the PE150 (paired-end 150 nt) recipe. The sequencing reads were mapped to human genome database by TopHat2 software and then quantified by the HTSeq and DESeq2 R packages.

### RNA-seq

BEAS-2B cells from the same cultures used in the Ribo-seq experiments were processed in parallel for RNA-seq. Total RNA was isolated with TRIzol reagent and RNA-seq libraries were prepared using the NEBNext® UltraTM RNA Library Prep Kit for Illumina® (NEB, USA) following manufacturer’s recommendations. Briefly, mRNA was purified from total RNA using the poly-T+ oligo-attached magnetic beads. RNA samples were fragmented and then used for first- and second-strand cDNA synthesis with random hexamer primers. The cDNA fragments were treated with DNA end repair kit and NEBNext adaptor was ligated at the 3′ end of the DNA fragments. The library fragments were purified with AMPure XP system (Beckman Coulter, Beverly, USA) and then subjected to PCR amplification. After cluster generation by the TruSeq PE Cluster Kit v3-cBot-HS (Illumia, USA), the library preparations were sequenced on an Illumina Novaseq platform (150 bp paired-end). Sequencing reads from RNA-seq were aligned to the reference genome (GRCh38) using Hisat2 v2.0.5. Feature Counts v1.5.0-p3 was used to count the reads numbers and gene expression levels were calculated by the Reads per kilobase of transcript, per millions mapped reads (RPKM). Differential expression analysis was performed using the DESeq2 R package (1.16.1).

### TE calculation

The abundance of ribosome footprints of an mRNA depends on the translation rate and on the level of mRNA expression. Therefore, TE (translation efficiency) is commonly estimated as the ratio between RPF and expressed mRNA counts (mRNA) (TE = RPKM in Ribo-seq/RPKM in RNA-seq). Differences in TE between PM2.5-exposed and control samples were calculated using the Ribodiff software.

### Animals and PM2.5 intratracheal instillation

4-week old male SD rats were purchased from Guangdong Experimental Animal Center (Foshan, China) and were housed in a pathogen-free environment with free access to food and water. After one-week adaption, rats were randomly divided into 4 groups (*n* = 5 per group): three experimental groups exposed to PM2.5 (1.8, 5.4, 16.2 mg/kg body weight) [27] by intratracheal instillation every 3 days for 24 days (in total 8 times), and one control group received equal volume of saline (Fig. S5A). At the end of experiments, the rats were euthanized, and the lung tissue were isolated and stored at -80 °C for subsequent analyses. All animal experiments were performed in accordance with the protocols approved by the Animal Ethical Committee of Shenzhen University.

### Immunohistochemical (IHC) staining of rat lung tissues

The lung tissue was fixed in 4% paraformaldehyde solution. After paraffin embedding, 4 μm sections were cut and stained with hematoxylin and eosin to reveal the pathology characteristics of lung tissue. The slides were treated with graded alcohols for rehydration, and with EDTA buffer for 10 min at 98 °C for antigen retrieval. Endogenous peroxidase activity was blocked with 3% hydrogen peroxide solution at room temperature for 10 minutes. Nonspecific binding was prevented by blocking with normal goat serum at room temperature for 30 minutes. Sections of lung tissue were incubated with primary antibodies against Ki-67 and Caspase3 (Proteintech, Wuhan, China) at 4 °C overnight. Tissue sections were then treated with secondary antibody (goat anti-rabbit IgG H&L, HRP), stopped with freshly configured DAB solution, and counter-stained with hemetoxylin. The areas of positive staining were evaluated by ImageJ 1.53a (National Institutes of Health, USA).

### Quantification of lactate and pyruvate

For in vitro measurement of L-lactate and pyruvate levels, 1 × 10^6^ cells were seeded into each well of the six-well plate. Cells were treated with PM2.5, PBS, DLAT-overexpression or NC vector, respectively. For tissue analysis, 100 mg tissues were individually ground with liquid nitrogen, and the homogenate was resuspended with 0.4 ml 0.86% saline water and thoroughly vortexed. The samples were then centrifuged at 3500 rpm/min at 4 °C for 15 min. Then the supernatants were collected for further measurements. L-lactate and pyruvate levels in the cell cultured medium or in the supernatants of lung tissue homogenates were quantified using the L-lactate assay kit (A019–2-1, Jiancheng Bioengineering Institute, Nanjing, China) and the pyruvate assay kit (A081–1-1, Jiancheng Bioengineering Institute, Nanjing, China) according to the manufacturer’s instruction. The sample absorbance (530 nm for L-lactate and 505 nm for pyruvate) was detected using the Synergy HTX Multi-Mode Microplate Reader (BioTek, Guangzhou, China). All in vitro experiments were performed in triplicate.

### Measurement of ECAR and OCR

The extracellular acidification rate (ECAR) and oxygen consumption rate (OCR) of cells were assessed using the Seahorse XFe24 Flux Analyzer (Seahorse Bioscience, Agilent). The glycolytic stress test kit (Seahorse Cat. #103020–100) and mitochondrial stress test kit (Seahorse Cat. #103015–100) were used for ECAR and OCR detection, respectively. Briefly, 1 × 10^4^ cells were seeded in the 96-well XF Seahorse incubation plate as the protocol indicated. Cells were cultured at 37 °C in XF base medium (pH 7.4), and glucose (10 mM), glutamine (1 mM), 2-DG (50 mM) and oligomycin (1 μM) were added sequentially into the plates at specific time points following the manufacturer’s guidelines. Data of OCR and ECAR were measured and plotted using the Seahorse XF24 software.

### Plasmid construction, siRNA, and cell transfection

DLAT overexpression plasmid and the empty negative control (NC) were constructed using the pcDNA3.1-GFP vectors, respectively. siRNAs targeting DLAT, eIF4E, and Sp1, and corresponding NCsequences were designed and synthesized by RiboBio Co., Ltd. (Guangzhou, China). Cells were transfected with plasmids, siRNAs, or NCs, respectively, using the Lipofectamine 2000 reagents (Invitrogen, Shanghai, China) according to the manufacturer’s protocol. All sequences of siRNAs are listed in Supplementary Table S10.

### Cell viability assay

Cell proliferation rate was determined with Cell Counting Kit-8 kit (Dojindo Molecular Technologies, Osaka, Japan) according to the manufacturer’s instructions. Briefly, cells were seeded into 96-well plates (3000 cells/well) and transfected with plasmid, siRNAs, or their corresponding NCs. After incubation for 48 h, 10 μl CCK-8 reagent was added to each well and then incubated for another 1 h. The absorbance at 450 nm was measured by a Synergy HTX Multi-Mode Microplate Reader (BioTek, Guangzhou, China). Each independent experiment was performed in triplicate.

### Transwell assay

Cell invasion was evaluated by Matrigel-coated Transwell chambers (Corning-Costar, NY, USA). Briefly, cells were incubated in the upper chamber of a 24-well Transwell plate. After transfection for 48 h, 5 × 10^4^ cells were seeded into the upper compartment, which contained RPMI1640 medium with 10% FBS. After 24 h incubation, non-invading cells were gently removed and the migrated cells were fixed with methanol followed by DAPI staining. The number of invaded cells was counted under light microscope (Nikon, ECLIPSE Ts2, Japan).

### Apoptosis assay

Flow cytometric analysis was performed to analyze the percentage of apoptotic cells using the Annexin V-FITC cell apoptosis kits (BD Biosciences, CA, USA) according to the manufacturer’s protocol. In brief, cells were transfected with plasmids or NCs for 48 h, and then stained with Annexin V-fluorescein isothiocyante (FITC) and propidium iodide (PI). The apoptotic rates of cells were determined by flow cytometer (Beckman Coulter, Inc., IN, USA) equipped with CytExpert software (version 2.3).

### Western blotting

Total proteins were extracted from cells or tissues using RIPA lysis buffer (Promega, Madison, WI, USA). Proteins were separated on an 8% SDS-PAGE gel and then transferred to PVDF membranes. The membrane was blocked with 5% skim milk at room temperature for 1 h and then incubated with primary antibodies (anti-DLAT, 1:1000 dilution, Abcam, ab51608; anti-SP1, ab101562, 1:1000 dilution, Abcam) at 4 °C overnight, followed by incubating with horseradish peroxidase (HRP) conjugated β-actin secondary antibodies (1:5000) at room temperature for 90 min. The protein bands were scanned and visualized by the GS700 imaging densitometer (Bio-Rad Laboratories) and analyzed by Image Studio software.

### Real-time PCR

Total RNA was isolated using the TRIzol reagents (Life Technologies, Shanghai, China) according to the manufacturer’s instructions. 1 μg of total RNA was reverse-transcribed into cDNA using the Yeasen cDNA kit (11121ES60, Shanghai, China). The transcript level of specific gene was amplified using the Yeasen qPCR kit (11201ES08) and was normalized to ACTB. The primers were synthesized by Riobio Co. (Guangzhou, China) and the sequences are listed in the Supplementary Table S10.

### Human lung tissue microarray

A tissue microarray containing slides of 92 NSCLC tumor tissues and adjacent normal lung tissue was purchased from Outdo Biotech, (Shanghai, China; HLugA180Su04). The patients’ clinical records and histopathological diagnoses were fully documented. Survival times was calculated in months and defined as the time from operation time until death, or censored if no death was noted at follow-up date. The protein level of DLAT was determined by semi-quantitative IHC assay, using the anti-DLAT antibody (Sigma-Aldrich，HPA040786). The results of IHC were independently scored by two independent observers. The positive stained samples were scored as follows: 1, ≤25% of positively stained cells; 2, > 25- ≤ 50% of positively stained cells; 3, > 50- ≤ 75% of positively stained cells; 4, ≥75% of positively stained cells. The intensity of staining was scored according to the following criteria: 0, negative staining; 1, weak staining; 2, moderate staining; and 3, strong staining. The final staining index (SI) was calculated by multiplying the percentage score by the staining intensity score, with SI values ≥3 being defined as high expression and SI < 3 being considered as low expression.

### Polysomal mRNA isolation and qRT-PCR

Isolation of ribosome-bound mRNA by polysome separation was performed as described previously with minor modifications. Briefly, A549 cells (1 × 10^7^) were incubated in DMEM medium containing 100 μg/mLcycloheximide for 10 min at 37 °C. All subsequent steps contained 100 μg/mL cycloheximide. After centrifugation, resuspended cells were treated with trypsin at 37 °C for 3 min, then lysed by polysome extraction buffer (PEB; 20 mM Tris-HCl, pH 7.5, 50 mM KCl; 10 mM MgCl_2_; 1 mM DTT; 100 μg/ml CHX; 200 μg/ml Heparin) containing 1% Triton-X100. After 30 min incubation on ice, lysates were centrifuged at 14,000 rpm for 30 min at 4 °C, and the resulting supernatant were collected and then loaded onto 5–50% sucrose density gradients. Gradients were then centrifuged at 38000 rpm for 120 min in an Optima L-100XP rotor (Beckman Coulter) using equal OD_260_ units of samples. Thirteen fractions of equal volume were collected, and their absorbances were measured. For technical reasons, fractions 1–3, 4–6, 7–9, 10–11, and 12–13 were pooled together as 1, 2, 3, 4, 5 combined fractions, respectively [30]. Messenger RNAs of 1 or 2 pooled fractions were light weight fraction (fractions 1–6) and mRNAs from 3, 4, 5 pooled fractions were heavy weight fraction (fractions 7–13). RNA was extracted from equal volume of the five combined fractions using TRIzol reagents (Life Technologies, Shanghai, China), reversely transcribed to cDNA using the PrimeScriptTM RT reagent Kit (Takara, Beijing, China) and amplified by qTR-PCR analysis using the TB Green Premix Ex TaqTM II kit (Takara, Beijing, China).

### Chromatin immunoprecipitation (ChIP) assay

The Sp1-binding sites surrounding DLAT promoter region were predicted using the JASPAR tools (http://jaspar.genereg.net/). Primers were designed to amplify the predicted binding sequences of DLAT promoter regions (Supplementary Table S10). In brief, the A549 cells were crosslinked with 1% formaldehyde (final concentration) and lysed with ChIP Lysis Buffer at 37 °C for 10 min. Chromatin was sonicated into 200 ~ 1000 bp fragments. The lysates were then incubated and precipitated with antibodies against the Sp1 antibody (mAb #9389) or control IgG (CST，9389S), after which DNA-protein immunocomplexes were collected, using the PierceTM protein A/G-agarose beads (Thermo Fisher Scientific, Shanghai, China), and treated with RNase A (Sigma) and proteinase K (Sigma). The precipitated chromatin DNA was recovered and analyzed by qRT-PCR.

### Dual luciferase reporter assay

To test the transcriptional influence of Sp1 on the DLAT promoter, wild-type and mutant DLAT promoter fragments containing the Sp1 binding site were cloned into the dual-luciferase reporter pGL3-basic, respectively. Sp1 was cloned into the pcDNA3.1 vector using the restriction enzymes EcoRI and XhoI. HEK293T cells were cultured at a density of 2 × 10^4^ cells/well in 96-well culture plates and were incubated overnight. On the next day, the cells were co-transfected with pGL3-basic-DLAT-WT and Sp1-pcDNA3.1, pGL3-basic-DLAT-MUT and Sp1-pcDNA3.1, or corresponding control vectors, and the internal control vector pRL-TK (Promega, Madison, WI), respectively, using LipofectamineTM 2000 (Invitrogen, Carlsbad, CA) according to instruction of the manufacturer. After 48 h post-transfection, luciferase activity was measured using the Dual Luciferase Reporter Gene Assay (Beyotime, Shanghai, China) on a Chemiluminescence analyzer (ZS/BK-L96C, Beijing, China). All vector constructs were verified by DNA sequencing. The primer sequences for the constructs are listed in Supplementary Table S10. At least three independent transfection experiments were carried out for each condition.

### In silico analyses

The gene sequences of DLAT (NM_001372031.1), FOXM1 (NM_001243088.2), GAPDH (NM_001256799.3), β-actin (NM_001101.5), MYC (NM_001354870.1), Cyclin D1 (NM_053056.3) were retrieved from the NCBI database (https://www.ncbi.nlm.nih.gov/gene/). The minimum free energies of 5′-UTRs of these genes and their corresponding secondary structures were predicted in silico using the online RNAfold software (http://rna.tbi.univie.ac.at/cgi-bin/RNAfold.cgi). The associations of overall survival (OS) and relapse free survival (RFS) with the expression levels of eIF4E, Sp1, and DLAT mRNA in NSCLC patients were analyzed by the online tool KM Plotter (https://kmplot.com), using median as the cutoff values in the TCGA dataset. Gene and protein expression levels of eIF4E, Sp1, and DLAT in tumor and normal tissues were analyzed using the TCGA dataset with the online analyzers (http://gepia.cancer-pku.cn/; http://ualcan.path.uab.edu/).

### Statistical analysis

Data are expressed as the means ± SD from at least three independent experiments. Statistical analyses were performed using SAS 9.4 software (SAS Institute, USA) and Graph Pad Prism 6 (Graph Pad, USA). Comparison between groups was conducted using Student’s t-test (for parametric data) or the Mann–Whitney test (for non-parametric data). The associations between DLAT expression levels and overall survivals of NSCLC patients were estimated by Kaplan-Meier method and analyzed by the log-rank test. Univariate and multivariate Cox proportional hazards models were performed to identify the factors that had a significant effect on survival outcome. *P*-value (two-sided) < 0.05 was considered statistically significant.

## Results

### PM2.5 causes distinct translational and transcriptional changes

**Fig. 1:**
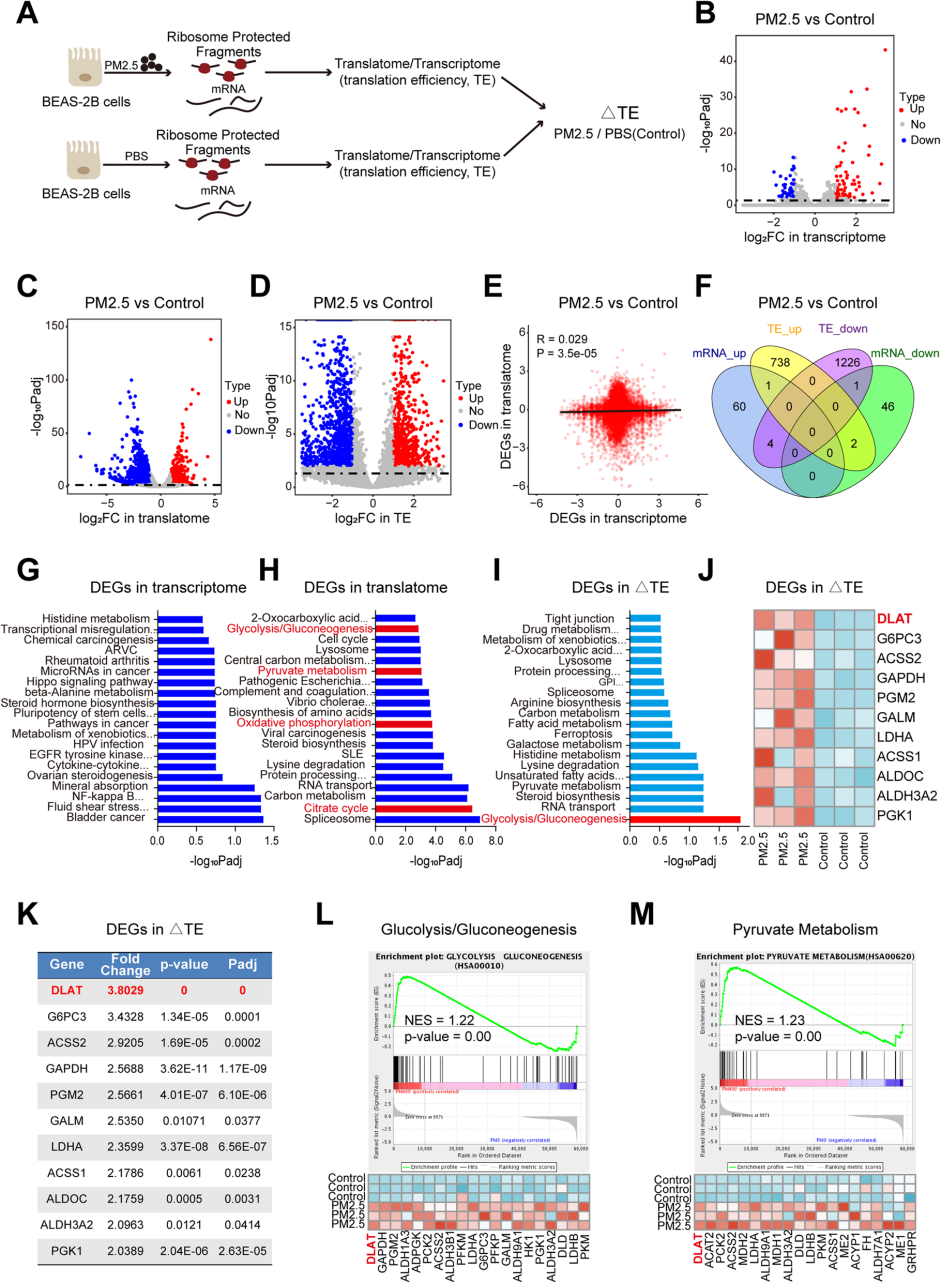
PM2.5 induces a translation efficiency (TE) shift towards up-regulation of glycolysis pathway genes. **A** Overview of experiments for RNA-seq and Ribo-seq in BEAS-2B cells following PM2.5 exposure. **B** Volcano map of differentially expressed genes (DEGs)in RNA-seq. Red and blue dots represent the upregulated and downregulated genes, respectively (FDR<0.05; |Log2(fold change)| >1). **C** Volcano plot of DEGs in Ribo-seq. **D** Volcano plot showing genes with significant changes in TE. **E** Genome-wide transcriptional and translational regulations showed very little correlation. **F** The four-way Venn diagram represented the different subsets of genes that are significantly upregulated or down-regulated at the TE and transcription levels. **G** KEGG pathway analysis of DEGs in transcriptome (RNA-seq). **H** Pathway analyses on DEGs in translatome revealed a shift towards glycolysis-related pathways. **I** Glycolysis/gluconeogenesis pathway was the only significantly enriched pathway in genes with TE changes. **J** Heatmap of DEGs in TE in the “Glycolysis/gluconeogenesis pathway”. **K** Fold change of TE for glycolytic genes between PM2.5-exposed cells and control cells. **L** Gene set enrichment analysis (GSEA) of Ribo-seq data showing the enrichment of glycolysis/gluconeogenesis pathway (upper) and the gene signature (lower) in PM2.5-exposed cells, with DLAT as the top up-regulated gene. **M** GSEA of Ribo-seq data revealing the enrichment of pyruvate metabolism pathway (upper) and the gene set in PM2.5-exposed cells, with DLAT ranking as the top gene. NES, normalized enrichment score

To identify the differentially expressed (DE) genes in transcription and translation in response to PM2.5 exposure, human normal bronchial epithelial cells (BEAS-2B) were treated with PM2.5 and phosphate-buffered saline (PBS), respectively. Parallel RNA-seq and Ribo-seq were carried out to contrast genome-wide transcriptional and translational differences between cells with and without PM2.5 exposure (Fig. 1A). Overall, RNA-seq and Ribo-seq reads were highly reproducible across three biological replicates (Fig. S2A). Notably, the length distributions of Ribo-seq libraries in PM2.5-exposed cells were different from that of control cells (Fig. S2B), suggesting that PM2.5 induced significant translation alterations. Furthermore, Ribo-seq reads were presented the expected length and triplet periodicity (Fig. S2C), and were mostly mapped to annotated protein-coding genes (Fig. S2D). In particular,we identified 405 DE-genes in RNA-seq (Fig. 1B, Fig. S3A), 3501 DE-genes in Ribo-seq (Fig. 1C, Fig. S3B), and 3697 DE-genes with changes in translation efficiency (TE) (Fig. 1D, Fig. S3C), indicating that the molecular responses to PM2.5 were more governed by translation than by transcription. Besides, it is also striking to observe extensive reduction in transcript level across the genome following PM2.5 exposure, and, conversely, a more pronounced increase in ribosome-associated transcripts following exposure to PM2.5 (Fig. S3D-F). Nevertheless, there was very little correlation or overlap between the DE-genes of transcriptome and that of translatome (Fig. 1E-F).

### PM2.5 induces translation efficiency shift towards glycolysis pathway

We next examined the functional implications of DE-genes between PM2.5-exposed and control cells. Pathway analysis revealed that transcriptionally DE-genes in PM2.5-exposed cells were enriched in cancer- and metabolism-related pathways (Fig. 1G). While the enrichment of translationally DE-genes showed a shift towards glucose metabolism-related pathways (Fig. 1H). When analysis was confined in DE-genes with changes in TE, the glycolysis/gluconeogenesis was the only significantly enriched pathway (Fig. 1I). Among the top DE-glycolytic genes regulated by PM2.5, DLAT displayed the highest fold change in TE and smallest *P*-value between PM2.5-exposed and control cells (Fig. 1 J-K). Similarly, gene set enrichment analysis (GSEA) showed that the glycolysis/gluconeogenesis and pyruvate metabolism pathway genes were significantly up-regulated in PM2.5-exposed cells, with DLAT ranking as the top gene in these pathways (Fig. 1 L-N). Since molecular responses in PM2.5-exposed cells were more remarkable in translatome than in transcriptome; and translation efficiency, rather than mRNA abundance, contributed most to the final protein amount [17], we hypothesized that translational alterations in glycolysis pathway may be critical for PM2.5-induced pathogenesis. To test this hypothesis, we examined the impacts of PM2.5 on glycolysis metabolism in vitro and in vivo.

### PM2.5 promotes glycolysis, but not mitochondrial respiration, in vitro and in vivo

**Fig. 2:**
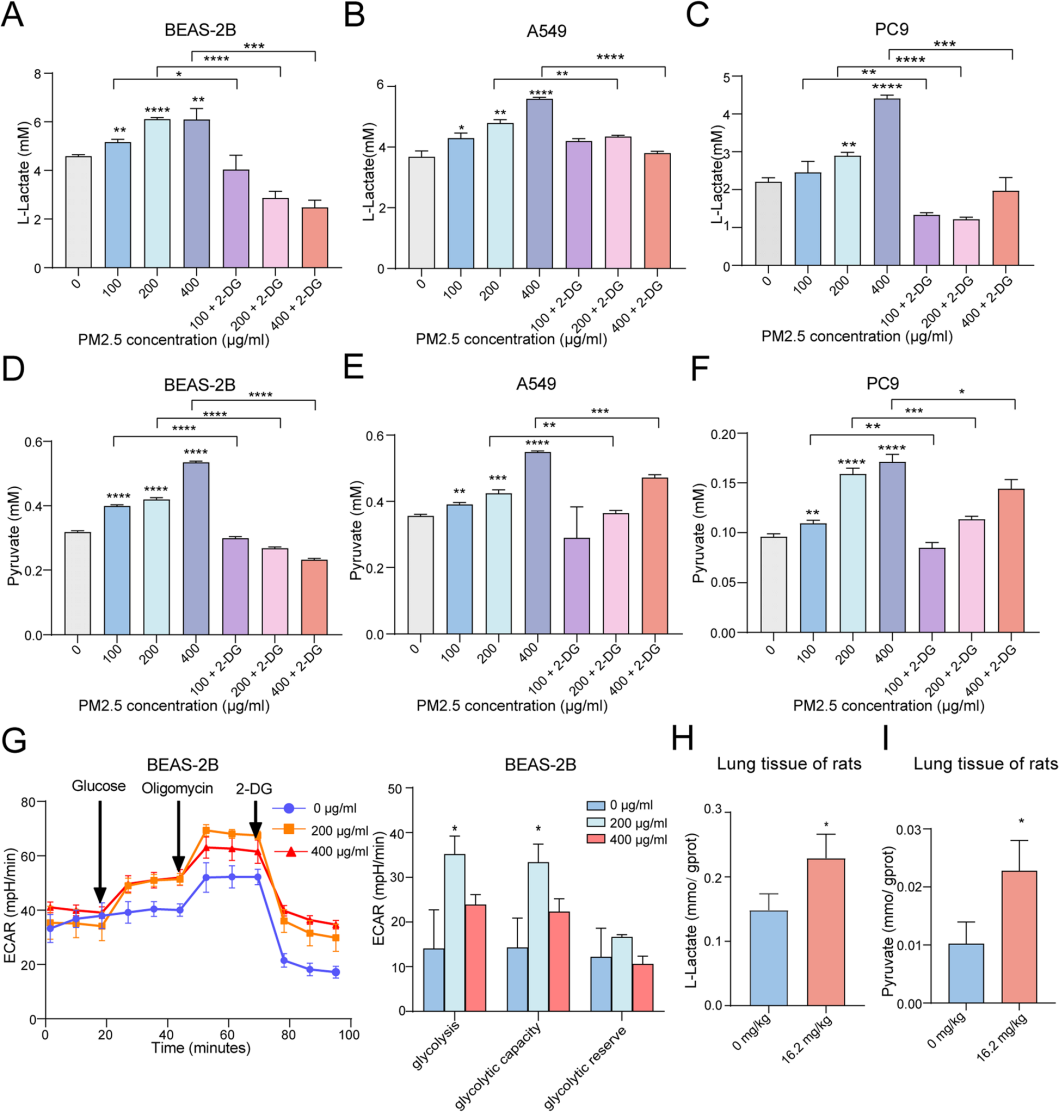
PM2.5 enhances glycolysis metabolism in vitro and in vivo. **A** PM2.5 enhanced L-lactate production in BEAS-2B cells, while 2-DG pretreatment reduced the generation of lactate in PM2.5-treated cells. **B** PM2.5 augmented L-lactate release from A549 cells in a dose response manner. **C** PM2.5 enhanced L-lactate production in PC9 cells. **D** PM2.5 increased pyruvate generation in BEAS-2B cells, and 2-DG pretreatment decreased pyruvate production in PM2.5-treated cells. **E** PM2.5 up-regulated pyruvate levels in A549 cells. **F** PM2.5 increased pyruvate levels in PC9 cells. **G** PM2.5 promoted ECAR levels in BEAS-2B cells. **H** PM2.5 exposure enhanced the production of L-lactate in lung issues of rats. **I** PM2.5 exposure promoted the generation of pyruvate in lung issues of rats. *P < 0.05, **P < 0.01, ***P < 0.001

We stimulated BEAS-2B and NSCLC cells with PM2.5. We found that PM2.5 exposure induced glycolytic reprograming in the cells, as evidenced by increased production of L-lactate and pyruvate, the end products of glycolysis metabolism, in a dose-dependent manner (Fig. 2A-F). When cells were co-incubated with PM2.5 and glycolysis inhibitor 2-DG (2-Deoxy-D-glucose), the generations of both L-lactate and pyruvate from these cells were significantly decreased (Fig. 2A, D). The ECAR assay revealed that PM2.5 significantly promoted cell ECAR levels, including glycolysis under basal conditions, glycolytic capacity, and compensatory glycolysis (Fig. 2G), confirming the impact of PM2.5 on glycolysis metabolism. To assess the in vivo effect of PM2.5 on glycolysis, SD rats were intratracheally instilled with PM2.5 for 4 weeks (Fig. S5A). It was found that PM2.5 induced higher expression of Ki67 and γH2AX, markers of cell proliferation and carcinogenesis, in lung tissue of rats in dose-response manners (Fig. S5B, S5D, S5F), confirming that PM2.5 samples in this study has oncogenic functions. PM2.5 also repressed the expression levels of caspase-3, marker of cell apoptosis (Fig. S5C, S5E). Furthermore, the concentrations of L-lactate and pyruvate in lung tissues of PM2.5-exposed rats were higher than those in control rats (Fig. 2H-I). These results establish that PM2.5 could drive glycolysis reprograming in vitro and in vivo.

Acetyl-CoA is the central metabolic node connecting glycolysis and TCA cycle, deriving mainly from the oxidation of pyruvate and the β-oxidation of free fatty acids (FFA) and finally enters into TCA cycle [31]. To examine whether PM2.5 may influence TCA cycle, we quantified acetyl-CoA levels in PM2.5-exposed cells and lung tissues of rats and compared them with control samples. Unexpectedly, we found that PM2.5 exposure significantly reduced acetyl-CoA levels in BEAS-2B, NSCLC cells, and in lung tissues of rats (Fig. S6-A-D). Furthermore, OCR analyses showed that even though PM2.5 could inhibit acetyl-CoA production, OCR levels in PM2.5-exposed cells were not significantly different from that of control cells (Fig. S6E-G), suggesting that PM2.5 had no significant influence on TCA-cycle metabolism. Future experiments are needed to determine whether there is other compensatory pathway, under the condition of decreased acetyl-CoA, to maintain TCA cycle homeostasis following PM2.5 exposure.

### PM2.5 augments the expression of glycolytic gene DLAT in vitro and in vivo

**Fig. 3:**
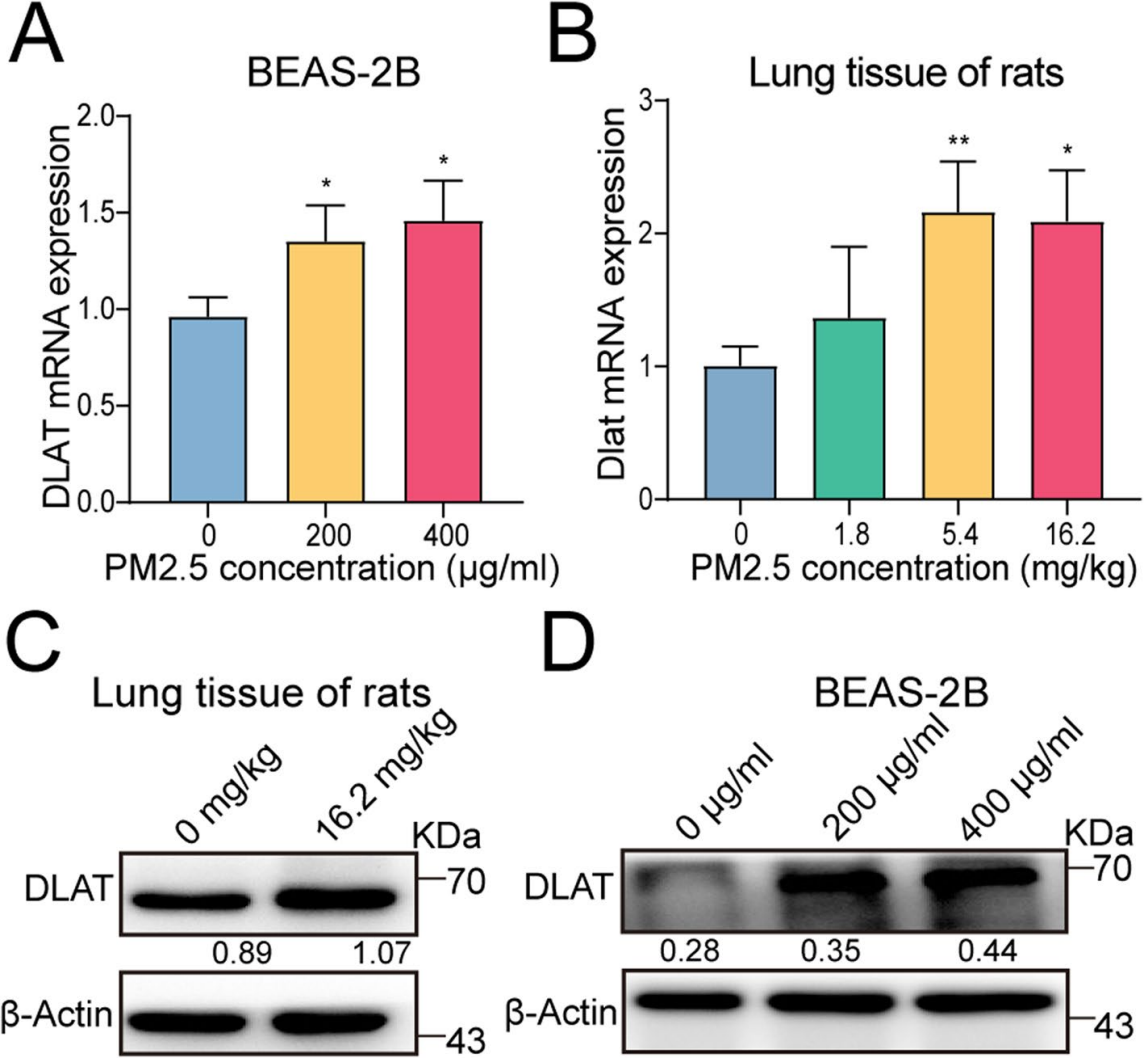
PM2.5 increases the expression of glycolytic gene DALT in vitro and in vivo. **A** PM2.5 exposure enhanced the expression level of DLAT gene in BEAS-2B cells. **B** PM2.5 inhalation up-regulated DLAT gene expression in lung tissues of rats. **C** PM2.5 exposure promoted DLAT protein expression in BEAS-2B cells. Numerical numbers denoted the ratio of integrated optical density (IOD) to β-actin. **D** PM2.5 exposure increased DLAT protein expression in lungs of rats. *P < 0.05, **P < 0.01

Since DLAT was the top DE-glycolytic gene in translation regulated by PM2.5, we further conducted in vitro and in vivo experiments to investigate the impact of PM2.5 on DLAT expression. Both qRT-PCR and western blot assays consistently showed that PM2.5 exposure could promote the expression levels of DLAT in a dose-response fashion in BEAS-2B cells (Fig. 3A, C). In agreement with these, in vivo analysis showed that inhalation of PM2.5 led to elevated expression of DLAT gene and protein in lung tissues of rats (Fig. 3B, D). Thus, our analyses point to DLAT as the primary target gene of PM2.5 exposure.

### DLAT increases glycolysis metabolism in NSCLC cells

**Fig. 4:**
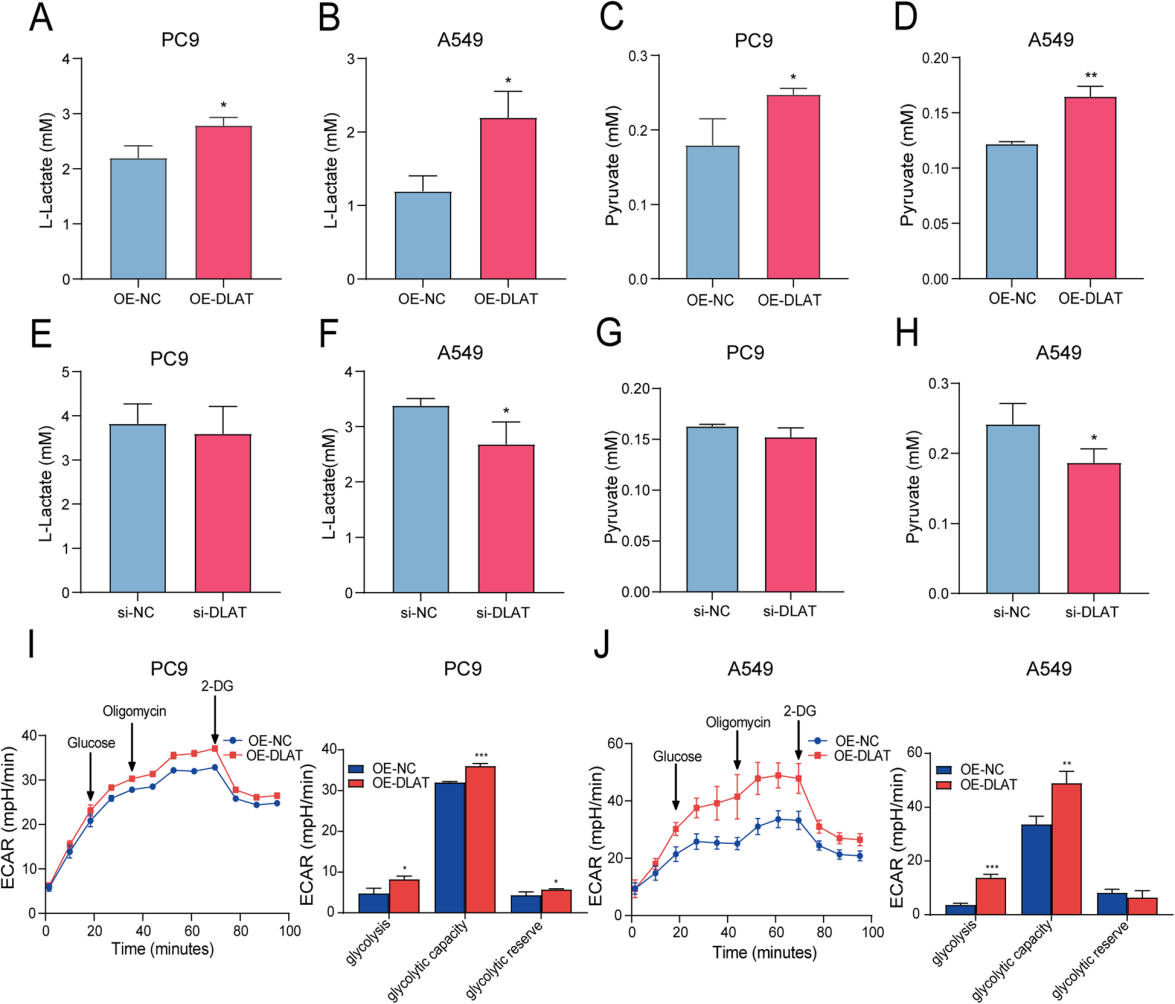
DLAT enhances glycolysis metabolism in NSCLC cells. **A** DLAT overexpression increased L-lactate production in PC9 cells. **B** DLAT up-regulation enhanced L-lactate production in A549 cells. **C** DLAT overexpression increased pyruvate release from PC9 cells. **D** Up-regulation of DLAT promoted pyruvate release from A549 cells. **E** Knockdown of DLAT suppressed L-lactate generation from PC9 cells. **F** Down-regulation of DLAT inhibited L-lactate generation from A549 cells. **G** Inhibition of DLAT expression decreased pyruvate production in PC9 cells. **H** Decreased DLAT expression reduced pyruvate production in A549 cells. **I** Overexpression of DLAT enhanced ECAR levels in PC9 cells. **J** Up-regulation of DLAT increased ECAR levels in A549 cells. *P < 0.05, **P < 0.01

To determine whether alterations of DLAT expression may affect glycolysis metabolism, DLAT-overexpression plasmids and siRNA against DLAT were transfected into In NSCLC cells (A549, PC9), respectively. The results showed that DLAT overexpression increased the extracellular levels of L-lactate and pyruvate in the medium of cell cultures (Fig. 4A-D). Conversely, knockdown of DLAT reduced the release of L-lactate and pyruvate from both PC9 and A549 cells (Fig. 4E-H). Moreover, the glycolytic stress test indicated that up-regulation of DLAT increased the ECAR levels, such as glycolysis under basal conditions, glycolytic capacity, and glycolysis reservation (Fig. 4I-J). Interestingly, overexpression of DLAT significantly repressed the production of acetyl-CoA from PC9 and A549 cells (Fig. S6C-D). But up-regulation of DLAT did not affect the OCR levels of PC9 and A549 cells (Fig. S6F-G). Taken together, these data suggest that PM2.5 accelerate the glycolysis metabolism of NSCLC cells through activating the expression of DLAT.

### DLAT promotes the malignancy of NSCLC cells

**Fig. 5:**
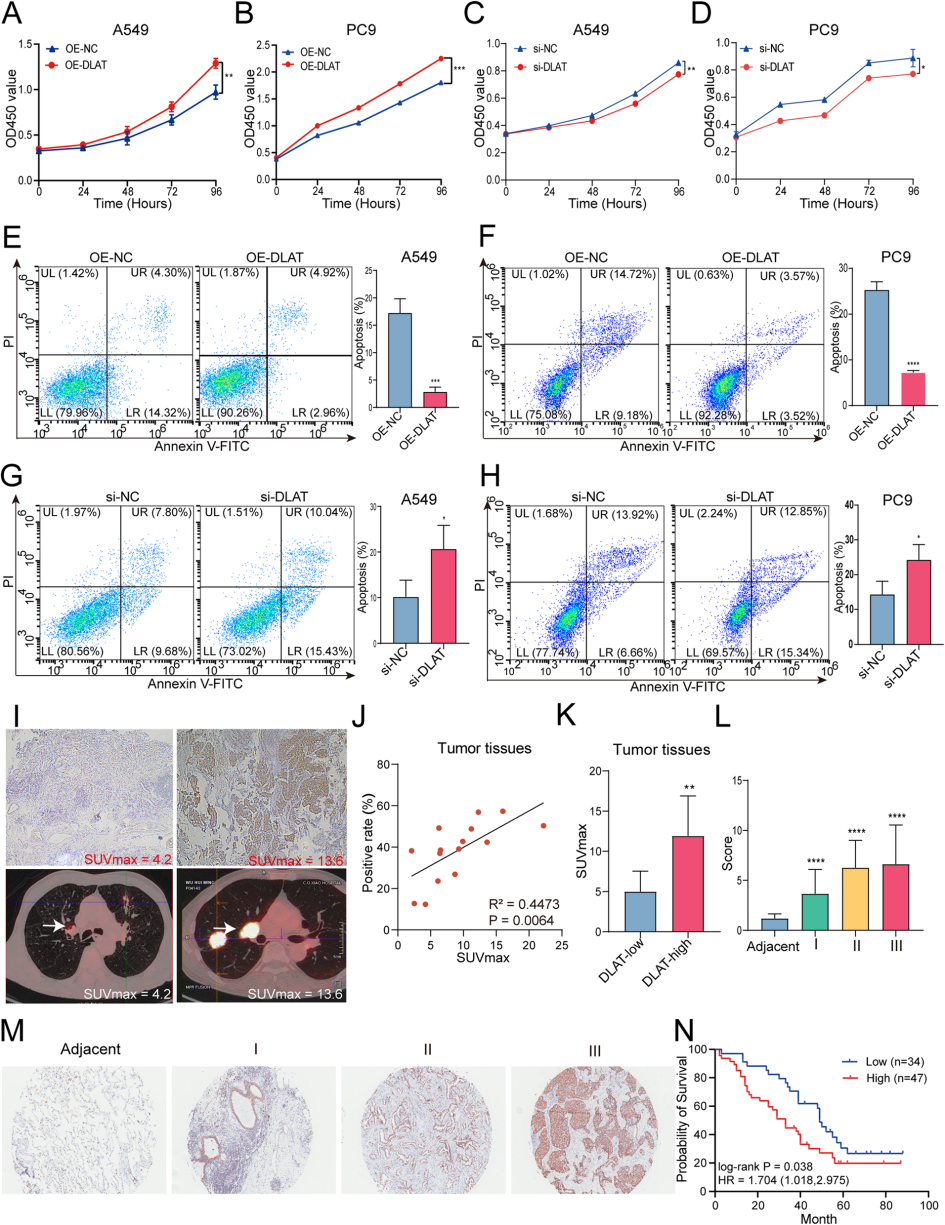
DLAT promotes the malignancy of NSCLC cells and correlates with glucose metabolism and poor prognosis in NSCLC patients. **A, B** Overexpression of DLAT promoted cell proliferation of NSCLC cells. **C, D** Knockdown of DLAT suppressed cell proliferation of A549 and PC9 cells. **E, F** Up-regulation of DLAT reduced apoptosis rate of PC9 and A549 cells. **G, H** Depletion of DLAT increased apoptosis rate of NSCLC cells. **I** Representative ^18^F-FDG PET/CT images in patients with NSCLC tumors exhibiting low or high expression of DLAT. **J** The expression levels (% of positive cells) of DLAT in tumor tissues were positively correlated with the SUVmax values in patients with NSCLC. **K** Analysis of SUVmax in the DLAT low and DLAT high groups. **L** DLAT expression levels in NSCLC tumor tissues increased as NSCLC progressed to more advanced stages. **M** Representative pictures showing that IHC signals of DLAT (brown staining) was increased along with the tumor stages of NSCLC. **N** Kaplan-Meier analysis showed that elevated expression of DLAT was associated with poorer overall survival (OS) in NSCLC patients. Data shown are mean ± SD. *P < 0.05, **P < 0.01, ***P < 0.001

We further explored the biological significance of DLAT in NSCLC. We transfected DLAT-overexpression plasmids or empty vectors into A549 and PC9 cells and the expression of DLAT was confirmed by RT-PCR assays (Fig. S4A-D). We then performed CCK8 assays to assess the effect of DLAT on NSCLC cell growth. We found that higher expression of DLAT significantly increased cell viability in both A549 and PC9 cells (Fig. 5A-B). While knockdown of DLAT with si-RNA led to decreased cell proliferation in A549 and PC9 cells (Fig. 5C-D). These observations suggested that DLAT may function as an oncogene in NSCLC.

To investigate whether DLAT may enhance NSCLC cell growth by influencing apoptosis, we conducted apoptosis assay by flow cytometry. As shown in Fig. 5E-F, both early and late apoptotic cell populations were significantly decreased in A549 and PC9 cells with over-expressing DLAT (all *P* values < 0.001) compared with control cells (Fig. 5E-F). In contrast, knockdown of DLAT significantly increased the rate of apoptotic cells (Fig. 5G-H). Together, these data indicate that up-regulation of DLAT inhibits NSCLC cell apoptosis.

### Up-regulation of DLAT is associated with SUVmax values of PET/CT scan and poorer prognosis in patients with NSCLC

To assess the clinical relevance of DLAT expression in patients with NSCLC, we analyzed the relationship between DLAT protein expression levels in NSCLC tumor tissues and the SUVmax value, a marker of tumor glycolysis metabolism detected by PET/CT (^18^F-FDG, 18F-fluorodeoxyglucose) scan, in patients with NSCLC (Supplementary Table S9). The results showed that tumors with high DLAT expression (IHC staining) exhibited higher SUVmax values than tumors with low DLAT expression (Fig. 5I). The expression levels of DLAT in tumors were positively correlated with the SUVmax values (Fig. 5 J). Furthermore, the SUVmax values were significantly higher in patients whose tissue samples exhibited a higher intensity of DLAT staining (Fig. 5 K). Together, these results indicated that DLAT expression levels are associated with^18^F-FDG uptake in NSCLC.

To evaluate the clinical significance of DLAT expression with the outcome in patients with NSCLC, we performed immunohistochemistry (IHC) assay on tissue-microarray (TMA) using tissue samples from NSCLC patients. We found that the DLAT expression scores of tumor tissue were significantly higher than that in adjacent normal tissues and significantly related to tumor grade: tumors with higher grades had higher expression levels of DLAT (Fig. 5 L). Fig. 5 M showed the representative images of the IHC results, indicating that DLAT expression was up-regulated in cancer tissues compared with adjacent normal lung tissues. Using the median score as the cutoff, we found that higher DLAT expression in NSCLC was significantly associated with lower overall survival in patients with NSCLC (Fig. 5 N). Finally, Cox proportional hazards analysis also revealed that higher DLAT expression level was independently associated with reduced survival of NSCLC patients (*P* = 0.017). In support of this, analyses in TCGA dataset also showed that DLAT was up-regulated in NSCLC and other types of cancers and was associated with worse survival outcomes of NSCLC patients (Fig. S7A-F). Thus, the dichotomized value of DLAT expression is an independent predictor for prognosis of NSCLC patients.

### PM2.5 enhances the expression of eIF4E which increases the translation of DLAT in polysomes

It has been known that most mRNAs in eukaryotic cells employ a cap-dependent mechanism to initiate translation [32], during which the 5′ end of mRNAs is modified with a m^7^Gppp cap structure that is recognized by the eukaryotic translation initiation factor 4 (eIF4E) [33]. eIF4E binds to the mRNA 5′ cap and stimulates the translation of a subset of mRNAs (“eIF4E-sensitive”) [34]. While mRNAs that have short and structured 5’UTRs are usually not eIF4E-sensitive and often encode for housekeeping genes such as *GAPDH* and *β-actin* [35]. To evaluate whether DLAT mRNA was eIF4E-sensitive, we analyzed the secondary structure and the 5’UTR length of DLAT, using the NCBI database and the RNAfold web server. It was found that the length of DLAT 5’UTR is 644 base pair (bp), which is much longer than that of GAPDH (102 bp) and β-actin (84 bp). Moreover, that the 5’UTR of DLAT was fold into a more complex and stable secondary structure featured by multiple hydrogen bonding between paired bases, compared with that of GAPDH and β-actin (Fig. S8E). In addition, the minimum free energy level of the 5’UTR of DLAT (− 235.60 kcal/mol) was much higher than that of GAPDH and β-actin (− 42.32 and − 15.92 kcal/mol respectively), and similar to that of eIF4E-sensitive mRNAs such as MYC and cyclin D1 (Fig. S8A-E). Thus, it is likely that the translation of DLAT was sensitive to eIF4E expression levels in the cell.

**Fig. 6:**
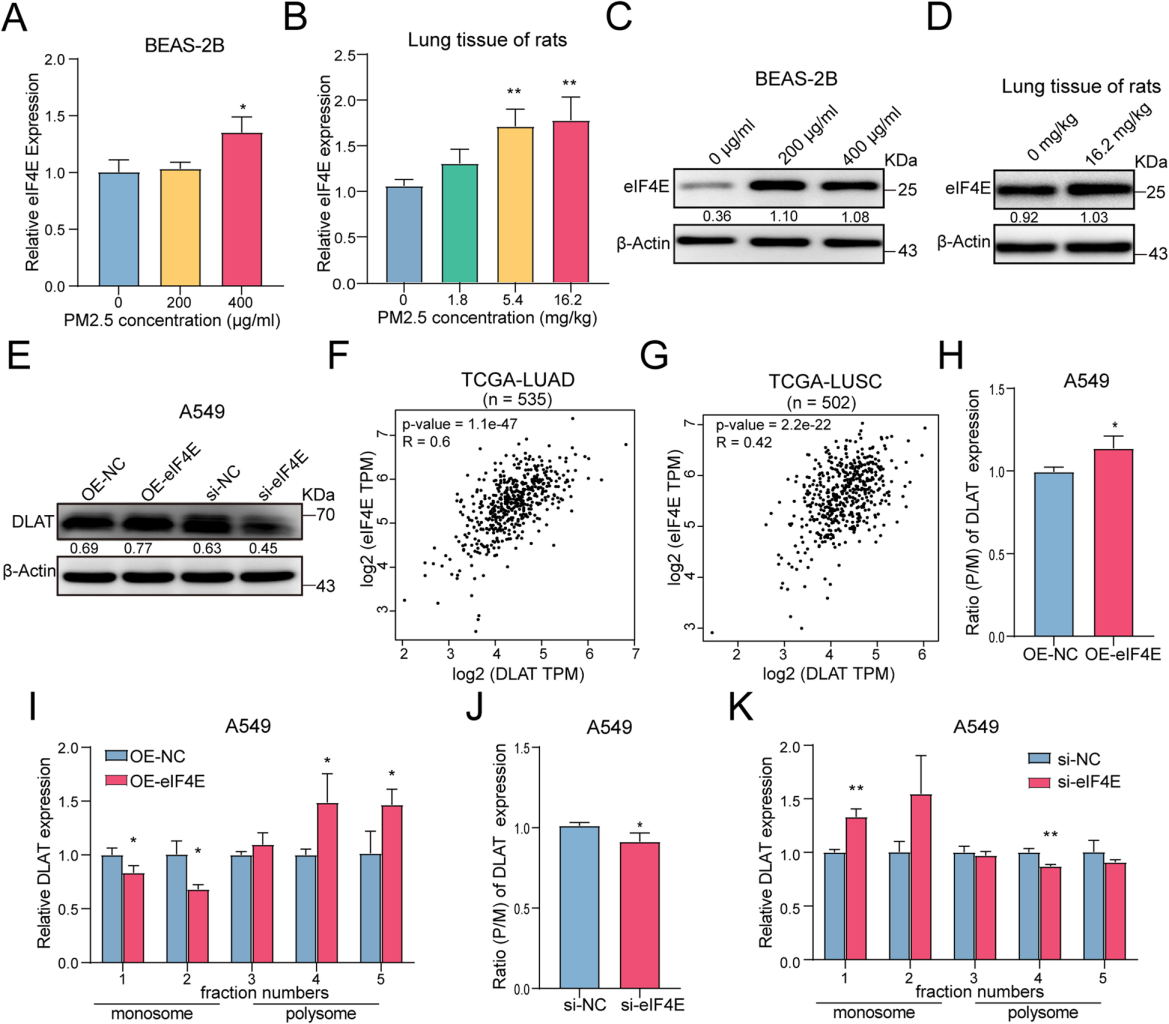
PM2.5 activates the expression of eIF4E that subsequently increases the translation of DLAT in polysomes. **A** PM2.5 enhanced eIF4E gene expression in BEAS-2B cells in a dose-response manner. **B** PM2.5 increased eIF4E gene expression in lung tissues of rats. **C** PM2.5 up-regulated eIF4E protein expression in BEAS-2B cells. **D** PM2.5 increased eIF4E protein expression in lung tissues of rats. **E** Up-regulation of eIF4E increased the expression of DLAT protein and knockdown of eIF4E decreased DLAT protein expression. **F** The expression level of eIF4E was positively correlated with that of DLAT in LUAD tumor tissues (TCGA datasets). **G** Correlation between eIF4E and DLAT expression levels in LUSC (TCGA datasets). **H** Overexpression of eIF4E increased the polysome to monosome (P/M) ratio of DLAT mRNA expression, suggesting an increase of DLAT translation initiation. **I** Up-regulation of eIF4E increased the abundance of DLAT mRNA in polysome but reduced DLAT expression in monosome in A549 cells. **J** Knockdown of eIF4E decreased the polysome to monosome ratio of DLAT mRNA expression, indicating a reduction of DLAT translation initiation. **K** Downregulation of eIF4E decreased the abundance of DLAT mRNA in polysome but enhanced DLAT expression in monosome in A549 cells. *P<0.05, **P<0.01

To investigate whether PM2.5 promotes an eIF4E-mediated DLAT translation, we firstly assessed the impact of PM2.5 exposure on eIF4E expression. It turned out that PM2.5 significantly elevated the expression of both eIF4E gene and protein (Fig. 6A-D). We next sought to determine whether DLAT translation was eIF4E-dependent. We found that up-regulation of eIF4E increased DLAT expression, while knockdown of eIF4E decreased DLAT expression in A549 cells (Fig. 6E). Similarly, analyses of TCGA dataset also indicated that eIF4E expression levels were positively correlated with that of DLAT in NSCLC tumor tissues (Fig. 6F-G). By comparing the expression levels of eIF4E and DLAT in monosome and polysome fractions in eIF4E-overexpression and eIF4E-knockdown cells, we found that overexpression of eIF4E increased the expression levels of DLAT in the heavy fraction of polysomes while knockdown of eIF4E resulted in decreased DLAT signals in the heavy fraction of polysomes (Fig. 6I). Importantly, overexpression of eIF4E induced an increased polysome to monosome expression ratio of DLAT, indicating an increase in translation initiation of DLAT (Fig. 6H). In contrast, down-regulation of eIF4E significantly decreased translation initiation, as evidenced by reduced polysome to monosome expression ratio of DLAT (Fig. 6 J), and increasing expression of DLAT in monosomes and decreasing expression of DLAT in polysomes (Fig. 6 K). Thus, our data provided evidence that PM2.5-induced up-regulation of eIF4E could account for the increased DLAT protein being translated.

### PM2.5 activates the expression of transcription factor Sp1 that up-regulates DLAT transcription

**Fig. 7:**
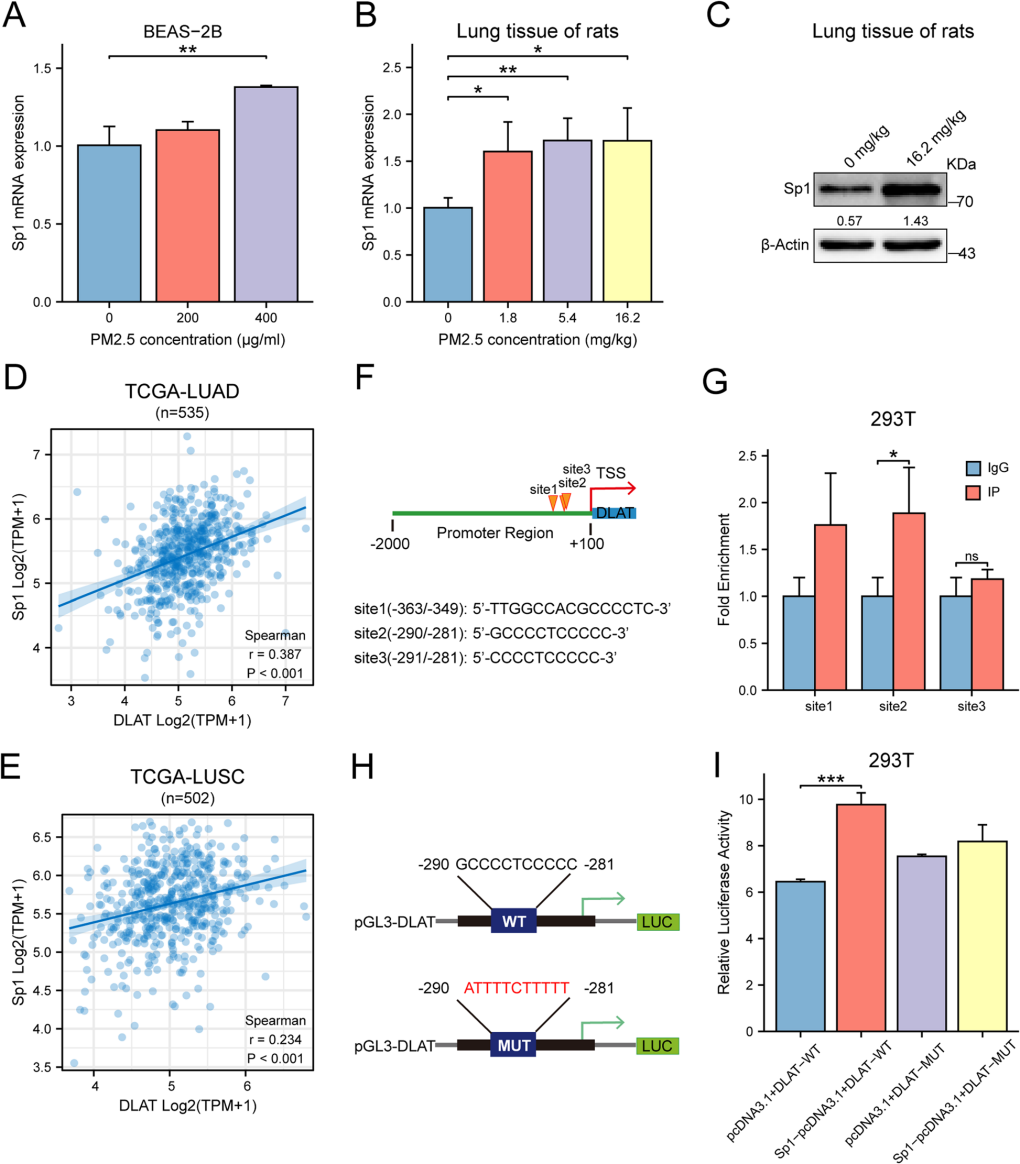
PM2.5 activates the expression of transcription factor Sp1 which enhances the transcription of DLAT. **A** PM2.5 promoted Sp1 expression in a dose-response manner in BEAS-2B cells. **B** PM2.5 enhanced the expression of Sp1 in lung tissues of rats. **C** PM2.5 increased the expression of Sp1 protein in lung tissues of rats. **D, E** The expression level of Sp1 was positively correlated with that of DLAT in LUAD (lung adenocarcinoma) and LUSC (lung squamous carcinoma) in TCGA dataset. **F** In silico analysis identified Sp1 putative binding sites in the promoter region of DLAT. **G** ChIP assay showed the binding of Sp1 with DLAT promoter region. **H** Potential binding sequences for Sp1 were found in the DLAT promoter region. **I** Luciferase reporter assay verified that the binding of Sp1 with wild-type DLAT promoter significantly increased the luciferase activity. While binding of Sp1 with mutant DLAT promoter did not changes the intensity of luciferase signals. **J** Schematic view for the mechanisms of action of DLAT-mediated glycolysis reprograming in PM2.5-induced carcinogenesis. *P<0.05, **P<0.01, ***P<0.001.

As previous studies indicated that the expressions of several glycolytic genes were regulated by transcription factors (TF), we wondered whether DLAT expression could also be modified by transcription factor. Using the JASPAR database and its algorithms, we found that there were several putative binding sites in the promoter of DLAT for transcription factor Sp1 (Fig. 7A), suggesting that transcription of DLAT may be modified by Sp1.We then exposed BEAS-2B cells to PM2.5 and found that PM2.5 enhanced Sp1 expression in a dose-response manner (Fig. 7B). PM2.5 exposure also enhanced the expression of Sp1 mRNA and proteins in lung tissues of rats (Fig. 7C-D). Moreover, bioinformatics analysis showed that Sp1 expression levels were positively correlated with that of DLAT in tumor tissues in TCGA data set (Fig. 7E-F). We confirmed the binding of DLAT promoter with Sp1 by ChIP-PCR assay (Fig. 7G). We then constructed luciferase reporter vectors containing wild-type or mutated DLAT promoter sequences to assess the functional effect of Sp1 on DLAT transcription (Fig. 7H). Co-transfection of Sp1 vector with the wild-type DLAT promoter construct resulted in significantly higher luciferase activity than that of control vectors, while co-culture of mutated DLAT promoter vector with Sp1 plasmid did not influence the luciferase activity of DLAT promoter (Fig. 7I). These results indicated that DLAT was a direct transcriptional target of Sp1.

## Discussion

Aerobic glycolysis, also known as Warburg effect, has been recognized as a hallmark of cancer progression and metastasis [36]. However, whether higher rate of glycolysis is a cause or consequence of carcinogenesis remains to be elucidated [37]. Most prior studies in cancer-associated glycolysis have focused on the contribution of Warburg effect on proliferation and metastasis [38], but little is known about its roles in carcinogen-induced tumorigenesis. Although a previous study demonstrated that carcinogen arsenite enhanced glycolytic gene expression and suppressed the expression of TCA cycle-related genes in liver cells [39], there is limited direct evidence existent to support primary causal role of glycolysis in carcinogen exposure-associated carcinogenesis. In this study, we showed that PM2.5 promoted the glycolysis phenotypes but inhibited TCA metabolism. In contrast, treatment of PM2.5 with glycolysis inhibitor 2-DG diminished the levels of PM2.5-induced glycolysis. Thus, our study provides evidence suggesting that PM2.5-enhanced glycolysis reprogramming may be causally involved in PM2.5-induced carcinogenesis.

Integration analysis of RNA-seq and Ribo-seq data revealed that PM2.5-induced transcriptome profile was poorly correlated with that of translatome. This observation was in line with other reports in which gene expression profiles shared little similarity with translatome patterns when cells encountered extracellular stimuli [40, 41]. However, when DE-genes were analyzed at translation levels, we found that PM2.5 preferentially induced a translation efficiency shift towards glucose metabolism pathways, especially glycolysis-related pathways. Our findings support the notion of translational reprogramming, in which cells selectively activate translation of certain mRNAs in response to stress [42]. Such “adaptive translation” allows a cell to orchestrate rapid changes in protein synthesis and tailor newly synthesized proteins to ensure cell survival and to modify tumor cell behavior such as invasion and metastasis [43]. In general, selective translation modulates stress response and various human disease phenotypes [44]. In particular, selective translation of oncogenic mRNAs can promote cancer cell proliferation, metastasis and tumor expansion [18]. For example, the capacity of oncogenic signaling to enhance cell proliferation is directly associated with the induction of replicative stress and genomic instability during early stages of tumorigenesis [45]. How specific subsets of mRNAs are selectively translated in response to each stress is not well understood but may be associated with the secondary structure of a given mRNA [46]. eIF4E is the translation initiation factor and is rate limiting for transcription initiation. Prior studies have shown that over-expression of eIF4E facilitates the translation of mRNAs with long 5’UTR [47]. Up-regulation of the abundance and activity of eIF4E occurs widely in cancers and selectively up-regulates translation of certain mRNAs involved in survival, proliferation, and metastasis [48]. Indeed, 5’UTR structures in several oncogenic mRNAs have been associated with translation in an eIF4E dependent manner [18]. Importantly, these mRNAs typically display a greater requirement for eIF4E, often a result of increased secondary structure close to the cap [49]. Here, we discovered that PM2.5 enhanced the expression levels of eIF4E and DLAT. Bioinformatics analysis showed that the secondary structure of DLAT mRNA was eIF4E-sensitive. We also found that expression levels of ribosomal eIF4E was positively correlated with that of ribosomal DLAT. In particular, up-regulation of ribosomal eIF4E increased the polysome/monosome ratio of DLAT expression, indicating increased translation of DLAT. Our study therefore uncovers a novel mechanism that PM2.5 up-regulates glycolysis through eIF4E-mediated selective translation of DLAT.

Pyruvate, catalyzed by the pyruvate dehydrogenase complex (PDC), is a key glycolytic metabolite that could either be reductively metabolized into lactate for glycolysis or be oxidatively converted into acetyl-CoA for TCA cycle. The human PDC is consisting of three catalytic subunits: pyruvate dehydrogenase (E1), dihydrolipoyl transacetylase (E2, DLAT), dihydrolipoyl dehydrogenase (E3), and one structural subunit (E3-binding protein, E3BP). E1 and E2 generate acetyl-coenzyme A, whereas the E3 performs redox recycling [50]. As the subunit E2 of PDC, DLAT was previously considered to be able to catalyze the breakdown of pyruvate into acetyl-coA, the start material for TCA cycle, thereby facilitating TCA cycle metabolism [51]. Thus, DLAT was intuitively supposed to be downregulated in cancer if the Warburg effect is assumed [52]. In this study, we showed that PM2.5 enhanced DLAT expression and reduced acetyl-CoA release. Moreover, we found that overexpression of DLAT suppressed acetyl-CoA production but had little impact on OCR levels. The reasons that DLAT did not enhance acetyl-CoA and had no significant effect on TCA cycle metabolisms were unclear, but may be due to the fact that PM2.5 exposure did not influence the expressions of E1 (PDHA1) and E3 (DLD) genes (Table S2). Because catalyzation of pyruvate requires the cooperation of E1, E2, and E3 of PDC, it is unlikely that DLAT alone could convert pyruvate into acetyl-CoA without the support of E1 and E3 components [53].

Our data showed that over-expression of DLAT significantly increased the production of L-lactate and pyruvate. In addition, we demonstrated that up-regulation of DLAT significantly enhanced the levels of ECAR. Lastly, we found that expression levels of DLAT in tumor tissues were positively correlated with SUVmax values, confirming that DLAT augmented glycolysis metabolism in NSCLC. Collectively, our data strongly suggested that the main function of DLAT was to promote glycolysis metabolism, rather than to facilitate TCA cycle. The molecular mechanism that how DLAT enhances glycolysis metabolism is unknown and beyond the scope of the present study. Further more detailed experiments are required to elucidate the regulatory mechanisms of DLAT on glycolysis. However, our findings on the oncogenic effects of DLAT in NSCLC were in agreement with the concept of Warburg effect in which cancer cells produced more lactate from pyruvate but converted less pyruvate into acetyl-CoA compared with their normal counterparts [54]. Future experiments are needed to determine whether there is other compensatory pathway, under the condition of decreased acetyl-CoA, to maintain TCA cycle homeostasis following PM2.5 exposure.

Transcription factors play a direct role in regulating the Warburg effect [55]. For instance, transcription factor SIX1 increased the expression of glycolytic genes and promoted tumor growth [56]. Hypoxia-inducible factor-1α (HIF-1α) enhanced aerobic glycolysis by binding to hypoxia-responsive elements of glycolytic gene promoters in cancer cells [57]. However, the mechanism underlying the induction of transcription factors in glycolysis switch remains to be elucidated. Transcription factor Sp1 (specificity protein 1) belongs to the Sp transcription factor family containing zinc finger DNA binding domains, which bind to the GC-rich promoter element, and is involved in the regulation of gene expression [58]. Sp1 might contribute to tumorigenesis through regulating transcription of oncogenes and tumor suppressor genes [58]. Many human cancer cells exhibited higher levels of Sp1 expression, and the levels of Sp1 expression was associated with the state of a tumor and poor clinical prognosis [59]. Furthermore, down-regulation of Sp1 inhibited tumor formation, cancer cell growth, and cell metastasis [60]. In this study, we discovered that PM2.5 induced Sp1 up-regulation in vitro and in vivo. We found that Sp1 bound to the promoter of DLAT gene, leading to increased DLAT transcription, which further enhanced glycolysis and promoted malignant phenotypes of NSCLC cells. Altogether, our results reveal a novel mechanism by which PM2.5 promotes glycolysis via Sp1-mediated transcription regulation of DLAT in NSCLC cells.

## Conclusions

**Fig. 8:**
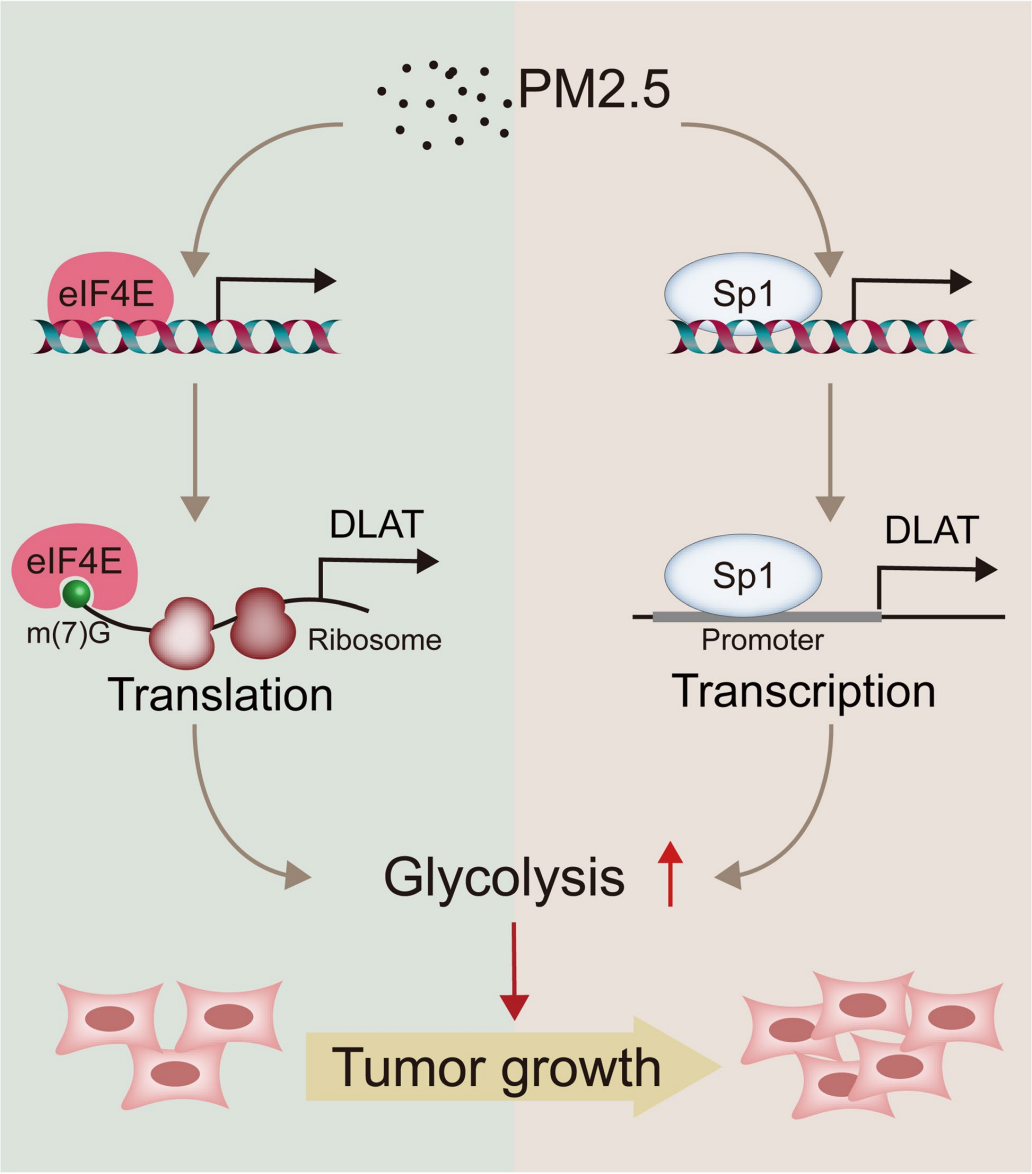
Schematic view for the mechanisms of action of DLAT-mediated glycolysis reprograming in PM2.5-induced carcinogenesis

This study demonstrates for the first time that PM2.5-induced glycolysis may be causally involved in the carcinogenesis of NSCLC. In addition, we reveal that DLAT is not only a novel glycolytic gene but also a novel oncogene in NSCLC. We also uncover a dual regulatory mechanism of Sp1-DLAT and eIF4E-DLAT axes by which PM2.5 promotes glycolysis and NSCLC tumorigenesis (Fig. 8). These findings expand our knowledge on the mechanisms of PM2.5-induced carcinogenesis and suggest that targeting DLAT-mediated glycolysis pathway may represent a potential strategy for NSCLC treatment.

## Supplementary Information

**Additional file 1: Fig. S1**. Effects of PM2.5 exposure doses on cell cytotoxicity in BEAS-2B cells. ****P < 0.0001.

**Additional file 2: Fig. S2.** RNA-seq and Ribo-seq on BEAS-2B cells following exposure to PM2.5. **(A)** RNA-seq and Ribo-seq reads were highly reproducible across biological replicates, respectively. **(B)** Size distribution and relative abundance of ribosome footprints reads between PM2.5-exposed and non-exposed BEAS-2B cells. **(C)** Representative bar plots showing the peptidyl-site (P-site) position derived from Ribo-seq reads across the first 50 nt and last 50 nt of open reading frames (ORFs). **(D)** The proportion of different categories of ORFs detected in our study. Annotated, annotated open reading frames (ORFs) for proteins; uORFs, upstream ORF; Dorf, downstream ORF; overlapped, uORF/dORF overlapped with main ORF; novel_PCGs, novel ORFs from protein-coding genes; novel_NonPCGs, novel ORFs from non-protein-coding genes.

**Additional file 3: Fig. S3**. Number of DE-genes and the abundance of DE-genes in transcriptome, translatome, and TE following PM2.5 exposure. **(A, B, C)** Numbers of significantly up- or down-regulated transcripts in transcriptome, translatome, and TE. **(D, E, F)** Relative log2 fold-change (FC) in transcript abundance between PM2.5-exposed cells versus control cells in transcriptome, translatome, and TE. Transcripts significantly up-regulated, down-regulated, or non-significant change are colored in red, blue, and light grey, respectively.

**Additional file 4: Fig. S4**. Transfection efficiencies of DLAT and eIF4E overexpression vector and corresponding si-RNAs. **(A)** Transfection efficiency of DLAT-overexpression vector in A549 cells. **(B)** knockdown efficiency of si-DLAT in A549 cells. **(C)** Transfection efficiency of eIF4E-overexpression vector in A549 cells. **(D)** Inhibition efficiency si-eIF4E in A549 cells. *P < 0.05; **P < 0.01; ***P < 0.001.

**Additional file 5: Fig. S5**. PM2.5 promotes the expression levels of cancer-associated markers in lung tissue of rats. **(A)** Schematic view of animal experiments. (**B)** Representative IHC staining images of the expression of Ki-67 protein, a marker of cell proliferation, in lung tissues of rats. **(C)** Representative pictures of IHC staining of caspase-3, a marker of apoptosis, in lung tissues of rats. **(D)** PM2.5 exposure increased Ki-67 expression levels in lung tissues of rats in a dose-response manner. **(E)** PM2.5 exposure decreased the expression levels of caspase-3 in lung tissues of rats. **(F)** PM2.5 enhanced the expression levels of γ-H2Ax, a marker of DNA damage and carcinogenesis, in lung tissues of rats in a dose-dependent manner. *P < 0.05; **P < 0.01.

**Additional file 6: Fig. S6**. PM2.5 and DLAT decrease acetyl-CoA production but do not alter TCA cycle metabolism level. **(A)** PM2.5 exposure inhibited acetyl-CoA generation in BEAS-2B cells. **(B)** There was a trend of decrease in acetyl-CoA levels in PM2.5-exposed lung tissues of rats. **(C, D)** Overexpression of DLAT suppressed acetyl-CoA production from NSCLC cells. **(E)** PM2.5 exposure did not significantly alter OCR levels in BEAS-2B cells. **(F, G)** Up-regulation of DLAT did not influence the OCR levels in NSCLC cells. **(H)** Schematic function of pyruvate in glycolysis metabolism and TCA cycle. *P < 0.05; **P < 0.01; ***P < 0.001.

**Additional file 7: Fig. S7**. Clinical significance of DLAT expression in NSCLC in TCGA datasets. **(A)** DLAT was up-regulated in tumor tissues of LUAD (lung adenocarcinoma). **(B)** The expression level of DLAT in LUSC (lung squamous cell carcinoma) tissue was higher than that in normal tissues. **(C)** The expression of DLAT protein in LUAD was higher than that in normal tissues. **(D)** Higher expression level of DLAT in tumor tissues was associated with worse overall survival of NSCLC. **(E)** Higher expression level of DLAT in tumor tissues was associated with worse progression free survival of NSCLC patients. **(F)** DLAT was up-regulated in other types of primary tumors.

**Additional file 8: Fig. S8**. Secondary structures in 5′-UTR of representative genes. **(A)** *β-actin*. **(B)** *GAPDH*. **(C)** *Cyclin D1*. **(D)** *MYC*. **(E)** *DLAT*. The structures were predicted using the RNAFold software. The minimum free energy was indicated.

**Additional file 9: Table S1**. QC data of Ribo-seq and RNA-seq.

**Additional file 10: Table S2**. Differentially expressed mRNAs regulated by PM2.5 in BEAS-2B cells.

**Additional file 11: Table S3**. Differentially expressed ribosome-associated mRNAs regulated by PM2.5 in BEAS-2B cells.

**Additional file 12: Table S4**. Genes with significant change in translation efficiency (TE) in PM2.5-exposed BEAS-2B cells.

**Additional file 13: Table S5**. Enrichment results of differentially expressed mRNAs by KEGG analysis.

**Additional file 14: Table S6**. Enrichment results of differentially translated genes by KEGG analysis.

**Additional file 15: Table S7**. Enrichment results of genes with significant translation efficiency (TE) changes by KEGG analysis.

**Additional file 16: Table S8**. Results of gene set enrichment analysis (GSEA) on differentially expressed genes in translatome.

**Additional file 17: Table S9**. Characteristics of NSCLC patients recruited for analysis of SUVmax values of PCT-CT and DLAT IHC staining.

**Additional file 18: Table S10.** Primers and antibodies used in this study.

## Data Availability

The RNA-seq and Ribo-seq data of this study have been deposited in the NCBI Gene Expression Omnibus (GEO) under accession number GSE182201.

## Abbreviations

LCNS: Lung cancer in never smokers
NSCLC: Non-small cell lung cancer
PM: Particulate matter
IARC: International Agency for Research on Cancer
Ribo-seq: Ribosome sequencing
RNA-seq: RNA sequencing
DE: Differentially expressed
GSEA: Gene set enrichment analysis
ECAR: Extracellular acidification rate
OCR: Oxygen consumption rates
2-DG: 2-Deoxy-D-glucose
TCA cycle: Tricarboxylic acid cycle
qRT-PCR: Quantitative real time PCR
^18^F-FDG: 18F-fluorodeoxyglucose
PET/CT: Positron Emission Tomography/computed tomography
IHC: Immunohistochemistry
TMA: Tissue-microarray
eIF4E: Eukaryotic translation initiation factor 4
Sp1: Specificity protein 1
5’UTR: The 5′′untranslated region
TF: Transcription factors
PDC: Pyruvate dehydrogenase complex
ChIP: Chromatin immunoprecipitation

## References

[1] Kang HR, Cho JY, Lee SH, Lee YJ, Park JS, Cho YJ, Yoon HI, Lee KW, Lee JH, Lee CT (2019). Role of low-dose computerized tomography in lung cancer screening among never-smokers. J Thorac Oncol.

[2] Casal-Mouriño A, Valdés L, Barros-Dios JM, Ruano-Ravina A (2019). Lung cancer survival among never smokers. Cancer Lett.

[3] Smolle E, Pichler M (2019). Non-smoking-associated lung cancer: a distinct entity in terms of tumor biology, patient characteristics and impact of hereditary cancer predisposition. Cancers (Basel).

[4] Lee YJ, Kim JH, Kim SK, Ha SJ, Mok TS, Mitsudomi T, Cho BC (2011). Lung cancer in never smokers: change of a mindset in the molecular era. Lung Cancer.

[5] Fajersztajn L, Veras M, Barrozo LV, Saldiva P (2013). Air pollution: a potentially modifiable risk factor for lung cancer. Nat Rev Cancer.

[6] Puett RC, Hart JE, Yanosky JD, Spiegelman D, Wang M, Fisher JA, Hong B, Laden F (2014). Particulate matter air pollution exposure, distance to road, and incident lung cancer in the nurses’ health study cohort. Environ Health Perspect.

[7] Raaschou-Nielsen O, Andersen ZJ, Beelen R, Samoli E, Stafoggia M, Weinmayr G, Hoffmann B, Fischer P, Nieuwenhuijsen MJ, Brunekreef B (2013). Air pollution and lung cancer incidence in 17 European cohorts: prospective analyses from the European study of cohorts for air pollution effects (ESCAPE). Lancet Oncol.

[8] Coleman NC, Burnett RT, Ezzati M, Marshall JD, Robinson AL, Pope CA (2020). Fine particulate matter exposure and cancer incidence: analysis of SEER cancer registry data from 1992-2016. Environ Health Perspect.

[9] Guo H, Li W, Wu J (2020). Ambient PM2.5 and annual lung cancer incidence: a Nationwide study in 295 Chinese counties. Int J Environ Res Public Health.

[10] Yin P, Brauer M, Cohen A, Burnett RT, Liu J, Liu Y, Liang R, Wang W, Qi J, Wang L (2017). Long-term fine particulate matter exposure and nonaccidental and cause-specific mortality in a large national cohort of Chinese men. Environ Health Perspect.

[11] Turner MC, Krewski D, Pope CA, Chen Y, Gapstur SM, Thun MJ (2011). Long-term ambient fine particulate matter air pollution and lung cancer in a large cohort of never-smokers. Am J Respir Crit Care Med.

[12] Eckel SP, Cockburn M, Shu YH, Deng H, Lurmann FW, Liu L, Gilliland FD (2016). Air pollution affects lung cancer survival. Thorax..

[13] Loomis D, Grosse Y, Lauby-Secretan B, El Ghissassi F, Bouvard V, Benbrahim-Tallaa L, Guha N, Baan R, Mattock H, Straif K (2013). The carcinogenicity of outdoor air pollution. Lancet Oncol.

[14] Clark CR, DuRose W, Starr TK (2019). Cancer gene discovery: past to present. Methods Mol Biol.

[15] Schwanhäusser B, Busse D, Li N, Dittmar G, Schuchhardt J, Wolf J, Chen W, Selbach M (2011). Global quantification of mammalian gene expression control. Nature..

[16] Brar GA, Weissman JS (2015). Ribosome profiling reveals the what, when, where and how of protein synthesis. Nat Rev Mol Cell Biol.

[17] Vogel C, Marcotte EM (2012). Insights into the regulation of protein abundance from proteomic and transcriptomic analyses. Nat Rev Genet.

[18] Truitt ML, Ruggero D (2016). New frontiers in translational control of the cancer genome. Nat Rev Cancer.

[19] Del Llano E, Masek T, Gahurova L, Pospisek M, Koncicka M, Jindrova A, Jansova D, Iyyappan R, Roucova K, Bruce AW (2020). Age-related differences in the translational landscape of mammalian oocytes. Aging Cell.

[20] Lee DC, Choi H, Oh JM, Lee J, Lee J, Lee HY, Kang JY (2020). Urban particulate matter regulates tight junction proteins by inducing oxidative stress via the Akt signal pathway in human nasal epithelial cells. Toxicol Lett.

[21] Das A, Habib G, Vivekanandan P, Kumar A (2021). Reactive oxygen species production and inflammatory effects of ambient PM(2.5) -associated metals on human lung epithelial A549 cells “one year-long study”: the Delhi chapter. Chemosphere..

[22] Zhou W, Yuan X, Zhang L, Su B, Tian D, Li Y, Zhao J, Wang Y, Peng S (2017). Overexpression of HO-1 assisted PM2.5-induced apoptosis failure and autophagy-related cell necrosis. Ecotoxicol Environ Saf.

[23] Kim HR, Cho HS, Shin DY, Chung KH (2017). Novel approach to study the cardiovascular effects and mechanism of action of urban particulate matter using lung epithelial-endothelial tetra-culture system. Toxicol in Vitro.

[24] Niu BY, Li WK, Li JS, Hong QH, Khodahemmati S, Gao JF, Zhou ZX (2020). Effects of DNA damage and oxidative stress in human bronchial epithelial cells exposed to PM(2.5) from Beijing, China, in winter. Int J Environ Res Public Health.

[25] Gawda A, Majka G, Nowak B, Śróttek M, Walczewska M, Marcinkiewicz J (2018). Air particulate matter SRM 1648a primes macrophages to hyperinflammatory response after LPS stimulation. Inflamm Res.

[26] Hetland RB, Cassee FR, Refsnes M, Schwarze PE, Låg M, Boere AJ, Dybing E (2004). Release of inflammatory cytokines, cell toxicity and apoptosis in epithelial lung cells after exposure to ambient air particles of different size fractions. Toxicol in Vitro.

[27] Zhang Y, Hu H, Shi Y, Yang X, Cao L, Wu J, Asweto CO, Feng L, Duan J, Sun Z (2017). ^1^H NMR-based metabolomics study on repeat dose toxicity of fine particulate matter in rats after intratracheal instillation. Sci Total Environ.

[28] Zhang J, Liu J, Ren L, Wei J, Duan J, Zhang L, Zhou X, Sun Z (2018). PM(2.5) induces male reproductive toxicity via mitochondrial dysfunction, DNA damage and RIPK1 mediated apoptotic signaling pathway. Sci Total Environ.

[29] Detterbeck FC, Boffa DJ, Kim AW, Tanoue LT (2017). The eighth edition lung cancer stage classification. Chest..

[30] Kampen KR, Fancello L, Girardi T, Rinaldi G, Planque M, Sulima SO, Loayza-Puch F, Verbelen B, Vereecke S, Verbeeck J (2019). Translatome analysis reveals altered serine and glycine metabolism in T-cell acute lymphoblastic leukemia cells. Nat Commun.

[31] Chen L, Vasoya RP, Toke NH, Parthasarathy A, Luo S, Chiles E, Flores J, Gao N, Bonder EM, Su X (2020). HNF4 regulates fatty acid oxidation and is required for renewal of intestinal stem cells in mice. Gastroenterology..

[32] Jackson RJ, Hellen CU, Pestova TV (2010). The mechanism of eukaryotic translation initiation and principles of its regulation. Nat Rev Mol Cell Biol.

[33] Gross JD, Moerke NJ, von der Haar T, Lugovskoy AA, Sachs AB, McCarthy JE, Wagner G (2003). Ribosome loading onto the mRNA cap is driven by conformational coupling between eIF4G and eIF4E. Cell..

[34] Hinnebusch AG, Ivanov IP, Sonenberg N (2016). Translational control by 5′-untranslated regions of eukaryotic mRNAs. Science..

[35] Uttam S, Wong C, Price TJ, Khoutorsky A (2018). eIF4E-dependent translational control: a central mechanism for regulation of pain plasticity. Front Genet.

[36] Ward PS, Thompson CB (2012). Metabolic reprogramming: a cancer hallmark even Warburg did not anticipate. Cancer Cell.

[37] Robey RB, Weisz J, Kuemmerle NB, Salzberg AC, Berg A, Brown DG, Kubik L, Palorini R, Al-Mulla F, Al-Temaimi R (2015). Metabolic reprogramming and dysregulated metabolism: cause, consequence and/or enabler of environmental carcinogenesis?. Carcinogenesis..

[38] Park JH, Pyun WY, Park HW (2020). Cancer metabolism: phenotype, signaling and therapeutic targets. Cells..

[39] Luo F, Zou Z, Liu X, Ling M, Wang Q, Wang Q, Lu L, Shi L, Liu Y, Liu Q (2017). Enhanced glycolysis, regulated by HIF-1α via MCT-4, promotes inflammation in arsenite-induced carcinogenesis. Carcinogenesis..

[40] Doroudgar S, Hofmann C, Boileau E, Malone B, Riechert E, Gorska AA, Jakobi T, Sandmann C, Jürgensen L, Kmietczyk V (2019). Monitoring cell-type-specific gene expression using ribosome profiling in vivo during cardiac hemodynamic stress. Circ Res.

[41] Bucca G, Pothi R, Hesketh A, Möller-Levet C, Hodgson DA, Laing EE, Stewart GR, Smith CP (2018). Translational control plays an important role in the adaptive heat-shock response of Streptomyces coelicolor. Nucleic Acids Res.

[42] Liu B, Qian SB (2014). Translational reprogramming in cellular stress response. Wiley Interdiscip Rev RNA.

[43] El-Naggar AM, Sorensen PH (2018). Translational control of aberrant stress responses as a hallmark of cancer. J Pathol.

[44] Schafer S, Adami E, Heinig M, Rodrigues KEC, Kreuchwig F, Silhavy J, van Heesch S, Simaite D, Rajewsky N, Cuppen E (2015). Translational regulation shapes the molecular landscape of complex disease phenotypes. Nat Commun.

[45] Gaillard H, García-Muse T, Aguilera A (2015). Replication stress and cancer. Nat Rev Cancer.

[46] Leppek K, Das R, Barna M (2018). Functional 5’ UTR mRNA structures in eukaryotic translation regulation and how to find them. Nat Rev Mol Cell Biol.

[47] De Benedetti A, Graff JR (2004). eIF-4E expression and its role in malignancies and metastases. Oncogene..

[48] Truitt ML, Conn CS, Shi Z, Pang X, Tokuyasu T, Coady AM, Seo Y, Barna M, Ruggero D (2015). Differential requirements for eIF4E dose in normal development and cancer. Cell..

[49] Hsieh AC, Liu Y, Edlind MP, Ingolia NT, Janes MR, Sher A, Shi EY, Stumpf CR, Christensen C, Bonham MJ (2012). The translational landscape of mTOR signalling steers cancer initiation and metastasis. Nature..

[50] Stacpoole PW (2017). Therapeutic targeting of the pyruvate dehydrogenase complex/pyruvate dehydrogenase kinase (PDC/PDK) axis in cancer. J Natl Cancer Inst.

[51] Goh WQ, Ow GS, Kuznetsov VA, Chong S, Lim YP (2015). DLAT subunit of the pyruvate dehydrogenase complex is upregulated in gastric cancer-implications in cancer therapy. Am J Transl Res.

[52] Schell JC, Rutter J (2013). The long and winding road to the mitochondrial pyruvate carrier. Cancer Metab.

[53] Patel MS, Nemeria NS, Furey W, Jordan F (2014). The pyruvate dehydrogenase complexes: structure-based function and regulation. J Biol Chem.

[54] Chen TY, Hsieh YT, Huang JM, Liu CJ, Chuang LT, Huang PC, Kuo TY, Chia HY, Chou CY, Chang CW (2019). Determination of pyruvate metabolic fates modulates head and neck tumorigenesis. Neoplasia..

[55] Rodríguez-Enríquez S, Marín-Hernández Á, Gallardo-Pérez JC, Pacheco-Velázquez SC, Belmont-Díaz JA, Robledo-Cadena DX, Vargas-Navarro JL, de la Peña NA C, Saavedra E, Moreno-Sánchez R (2019). Transcriptional regulation of energy metabolism in cancer cells. Cells..

[56] Li L, Liang Y, Kang L, Liu Y, Gao S, Chen S, Li Y, You W, Dong Q, Hong T (2018). Transcriptional regulation of the Warburg effect in cancer by SIX1. Cancer Cell.

[57] Tirpe AA, Gulei D, Ciortea SM, Crivii C, Berindan-Neagoe I (2019). Hypoxia: overview on hypoxia-mediated mechanisms with a focus on the role of HIF genes. Int J Mol Sci.

[58] Vizcaíno C, Mansilla S, Portugal J (2015). Sp1 transcription factor: a long-standing target in cancer chemotherapy. Pharmacol Ther.

[59] Safe S, Abbruzzese J, Abdelrahim M, Hedrick E (2018). Specificity protein transcription factors and cancer: opportunities for drug development. Cancer Prev Res (Phila).

[60] Peng F, Zhou Y, Wang J, Guo B, Wei Y, Deng H, Wu Z, Zhang C, Shi K, Li Y (2020). The transcription factor Sp1 modulates RNA polymerase III gene transcription by controlling BRF1 and GTF3C2 expression in human cells. J Biol Chem.

