# Supplementary figures and images for: PM2.5 promotes NSCLC carcinogenesis through translationally and transcriptionally activating DLAT-mediated glycolysis reprograming
Source: J Exp Clin Cancer Res. 2022 Jul 22;41:229. doi: 10.1186/s13046-022-02437-8 (PMC9308224; doi:10.1186/s13046-022-02437-8)

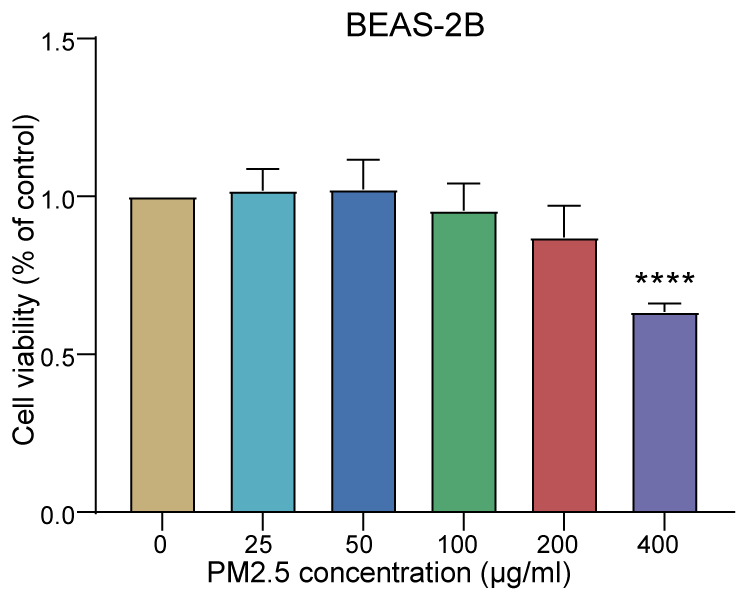

Supplement: Supplementary file 1 — Additional file 1: Fig. S1. Effects of PM2.5 exposure doses on cell cytotoxicity in BEAS-2B cells. ****P < 0.0001. [file 13046_2022_2437_MOESM1_ESM.tif]

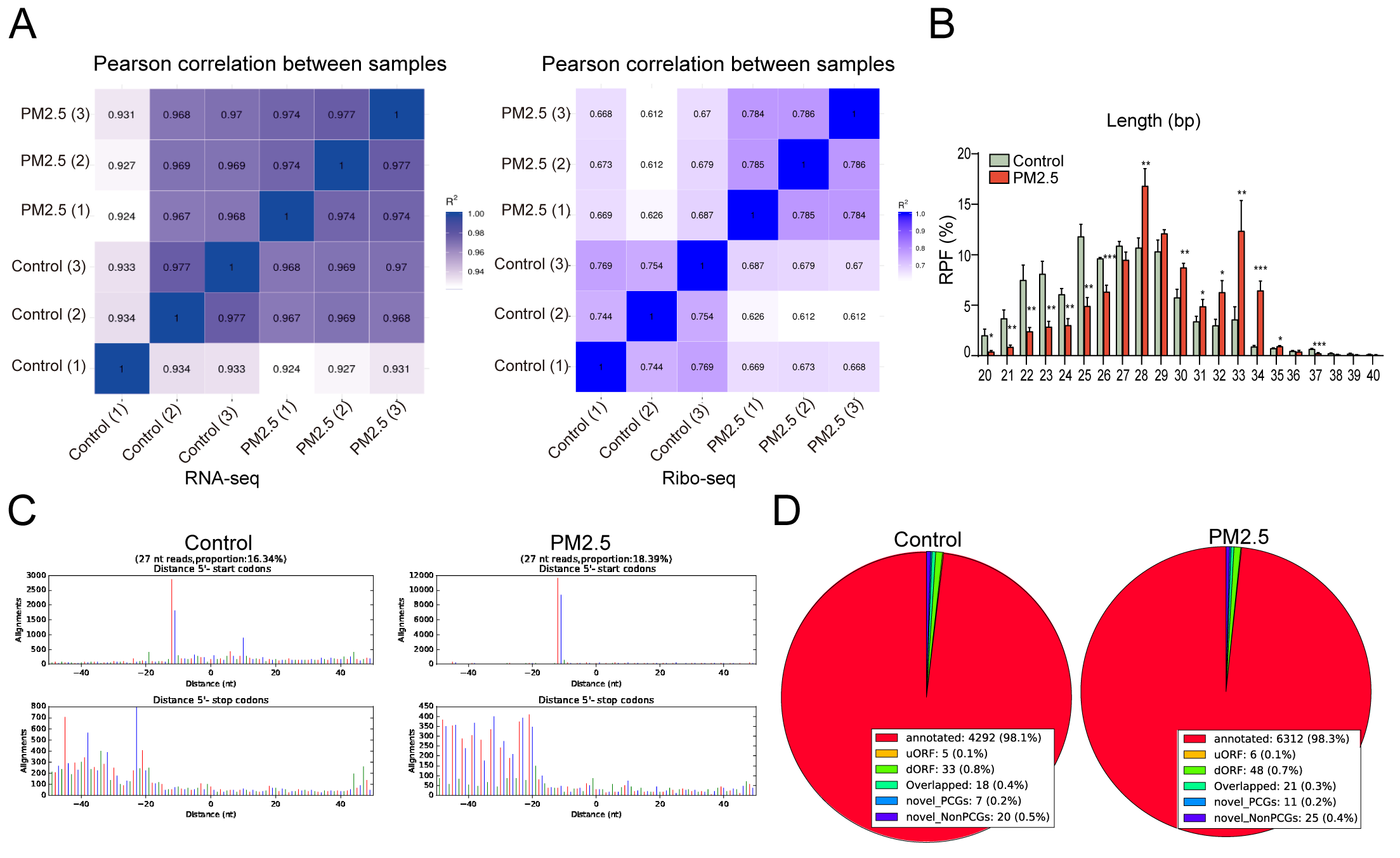

Supplement: Supplementary file 2 — Additional file 2: Fig. S2. RNA-seq and Ribo-seq on BEAS-2B cells following exposure to PM2.5. (A) RNA-seq and Ribo-seq reads were highly reproducible across biological replicates, respectively. (B) Size distribution and relative abundance of ribosome footprints reads between PM2.5-exposed and non-exposed BEAS-2B cells. (C) Representative bar plots showing the peptidyl-site (P-site) position derived from Ribo-seq reads across the first 50 nt and last 50 nt of open reading frames (ORFs). (D) The proportion of different categories of ORFs detected in our study. Annotated, annotated open reading frames (ORFs) for proteins; uORFs, upstream ORF; Dorf, downstream ORF; overlapped, uORF/dORF overlapped with main ORF; novel_PCGs, novel ORFs from protein-coding genes; novel_NonPCGs, novel ORFs from non-protein-coding genes. [file 13046_2022_2437_MOESM2_ESM.tif]

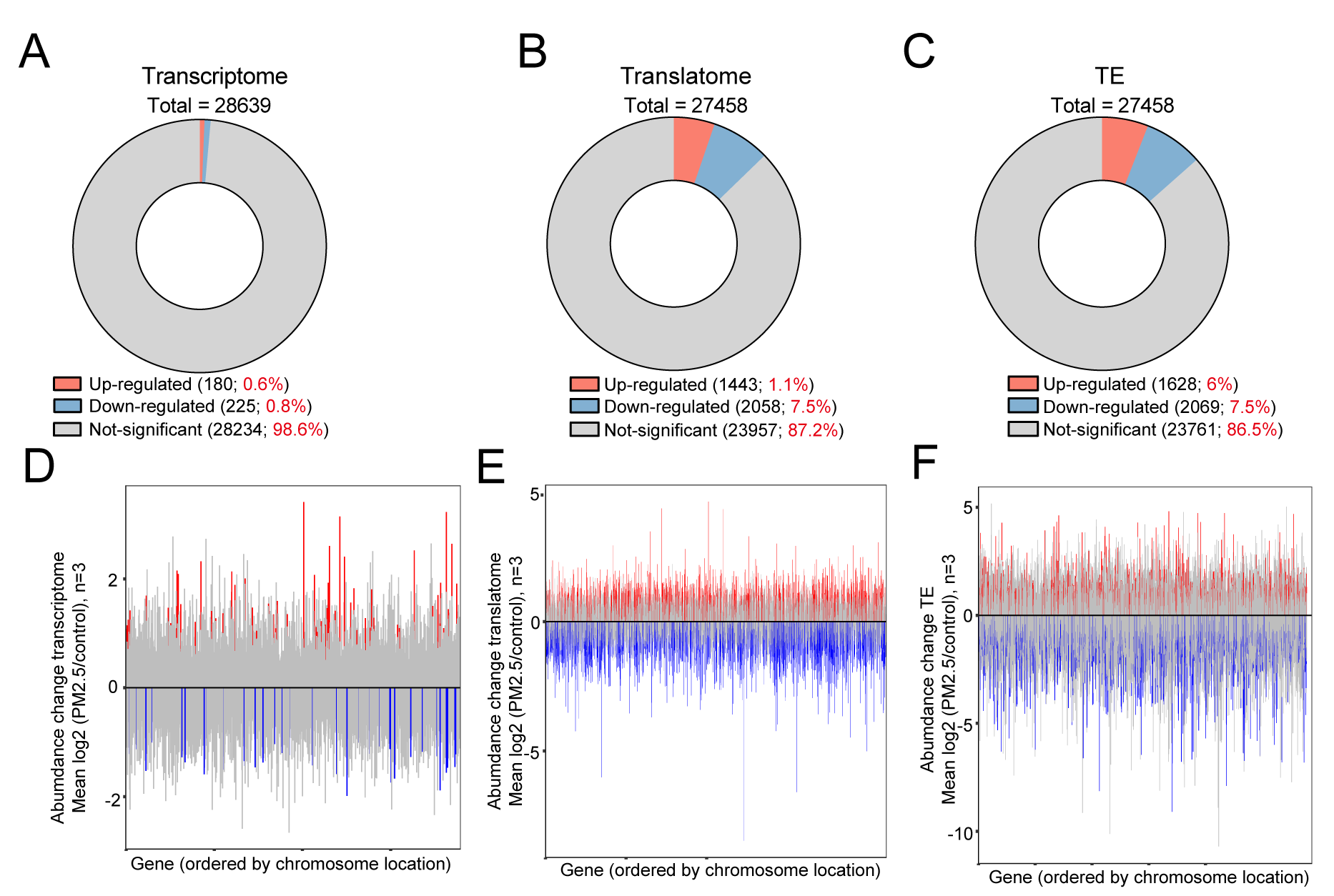

Supplement: Supplementary file 3 — Additional file 3: Fig. S3. Number of DE-genes and the abundance of DE-genes in transcriptome, translatome, and TE following PM2.5 exposure. (A, B, C) Numbers of significantly up- or down-regulated transcripts in transcriptome, translatome, and TE. (D, E, F) Relative log2 fold-change (FC) in transcript abundance between PM2.5-exposed cells versus control cells in transcriptome, translatome, and TE. Transcripts significantly up-regulated, down-regulated, or non-significant change are colored in red, blue, and light grey, respectively. [file 13046_2022_2437_MOESM3_ESM.tif]

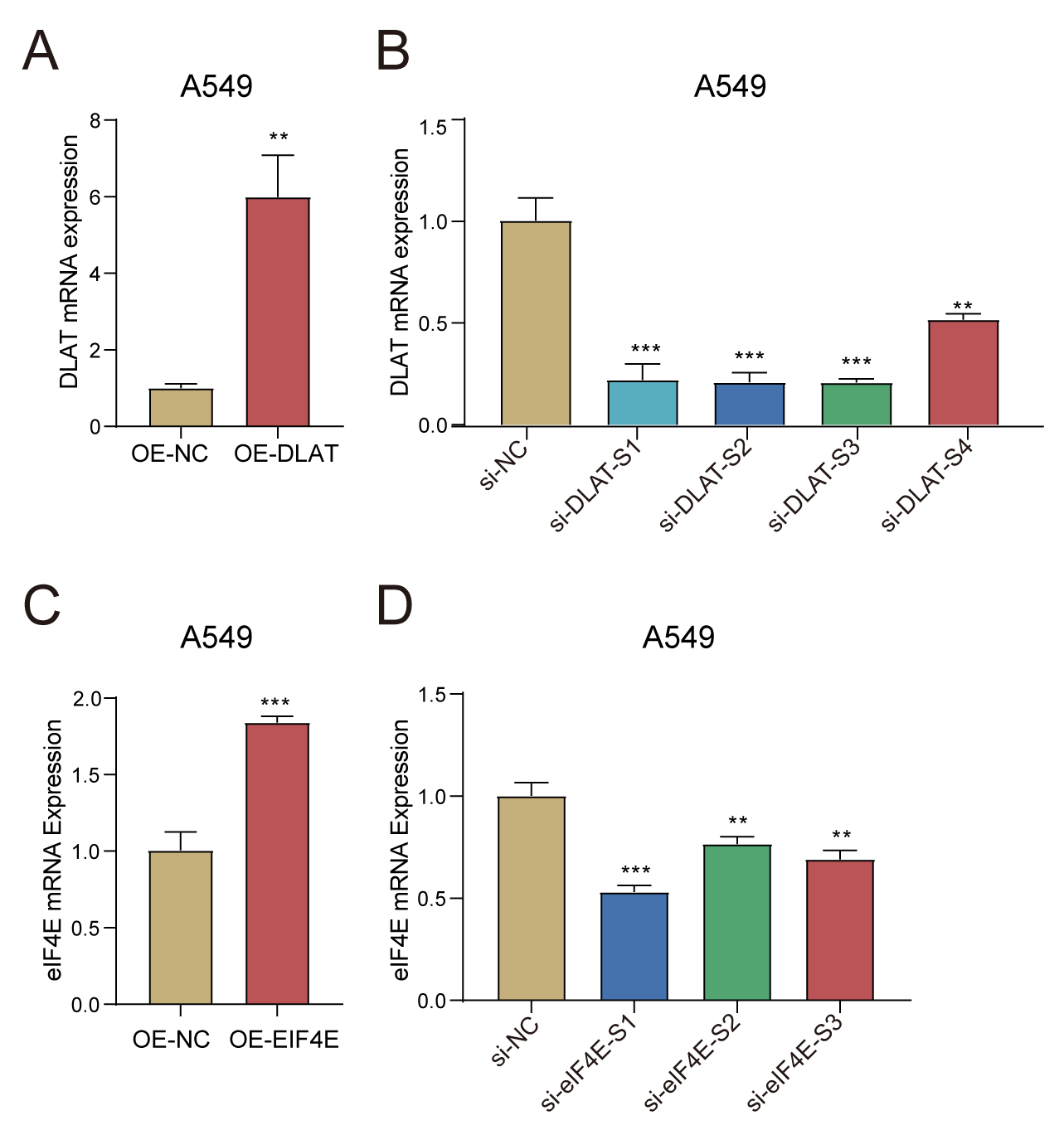

Supplement: Supplementary file 4 — Additional file 4: Fig. S4. Transfection efficiencies of DLAT and eIF4E overexpression vector and corresponding si-RNAs. (A) Transfection efficiency of DLAT-overexpression vector in A549 cells. (B) knockdown efficiency of si-DLAT in A549 cells. (C) Transfection efficiency of eIF4E-overexpression vector in A549 cells. (D) Inhibition efficiency si-eIF4E in A549 cells. *P < 0.05; **P < 0.01; ***P < 0.001. [file 13046_2022_2437_MOESM4_ESM.tif]

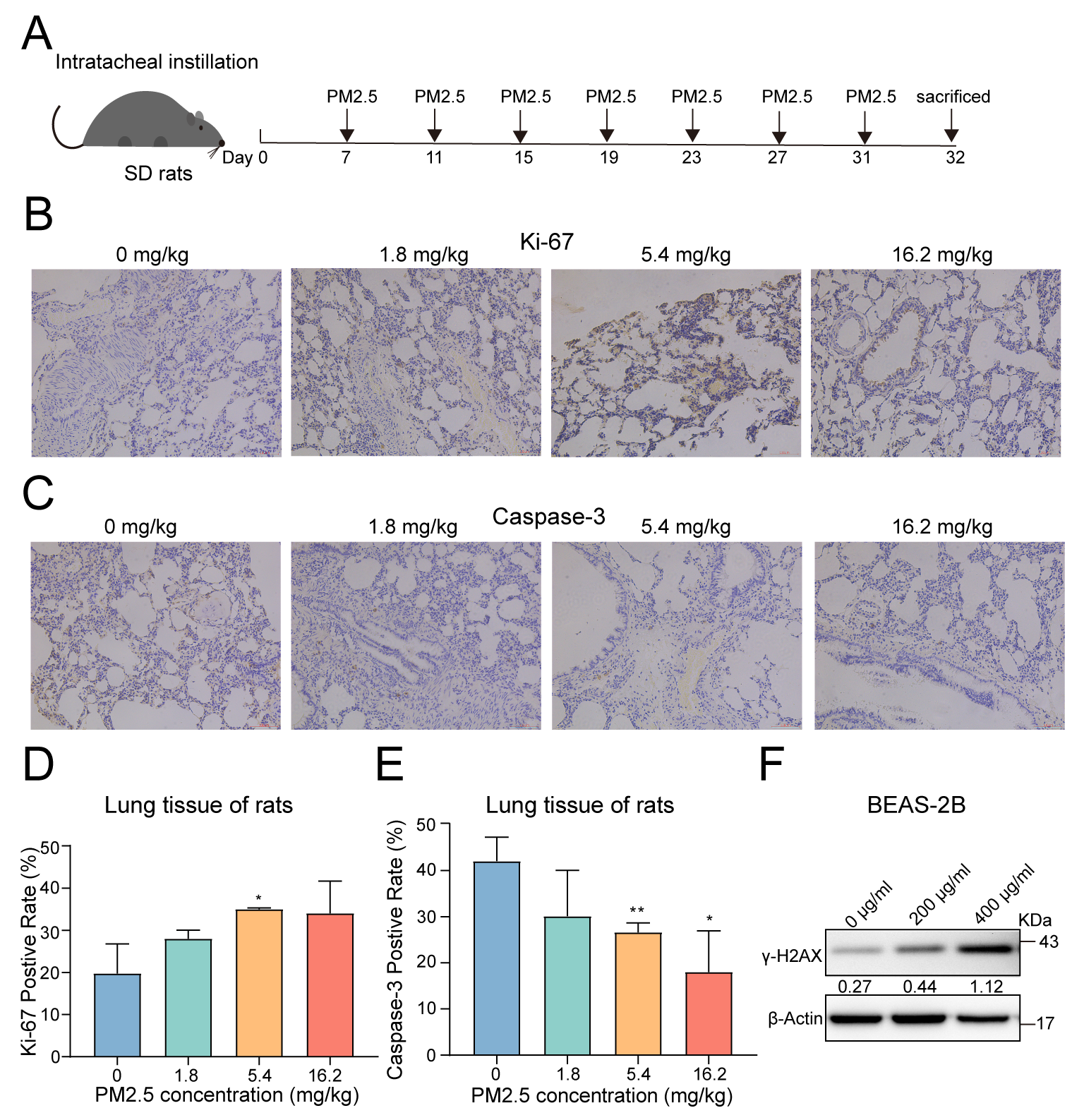

Supplement: Supplementary file 5 — Additional file 5: Fig. S5. PM2.5 promotes the expression levels of cancer-associated markers in lung tissue of rats. (A) Schematic view of animal experiments. (B) Representative IHC staining images of the expression of Ki-67 protein, a marker of cell proliferation, in lung tissues of rats. (C) Representative pictures of IHC staining of caspase-3, a marker of apoptosis, in lung tissues of rats. (D) PM2.5 exposure increased Ki-67 expression levels in lung tissues of rats in a dose-response manner. (E) PM2.5 exposure decreased the expression levels of caspase-3 in lung tissues of rats. (F) PM2.5 enhanced the expression levels of γ-H2Ax, a marker of DNA damage and carcinogenesis, in lung tissues of rats in a dose-dependent manner. *P < 0.05; **P < 0.01. [file 13046_2022_2437_MOESM5_ESM.tif]

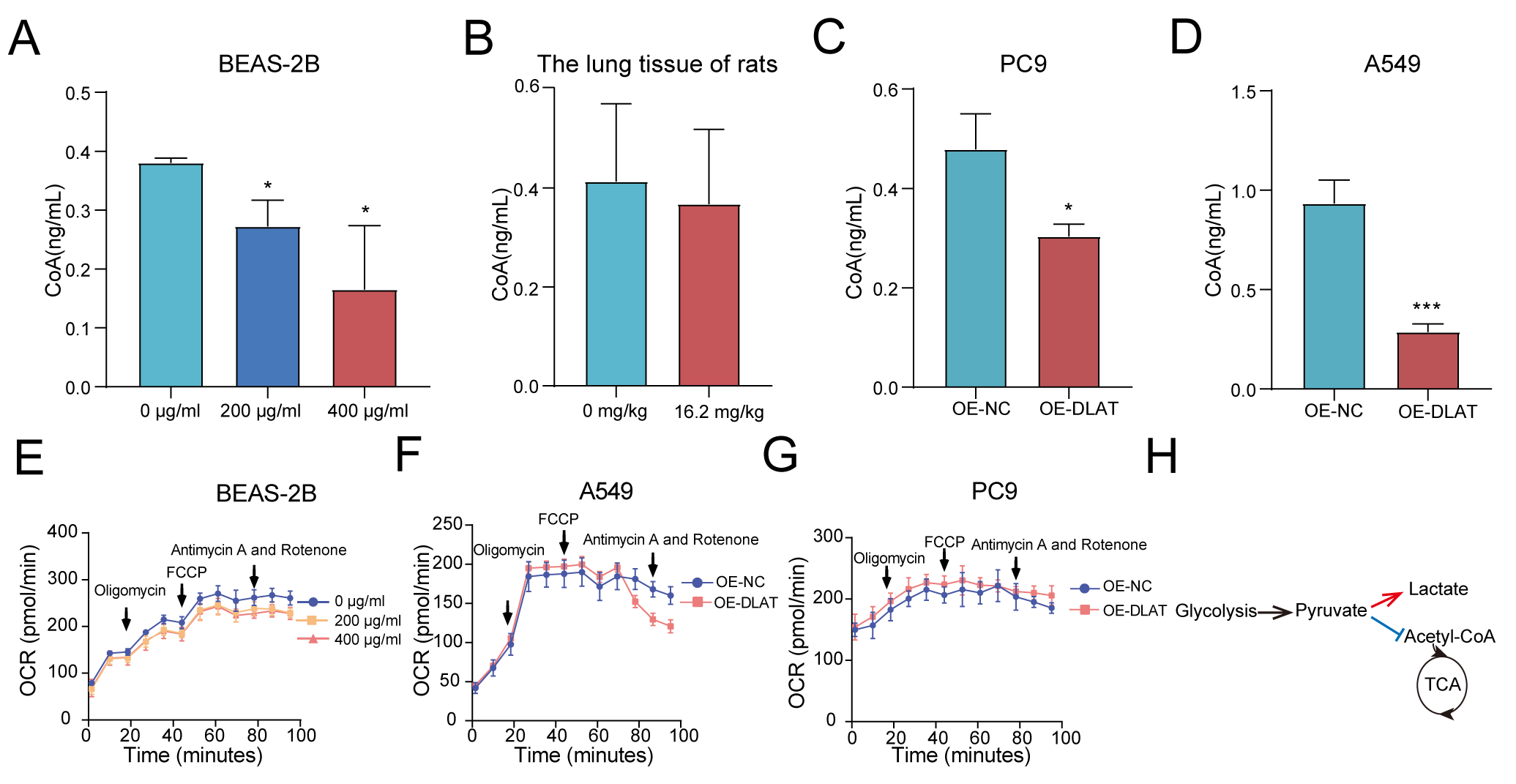

Supplement: Supplementary file 6 — Additional file 6: Fig. S6. PM2.5 and DLAT decrease acetyl-CoA production but do not alter TCA cycle metabolism level. (A) PM2.5 exposure inhibited acetyl-CoA generation in BEAS-2B cells. (B) There was a trend of decrease in acetyl-CoA levels in PM2.5-exposed lung tissues of rats. (C, D) Overexpression of DLAT suppressed acetyl-CoA production from NSCLC cells. (E) PM2.5 exposure did not significantly alter OCR levels in BEAS-2B cells. (F, G) Up-regulation of DLAT did not influence the OCR levels in NSCLC cells. (H) Schematic function of pyruvate in glycolysis metabolism and TCA cycle. *P < 0.05; **P < 0.01; ***P < 0.001. [file 13046_2022_2437_MOESM6_ESM.tif]

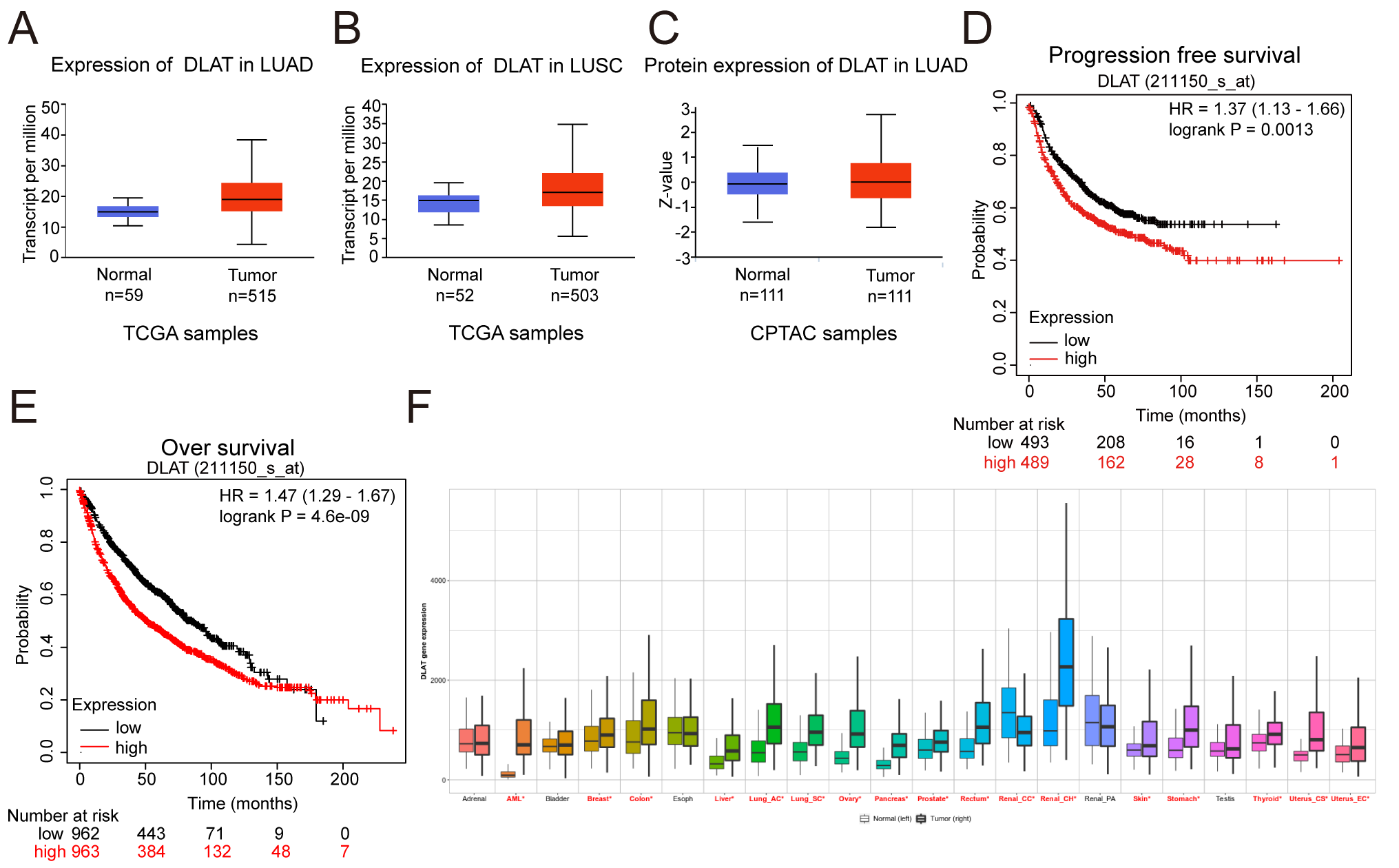

Supplement: Supplementary file 7 — Additional file 7: Fig. S7. Clinical significance of DLAT expression in NSCLC in TCGA datasets. (A) DLAT was up-regulated in tumor tissues of LUAD (lung adenocarcinoma). (B) The expression level of DLAT in LUSC (lung squamous cell carcinoma) tissue was higher than that in normal tissues. (C) The expression of DLAT protein in LUAD was higher than that in normal tissues. (D) Higher expression level of DLAT in tumor tissues was associated with worse overall survival of NSCLC. (E) Higher expression level of DLAT in tumor tissues was associated with worse progression free survival of NSCLC patients. (F) DLAT was up-regulated in other types of primary tumors. [file 13046_2022_2437_MOESM7_ESM.tif]

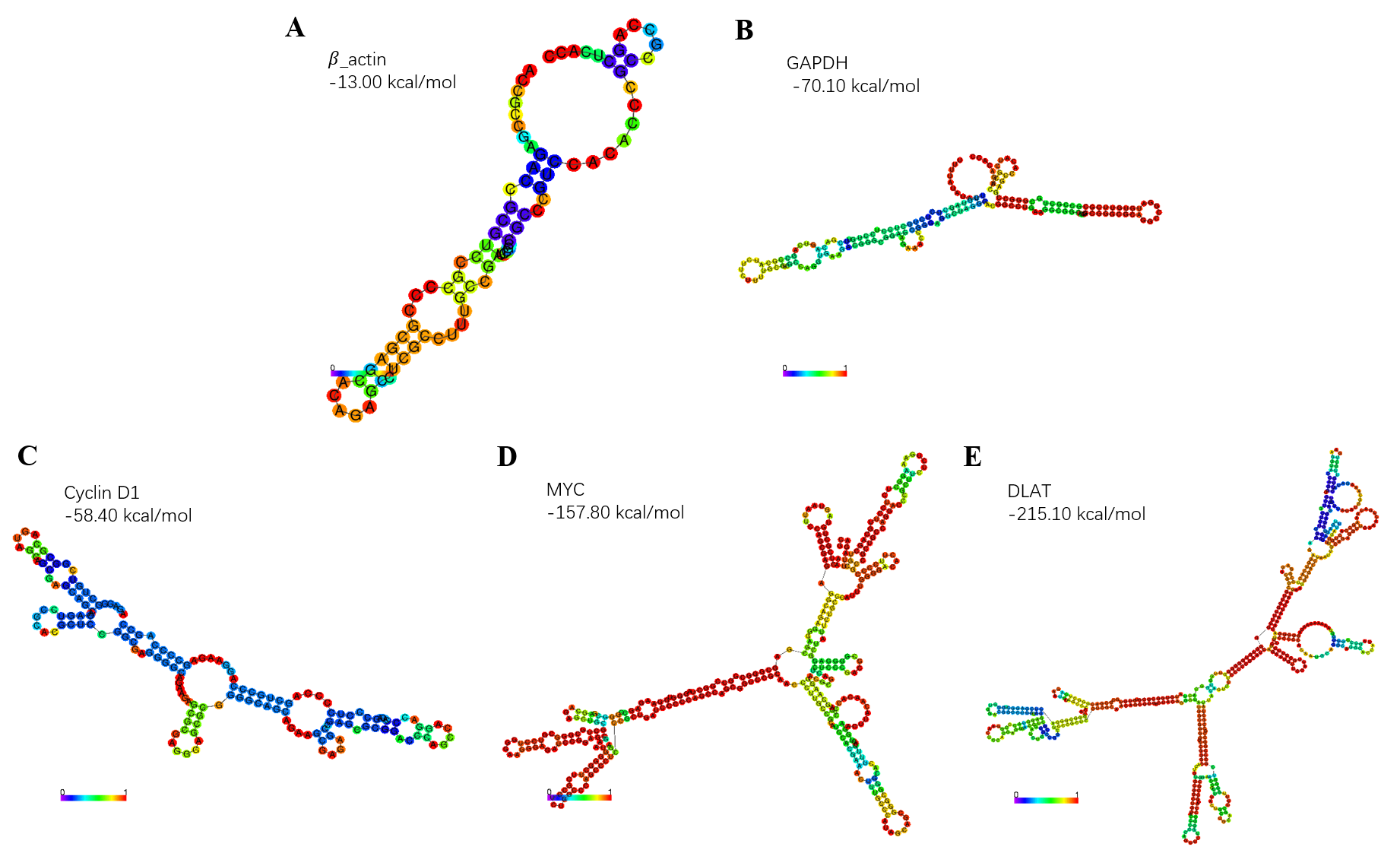

Supplement: Supplementary file 8 — Additional file 8: Fig. S8. Secondary structures in 5′-UTR of representative genes. (A) β-actin. (B) GAPDH. (C) Cyclin D1. (D) MYC. (E) DLAT. The structures were predicted using the RNAFold software. The minimum free energy was indicated. [file 13046_2022_2437_MOESM8_ESM.tif]
