# Supplementary material for: PM2.5 promotes NSCLC carcinogenesis through translationally and transcriptionally activating DLAT-mediated glycolysis reprograming
Source: J Exp Clin Cancer Res. 2022 Jul 22;41:229. doi: 10.1186/s13046-022-02437-8 (PMC9308224; doi:10.1186/s13046-022-02437-8)
Supplement: Supplementary file 9 — Additional file 9: Table S1. QC data of Ribo-seq and RNA-seq. [file 13046_2022_2437_MOESM9_ESM.docx]

| **Table S1.** **QC data of Ribo-seq and RNA-seq** | | | | | | |
| --- | --- | --- | --- | --- | --- | --- |
|  | **PM2.5-exposed Sample 1** | **PM2.5-exposed Sample 2** | **PM2.5-exposed Sample 3** | **Control**  **Sample 1** | **Control**  **Sample 2** | **Control**  **Sample 3** |
| **Ribo-seq** |  |  |  |  |  |  |
| Raw Reads Number | 37893546 | 48613604 | 33865552 | 48060166 | 40271844 | 44687624 |
| Clean Reads Number | 37293581 | 47931176 | 33379427 | 47475015 | 39670425 | 44115556 |
| rRNA Mapping Reads Number | 28296016 | 39763374 | 27748359 | 37894793 | 30053878 | 34389563 |
| Total mapped reads | 2462102 | 2299057 | 1770613 | 2705510 | 2526277 | 2495409 |
| Uniquely mapped reads | 1237230 | 991248 | 853601 | 1600703 | 1576328 | 1475731 |
| **RNA-seq** |  |  |  |  |  |  |
| Raw Reads Number | 45628152 | 48724304 | 48089716 | 45369770 | 47277960 | 47109134 |
| Clean Reads Number | 43846264 | 46743958 | 46245520 | 43449218 | 44899058 | 45093470 |
| Total mapped reads | 40349173 | 43078965 | 42576880 | 39818351 | 40849551 | 41018180 |
| Uniquely mapped reads | 39388565 | 42045296 | 41552214 | 38840099 | 39849252 | 40035966 |
