# Supplementary material for: PM2.5 promotes NSCLC carcinogenesis through translationally and transcriptionally activating DLAT-mediated glycolysis reprograming
Source: J Exp Clin Cancer Res. 2022 Jul 22;41:229. doi: 10.1186/s13046-022-02437-8 (PMC9308224; doi:10.1186/s13046-022-02437-8)
Supplement: Supplementary file 10 — Additional file 10: Table S2. Differentially expressed mRNAs regulated by PM2.5 in BEAS-2B cells. [file 13046_2022_2437_MOESM10_ESM.docx]

| **Table S2. Differentially expressed mRNAs regulated by PM2.5 in BEAS-2B cells** | | | | |
| --- | --- | --- | --- | --- |
| **Gene_id** | **log2FoldChange** | **P value** | **Padj** | **Gene name** |
| ENSG00000197632 | 3.411151874 | 5.00E-48 | 7.37E-44 | SERPINB2 |
| ENSG00000151012 | 2.522507696 | 7.88E-37 | 5.81E-33 | SLC7A11 |
| ENSG00000138061 | 1.760179555 | 6.23E-36 | 3.07E-32 | CYP1B1 |
| ENSG00000163659 | 1.50065463 | 5.41E-31 | 2.00E-27 | TIPARP |
| ENSG00000161011 | 1.095952466 | 7.09E-31 | 2.02E-27 | SQSTM1 |
| ENSG00000113739 | 1.929074393 | 8.20E-31 | 2.02E-27 | STC2 |
| ENSG00000198431 | 1.284631767 | 3.47E-30 | 7.32E-27 | TXNRD1 |
| ENSG00000108448 | 2.090655829 | 1.08E-29 | 1.99E-26 | TRIM16L |
| ENSG00000140961 | 2.408321777 | 4.53E-26 | 7.42E-23 | OSGIN1 |
| ENSG00000124882 | 1.462962988 | 3.72E-21 | 5.49E-18 | EREG |
| ENSG00000162892 | 2.644077594 | 3.34E-20 | 4.49E-17 | IL24 |
| ENSG00000156804 | 1.157750458 | 6.88E-20 | 8.46E-17 | FBXO32 |
| ENSG00000100292 | 1.368749957 | 6.95E-19 | 7.89E-16 | HMOX1 |
| ENSG00000137491 | 2.603271402 | 1.17E-17 | 1.24E-14 | SLCO2B1 |
| ENSG00000144810 | -1.055417397 | 5.32E-17 | 5.23E-14 | COL8A1 |
| ENSG00000135114 | -1.037189396 | 6.19E-17 | 5.71E-14 | OASL |
| ENSG00000105974 | -0.941826072 | 8.35E-17 | 7.25E-14 | CAV1 |
| ENSG00000166923 | 1.274257119 | 1.76E-15 | 1.44E-12 | GREM1 |
| ENSG00000248323 | 1.826622628 | 1.86E-15 | 1.45E-12 | LUCAT1 |
| ENSG00000171658 | 3.22599965 | 5.35E-15 | 3.94E-12 | NMRAL2P |
| ENSG00000180730 | 1.211037625 | 1.59E-14 | 1.12E-11 | SHISA2 |
| ENSG00000128510 | -1.038154039 | 2.30E-14 | 1.54E-11 | CPA4 |
| ENSG00000023909 | 1.058198335 | 3.56E-14 | 2.28E-11 | GCLM |
| ENSG00000134321 | -1.005818442 | 8.17E-14 | 5.02E-11 | RSAD2 |
| ENSG00000136999 | 0.979077 | 2.08E-13 | 1.23E-10 | NOV |
| ENSG00000152661 | -1.118393754 | 2.38E-13 | 1.35E-10 | GJA1 |
| ENSG00000105855 | -1.084134097 | 2.69E-13 | 1.47E-10 | ITGB8 |
| ENSG00000280208 | 1.499554855 | 1.15E-12 | 6.05E-10 | GGT4P |
| ENSG00000184564 | -1.986961461 | 1.22E-12 | 6.18E-10 | SLITRK6 |
| ENSG00000164442 | -0.968767752 | 2.43E-12 | 1.19E-09 | CITED2 |
| ENSG00000153094 | -0.91733195 | 5.98E-12 | 2.84E-09 | BCL2L11 |
| ENSG00000138678 | 1.571002225 | 1.05E-11 | 4.84E-09 | GPAT3 |
| ENSG00000134363 | -1.198021929 | 1.37E-11 | 6.13E-09 | FST |
| ENSG00000063438 | 1.247831264 | 1.95E-11 | 8.46E-09 | AHRR |
| ENSG00000124813 | 1.04673592 | 2.11E-11 | 8.90E-09 | RUNX2 |
| ENSG00000066468 | -1.668398406 | 2.30E-11 | 9.42E-09 | FGFR2 |
| ENSG00000137809 | 2.071503903 | 3.17E-11 | 1.27E-08 | ITGA11 |
| ENSG00000019549 | 1.545505437 | 7.02E-11 | 2.73E-08 | SNAI2 |
| ENSG00000163435 | -1.402745764 | 1.21E-10 | 4.58E-08 | ELF3 |
| ENSG00000125378 | -0.991554972 | 2.28E-10 | 8.43E-08 | BMP4 |
| ENSG00000131094 | -1.03498563 | 2.57E-10 | 9.24E-08 | C1QL1 |
| ENSG00000163430 | -0.645187163 | 2.77E-10 | 9.73E-08 | FSTL1 |
| ENSG00000121895 | 1.887910561 | 3.82E-10 | 1.31E-07 | TMEM156 |
| ENSG00000073150 | 1.346567486 | 4.69E-10 | 1.57E-07 | PANX2 |
| ENSG00000134955 | 1.095259492 | 4.93E-10 | 1.62E-07 | SLC37A2 |
| ENSG00000163931 | 0.665826374 | 1.14E-09 | 3.64E-07 | TKT |
| ENSG00000107738 | -0.986954903 | 1.17E-09 | 3.66E-07 | VSIR |
| ENSG00000232973 | 2.135783733 | 1.65E-09 | 5.06E-07 | CYP1B1-AS1 |
| ENSG00000087074 | 0.59261655 | 1.83E-09 | 5.53E-07 | PPP1R15A |
| ENSG00000067798 | 1.414790349 | 2.76E-09 | 8.14E-07 | NAV3 |
| ENSG00000127561 | -1.127758741 | 3.39E-09 | 9.81E-07 | SYNGR3 |
| ENSG00000171246 | 3.146700695 | 3.53E-09 | 1.00E-06 | NPTX1 |
| ENSG00000140465 | 1.927311741 | 5.32E-09 | 1.48E-06 | CYP1A1 |
| ENSG00000242265 | -1.003345275 | 5.52E-09 | 1.48E-06 | PEG10 |
| ENSG00000196611 | 1.807777028 | 5.59E-09 | 1.48E-06 | MMP1 |
| ENSG00000095203 | -1.077032584 | 5.60E-09 | 1.48E-06 | EPB41L4B |
| ENSG00000157168 | 1.409382673 | 6.00E-09 | 1.55E-06 | NRG1 |
| ENSG00000132429 | 0.978337131 | 6.69E-09 | 1.70E-06 | POPDC3 |
| ENSG00000166250 | 0.604095911 | 7.37E-09 | 1.84E-06 | CLMP |
| ENSG00000221926 | 1.185629957 | 7.96E-09 | 1.91E-06 | TRIM16 |
| ENSG00000168497 | -1.465478471 | 7.97E-09 | 1.91E-06 | CAVIN2 |
| ENSG00000106484 | -0.893157795 | 8.03E-09 | 1.91E-06 | MEST |
| ENSG00000197063 | 0.892198533 | 8.45E-09 | 1.98E-06 | MAFG |
| ENSG00000139174 | -1.432796241 | 8.74E-09 | 2.02E-06 | PRICKLE1 |
| ENSG00000057294 | -0.970319332 | 1.03E-08 | 2.34E-06 | PKP2 |
| ENSG00000121858 | -1.88229162 | 1.19E-08 | 2.63E-06 | TNFSF10 |
| ENSG00000118898 | -1.526703139 | 1.19E-08 | 2.63E-06 | PPL |
| ENSG00000102312 | 0.880897584 | 1.36E-08 | 2.95E-06 | PORCN |
| ENSG00000087245 | 0.718048503 | 1.39E-08 | 2.98E-06 | MMP2 |
| ENSG00000265972 | -0.892296188 | 1.60E-08 | 3.35E-06 | TXNIP |
| ENSG00000145685 | 0.788016704 | 1.61E-08 | 3.35E-06 | LHFPL2 |
| ENSG00000023330 | 0.614162906 | 1.96E-08 | 4.01E-06 | ALAS1 |
| ENSG00000128342 | 0.98016415 | 2.09E-08 | 4.22E-06 | LIF |
| ENSG00000162433 | -0.756194559 | 2.29E-08 | 4.57E-06 | AK4 |
| ENSG00000116285 | -0.622288254 | 3.18E-08 | 6.25E-06 | ERRFI1 |
| ENSG00000279124 | 2.152060567 | 3.40E-08 | 6.61E-06 | AL356585.2 |
| ENSG00000119922 | -0.721391247 | 3.46E-08 | 6.64E-06 | IFIT2 |
| ENSG00000133106 | -0.811300937 | 3.85E-08 | 7.28E-06 | EPSTI1 |
| ENSG00000048052 | 1.090609308 | 7.32E-08 | 1.35E-05 | HDAC9 |
| ENSG00000184254 | 0.721252951 | 7.34E-08 | 1.35E-05 | ALDH1A3 |
| ENSG00000138944 | 1.472163016 | 7.98E-08 | 1.45E-05 | SHISAL1 |
| ENSG00000156475 | -1.173494155 | 8.49E-08 | 1.53E-05 | PPP2R2B |
| ENSG00000171951 | -1.452494435 | 8.73E-08 | 1.55E-05 | SCG2 |
| ENSG00000197971 | 0.983587357 | 9.83E-08 | 1.73E-05 | MBP |
| ENSG00000134326 | -0.961280234 | 1.32E-07 | 2.30E-05 | CMPK2 |
| ENSG00000247095 | -1.215082911 | 1.99E-07 | 3.42E-05 | MIR210HG |
| ENSG00000175567 | -0.705351082 | 2.21E-07 | 3.75E-05 | UCP2 |
| ENSG00000167552 | -0.959572381 | 2.68E-07 | 4.49E-05 | TUBA1A |
| ENSG00000023839 | 1.278637147 | 2.94E-07 | 4.88E-05 | ABCC2 |
| ENSG00000108179 | 0.75346954 | 3.02E-07 | 4.94E-05 | PPIF |
| ENSG00000115594 | 1.1031891 | 3.15E-07 | 5.11E-05 | IL1R1 |
| ENSG00000092295 | -1.138838011 | 3.39E-07 | 5.44E-05 | TGM1 |
| ENSG00000114812 | 2.006247493 | 3.43E-07 | 5.44E-05 | VIPR1 |
| ENSG00000135678 | 1.140485679 | 3.56E-07 | 5.59E-05 | CPM |
| ENSG00000180447 | -0.686806655 | 4.70E-07 | 7.31E-05 | GAS1 |
| ENSG00000102466 | -1.211963785 | 5.32E-07 | 8.18E-05 | FGF14 |
| ENSG00000271303 | 1.420278608 | 6.63E-07 | 0.000101 | SRXN1 |
| ENSG00000154556 | -1.558311876 | 7.16E-07 | 0.000108 | SORBS2 |
| ENSG00000107105 | -1.09659919 | 8.46E-07 | 0.000126 | ELAVL2 |
| ENSG00000115380 | -0.670110366 | 8.50E-07 | 0.000126 | EFEMP1 |
| ENSG00000144579 | -0.552663495 | 9.81E-07 | 0.000142 | CTDSP1 |
| ENSG00000103257 | 0.896873981 | 9.83E-07 | 0.000142 | SLC7A5 |
| ENSG00000111057 | -1.077474118 | 1.02E-06 | 0.000147 | KRT18 |
| ENSG00000109107 | -0.688465232 | 1.44E-06 | 0.000204 | ALDOC |
| ENSG00000215267 | 4.168529402 | 1.49E-06 | 0.000209 | AKR1C7P |
| ENSG00000108846 | 1.090220412 | 1.50E-06 | 0.000209 | ABCC3 |
| ENSG00000104687 | 0.610160758 | 1.70E-06 | 0.000235 | GSR |
| ENSG00000179630 | 0.797768696 | 1.74E-06 | 0.000238 | LACC1 |
| ENSG00000118523 | -0.914391715 | 1.87E-06 | 0.000254 | CTGF |
| ENSG00000168453 | -1.107321242 | 2.15E-06 | 0.000288 | HR |
| ENSG00000182809 | -0.912133207 | 2.24E-06 | 0.000296 | CRIP2 |
| ENSG00000165507 | -1.460459891 | 2.24E-06 | 0.000296 | DEPP1 |
| ENSG00000171388 | -0.886994153 | 2.67E-06 | 0.000346 | APLN |
| ENSG00000188613 | -1.121391768 | 2.67E-06 | 0.000346 | NANOS1 |
| ENSG00000100311 | -0.847905646 | 2.73E-06 | 0.00035 | PDGFB |
| ENSG00000138646 | -0.887634099 | 2.75E-06 | 0.00035 | HERC5 |
| ENSG00000102981 | -1.424776446 | 2.89E-06 | 0.000364 | PARD6A |
| ENSG00000012171 | -0.760947707 | 2.98E-06 | 0.000373 | SEMA3B |
| ENSG00000142910 | -0.55988316 | 3.14E-06 | 0.000389 | TINAGL1 |
| ENSG00000133805 | 0.902482546 | 3.25E-06 | 0.000398 | AMPD3 |
| ENSG00000250033 | 2.779083781 | 3.26E-06 | 0.000398 | SLC7A11-AS1 |
| ENSG00000162654 | -0.894890259 | 3.33E-06 | 0.000403 | GBP4 |
| ENSG00000198835 | -1.220249845 | 3.37E-06 | 0.000404 | GJC2 |
| ENSG00000134070 | 0.66637606 | 3.39E-06 | 0.000404 | IRAK2 |
| ENSG00000113552 | 0.589575994 | 4.05E-06 | 0.000475 | GNPDA1 |
| ENSG00000107159 | -1.501962247 | 4.05E-06 | 0.000475 | CA9 |
| ENSG00000173801 | -0.690977386 | 4.23E-06 | 0.000492 | JUP |
| ENSG00000099864 | -0.670428541 | 4.55E-06 | 0.000525 | PALM |
| ENSG00000119938 | -0.870485401 | 5.19E-06 | 0.000594 | PPP1R3C |
| ENSG00000230615 | -0.964600735 | 5.36E-06 | 0.000609 | AL139220.2 |
| ENSG00000134668 | 0.811261652 | 5.40E-06 | 0.000609 | SPOCD1 |
| ENSG00000164509 | 1.350395869 | 5.48E-06 | 0.000611 | IL31RA |
| ENSG00000181019 | 1.025237415 | 5.50E-06 | 0.000611 | NQO1 |
| ENSG00000170017 | -0.421413086 | 5.89E-06 | 0.000649 | ALCAM |
| ENSG00000162366 | -1.372007383 | 6.29E-06 | 0.000683 | PDZK1IP1 |
| ENSG00000167693 | -0.532809896 | 6.35E-06 | 0.000683 | NXN |
| ENSG00000187134 | 1.691615483 | 6.35E-06 | 0.000683 | AKR1C1 |
| ENSG00000169429 | 1.15844583 | 6.39E-06 | 0.000683 | CXCL8 |
| ENSG00000160211 | 0.459200025 | 7.06E-06 | 0.00075 | G6PD |
| ENSG00000254087 | 0.655288067 | 7.43E-06 | 0.000783 | LYN |
| ENSG00000166508 | -0.426543271 | 8.19E-06 | 0.000857 | MCM7 |
| ENSG00000137699 | -1.345073136 | 8.31E-06 | 0.000863 | TRIM29 |
| ENSG00000075213 | -0.636539975 | 8.47E-06 | 0.000874 | SEMA3A |
| ENSG00000118777 | 0.987889416 | 9.61E-06 | 0.000984 | ABCG2 |
| ENSG00000090238 | -1.062680079 | 1.00E-05 | 0.001019 | YPEL3 |
| ENSG00000143067 | 0.738232188 | 1.02E-05 | 0.00103 | ZNF697 |
| ENSG00000205609 | 2.322637225 | 1.03E-05 | 0.00103 | EIF3CL |
| ENSG00000131389 | 1.469139874 | 1.11E-05 | 0.001102 | SLC6A6 |
| ENSG00000084636 | 0.730256994 | 1.14E-05 | 0.001129 | COL16A1 |
| ENSG00000153721 | -0.573364146 | 1.15E-05 | 0.001129 | CNKSR3 |
| ENSG00000170385 | 0.508221217 | 1.16E-05 | 0.001129 | SLC30A1 |
| ENSG00000131941 | -0.49937113 | 1.36E-05 | 0.00132 | RHPN2 |
| ENSG00000150347 | -0.644607536 | 1.38E-05 | 0.001328 | ARID5B |
| ENSG00000223749 | -1.268823801 | 1.39E-05 | 0.001333 | MIR503HG |
| ENSG00000107201 | -0.528999253 | 1.44E-05 | 0.001372 | DDX58 |
| ENSG00000139211 | 0.774923744 | 1.46E-05 | 0.001377 | AMIGO2 |
| ENSG00000153404 | 1.144235931 | 1.49E-05 | 0.001398 | PLEKHG4B |
| ENSG00000157227 | 0.514584731 | 1.60E-05 | 0.001493 | MMP14 |
| ENSG00000221955 | 1.095224713 | 1.70E-05 | 0.001577 | SLC12A8 |
| ENSG00000177595 | -0.550460883 | 1.78E-05 | 0.001641 | PIDD1 |
| ENSG00000108828 | 0.433667873 | 1.88E-05 | 0.001722 | VAT1 |
| ENSG00000138411 | 1.441244872 | 1.94E-05 | 0.00176 | HECW2 |
| ENSG00000145107 | 1.377358674 | 1.94E-05 | 0.00176 | TM4SF19 |
| ENSG00000271503 | -0.740979457 | 1.98E-05 | 0.001783 | CCL5 |
| ENSG00000144677 | -0.499838785 | 1.99E-05 | 0.001783 | CTDSPL |
| ENSG00000197355 | 0.694104889 | 2.01E-05 | 0.001787 | UAP1L1 |
| ENSG00000176532 | -1.589975726 | 2.06E-05 | 0.001824 | PRR15 |
| ENSG00000156510 | 1.115012775 | 2.11E-05 | 0.001852 | HKDC1 |
| ENSG00000166165 | -0.970002779 | 2.12E-05 | 0.001853 | CKB |
| ENSG00000183691 | -1.236768522 | 2.47E-05 | 0.00214 | NOG |
| ENSG00000155324 | -0.62966205 | 2.83E-05 | 0.002439 | GRAMD2B |
| ENSG00000213347 | -0.851191939 | 3.11E-05 | 0.002672 | MXD3 |
| ENSG00000114251 | 0.556683669 | 3.46E-05 | 0.002952 | WNT5A |
| ENSG00000117152 | -0.916825222 | 3.52E-05 | 0.002982 | RGS4 |
| ENSG00000178531 | -1.248356955 | 3.57E-05 | 0.003007 | CTXN1 |
| ENSG00000148082 | 1.563783027 | 3.65E-05 | 0.003064 | SHC3 |
| ENSG00000147676 | -1.740648547 | 3.72E-05 | 0.003101 | MAL2 |
| ENSG00000092820 | -0.436516889 | 3.81E-05 | 0.003157 | EZR |
| ENSG00000109511 | 1.700594177 | 3.87E-05 | 0.003191 | ANXA10 |
| ENSG00000167994 | 0.716696762 | 4.03E-05 | 0.003301 | RAB3IL1 |
| ENSG00000112541 | -1.255486248 | 4.14E-05 | 0.003376 | PDE10A |
| ENSG00000168685 | -0.61208449 | 4.17E-05 | 0.003378 | IL7R |
| ENSG00000261888 | -1.048232968 | 4.27E-05 | 0.003446 | AC144831.1 |
| ENSG00000185745 | -0.840008239 | 4.36E-05 | 0.003496 | IFIT1 |
| ENSG00000151892 | -0.97413756 | 4.65E-05 | 0.003712 | GFRA1 |
| ENSG00000261801 | -0.558070021 | 4.74E-05 | 0.003764 | LOXL1-AS1 |
| ENSG00000119900 | -0.496206364 | 4.83E-05 | 0.003812 | OGFRL1 |
| ENSG00000136826 | -0.643843858 | 5.00E-05 | 0.003925 | KLF4 |
| ENSG00000078269 | 0.57827267 | 5.24E-05 | 0.004088 | SYNJ2 |
| ENSG00000157193 | 0.894136862 | 5.36E-05 | 0.004161 | LRP8 |
| ENSG00000204103 | 0.909331403 | 5.39E-05 | 0.004161 | MAFB |
| ENSG00000170421 | -0.68029661 | 5.47E-05 | 0.004207 | KRT8 |
| ENSG00000175745 | -0.935543993 | 5.54E-05 | 0.004234 | NR2F1 |
| ENSG00000128641 | 0.531622994 | 5.87E-05 | 0.004463 | MYO1B |
| ENSG00000154803 | 0.512469668 | 5.90E-05 | 0.004465 | FLCN |
| ENSG00000115844 | 1.181008328 | 5.96E-05 | 0.004488 | DLX2 |
| ENSG00000158106 | -0.81318718 | 6.04E-05 | 0.004524 | RHPN1 |
| ENSG00000160179 | -1.343973908 | 6.24E-05 | 0.004645 | ABCG1 |
| ENSG00000137831 | -0.405617392 | 6.26E-05 | 0.004645 | UACA |
| ENSG00000004838 | -1.033631766 | 6.36E-05 | 0.004694 | ZMYND10 |
| ENSG00000021645 | -1.585648082 | 6.43E-05 | 0.004722 | NRXN3 |
| ENSG00000176720 | -0.552359765 | 6.46E-05 | 0.004722 | BOK |
| ENSG00000178860 | 1.222165036 | 6.53E-05 | 0.004746 | MSC |
| ENSG00000102996 | -0.572621823 | 6.60E-05 | 0.004772 | MMP15 |
| ENSG00000130270 | -0.775551198 | 6.89E-05 | 0.00496 | ATP8B3 |
| ENSG00000188747 | -0.90240893 | 7.07E-05 | 0.005068 | NOXA1 |
| ENSG00000253661 | -1.622611013 | 7.48E-05 | 0.005333 | ZFHX4-AS1 |
| ENSG00000110723 | 0.747720731 | 8.03E-05 | 0.005694 | EXPH5 |
| ENSG00000105877 | -1.367126056 | 8.08E-05 | 0.005708 | DNAH11 |
| ENSG00000141753 | -0.721245134 | 8.16E-05 | 0.005734 | IGFBP4 |
| ENSG00000100439 | 0.556276524 | 8.28E-05 | 0.005788 | ABHD4 |
| ENSG00000075618 | -0.421729163 | 8.35E-05 | 0.005814 | FSCN1 |
| ENSG00000135931 | 0.65953231 | 8.79E-05 | 0.00608 | ARMC9 |
| ENSG00000149573 | -1.125085647 | 8.82E-05 | 0.00608 | MPZL2 |
| ENSG00000171365 | 0.91565893 | 9.01E-05 | 0.006186 | CLCN5 |
| ENSG00000196411 | -0.454341385 | 9.15E-05 | 0.006251 | EPHB4 |
| ENSG00000167703 | -0.528950125 | 9.20E-05 | 0.00626 | SLC43A2 |
| ENSG00000101096 | 1.593445403 | 9.76E-05 | 0.006606 | NFATC2 |
| ENSG00000147862 | -0.424904476 | 9.99E-05 | 0.006731 | NFIB |
| ENSG00000104419 | -0.66105616 | 0.000102 | 0.006867 | NDRG1 |
| ENSG00000079263 | 1.910352762 | 0.000106 | 0.007091 | SP140 |
| ENSG00000104899 | -0.661986001 | 0.000107 | 0.007143 | AMH |
| ENSG00000138166 | -0.657903996 | 0.000113 | 0.00751 | DUSP5 |
| ENSG00000091490 | 0.767384602 | 0.000116 | 0.007653 | SEL1L3 |
| ENSG00000152894 | 0.715017186 | 0.000118 | 0.007738 | PTPRK |
| ENSG00000177666 | -0.429901014 | 0.000124 | 0.008125 | PNPLA2 |
| ENSG00000104142 | 0.507781825 | 0.000135 | 0.008762 | VPS18 |
| ENSG00000188042 | 0.601748606 | 0.000136 | 0.008783 | ARL4C |
| ENSG00000165474 | -1.277851394 | 0.000138 | 0.008871 | GJB2 |
| ENSG00000112715 | 0.556915072 | 0.000139 | 0.008879 | VEGFA |
| ENSG00000107281 | -0.723543682 | 0.000139 | 0.008879 | NPDC1 |
| ENSG00000108602 | 1.53360797 | 0.00014 | 0.008879 | ALDH3A1 |
| ENSG00000088826 | -0.545099437 | 0.000141 | 0.008945 | SMOX |
| ENSG00000107104 | 0.797978772 | 0.000149 | 0.009382 | KANK1 |
| ENSG00000152582 | -0.79902184 | 0.000149 | 0.009382 | SPEF2 |
| ENSG00000228253 | 1.074140769 | 0.000152 | 0.009504 | MT-ATP8 |
| ENSG00000072210 | 0.464680808 | 0.000155 | 0.009632 | ALDH3A2 |
| ENSG00000110031 | 0.883422244 | 0.000162 | 0.010063 | LPXN |
| ENSG00000154639 | -0.83774027 | 0.000165 | 0.010211 | CXADR |
| ENSG00000204682 | -0.929610717 | 0.000166 | 0.01022 | CASC10 |
| ENSG00000050344 | -0.494684764 | 0.000176 | 0.010766 | NFE2L3 |
| ENSG00000103196 | 1.141421061 | 0.000177 | 0.010766 | CRISPLD2 |
| ENSG00000008083 | 1.010992363 | 0.000179 | 0.010835 | JARID2 |
| ENSG00000162645 | -0.650239254 | 0.000179 | 0.010835 | GBP2 |
| ENSG00000197457 | -0.470238277 | 0.000186 | 0.011187 | STMN3 |
| ENSG00000255150 | 1.501882238 | 0.000188 | 0.01128 | EID3 |
| ENSG00000211448 | -0.904386962 | 0.000191 | 0.011433 | DIO2 |
| ENSG00000005059 | -0.622542358 | 0.000194 | 0.011521 | MCUB |
| ENSG00000166825 | 0.733228787 | 0.000196 | 0.011588 | ANPEP |
| ENSG00000196139 | 1.230115584 | 0.0002 | 0.011824 | AKR1C3 |
| ENSG00000182010 | -0.502126468 | 0.000202 | 0.011855 | RTKN2 |
| ENSG00000187608 | -1.051387622 | 0.000203 | 0.011869 | ISG15 |
| ENSG00000115468 | -1.252905309 | 0.000203 | 0.011869 | EFHD1 |
| ENSG00000088340 | -0.990690631 | 0.000205 | 0.011905 | FER1L4 |
| ENSG00000117525 | 0.469007488 | 0.000208 | 0.012033 | F3 |
| ENSG00000198417 | 0.975676521 | 0.000213 | 0.012302 | MT1F |
| ENSG00000143469 | 0.600186194 | 0.000217 | 0.012447 | SYT14 |
| ENSG00000197766 | -1.244597862 | 0.00022 | 0.012579 | CFD |
| ENSG00000182272 | -0.500886703 | 0.000221 | 0.012579 | B4GALNT4 |
| ENSG00000105516 | -0.928044833 | 0.000223 | 0.012671 | DBP |
| ENSG00000238113 | 1.299836333 | 0.000229 | 0.012971 | LINC01410 |
| ENSG00000128422 | -0.698127882 | 0.000245 | 0.013787 | KRT17 |
| ENSG00000127528 | -0.727554409 | 0.000249 | 0.013922 | KLF2 |
| ENSG00000189057 | -0.494566387 | 0.000249 | 0.013922 | FAM111B |
| ENSG00000157625 | 0.574055566 | 0.000252 | 0.014011 | TAB3 |
| ENSG00000174804 | -0.697184116 | 0.000256 | 0.014213 | FZD4 |
| ENSG00000180537 | -0.597835367 | 0.000265 | 0.014674 | RNF182 |
| ENSG00000176171 | -0.684405358 | 0.00027 | 0.01489 | BNIP3 |
| ENSG00000104765 | -0.517697108 | 0.000272 | 0.014926 | BNIP3L |
| ENSG00000189410 | 0.496476975 | 0.000275 | 0.015045 | SH2D5 |
| ENSG00000102804 | 0.462188419 | 0.000278 | 0.015099 | TSC22D1 |
| ENSG00000124107 | -1.549113952 | 0.000278 | 0.015099 | SLPI |
| ENSG00000153048 | -0.582649207 | 0.000285 | 0.015417 | CARHSP1 |
| ENSG00000100605 | -0.35952074 | 0.000289 | 0.015556 | ITPK1 |
| ENSG00000163131 | 0.64419616 | 0.000296 | 0.015858 | CTSS |
| ENSG00000059145 | 0.563647748 | 0.000297 | 0.015858 | UNKL |
| ENSG00000007968 | -0.547772097 | 0.000302 | 0.016066 | E2F2 |
| ENSG00000101665 | 0.860470862 | 0.000306 | 0.016223 | SMAD7 |
| ENSG00000177425 | -0.468020358 | 0.000313 | 0.016551 | PAWR |
| ENSG00000123080 | -0.663910222 | 0.000314 | 0.016559 | CDKN2C |
| ENSG00000142089 | -0.628435685 | 0.000321 | 0.016851 | IFITM3 |
| ENSG00000172915 | 0.931669465 | 0.000331 | 0.017273 | NBEA |
| ENSG00000251381 | 0.701332054 | 0.000332 | 0.017273 | LINC00958 |
| ENSG00000136235 | 0.401386364 | 0.000332 | 0.017273 | GPNMB |
| ENSG00000047617 | -0.960234465 | 0.000342 | 0.017695 | ANO2 |
| ENSG00000112137 | 0.812123732 | 0.000343 | 0.017712 | PHACTR1 |
| ENSG00000130787 | -0.549654483 | 0.000347 | 0.01782 | HIP1R |
| ENSG00000183765 | -0.749082487 | 0.000348 | 0.01784 | CHEK2 |
| ENSG00000169245 | -0.863889129 | 0.000349 | 0.01784 | CXCL10 |
| ENSG00000171492 | -0.415586927 | 0.000353 | 0.017982 | LRRC8D |
| ENSG00000150551 | -0.816557516 | 0.000363 | 0.018412 | LYPD1 |
| ENSG00000214114 | -0.898158547 | 0.000365 | 0.01845 | MYCBP |
| ENSG00000108861 | 0.369745954 | 0.00037 | 0.018614 | DUSP3 |
| ENSG00000072310 | -0.666843522 | 0.000379 | 0.019026 | SREBF1 |
| ENSG00000159263 | 0.797139839 | 0.000394 | 0.019692 | SIM2 |
| ENSG00000150782 | -1.124070546 | 0.000397 | 0.019809 | IL18 |
| ENSG00000164220 | 1.341138165 | 0.000401 | 0.019909 | F2RL2 |
| ENSG00000279118 | -0.749086386 | 0.000402 | 0.019909 | AC093535.2 |
| ENSG00000135111 | 0.826702484 | 0.000413 | 0.020351 | TBX3 |
| ENSG00000109586 | -0.407107927 | 0.000414 | 0.020351 | GALNT7 |
| ENSG00000171522 | -0.733196148 | 0.00042 | 0.02057 | PTGER4 |
| ENSG00000185201 | -0.583000306 | 0.000451 | 0.021905 | IFITM2 |
| ENSG00000196730 | -0.796035102 | 0.000451 | 0.021905 | DAPK1 |
| ENSG00000141655 | 0.57784843 | 0.000451 | 0.021905 | TNFRSF11A |
| ENSG00000183087 | -0.50657667 | 0.00046 | 0.022245 | GAS6 |
| ENSG00000050730 | 1.690078821 | 0.000461 | 0.022245 | TNIP3 |
| ENSG00000128284 | -0.617796939 | 0.000468 | 0.022482 | APOL3 |
| ENSG00000099849 | -0.579982203 | 0.000473 | 0.022676 | RASSF7 |
| ENSG00000113083 | -0.592078571 | 0.000475 | 0.022676 | LOX |
| ENSG00000100307 | -0.464331322 | 0.000478 | 0.022736 | CBX7 |
| ENSG00000137962 | -0.385020882 | 0.000483 | 0.022922 | ARHGAP29 |
| ENSG00000091073 | -0.505476244 | 0.000494 | 0.023385 | DTX2 |
| ENSG00000129757 | -1.409708252 | 0.000504 | 0.02374 | CDKN1C |
| ENSG00000137628 | -0.444675578 | 0.000542 | 0.025469 | DDX60 |
| ENSG00000101680 | 1.186198324 | 0.000548 | 0.025679 | LAMA1 |
| ENSG00000135905 | 0.608839226 | 0.000557 | 0.026 | DOCK10 |
| ENSG00000169504 | -0.348496381 | 0.000559 | 0.026 | CLIC4 |
| ENSG00000128578 | 0.577780839 | 0.000565 | 0.026208 | STRIP2 |
| ENSG00000091972 | -0.937882184 | 0.000567 | 0.026208 | CD200 |
| ENSG00000197635 | 0.804808874 | 0.000575 | 0.026509 | DPP4 |
| ENSG00000093217 | 0.775172614 | 0.000585 | 0.026896 | XYLB |
| ENSG00000070081 | -0.49490544 | 0.000598 | 0.027404 | NUCB2 |
| ENSG00000110719 | -0.420032939 | 0.000618 | 0.028221 | TCIRG1 |
| ENSG00000112773 | -0.468474833 | 0.000621 | 0.028298 | TENT5A |
| ENSG00000254612 | -1.662382063 | 0.000626 | 0.028422 | AP001000.1 |
| ENSG00000101187 | -0.816565816 | 0.000628 | 0.028422 | SLCO4A1 |
| ENSG00000109736 | -0.404843672 | 0.000632 | 0.028496 | MFSD10 |
| ENSG00000118960 | 0.436222907 | 0.000633 | 0.028496 | HS1BP3 |
| ENSG00000073756 | 1.53567269 | 0.000642 | 0.028815 | PTGS2 |
| ENSG00000126790 | 0.509301031 | 0.000652 | 0.029119 | L3HYPDH |
| ENSG00000170458 | -0.9063227 | 0.000653 | 0.029119 | CD14 |
| ENSG00000173432 | -1.017506624 | 0.000655 | 0.029119 | SAA1 |
| ENSG00000188064 | 1.033223787 | 0.00066 | 0.02927 | WNT7B |
| ENSG00000083799 | 0.382676483 | 0.000668 | 0.029503 | CYLD |
| ENSG00000183255 | -0.50032658 | 0.000679 | 0.029926 | PTTG1IP |
| ENSG00000133519 | -1.078675761 | 0.000686 | 0.030108 | ZDHHC8P1 |
| ENSG00000172164 | -0.806417824 | 0.000688 | 0.030108 | SNTB1 |
| ENSG00000148677 | -1.001678657 | 0.000702 | 0.030645 | ANKRD1 |
| ENSG00000149591 | -0.527611713 | 0.000705 | 0.030675 | TAGLN |
| ENSG00000226419 | 0.588434725 | 0.000713 | 0.030889 | SLC16A1-AS1 |
| ENSG00000151725 | -0.646889482 | 0.000714 | 0.030889 | CENPU |
| ENSG00000146674 | -0.779617232 | 0.000717 | 0.03095 | IGFBP3 |
| ENSG00000075461 | -0.620467306 | 0.000737 | 0.031598 | CACNG4 |
| ENSG00000144476 | -1.038331947 | 0.000738 | 0.031598 | ACKR3 |
| ENSG00000128274 | -0.681264741 | 0.000739 | 0.031598 | A4GALT |
| ENSG00000124216 | 1.064969063 | 0.000742 | 0.031666 | SNAI1 |
| ENSG00000185630 | -0.96071582 | 0.000756 | 0.032162 | PBX1 |
| ENSG00000241494 | 1.56016524 | 0.000767 | 0.032529 | AL355032.1 |
| ENSG00000072163 | -0.723833898 | 0.00077 | 0.03257 | LIMS2 |
| ENSG00000105499 | 1.269214565 | 0.000775 | 0.032669 | PLA2G4C |
| ENSG00000035681 | 0.366316547 | 0.000796 | 0.03346 | NSMAF |
| ENSG00000138018 | 0.470579679 | 0.000799 | 0.033506 | SELENOI |
| ENSG00000145687 | -0.61315975 | 0.00081 | 0.03388 | SSBP2 |
| ENSG00000157617 | 0.615341082 | 0.000816 | 0.034003 | C2CD2 |
| ENSG00000169122 | 0.804665195 | 0.000838 | 0.034834 | FAM110B |
| ENSG00000137801 | 0.680195638 | 0.000864 | 0.035834 | THBS1 |
| ENSG00000197989 | -0.632779954 | 0.000873 | 0.036101 | SNHG12 |
| ENSG00000087842 | 0.990657199 | 0.000881 | 0.036132 | PIR |
| ENSG00000158715 | 0.84353085 | 0.000881 | 0.036132 | SLC45A3 |
| ENSG00000137309 | 0.448188981 | 0.000882 | 0.036132 | HMGA1 |
| ENSG00000058085 | 0.827856894 | 0.000884 | 0.036132 | LAMC2 |
| ENSG00000143153 | -0.48236288 | 0.00089 | 0.036281 | ATP1B1 |
| ENSG00000062582 | 0.630989566 | 0.000894 | 0.036338 | MRPS24 |
| ENSG00000147813 | -0.545367488 | 0.000901 | 0.036484 | NAPRT |
| ENSG00000184178 | 0.531694396 | 0.000902 | 0.036484 | SCFD2 |
| ENSG00000146281 | -0.432502694 | 0.000909 | 0.03665 | PM20D2 |
| ENSG00000168297 | 0.547237405 | 0.000937 | 0.037667 | PXK |
| ENSG00000092964 | -0.410930193 | 0.000944 | 0.037847 | DPYSL2 |
| ENSG00000089505 | 0.836153678 | 0.000947 | 0.037863 | CMTM1 |
| ENSG00000185924 | -0.978687128 | 0.000956 | 0.038116 | RTN4RL1 |
| ENSG00000132170 | 0.680883183 | 0.000964 | 0.03834 | PPARG |
| ENSG00000175592 | 0.727316457 | 0.000973 | 0.03859 | FOSL1 |
| ENSG00000165244 | -0.424505092 | 0.000983 | 0.038874 | ZNF367 |
| ENSG00000125398 | -0.535659527 | 0.000986 | 0.038908 | SOX9 |
| ENSG00000198768 | 1.210427243 | 0.000998 | 0.039262 | APCDD1L |
| ENSG00000107968 | -0.554424612 | 0.001006 | 0.039476 | MAP3K8 |
| ENSG00000135480 | -0.802019273 | 0.001011 | 0.039572 | KRT7 |
| ENSG00000165312 | -0.591321848 | 0.001041 | 0.040639 | OTUD1 |
| ENSG00000160014 | -0.345224774 | 0.001045 | 0.04067 | CALM3 |
| ENSG00000198732 | -1.49350906 | 0.001047 | 0.04067 | SMOC1 |
| ENSG00000107438 | -0.529392207 | 0.001064 | 0.041212 | PDLIM1 |
| ENSG00000187193 | 1.188132787 | 0.00107 | 0.041344 | MT1X |
| ENSG00000187244 | -0.406546939 | 0.001076 | 0.04145 | BCAM |
| ENSG00000104368 | -0.561085639 | 0.001079 | 0.041479 | PLAT |
| ENSG00000146054 | -0.683828439 | 0.001087 | 0.041673 | TRIM7 |
| ENSG00000114626 | -0.650275131 | 0.001097 | 0.041942 | ABTB1 |
| ENSG00000164823 | 0.472444877 | 0.001108 | 0.04226 | OSGIN2 |
| ENSG00000177469 | -0.322096596 | 0.001114 | 0.042361 | CAVIN1 |
| ENSG00000145743 | 0.460702044 | 0.001121 | 0.042533 | FBXL17 |
| ENSG00000119917 | -0.401761654 | 0.001155 | 0.043686 | IFIT3 |
| ENSG00000170088 | 0.428543208 | 0.001162 | 0.04384 | TMEM192 |
| ENSG00000132561 | -0.60407108 | 0.001199 | 0.045138 | MATN2 |
| ENSG00000153714 | 0.754889103 | 0.001202 | 0.045138 | LURAP1L |
| ENSG00000121236 | -0.80011784 | 0.00121 | 0.04515 | TRIM6 |
| ENSG00000116191 | -0.381171949 | 0.001211 | 0.04515 | RALGPS2 |
| ENSG00000150054 | -0.783576768 | 0.001212 | 0.04515 | MPP7 |
| ENSG00000168003 | 0.558975646 | 0.001239 | 0.045983 | SLC3A2 |
| ENSG00000070961 | 0.333624371 | 0.001242 | 0.045983 | ATP2B1 |
| ENSG00000163346 | -0.402268391 | 0.001243 | 0.045983 | PBXIP1 |
| ENSG00000243566 | -1.640864499 | 0.001262 | 0.04657 | UPK3B |
| ENSG00000130766 | 0.549613804 | 0.00127 | 0.046752 | SESN2 |
| ENSG00000155265 | 0.877816812 | 0.001293 | 0.047467 | GOLGA7B |
| ENSG00000137463 | -1.155821583 | 0.001327 | 0.048597 | MGARP |
| ENSG00000082497 | -0.799596107 | 0.001359 | 0.049535 | SERTAD4 |
| ENSG00000101255 | 0.450566949 | 0.001359 | 0.049535 | TRIB3 |
