# Supplementary material for: PM2.5 promotes NSCLC carcinogenesis through translationally and transcriptionally activating DLAT-mediated glycolysis reprograming
Source: J Exp Clin Cancer Res. 2022 Jul 22;41:229. doi: 10.1186/s13046-022-02437-8 (PMC9308224; doi:10.1186/s13046-022-02437-8)
Supplement: Supplementary file 11 — Additional file 11: Table S3. Differentially expressed ribosome-associated mRNAs regulated by PM2.5 in BEAS-2B cells. [file 13046_2022_2437_MOESM11_ESM.docx]

| **Table S3. Differentially expressed ribosome-associated mRNAs regulated by PM2.5 in BEAS-2B cells** | | | | | |
| --- | --- | --- | --- | --- | --- |
| **Gene_id** | **log2FoldChange** | **P value** | **Padj** | **Gene name** | |
| ENSG00000125148 | 4.639665268 | 7.64E-143 | 1.10E-138 | MT2A |  |
| ENSG00000125977 | -2.698828506 | 2.22E-104 | 1.60E-100 | EIF2S2 |  |
| ENSG00000275714 | 2.948528585 | 1.77E-95 | 8.50E-92 | HIST1H3A |  |
| ENSG00000118181 | -2.951406897 | 4.51E-93 | 1.63E-89 | RPS25 |  |
| ENSG00000138061 | 3.489312048 | 2.50E-91 | 7.23E-88 | CYP1B1 |  |
| ENSG00000148773 | -2.350181974 | 7.50E-89 | 1.81E-85 | MKI67 |  |
| ENSG00000235408 | -2.816318788 | 5.28E-88 | 1.09E-84 | SNORA71B |  |
| ENSG00000164104 | -2.796328833 | 2.09E-87 | 3.78E-84 | HMGB2 |  |
| ENSG00000255823 | -3.745828856 | 4.64E-82 | 7.45E-79 | MTRNR2L8 |  |
| ENSG00000138180 | -2.519589068 | 9.66E-82 | 1.39E-78 | CEP55 |  |
| ENSG00000102317 | 2.81204072 | 4.89E-81 | 6.42E-78 | RBM3 |  |
| ENSG00000140416 | -2.452725558 | 3.51E-78 | 4.23E-75 | TPM1 |  |
| ENSG00000115268 | 2.369287017 | 2.95E-76 | 3.28E-73 | RPS15 |  |
| ENSG00000163631 | -8.47353416 | 3.17E-75 | 3.27E-72 | ALB |  |
| ENSG00000200087 | -3.064503184 | 5.79E-69 | 5.58E-66 | SNORA73B |  |
| ENSG00000198467 | -2.114583201 | 1.06E-66 | 9.55E-64 | TPM2 |  |
| ENSG00000168003 | 2.008062357 | 3.00E-62 | 2.55E-59 | SLC3A2 |  |
| ENSG00000207994 | -3.581395793 | 8.31E-62 | 6.67E-59 | MIR100 |  |
| ENSG00000187109 | -2.166093357 | 1.91E-59 | 1.45E-56 | NAP1L1 |  |
| ENSG00000161011 | 2.085410027 | 4.46E-59 | 3.22E-56 | SQSTM1 |  |
| ENSG00000225921 | -2.691870921 | 1.09E-57 | 7.47E-55 | NOL7 |  |
| ENSG00000207523 | -2.468267259 | 3.84E-57 | 2.52E-54 | SNORA66 |  |
| ENSG00000126777 | -2.321225756 | 4.52E-57 | 2.84E-54 | KTN1 |  |
| ENSG00000119707 | -2.210395691 | 1.90E-56 | 1.14E-53 | RBM25 |  |
| ENSG00000165733 | -2.503653144 | 4.10E-56 | 2.37E-53 | BMS1 |  |
| ENSG00000117395 | -2.485437656 | 2.91E-55 | 1.61E-52 | EBNA1BP2 |  |
| ENSG00000069275 | -1.897470204 | 6.00E-55 | 3.21E-52 | NUCKS1 |  |
| ENSG00000153113 | -2.324137924 | 1.30E-54 | 6.71E-52 | CAST |  |
| ENSG00000184357 | -1.726710894 | 4.00E-54 | 1.99E-51 | HIST1H1B |  |
| ENSG00000212158 | -2.584714571 | 5.82E-54 | 2.80E-51 | SNORD66 |  |
| ENSG00000207638 | -2.373380817 | 8.51E-54 | 3.97E-51 | MIR99A |  |
| ENSG00000141458 | 2.017065753 | 1.48E-53 | 6.70E-51 | NPC1 |  |
| ENSG00000009954 | -2.16374072 | 2.75E-53 | 1.21E-50 | BAZ1B |  |
| ENSG00000147231 | -6.592790591 | 4.49E-53 | 1.91E-50 | CXorf57 |  |
| ENSG00000175550 | -2.150048744 | 2.41E-52 | 9.94E-50 | DRAP1 |  |
| ENSG00000203814 | -3.151136451 | 5.65E-52 | 2.27E-49 | HIST2H2BF | |
| ENSG00000200354 | -3.133966795 | 3.22E-51 | 1.26E-48 | SNORA71D |  |
| ENSG00000006327 | 1.968950177 | 2.21E-50 | 8.41E-48 | TNFRSF12A | |
| ENSG00000131389 | 2.42474265 | 5.78E-49 | 2.14E-46 | SLC6A6 |  |
| ENSG00000200816 | -3.546246132 | 1.56E-47 | 5.62E-45 | SNORA38 |  |
| ENSG00000200296 | -2.362415216 | 2.71E-47 | 9.56E-45 | RNU1-83P |  |
| ENSG00000169429 | 2.018156994 | 1.52E-46 | 5.22E-44 | CXCL8 |  |
| ENSG00000133226 | -2.483980565 | 2.14E-46 | 7.19E-44 | SRRM1 |  |
| ENSG00000047410 | -1.897069508 | 2.86E-46 | 9.40E-44 | TPR |  |
| ENSG00000135404 | 1.862400001 | 7.20E-46 | 2.31E-43 | CD63 |  |
| ENSG00000137776 | -2.400083801 | 1.62E-45 | 5.10E-43 | SLTM |  |
| ENSG00000201518 | -3.962587315 | 4.89E-44 | 1.50E-41 | RNA5SP513 | |
| ENSG00000164985 | -2.313852207 | 5.74E-44 | 1.73E-41 | PSIP1 |  |
| ENSG00000221740 | -2.416865306 | 7.12E-44 | 2.10E-41 | SNORD93 |  |
| ENSG00000188529 | -2.1601578 | 5.10E-43 | 1.47E-40 | SRSF10 |  |
| ENSG00000130779 | -1.965397185 | 2.02E-42 | 5.72E-40 | CLIP1 |  |
| ENSG00000234741 | -2.090197532 | 3.18E-42 | 8.72E-40 | GAS5 |  |
| ENSG00000196504 | -1.945484227 | 3.20E-42 | 8.72E-40 | PRPF40A |  |
| ENSG00000202031 | -2.910546655 | 6.16E-41 | 1.65E-38 | SNORD38A |  |
| ENSG00000101421 | -1.947879701 | 7.76E-41 | 2.04E-38 | CHMP4B |  |
| ENSG00000070081 | -2.320725798 | 1.08E-40 | 2.78E-38 | NUCB2 |  |
| ENSG00000108518 | 1.679726627 | 1.99E-40 | 5.06E-38 | PFN1 |  |
| ENSG00000207280 | -3.529864816 | 3.53E-40 | 8.79E-38 | SNORD20 |  |
| ENSG00000064042 | -2.387539485 | 9.26E-40 | 2.26E-37 | LIMCH1 |  |
| ENSG00000164609 | -2.50926142 | 9.38E-40 | 2.26E-37 | SLU7 |  |
| ENSG00000197238 | 1.726725734 | 1.38E-39 | 3.26E-37 | HIST1H4J |  |
| ENSG00000091513 | -6.914209769 | 2.53E-39 | 5.89E-37 | TF |  |
| ENSG00000141867 | -2.050252671 | 2.68E-39 | 6.16E-37 | BRD4 |  |
| ENSG00000169710 | 1.591953302 | 3.51E-39 | 7.93E-37 | FASN |  |
| ENSG00000040275 | -2.28365315 | 1.01E-38 | 2.24E-36 | SPDL1 |  |
| ENSG00000201201 | -7.469828931 | 2.40E-38 | 5.26E-36 | RN7SKP118 | |
| ENSG00000207725 | -1.975251403 | 4.53E-38 | 9.77E-36 | MIR222 |  |
| ENSG00000115241 | -1.786373732 | 5.63E-38 | 1.20E-35 | PPM1G |  |
| ENSG00000134046 | -2.507746385 | 7.93E-38 | 1.66E-35 | MBD2 |  |
| ENSG00000108848 | -1.980786874 | 1.30E-37 | 2.69E-35 | LUC7L3 |  |
| ENSG00000142227 | 1.834366837 | 7.26E-37 | 1.48E-34 | EMP3 |  |
| ENSG00000170860 | 1.904181411 | 8.65E-37 | 1.74E-34 | LSM3 |  |
| ENSG00000137331 | 2.373705874 | 3.67E-36 | 7.25E-34 | IER3 |  |
| ENSG00000080824 | -1.6134638 | 7.02E-36 | 1.37E-33 | HSP90AA1 |  |
| ENSG00000129680 | -2.595051933 | 8.33E-36 | 1.60E-33 | MAP7D3 |  |
| ENSG00000207605 | -3.273446928 | 1.01E-35 | 1.92E-33 | MIR191 |  |
| ENSG00000072501 | -1.680764012 | 1.67E-35 | 3.14E-33 | SMC1A |  |
| ENSG00000103257 | 1.585504866 | 2.45E-35 | 4.55E-33 | SLC7A5 |  |
| ENSG00000277754 | -7.799878192 | 3.75E-35 | 6.87E-33 | RF00012 |  |
| ENSG00000199150 | -2.27638395 | 3.95E-35 | 7.13E-33 | MIRLET7G |  |
| ENSG00000203875 | -1.72446441 | 4.23E-35 | 7.54E-33 | SNHG5 |  |
| ENSG00000123200 | -2.008844547 | 1.03E-34 | 1.82E-32 | ZC3H13 |  |
| ENSG00000142864 | -1.844906431 | 3.07E-34 | 5.34E-32 | SERBP1 |  |
| ENSG00000138758 | -2.015065807 | 3.23E-34 | 5.56E-32 | 11-Sep |  |
| ENSG00000238363 | -2.10748417 | 3.88E-34 | 6.59E-32 | SNORA13 |  |
| ENSG00000117394 | 1.674565515 | 1.11E-33 | 1.86E-31 | SLC2A1 |  |
| ENSG00000137815 | -2.018435621 | 1.45E-33 | 2.41E-31 | RTF1 |  |
| ENSG00000141526 | 1.644111068 | 5.08E-33 | 8.33E-31 | SLC16A3 |  |
| ENSG00000158417 | -1.906726722 | 1.04E-32 | 1.69E-30 | EIF5B |  |
| ENSG00000134910 | 1.70505549 | 2.03E-32 | 3.26E-30 | STT3A |  |
| ENSG00000141367 | 1.387901811 | 2.51E-32 | 3.99E-30 | CLTC |  |
| ENSG00000101361 | -1.909119301 | 2.63E-32 | 4.13E-30 | NOP56 |  |
| ENSG00000149503 | -1.668072083 | 2.97E-32 | 4.60E-30 | INCENP |  |
| ENSG00000197081 | 1.516395013 | 2.99E-32 | 4.60E-30 | IGF2R |  |
| ENSG00000201998 | -4.238435206 | 4.17E-32 | 6.35E-30 | SNORA23 |  |
| ENSG00000204138 | -2.218526187 | 5.67E-32 | 8.53E-30 | PHACTR4 |  |
| ENSG00000163950 | -1.987799593 | 5.82E-32 | 8.67E-30 | SLBP |  |
| ENSG00000105281 | 1.928307378 | 1.02E-31 | 1.51E-29 | SLC1A5 |  |
| ENSG00000272533 | -2.399462615 | 1.12E-31 | 1.62E-29 | SNORA28 |  |
| ENSG00000197694 | -1.324753844 | 1.12E-31 | 1.62E-29 | SPTAN1 |  |
| ENSG00000087365 | -1.647800806 | 1.14E-31 | 1.64E-29 | SF3B2 |  |
| ENSG00000198176 | -1.847922547 | 1.62E-31 | 2.29E-29 | TFDP1 |  |
| ENSG00000117523 | -1.695540026 | 1.69E-31 | 2.37E-29 | PRRC2C |  |
| ENSG00000114446 | -2.36797637 | 3.23E-31 | 4.48E-29 | IFT57 |  |
| ENSG00000202314 | -1.962212038 | 4.21E-31 | 5.79E-29 | SNORD6 |  |
| ENSG00000252010 | -1.845484818 | 4.56E-31 | 6.21E-29 | SCARNA5 |  |
| ENSG00000136872 | -7.390895707 | 8.38E-31 | 1.13E-28 | ALDOB |  |
| ENSG00000197632 | 4.351431897 | 8.51E-31 | 1.14E-28 | SERPINB2 |  |
| ENSG00000038382 | -1.485470402 | 9.25E-31 | 1.23E-28 | TRIO |  |
| ENSG00000235162 | -1.992110736 | 1.25E-30 | 1.64E-28 | C12orf75 |  |
| ENSG00000196611 | 2.395254534 | 2.75E-30 | 3.58E-28 | MMP1 |  |
| ENSG00000057252 | 2.089571617 | 5.11E-30 | 6.58E-28 | SOAT1 |  |
| ENSG00000115677 | -1.368091714 | 5.15E-30 | 6.58E-28 | HDLBP |  |
| ENSG00000187514 | -1.344526309 | 6.00E-30 | 7.60E-28 | PTMA |  |
| ENSG00000144674 | -1.708593218 | 8.13E-30 | 1.02E-27 | GOLGA4 |  |
| ENSG00000109111 | -1.530198488 | 1.11E-29 | 1.39E-27 | SUPT6H |  |
| ENSG00000212402 | -3.979540391 | 1.49E-29 | 1.83E-27 | SNORA74B |  |
| ENSG00000084674 | -6.021592373 | 1.62E-29 | 1.99E-27 | APOB |  |
| ENSG00000251898 | -2.341714237 | 3.79E-29 | 4.60E-27 | SCARNA11 |  |
| ENSG00000198431 | 1.540871422 | 4.17E-29 | 5.02E-27 | TXNRD1 |  |
| ENSG00000156802 | -1.374300604 | 5.77E-29 | 6.89E-27 | ATAD2 |  |
| ENSG00000143409 | -4.479452742 | 8.69E-29 | 1.03E-26 | MINDY1 |  |
| ENSG00000113739 | 1.803794337 | 1.12E-28 | 1.32E-26 | STC2 |  |
| ENSG00000175467 | -1.711359789 | 1.14E-28 | 1.32E-26 | SART1 |  |
| ENSG00000139644 | 1.42063715 | 1.22E-28 | 1.41E-26 | TMBIM6 |  |
| ENSG00000166598 | -1.481059311 | 1.78E-28 | 2.04E-26 | HSP90B1 |  |
| ENSG00000156535 | 1.906983016 | 1.82E-28 | 2.07E-26 | CD109 |  |
| ENSG00000198987 | -2.941392181 | 1.87E-28 | 2.11E-26 | MIR16-2 |  |
| ENSG00000136819 | -2.777612336 | 2.47E-28 | 2.76E-26 | C9orf78 |  |
| ENSG00000130816 | -1.369108927 | 2.93E-28 | 3.25E-26 | DNMT1 |  |
| ENSG00000166477 | -2.158200546 | 3.01E-28 | 3.31E-26 | LEO1 |  |
| ENSG00000088325 | -1.401602594 | 3.05E-28 | 3.34E-26 | TPX2 |  |
| ENSG00000167460 | -1.429589415 | 3.42E-28 | 3.72E-26 | TPM4 |  |
| ENSG00000171634 | -1.516712921 | 6.15E-28 | 6.63E-26 | BPTF |  |
| ENSG00000284231 | -3.540510276 | 8.42E-28 | 9.01E-26 | MIR424 |  |
| ENSG00000058272 | -1.881646128 | 1.04E-27 | 1.11E-25 | PPP1R12A |  |
| ENSG00000168298 | -1.245622464 | 1.65E-27 | 1.74E-25 | HIST1H1E |  |
| ENSG00000200394 | -3.591670264 | 1.66E-27 | 1.74E-25 | SNORA38B |  |
| ENSG00000151914 | -1.564716672 | 2.30E-27 | 2.39E-25 | DST |  |
| ENSG00000106244 | -1.7899299 | 2.48E-27 | 2.56E-25 | PDAP1 |  |
| ENSG00000087269 | -1.969358202 | 3.46E-27 | 3.54E-25 | NOP14 |  |
| ENSG00000163399 | 1.220323078 | 3.95E-27 | 4.02E-25 | ATP1A1 |  |
| ENSG00000169715 | 2.760615342 | 4.13E-27 | 4.18E-25 | MT1E |  |
| ENSG00000101161 | -1.721013668 | 7.72E-27 | 7.75E-25 | PRPF6 |  |
| ENSG00000017797 | -2.243905025 | 8.47E-27 | 8.44E-25 | RALBP1 |  |
| ENSG00000112419 | -1.73053737 | 9.13E-27 | 9.04E-25 | PHACTR2 |  |
| ENSG00000255112 | 2.088166552 | 1.21E-26 | 1.19E-24 | CHMP1B |  |
| ENSG00000276966 | 1.838584538 | 1.29E-26 | 1.26E-24 | HIST1H4E |  |
| ENSG00000102241 | -2.029611605 | 1.47E-26 | 1.43E-24 | HTATSF1 |  |
| ENSG00000283203 | -5.014331918 | 1.59E-26 | 1.53E-24 | MIR1246 |  |
| ENSG00000140575 | -1.294082867 | 1.98E-26 | 1.89E-24 | IQGAP1 |  |
| ENSG00000168724 | -2.359047182 | 2.77E-26 | 2.63E-24 | DNAJC21 |  |
| ENSG00000129518 | -2.359824685 | 3.19E-26 | 3.01E-24 | EAPP |  |
| ENSG00000221539 | -2.477175662 | 3.44E-26 | 3.23E-24 | SNORD99 |  |
| ENSG00000199266 | -3.696113221 | 3.73E-26 | 3.48E-24 | SNORA60 |  |
| ENSG00000105438 | 1.704687721 | 4.20E-26 | 3.89E-24 | KDELR1 |  |
| ENSG00000143549 | -1.45166477 | 4.24E-26 | 3.90E-24 | TPM3 |  |
| ENSG00000253729 | 1.154062534 | 4.58E-26 | 4.18E-24 | PRKDC |  |
| ENSG00000122545 | -1.565625424 | 4.71E-26 | 4.28E-24 | 7-Sep |  |
| ENSG00000186522 | -1.834273936 | 4.99E-26 | 4.50E-24 | 10-Sep |  |
| ENSG00000222355 | -3.043060905 | 5.31E-26 | 4.76E-24 | RNU2-29P |  |
| ENSG00000151012 | 2.521339846 | 6.02E-26 | 5.37E-24 | SLC7A11 |  |
| ENSG00000127483 | -1.488643611 | 1.06E-25 | 9.40E-24 | HP1BP3 |  |
| ENSG00000171530 | -1.617374073 | 1.36E-25 | 1.20E-23 | TBCA |  |
| ENSG00000091527 | -1.687139908 | 1.47E-25 | 1.29E-23 | CDV3 |  |
| ENSG00000099901 | -1.981683565 | 1.51E-25 | 1.31E-23 | RANBP1 |  |
| ENSG00000144118 | -1.87207562 | 3.88E-25 | 3.35E-23 | RALB |  |
| ENSG00000133789 | -1.712765993 | 4.67E-25 | 4.01E-23 | SWAP70 |  |
| ENSG00000208892 | -2.893236259 | 4.98E-25 | 4.25E-23 | SNORA49 |  |
| ENSG00000116754 | -1.577488019 | 5.07E-25 | 4.31E-23 | SRSF11 |  |
| ENSG00000198160 | -1.876751129 | 7.21E-25 | 6.09E-23 | MIER1 |  |
| ENSG00000176788 | -1.76410169 | 7.69E-25 | 6.46E-23 | BASP1 |  |
| ENSG00000278637 | 1.557511996 | 9.81E-25 | 8.19E-23 | HIST1H4A |  |
| ENSG00000167674 | -1.612739778 | 1.00E-24 | 8.31E-23 | HDGFL2 |  |
| ENSG00000147649 | -1.572795562 | 1.03E-24 | 8.46E-23 | MTDH |  |
| ENSG00000168439 | -1.476840197 | 1.44E-24 | 1.18E-22 | STIP1 |  |
| ENSG00000128487 | -1.960099404 | 1.65E-24 | 1.34E-22 | SPECC1 |  |
| ENSG00000277075 | 1.141477889 | 2.95E-24 | 2.39E-22 | HIST1H2AE | |
| ENSG00000107581 | -1.207304051 | 6.11E-24 | 4.93E-22 | EIF3A |  |
| ENSG00000139613 | -1.631287943 | 6.63E-24 | 5.32E-22 | SMARCC2 |  |
| ENSG00000146007 | -1.525746832 | 7.12E-24 | 5.68E-22 | ZMAT2 |  |
| ENSG00000101343 | -2.292565371 | 1.11E-23 | 8.79E-22 | CRNKL1 |  |
| ENSG00000198900 | -1.883822217 | 2.07E-23 | 1.64E-21 | TOP1 |  |
| ENSG00000207445 | -2.056984493 | 2.79E-23 | 2.19E-21 | SNORD15B |  |
| ENSG00000171560 | -6.604129972 | 3.73E-23 | 2.91E-21 | FGA |  |
| ENSG00000096401 | -1.721382244 | 3.78E-23 | 2.94E-21 | CDC5L |  |
| ENSG00000173473 | -1.530247193 | 4.07E-23 | 3.14E-21 | SMARCC1 |  |
| ENSG00000060339 | -1.554243332 | 4.88E-23 | 3.75E-21 | CCAR1 |  |
| ENSG00000118363 | 3.303182126 | 6.98E-23 | 5.34E-21 | SPCS2 |  |
| ENSG00000196535 | -1.815589317 | 8.29E-23 | 6.27E-21 | MYO18A |  |
| ENSG00000234176 | -2.108282055 | 8.29E-23 | 6.27E-21 | HSPA8P1 |  |
| ENSG00000065613 | -1.525205321 | 9.40E-23 | 7.07E-21 | SLK |  |
| ENSG00000100503 | -1.810847712 | 1.03E-22 | 7.74E-21 | NIN |  |
| ENSG00000122786 | -1.75199828 | 1.20E-22 | 8.91E-21 | CALD1 |  |
| ENSG00000278677 | 1.511012304 | 1.29E-22 | 9.57E-21 | HIST1H2AM | |
| ENSG00000166197 | -1.62729685 | 1.30E-22 | 9.57E-21 | NOLC1 |  |
| ENSG00000196305 | 1.438426664 | 1.36E-22 | 9.96E-21 | IARS |  |
| ENSG00000105323 | -1.413905799 | 1.37E-22 | 1.00E-20 | HNRNPUL1 |  |
| ENSG00000184640 | -1.313990065 | 1.39E-22 | 1.01E-20 | 9-Sep |  |
| ENSG00000149136 | -1.430083058 | 1.43E-22 | 1.04E-20 | SSRP1 |  |
| ENSG00000089597 | 1.287288552 | 1.48E-22 | 1.06E-20 | GANAB |  |
| ENSG00000004455 | 1.907406968 | 1.55E-22 | 1.11E-20 | AK2 |  |
| ENSG00000185697 | -1.714465774 | 2.69E-22 | 1.91E-20 | MYBL1 |  |
| ENSG00000230067 | -5.993909545 | 2.69E-22 | 1.91E-20 | HSPD1P6 |  |
| ENSG00000131711 | -1.145947408 | 2.92E-22 | 2.06E-20 | MAP1B |  |
| ENSG00000140259 | -2.062147485 | 3.98E-22 | 2.79E-20 | MFAP1 |  |
| ENSG00000135387 | -1.282328238 | 4.52E-22 | 3.15E-20 | CAPRIN1 |  |
| ENSG00000117724 | -1.197766315 | 4.92E-22 | 3.42E-20 | CENPF |  |
| ENSG00000108055 | -1.529358458 | 6.05E-22 | 4.18E-20 | SMC3 |  |
| ENSG00000276903 | 1.4272839 | 8.87E-22 | 6.10E-20 | HIST1H2AL | |
| ENSG00000141027 | -1.771839185 | 1.86E-21 | 1.28E-19 | NCOR1 |  |
| ENSG00000135250 | -1.772902666 | 1.96E-21 | 1.34E-19 | SRPK2 |  |
| ENSG00000199133 | -2.734014934 | 2.01E-21 | 1.36E-19 | MIRLET7D |  |
| ENSG00000068697 | 1.403195006 | 2.02E-21 | 1.37E-19 | LAPTM4A |  |
| ENSG00000011426 | -1.25175593 | 2.10E-21 | 1.41E-19 | ANLN |  |
| ENSG00000125304 | 1.300587852 | 2.91E-21 | 1.95E-19 | TM9SF2 |  |
| ENSG00000198918 | 2.240177502 | 3.53E-21 | 2.35E-19 | RPL39 |  |
| ENSG00000115306 | -1.224182021 | 3.69E-21 | 2.44E-19 | SPTBN1 |  |
| ENSG00000181019 | 1.602965991 | 3.70E-21 | 2.44E-19 | NQO1 |  |
| ENSG00000108592 | -1.378158832 | 4.14E-21 | 2.72E-19 | FTSJ3 |  |
| ENSG00000151247 | 1.994233563 | 4.22E-21 | 2.76E-19 | EIF4E |  |
| ENSG00000160299 | -1.639622934 | 4.45E-21 | 2.90E-19 | PCNT |  |
| ENSG00000183431 | -1.347946036 | 4.60E-21 | 2.98E-19 | SF3A3 |  |
| ENSG00000124253 | -6.192026845 | 5.38E-21 | 3.47E-19 | PCK1 |  |
| ENSG00000140743 | -1.814662439 | 5.68E-21 | 3.65E-19 | CDR2 |  |
| ENSG00000108312 | -1.513173313 | 5.91E-21 | 3.78E-19 | UBTF |  |
| ENSG00000124228 | -1.567203975 | 6.60E-21 | 4.20E-19 | DDX27 |  |
| ENSG00000197837 | 1.419608494 | 6.78E-21 | 4.30E-19 | HIST4H4 |  |
| ENSG00000044574 | -1.128245616 | 7.03E-21 | 4.43E-19 | HSPA5 |  |
| ENSG00000166986 | 1.459697008 | 9.20E-21 | 5.78E-19 | MARS |  |
| ENSG00000116560 | -1.280460792 | 1.13E-20 | 7.05E-19 | SFPQ |  |
| ENSG00000204209 | -1.749224415 | 1.22E-20 | 7.61E-19 | DAXX |  |
| ENSG00000277846 | -2.641088685 | 1.27E-20 | 7.90E-19 | SNORD30 |  |
| ENSG00000181163 | -1.188013502 | 1.37E-20 | 8.47E-19 | NPM1 |  |
| ENSG00000111897 | 1.62852176 | 1.55E-20 | 9.51E-19 | SERINC1 |  |
| ENSG00000138092 | 1.716465252 | 1.84E-20 | 1.12E-18 | CENPO |  |
| ENSG00000160310 | -1.958128703 | 1.94E-20 | 1.18E-18 | PRMT2 |  |
| ENSG00000113141 | -1.823840807 | 2.08E-20 | 1.26E-18 | IK |  |
| ENSG00000124383 | -2.563777994 | 2.80E-20 | 1.69E-18 | MPHOSPH10 | |
| ENSG00000147416 | 1.57080355 | 2.93E-20 | 1.76E-18 | ATP6V1B2 |  |
| ENSG00000131016 | -1.601005762 | 2.97E-20 | 1.78E-18 | AKAP12 |  |
| ENSG00000090615 | -1.4425851 | 3.25E-20 | 1.94E-18 | GOLGA3 |  |
| ENSG00000207973 | -3.287454399 | 3.51E-20 | 2.08E-18 | MIR589 |  |
| ENSG00000180879 | 1.488150871 | 4.14E-20 | 2.45E-18 | SSR4 |  |
| ENSG00000253190 | -2.074865613 | 5.63E-20 | 3.32E-18 | AC084082.1 | |
| ENSG00000234745 | 1.182962563 | 6.24E-20 | 3.66E-18 | HLA-B |  |
| ENSG00000144824 | -1.642200646 | 6.88E-20 | 4.02E-18 | PHLDB2 |  |
| ENSG00000129347 | -1.58477621 | 7.83E-20 | 4.56E-18 | KRI1 |  |
| ENSG00000221540 | -2.404264599 | 8.33E-20 | 4.83E-18 | MIR1180 |  |
| ENSG00000085719 | 1.772031293 | 9.53E-20 | 5.51E-18 | CPNE3 |  |
| ENSG00000008394 | 1.735427469 | 1.01E-19 | 5.80E-18 | MGST1 |  |
| ENSG00000125817 | -1.597349255 | 1.05E-19 | 5.99E-18 | CENPB |  |
| ENSG00000197249 | -3.976890789 | 1.05E-19 | 5.99E-18 | SERPINA1 |  |
| ENSG00000169925 | -2.405904488 | 1.10E-19 | 6.25E-18 | BRD3 |  |
| ENSG00000011260 | -1.699872512 | 1.12E-19 | 6.36E-18 | UTP18 |  |
| ENSG00000143753 | 1.979911443 | 1.20E-19 | 6.76E-18 | DEGS1 |  |
| ENSG00000071859 | -1.353050191 | 1.29E-19 | 7.24E-18 | FAM50A |  |
| ENSG00000092201 | -1.165423762 | 1.71E-19 | 9.57E-18 | SUPT16H |  |
| ENSG00000172270 | 1.083475133 | 2.08E-19 | 1.16E-17 | BSG |  |
| ENSG00000143401 | -1.323826097 | 2.09E-19 | 1.16E-17 | ANP32E |  |
| ENSG00000143252 | 1.952148166 | 2.17E-19 | 1.20E-17 | SDHC |  |
| ENSG00000136861 | -1.44991741 | 2.19E-19 | 1.21E-17 | CDK5RAP2 |  |
| ENSG00000199477 | -1.216754001 | 2.35E-19 | 1.29E-17 | SNORA31 |  |
| ENSG00000163001 | -2.778300105 | 2.43E-19 | 1.33E-17 | CFAP36 |  |
| ENSG00000108091 | -1.858710192 | 2.70E-19 | 1.47E-17 | CCDC6 |  |
| ENSG00000113643 | 1.377797909 | 2.74E-19 | 1.49E-17 | RARS |  |
| ENSG00000177700 | 1.244060973 | 2.82E-19 | 1.52E-17 | POLR2L |  |
| ENSG00000108946 | 1.239508402 | 3.47E-19 | 1.87E-17 | PRKAR1A |  |
| ENSG00000199961 | -1.258605755 | 3.48E-19 | 1.87E-17 | SNORD1B |  |
| ENSG00000067533 | -2.241712221 | 3.71E-19 | 1.99E-17 | RRP15 |  |
| ENSG00000113161 | 1.49831381 | 4.64E-19 | 2.47E-17 | HMGCR |  |
| ENSG00000151461 | -1.94018777 | 4.76E-19 | 2.53E-17 | UPF2 |  |
| ENSG00000109881 | -1.93983897 | 5.28E-19 | 2.80E-17 | CCDC34 |  |
| ENSG00000274970 | -5.958131401 | 5.45E-19 | 2.87E-17 | AC013470.4 | |
| ENSG00000134240 | -5.930502343 | 6.30E-19 | 3.31E-17 | HMGCS2 |  |
| ENSG00000030582 | 1.424396518 | 6.60E-19 | 3.45E-17 | GRN |  |
| ENSG00000123453 | -4.619161547 | 6.70E-19 | 3.49E-17 | SARDH |  |
| ENSG00000142166 | 2.51019598 | 8.32E-19 | 4.32E-17 | IFNAR1 |  |
| ENSG00000207947 | -2.824823501 | 8.60E-19 | 4.45E-17 | MIR152 |  |
| ENSG00000084234 | -1.183761964 | 9.87E-19 | 5.09E-17 | APLP2 |  |
| ENSG00000234234 | -5.901946936 | 1.16E-18 | 5.95E-17 | MTCO2P8 |  |
| ENSG00000065809 | -1.636702998 | 1.17E-18 | 6.00E-17 | FAM107B |  |
| ENSG00000145692 | -5.900692253 | 1.20E-18 | 6.12E-17 | BHMT |  |
| ENSG00000072571 | -1.811651347 | 1.29E-18 | 6.57E-17 | HMMR |  |
| ENSG00000165795 | -5.261052583 | 1.37E-18 | 6.96E-17 | NDRG2 |  |
| ENSG00000134250 | 1.221970289 | 1.40E-18 | 7.06E-17 | NOTCH2 |  |
| ENSG00000111640 | 1.332963102 | 1.76E-18 | 8.85E-17 | GAPDH |  |
| ENSG00000197746 | 1.000307307 | 1.97E-18 | 9.90E-17 | PSAP |  |
| ENSG00000201457 | -1.912172696 | 2.27E-18 | 1.13E-16 | SNORA55 |  |
| ENSG00000199179 | -2.631964346 | 2.35E-18 | 1.17E-16 | MIRLET7I |  |
| ENSG00000164587 | -1.078117346 | 2.56E-18 | 1.27E-16 | RPS14 |  |
| ENSG00000052802 | 1.830972703 | 2.58E-18 | 1.27E-16 | MSMO1 |  |
| ENSG00000090273 | -1.12191759 | 2.81E-18 | 1.38E-16 | NUDC |  |
| ENSG00000135919 | 1.294006046 | 3.28E-18 | 1.61E-16 | SERPINE2 |  |
| ENSG00000197345 | -1.90164264 | 3.84E-18 | 1.88E-16 | MRPL21 |  |
| ENSG00000179820 | 1.557909387 | 3.92E-18 | 1.91E-16 | MYADM |  |
| ENSG00000105193 | 1.318432369 | 3.93E-18 | 1.91E-16 | RPS16 |  |
| ENSG00000160208 | -1.537743445 | 3.98E-18 | 1.93E-16 | RRP1B |  |
| ENSG00000031003 | -1.975133007 | 4.96E-18 | 2.40E-16 | FAM13B |  |
| ENSG00000150991 | -1.205556983 | 6.76E-18 | 3.26E-16 | UBC |  |
| ENSG00000131051 | -1.429879376 | 7.03E-18 | 3.37E-16 | RBM39 |  |
| ENSG00000199629 | -3.411140702 | 7.10E-18 | 3.39E-16 | RNU1-14P |  |
| ENSG00000271886 | -2.818253022 | 7.76E-18 | 3.70E-16 | MIR98 |  |
| ENSG00000138071 | 1.308870537 | 7.79E-18 | 3.70E-16 | ACTR2 |  |
| ENSG00000253556 | -5.480649273 | 8.09E-18 | 3.83E-16 | MTCO1P4 |  |
| ENSG00000096696 | -2.143414698 | 8.33E-18 | 3.93E-16 | DSP |  |
| ENSG00000130770 | -1.419062743 | 8.42E-18 | 3.96E-16 | ATP5IF1 |  |
| ENSG00000124713 | -5.435390109 | 8.52E-18 | 4.00E-16 | GNMT |  |
| ENSG00000174437 | 1.064048742 | 1.05E-17 | 4.93E-16 | ATP2A2 |  |
| ENSG00000029725 | -1.7577526 | 1.17E-17 | 5.43E-16 | RABEP1 |  |
| ENSG00000145220 | -2.190949365 | 1.23E-17 | 5.72E-16 | LYAR |  |
| ENSG00000145495 | 1.391262605 | 1.26E-17 | 5.84E-16 | 6-Mar |  |
| ENSG00000167325 | 1.306045334 | 1.27E-17 | 5.88E-16 | RRM1 |  |
| ENSG00000185896 | 1.237682638 | 1.40E-17 | 6.43E-16 | LAMP1 |  |
| ENSG00000274452 | -3.140090371 | 1.40E-17 | 6.43E-16 | RF00004 |  |
| ENSG00000197208 | 2.148584066 | 1.57E-17 | 7.16E-16 | SLC22A4 |  |
| ENSG00000212452 | -1.480232901 | 1.78E-17 | 8.10E-16 | SNORD69 |  |
| ENSG00000062194 | -1.632754167 | 2.11E-17 | 9.58E-16 | GPBP1 |  |
| ENSG00000115091 | 1.334478928 | 2.17E-17 | 9.82E-16 | ACTR3 |  |
| ENSG00000117601 | -5.411648995 | 2.28E-17 | 1.03E-15 | SERPINC1 |  |
| ENSG00000077380 | -1.540523214 | 2.49E-17 | 1.12E-15 | DYNC1I2 |  |
| ENSG00000140367 | -2.71370774 | 2.54E-17 | 1.14E-15 | UBE2Q2 |  |
| ENSG00000152818 | -1.223019367 | 2.57E-17 | 1.15E-15 | UTRN |  |
| ENSG00000139641 | 1.172504596 | 2.76E-17 | 1.23E-15 | ESYT1 |  |
| ENSG00000185803 | 1.986928345 | 2.97E-17 | 1.32E-15 | SLC52A2 |  |
| ENSG00000106052 | -1.758200178 | 3.00E-17 | 1.33E-15 | TAX1BP1 |  |
| ENSG00000132670 | -1.679502955 | 3.26E-17 | 1.44E-15 | PTPRA |  |
| ENSG00000012223 | -5.441985819 | 3.40E-17 | 1.50E-15 | LTF |  |
| ENSG00000104408 | 1.526881732 | 3.50E-17 | 1.54E-15 | EIF3E |  |
| ENSG00000054654 | -1.706219483 | 3.77E-17 | 1.65E-15 | SYNE2 |  |
| ENSG00000084073 | 1.540738669 | 4.51E-17 | 1.97E-15 | ZMPSTE24 |  |
| ENSG00000199051 | -1.679395311 | 4.53E-17 | 1.97E-15 | MIR361 |  |
| ENSG00000196642 | -1.353208346 | 4.74E-17 | 2.06E-15 | RABL6 |  |
| ENSG00000204394 | 1.57073101 | 4.88E-17 | 2.11E-15 | VARS |  |
| ENSG00000170289 | -5.896530685 | 5.25E-17 | 2.26E-15 | CNGB3 |  |
| ENSG00000091986 | -1.645918957 | 5.28E-17 | 2.27E-15 | CCDC80 |  |
| ENSG00000111142 | -1.309101723 | 5.64E-17 | 2.42E-15 | METAP2 |  |
| ENSG00000277157 | 1.233424539 | 5.67E-17 | 2.42E-15 | HIST1H4D |  |
| ENSG00000084652 | -1.288121275 | 7.21E-17 | 3.07E-15 | TXLNA |  |
| ENSG00000134917 | 2.448987257 | 7.92E-17 | 3.37E-15 | ADAMTS8 |  |
| ENSG00000183955 | -1.746695946 | 7.99E-17 | 3.39E-15 | KMT5A |  |
| ENSG00000178252 | 1.745277515 | 8.32E-17 | 3.51E-15 | WDR6 |  |
| ENSG00000171557 | -5.660313432 | 8.53E-17 | 3.59E-15 | FGG |  |
| ENSG00000151693 | -1.629878951 | 8.92E-17 | 3.75E-15 | ASAP2 |  |
| ENSG00000104904 | -1.590909845 | 9.30E-17 | 3.89E-15 | OAZ1 |  |
| ENSG00000256069 | -5.807585231 | 9.84E-17 | 4.11E-15 | A2MP1 |  |
| ENSG00000101464 | 2.230951282 | 1.18E-16 | 4.91E-15 | PIGU |  |
| ENSG00000207585 | -2.121795341 | 1.24E-16 | 5.14E-15 | MIR181D |  |
| ENSG00000172053 | 1.17036106 | 1.53E-16 | 6.32E-15 | QARS |  |
| ENSG00000155100 | -2.365922792 | 1.83E-16 | 7.57E-15 | OTUD6B |  |
| ENSG00000145734 | -1.825542228 | 1.93E-16 | 7.92E-15 | BDP1 |  |
| ENSG00000249784 | -2.948419344 | 1.96E-16 | 8.03E-15 | SCARNA22 |  |
| ENSG00000270672 | -3.656581249 | 2.03E-16 | 8.30E-15 | MTRNR2L6 |  |
| ENSG00000209645 | -2.683535483 | 2.30E-16 | 9.38E-15 | SNORD105 |  |
| ENSG00000207547 | -3.061099552 | 2.39E-16 | 9.72E-15 | MIR25 |  |
| ENSG00000167671 | -1.46008489 | 2.53E-16 | 1.03E-14 | UBXN6 |  |
| ENSG00000202400 | -3.428651789 | 2.58E-16 | 1.05E-14 | SNORD82 |  |
| ENSG00000064601 | 1.551749292 | 2.77E-16 | 1.12E-14 | CTSA |  |
| ENSG00000092853 | -1.622696935 | 3.07E-16 | 1.23E-14 | CLSPN |  |
| ENSG00000207932 | 4.376433718 | 3.42E-16 | 1.37E-14 | MIR33A |  |
| ENSG00000165629 | 1.389647023 | 3.46E-16 | 1.38E-14 | ATP5F1C |  |
| ENSG00000101335 | -1.436978622 | 3.75E-16 | 1.50E-14 | MYL9 |  |
| ENSG00000048649 | -1.46938284 | 3.93E-16 | 1.56E-14 | RSF1 |  |
| ENSG00000136930 | 1.34352399 | 4.03E-16 | 1.60E-14 | PSMB7 |  |
| ENSG00000050405 | -1.164475812 | 4.03E-16 | 1.60E-14 | LIMA1 |  |
| ENSG00000174720 | -2.620503993 | 4.65E-16 | 1.84E-14 | LARP7 |  |
| ENSG00000138207 | -4.232116159 | 4.74E-16 | 1.86E-14 | RBP4 |  |
| ENSG00000080986 | -1.534992355 | 4.98E-16 | 1.95E-14 | NDC80 |  |
| ENSG00000023191 | 1.219494771 | 5.02E-16 | 1.96E-14 | RNH1 |  |
| ENSG00000173905 | -1.5050628 | 5.16E-16 | 2.01E-14 | GOLIM4 |  |
| ENSG00000124177 | -1.751417331 | 5.40E-16 | 2.10E-14 | CHD6 |  |
| ENSG00000147526 | -1.282980655 | 5.72E-16 | 2.22E-14 | TACC1 |  |
| ENSG00000075151 | -1.503365808 | 6.05E-16 | 2.34E-14 | EIF4G3 |  |
| ENSG00000123384 | 1.039334952 | 6.06E-16 | 2.34E-14 | LRP1 |  |
| ENSG00000171759 | -5.527632042 | 6.19E-16 | 2.38E-14 | PAH |  |
| ENSG00000078674 | -1.091577207 | 7.00E-16 | 2.69E-14 | PCM1 |  |
| ENSG00000174243 | -1.328773076 | 7.17E-16 | 2.75E-14 | DDX23 |  |
| ENSG00000167088 | -1.274221691 | 7.40E-16 | 2.83E-14 | SNRPD1 |  |
| ENSG00000171564 | -5.520199858 | 7.83E-16 | 2.99E-14 | FGB |  |
| ENSG00000200211 | 1.758106945 | 7.89E-16 | 3.00E-14 | RNY4P27 |  |
| ENSG00000145321 | -5.641609905 | 9.04E-16 | 3.43E-14 | GC |  |
| ENSG00000263711 | -5.515195603 | 9.93E-16 | 3.75E-14 | AC079062.1 | |
| ENSG00000259905 | -2.506412467 | 1.07E-15 | 4.03E-14 | PWRN1 |  |
| ENSG00000204256 | -1.69237848 | 1.18E-15 | 4.42E-14 | BRD2 |  |
| ENSG00000167670 | -1.475537408 | 1.22E-15 | 4.57E-14 | CHAF1A |  |
| ENSG00000204463 | 1.214360848 | 1.24E-15 | 4.66E-14 | BAG6 |  |
| ENSG00000177733 | 1.236870315 | 1.33E-15 | 4.96E-14 | HNRNPA0 |  |
| ENSG00000108179 | 1.119760758 | 1.36E-15 | 5.07E-14 | PPIF |  |
| ENSG00000183258 | -1.547699364 | 1.54E-15 | 5.72E-14 | DDX41 |  |
| ENSG00000271480 | -5.470852565 | 1.61E-15 | 5.96E-14 | MTND3P19 |  |
| ENSG00000254324 | -1.20658313 | 1.72E-15 | 6.34E-14 | MIR151A |  |
| ENSG00000138600 | 1.465596184 | 1.81E-15 | 6.67E-14 | SPPL2A |  |
| ENSG00000005884 | 0.917845893 | 1.84E-15 | 6.77E-14 | ITGA3 |  |
| ENSG00000105223 | 1.427751397 | 2.13E-15 | 7.80E-14 | PLD3 |  |
| ENSG00000196498 | -1.447233208 | 2.15E-15 | 7.88E-14 | NCOR2 |  |
| ENSG00000163166 | -1.374014137 | 2.20E-15 | 8.03E-14 | IWS1 |  |
| ENSG00000161981 | -1.983254949 | 2.46E-15 | 8.96E-14 | SNRNP25 |  |
| ENSG00000240103 | -5.447980464 | 2.54E-15 | 9.22E-14 | AC132825.1 | |
| ENSG00000137203 | 1.454358835 | 2.58E-15 | 9.36E-14 | TFAP2A |  |
| ENSG00000130726 | 1.143523096 | 2.82E-15 | 1.02E-13 | TRIM28 |  |
| ENSG00000108349 | -1.364685593 | 3.66E-15 | 1.32E-13 | CASC3 |  |
| ENSG00000141858 | -2.283841836 | 3.88E-15 | 1.39E-13 | SAMD1 |  |
| ENSG00000084463 | -1.768301592 | 3.90E-15 | 1.40E-13 | WBP11 |  |
| ENSG00000163527 | 1.198627791 | 4.98E-15 | 1.78E-13 | STT3B |  |
| ENSG00000136936 | -2.402679983 | 5.66E-15 | 2.02E-13 | XPA |  |
| ENSG00000158104 | -5.388970026 | 5.99E-15 | 2.13E-13 | HPD |  |
| ENSG00000140990 | -1.663768056 | 6.50E-15 | 2.31E-13 | NDUFB10 |  |
| ENSG00000200913 | -2.835648964 | 6.85E-15 | 2.43E-13 | SNORD46 |  |
| ENSG00000169554 | -2.161732997 | 6.92E-15 | 2.45E-13 | ZEB2 |  |
| ENSG00000008282 | 1.822172968 | 7.12E-15 | 2.51E-13 | SYPL1 |  |
| ENSG00000123240 | -1.312259528 | 7.63E-15 | 2.68E-13 | OPTN |  |
| ENSG00000202347 | -1.886779905 | 9.00E-15 | 3.15E-13 | RNU1-16P |  |
| ENSG00000167522 | -1.612327263 | 9.03E-15 | 3.16E-13 | ANKRD11 |  |
| ENSG00000115504 | -1.824820588 | 9.36E-15 | 3.26E-13 | EHBP1 |  |
| ENSG00000101773 | -2.176115592 | 9.73E-15 | 3.39E-13 | RBBP8 |  |
| ENSG00000010256 | 1.172573449 | 1.11E-14 | 3.86E-13 | UQCRC1 |  |
| ENSG00000100345 | -0.956930357 | 1.15E-14 | 3.97E-13 | MYH9 |  |
| ENSG00000105402 | 1.793688195 | 1.16E-14 | 4.02E-13 | NAPA |  |
| ENSG00000105401 | -1.240137877 | 1.27E-14 | 4.38E-13 | CDC37 |  |
| ENSG00000005022 | 1.453519185 | 1.28E-14 | 4.41E-13 | SLC25A5 |  |
| ENSG00000071054 | -1.105804227 | 1.36E-14 | 4.66E-13 | MAP4K4 |  |
| ENSG00000078668 | 1.528701268 | 1.37E-14 | 4.69E-13 | VDAC3 |  |
| ENSG00000104218 | -2.21404923 | 1.45E-14 | 4.94E-13 | CSPP1 |  |
| ENSG00000252759 | 1.770050696 | 1.53E-14 | 5.23E-13 | RF00019 |  |
| ENSG00000196787 | 1.133478542 | 1.54E-14 | 5.23E-13 | HIST1H2AG | |
| ENSG00000181090 | -1.658589624 | 1.58E-14 | 5.37E-13 | EHMT1 |  |
| ENSG00000113758 | -1.561105621 | 1.61E-14 | 5.46E-13 | DBN1 |  |
| ENSG00000207201 | -2.507274467 | 1.69E-14 | 5.70E-13 | RNU1-148P | |
| ENSG00000128918 | -5.324463964 | 1.69E-14 | 5.71E-13 | ALDH1A2 |  |
| ENSG00000083896 | -1.568806122 | 1.87E-14 | 6.26E-13 | YTHDC1 |  |
| ENSG00000086061 | -1.122263004 | 1.87E-14 | 6.26E-13 | DNAJA1 |  |
| ENSG00000168495 | -2.029132181 | 1.92E-14 | 6.41E-13 | POLR3D |  |
| ENSG00000144908 | -4.718259316 | 1.96E-14 | 6.54E-13 | ALDH1L1 |  |
| ENSG00000092820 | -0.977131117 | 2.07E-14 | 6.90E-13 | EZR |  |
| ENSG00000125651 | -1.316602341 | 2.13E-14 | 7.08E-13 | GTF2F1 |  |
| ENSG00000264229 | -2.162924787 | 2.32E-14 | 7.70E-13 | RNU4ATAC |  |
| ENSG00000173575 | -1.518143529 | 2.62E-14 | 8.67E-13 | CHD2 |  |
| ENSG00000116521 | 1.767577311 | 2.68E-14 | 8.84E-13 | SCAMP3 |  |
| ENSG00000110108 | 1.481226641 | 2.76E-14 | 9.07E-13 | TMEM109 |  |
| ENSG00000130649 | -5.47591435 | 2.88E-14 | 9.44E-13 | CYP2E1 |  |
| ENSG00000163714 | -1.498754595 | 3.09E-14 | 1.01E-12 | U2SURP |  |
| ENSG00000168036 | 1.071169506 | 3.14E-14 | 1.03E-12 | CTNNB1 |  |
| ENSG00000231034 | -5.270517474 | 3.19E-14 | 1.04E-12 | AL118520.1 | |
| ENSG00000173230 | -1.207570528 | 3.32E-14 | 1.08E-12 | GOLGB1 |  |
| ENSG00000116729 | 1.76632665 | 3.33E-14 | 1.08E-12 | WLS |  |
| ENSG00000145555 | -1.418463759 | 3.70E-14 | 1.20E-12 | MYO10 |  |
| ENSG00000153046 | -1.589082072 | 3.77E-14 | 1.22E-12 | CDYL |  |
| ENSG00000082805 | -1.43153229 | 3.78E-14 | 1.22E-12 | ERC1 |  |
| ENSG00000198087 | -1.539200765 | 3.92E-14 | 1.26E-12 | CD2AP |  |
| ENSG00000134884 | -1.869083862 | 3.97E-14 | 1.28E-12 | ARGLU1 |  |
| ENSG00000090339 | 1.598009721 | 4.25E-14 | 1.36E-12 | ICAM1 |  |
| ENSG00000058262 | 1.240510503 | 4.50E-14 | 1.44E-12 | SEC61A1 |  |
| ENSG00000160818 | -1.805120769 | 4.51E-14 | 1.44E-12 | GPATCH4 |  |
| ENSG00000028310 | -1.538885681 | 4.56E-14 | 1.45E-12 | BRD9 |  |
| ENSG00000159335 | -1.241353585 | 4.79E-14 | 1.52E-12 | PTMS |  |
| ENSG00000100258 | 1.416152094 | 4.88E-14 | 1.54E-12 | LMF2 |  |
| ENSG00000104635 | 1.349521516 | 5.01E-14 | 1.58E-12 | SLC39A14 |  |
| ENSG00000134371 | -1.847550574 | 5.07E-14 | 1.60E-12 | CDC73 |  |
| ENSG00000136560 | -1.92196477 | 5.24E-14 | 1.65E-12 | TANK |  |
| ENSG00000206885 | -2.715932921 | 5.25E-14 | 1.65E-12 | SNORA75 |  |
| ENSG00000074356 | -1.584394568 | 5.29E-14 | 1.66E-12 | NCBP3 |  |
| ENSG00000132824 | 1.281273052 | 5.33E-14 | 1.67E-12 | SERINC3 |  |
| ENSG00000156508 | 0.95668358 | 5.89E-14 | 1.84E-12 | EEF1A1 |  |
| ENSG00000134453 | -1.537802098 | 6.17E-14 | 1.92E-12 | RBM17 |  |
| ENSG00000134308 | 0.913247411 | 6.30E-14 | 1.96E-12 | YWHAQ |  |
| ENSG00000067167 | 1.13748617 | 6.53E-14 | 2.02E-12 | TRAM1 |  |
| ENSG00000185825 | 1.082176967 | 6.78E-14 | 2.10E-12 | BCAP31 |  |
| ENSG00000136436 | -1.183150726 | 6.86E-14 | 2.12E-12 | CALCOCO2 |  |
| ENSG00000204592 | 1.180336865 | 7.21E-14 | 2.22E-12 | HLA-E |  |
| ENSG00000106028 | 1.727180617 | 7.45E-14 | 2.29E-12 | SSBP1 |  |
| ENSG00000085224 | -1.488693121 | 7.74E-14 | 2.38E-12 | ATRX |  |
| ENSG00000116161 | -1.741227502 | 7.80E-14 | 2.39E-12 | CACYBP |  |
| ENSG00000167468 | -1.202104611 | 8.86E-14 | 2.71E-12 | GPX4 |  |
| ENSG00000143228 | -1.415649106 | 9.22E-14 | 2.81E-12 | NUF2 |  |
| ENSG00000156261 | 1.109802458 | 9.30E-14 | 2.83E-12 | CCT8 |  |
| ENSG00000170004 | -1.448174362 | 9.77E-14 | 2.96E-12 | CHD3 |  |
| ENSG00000113645 | -1.441305548 | 9.86E-14 | 2.99E-12 | WWC1 |  |
| ENSG00000170633 | -1.637639646 | 9.97E-14 | 3.01E-12 | RNF34 |  |
| ENSG00000161526 | -1.324854206 | 1.10E-13 | 3.33E-12 | SAP30BP |  |
| ENSG00000099331 | -1.3436999 | 1.21E-13 | 3.63E-12 | MYO9B |  |
| ENSG00000182195 | -1.27771828 | 1.23E-13 | 3.69E-12 | LDOC1 |  |
| ENSG00000134824 | 1.162977963 | 1.27E-13 | 3.81E-12 | FADS2 |  |
| ENSG00000196141 | -1.437140292 | 1.32E-13 | 3.95E-12 | SPATS2L |  |
| ENSG00000164934 | -1.408496851 | 1.35E-13 | 4.02E-12 | DCAF13 |  |
| ENSG00000142798 | 1.123074287 | 1.40E-13 | 4.18E-12 | HSPG2 |  |
| ENSG00000242265 | -1.618158336 | 1.55E-13 | 4.59E-12 | PEG10 |  |
| ENSG00000255298 | -5.169846567 | 1.58E-13 | 4.68E-12 | OR8G5 |  |
| ENSG00000079459 | 1.273605417 | 1.58E-13 | 4.68E-12 | FDFT1 |  |
| ENSG00000122966 | -1.090284427 | 1.63E-13 | 4.81E-12 | CIT |  |
| ENSG00000197714 | 2.180522509 | 1.70E-13 | 5.02E-12 | ZNF460 |  |
| ENSG00000222426 | -4.620115139 | 1.77E-13 | 5.20E-12 | RNU2-50P |  |
| ENSG00000184863 | -1.450159586 | 2.01E-13 | 5.91E-12 | RBM33 |  |
| ENSG00000186480 | 1.55709479 | 2.08E-13 | 6.08E-12 | INSIG1 |  |
| ENSG00000214530 | -3.034279524 | 2.19E-13 | 6.41E-12 | STARD10 |  |
| ENSG00000150316 | -1.555290032 | 2.23E-13 | 6.50E-12 | CWC15 |  |
| ENSG00000105821 | -2.047548002 | 2.28E-13 | 6.64E-12 | DNAJC2 |  |
| ENSG00000164167 | -2.46536392 | 2.66E-13 | 7.72E-12 | LSM6 |  |
| ENSG00000134480 | -1.349779417 | 2.76E-13 | 8.01E-12 | CCNH |  |
| ENSG00000212907 | -1.109437292 | 2.77E-13 | 8.02E-12 | MT-ND4L |  |
| ENSG00000101182 | -0.964628568 | 2.87E-13 | 8.29E-12 | PSMA7 |  |
| ENSG00000231464 | -3.870946743 | 2.88E-13 | 8.31E-12 | AC024937.2 | |
| ENSG00000188229 | 1.137239009 | 2.93E-13 | 8.44E-12 | TUBB4B |  |
| ENSG00000162704 | -0.904467148 | 2.97E-13 | 8.52E-12 | ARPC5 |  |
| ENSG00000108588 | -1.030273854 | 3.08E-13 | 8.83E-12 | CCDC47 |  |
| ENSG00000036473 | -5.112610896 | 3.30E-13 | 9.43E-12 | OTC |  |
| ENSG00000140319 | -1.506673323 | 3.30E-13 | 9.43E-12 | SRP14 |  |
| ENSG00000106853 | 1.550776223 | 3.35E-13 | 9.54E-12 | PTGR1 |  |
| ENSG00000123737 | -1.214352156 | 3.42E-13 | 9.73E-12 | EXOSC9 |  |
| ENSG00000155380 | 1.392507789 | 3.45E-13 | 9.80E-12 | SLC16A1 |  |
| ENSG00000122873 | 2.025754722 | 3.57E-13 | 1.01E-11 | CISD1 |  |
| ENSG00000071967 | 1.780005228 | 4.59E-13 | 1.30E-11 | CYBRD1 |  |
| ENSG00000073578 | 1.18880936 | 4.60E-13 | 1.30E-11 | SDHA |  |
| ENSG00000204628 | 0.848977706 | 4.78E-13 | 1.35E-11 | RACK1 |  |
| ENSG00000111450 | -1.571429398 | 4.85E-13 | 1.36E-11 | STX2 |  |
| ENSG00000108953 | -0.951648169 | 5.16E-13 | 1.45E-11 | YWHAE |  |
| ENSG00000150768 | 2.039735326 | 5.45E-13 | 1.53E-11 | DLAT |  |
| ENSG00000252635 | -4.838122624 | 5.50E-13 | 1.54E-11 | RNU2-56P |  |
| ENSG00000119397 | -2.219280286 | 5.75E-13 | 1.60E-11 | CNTRL |  |
| ENSG00000138095 | 1.075778721 | 6.39E-13 | 1.78E-11 | LRPPRC |  |
| ENSG00000167447 | 2.024229947 | 8.13E-13 | 2.26E-11 | SMG8 |  |
| ENSG00000135521 | -1.911444792 | 8.85E-13 | 2.45E-11 | LTV1 |  |
| ENSG00000065534 | -1.48983441 | 9.92E-13 | 2.75E-11 | MYLK |  |
| ENSG00000100281 | -1.4924299 | 1.02E-12 | 2.82E-11 | HMGXB4 |  |
| ENSG00000101057 | -1.100118581 | 1.03E-12 | 2.83E-11 | MYBL2 |  |
| ENSG00000005339 | -1.279873538 | 1.05E-12 | 2.89E-11 | CREBBP |  |
| ENSG00000160211 | 1.017626284 | 1.17E-12 | 3.22E-11 | G6PD |  |
| ENSG00000198604 | -1.665760581 | 1.30E-12 | 3.55E-11 | BAZ1A |  |
| ENSG00000239995 | -5.011377931 | 1.31E-12 | 3.60E-11 | TPT1P11 |  |
| ENSG00000249262 | -5.253510121 | 1.38E-12 | 3.76E-11 | AC112250.1 | |
| ENSG00000162614 | -2.735869194 | 1.44E-12 | 3.91E-11 | NEXN |  |
| ENSG00000162892 | 3.06966549 | 1.51E-12 | 4.10E-11 | IL24 |  |
| ENSG00000084754 | 1.253334145 | 1.60E-12 | 4.35E-11 | HADHA |  |
| ENSG00000116260 | 1.014244275 | 1.66E-12 | 4.49E-11 | QSOX1 |  |
| ENSG00000256045 | -4.995289244 | 1.69E-12 | 4.57E-11 | MTRNR2L10 | |
| ENSG00000135046 | -1.056921249 | 1.71E-12 | 4.63E-11 | ANXA1 |  |
| ENSG00000178105 | -1.642283228 | 1.74E-12 | 4.68E-11 | DDX10 |  |
| ENSG00000078618 | -1.153931535 | 1.80E-12 | 4.84E-11 | NRDC |  |
| ENSG00000126001 | -1.228091687 | 1.87E-12 | 5.01E-11 | CEP250 |  |
| ENSG00000167986 | 1.035916117 | 1.87E-12 | 5.02E-11 | DDB1 |  |
| ENSG00000201910 | -2.898806916 | 1.94E-12 | 5.19E-11 | RNU1-140P | |
| ENSG00000148834 | 1.43249875 | 1.99E-12 | 5.30E-11 | GSTO1 |  |
| ENSG00000272333 | -2.048819022 | 2.06E-12 | 5.50E-11 | KMT2B |  |
| ENSG00000106771 | 1.1413576 | 2.11E-12 | 5.62E-11 | TMEM245 |  |
| ENSG00000136240 | 1.110809458 | 2.12E-12 | 5.62E-11 | KDELR2 |  |
| ENSG00000132424 | -1.446260472 | 2.19E-12 | 5.80E-11 | PNISR |  |
| ENSG00000159377 | 1.018520889 | 2.26E-12 | 5.98E-11 | PSMB4 |  |
| ENSG00000065548 | -1.643313199 | 2.37E-12 | 6.27E-11 | ZC3H15 |  |
| ENSG00000131626 | -1.51998229 | 2.38E-12 | 6.27E-11 | PPFIA1 |  |
| ENSG00000075415 | 1.057259404 | 2.42E-12 | 6.38E-11 | SLC25A3 |  |
| ENSG00000160551 | -1.258480095 | 2.68E-12 | 7.04E-11 | TAOK1 |  |
| ENSG00000207970 | -2.856591897 | 3.05E-12 | 7.99E-11 | MIR660 |  |
| ENSG00000101350 | -1.418037756 | 3.34E-12 | 8.73E-11 | KIF3B |  |
| ENSG00000196235 | -1.035305166 | 3.37E-12 | 8.81E-11 | SUPT5H |  |
| ENSG00000015171 | -1.448233165 | 3.58E-12 | 9.33E-11 | ZMYND11 |  |
| ENSG00000208036 | -1.549238136 | 3.67E-12 | 9.54E-11 | MIR106B |  |
| ENSG00000159131 | 1.4768765 | 3.71E-12 | 9.63E-11 | GART |  |
| ENSG00000122257 | -1.501286302 | 3.80E-12 | 9.86E-11 | RBBP6 |  |
| ENSG00000080503 | -1.241615625 | 3.88E-12 | 1.01E-10 | SMARCA2 |  |
| ENSG00000148248 | 1.021405016 | 3.90E-12 | 1.01E-10 | SURF4 |  |
| ENSG00000227195 | -1.358729843 | 4.15E-12 | 1.07E-10 | MIR663AHG | |
| ENSG00000126457 | -0.964316021 | 4.35E-12 | 1.12E-10 | PRMT1 |  |
| ENSG00000047315 | 1.21379567 | 4.54E-12 | 1.17E-10 | POLR2B |  |
| ENSG00000115216 | -1.010897697 | 4.55E-12 | 1.17E-10 | NRBP1 |  |
| ENSG00000145860 | 1.163775756 | 4.86E-12 | 1.24E-10 | RNF145 |  |
| ENSG00000101294 | 1.066094573 | 5.03E-12 | 1.29E-10 | HM13 |  |
| ENSG00000150779 | 1.787486576 | 5.37E-12 | 1.37E-10 | TIMM8B |  |
| ENSG00000125844 | -1.022370177 | 5.66E-12 | 1.44E-10 | RRBP1 |  |
| ENSG00000238531 | -2.338572514 | 5.81E-12 | 1.48E-10 | SNORD105B | |
| ENSG00000266019 | -2.048106715 | 5.84E-12 | 1.48E-10 | MIR3609 |  |
| ENSG00000182544 | 2.121918371 | 6.07E-12 | 1.54E-10 | MFSD5 |  |
| ENSG00000189060 | -1.192909873 | 6.29E-12 | 1.59E-10 | H1F0 |  |
| ENSG00000093167 | -1.495780718 | 6.61E-12 | 1.67E-10 | LRRFIP2 |  |
| ENSG00000213699 | 1.969622541 | 7.32E-12 | 1.84E-10 | SLC35F6 |  |
| ENSG00000065154 | 1.482557909 | 7.41E-12 | 1.87E-10 | OAT |  |
| ENSG00000153187 | -0.79562298 | 7.54E-12 | 1.90E-10 | HNRNPU |  |
| ENSG00000198301 | -1.743329819 | 7.83E-12 | 1.96E-10 | SDAD1 |  |
| ENSG00000108679 | 0.920377961 | 7.93E-12 | 1.99E-10 | LGALS3BP |  |
| ENSG00000151655 | -4.609139573 | 8.87E-12 | 2.22E-10 | ITIH2 |  |
| ENSG00000178719 | 1.490965876 | 9.32E-12 | 2.33E-10 | GRINA |  |
| ENSG00000165092 | -4.581946383 | 9.47E-12 | 2.36E-10 | ALDH1A1 |  |
| ENSG00000105220 | 0.904387633 | 9.54E-12 | 2.37E-10 | GPI |  |
| ENSG00000077147 | 1.113257065 | 1.09E-11 | 2.70E-10 | TM9SF3 |  |
| ENSG00000112118 | -0.881199377 | 1.09E-11 | 2.70E-10 | MCM3 |  |
| ENSG00000100266 | -1.001685115 | 1.10E-11 | 2.72E-10 | PACSIN2 |  |
| ENSG00000147140 | -1.039280904 | 1.14E-11 | 2.81E-10 | NONO |  |
| ENSG00000159720 | 1.085578419 | 1.18E-11 | 2.90E-10 | ATP6V0D1 |  |
| ENSG00000084774 | 1.182595619 | 1.28E-11 | 3.16E-10 | CAD |  |
| ENSG00000113649 | -1.086914647 | 1.30E-11 | 3.20E-10 | TCERG1 |  |
| ENSG00000172893 | 1.419710737 | 1.31E-11 | 3.22E-10 | DHCR7 |  |
| ENSG00000110955 | 0.823381389 | 1.34E-11 | 3.28E-10 | ATP5F1B |  |
| ENSG00000145901 | -1.104041895 | 1.34E-11 | 3.28E-10 | TNIP1 |  |
| ENSG00000125398 | -2.40817072 | 1.46E-11 | 3.57E-10 | SOX9 |  |
| ENSG00000118007 | -1.404290161 | 1.49E-11 | 3.62E-10 | STAG1 |  |
| ENSG00000133026 | -1.275958784 | 1.55E-11 | 3.76E-10 | MYH10 |  |
| ENSG00000202566 | -2.976542686 | 1.58E-11 | 3.84E-10 | MIR421 |  |
| ENSG00000155657 | -0.962311981 | 1.66E-11 | 4.03E-10 | TTN |  |
| ENSG00000065000 | -1.036568597 | 1.69E-11 | 4.09E-10 | AP3D1 |  |
| ENSG00000104611 | -2.134663344 | 1.80E-11 | 4.34E-10 | SH2D4A |  |
| ENSG00000152234 | 0.846493971 | 1.80E-11 | 4.35E-10 | ATP5F1A |  |
| ENSG00000158874 | -3.933838038 | 1.86E-11 | 4.47E-10 | APOA2 |  |
| ENSG00000135837 | -1.449811452 | 1.93E-11 | 4.65E-10 | CEP350 |  |
| ENSG00000278099 | -1.810626158 | 1.94E-11 | 4.66E-10 | RF00003 |  |
| ENSG00000208012 | -2.211588625 | 1.99E-11 | 4.77E-10 | MIRLET7F2 | |
| ENSG00000206754 | -1.184846135 | 2.14E-11 | 5.12E-10 | SNORD101 |  |
| ENSG00000214160 | 2.057392351 | 2.16E-11 | 5.16E-10 | ALG3 |  |
| ENSG00000152700 | 1.408063238 | 2.17E-11 | 5.17E-10 | SAR1B |  |
| ENSG00000101040 | -1.541072508 | 2.27E-11 | 5.40E-10 | ZMYND8 |  |
| ENSG00000131747 | -0.957367214 | 2.28E-11 | 5.42E-10 | TOP2A |  |
| ENSG00000104979 | -1.275176367 | 2.45E-11 | 5.81E-10 | C19orf53 |  |
| ENSG00000125450 | 1.256684292 | 2.48E-11 | 5.87E-10 | NUP85 |  |
| ENSG00000066455 | -1.694424548 | 2.56E-11 | 6.06E-10 | GOLGA5 |  |
| ENSG00000113732 | 1.569328134 | 2.62E-11 | 6.18E-10 | ATP6V0E1 |  |
| ENSG00000197858 | 1.566681914 | 2.70E-11 | 6.37E-10 | GPAA1 |  |
| ENSG00000136810 | 0.994733168 | 2.81E-11 | 6.62E-10 | TXN |  |
| ENSG00000147065 | -0.861159673 | 2.86E-11 | 6.72E-10 | MSN |  |
| ENSG00000119335 | -1.000376076 | 3.08E-11 | 7.21E-10 | SET |  |
| ENSG00000125995 | 1.514130312 | 3.26E-11 | 7.64E-10 | ROMO1 |  |
| ENSG00000071553 | 1.152354447 | 3.36E-11 | 7.86E-10 | ATP6AP1 |  |
| ENSG00000085662 | 1.140202847 | 3.42E-11 | 7.99E-10 | AKR1B1 |  |
| ENSG00000199059 | -3.715593435 | 3.44E-11 | 8.02E-10 | MIR135B |  |
| ENSG00000167863 | -1.061964502 | 3.74E-11 | 8.71E-10 | ATP5PD |  |
| ENSG00000134318 | -1.133774376 | 3.85E-11 | 8.94E-10 | ROCK2 |  |
| ENSG00000270268 | -4.767664044 | 4.01E-11 | 9.31E-10 | AL009028.1 | |
| ENSG00000148143 | -1.504004596 | 4.17E-11 | 9.65E-10 | ZNF462 |  |
| ENSG00000208037 | -2.002903215 | 4.31E-11 | 9.96E-10 | MIR320A |  |
| ENSG00000128908 | -1.754814238 | 4.42E-11 | 1.02E-09 | INO80 |  |
| ENSG00000165672 | 1.09119896 | 4.65E-11 | 1.07E-09 | PRDX3 |  |
| ENSG00000118271 | -4.243114858 | 4.69E-11 | 1.08E-09 | TTR |  |
| ENSG00000125505 | 1.298246916 | 4.96E-11 | 1.14E-09 | MBOAT7 |  |
| ENSG00000204574 | -1.194947214 | 5.34E-11 | 1.22E-09 | ABCF1 |  |
| ENSG00000124486 | 0.909696437 | 5.54E-11 | 1.27E-09 | USP9X |  |
| ENSG00000134874 | -1.740668517 | 5.64E-11 | 1.29E-09 | DZIP1 |  |
| ENSG00000254759 | -2.14462204 | 5.68E-11 | 1.30E-09 | NAP1L1P1 |  |
| ENSG00000125691 | 1.06521851 | 5.83E-11 | 1.33E-09 | RPL23 |  |
| ENSG00000074696 | 1.241821893 | 6.12E-11 | 1.39E-09 | HACD3 |  |
| ENSG00000038219 | -1.002807994 | 6.46E-11 | 1.47E-09 | BOD1L1 |  |
| ENSG00000166710 | 0.951457533 | 6.62E-11 | 1.50E-09 | B2M |  |
| ENSG00000159063 | 1.742072453 | 6.91E-11 | 1.57E-09 | ALG8 |  |
| ENSG00000110696 | -1.450187156 | 6.93E-11 | 1.57E-09 | C11orf58 |  |
| ENSG00000145439 | 2.501175984 | 6.96E-11 | 1.57E-09 | CBR4 |  |
| ENSG00000140350 | -1.201971072 | 6.97E-11 | 1.57E-09 | ANP32A |  |
| ENSG00000104549 | 1.516780811 | 7.09E-11 | 1.59E-09 | SQLE |  |
| ENSG00000087087 | -1.07440304 | 7.23E-11 | 1.62E-09 | SRRT |  |
| ENSG00000034510 | -0.790930644 | 7.35E-11 | 1.65E-09 | TMSB10 |  |
| ENSG00000263001 | -1.506296424 | 7.53E-11 | 1.69E-09 | GTF2I |  |
| ENSG00000160293 | -1.576288158 | 7.65E-11 | 1.71E-09 | VAV2 |  |
| ENSG00000167565 | 2.224289655 | 7.75E-11 | 1.73E-09 | SERTAD3 |  |
| ENSG00000106462 | -1.538269541 | 7.95E-11 | 1.77E-09 | EZH2 |  |
| ENSG00000168386 | -2.098536907 | 8.38E-11 | 1.87E-09 | FILIP1L |  |
| ENSG00000153147 | -1.056151104 | 8.48E-11 | 1.88E-09 | SMARCA5 |  |
| ENSG00000226953 | -4.691313919 | 9.00E-11 | 2.00E-09 | AC010890.1 | |
| ENSG00000230654 | -4.693719374 | 9.01E-11 | 2.00E-09 | MTCO2P25 |  |
| ENSG00000162385 | -1.66067142 | 9.69E-11 | 2.14E-09 | MAGOH |  |
| ENSG00000222659 | -2.897233722 | 9.72E-11 | 2.15E-09 | RNU2-8P |  |
| ENSG00000125733 | -1.142770214 | 9.75E-11 | 2.15E-09 | TRIP10 |  |
| ENSG00000273559 | -2.111743318 | 9.76E-11 | 2.15E-09 | CWC25 |  |
| ENSG00000047849 | 0.948937217 | 9.80E-11 | 2.15E-09 | MAP4 |  |
| ENSG00000153487 | -2.201465173 | 9.81E-11 | 2.15E-09 | ING1 |  |
| ENSG00000139324 | 1.604550511 | 9.82E-11 | 2.15E-09 | TMTC3 |  |
| ENSG00000092969 | -1.617425873 | 1.02E-10 | 2.24E-09 | TGFB2 |  |
| ENSG00000174891 | -1.458858433 | 1.06E-10 | 2.31E-09 | RSRC1 |  |
| ENSG00000179409 | 1.627957906 | 1.07E-10 | 2.33E-09 | GEMIN4 |  |
| ENSG00000149131 | -4.681272453 | 1.11E-10 | 2.41E-09 | SERPING1 |  |
| ENSG00000154781 | -1.80620121 | 1.16E-10 | 2.53E-09 | CCDC174 |  |
| ENSG00000084093 | -1.371426726 | 1.24E-10 | 2.70E-09 | REST |  |
| ENSG00000121851 | -2.664157388 | 1.26E-10 | 2.72E-09 | POLR3GL |  |
| ENSG00000132837 | -4.649195556 | 1.30E-10 | 2.82E-09 | DMGDH |  |
| ENSG00000170759 | -0.855265782 | 1.31E-10 | 2.82E-09 | KIF5B |  |
| ENSG00000186834 | -1.105257784 | 1.31E-10 | 2.82E-09 | HEXIM1 |  |
| ENSG00000102265 | 1.335625834 | 1.31E-10 | 2.83E-09 | TIMP1 |  |
| ENSG00000212609 | -2.415696964 | 1.32E-10 | 2.84E-09 | RNU1-139P | |
| ENSG00000188846 | -1.158442702 | 1.36E-10 | 2.92E-09 | RPL14 |  |
| ENSG00000064961 | -1.560330875 | 1.41E-10 | 3.02E-09 | HMG20B |  |
| ENSG00000196405 | -2.928286608 | 1.41E-10 | 3.02E-09 | EVL |  |
| ENSG00000135624 | 1.027359507 | 1.47E-10 | 3.15E-09 | CCT7 |  |
| ENSG00000153707 | -4.137380066 | 1.51E-10 | 3.23E-09 | PTPRD |  |
| ENSG00000107672 | -1.615819039 | 1.55E-10 | 3.31E-09 | NSMCE4A |  |
| ENSG00000143970 | -1.388772143 | 1.61E-10 | 3.43E-09 | ASXL2 |  |
| ENSG00000198995 | -1.309202542 | 1.64E-10 | 3.49E-09 | MIR340 |  |
| ENSG00000065978 | -1.137507104 | 1.65E-10 | 3.52E-09 | YBX1 |  |
| ENSG00000139514 | 1.176492717 | 1.68E-10 | 3.55E-09 | SLC7A1 |  |
| ENSG00000163659 | 1.480145484 | 1.70E-10 | 3.59E-09 | TIPARP |  |
| ENSG00000168496 | 1.282063161 | 1.70E-10 | 3.59E-09 | FEN1 |  |
| ENSG00000010278 | 1.032700153 | 1.72E-10 | 3.64E-09 | CD9 |  |
| ENSG00000186566 | -1.250749875 | 1.80E-10 | 3.79E-09 | GPATCH8 |  |
| ENSG00000171951 | -3.183017213 | 1.80E-10 | 3.79E-09 | SCG2 |  |
| ENSG00000138829 | 0.856351257 | 1.89E-10 | 3.97E-09 | FBN2 |  |
| ENSG00000130749 | -1.461662554 | 1.94E-10 | 4.07E-09 | ZC3H4 |  |
| ENSG00000155660 | -0.868596835 | 1.95E-10 | 4.09E-09 | PDIA4 |  |
| ENSG00000124207 | 0.932639867 | 1.95E-10 | 4.09E-09 | CSE1L |  |
| ENSG00000149761 | 1.163786895 | 2.02E-10 | 4.23E-09 | NUDT22 |  |
| ENSG00000105974 | -0.943632599 | 2.08E-10 | 4.34E-09 | CAV1 |  |
| ENSG00000275538 | 2.412310085 | 2.10E-10 | 4.39E-09 | RNVU1-19 |  |
| ENSG00000155463 | 1.508975032 | 2.14E-10 | 4.44E-09 | OXA1L |  |
| ENSG00000198242 | -1.177451304 | 2.14E-10 | 4.45E-09 | RPL23A |  |
| ENSG00000135090 | -1.819344481 | 2.34E-10 | 4.85E-09 | TAOK3 |  |
| ENSG00000207870 | -1.631044461 | 2.45E-10 | 5.07E-09 | MIR221 |  |
| ENSG00000137100 | 1.33570399 | 2.46E-10 | 5.10E-09 | DCTN3 |  |
| ENSG00000101290 | 1.171629419 | 2.60E-10 | 5.38E-09 | CDS2 |  |
| ENSG00000160789 | 0.801568988 | 2.74E-10 | 5.65E-09 | LMNA |  |
| ENSG00000204120 | -1.163501404 | 2.81E-10 | 5.79E-09 | GIGYF2 |  |
| ENSG00000118520 | -4.315494075 | 2.82E-10 | 5.80E-09 | ARG1 |  |
| ENSG00000106105 | 1.099528051 | 2.85E-10 | 5.86E-09 | GARS |  |
| ENSG00000257907 | -3.577535084 | 2.88E-10 | 5.92E-09 | EEF1A1P17 | |
| ENSG00000135486 | 1.004033343 | 3.05E-10 | 6.24E-09 | HNRNPA1 |  |
| ENSG00000237466 | -4.584221158 | 3.05E-10 | 6.24E-09 | MTCO3P41 |  |
| ENSG00000029993 | -1.105483924 | 3.12E-10 | 6.38E-09 | HMGB3 |  |
| ENSG00000138778 | -1.075836224 | 3.21E-10 | 6.55E-09 | CENPE |  |
| ENSG00000136235 | 1.566969742 | 3.24E-10 | 6.60E-09 | GPNMB |  |
| ENSG00000212447 | -1.918642219 | 3.25E-10 | 6.60E-09 | SNORD90 |  |
| ENSG00000155313 | -1.684020698 | 3.28E-10 | 6.67E-09 | USP25 |  |
| ENSG00000207340 | -2.352826058 | 3.38E-10 | 6.86E-09 | RNVU1-1 |  |
| ENSG00000207313 | -2.156012126 | 3.44E-10 | 6.97E-09 | SNORA2B |  |
| ENSG00000124201 | -0.989838689 | 3.44E-10 | 6.97E-09 | ZNFX1 |  |
| ENSG00000160014 | -1.067569158 | 3.53E-10 | 7.12E-09 | CALM3 |  |
| ENSG00000103363 | 1.067530975 | 3.53E-10 | 7.12E-09 | ELOB |  |
| ENSG00000163586 | -4.570045955 | 3.59E-10 | 7.23E-09 | FABP1 |  |
| ENSG00000163870 | 1.701124191 | 3.75E-10 | 7.55E-09 | TPRA1 |  |
| ENSG00000145741 | 0.793153559 | 3.76E-10 | 7.56E-09 | BTF3 |  |
| ENSG00000100300 | 1.482877239 | 3.78E-10 | 7.58E-09 | TSPO |  |
| ENSG00000130956 | -1.962814609 | 3.79E-10 | 7.59E-09 | HABP4 |  |
| ENSG00000212378 | -3.88841983 | 3.79E-10 | 7.59E-09 | RF00592 |  |
| ENSG00000142534 | -0.716956062 | 3.94E-10 | 7.88E-09 | RPS11 |  |
| ENSG00000128283 | 1.826623226 | 4.03E-10 | 8.04E-09 | CDC42EP1 |  |
| ENSG00000099381 | -1.533213452 | 4.07E-10 | 8.11E-09 | SETD1A |  |
| ENSG00000063660 | 1.292799786 | 4.17E-10 | 8.30E-09 | GPC1 |  |
| ENSG00000207595 | -2.604138841 | 4.37E-10 | 8.68E-09 | MIR181A2 |  |
| ENSG00000174938 | 1.653265426 | 4.45E-10 | 8.82E-09 | SEZ6L2 |  |
| ENSG00000240520 | -4.335738975 | 4.65E-10 | 9.22E-09 | UOX |  |
| ENSG00000134333 | 0.816261414 | 4.72E-10 | 9.34E-09 | LDHA |  |
| ENSG00000119242 | -2.211323284 | 4.76E-10 | 9.40E-09 | CCDC92 |  |
| ENSG00000133030 | -1.019191704 | 4.79E-10 | 9.46E-09 | MPRIP |  |
| ENSG00000061936 | -1.375939462 | 4.82E-10 | 9.50E-09 | SFSWAP |  |
| ENSG00000136153 | -1.927642358 | 5.26E-10 | 1.04E-08 | LMO7 |  |
| ENSG00000282815 | -3.078736769 | 5.33E-10 | 1.05E-08 | TEX13C |  |
| ENSG00000105048 | -2.050051308 | 5.38E-10 | 1.06E-08 | TNNT1 |  |
| ENSG00000066322 | 1.353868498 | 5.43E-10 | 1.06E-08 | ELOVL1 |  |
| ENSG00000127616 | -0.894041814 | 5.85E-10 | 1.14E-08 | SMARCA4 |  |
| ENSG00000101076 | -4.525319428 | 5.94E-10 | 1.16E-08 | HNF4A |  |
| ENSG00000200231 | -4.007384225 | 6.10E-10 | 1.19E-08 | RNU1-23P |  |
| ENSG00000199165 | 3.020836542 | 6.17E-10 | 1.20E-08 | MIRLET7A1 | |
| ENSG00000158406 | 0.867721105 | 6.45E-10 | 1.26E-08 | HIST1H4H |  |
| ENSG00000137710 | -1.287389396 | 7.25E-10 | 1.41E-08 | RDX |  |
| ENSG00000272398 | 0.915799736 | 7.62E-10 | 1.48E-08 | CD24 |  |
| ENSG00000187957 | 1.216607766 | 7.84E-10 | 1.52E-08 | DNER |  |
| ENSG00000171241 | 1.161070943 | 7.90E-10 | 1.53E-08 | SHCBP1 |  |
| ENSG00000163808 | -1.315850437 | 7.92E-10 | 1.53E-08 | KIF15 |  |
| ENSG00000105568 | 0.975780775 | 8.31E-10 | 1.61E-08 | PPP2R1A |  |
| ENSG00000284256 | -4.515567131 | 8.45E-10 | 1.63E-08 | MIR5004 |  |
| ENSG00000165525 | -1.846724328 | 8.51E-10 | 1.64E-08 | NEMF |  |
| ENSG00000095319 | 1.098367376 | 8.52E-10 | 1.64E-08 | NUP188 |  |
| ENSG00000142192 | -0.818806766 | 8.65E-10 | 1.66E-08 | APP |  |
| ENSG00000019186 | -4.227398442 | 8.68E-10 | 1.67E-08 | CYP24A1 |  |
| ENSG00000110169 | -3.521030697 | 8.84E-10 | 1.69E-08 | HPX |  |
| ENSG00000199030 | -2.027136436 | 9.32E-10 | 1.78E-08 | MIRLET7C |  |
| ENSG00000207349 | -1.947804879 | 9.64E-10 | 1.84E-08 | RNVU1-17 |  |
| ENSG00000265802 | -2.384232939 | 9.81E-10 | 1.87E-08 | RN7SL49P |  |
| ENSG00000181555 | -1.249466557 | 1.00E-09 | 1.91E-08 | SETD2 |  |
| ENSG00000136986 | 1.542454318 | 1.04E-09 | 1.98E-08 | DERL1 |  |
| ENSG00000035928 | -1.375010907 | 1.06E-09 | 2.01E-08 | RFC1 |  |
| ENSG00000060138 | -1.12594445 | 1.09E-09 | 2.06E-08 | YBX3 |  |
| ENSG00000123349 | -1.051400101 | 1.14E-09 | 2.17E-08 | PFDN5 |  |
| ENSG00000111530 | 1.053619661 | 1.18E-09 | 2.24E-08 | CAND1 |  |
| ENSG00000160326 | 2.033808639 | 1.19E-09 | 2.25E-08 | SLC2A6 |  |
| ENSG00000058668 | 1.080811073 | 1.26E-09 | 2.39E-08 | ATP2B4 |  |
| ENSG00000147010 | -1.565754935 | 1.28E-09 | 2.42E-08 | SH3KBP1 |  |
| ENSG00000130707 | -3.513827465 | 1.29E-09 | 2.42E-08 | ASS1 |  |
| ENSG00000143819 | 1.647074334 | 1.30E-09 | 2.45E-08 | EPHX1 |  |
| ENSG00000136997 | -1.255640596 | 1.34E-09 | 2.52E-08 | MYC |  |
| ENSG00000131781 | -4.447617031 | 1.39E-09 | 2.60E-08 | FMO5 |  |
| ENSG00000108828 | 1.035384631 | 1.39E-09 | 2.60E-08 | VAT1 |  |
| ENSG00000050130 | 1.750693603 | 1.41E-09 | 2.63E-08 | JKAMP |  |
| ENSG00000167900 | 0.979378308 | 1.43E-09 | 2.67E-08 | TK1 |  |
| ENSG00000054267 | -1.617658833 | 1.43E-09 | 2.67E-08 | ARID4B |  |
| ENSG00000144959 | 1.184334687 | 1.43E-09 | 2.67E-08 | NCEH1 |  |
| ENSG00000102030 | -1.74172139 | 1.44E-09 | 2.69E-08 | NAA10 |  |
| ENSG00000151718 | -1.137814616 | 1.45E-09 | 2.69E-08 | WWC2 |  |
| ENSG00000004534 | -1.321478862 | 1.45E-09 | 2.69E-08 | RBM6 |  |
| ENSG00000129562 | 1.34527526 | 1.49E-09 | 2.76E-08 | DAD1 |  |
| ENSG00000163605 | -1.265716598 | 1.49E-09 | 2.77E-08 | PPP4R2 |  |
| ENSG00000180992 | -1.536672889 | 1.58E-09 | 2.92E-08 | MRPL14 |  |
| ENSG00000156471 | 1.123259077 | 1.60E-09 | 2.95E-08 | PTDSS1 |  |
| ENSG00000089737 | -0.98689681 | 1.62E-09 | 3.00E-08 | DDX24 |  |
| ENSG00000212232 | -1.455364061 | 1.64E-09 | 3.01E-08 | SNORD17 |  |
| ENSG00000222365 | -1.236454393 | 1.70E-09 | 3.12E-08 | SNORD12B |  |
| ENSG00000105355 | 1.109501282 | 1.75E-09 | 3.22E-08 | PLIN3 |  |
| ENSG00000222624 | -3.934057576 | 1.76E-09 | 3.22E-08 | RNU2-15P |  |
| ENSG00000103249 | 1.41853821 | 1.80E-09 | 3.29E-08 | CLCN7 |  |
| ENSG00000249264 | 2.925051695 | 1.86E-09 | 3.41E-08 | EEF1A1P9 |  |
| ENSG00000163029 | -1.315328629 | 1.91E-09 | 3.49E-08 | SMC6 |  |
| ENSG00000209702 | -2.136002474 | 1.92E-09 | 3.51E-08 | SNORD41 |  |
| ENSG00000112701 | -1.312315909 | 1.97E-09 | 3.59E-08 | SENP6 |  |
| ENSG00000067704 | 1.270298562 | 2.03E-09 | 3.69E-08 | IARS2 |  |
| ENSG00000104067 | -1.133286812 | 2.04E-09 | 3.70E-08 | TJP1 |  |
| ENSG00000049860 | 1.108759424 | 2.04E-09 | 3.70E-08 | HEXB |  |
| ENSG00000155561 | 1.051858908 | 2.07E-09 | 3.76E-08 | NUP205 |  |
| ENSG00000196914 | -0.990669266 | 2.08E-09 | 3.77E-08 | ARHGEF12 |  |
| ENSG00000123989 | 1.776463531 | 2.12E-09 | 3.83E-08 | CHPF |  |
| ENSG00000223224 | -2.258060447 | 2.14E-09 | 3.88E-08 | SNORD71 |  |
| ENSG00000120437 | 1.120669461 | 2.17E-09 | 3.91E-08 | ACAT2 |  |
| ENSG00000082515 | 2.005554192 | 2.19E-09 | 3.96E-08 | MRPL22 |  |
| ENSG00000198752 | -0.989475472 | 2.28E-09 | 4.10E-08 | CDC42BPB |  |
| ENSG00000115816 | -1.35334178 | 2.30E-09 | 4.13E-08 | CEBPZ |  |
| ENSG00000067057 | 0.779663975 | 2.38E-09 | 4.27E-08 | PFKP |  |
| ENSG00000173546 | 1.262556799 | 2.43E-09 | 4.36E-08 | CSPG4 |  |
| ENSG00000199038 | -2.224354201 | 2.44E-09 | 4.37E-08 | MIR210 |  |
| ENSG00000164879 | -4.170160461 | 2.52E-09 | 4.50E-08 | CA3 |  |
| ENSG00000164754 | -0.859830424 | 2.67E-09 | 4.77E-08 | RAD21 |  |
| ENSG00000108691 | 1.491488222 | 2.92E-09 | 5.22E-08 | CCL2 |  |
| ENSG00000141580 | -1.243503405 | 3.11E-09 | 5.55E-08 | WDR45B |  |
| ENSG00000148229 | -1.354372075 | 3.13E-09 | 5.58E-08 | POLE3 |  |
| ENSG00000234545 | -2.856969429 | 3.20E-09 | 5.70E-08 | FAM133B |  |
| ENSG00000174799 | -1.854189407 | 3.36E-09 | 5.97E-08 | CEP135 |  |
| ENSG00000207562 | -1.820603567 | 3.37E-09 | 5.98E-08 | MIR34C |  |
| ENSG00000140612 | 1.277381526 | 3.58E-09 | 6.35E-08 | SEC11A |  |
| ENSG00000225091 | -3.656451519 | 3.62E-09 | 6.40E-08 | SNORA71A |  |
| ENSG00000284726 | 1.77115263 | 3.64E-09 | 6.43E-08 | AL109936.6 | |
| ENSG00000162267 | -4.356870875 | 3.73E-09 | 6.58E-08 | ITIH3 |  |
| ENSG00000221514 | -2.058009228 | 3.73E-09 | 6.58E-08 | SNORD111B | |
| ENSG00000107223 | -1.06105419 | 3.82E-09 | 6.73E-08 | EDF1 |  |
| ENSG00000172428 | -1.712992346 | 3.91E-09 | 6.87E-08 | COPS9 |  |
| ENSG00000172809 | 0.789550489 | 3.99E-09 | 7.01E-08 | RPL38 |  |
| ENSG00000156976 | -1.175512759 | 4.24E-09 | 7.45E-08 | EIF4A2 |  |
| ENSG00000199377 | -2.086768195 | 4.31E-09 | 7.55E-08 | RNU5F-1 |  |
| ENSG00000118200 | -1.201043549 | 4.33E-09 | 7.58E-08 | CAMSAP2 |  |
| ENSG00000111011 | -1.243379252 | 4.50E-09 | 7.87E-08 | RSRC2 |  |
| ENSG00000173812 | 0.980504652 | 4.52E-09 | 7.90E-08 | EIF1 |  |
| ENSG00000105058 | -1.549737061 | 4.55E-09 | 7.94E-08 | FAM32A |  |
| ENSG00000128191 | -1.679595021 | 4.71E-09 | 8.20E-08 | DGCR8 |  |
| ENSG00000208005 | -2.251050994 | 4.73E-09 | 8.24E-08 | MIR503 |  |
| ENSG00000135315 | -2.425361512 | 4.81E-09 | 8.37E-08 | CEP162 |  |
| ENSG00000114353 | -0.781364989 | 4.91E-09 | 8.53E-08 | GNAI2 |  |
| ENSG00000108424 | 0.685675174 | 5.08E-09 | 8.80E-08 | KPNB1 |  |
| ENSG00000124795 | -1.056562072 | 5.09E-09 | 8.81E-08 | DEK |  |
| ENSG00000173692 | 0.955031743 | 5.18E-09 | 8.97E-08 | PSMD1 |  |
| ENSG00000144283 | -1.467514483 | 5.20E-09 | 8.98E-08 | PKP4 |  |
| ENSG00000087206 | -1.431603101 | 5.20E-09 | 8.98E-08 | UIMC1 |  |
| ENSG00000112695 | 1.288734811 | 5.24E-09 | 9.03E-08 | COX7A2 |  |
| ENSG00000132646 | -0.786738136 | 5.27E-09 | 9.07E-08 | PCNA |  |
| ENSG00000156467 | -1.30620778 | 5.29E-09 | 9.11E-08 | UQCRB |  |
| ENSG00000126653 | -1.782606109 | 5.30E-09 | 9.11E-08 | NSRP1 |  |
| ENSG00000145685 | 1.695110449 | 5.35E-09 | 9.18E-08 | LHFPL2 |  |
| ENSG00000100994 | 1.018268764 | 5.63E-09 | 9.64E-08 | PYGB |  |
| ENSG00000240499 | -4.317995344 | 5.64E-09 | 9.66E-08 | AC004594.1 | |
| ENSG00000116016 | -0.877430653 | 5.70E-09 | 9.74E-08 | EPAS1 |  |
| ENSG00000033030 | -1.59406874 | 5.70E-09 | 9.74E-08 | ZCCHC8 |  |
| ENSG00000067560 | 0.857576351 | 5.77E-09 | 9.85E-08 | RHOA |  |
| ENSG00000150403 | -1.091377245 | 6.02E-09 | 1.03E-07 | TMCO3 |  |
| ENSG00000129250 | -0.952595256 | 6.04E-09 | 1.03E-07 | KIF1C |  |
| ENSG00000174231 | 0.670979566 | 6.07E-09 | 1.03E-07 | PRPF8 |  |
| ENSG00000187045 | -4.064509991 | 6.24E-09 | 1.06E-07 | TMPRSS6 |  |
| ENSG00000103222 | 1.024344461 | 6.48E-09 | 1.10E-07 | ABCC1 |  |
| ENSG00000104131 | -1.018253295 | 6.50E-09 | 1.10E-07 | EIF3J |  |
| ENSG00000204104 | -1.639583138 | 6.78E-09 | 1.15E-07 | TRAF3IP1 |  |
| ENSG00000175470 | -1.446544587 | 6.88E-09 | 1.16E-07 | PPP2R2D |  |
| ENSG00000138074 | 1.625690239 | 7.10E-09 | 1.20E-07 | SLC5A6 |  |
| ENSG00000171681 | -1.104388536 | 7.22E-09 | 1.22E-07 | ATF7IP |  |
| ENSG00000177628 | 1.941383819 | 7.34E-09 | 1.24E-07 | GBA |  |
| ENSG00000160752 | 1.35121564 | 7.46E-09 | 1.25E-07 | FDPS |  |
| ENSG00000172534 | 0.990179022 | 7.75E-09 | 1.30E-07 | HCFC1 |  |
| ENSG00000168394 | 1.32055572 | 7.82E-09 | 1.31E-07 | TAP1 |  |
| ENSG00000065526 | -1.089033261 | 7.82E-09 | 1.31E-07 | SPEN |  |
| ENSG00000237910 | -4.293202564 | 7.85E-09 | 1.31E-07 | MTCO1P18 |  |
| ENSG00000207721 | -1.67719767 | 8.24E-09 | 1.38E-07 | MIR186 |  |
| ENSG00000175166 | 0.709320357 | 8.71E-09 | 1.46E-07 | PSMD2 |  |
| ENSG00000147155 | 1.113493356 | 8.90E-09 | 1.49E-07 | EBP |  |
| ENSG00000201302 | -1.714681096 | 9.44E-09 | 1.57E-07 | SNORA65 |  |
| ENSG00000113810 | -0.820985667 | 9.45E-09 | 1.57E-07 | SMC4 |  |
| ENSG00000128340 | 1.963291464 | 9.50E-09 | 1.58E-07 | RAC2 |  |
| ENSG00000081051 | -4.00506282 | 9.51E-09 | 1.58E-07 | AFP |  |
| ENSG00000207726 | -1.880626091 | 9.98E-09 | 1.66E-07 | MIR455 |  |
| ENSG00000183726 | 1.175475671 | 1.05E-08 | 1.74E-07 | TMEM50A |  |
| ENSG00000171316 | -1.583465647 | 1.05E-08 | 1.74E-07 | CHD7 |  |
| ENSG00000100722 | -1.243934718 | 1.07E-08 | 1.77E-07 | ZC3H14 |  |
| ENSG00000199024 | 4.063852713 | 1.08E-08 | 1.79E-07 | MIR103A2 |  |
| ENSG00000142599 | -1.112626742 | 1.09E-08 | 1.79E-07 | RERE |  |
| ENSG00000130988 | -4.247221243 | 1.14E-08 | 1.88E-07 | RGN |  |
| ENSG00000087088 | 1.170247344 | 1.18E-08 | 1.93E-07 | BAX |  |
| ENSG00000184254 | 1.421963199 | 1.19E-08 | 1.95E-07 | ALDH1A3 |  |
| ENSG00000166004 | -2.177986277 | 1.20E-08 | 1.97E-07 | CEP295 |  |
| ENSG00000117318 | 0.983103563 | 1.23E-08 | 2.02E-07 | ID3 |  |
| ENSG00000130204 | 1.423033499 | 1.24E-08 | 2.03E-07 | TOMM40 |  |
| ENSG00000100815 | -1.195271839 | 1.25E-08 | 2.04E-07 | TRIP11 |  |
| ENSG00000089154 | 0.86072701 | 1.25E-08 | 2.05E-07 | GCN1 |  |
| ENSG00000135899 | -1.75237416 | 1.27E-08 | 2.08E-07 | SP110 |  |
| ENSG00000205581 | -1.120130417 | 1.29E-08 | 2.11E-07 | HMGN1 |  |
| ENSG00000151466 | -2.313796429 | 1.37E-08 | 2.23E-07 | SCLT1 |  |
| ENSG00000101882 | -1.994492574 | 1.37E-08 | 2.23E-07 | NKAP |  |
| ENSG00000071082 | -0.840642784 | 1.38E-08 | 2.25E-07 | RPL31 |  |
| ENSG00000138463 | 1.663177339 | 1.38E-08 | 2.25E-07 | DIRC2 |  |
| ENSG00000100916 | -1.966282848 | 1.39E-08 | 2.25E-07 | BRMS1L |  |
| ENSG00000100665 | -4.243756149 | 1.40E-08 | 2.26E-07 | SERPINA4 |  |
| ENSG00000052126 | -1.385019241 | 1.41E-08 | 2.28E-07 | PLEKHA5 |  |
| ENSG00000200879 | -2.432988546 | 1.42E-08 | 2.30E-07 | SNORD14E |  |
| ENSG00000116285 | -1.743480968 | 1.46E-08 | 2.36E-07 | ERRFI1 |  |
| ENSG00000108395 | -1.052678341 | 1.48E-08 | 2.39E-07 | TRIM37 |  |
| ENSG00000084207 | 0.939206367 | 1.49E-08 | 2.40E-07 | GSTP1 |  |
| ENSG00000137801 | 0.641096559 | 1.55E-08 | 2.49E-07 | THBS1 |  |
| ENSG00000032219 | -1.955826592 | 1.55E-08 | 2.49E-07 | ARID4A |  |
| ENSG00000083457 | 1.374495709 | 1.58E-08 | 2.53E-07 | ITGAE |  |
| ENSG00000213107 | -4.216409757 | 1.58E-08 | 2.54E-07 | AHCYP5 |  |
| ENSG00000180287 | -0.980563551 | 1.61E-08 | 2.58E-07 | PLD5 |  |
| ENSG00000229704 | -3.450786563 | 1.62E-08 | 2.59E-07 | EIF2S2P2 |  |
| ENSG00000007392 | -1.621091536 | 1.65E-08 | 2.64E-07 | LUC7L |  |
| ENSG00000104375 | -1.630354623 | 1.66E-08 | 2.65E-07 | STK3 |  |
| ENSG00000134697 | -1.168602036 | 1.76E-08 | 2.80E-07 | GNL2 |  |
| ENSG00000170421 | -0.744901381 | 1.77E-08 | 2.81E-07 | KRT8 |  |
| ENSG00000110107 | 0.978312303 | 1.88E-08 | 2.99E-07 | PRPF19 |  |
| ENSG00000149485 | 1.107716605 | 1.95E-08 | 3.10E-07 | FADS1 |  |
| ENSG00000158373 | -0.682408994 | 1.95E-08 | 3.10E-07 | HIST1H2BD | |
| ENSG00000127914 | -1.365531319 | 1.99E-08 | 3.16E-07 | AKAP9 |  |
| ENSG00000108819 | -0.896315919 | 2.03E-08 | 3.22E-07 | PPP1R9B |  |
| ENSG00000156411 | 1.517621193 | 2.04E-08 | 3.22E-07 | ATP5MPL |  |
| ENSG00000147133 | -1.54914374 | 2.06E-08 | 3.25E-07 | TAF1 |  |
| ENSG00000099194 | 0.905718197 | 2.19E-08 | 3.45E-07 | SCD |  |
| ENSG00000115310 | 0.723767963 | 2.23E-08 | 3.52E-07 | RTN4 |  |
| ENSG00000118900 | -1.229350995 | 2.23E-08 | 3.52E-07 | UBN1 |  |
| ENSG00000160285 | 1.010490941 | 2.28E-08 | 3.59E-07 | LSS |  |
| ENSG00000112983 | -0.966231323 | 2.30E-08 | 3.61E-07 | BRD8 |  |
| ENSG00000086062 | 1.228217362 | 2.39E-08 | 3.75E-07 | B4GALT1 |  |
| ENSG00000164951 | 1.555553841 | 2.39E-08 | 3.75E-07 | PDP1 |  |
| ENSG00000238741 | -1.723249017 | 2.40E-08 | 3.76E-07 | SCARNA7 |  |
| ENSG00000115415 | 1.101700504 | 2.40E-08 | 3.76E-07 | STAT1 |  |
| ENSG00000113368 | -0.779980747 | 2.45E-08 | 3.82E-07 | LMNB1 |  |
| ENSG00000147647 | -4.177979131 | 2.46E-08 | 3.84E-07 | DPYS |  |
| ENSG00000130826 | -0.817366129 | 2.47E-08 | 3.85E-07 | DKC1 |  |
| ENSG00000123136 | -0.94060287 | 2.50E-08 | 3.89E-07 | DDX39A |  |
| ENSG00000124783 | -0.855120137 | 2.51E-08 | 3.91E-07 | SSR1 |  |
| ENSG00000117133 | -1.835717519 | 2.52E-08 | 3.92E-07 | RPF1 |  |
| ENSG00000163510 | -1.497911926 | 2.57E-08 | 4.00E-07 | CWC22 |  |
| ENSG00000116171 | -1.376772162 | 2.77E-08 | 4.29E-07 | SCP2 |  |
| ENSG00000148296 | -1.684245703 | 2.99E-08 | 4.63E-07 | SURF6 |  |
| ENSG00000136811 | -1.228925173 | 3.09E-08 | 4.78E-07 | ODF2 |  |
| ENSG00000161638 | 0.888745509 | 3.11E-08 | 4.81E-07 | ITGA5 |  |
| ENSG00000266297 | -1.190303026 | 3.18E-08 | 4.92E-07 | MIR744 |  |
| ENSG00000125633 | -1.100285499 | 3.35E-08 | 5.18E-07 | CCDC93 |  |
| ENSG00000070961 | 1.026742895 | 3.43E-08 | 5.29E-07 | ATP2B1 |  |
| ENSG00000123485 | -1.116082364 | 3.44E-08 | 5.30E-07 | HJURP |  |
| ENSG00000099783 | -0.804968033 | 3.45E-08 | 5.30E-07 | HNRNPM |  |
| ENSG00000123374 | 1.428733315 | 3.54E-08 | 5.43E-07 | CDK2 |  |
| ENSG00000177058 | 1.769077121 | 3.60E-08 | 5.52E-07 | SLC38A9 |  |
| ENSG00000101132 | -2.339617887 | 3.60E-08 | 5.52E-07 | PFDN4 |  |
| ENSG00000111057 | -0.978484456 | 3.66E-08 | 5.61E-07 | KRT18 |  |
| ENSG00000091136 | 0.638697231 | 3.71E-08 | 5.67E-07 | LAMB1 |  |
| ENSG00000100292 | 2.309706225 | 3.71E-08 | 5.67E-07 | HMOX1 |  |
| ENSG00000062282 | -3.665938575 | 3.72E-08 | 5.68E-07 | DGAT2 |  |
| ENSG00000137831 | -1.135379353 | 3.73E-08 | 5.68E-07 | UACA |  |
| ENSG00000178982 | 1.055756228 | 3.76E-08 | 5.73E-07 | EIF3K |  |
| ENSG00000017427 | -4.122720103 | 3.85E-08 | 5.85E-07 | IGF1 |  |
| ENSG00000100347 | 1.187075104 | 3.88E-08 | 5.89E-07 | SAMM50 |  |
| ENSG00000106025 | -4.141483859 | 4.01E-08 | 6.09E-07 | TSPAN12 |  |
| ENSG00000169398 | -1.011994498 | 4.03E-08 | 6.12E-07 | PTK2 |  |
| ENSG00000148798 | -2.33578467 | 4.06E-08 | 6.15E-07 | INA |  |
| ENSG00000119705 | -0.88158951 | 4.07E-08 | 6.17E-07 | SLIRP |  |
| ENSG00000107262 | -1.846631918 | 4.20E-08 | 6.35E-07 | BAG1 |  |
| ENSG00000169562 | -4.119769796 | 4.23E-08 | 6.39E-07 | GJB1 |  |
| ENSG00000165678 | 1.031371514 | 4.57E-08 | 6.90E-07 | GHITM |  |
| ENSG00000106615 | 1.361547778 | 4.70E-08 | 7.09E-07 | RHEB |  |
| ENSG00000135211 | 2.422664749 | 4.88E-08 | 7.34E-07 | TMEM60 |  |
| ENSG00000107949 | -1.078943695 | 4.89E-08 | 7.35E-07 | BCCIP |  |
| ENSG00000139370 | 1.784503013 | 4.93E-08 | 7.40E-07 | SLC15A4 |  |
| ENSG00000143476 | 1.359015943 | 4.97E-08 | 7.46E-07 | DTL |  |
| ENSG00000101310 | 1.275099405 | 5.01E-08 | 7.51E-07 | SEC23B |  |
| ENSG00000181104 | 1.129159071 | 5.18E-08 | 7.76E-07 | F2R |  |
| ENSG00000146674 | -1.028847144 | 5.21E-08 | 7.80E-07 | IGFBP3 |  |
| ENSG00000175756 | -0.996596341 | 5.23E-08 | 7.82E-07 | AURKAIP1 |  |
| ENSG00000111711 | 1.610904017 | 5.24E-08 | 7.83E-07 | GOLT1B |  |
| ENSG00000284032 | -2.393960307 | 5.31E-08 | 7.92E-07 | MIR29A |  |
| ENSG00000250722 | -3.846327622 | 5.57E-08 | 8.30E-07 | SELENOP |  |
| ENSG00000186575 | -1.050909545 | 5.57E-08 | 8.30E-07 | NF2 |  |
| ENSG00000089177 | -1.717299789 | 5.65E-08 | 8.41E-07 | KIF16B |  |
| ENSG00000211575 | -2.231508415 | 5.66E-08 | 8.41E-07 | MIR760 |  |
| ENSG00000160691 | 1.327751245 | 5.97E-08 | 8.86E-07 | SHC1 |  |
| ENSG00000111775 | 1.200064762 | 6.20E-08 | 9.19E-07 | COX6A1 |  |
| ENSG00000021826 | -4.147436626 | 6.24E-08 | 9.24E-07 | CPS1 |  |
| ENSG00000214063 | 1.206166762 | 6.50E-08 | 9.62E-07 | TSPAN4 |  |
| ENSG00000072110 | -0.606236941 | 6.63E-08 | 9.80E-07 | ACTN1 |  |
| ENSG00000058804 | 1.278355304 | 6.70E-08 | 9.90E-07 | NDC1 |  |
| ENSG00000134779 | -1.069220041 | 6.84E-08 | 1.01E-06 | TPGS2 |  |
| ENSG00000215301 | 0.694520241 | 6.86E-08 | 1.01E-06 | DDX3X |  |
| ENSG00000200367 | -1.165328343 | 6.93E-08 | 1.02E-06 | SNORD113-8 | |
| ENSG00000170017 | -0.849150278 | 7.02E-08 | 1.03E-06 | ALCAM |  |
| ENSG00000109854 | 2.016475077 | 7.11E-08 | 1.04E-06 | HTATIP2 |  |
| ENSG00000143839 | -4.067176897 | 7.16E-08 | 1.05E-06 | REN |  |
| ENSG00000206503 | 0.84752246 | 7.19E-08 | 1.05E-06 | HLA-A |  |
| ENSG00000114544 | 2.043309837 | 7.20E-08 | 1.05E-06 | SLC41A3 |  |
| ENSG00000182827 | -1.511122891 | 7.20E-08 | 1.05E-06 | ACBD3 |  |
| ENSG00000242372 | 0.929832909 | 7.71E-08 | 1.13E-06 | EIF6 |  |
| ENSG00000261701 | -4.055272201 | 7.81E-08 | 1.14E-06 | HPR |  |
| ENSG00000118705 | 0.719239637 | 7.82E-08 | 1.14E-06 | RPN2 |  |
| ENSG00000182923 | -1.906208993 | 7.95E-08 | 1.16E-06 | CEP63 |  |
| ENSG00000185989 | -1.269974834 | 8.36E-08 | 1.22E-06 | RASA3 |  |
| ENSG00000230916 | -3.303086565 | 8.41E-08 | 1.22E-06 | MTCO1P53 |  |
| ENSG00000163939 | -1.102062016 | 8.59E-08 | 1.25E-06 | PBRM1 |  |
| ENSG00000176903 | 1.56132025 | 8.59E-08 | 1.25E-06 | PNMA1 |  |
| ENSG00000003056 | 1.324933004 | 8.72E-08 | 1.26E-06 | M6PR |  |
| ENSG00000138018 | 1.457130872 | 8.81E-08 | 1.27E-06 | SELENOI |  |
| ENSG00000059769 | -1.911407132 | 8.82E-08 | 1.27E-06 | DNAJC25 |  |
| ENSG00000055957 | -4.037912414 | 8.82E-08 | 1.27E-06 | ITIH1 |  |
| ENSG00000166881 | 1.321849837 | 8.86E-08 | 1.28E-06 | NEMP1 |  |
| ENSG00000148484 | 1.382046344 | 8.91E-08 | 1.29E-06 | RSU1 |  |
| ENSG00000159650 | -4.028858352 | 9.47E-08 | 1.37E-06 | UROC1 |  |
| ENSG00000196747 | -1.283544591 | 9.54E-08 | 1.37E-06 | HIST1H2AI | |
| ENSG00000126883 | 1.301500704 | 9.58E-08 | 1.38E-06 | NUP214 |  |
| ENSG00000140497 | 1.472552958 | 9.62E-08 | 1.38E-06 | SCAMP2 |  |
| ENSG00000105976 | 0.926396981 | 9.76E-08 | 1.40E-06 | MET |  |
| ENSG00000083937 | -1.34740158 | 9.77E-08 | 1.40E-06 | CHMP2B |  |
| ENSG00000126581 | -1.338845804 | 9.89E-08 | 1.42E-06 | BECN1 |  |
| ENSG00000113569 | 1.07867485 | 1.00E-07 | 1.43E-06 | NUP155 |  |
| ENSG00000130254 | -0.755625331 | 1.01E-07 | 1.44E-06 | SAFB2 |  |
| ENSG00000090020 | 1.307015231 | 1.02E-07 | 1.46E-06 | SLC9A1 |  |
| ENSG00000010292 | 0.833193523 | 1.02E-07 | 1.46E-06 | NCAPD2 |  |
| ENSG00000177600 | -0.668195578 | 1.03E-07 | 1.46E-06 | RPLP2 |  |
| ENSG00000013297 | 1.185876084 | 1.03E-07 | 1.46E-06 | CLDN11 |  |
| ENSG00000177469 | -0.686374153 | 1.03E-07 | 1.47E-06 | CAVIN1 |  |
| ENSG00000201938 | 1.280829343 | 1.03E-07 | 1.47E-06 | RF00019 |  |
| ENSG00000140299 | -1.12154545 | 1.04E-07 | 1.47E-06 | BNIP2 |  |
| ENSG00000205542 | -0.763695779 | 1.06E-07 | 1.50E-06 | TMSB4X |  |
| ENSG00000132128 | 1.549121179 | 1.07E-07 | 1.51E-06 | LRRC41 |  |
| ENSG00000145703 | -2.839296756 | 1.07E-07 | 1.51E-06 | IQGAP2 |  |
| ENSG00000143079 | -1.75383437 | 1.13E-07 | 1.60E-06 | CTTNBP2NL | |
| ENSG00000136485 | 0.960109256 | 1.16E-07 | 1.64E-06 | DCAF7 |  |
| ENSG00000170312 | 1.211572517 | 1.16E-07 | 1.64E-06 | CDK1 |  |
| ENSG00000134955 | 1.878412221 | 1.17E-07 | 1.65E-06 | SLC37A2 |  |
| ENSG00000100023 | -1.457666027 | 1.17E-07 | 1.65E-06 | PPIL2 |  |
| ENSG00000109501 | 1.13960321 | 1.18E-07 | 1.66E-06 | WFS1 |  |
| ENSG00000131966 | 1.733769244 | 1.22E-07 | 1.72E-06 | ACTR10 |  |
| ENSG00000249020 | -1.369868868 | 1.22E-07 | 1.72E-06 | SNORA58 |  |
| ENSG00000088035 | 1.863812517 | 1.24E-07 | 1.74E-06 | ALG6 |  |
| ENSG00000106804 | -3.774501457 | 1.24E-07 | 1.74E-06 | C5 |  |
| ENSG00000135926 | 1.403647811 | 1.24E-07 | 1.74E-06 | TMBIM1 |  |
| ENSG00000116133 | 0.829946397 | 1.26E-07 | 1.76E-06 | DHCR24 |  |
| ENSG00000172269 | 1.775497846 | 1.28E-07 | 1.79E-06 | DPAGT1 |  |
| ENSG00000068400 | -1.366051968 | 1.29E-07 | 1.80E-06 | GRIPAP1 |  |
| ENSG00000152465 | -1.358035538 | 1.29E-07 | 1.80E-06 | NMT2 |  |
| ENSG00000206838 | -1.942477181 | 1.31E-07 | 1.83E-06 | SNORA5A |  |
| ENSG00000091947 | 1.535765022 | 1.32E-07 | 1.84E-06 | TMEM101 |  |
| ENSG00000198786 | -0.701005974 | 1.32E-07 | 1.84E-06 | MT-ND5 |  |
| ENSG00000221983 | 0.826044934 | 1.35E-07 | 1.87E-06 | UBA52 |  |
| ENSG00000139722 | 1.61679218 | 1.35E-07 | 1.87E-06 | VPS37B |  |
| ENSG00000116871 | -1.052969442 | 1.36E-07 | 1.89E-06 | MAP7D1 |  |
| ENSG00000131069 | 1.302255581 | 1.39E-07 | 1.93E-06 | ACSS2 |  |
| ENSG00000222650 | -2.932423406 | 1.41E-07 | 1.95E-06 | RNU2-70P |  |
| ENSG00000245910 | -1.487703429 | 1.41E-07 | 1.96E-06 | SNHG6 |  |
| ENSG00000160075 | -1.110507348 | 1.43E-07 | 1.98E-06 | SSU72 |  |
| ENSG00000167491 | -0.953101067 | 1.46E-07 | 2.02E-06 | GATAD2A |  |
| ENSG00000141499 | 1.407410487 | 1.48E-07 | 2.04E-06 | WRAP53 |  |
| ENSG00000106397 | 0.938825852 | 1.57E-07 | 2.16E-06 | PLOD3 |  |
| ENSG00000089048 | -1.312756406 | 1.59E-07 | 2.19E-06 | ESF1 |  |
| ENSG00000198000 | -1.420839713 | 1.59E-07 | 2.19E-06 | NOL8 |  |
| ENSG00000145192 | -3.970565018 | 1.66E-07 | 2.29E-06 | AHSG |  |
| ENSG00000117360 | -1.29834949 | 1.69E-07 | 2.32E-06 | PRPF3 |  |
| ENSG00000221978 | -1.203068803 | 1.71E-07 | 2.34E-06 | CCNL2 |  |
| ENSG00000154767 | -1.655331544 | 1.76E-07 | 2.42E-06 | XPC |  |
| ENSG00000100350 | 1.502010192 | 1.77E-07 | 2.42E-06 | FOXRED2 |  |
| ENSG00000141905 | -0.762754843 | 1.79E-07 | 2.45E-06 | NFIC |  |
| ENSG00000131981 | 1.25948575 | 1.83E-07 | 2.50E-06 | LGALS3 |  |
| ENSG00000112739 | -1.768435036 | 1.84E-07 | 2.52E-06 | PRPF4B |  |
| ENSG00000198650 | -4.086790282 | 1.87E-07 | 2.55E-06 | TAT |  |
| ENSG00000197535 | -1.231514339 | 1.88E-07 | 2.57E-06 | MYO5A |  |
| ENSG00000151348 | 1.121826302 | 1.89E-07 | 2.57E-06 | EXT2 |  |
| ENSG00000123562 | -0.966231284 | 1.91E-07 | 2.60E-06 | MORF4L2 |  |
| ENSG00000101474 | 1.074803353 | 1.92E-07 | 2.61E-06 | APMAP |  |
| ENSG00000207175 | -1.878590339 | 1.93E-07 | 2.62E-06 | RNU1-67P |  |
| ENSG00000169908 | 1.12223527 | 1.95E-07 | 2.65E-06 | TM4SF1 |  |
| ENSG00000088038 | -1.975552599 | 2.07E-07 | 2.80E-06 | CNOT3 |  |
| ENSG00000197321 | -1.608133578 | 2.21E-07 | 2.99E-06 | SVIL |  |
| ENSG00000198830 | -0.733694735 | 2.23E-07 | 3.02E-06 | HMGN2 |  |
| ENSG00000165632 | -2.107522388 | 2.23E-07 | 3.02E-06 | TAF3 |  |
| ENSG00000134716 | -3.942784296 | 2.25E-07 | 3.04E-06 | CYP2J2 |  |
| ENSG00000064651 | 1.344528959 | 2.27E-07 | 3.05E-06 | SLC12A2 |  |
| ENSG00000185619 | -1.2960537 | 2.27E-07 | 3.05E-06 | PCGF3 |  |
| ENSG00000258890 | -1.485006805 | 2.27E-07 | 3.05E-06 | CEP95 |  |
| ENSG00000103995 | -1.27166152 | 2.28E-07 | 3.07E-06 | CEP152 |  |
| ENSG00000152193 | 2.162472815 | 2.29E-07 | 3.07E-06 | RNF219 |  |
| ENSG00000075213 | -1.709927762 | 2.30E-07 | 3.09E-06 | SEMA3A |  |
| ENSG00000109670 | -1.97216962 | 2.34E-07 | 3.13E-06 | FBXW7 |  |
| ENSG00000127463 | 0.911961736 | 2.34E-07 | 3.14E-06 | EMC1 |  |
| ENSG00000167815 | 1.035263102 | 2.36E-07 | 3.16E-06 | PRDX2 |  |
| ENSG00000121289 | -1.433891635 | 2.37E-07 | 3.17E-06 | CEP89 |  |
| ENSG00000163161 | -1.126215805 | 2.40E-07 | 3.20E-06 | ERCC3 |  |
| ENSG00000264994 | -1.243178843 | 2.41E-07 | 3.22E-06 | SNORD92 |  |
| ENSG00000200897 | -2.87754431 | 2.41E-07 | 3.22E-06 | RF00284 |  |
| ENSG00000046604 | 1.517518533 | 2.47E-07 | 3.29E-06 | DSG2 |  |
| ENSG00000134013 | 0.712823653 | 2.65E-07 | 3.52E-06 | LOXL2 |  |
| ENSG00000163565 | -1.037410994 | 2.65E-07 | 3.53E-06 | IFI16 |  |
| ENSG00000100813 | -0.750884147 | 2.66E-07 | 3.53E-06 | ACIN1 |  |
| ENSG00000072042 | 1.510347 | 2.66E-07 | 3.54E-06 | RDH11 |  |
| ENSG00000100462 | 1.355711715 | 2.67E-07 | 3.54E-06 | PRMT5 |  |
| ENSG00000143546 | -3.355343819 | 2.68E-07 | 3.56E-06 | S100A8 |  |
| ENSG00000238181 | -3.920552575 | 2.71E-07 | 3.59E-06 | AHCYP2 |  |
| ENSG00000198886 | -0.733450622 | 2.74E-07 | 3.62E-06 | MT-ND4 |  |
| ENSG00000100991 | 1.038303181 | 2.76E-07 | 3.65E-06 | TRPC4AP |  |
| ENSG00000155304 | 1.251917715 | 2.77E-07 | 3.65E-06 | HSPA13 |  |
| ENSG00000159842 | -0.862907751 | 2.81E-07 | 3.71E-06 | ABR |  |
| ENSG00000232752 | -3.92727846 | 2.87E-07 | 3.79E-06 | MTCO3P21 |  |
| ENSG00000198734 | -3.903323359 | 2.93E-07 | 3.86E-06 | F5 |  |
| ENSG00000109919 | 1.137376771 | 2.95E-07 | 3.88E-06 | MTCH2 |  |
| ENSG00000111275 | -2.289434284 | 2.96E-07 | 3.89E-06 | ALDH2 |  |
| ENSG00000226479 | 1.746392896 | 2.99E-07 | 3.93E-06 | TMEM185B |  |
| ENSG00000122729 | 1.103815963 | 3.00E-07 | 3.94E-06 | ACO1 |  |
| ENSG00000116237 | 0.826303561 | 3.02E-07 | 3.96E-06 | ICMT |  |
| ENSG00000283891 | -3.319225225 | 3.03E-07 | 3.97E-06 | MIR628 |  |
| ENSG00000123636 | -1.615692358 | 3.05E-07 | 3.99E-06 | BAZ2B |  |
| ENSG00000147862 | -0.956184328 | 3.19E-07 | 4.17E-06 | NFIB |  |
| ENSG00000075856 | -1.112626865 | 3.19E-07 | 4.17E-06 | SART3 |  |
| ENSG00000120963 | 1.777120965 | 3.23E-07 | 4.22E-06 | ZNF706 |  |
| ENSG00000117450 | 0.950476218 | 3.30E-07 | 4.30E-06 | PRDX1 |  |
| ENSG00000128973 | 1.957019666 | 3.30E-07 | 4.30E-06 | CLN6 |  |
| ENSG00000212443 | -2.122484676 | 3.43E-07 | 4.46E-06 | SNORA53 |  |
| ENSG00000063177 | 0.833558497 | 3.44E-07 | 4.47E-06 | RPL18 |  |
| ENSG00000149231 | -1.282879334 | 3.46E-07 | 4.49E-06 | CCDC82 |  |
| ENSG00000115446 | 1.513949113 | 3.49E-07 | 4.52E-06 | UNC50 |  |
| ENSG00000132406 | 1.48351264 | 3.50E-07 | 4.54E-06 | TMEM128 |  |
| ENSG00000221491 | -2.980388181 | 3.55E-07 | 4.59E-06 | SNORA2C |  |
| ENSG00000177606 | -0.783757129 | 3.55E-07 | 4.60E-06 | JUN |  |
| ENSG00000157827 | -1.161368837 | 3.58E-07 | 4.63E-06 | FMNL2 |  |
| ENSG00000116539 | -1.084509252 | 3.68E-07 | 4.75E-06 | ASH1L |  |
| ENSG00000124702 | 1.206810732 | 3.73E-07 | 4.82E-06 | KLHDC3 |  |
| ENSG00000105707 | -3.874362488 | 3.80E-07 | 4.90E-06 | HPN |  |
| ENSG00000165669 | -1.594706149 | 3.81E-07 | 4.91E-06 | FAM204A |  |
| ENSG00000169607 | -1.234790488 | 3.82E-07 | 4.92E-06 | CKAP2L |  |
| ENSG00000083223 | -1.351912111 | 3.83E-07 | 4.92E-06 | TUT7 |  |
| ENSG00000144029 | -1.0327793 | 4.03E-07 | 5.18E-06 | MRPS5 |  |
| ENSG00000153317 | -0.864945572 | 4.05E-07 | 5.20E-06 | ASAP1 |  |
| ENSG00000164308 | 0.936700265 | 4.13E-07 | 5.30E-06 | ERAP2 |  |
| ENSG00000103978 | 1.454847909 | 4.17E-07 | 5.34E-06 | TMEM87A |  |
| ENSG00000117592 | 1.024147211 | 4.19E-07 | 5.36E-06 | PRDX6 |  |
| ENSG00000080608 | -1.336442515 | 4.20E-07 | 5.38E-06 | PUM3 |  |
| ENSG00000165282 | 1.445356813 | 4.22E-07 | 5.39E-06 | PIGO |  |
| ENSG00000124762 | 1.33005789 | 4.23E-07 | 5.40E-06 | CDKN1A |  |
| ENSG00000143416 | -3.477485727 | 4.24E-07 | 5.41E-06 | SELENBP1 |  |
| ENSG00000187239 | -1.520444952 | 4.36E-07 | 5.56E-06 | FNBP1 |  |
| ENSG00000144747 | -0.994637349 | 4.37E-07 | 5.56E-06 | TMF1 |  |
| ENSG00000112514 | 1.027749861 | 4.37E-07 | 5.57E-06 | CUTA |  |
| ENSG00000255717 | -1.21873061 | 4.40E-07 | 5.60E-06 | SNHG1 |  |
| ENSG00000242337 | -3.860161983 | 4.43E-07 | 5.63E-06 | TFP1 |  |
| ENSG00000200534 | -1.913818223 | 4.49E-07 | 5.70E-06 | SNORA33 |  |
| ENSG00000207965 | -2.031833157 | 4.50E-07 | 5.71E-06 | MIR629 |  |
| ENSG00000086712 | -1.750431195 | 4.51E-07 | 5.71E-06 | TXLNG |  |
| ENSG00000184220 | -1.256224038 | 4.52E-07 | 5.72E-06 | CMSS1 |  |
| ENSG00000143815 | 1.15354741 | 4.53E-07 | 5.73E-06 | LBR |  |
| ENSG00000273703 | -1.166104995 | 4.58E-07 | 5.79E-06 | HIST1H2BM | |
| ENSG00000145623 | 0.853546966 | 4.61E-07 | 5.83E-06 | OSMR |  |
| ENSG00000166164 | -1.711801766 | 4.67E-07 | 5.90E-06 | BRD7 |  |
| ENSG00000075785 | 0.769259009 | 4.69E-07 | 5.91E-06 | RAB7A |  |
| ENSG00000217128 | 0.992720655 | 4.81E-07 | 6.05E-06 | FNIP1 |  |
| ENSG00000213585 | 0.896787626 | 4.86E-07 | 6.11E-06 | VDAC1 |  |
| ENSG00000115685 | -1.073972872 | 4.88E-07 | 6.13E-06 | PPP1R7 |  |
| ENSG00000201786 | 2.947966652 | 4.92E-07 | 6.18E-06 | RF00019 |  |
| ENSG00000110628 | 2.306620828 | 5.03E-07 | 6.31E-06 | SLC22A18 |  |
| ENSG00000115207 | 1.632234428 | 5.03E-07 | 6.31E-06 | GTF3C2 |  |
| ENSG00000206917 | -2.604117425 | 5.04E-07 | 6.32E-06 | RNU1-52P |  |
| ENSG00000129484 | -1.345000374 | 5.11E-07 | 6.39E-06 | PARP2 |  |
| ENSG00000139718 | -1.357154578 | 5.19E-07 | 6.49E-06 | SETD1B |  |
| ENSG00000125944 | -0.798501874 | 5.24E-07 | 6.54E-06 | HNRNPR |  |
| ENSG00000090889 | -0.966211778 | 5.26E-07 | 6.56E-06 | KIF4A |  |
| ENSG00000141574 | 1.40006252 | 5.30E-07 | 6.61E-06 | SECTM1 |  |
| ENSG00000222414 | -1.19610406 | 5.35E-07 | 6.66E-06 | RNU2-59P |  |
| ENSG00000154310 | -1.198536356 | 5.40E-07 | 6.73E-06 | TNIK |  |
| ENSG00000148337 | -1.031474142 | 5.42E-07 | 6.74E-06 | CIZ1 |  |
| ENSG00000233219 | -3.834625777 | 5.45E-07 | 6.78E-06 | AC008080.3 | |
| ENSG00000257740 | 1.597830343 | 5.46E-07 | 6.78E-06 | AC073896.3 | |
| ENSG00000279086 | -3.622443812 | 5.47E-07 | 6.79E-06 | AC073130.3 | |
| ENSG00000178538 | -3.839489063 | 5.49E-07 | 6.80E-06 | CA8 |  |
| ENSG00000231925 | 1.148004923 | 5.56E-07 | 6.89E-06 | TAPBP |  |
| ENSG00000153922 | -1.094836455 | 5.60E-07 | 6.94E-06 | CHD1 |  |
| ENSG00000200197 | -1.673705227 | 5.65E-07 | 6.99E-06 | RNU1-21P |  |
| ENSG00000122705 | -0.732697574 | 5.75E-07 | 7.10E-06 | CLTA |  |
| ENSG00000215883 | -2.827278962 | 5.76E-07 | 7.11E-06 | CYB5RL |  |
| ENSG00000126432 | 1.088848737 | 5.81E-07 | 7.17E-06 | PRDX5 |  |
| ENSG00000255291 | -2.9134011 | 5.83E-07 | 7.18E-06 | HMGB1P40 |  |
| ENSG00000079246 | 0.618204942 | 5.86E-07 | 7.21E-06 | XRCC5 |  |
| ENSG00000233026 | -3.827250765 | 5.86E-07 | 7.21E-06 | MTCO1P5 |  |
| ENSG00000006118 | 1.310688542 | 5.87E-07 | 7.22E-06 | TMEM132A |  |
| ENSG00000197324 | 1.132049843 | 6.01E-07 | 7.38E-06 | LRP10 |  |
| ENSG00000137575 | 1.07627459 | 6.20E-07 | 7.61E-06 | SDCBP |  |
| ENSG00000185104 | -0.994068436 | 6.25E-07 | 7.67E-06 | FAF1 |  |
| ENSG00000162244 | -1.632537907 | 6.32E-07 | 7.74E-06 | RPL29 |  |
| ENSG00000131475 | 1.036958781 | 6.46E-07 | 7.90E-06 | VPS25 |  |
| ENSG00000176915 | -0.744932363 | 6.52E-07 | 7.98E-06 | ANKLE2 |  |
| ENSG00000107537 | -1.963132733 | 6.59E-07 | 8.06E-06 | PHYH |  |
| ENSG00000198668 | -0.73289534 | 6.61E-07 | 8.07E-06 | CALM1 |  |
| ENSG00000140538 | -3.810433233 | 6.69E-07 | 8.16E-06 | NTRK3 |  |
| ENSG00000143198 | 1.167841648 | 6.81E-07 | 8.30E-06 | MGST3 |  |
| ENSG00000206612 | -1.729836737 | 6.86E-07 | 8.36E-06 | SNORA2A |  |
| ENSG00000108669 | -1.404024514 | 6.90E-07 | 8.40E-06 | CYTH1 |  |
| ENSG00000234017 | -3.820520424 | 6.93E-07 | 8.43E-06 | AC022018.1 | |
| ENSG00000244038 | 0.845195216 | 7.19E-07 | 8.74E-06 | DDOST |  |
| ENSG00000062716 | 0.94795608 | 7.25E-07 | 8.80E-06 | VMP1 |  |
| ENSG00000184007 | 0.69955824 | 7.41E-07 | 8.99E-06 | PTP4A2 |  |
| ENSG00000138109 | -3.799166788 | 7.51E-07 | 9.10E-06 | CYP2C9 |  |
| ENSG00000151176 | 1.004418065 | 7.57E-07 | 9.16E-06 | PLBD2 |  |
| ENSG00000143793 | -1.535037019 | 7.69E-07 | 9.31E-06 | C1orf35 |  |
| ENSG00000069535 | -3.789461746 | 7.80E-07 | 9.43E-06 | MAOB |  |
| ENSG00000111832 | -1.493404374 | 8.13E-07 | 9.82E-06 | RWDD1 |  |
| ENSG00000011454 | -1.044432849 | 8.16E-07 | 9.84E-06 | RABGAP1 |  |
| ENSG00000112531 | -0.784140269 | 8.19E-07 | 9.88E-06 | QKI |  |
| ENSG00000188338 | -3.122773938 | 8.24E-07 | 9.93E-06 | SLC38A3 |  |
| ENSG00000164307 | 1.041373294 | 8.35E-07 | 1.01E-05 | ERAP1 |  |
| ENSG00000168066 | -0.790479984 | 8.40E-07 | 1.01E-05 | SF1 |  |
| ENSG00000158710 | 0.656961736 | 8.52E-07 | 1.02E-05 | TAGLN2 |  |
| ENSG00000198938 | -0.66352958 | 8.54E-07 | 1.03E-05 | MT-CO3 |  |
| ENSG00000166913 | 0.607560823 | 8.63E-07 | 1.04E-05 | YWHAB |  |
| ENSG00000207181 | -3.553105539 | 8.68E-07 | 1.04E-05 | SNORA14B |  |
| ENSG00000171988 | -1.171315359 | 8.72E-07 | 1.04E-05 | JMJD1C |  |
| ENSG00000142541 | -0.744129229 | 8.90E-07 | 1.06E-05 | RPL13A |  |
| ENSG00000108829 | 0.702291726 | 8.96E-07 | 1.07E-05 | LRRC59 |  |
| ENSG00000176845 | -1.352801056 | 8.99E-07 | 1.07E-05 | METRNL |  |
| ENSG00000265241 | -1.959726058 | 8.99E-07 | 1.07E-05 | RBM8A |  |
| ENSG00000170142 | 1.443374191 | 9.20E-07 | 1.10E-05 | UBE2E1 |  |
| ENSG00000143627 | -3.601861785 | 9.20E-07 | 1.10E-05 | PKLR |  |
| ENSG00000212607 | -1.845721718 | 9.25E-07 | 1.10E-05 | SNORA3B |  |
| ENSG00000207757 | -1.68432593 | 9.30E-07 | 1.11E-05 | MIR93 |  |
| ENSG00000156650 | -1.387479063 | 9.34E-07 | 1.11E-05 | KAT6B |  |
| ENSG00000119922 | -0.896459354 | 9.47E-07 | 1.12E-05 | IFIT2 |  |
| ENSG00000113580 | 0.706414283 | 9.48E-07 | 1.12E-05 | NR3C1 |  |
| ENSG00000141349 | 1.735315014 | 9.50E-07 | 1.13E-05 | G6PC3 |  |
| ENSG00000141577 | -1.595275214 | 9.70E-07 | 1.15E-05 | CEP131 |  |
| ENSG00000164610 | -2.513267651 | 9.78E-07 | 1.16E-05 | RP9 |  |
| ENSG00000135956 | 1.450119994 | 9.82E-07 | 1.16E-05 | TMEM127 |  |
| ENSG00000017483 | 2.022170304 | 9.83E-07 | 1.16E-05 | SLC38A5 |  |
| ENSG00000208772 | -1.525665917 | 9.96E-07 | 1.18E-05 | SNORD94 |  |
| ENSG00000179218 | -0.558852961 | 1.01E-06 | 1.19E-05 | CALR |  |
| ENSG00000236254 | -3.375004778 | 1.01E-06 | 1.19E-05 | MTND4P14 |  |
| ENSG00000166813 | -1.364455019 | 1.03E-06 | 1.21E-05 | KIF7 |  |
| ENSG00000127054 | -1.002879597 | 1.06E-06 | 1.25E-05 | INTS11 |  |
| ENSG00000196230 | 0.660608701 | 1.06E-06 | 1.25E-05 | TUBB |  |
| ENSG00000196455 | 1.499731087 | 1.06E-06 | 1.25E-05 | PIK3R4 |  |
| ENSG00000163933 | 1.831757988 | 1.07E-06 | 1.26E-05 | RFT1 |  |
| ENSG00000156515 | 0.687842442 | 1.11E-06 | 1.30E-05 | HK1 |  |
| ENSG00000113300 | -1.013329359 | 1.12E-06 | 1.31E-05 | CNOT6 |  |
| ENSG00000132432 | 1.030275221 | 1.16E-06 | 1.35E-05 | SEC61G |  |
| ENSG00000070756 | -0.611512834 | 1.16E-06 | 1.36E-05 | PABPC1 |  |
| ENSG00000172292 | 1.788313902 | 1.17E-06 | 1.37E-05 | CERS6 |  |
| ENSG00000176809 | 1.707976221 | 1.18E-06 | 1.38E-05 | LRRC37A3 |  |
| ENSG00000121621 | -1.213529341 | 1.20E-06 | 1.40E-05 | KIF18A |  |
| ENSG00000178913 | -1.060879302 | 1.21E-06 | 1.42E-05 | TAF7 |  |
| ENSG00000135966 | -1.406694883 | 1.23E-06 | 1.43E-05 | TGFBRAP1 |  |
| ENSG00000143418 | 0.730095477 | 1.23E-06 | 1.43E-05 | CERS2 |  |
| ENSG00000206897 | -2.451369925 | 1.26E-06 | 1.47E-05 | SNORA9B |  |
| ENSG00000115539 | -1.373211103 | 1.30E-06 | 1.51E-05 | PDCL3 |  |
| ENSG00000197170 | 0.832663555 | 1.31E-06 | 1.52E-05 | PSMD12 |  |
| ENSG00000151322 | -3.733194714 | 1.31E-06 | 1.52E-05 | NPAS3 |  |
| ENSG00000139624 | 1.328624869 | 1.32E-06 | 1.53E-05 | CERS5 |  |
| ENSG00000101189 | -1.340455113 | 1.33E-06 | 1.54E-05 | MRGBP |  |
| ENSG00000201595 | -1.370452482 | 1.33E-06 | 1.54E-05 | RNA5SP132 | |
| ENSG00000173638 | 1.580401785 | 1.33E-06 | 1.54E-05 | SLC19A1 |  |
| ENSG00000064545 | 1.681815585 | 1.34E-06 | 1.55E-05 | TMEM161A |  |
| ENSG00000115053 | -0.717075491 | 1.34E-06 | 1.55E-05 | NCL |  |
| ENSG00000201950 | 1.875792531 | 1.34E-06 | 1.55E-05 | SNORD113-9 | |
| ENSG00000248333 | -1.758822062 | 1.35E-06 | 1.55E-05 | CDK11B |  |
| ENSG00000200959 | -2.020401533 | 1.35E-06 | 1.55E-05 | SNORA74A |  |
| ENSG00000025293 | -0.926037221 | 1.36E-06 | 1.57E-05 | PHF20 |  |
| ENSG00000169062 | -1.592815204 | 1.36E-06 | 1.57E-05 | UPF3A |  |
| ENSG00000065150 | 0.634513562 | 1.37E-06 | 1.57E-05 | IPO5 |  |
| ENSG00000207233 | -2.58064469 | 1.38E-06 | 1.59E-05 | SNORA37 |  |
| ENSG00000139437 | -1.298650496 | 1.40E-06 | 1.61E-05 | TCHP |  |
| ENSG00000113441 | 0.965441101 | 1.43E-06 | 1.65E-05 | LNPEP |  |
| ENSG00000176619 | -0.650881287 | 1.46E-06 | 1.67E-05 | LMNB2 |  |
| ENSG00000198356 | -1.015117326 | 1.46E-06 | 1.68E-05 | ASNA1 |  |
| ENSG00000198743 | 1.255154376 | 1.48E-06 | 1.69E-05 | SLC5A3 |  |
| ENSG00000137561 | -3.713906745 | 1.48E-06 | 1.70E-05 | TTPA |  |
| ENSG00000270282 | -3.725173371 | 1.53E-06 | 1.75E-05 | AL096855.2 | |
| ENSG00000222076 | -2.149053925 | 1.55E-06 | 1.77E-05 | RNU2-3P |  |
| ENSG00000110321 | -0.644319068 | 1.60E-06 | 1.83E-05 | EIF4G2 |  |
| ENSG00000007001 | -3.706243645 | 1.61E-06 | 1.84E-05 | UPP2 |  |
| ENSG00000099875 | -1.369082155 | 1.64E-06 | 1.87E-05 | MKNK2 |  |
| ENSG00000162889 | -0.925359944 | 1.68E-06 | 1.91E-05 | MAPKAPK2 |  |
| ENSG00000099795 | -1.155127131 | 1.72E-06 | 1.95E-05 | NDUFB7 |  |
| ENSG00000134531 | 1.215931401 | 1.72E-06 | 1.95E-05 | EMP1 |  |
| ENSG00000114867 | -0.636586694 | 1.73E-06 | 1.96E-05 | EIF4G1 |  |
| ENSG00000117425 | -3.692871455 | 1.73E-06 | 1.96E-05 | PTCH2 |  |
| ENSG00000227682 | -3.693548837 | 1.73E-06 | 1.97E-05 | ATP5F1AP2 | |
| ENSG00000251538 | -3.70181626 | 1.76E-06 | 1.99E-05 | LINC02201 | |
| ENSG00000120438 | 0.87106263 | 1.77E-06 | 2.01E-05 | TCP1 |  |
| ENSG00000166900 | -1.13587509 | 1.80E-06 | 2.03E-05 | STX3 |  |
| ENSG00000151790 | -3.499390332 | 1.80E-06 | 2.03E-05 | TDO2 |  |
| ENSG00000147955 | 1.506074292 | 1.80E-06 | 2.03E-05 | SIGMAR1 |  |
| ENSG00000202538 | -1.871755183 | 1.85E-06 | 2.09E-05 | RNU4-2 |  |
| ENSG00000145241 | -2.841684804 | 1.86E-06 | 2.10E-05 | CENPC |  |
| ENSG00000180210 | -3.683817895 | 1.88E-06 | 2.11E-05 | F2 |  |
| ENSG00000163931 | 0.686260186 | 1.88E-06 | 2.11E-05 | TKT |  |
| ENSG00000025800 | 0.748890377 | 1.88E-06 | 2.11E-05 | KPNA6 |  |
| ENSG00000069943 | 2.449006552 | 1.91E-06 | 2.14E-05 | PIGB |  |
| ENSG00000116717 | -1.233131951 | 1.93E-06 | 2.17E-05 | GADD45A |  |
| ENSG00000118137 | -3.177140307 | 1.96E-06 | 2.20E-05 | APOA1 |  |
| ENSG00000149115 | 0.93335009 | 1.99E-06 | 2.23E-05 | TNKS1BP1 |  |
| ENSG00000251733 | 0.617074437 | 1.99E-06 | 2.23E-05 | SCARNA8 |  |
| ENSG00000115970 | 1.670713558 | 2.00E-06 | 2.24E-05 | THADA |  |
| ENSG00000157227 | 0.994956946 | 2.00E-06 | 2.24E-05 | MMP14 |  |
| ENSG00000137817 | -1.679130644 | 2.01E-06 | 2.25E-05 | PARP6 |  |
| ENSG00000128829 | -0.939185017 | 2.03E-06 | 2.27E-05 | EIF2AK4 |  |
| ENSG00000005421 | -3.688730025 | 2.06E-06 | 2.30E-05 | PON1 |  |
| ENSG00000167085 | 1.239768882 | 2.11E-06 | 2.35E-05 | PHB |  |
| ENSG00000077097 | -0.902154979 | 2.12E-06 | 2.36E-05 | TOP2B |  |
| ENSG00000005483 | -1.14487807 | 2.12E-06 | 2.36E-05 | KMT2E |  |
| ENSG00000041802 | -1.077035131 | 2.12E-06 | 2.36E-05 | LSG1 |  |
| ENSG00000108846 | 1.279985201 | 2.12E-06 | 2.36E-05 | ABCC3 |  |
| ENSG00000107404 | -1.315261312 | 2.13E-06 | 2.37E-05 | DVL1 |  |
| ENSG00000279620 | -3.680506874 | 2.14E-06 | 2.37E-05 | AC099494.3 | |
| ENSG00000284591 | -3.670253304 | 2.16E-06 | 2.40E-05 | AC006927.4 | |
| ENSG00000239128 | -2.880229867 | 2.17E-06 | 2.40E-05 | SNORD13P3 | |
| ENSG00000111371 | 0.837359267 | 2.19E-06 | 2.43E-05 | SLC38A1 |  |
| ENSG00000170949 | -2.234409033 | 2.19E-06 | 2.43E-05 | ZNF160 |  |
| ENSG00000075292 | -1.08265003 | 2.21E-06 | 2.44E-05 | ZNF638 |  |
| ENSG00000067900 | -1.035740505 | 2.23E-06 | 2.46E-05 | ROCK1 |  |
| ENSG00000102854 | 1.141666299 | 2.25E-06 | 2.49E-05 | MSLN |  |
| ENSG00000136271 | 1.022403498 | 2.33E-06 | 2.57E-05 | DDX56 |  |
| ENSG00000129596 | -2.547026475 | 2.35E-06 | 2.59E-05 | CDO1 |  |
| ENSG00000186832 | -3.44572815 | 2.38E-06 | 2.62E-05 | KRT16 |  |
| ENSG00000137309 | 1.136224923 | 2.40E-06 | 2.64E-05 | HMGA1 |  |
| ENSG00000166012 | -1.063275785 | 2.41E-06 | 2.66E-05 | TAF1D |  |
| ENSG00000106012 | -1.811296921 | 2.42E-06 | 2.67E-05 | IQCE |  |
| ENSG00000113194 | 1.108311755 | 2.43E-06 | 2.67E-05 | FAF2 |  |
| ENSG00000130559 | -1.354587953 | 2.46E-06 | 2.70E-05 | CAMSAP1 |  |
| ENSG00000136935 | -1.314220504 | 2.47E-06 | 2.70E-05 | GOLGA1 |  |
| ENSG00000126247 | 0.723636582 | 2.51E-06 | 2.75E-05 | CAPNS1 |  |
| ENSG00000143368 | 1.274727022 | 2.53E-06 | 2.77E-05 | SF3B4 |  |
| ENSG00000169299 | 1.281931604 | 2.56E-06 | 2.80E-05 | PGM2 |  |
| ENSG00000204262 | 0.754031234 | 2.60E-06 | 2.84E-05 | COL5A2 |  |
| ENSG00000163520 | 0.892701784 | 2.60E-06 | 2.84E-05 | FBLN2 |  |
| ENSG00000213398 | -3.170752274 | 2.61E-06 | 2.85E-05 | LCAT |  |
| ENSG00000102226 | 0.8167256 | 2.61E-06 | 2.85E-05 | USP11 |  |
| ENSG00000102081 | -0.89543799 | 2.62E-06 | 2.85E-05 | FMR1 |  |
| ENSG00000161057 | 0.900911274 | 2.64E-06 | 2.88E-05 | PSMC2 |  |
| ENSG00000118412 | -1.524309286 | 2.65E-06 | 2.89E-05 | CASP8AP2 |  |
| ENSG00000130724 | -1.007133098 | 2.66E-06 | 2.90E-05 | CHMP2A |  |
| ENSG00000138029 | 1.372230636 | 2.68E-06 | 2.91E-05 | HADHB |  |
| ENSG00000157637 | 1.307737056 | 2.72E-06 | 2.96E-05 | SLC38A10 |  |
| ENSG00000084072 | -1.615777188 | 2.72E-06 | 2.96E-05 | PPIE |  |
| ENSG00000109118 | -1.28389325 | 2.75E-06 | 2.98E-05 | PHF12 |  |
| ENSG00000145632 | -0.648894268 | 2.81E-06 | 3.04E-05 | PLK2 |  |
| ENSG00000207784 | -2.722442923 | 2.88E-06 | 3.12E-05 | MIR542 |  |
| ENSG00000198171 | -1.101079449 | 2.88E-06 | 3.12E-05 | DDRGK1 |  |
| ENSG00000099800 | -1.046362884 | 2.93E-06 | 3.16E-05 | TIMM13 |  |
| ENSG00000205726 | -1.093652258 | 2.93E-06 | 3.17E-05 | ITSN1 |  |
| ENSG00000135446 | 1.026245069 | 2.95E-06 | 3.19E-05 | CDK4 |  |
| ENSG00000204323 | -3.634985291 | 2.96E-06 | 3.19E-05 | SMIM5 |  |
| ENSG00000136942 | -0.871805864 | 3.07E-06 | 3.31E-05 | RPL35 |  |
| ENSG00000171914 | -0.991622124 | 3.08E-06 | 3.32E-05 | TLN2 |  |
| ENSG00000096092 | 1.762693942 | 3.15E-06 | 3.39E-05 | TMEM14A |  |
| ENSG00000141076 | 1.076813653 | 3.15E-06 | 3.39E-05 | UTP4 |  |
| ENSG00000115520 | 1.425611808 | 3.17E-06 | 3.40E-05 | COQ10B |  |
| ENSG00000014641 | 0.695535375 | 3.21E-06 | 3.45E-05 | MDH1 |  |
| ENSG00000172037 | 0.870788683 | 3.22E-06 | 3.45E-05 | LAMB2 |  |
| ENSG00000163512 | -1.785585512 | 3.26E-06 | 3.50E-05 | AZI2 |  |
| ENSG00000154473 | 0.775151591 | 3.33E-06 | 3.57E-05 | BUB3 |  |
| ENSG00000131773 | -2.525320249 | 3.36E-06 | 3.60E-05 | KHDRBS3 |  |
| ENSG00000165916 | -0.642445314 | 3.38E-06 | 3.61E-05 | PSMC3 |  |
| ENSG00000156282 | -3.614850138 | 3.43E-06 | 3.67E-05 | CLDN17 |  |
| ENSG00000055609 | -1.122374046 | 3.46E-06 | 3.69E-05 | KMT2C |  |
| ENSG00000010379 | -3.617842406 | 3.47E-06 | 3.71E-05 | SLC6A13 |  |
| ENSG00000128951 | 1.003950052 | 3.49E-06 | 3.72E-05 | DUT |  |
| ENSG00000101337 | 0.808242808 | 3.53E-06 | 3.77E-05 | TM9SF4 |  |
| ENSG00000207779 | -3.152260911 | 3.58E-06 | 3.81E-05 | MIR15B |  |
| ENSG00000100614 | -1.218627255 | 3.58E-06 | 3.81E-05 | PPM1A |  |
| ENSG00000112130 | -1.411381502 | 3.59E-06 | 3.81E-05 | RNF8 |  |
| ENSG00000112096 | 0.742200761 | 3.61E-06 | 3.84E-05 | SOD2 |  |
| ENSG00000124831 | -1.001220675 | 3.63E-06 | 3.86E-05 | LRRFIP1 |  |
| ENSG00000179119 | -1.705246379 | 3.78E-06 | 4.01E-05 | SPTY2D1 |  |
| ENSG00000116747 | 1.197029864 | 3.85E-06 | 4.08E-05 | TROVE2 |  |
| ENSG00000201470 | 2.097029698 | 3.90E-06 | 4.14E-05 | RNY4P7 |  |
| ENSG00000131473 | 0.603291833 | 3.92E-06 | 4.15E-05 | ACLY |  |
| ENSG00000260765 | -3.589847719 | 3.93E-06 | 4.16E-05 | CES1P2 |  |
| ENSG00000102316 | 0.979943355 | 3.97E-06 | 4.20E-05 | MAGED2 |  |
| ENSG00000115828 | 2.216797293 | 4.01E-06 | 4.24E-05 | QPCT |  |
| ENSG00000200492 | -3.38801654 | 4.03E-06 | 4.25E-05 | RF00012 |  |
| ENSG00000133318 | 0.871319112 | 4.23E-06 | 4.47E-05 | RTN3 |  |
| ENSG00000214025 | -3.579465155 | 4.30E-06 | 4.54E-05 | ATP5PBP4 |  |
| ENSG00000124571 | 0.91674995 | 4.33E-06 | 4.56E-05 | XPO5 |  |
| ENSG00000261889 | -1.273696001 | 4.35E-06 | 4.58E-05 | AC108134.2 | |
| ENSG00000003436 | 0.927619401 | 4.42E-06 | 4.65E-05 | TFPI |  |
| ENSG00000144744 | 0.978724947 | 4.47E-06 | 4.70E-05 | UBA3 |  |
| ENSG00000198856 | 1.794659002 | 4.48E-06 | 4.71E-05 | OSTC |  |
| ENSG00000116221 | 1.235495208 | 4.59E-06 | 4.82E-05 | MRPL37 |  |
| ENSG00000139344 | -3.563322439 | 4.72E-06 | 4.95E-05 | AMDHD1 |  |
| ENSG00000162341 | 1.222528056 | 4.75E-06 | 4.98E-05 | TPCN2 |  |
| ENSG00000089685 | -0.729154268 | 4.76E-06 | 4.98E-05 | BIRC5 |  |
| ENSG00000248144 | -3.562723395 | 4.76E-06 | 4.98E-05 | ADH1C |  |
| ENSG00000068438 | 1.01581374 | 4.86E-06 | 5.08E-05 | FTSJ1 |  |
| ENSG00000134352 | 0.717576849 | 4.94E-06 | 5.16E-05 | IL6ST |  |
| ENSG00000062485 | 1.073210523 | 4.94E-06 | 5.16E-05 | CS |  |
| ENSG00000207864 | -1.389375943 | 4.98E-06 | 5.20E-05 | MIR27B |  |
| ENSG00000122965 | -1.250405139 | 5.05E-06 | 5.27E-05 | RBM19 |  |
| ENSG00000189403 | -0.779729617 | 5.10E-06 | 5.31E-05 | HMGB1 |  |
| ENSG00000169641 | -0.91619559 | 5.19E-06 | 5.40E-05 | LUZP1 |  |
| ENSG00000152894 | 0.961864878 | 5.33E-06 | 5.54E-05 | PTPRK |  |
| ENSG00000095261 | 1.681582815 | 5.33E-06 | 5.54E-05 | PSMD5 |  |
| ENSG00000163507 | 1.098453729 | 5.34E-06 | 5.55E-05 | CIP2A |  |
| ENSG00000207758 | -0.848840698 | 5.56E-06 | 5.77E-05 | MIR532 |  |
| ENSG00000149968 | 1.321962495 | 5.58E-06 | 5.79E-05 | MMP3 |  |
| ENSG00000159086 | -1.112348714 | 5.65E-06 | 5.85E-05 | PAXBP1 |  |
| ENSG00000129003 | 0.883940239 | 5.74E-06 | 5.95E-05 | VPS13C |  |
| ENSG00000253504 | -3.537895518 | 5.85E-06 | 6.06E-05 | MTCYBP19 |  |
| ENSG00000100888 | -0.814984606 | 5.90E-06 | 6.10E-05 | CHD8 |  |
| ENSG00000204386 | 1.047936676 | 5.92E-06 | 6.12E-05 | NEU1 |  |
| ENSG00000109084 | 1.640665274 | 5.99E-06 | 6.19E-05 | TMEM97 |  |
| ENSG00000196365 | -0.804937056 | 5.99E-06 | 6.19E-05 | LONP1 |  |
| ENSG00000126698 | -0.994675074 | 6.01E-06 | 6.21E-05 | DNAJC8 |  |
| ENSG00000157593 | 1.598689639 | 6.05E-06 | 6.24E-05 | SLC35B2 |  |
| ENSG00000222536 | -3.338891341 | 6.07E-06 | 6.25E-05 | RNU2-39P |  |
| ENSG00000122692 | 1.13755465 | 6.10E-06 | 6.28E-05 | SMU1 |  |
| ENSG00000143761 | 0.668087264 | 6.16E-06 | 6.34E-05 | ARF1 |  |
| ENSG00000011275 | -1.064598227 | 6.20E-06 | 6.37E-05 | RNF216 |  |
| ENSG00000134321 | -1.186288136 | 6.27E-06 | 6.44E-05 | RSAD2 |  |
| ENSG00000156642 | 0.903933948 | 6.42E-06 | 6.59E-05 | NPTN |  |
| ENSG00000136731 | 0.680652804 | 6.53E-06 | 6.70E-05 | UGGT1 |  |
| ENSG00000132561 | -1.421252197 | 6.55E-06 | 6.72E-05 | MATN2 |  |
| ENSG00000005471 | -3.521094962 | 6.57E-06 | 6.73E-05 | ABCB4 |  |
| ENSG00000184260 | 1.379568932 | 6.58E-06 | 6.74E-05 | HIST2H2AC | |
| ENSG00000230453 | -2.068012504 | 6.62E-06 | 6.77E-05 | ANKRD18B |  |
| ENSG00000176102 | 1.164074782 | 6.62E-06 | 6.77E-05 | CSTF3 |  |
| ENSG00000152683 | 1.591735392 | 6.71E-06 | 6.86E-05 | SLC30A6 |  |
| ENSG00000164402 | -1.186715446 | 6.79E-06 | 6.93E-05 | 8-Sep |  |
| ENSG00000198015 | 1.485935503 | 6.84E-06 | 6.98E-05 | MRPL42 |  |
| ENSG00000111707 | -1.245787626 | 6.87E-06 | 7.01E-05 | SUDS3 |  |
| ENSG00000176783 | -1.144030788 | 6.90E-06 | 7.02E-05 | RUFY1 |  |
| ENSG00000136541 | -3.327334091 | 6.93E-06 | 7.06E-05 | ERMN |  |
| ENSG00000168556 | -1.493738634 | 7.06E-06 | 7.18E-05 | ING2 |  |
| ENSG00000165271 | 1.257534789 | 7.06E-06 | 7.18E-05 | NOL6 |  |
| ENSG00000212588 | -2.124905833 | 7.21E-06 | 7.33E-05 | SNORA26 |  |
| ENSG00000182890 | -3.512690587 | 7.22E-06 | 7.33E-05 | GLUD2 |  |
| ENSG00000125740 | -3.1377565 | 7.23E-06 | 7.34E-05 | FOSB |  |
| ENSG00000126773 | 1.096222663 | 7.29E-06 | 7.39E-05 | PCNX4 |  |
| ENSG00000154845 | -0.865681225 | 7.31E-06 | 7.41E-05 | PPP4R1 |  |
| ENSG00000079134 | -1.583149885 | 7.32E-06 | 7.41E-05 | THOC1 |  |
| ENSG00000120915 | -3.508548622 | 7.61E-06 | 7.70E-05 | EPHX2 |  |
| ENSG00000105939 | -0.758689328 | 7.62E-06 | 7.70E-05 | ZC3HAV1 |  |
| ENSG00000200800 | -2.12574823 | 7.70E-06 | 7.78E-05 | RNU1-130P | |
| ENSG00000207651 | -1.383436605 | 7.82E-06 | 7.89E-05 | MIR28 |  |
| ENSG00000105486 | -0.898711205 | 7.82E-06 | 7.89E-05 | LIG1 |  |
| ENSG00000164902 | -0.983230503 | 7.90E-06 | 7.96E-05 | PHAX |  |
| ENSG00000175697 | -3.350134863 | 7.91E-06 | 7.96E-05 | GPR156 |  |
| ENSG00000009307 | -0.558535781 | 8.01E-06 | 8.06E-05 | CSDE1 |  |
| ENSG00000099940 | -1.352541104 | 8.04E-06 | 8.08E-05 | SNAP29 |  |
| ENSG00000169375 | -0.782343753 | 8.04E-06 | 8.08E-05 | SIN3A |  |
| ENSG00000130396 | -0.944004962 | 8.14E-06 | 8.18E-05 | AFDN |  |
| ENSG00000108797 | 1.10370212 | 8.28E-06 | 8.32E-05 | CNTNAP1 |  |
| ENSG00000125482 | -1.615873567 | 8.53E-06 | 8.56E-05 | TTF1 |  |
| ENSG00000105127 | -1.129910992 | 8.66E-06 | 8.68E-05 | AKAP8 |  |
| ENSG00000147459 | -0.854428476 | 8.67E-06 | 8.69E-05 | DOCK5 |  |
| ENSG00000135677 | 0.750020256 | 8.79E-06 | 8.80E-05 | GNS |  |
| ENSG00000138442 | 1.294207829 | 8.82E-06 | 8.82E-05 | WDR12 |  |
| ENSG00000275126 | 1.404136442 | 8.84E-06 | 8.84E-05 | HIST1H4L |  |
| ENSG00000135931 | -1.046641741 | 8.93E-06 | 8.93E-05 | ARMC9 |  |
| ENSG00000248282 | -3.488251296 | 8.98E-06 | 8.97E-05 | MTCYBP44 |  |
| ENSG00000134058 | 1.695459359 | 9.04E-06 | 9.01E-05 | CDK7 |  |
| ENSG00000115761 | -1.035040135 | 9.04E-06 | 9.01E-05 | NOL10 |  |
| ENSG00000140284 | -2.669379182 | 9.19E-06 | 9.16E-05 | SLC27A2 |  |
| ENSG00000131351 | -1.584941551 | 9.33E-06 | 9.29E-05 | HAUS8 |  |
| ENSG00000090372 | -0.707542897 | 9.39E-06 | 9.34E-05 | STRN4 |  |
| ENSG00000107863 | -1.507704984 | 9.46E-06 | 9.40E-05 | ARHGAP21 |  |
| ENSG00000120647 | -1.293257457 | 9.79E-06 | 9.73E-05 | CCDC77 |  |
| ENSG00000130985 | 0.656846356 | 9.88E-06 | 9.81E-05 | UBA1 |  |
| ENSG00000163739 | 0.897866393 | 9.88E-06 | 9.81E-05 | CXCL1 |  |
| ENSG00000253522 | 2.483435174 | 9.91E-06 | 9.83E-05 | MIR3142HG | |
| ENSG00000109072 | -1.902425483 | 1.04E-05 | 0.000103044 | VTN |  |
| ENSG00000142230 | 0.773708727 | 1.04E-05 | 0.000103457 | SAE1 |  |
| ENSG00000145817 | 1.027773482 | 1.06E-05 | 0.000104465 | YIPF5 |  |
| ENSG00000125166 | 1.036052548 | 1.07E-05 | 0.000105445 | GOT2 |  |
| ENSG00000132382 | 0.869143539 | 1.07E-05 | 0.000105671 | MYBBP1A |  |
| ENSG00000143507 | -2.856662758 | 1.08E-05 | 0.000106897 | DUSP10 |  |
| ENSG00000107937 | -0.839537974 | 1.08E-05 | 0.000106955 | GTPBP4 |  |
| ENSG00000164741 | -1.006601132 | 1.09E-05 | 0.0001075 | DLC1 |  |
| ENSG00000199168 | 1.470125278 | 1.10E-05 | 0.000108567 | MIR374A |  |
| ENSG00000105954 | -3.447898166 | 1.11E-05 | 0.000109106 | NPVF |  |
| ENSG00000109756 | -1.018566492 | 1.12E-05 | 0.000110027 | RAPGEF2 |  |
| ENSG00000168172 | -0.859001486 | 1.12E-05 | 0.000110027 | HOOK3 |  |
| ENSG00000139168 | -1.238061633 | 1.12E-05 | 0.00011009 | ZCRB1 |  |
| ENSG00000199805 | -2.553214122 | 1.13E-05 | 0.000110799 | RNU1-134P | |
| ENSG00000070047 | -0.97661813 | 1.13E-05 | 0.000110898 | PHRF1 |  |
| ENSG00000162627 | -1.241485471 | 1.14E-05 | 0.000112158 | SNX7 |  |
| ENSG00000113552 | 0.92933031 | 1.15E-05 | 0.000112717 | GNPDA1 |  |
| ENSG00000170266 | 0.966184353 | 1.15E-05 | 0.000112717 | GLB1 |  |
| ENSG00000173599 | -1.523017865 | 1.15E-05 | 0.000112753 | PC |  |
| ENSG00000175334 | 0.771153877 | 1.16E-05 | 0.000113085 | BANF1 |  |
| ENSG00000109685 | -0.741113794 | 1.17E-05 | 0.000114132 | NSD2 |  |
| ENSG00000145386 | -0.701633102 | 1.17E-05 | 0.000114546 | CCNA2 |  |
| ENSG00000206775 | -1.333140607 | 1.18E-05 | 0.000114799 | SNORD37 |  |
| ENSG00000057019 | 0.779311213 | 1.19E-05 | 0.000115806 | DCBLD2 |  |
| ENSG00000114850 | 0.743259585 | 1.20E-05 | 0.000116551 | SSR3 |  |
| ENSG00000104332 | -1.517762001 | 1.20E-05 | 0.000117123 | SFRP1 |  |
| ENSG00000105011 | 0.857442762 | 1.20E-05 | 0.00011713 | ASF1B |  |
| ENSG00000230302 | -3.44557483 | 1.21E-05 | 0.000117415 | MTND3P4 |  |
| ENSG00000197386 | 0.785323718 | 1.21E-05 | 0.00011787 | HTT |  |
| ENSG00000143164 | -1.032818995 | 1.22E-05 | 0.000118054 | DCAF6 |  |
| ENSG00000182400 | 1.723208569 | 1.22E-05 | 0.000118254 | TRAPPC6B |  |
| ENSG00000229980 | -3.095084293 | 1.24E-05 | 0.000119961 | TOB1-AS1 |  |
| ENSG00000196655 | 1.391617006 | 1.25E-05 | 0.000121369 | TRAPPC4 |  |
| ENSG00000106608 | 1.325149542 | 1.25E-05 | 0.000121541 | URGCP |  |
| ENSG00000166025 | -1.075550824 | 1.26E-05 | 0.000122323 | AMOTL1 |  |
| ENSG00000011007 | -1.267180675 | 1.27E-05 | 0.000122653 | ELOA |  |
| ENSG00000148175 | 1.051916307 | 1.28E-05 | 0.000123638 | STOM |  |
| ENSG00000146457 | -1.196903373 | 1.28E-05 | 0.000124065 | WTAP |  |
| ENSG00000196743 | 1.451785511 | 1.29E-05 | 0.000124308 | GM2A |  |
| ENSG00000259986 | 0.880776598 | 1.29E-05 | 0.000124308 | AC103876.1 | |
| ENSG00000084110 | -2.898944083 | 1.29E-05 | 0.000124521 | HAL |  |
| ENSG00000199094 | -1.370897518 | 1.30E-05 | 0.000125409 | MIR30C2 |  |
| ENSG00000125356 | 1.219491207 | 1.31E-05 | 0.00012631 | NDUFA1 |  |
| ENSG00000112655 | 1.200641306 | 1.35E-05 | 0.00013027 | PTK7 |  |
| ENSG00000091428 | -2.619183924 | 1.36E-05 | 0.000130593 | RAPGEF4 |  |
| ENSG00000125354 | -1.353589591 | 1.38E-05 | 0.000132995 | 6-Sep |  |
| ENSG00000183535 | -3.420191873 | 1.39E-05 | 0.000133799 | COL18A1-AS1 | |
| ENSG00000070814 | -0.759848095 | 1.40E-05 | 0.000134299 | TCOF1 |  |
| ENSG00000031823 | -0.880528591 | 1.40E-05 | 0.000134558 | RANBP3 |  |
| ENSG00000186910 | -3.41314476 | 1.42E-05 | 0.00013572 | SERPINA11 | |
| ENSG00000196591 | -0.759245784 | 1.42E-05 | 0.00013572 | HDAC2 |  |
| ENSG00000187164 | -1.404787629 | 1.42E-05 | 0.000136301 | SHTN1 |  |
| ENSG00000008838 | 1.241753636 | 1.43E-05 | 0.000136643 | MED24 |  |
| ENSG00000170606 | 0.570926141 | 1.43E-05 | 0.000136858 | HSPA4 |  |
| ENSG00000108784 | 1.331536269 | 1.43E-05 | 0.000136858 | NAGLU |  |
| ENSG00000019995 | -1.119414221 | 1.44E-05 | 0.000137792 | ZRANB1 |  |
| ENSG00000102007 | 0.954906327 | 1.47E-05 | 0.000140171 | PLP2 |  |
| ENSG00000200795 | -1.059175262 | 1.47E-05 | 0.000140171 | RNU4-1 |  |
| ENSG00000003989 | -3.065560645 | 1.47E-05 | 0.000140353 | SLC7A2 |  |
| ENSG00000102882 | 1.352357856 | 1.48E-05 | 0.000140552 | MAPK3 |  |
| ENSG00000164040 | 0.943553123 | 1.51E-05 | 0.000143983 | PGRMC2 |  |
| ENSG00000117594 | -3.407396866 | 1.52E-05 | 0.000144534 | HSD11B1 |  |
| ENSG00000130695 | -1.285962875 | 1.52E-05 | 0.000144792 | CEP85 |  |
| ENSG00000165283 | 0.900631002 | 1.57E-05 | 0.000148632 | STOML2 |  |
| ENSG00000128833 | -2.918726672 | 1.57E-05 | 0.000148632 | MYO5C |  |
| ENSG00000239043 | -1.039077949 | 1.59E-05 | 0.000151012 | SNORD127 |  |
| ENSG00000132405 | -1.373977965 | 1.59E-05 | 0.000151012 | TBC1D14 |  |
| ENSG00000100804 | 0.974924899 | 1.60E-05 | 0.000151538 | PSMB5 |  |
| ENSG00000169567 | 0.788920617 | 1.62E-05 | 0.000153028 | HINT1 |  |
| ENSG00000164244 | 1.11996228 | 1.64E-05 | 0.000155195 | PRRC1 |  |
| ENSG00000047457 | -3.220347211 | 1.68E-05 | 0.000158436 | CP |  |
| ENSG00000107959 | 0.987497636 | 1.69E-05 | 0.000159565 | PITRM1 |  |
| ENSG00000162607 | -0.795809692 | 1.69E-05 | 0.000159734 | USP1 |  |
| ENSG00000232936 | -3.399905932 | 1.69E-05 | 0.000159734 | AL157400.2 | |
| ENSG00000014216 | 0.790317318 | 1.70E-05 | 0.000160208 | CAPN1 |  |
| ENSG00000141543 | 0.597524578 | 1.72E-05 | 0.000162125 | EIF4A3 |  |
| ENSG00000119596 | -0.781302482 | 1.73E-05 | 0.000162798 | YLPM1 |  |
| ENSG00000135480 | -0.498962292 | 1.74E-05 | 0.00016321 | KRT7 |  |
| ENSG00000171298 | 1.012351296 | 1.74E-05 | 0.00016321 | GAA |  |
| ENSG00000101365 | 0.825547758 | 1.74E-05 | 0.00016351 | IDH3B |  |
| ENSG00000198920 | -1.488241634 | 1.75E-05 | 0.000163892 | KIAA0753 |  |
| ENSG00000115419 | 0.956192141 | 1.75E-05 | 0.000164376 | GLS |  |
| ENSG00000082898 | 0.621483674 | 1.76E-05 | 0.000165493 | XPO1 |  |
| ENSG00000104760 | -3.387518274 | 1.77E-05 | 0.000165844 | FGL1 |  |
| ENSG00000083845 | 0.608223948 | 1.79E-05 | 0.000167811 | RPS5 |  |
| ENSG00000143799 | -0.704336133 | 1.81E-05 | 0.000169842 | PARP1 |  |
| ENSG00000107798 | 0.972013593 | 1.82E-05 | 0.000170172 | LIPA |  |
| ENSG00000101745 | -1.046514823 | 1.84E-05 | 0.000171984 | ANKRD12 |  |
| ENSG00000102931 | -2.819394986 | 1.87E-05 | 0.000174457 | ARL2BP |  |
| ENSG00000207392 | -1.523714728 | 1.88E-05 | 0.000175329 | SNORA20 |  |
| ENSG00000102974 | -1.040303283 | 1.88E-05 | 0.000175484 | CTCF |  |
| ENSG00000198899 | -0.813098972 | 1.90E-05 | 0.000176955 | MT-ATP6 |  |
| ENSG00000223156 | -1.762298493 | 1.94E-05 | 0.000180702 | RNU2-18P |  |
| ENSG00000115128 | -2.03847312 | 1.95E-05 | 0.000181557 | SF3B6 |  |
| ENSG00000135945 | -1.384039679 | 1.96E-05 | 0.000182885 | REV1 |  |
| ENSG00000144659 | 1.597293508 | 1.97E-05 | 0.000183157 | SLC25A38 |  |
| ENSG00000142945 | 0.736142811 | 1.97E-05 | 0.000183545 | KIF2C |  |
| ENSG00000089280 | 0.568907202 | 1.99E-05 | 0.000185317 | FUS |  |
| ENSG00000125386 | -1.449648037 | 2.00E-05 | 0.000186112 | FAM193A |  |
| ENSG00000181789 | 0.687292626 | 2.03E-05 | 0.00018878 | COPG1 |  |
| ENSG00000188153 | 1.129348054 | 2.04E-05 | 0.00018878 | COL4A5 |  |
| ENSG00000099937 | -3.361660224 | 2.04E-05 | 0.000188831 | SERPIND1 |  |
| ENSG00000222985 | -2.678091094 | 2.06E-05 | 0.00019032 | RNU2-14P |  |
| ENSG00000188559 | -2.162655196 | 2.08E-05 | 0.000192781 | RALGAPA2 |  |
| ENSG00000143369 | 1.069643456 | 2.09E-05 | 0.000193518 | ECM1 |  |
| ENSG00000255417 | -3.05413418 | 2.10E-05 | 0.000193636 | MTCO2P15 |  |
| ENSG00000117399 | 0.902596337 | 2.12E-05 | 0.000195654 | CDC20 |  |
| ENSG00000134001 | 0.855290351 | 2.19E-05 | 0.000202418 | EIF2S1 |  |
| ENSG00000176444 | -1.058799045 | 2.19E-05 | 0.000202418 | CLK2 |  |
| ENSG00000104142 | 1.253793381 | 2.25E-05 | 0.000207115 | VPS18 |  |
| ENSG00000132780 | -0.684453607 | 2.28E-05 | 0.000210431 | NASP |  |
| ENSG00000167258 | -0.934169967 | 2.29E-05 | 0.000211271 | CDK12 |  |
| ENSG00000198251 | -3.345838601 | 2.30E-05 | 0.000212074 | CYP2A7P2 |  |
| ENSG00000196912 | -2.755919218 | 2.31E-05 | 0.000212429 | ANKRD36B |  |
| ENSG00000065883 | -0.984228146 | 2.32E-05 | 0.000213483 | CDK13 |  |
| ENSG00000207827 | -1.142587132 | 2.32E-05 | 0.000213483 | MIR30A |  |
| ENSG00000125257 | 0.933641158 | 2.34E-05 | 0.000214302 | ABCC4 |  |
| ENSG00000106344 | -1.032123191 | 2.34E-05 | 0.000214733 | RBM28 |  |
| ENSG00000008988 | 0.711577683 | 2.35E-05 | 0.000215346 | RPS20 |  |
| ENSG00000089094 | -1.115529081 | 2.36E-05 | 0.000216227 | KDM2B |  |
| ENSG00000118482 | -1.036093983 | 2.38E-05 | 0.000217433 | PHF3 |  |
| ENSG00000213949 | 1.324473355 | 2.38E-05 | 0.000217895 | ITGA1 |  |
| ENSG00000198231 | -0.70207077 | 2.38E-05 | 0.000217945 | DDX42 |  |
| ENSG00000087245 | 1.386846124 | 2.40E-05 | 0.000219686 | MMP2 |  |
| ENSG00000112312 | -0.963488188 | 2.42E-05 | 0.000221006 | GMNN |  |
| ENSG00000124067 | 0.881522987 | 2.43E-05 | 0.000221939 | SLC12A4 |  |
| ENSG00000207292 | -1.219483622 | 2.43E-05 | 0.000221939 | RF00019 |  |
| ENSG00000214944 | -0.913940326 | 2.44E-05 | 0.00022276 | ARHGEF28 |  |
| ENSG00000119541 | -0.790540123 | 2.46E-05 | 0.000224079 | VPS4B |  |
| ENSG00000266179 | -2.889690939 | 2.48E-05 | 0.000225506 | AC025627.3 | |
| ENSG00000006625 | 1.461169158 | 2.49E-05 | 0.00022671 | GGCT |  |
| ENSG00000200204 | -2.337988277 | 2.50E-05 | 0.000227146 | RNU1-22P |  |
| ENSG00000275084 | -2.400474359 | 2.50E-05 | 0.000227172 | SNORD91B |  |
| ENSG00000163220 | -3.329220614 | 2.53E-05 | 0.000229629 | S100A9 |  |
| ENSG00000179115 | 0.961634815 | 2.54E-05 | 0.000230203 | FARSA |  |
| ENSG00000164611 | 0.604196885 | 2.57E-05 | 0.000232848 | PTTG1 |  |
| ENSG00000166226 | 0.811033182 | 2.57E-05 | 0.000233052 | CCT2 |  |
| ENSG00000101457 | -1.196785388 | 2.58E-05 | 0.000233589 | DNTTIP1 |  |
| ENSG00000173208 | -3.339638299 | 2.58E-05 | 0.000233891 | ABCD2 |  |
| ENSG00000101146 | 1.104858261 | 2.61E-05 | 0.000236125 | RAE1 |  |
| ENSG00000174744 | -1.088967643 | 2.63E-05 | 0.000237811 | BRMS1 |  |
| ENSG00000149547 | 1.271347547 | 2.63E-05 | 0.000237811 | EI24 |  |
| ENSG00000103642 | 1.167047258 | 2.64E-05 | 0.000237811 | LACTB |  |
| ENSG00000089022 | -1.503012337 | 2.64E-05 | 0.000237811 | MAPKAPK5 |  |
| ENSG00000159202 | 0.670032885 | 2.66E-05 | 0.000239466 | UBE2Z |  |
| ENSG00000152952 | 0.621893487 | 2.68E-05 | 0.000241178 | PLOD2 |  |
| ENSG00000104783 | 2.153953963 | 2.71E-05 | 0.000244165 | KCNN4 |  |
| ENSG00000234586 | -3.318226188 | 2.72E-05 | 0.000244898 | AL161449.1 | |
| ENSG00000189091 | 0.638170752 | 2.73E-05 | 0.00024541 | SF3B3 |  |
| ENSG00000090674 | 1.295327678 | 2.77E-05 | 0.000249303 | MCOLN1 |  |
| ENSG00000125968 | -0.726919507 | 2.83E-05 | 0.000254013 | ID1 |  |
| ENSG00000146276 | -2.991342642 | 2.84E-05 | 0.000255344 | GABRR1 |  |
| ENSG00000214517 | 1.030992654 | 2.91E-05 | 0.000260936 | PPME1 |  |
| ENSG00000207008 | -2.242530839 | 2.92E-05 | 0.000261412 | SNORA54 |  |
| ENSG00000130638 | 0.636269084 | 2.92E-05 | 0.000261412 | ATXN10 |  |
| ENSG00000242593 | -2.291085914 | 2.92E-05 | 0.000261603 | AC006148.1 | |
| ENSG00000132485 | -1.090468382 | 2.97E-05 | 0.000265853 | ZRANB2 |  |
| ENSG00000165140 | -3.314271968 | 2.98E-05 | 0.000266913 | FBP1 |  |
| ENSG00000180957 | 0.70402708 | 2.99E-05 | 0.000267012 | PITPNB |  |
| ENSG00000142546 | -0.8594976 | 3.00E-05 | 0.000268199 | NOSIP |  |
| ENSG00000106245 | -1.412030443 | 3.02E-05 | 0.000269343 | BUD31 |  |
| ENSG00000104824 | 0.822177392 | 3.05E-05 | 0.000272542 | HNRNPL |  |
| ENSG00000152404 | -1.560901888 | 3.06E-05 | 0.000273062 | CWF19L2 |  |
| ENSG00000077463 | 2.131604194 | 3.08E-05 | 0.000274818 | SIRT6 |  |
| ENSG00000169020 | -1.415162014 | 3.09E-05 | 0.000275121 | ATP5ME |  |
| ENSG00000207633 | -1.867781288 | 3.13E-05 | 0.000278163 | MIR505 |  |
| ENSG00000234842 | -3.303563018 | 3.17E-05 | 0.000282377 | MTCO2P16 |  |
| ENSG00000238795 | -1.479794498 | 3.20E-05 | 0.000284187 | SCARNA12 |  |
| ENSG00000166348 | -1.666991878 | 3.22E-05 | 0.000286002 | USP54 |  |
| ENSG00000198715 | 1.744386305 | 3.23E-05 | 0.000286896 | GLMP |  |
| ENSG00000176340 | 0.975078074 | 3.24E-05 | 0.000287205 | COX8A |  |
| ENSG00000173674 | -0.95084886 | 3.26E-05 | 0.00028889 | EIF1AX |  |
| ENSG00000135018 | 0.813238884 | 3.27E-05 | 0.000290169 | UBQLN1 |  |
| ENSG00000136021 | 1.440551127 | 3.28E-05 | 0.000290443 | SCYL2 |  |
| ENSG00000267135 | -3.289197918 | 3.29E-05 | 0.000290772 | AD000091.1 | |
| ENSG00000106927 | -3.287288125 | 3.34E-05 | 0.00029508 | AMBP |  |
| ENSG00000101323 | -2.633237035 | 3.34E-05 | 0.000295127 | HAO1 |  |
| ENSG00000143537 | 0.893670252 | 3.34E-05 | 0.000295342 | ADAM15 |  |
| ENSG00000113360 | -0.917279133 | 3.35E-05 | 0.00029555 | DROSHA |  |
| ENSG00000140545 | 1.270892546 | 3.42E-05 | 0.000301937 | MFGE8 |  |
| ENSG00000114923 | -2.950709392 | 3.48E-05 | 0.000306469 | SLC4A3 |  |
| ENSG00000178445 | -3.283443778 | 3.50E-05 | 0.000308051 | GLDC |  |
| ENSG00000132002 | -0.904168489 | 3.50E-05 | 0.000308305 | DNAJB1 |  |
| ENSG00000100823 | 0.816784305 | 3.53E-05 | 0.000311119 | APEX1 |  |
| ENSG00000221643 | -1.994722965 | 3.54E-05 | 0.000311385 | SNORA77 |  |
| ENSG00000006451 | 1.425491492 | 3.56E-05 | 0.000312658 | RALA |  |
| ENSG00000102144 | 0.642065296 | 3.58E-05 | 0.000314276 | PGK1 |  |
| ENSG00000014824 | 0.981187238 | 3.58E-05 | 0.00031437 | SLC30A9 |  |
| ENSG00000140044 | -1.823147426 | 3.62E-05 | 0.000317478 | JDP2 |  |
| ENSG00000227500 | 1.085595735 | 3.62E-05 | 0.000317478 | SCAMP4 |  |
| ENSG00000164989 | -2.494502146 | 3.66E-05 | 0.000321189 | CCDC171 |  |
| ENSG00000080822 | 1.436908942 | 3.68E-05 | 0.00032221 | CLDND1 |  |
| ENSG00000122034 | -1.370382967 | 3.69E-05 | 0.000323324 | GTF3A |  |
| ENSG00000065328 | -1.54348468 | 3.73E-05 | 0.000326024 | MCM10 |  |
| ENSG00000131100 | 1.070189853 | 3.75E-05 | 0.000328199 | ATP6V1E1 |  |
| ENSG00000169599 | 1.75274532 | 3.76E-05 | 0.000328476 | NFU1 |  |
| ENSG00000184967 | 1.528904827 | 3.78E-05 | 0.000330178 | NOC4L |  |
| ENSG00000275662 | -2.16740907 | 3.79E-05 | 0.000330838 | SNORD112 |  |
| ENSG00000131236 | 0.780727041 | 3.81E-05 | 0.000332059 | CAP1 |  |
| ENSG00000107890 | -1.371500083 | 3.83E-05 | 0.000333884 | ANKRD26 |  |
| ENSG00000117410 | 0.984228025 | 3.92E-05 | 0.000341203 | ATP6V0B |  |
| ENSG00000117298 | 0.697237715 | 3.92E-05 | 0.000341569 | ECE1 |  |
| ENSG00000067369 | 0.795306972 | 3.93E-05 | 0.000342337 | TP53BP1 |  |
| ENSG00000012963 | 1.015853459 | 3.94E-05 | 0.000342841 | UBR7 |  |
| ENSG00000172757 | -0.548258626 | 4.06E-05 | 0.000353135 | CFL1 |  |
| ENSG00000140391 | 1.122210575 | 4.07E-05 | 0.000353493 | TSPAN3 |  |
| ENSG00000264864 | -2.93402468 | 4.09E-05 | 0.000354758 | MIR3613 |  |
| ENSG00000176771 | -2.363005294 | 4.10E-05 | 0.000355694 | NCKAP5 |  |
| ENSG00000184205 | -1.162573173 | 4.11E-05 | 0.000356556 | TSPYL2 |  |
| ENSG00000201823 | -2.110942556 | 4.13E-05 | 0.000358294 | SNORD48 |  |
| ENSG00000221241 | -0.677831446 | 4.14E-05 | 0.000358699 | SNORD88A |  |
| ENSG00000125352 | -2.240867499 | 4.17E-05 | 0.000360566 | RNF113A |  |
| ENSG00000146701 | 0.824014195 | 4.18E-05 | 0.000361545 | MDH2 |  |
| ENSG00000207611 | 1.332175772 | 4.20E-05 | 0.000363398 | MIR149 |  |
| ENSG00000228695 | -3.265497627 | 4.28E-05 | 0.000369477 | CES1P1 |  |
| ENSG00000148396 | 0.895923118 | 4.28E-05 | 0.000369525 | SEC16A |  |
| ENSG00000170561 | -0.884579626 | 4.30E-05 | 0.000370501 | IRX2 |  |
| ENSG00000162869 | -1.571587488 | 4.30E-05 | 0.000370501 | PPP1R21 |  |
| ENSG00000113282 | -0.667155377 | 4.30E-05 | 0.000370683 | CLINT1 |  |
| ENSG00000117020 | -0.939091683 | 4.33E-05 | 0.000372802 | AKT3 |  |
| ENSG00000222489 | -2.054141797 | 4.35E-05 | 0.000374462 | SNORA79B |  |
| ENSG00000110911 | 1.099821517 | 4.37E-05 | 0.000375894 | SLC11A2 |  |
| ENSG00000081026 | -1.582858696 | 4.38E-05 | 0.000376175 | MAGI3 |  |
| ENSG00000012660 | 0.844970825 | 4.39E-05 | 0.000377106 | ELOVL5 |  |
| ENSG00000148358 | 0.944612092 | 4.39E-05 | 0.000377354 | GPR107 |  |
| ENSG00000106443 | -0.991024758 | 4.43E-05 | 0.000379876 | PHF14 |  |
| ENSG00000124181 | 0.816507236 | 4.45E-05 | 0.000381402 | PLCG1 |  |
| ENSG00000105894 | -2.248507451 | 4.46E-05 | 0.00038235 | PTN |  |
| ENSG00000198794 | 2.711665896 | 4.51E-05 | 0.000386026 | SCAMP5 |  |
| ENSG00000226179 | -1.660080375 | 4.66E-05 | 0.000399226 | LINC00685 | |
| ENSG00000114988 | 1.771066265 | 4.68E-05 | 0.0004003 | LMAN2L |  |
| ENSG00000109758 | -3.244167751 | 4.70E-05 | 0.000402189 | HGFAC |  |
| ENSG00000196262 | 0.746525349 | 4.75E-05 | 0.00040559 | PPIA |  |
| ENSG00000080815 | 0.899753654 | 4.75E-05 | 0.00040559 | PSEN1 |  |
| ENSG00000132792 | -0.659576016 | 4.75E-05 | 0.00040559 | CTNNBL1 |  |
| ENSG00000104980 | -0.931044053 | 4.78E-05 | 0.000407942 | TIMM44 |  |
| ENSG00000177732 | -0.967754727 | 4.80E-05 | 0.000408866 | SOX12 |  |
| ENSG00000176390 | 0.557389411 | 4.80E-05 | 0.000408866 | CRLF3 |  |
| ENSG00000173457 | -0.944307104 | 4.82E-05 | 0.000410459 | PPP1R14B |  |
| ENSG00000199032 | -0.898373847 | 4.92E-05 | 0.000418551 | MIR425 |  |
| ENSG00000105176 | -1.266218424 | 5.03E-05 | 0.000427511 | URI1 |  |
| ENSG00000131504 | -0.627075143 | 5.14E-05 | 0.000436813 | DIAPH1 |  |
| ENSG00000068366 | 0.871540246 | 5.15E-05 | 0.000437336 | ACSL4 |  |
| ENSG00000169856 | -3.221089318 | 5.21E-05 | 0.000442051 | ONECUT1 |  |
| ENSG00000204525 | 0.502722826 | 5.22E-05 | 0.00044277 | HLA-C |  |
| ENSG00000144136 | 0.834164205 | 5.25E-05 | 0.000445257 | SLC20A1 |  |
| ENSG00000167515 | -1.693944955 | 5.29E-05 | 0.000448395 | TRAPPC2L |  |
| ENSG00000133065 | 1.246235884 | 5.35E-05 | 0.000453495 | SLC41A1 |  |
| ENSG00000113522 | 2.220311943 | 5.36E-05 | 0.000453851 | RAD50 |  |
| ENSG00000065308 | 0.722244528 | 5.39E-05 | 0.000455744 | TRAM2 |  |
| ENSG00000013588 | 0.774625236 | 5.43E-05 | 0.000459603 | GPRC5A |  |
| ENSG00000100941 | -0.763140027 | 5.44E-05 | 0.000459716 | PNN |  |
| ENSG00000278828 | 0.625479138 | 5.46E-05 | 0.000461088 | HIST1H3H |  |
| ENSG00000040531 | 1.526561973 | 5.47E-05 | 0.000461821 | CTNS |  |
| ENSG00000283128 | -1.937081956 | 5.48E-05 | 0.000462456 | AC009403.2 | |
| ENSG00000249921 | -3.213253882 | 5.52E-05 | 0.000465241 | AC034207.1 | |
| ENSG00000176170 | 1.416806616 | 5.53E-05 | 0.000465804 | SPHK1 |  |
| ENSG00000051825 | -1.127405444 | 5.55E-05 | 0.00046758 | MPHOSPH9 |  |
| ENSG00000001036 | 1.072958093 | 5.58E-05 | 0.000469404 | FUCA2 |  |
| ENSG00000197457 | -1.212102332 | 5.59E-05 | 0.000470079 | STMN3 |  |
| ENSG00000170889 | 0.689227093 | 5.59E-05 | 0.000470079 | RPS9 |  |
| ENSG00000106009 | 1.421391401 | 5.61E-05 | 0.000471794 | BRAT1 |  |
| ENSG00000154240 | -1.21996822 | 5.76E-05 | 0.000483364 | CEP112 |  |
| ENSG00000146918 | 0.709798099 | 5.81E-05 | 0.000487603 | NCAPG2 |  |
| ENSG00000155827 | -1.545388364 | 5.90E-05 | 0.000494856 | RNF20 |  |
| ENSG00000165006 | 1.297985455 | 5.98E-05 | 0.00050144 | UBAP1 |  |
| ENSG00000243521 | -3.199795741 | 6.06E-05 | 0.0005075 | RPL5P33 |  |
| ENSG00000179271 | -1.041984224 | 6.07E-05 | 0.000507925 | GADD45GIP1 | |
| ENSG00000162910 | 1.53427527 | 6.07E-05 | 0.000507925 | MRPL55 |  |
| ENSG00000120733 | 0.86648705 | 6.08E-05 | 0.000508193 | KDM3B |  |
| ENSG00000162545 | -1.863719732 | 6.12E-05 | 0.000511756 | CAMK2N1 |  |
| ENSG00000143771 | 1.140169578 | 6.18E-05 | 0.000515989 | CNIH4 |  |
| ENSG00000181523 | 1.379376697 | 6.22E-05 | 0.000519503 | SGSH |  |
| ENSG00000091583 | -2.877245571 | 6.24E-05 | 0.000520349 | APOH |  |
| ENSG00000197989 | -1.327263154 | 6.37E-05 | 0.000531224 | SNHG12 |  |
| ENSG00000133114 | -1.120291205 | 6.38E-05 | 0.000531875 | GPALPP1 |  |
| ENSG00000143933 | -0.627582773 | 6.38E-05 | 0.000531875 | CALM2 |  |
| ENSG00000127603 | -0.627078757 | 6.41E-05 | 0.000533609 | MACF1 |  |
| ENSG00000175029 | -1.258737216 | 6.42E-05 | 0.000533803 | CTBP2 |  |
| ENSG00000074695 | 0.900097085 | 6.45E-05 | 0.000536481 | LMAN1 |  |
| ENSG00000165494 | -1.032524146 | 6.46E-05 | 0.000536995 | PCF11 |  |
| ENSG00000129255 | 1.084185262 | 6.47E-05 | 0.000537689 | MPDU1 |  |
| ENSG00000137509 | 1.023964992 | 6.56E-05 | 0.00054431 | PRCP |  |
| ENSG00000128872 | -1.498556081 | 6.70E-05 | 0.000555837 | TMOD2 |  |
| ENSG00000139645 | 0.632603586 | 6.73E-05 | 0.000557892 | ANKRD52 |  |
| ENSG00000109920 | -1.137429278 | 6.75E-05 | 0.000559071 | FNBP4 |  |
| ENSG00000077721 | 1.041773226 | 6.78E-05 | 0.000561165 | UBE2A |  |
| ENSG00000102119 | -0.731465998 | 6.82E-05 | 0.000564208 | EMD |  |
| ENSG00000181896 | 0.682376165 | 6.83E-05 | 0.000565225 | ZNF101 |  |
| ENSG00000102580 | -0.866886004 | 6.88E-05 | 0.000568592 | DNAJC3 |  |
| ENSG00000116898 | -0.958529402 | 6.95E-05 | 0.000573752 | MRPS15 |  |
| ENSG00000134982 | -0.969136011 | 6.95E-05 | 0.000573752 | APC |  |
| ENSG00000103148 | 1.554975601 | 6.96E-05 | 0.000574565 | NPRL3 |  |
| ENSG00000124145 | 0.567851906 | 6.96E-05 | 0.000574565 | SDC4 |  |
| ENSG00000198695 | -0.481121055 | 6.97E-05 | 0.000574929 | MT-ND6 |  |
| ENSG00000101079 | 1.269775678 | 6.99E-05 | 0.00057607 | NDRG3 |  |
| ENSG00000107651 | 1.074734251 | 7.03E-05 | 0.000578651 | SEC23IP |  |
| ENSG00000144224 | -0.709691775 | 7.03E-05 | 0.000578651 | UBXN4 |  |
| ENSG00000007255 | 2.294529524 | 7.13E-05 | 0.000586359 | TRAPPC6A |  |
| ENSG00000059573 | 1.101097776 | 7.24E-05 | 0.000595453 | ALDH18A1 |  |
| ENSG00000132680 | -1.260871083 | 7.27E-05 | 0.000597732 | KHDC4 |  |
| ENSG00000178952 | 0.706581003 | 7.35E-05 | 0.000603224 | TUFM |  |
| ENSG00000122565 | -0.99239257 | 7.40E-05 | 0.00060761 | CBX3 |  |
| ENSG00000110075 | -0.708362756 | 7.42E-05 | 0.000608931 | PPP6R3 |  |
| ENSG00000185722 | 0.956083669 | 7.48E-05 | 0.000613453 | ANKFY1 |  |
| ENSG00000183087 | -0.617478571 | 7.49E-05 | 0.000613671 | GAS6 |  |
| ENSG00000153015 | -1.218878296 | 7.53E-05 | 0.000616475 | CWC27 |  |
| ENSG00000086758 | 0.482404622 | 7.56E-05 | 0.000618356 | HUWE1 |  |
| ENSG00000244405 | -1.862908833 | 7.58E-05 | 0.000619682 | ETV5 |  |
| ENSG00000067066 | -0.858422787 | 7.60E-05 | 0.000621615 | SP100 |  |
| ENSG00000100344 | 2.42231252 | 7.69E-05 | 0.000628513 | PNPLA3 |  |
| ENSG00000080618 | -3.164005251 | 7.70E-05 | 0.00062903 | CPB2 |  |
| ENSG00000207988 | -2.126413041 | 7.76E-05 | 0.000632891 | MIR576 |  |
| ENSG00000095002 | 0.942791223 | 7.82E-05 | 0.000638209 | MSH2 |  |
| ENSG00000117569 | -1.327951912 | 7.83E-05 | 0.000638641 | PTBP2 |  |
| ENSG00000182899 | 0.643781696 | 7.84E-05 | 0.000639125 | RPL35A |  |
| ENSG00000115275 | 1.279745803 | 7.89E-05 | 0.000642647 | MOGS |  |
| ENSG00000138942 | 1.487547114 | 7.90E-05 | 0.000642846 | RNF185 |  |
| ENSG00000100353 | -0.748011829 | 7.92E-05 | 0.000643929 | EIF3D |  |
| ENSG00000135052 | -0.798602476 | 7.99E-05 | 0.000649603 | GOLM1 |  |
| ENSG00000118898 | -2.080287268 | 8.05E-05 | 0.000653738 | PPL |  |
| ENSG00000105618 | -0.799148125 | 8.06E-05 | 0.000654236 | PRPF31 |  |
| ENSG00000164120 | -3.148192024 | 8.08E-05 | 0.000655778 | HPGD |  |
| ENSG00000173113 | 0.986564289 | 8.09E-05 | 0.000655778 | TRMT112 |  |
| ENSG00000115649 | 1.130663311 | 8.12E-05 | 0.000658116 | CNPPD1 |  |
| ENSG00000139116 | -1.809246907 | 8.18E-05 | 0.000663106 | KIF21A |  |
| ENSG00000205413 | -1.046833036 | 8.23E-05 | 0.000666079 | SAMD9 |  |
| ENSG00000109762 | -1.906206716 | 8.24E-05 | 0.000666624 | SNX25 |  |
| ENSG00000101407 | 1.027485084 | 8.27E-05 | 0.000669167 | TTI1 |  |
| ENSG00000004478 | -0.863496061 | 8.31E-05 | 0.00067156 | FKBP4 |  |
| ENSG00000233449 | -3.143207334 | 8.33E-05 | 0.000673209 | MTATP6P14 | |
| ENSG00000126870 | -1.520565989 | 8.35E-05 | 0.000674216 | WDR60 |  |
| ENSG00000072786 | -0.686546402 | 8.37E-05 | 0.000675166 | STK10 |  |
| ENSG00000130479 | 1.548661873 | 8.42E-05 | 0.000679288 | MAP1S |  |
| ENSG00000089693 | 0.821581894 | 8.45E-05 | 0.000681395 | MLF2 |  |
| ENSG00000165650 | -0.973315399 | 8.48E-05 | 0.000683255 | PDZD8 |  |
| ENSG00000143847 | -2.742139905 | 8.50E-05 | 0.000684746 | PPFIA4 |  |
| ENSG00000254999 | 1.311294114 | 8.54E-05 | 0.00068695 | BRK1 |  |
| ENSG00000125652 | 1.52104233 | 8.60E-05 | 0.000691364 | ALKBH7 |  |
| ENSG00000158864 | 0.787185293 | 8.60E-05 | 0.000691665 | NDUFS2 |  |
| ENSG00000221066 | -1.826917838 | 8.63E-05 | 0.00069319 | SNORD111 |  |
| ENSG00000054523 | -0.850925063 | 8.64E-05 | 0.000694128 | KIF1B |  |
| ENSG00000136824 | -0.692305919 | 8.65E-05 | 0.000694387 | SMC2 |  |
| ENSG00000067334 | -0.909690844 | 8.66E-05 | 0.000694923 | DNTTIP2 |  |
| ENSG00000112759 | 1.066807884 | 8.69E-05 | 0.000696944 | SLC29A1 |  |
| ENSG00000233045 | -3.137382587 | 8.72E-05 | 0.000698266 | AC097523.1 | |
| ENSG00000130175 | 0.483413982 | 8.74E-05 | 0.000699634 | PRKCSH |  |
| ENSG00000091656 | -0.908951446 | 8.78E-05 | 0.000702913 | ZFHX4 |  |
| ENSG00000041357 | 0.681791043 | 8.81E-05 | 0.000704897 | PSMA4 |  |
| ENSG00000105372 | -0.535080943 | 8.82E-05 | 0.000705426 | RPS19 |  |
| ENSG00000165244 | -1.29802858 | 8.86E-05 | 0.0007076 | ZNF367 |  |
| ENSG00000171566 | 0.937432199 | 8.86E-05 | 0.0007076 | PLRG1 |  |
| ENSG00000198984 | -2.167624044 | 8.89E-05 | 0.000709808 | MIR345 |  |
| ENSG00000232992 | -2.977639333 | 8.94E-05 | 0.000713157 | AHCYP4 |  |
| ENSG00000244176 | -3.132799891 | 8.95E-05 | 0.000713415 | AP003733.1 | |
| ENSG00000207110 | -1.915056608 | 8.97E-05 | 0.000714991 | RNU1-106P | |
| ENSG00000113013 | -0.502013139 | 9.00E-05 | 0.000716495 | HSPA9 |  |
| ENSG00000137055 | 1.227597534 | 9.05E-05 | 0.00072036 | PLAA |  |
| ENSG00000008710 | -1.518604175 | 9.10E-05 | 0.000724104 | PKD1 |  |
| ENSG00000138448 | 0.800312436 | 9.28E-05 | 0.000737644 | ITGAV |  |
| ENSG00000135423 | -3.126807241 | 9.31E-05 | 0.000739477 | GLS2 |  |
| ENSG00000089159 | 0.697868468 | 9.33E-05 | 0.000740903 | PXN |  |
| ENSG00000184500 | 1.615839974 | 9.34E-05 | 0.000740903 | PROS1 |  |
| ENSG00000109099 | 1.233780684 | 9.44E-05 | 0.00074849 | PMP22 |  |
| ENSG00000121073 | 0.947855331 | 9.46E-05 | 0.000749684 | SLC35B1 |  |
| ENSG00000152782 | -1.836572504 | 9.53E-05 | 0.000755128 | PANK1 |  |
| ENSG00000078403 | -1.045293222 | 9.65E-05 | 0.000763909 | MLLT10 |  |
| ENSG00000112511 | -1.35592956 | 9.96E-05 | 0.000788719 | PHF1 |  |
| ENSG00000106554 | -1.052478769 | 0.00010003 | 0.000791293 | CHCHD3 |  |
| ENSG00000124786 | 1.837299381 | 0.00010048 | 0.000794436 | SLC35B3 |  |
| ENSG00000189337 | -1.882209491 | 0.00010067 | 0.000795477 | KAZN |  |
| ENSG00000162434 | -0.682434103 | 0.00010085 | 0.000796487 | JAK1 |  |
| ENSG00000090661 | 2.014469374 | 0.00010276 | 0.00081111 | CERS4 |  |
| ENSG00000181045 | 1.196426146 | 0.00010294 | 0.000812068 | SLC26A11 |  |
| ENSG00000196323 | -1.486034374 | 0.00010398 | 0.000819825 | ZBTB44 |  |
| ENSG00000182272 | -1.348383956 | 0.00010413 | 0.000820612 | B4GALNT4 |  |
| ENSG00000011243 | -1.278205293 | 0.00010437 | 0.00082208 | AKAP8L |  |
| ENSG00000171467 | -1.064456223 | 0.0001057 | 0.000832027 | ZNF318 |  |
| ENSG00000171984 | 2.558187499 | 0.00010597 | 0.000833726 | SHLD1 |  |
| ENSG00000078061 | -1.006914624 | 0.00010629 | 0.000835763 | ARAF |  |
| ENSG00000107882 | 0.940625437 | 0.00010672 | 0.000838425 | SUFU |  |
| ENSG00000183207 | -0.656877529 | 0.00010674 | 0.000838425 | RUVBL2 |  |
| ENSG00000188343 | -1.633471303 | 0.00010702 | 0.000840155 | FAM92A |  |
| ENSG00000133872 | 1.139468808 | 0.0001088 | 0.00085345 | SARAF |  |
| ENSG00000111229 | 0.809897449 | 0.00010883 | 0.00085345 | ARPC3 |  |
| ENSG00000118855 | 1.231021761 | 0.00011171 | 0.000875544 | MFSD1 |  |
| ENSG00000183155 | 1.292487139 | 0.00011181 | 0.000875897 | RABIF |  |
| ENSG00000213614 | 1.332462746 | 0.0001121 | 0.000877692 | HEXA |  |
| ENSG00000056097 | -0.691570784 | 0.00011275 | 0.000882257 | ZFR |  |
| ENSG00000135503 | 1.629626108 | 0.00011365 | 0.000888862 | ACVR1B |  |
| ENSG00000154277 | 0.71987427 | 0.00011465 | 0.00089616 | UCHL1 |  |
| ENSG00000126088 | 0.849038213 | 0.00011518 | 0.000899404 | UROD |  |
| ENSG00000101346 | 0.889286782 | 0.00011519 | 0.000899404 | POFUT1 |  |
| ENSG00000126062 | 1.484455094 | 0.00011558 | 0.000901943 | TMEM115 |  |
| ENSG00000199753 | -0.580095842 | 0.00011582 | 0.000903325 | SNORD104 |  |
| ENSG00000176393 | 1.291552607 | 0.00011684 | 0.000910843 | RNPEP |  |
| ENSG00000234421 | -3.092693935 | 0.00011702 | 0.000911703 | SLC25A5P4 | |
| ENSG00000170522 | 0.952016313 | 0.00011726 | 0.000913132 | ELOVL6 |  |
| ENSG00000159403 | 1.010443237 | 0.0001174 | 0.00091369 | C1R |  |
| ENSG00000182220 | 0.828507898 | 0.00011762 | 0.000914933 | ATP6AP2 |  |
| ENSG00000116584 | -0.956188367 | 0.00011913 | 0.000926185 | ARHGEF2 |  |
| ENSG00000243156 | -1.010162887 | 0.00011928 | 0.000926814 | MICAL3 |  |
| ENSG00000146242 | 0.968433432 | 0.00011968 | 0.000929455 | TPBG |  |
| ENSG00000123643 | 1.523847555 | 0.00011986 | 0.000930341 | SLC36A1 |  |
| ENSG00000200807 | -2.305305468 | 0.00012002 | 0.000931076 | RNU1-32P |  |
| ENSG00000264049 | -2.360324583 | 0.0001201 | 0.000931185 | MIR4737 |  |
| ENSG00000160799 | -0.94206123 | 0.00012072 | 0.000935548 | CCDC12 |  |
| ENSG00000185591 | 0.963933295 | 0.0001219 | 0.000944153 | SP1 |  |
| ENSG00000102312 | 1.458572461 | 0.00012199 | 0.000944153 | PORCN |  |
| ENSG00000252316 | 2.221195744 | 0.00012203 | 0.000944153 | RNY4 |  |
| ENSG00000181830 | 1.846500858 | 0.00012252 | 0.000947448 | SLC35C1 |  |
| ENSG00000117632 | -0.463992278 | 0.00012277 | 0.000948828 | STMN1 |  |
| ENSG00000100883 | 0.919154805 | 0.00012408 | 0.000958258 | SRP54 |  |
| ENSG00000134759 | 0.965795996 | 0.00012412 | 0.000958258 | ELP2 |  |
| ENSG00000206755 | -2.930533657 | 0.00012538 | 0.000967501 | SNORA30 |  |
| ENSG00000126838 | -2.957432025 | 0.00012545 | 0.000967514 | PZP |  |
| ENSG00000206908 | -2.788992136 | 0.00012574 | 0.000969237 | RNU1-136P | |
| ENSG00000197043 | 0.587490997 | 0.00012671 | 0.000976182 | ANXA6 |  |
| ENSG00000147872 | 0.838828438 | 0.00012732 | 0.000980359 | PLIN2 |  |
| ENSG00000125037 | 1.138310977 | 0.00012749 | 0.000981152 | EMC3 |  |
| ENSG00000184178 | 2.187146495 | 0.00012775 | 0.000982625 | SCFD2 |  |
| ENSG00000143514 | -1.010145174 | 0.00012805 | 0.000984362 | TP53BP2 |  |
| ENSG00000099956 | -0.673363225 | 0.00012822 | 0.0009852 | SMARCB1 |  |
| ENSG00000025423 | -3.074210949 | 0.00012829 | 0.0009852 | HSD17B6 |  |
| ENSG00000111481 | 0.970813432 | 0.00012879 | 0.000988476 | COPZ1 |  |
| ENSG00000185515 | 1.011436474 | 0.00013015 | 0.000998438 | BRCC3 |  |
| ENSG00000005448 | 1.061695305 | 0.00013073 | 0.001002367 | WDR54 |  |
| ENSG00000134369 | -0.783954675 | 0.00013124 | 0.001005706 | NAV1 |  |
| ENSG00000100239 | -1.061126672 | 0.00013294 | 0.001018197 | PPP6R2 |  |
| ENSG00000139697 | -0.715917309 | 0.00013323 | 0.001019698 | SBNO1 |  |
| ENSG00000113140 | 0.433966174 | 0.00013328 | 0.001019698 | SPARC |  |
| ENSG00000140829 | -0.751016034 | 0.00013388 | 0.001023767 | DHX38 |  |
| ENSG00000212283 | -2.649967897 | 0.00013599 | 0.001039349 | SNORD89 |  |
| ENSG00000204632 | 1.846631456 | 0.00013749 | 0.001050264 | HLA-G |  |
| ENSG00000145414 | 1.601734162 | 0.0001385 | 0.001057443 | NAF1 |  |
| ENSG00000136940 | -1.212619074 | 0.0001394 | 0.001063746 | PDCL |  |
| ENSG00000127022 | -0.505114953 | 0.00014112 | 0.001075994 | CANX |  |
| ENSG00000255314 | -3.058990762 | 0.00014119 | 0.001075994 | AC024475.4 | |
| ENSG00000271687 | -2.917096062 | 0.00014123 | 0.001075994 | MTND5P10 |  |
| ENSG00000165168 | 1.90754963 | 0.00014227 | 0.001083372 | CYBB |  |
| ENSG00000125730 | 0.435770261 | 0.00014256 | 0.001084322 | C3 |  |
| ENSG00000132466 | -0.655938012 | 0.00014257 | 0.001084322 | ANKRD17 |  |
| ENSG00000183291 | 0.887389061 | 0.00014262 | 0.001084322 | SELENOF |  |
| ENSG00000136492 | 0.908561619 | 0.00014282 | 0.001084916 | BRIP1 |  |
| ENSG00000115355 | -0.773692736 | 0.00014285 | 0.001084916 | CCDC88A |  |
| ENSG00000066136 | -1.118646074 | 0.00014329 | 0.001087634 | NFYC |  |
| ENSG00000180340 | 0.978175192 | 0.00014409 | 0.001093169 | FZD2 |  |
| ENSG00000107796 | -2.909063564 | 0.000145 | 0.001099481 | ACTA2 |  |
| ENSG00000141002 | -0.891810121 | 0.00014588 | 0.00110537 | TCF25 |  |
| ENSG00000152492 | -1.124220029 | 0.00014593 | 0.00110537 | CCDC50 |  |
| ENSG00000198783 | -1.782981368 | 0.00014646 | 0.001108846 | ZNF830 |  |
| ENSG00000131013 | -1.128106286 | 0.00014663 | 0.001109492 | PPIL4 |  |
| ENSG00000189241 | 0.840015073 | 0.00014671 | 0.001109522 | TSPYL1 |  |
| ENSG00000186130 | 1.325169485 | 0.00014737 | 0.001113973 | ZBTB6 |  |
| ENSG00000111639 | 0.697146647 | 0.00014761 | 0.001115153 | MRPL51 |  |
| ENSG00000167711 | -3.046602593 | 0.00014852 | 0.001120851 | SERPINF2 |  |
| ENSG00000138823 | -3.046602593 | 0.00014852 | 0.001120851 | MTTP |  |
| ENSG00000145050 | -0.826826598 | 0.00015189 | 0.001145715 | MANF |  |
| ENSG00000158195 | -0.770335497 | 0.00015296 | 0.001153198 | WASF2 |  |
| ENSG00000100216 | -0.900934765 | 0.00015337 | 0.00115567 | TOMM22 |  |
| ENSG00000055950 | -1.295199671 | 0.00015425 | 0.001161706 | MRPL43 |  |
| ENSG00000047932 | 1.076006282 | 0.00015477 | 0.001165007 | GOPC |  |
| ENSG00000177156 | 0.620045417 | 0.00015553 | 0.001170089 | TALDO1 |  |
| ENSG00000133835 | 0.899350381 | 0.0001571 | 0.001181341 | HSD17B4 |  |
| ENSG00000105705 | -1.407939325 | 0.0001573 | 0.001182227 | SUGP1 |  |
| ENSG00000082701 | 0.733162338 | 0.00015801 | 0.001186706 | GSK3B |  |
| ENSG00000178467 | 1.14632993 | 0.00015806 | 0.001186706 | P4HTM |  |
| ENSG00000154380 | -0.602018142 | 0.00015858 | 0.001189968 | ENAH |  |
| ENSG00000162298 | 1.052695821 | 0.0001598 | 0.001198274 | SYVN1 |  |
| ENSG00000120370 | -1.698192365 | 0.00015985 | 0.001198274 | GORAB |  |
| ENSG00000008311 | -1.558572122 | 0.00016057 | 0.001203047 | AASS |  |
| ENSG00000100425 | -1.034613419 | 0.00016205 | 0.001213469 | BRD1 |  |
| ENSG00000182606 | -1.007717371 | 0.00016226 | 0.001214416 | TRAK1 |  |
| ENSG00000252481 | -1.992001941 | 0.00016345 | 0.001222643 | SCARNA13 |  |
| ENSG00000167118 | 1.015557226 | 0.00016353 | 0.001222643 | URM1 |  |
| ENSG00000136938 | -0.469120213 | 0.00016367 | 0.001222643 | ANP32B |  |
| ENSG00000262322 | -3.037698491 | 0.0001637 | 0.001222643 | MTND4P34 |  |
| ENSG00000130119 | 1.191164401 | 0.00016663 | 0.00124391 | GNL3L |  |
| ENSG00000110888 | -2.297415107 | 0.00016708 | 0.001246627 | CAPRIN2 |  |
| ENSG00000088812 | 0.690930186 | 0.00016778 | 0.001251213 | ATRN |  |
| ENSG00000188177 | -1.431444173 | 0.00017446 | 0.001300357 | ZC3H6 |  |
| ENSG00000196923 | -0.689737972 | 0.00017537 | 0.001305759 | PDLIM7 |  |
| ENSG00000221823 | 1.133218528 | 0.00017543 | 0.001305759 | PPP3R1 |  |
| ENSG00000163541 | 1.04874555 | 0.00017546 | 0.001305759 | SUCLG1 |  |
| ENSG00000055130 | -0.724588875 | 0.00017625 | 0.001310951 | CUL1 |  |
| ENSG00000151276 | -1.555125541 | 0.00017762 | 0.001320524 | MAGI1 |  |
| ENSG00000020922 | -1.315066537 | 0.00017934 | 0.001332594 | MRE11 |  |
| ENSG00000174915 | 1.034656241 | 0.0001798 | 0.001334905 | PTDSS2 |  |
| ENSG00000176014 | -0.531729236 | 0.00017984 | 0.001334905 | TUBB6 |  |
| ENSG00000131435 | 1.166227086 | 0.00018013 | 0.001336416 | PDLIM4 |  |
| ENSG00000092841 | -0.446047392 | 0.00018032 | 0.001337093 | MYL6 |  |
| ENSG00000162645 | -2.18677727 | 0.00018247 | 0.00135191 | GBP2 |  |
| ENSG00000110700 | 0.640432823 | 0.0001825 | 0.00135191 | RPS13 |  |
| ENSG00000222810 | -0.802078387 | 0.00018279 | 0.00135335 | RNU2-68P |  |
| ENSG00000066933 | -0.817314325 | 0.0001839 | 0.001360423 | MYO9A |  |
| ENSG00000168502 | -1.063740776 | 0.00018393 | 0.001360423 | MTCL1 |  |
| ENSG00000101443 | 1.97038455 | 0.00018438 | 0.001363068 | WFDC2 |  |
| ENSG00000083844 | 1.988238118 | 0.0001846 | 0.001363948 | ZNF264 |  |
| ENSG00000121691 | -2.242026624 | 0.00018522 | 0.001367822 | CAT |  |
| ENSG00000109063 | -2.417138566 | 0.00018545 | 0.001368868 | MYH3 |  |
| ENSG00000115514 | 1.105030408 | 0.00018674 | 0.001377642 | TXNDC9 |  |
| ENSG00000185222 | 1.101502916 | 0.00018905 | 0.001394018 | TCEAL9 |  |
| ENSG00000096070 | -1.02416629 | 0.00018919 | 0.001394303 | BRPF3 |  |
| ENSG00000166130 | -1.066206812 | 0.00018975 | 0.001397694 | IKBIP |  |
| ENSG00000136628 | 0.661297562 | 0.00019003 | 0.001398608 | EPRS |  |
| ENSG00000175782 | 1.434231988 | 0.00019006 | 0.001398608 | SLC35E3 |  |
| ENSG00000049246 | -1.023736223 | 0.00019177 | 0.001410435 | PER3 |  |
| ENSG00000103512 | 1.70383438 | 0.00019204 | 0.001411694 | NOMO1 |  |
| ENSG00000108861 | 1.019242619 | 0.00019323 | 0.001419738 | DUSP3 |  |
| ENSG00000138385 | -1.124649603 | 0.00019411 | 0.00142548 | SSB |  |
| ENSG00000182481 | 0.560179506 | 0.00019732 | 0.001448299 | KPNA2 |  |
| ENSG00000164031 | -1.114096362 | 0.0001986 | 0.001456937 | DNAJB14 |  |
| ENSG00000168944 | -1.03395956 | 0.00020084 | 0.00147269 | CEP120 |  |
| ENSG00000105404 | 1.496752915 | 0.00020274 | 0.00148582 | RABAC1 |  |
| ENSG00000075188 | 1.483705317 | 0.000203 | 0.001486991 | NUP37 |  |
| ENSG00000167657 | -0.862490782 | 0.00020338 | 0.001488986 | DAPK3 |  |
| ENSG00000132591 | 1.290211425 | 0.00020582 | 0.001506089 | ERAL1 |  |
| ENSG00000185499 | 2.077708756 | 0.0002079 | 0.001520562 | MUC1 |  |
| ENSG00000178927 | 1.283092828 | 0.00020906 | 0.001528241 | CYBC1 |  |
| ENSG00000202459 | -1.971674664 | 0.0002104 | 0.001537278 | RF00019 |  |
| ENSG00000143740 | -1.466843732 | 0.00021063 | 0.001538231 | SNAP47 |  |
| ENSG00000278135 | -1.222994391 | 0.00021103 | 0.001540313 | RF00004 |  |
| ENSG00000204356 | -0.881745786 | 0.00021209 | 0.001547319 | NELFE |  |
| ENSG00000116688 | 0.725126891 | 0.00021521 | 0.001569167 | MFN2 |  |
| ENSG00000252487 | 1.068034929 | 0.00021531 | 0.001569167 | RNY4P20 |  |
| ENSG00000164815 | 1.453378931 | 0.00021617 | 0.001574707 | ORC5 |  |
| ENSG00000113575 | 0.717375608 | 0.00021654 | 0.00157658 | PPP2CA |  |
| ENSG00000120727 | -0.843667961 | 0.000217 | 0.001579149 | PAIP2 |  |
| ENSG00000047188 | 0.929391025 | 0.00021775 | 0.001583819 | YTHDC2 |  |
| ENSG00000073792 | -1.061834606 | 0.00022017 | 0.001600602 | IGF2BP2 |  |
| ENSG00000100764 | -1.341659709 | 0.00022061 | 0.001602986 | PSMC1 |  |
| ENSG00000120708 | 0.440557155 | 0.00022097 | 0.001604786 | TGFBI |  |
| ENSG00000198055 | -0.981869115 | 0.00022507 | 0.001633756 | GRK6 |  |
| ENSG00000172775 | -0.814017347 | 0.00022584 | 0.001638529 | FAM192A |  |
| ENSG00000198818 | 1.249061351 | 0.00022652 | 0.001642622 | SFT2D1 |  |
| ENSG00000072518 | -1.136973093 | 0.00022859 | 0.001656775 | MARK2 |  |
| ENSG00000101017 | 2.620700748 | 0.00023063 | 0.001670747 | CD40 |  |
| ENSG00000140836 | -0.973322635 | 0.00023299 | 0.001686987 | ZFHX3 |  |
| ENSG00000099246 | 0.835367223 | 0.0002341 | 0.001694177 | RAB18 |  |
| ENSG00000121390 | -0.665532243 | 0.00023428 | 0.001694623 | PSPC1 |  |
| ENSG00000177084 | -0.699418199 | 0.00023508 | 0.001699593 | POLE |  |
| ENSG00000149923 | 0.72911638 | 0.00023566 | 0.00170293 | PPP4C |  |
| ENSG00000185567 | 0.645406326 | 0.00023724 | 0.001713468 | AHNAK2 |  |
| ENSG00000115457 | -2.444169111 | 0.00023852 | 0.001721828 | IGFBP2 |  |
| ENSG00000078140 | 0.883619095 | 0.00023873 | 0.001722541 | UBE2K |  |
| ENSG00000197771 | 0.69819021 | 0.0002389 | 0.001722899 | MCMBP |  |
| ENSG00000125870 | 1.114136236 | 0.00023998 | 0.001729767 | SNRPB2 |  |
| ENSG00000274210 | -0.737324782 | 0.000242 | 0.001743066 | RF00003 |  |
| ENSG00000081087 | 1.098331115 | 0.00024206 | 0.001743066 | OSTM1 |  |
| ENSG00000235173 | 1.443841995 | 0.00024643 | 0.001773367 | HGH1 |  |
| ENSG00000105135 | 1.005471093 | 0.00024653 | 0.001773367 | ILVBL |  |
| ENSG00000065882 | -1.184219948 | 0.00024664 | 0.001773367 | TBC1D1 |  |
| ENSG00000196562 | -1.53117595 | 0.00024871 | 0.001787401 | SULF2 |  |
| ENSG00000198836 | -0.776098673 | 0.00024951 | 0.001792253 | OPA1 |  |
| ENSG00000130821 | 1.216444236 | 0.00024964 | 0.001792296 | SLC6A8 |  |
| ENSG00000198888 | -0.454882822 | 0.0002504 | 0.001796816 | MT-ND1 |  |
| ENSG00000269119 | -1.772620435 | 0.00025131 | 0.001802498 | HNRNPA1P52 | |
| ENSG00000168872 | 1.748086788 | 0.0002525 | 0.001810085 | DDX19A |  |
| ENSG00000128245 | 0.62761118 | 0.00025335 | 0.001815304 | YWHAH |  |
| ENSG00000252682 | -1.266933512 | 0.00025521 | 0.001827701 | RF00273 |  |
| ENSG00000139746 | -0.944604692 | 0.00025585 | 0.001831377 | RBM26 |  |
| ENSG00000241973 | 0.812015928 | 0.00025772 | 0.001843835 | PI4KA |  |
| ENSG00000101367 | -0.462882634 | 0.00026066 | 0.001863953 | MAPRE1 |  |
| ENSG00000160633 | -0.572811527 | 0.00026278 | 0.001878224 | SAFB |  |
| ENSG00000188157 | 0.558254979 | 0.00026339 | 0.001881668 | AGRN |  |
| ENSG00000163017 | -1.440171789 | 0.00026475 | 0.001890412 | ACTG2 |  |
| ENSG00000125484 | 0.901192775 | 0.00026831 | 0.001914862 | GTF3C4 |  |
| ENSG00000223001 | -2.798726901 | 0.00026958 | 0.001923028 | RNU2-61P |  |
| ENSG00000104853 | 0.517492674 | 0.00027176 | 0.001937631 | CLPTM1 |  |
| ENSG00000119408 | -0.72665655 | 0.00027323 | 0.001947143 | NEK6 |  |
| ENSG00000136770 | -1.065543088 | 0.00027581 | 0.001964523 | DNAJC1 |  |
| ENSG00000138587 | -1.658631374 | 0.00027613 | 0.001965819 | MNS1 |  |
| ENSG00000183624 | 1.1986385 | 0.00027659 | 0.001967469 | HMCES |  |
| ENSG00000204392 | 1.479323611 | 0.00027663 | 0.001967469 | LSM2 |  |
| ENSG00000215930 | -2.439079812 | 0.00027737 | 0.001971783 | MIR942 |  |
| ENSG00000168374 | 0.81280465 | 0.00028042 | 0.001992431 | ARF4 |  |
| ENSG00000197712 | -0.794549461 | 0.00028245 | 0.002005859 | FAM114A1 |  |
| ENSG00000133706 | 0.654882796 | 0.00028323 | 0.002010445 | LARS |  |
| ENSG00000283174 | -2.93643399 | 0.00028393 | 0.002014442 | AL590396.3 | |
| ENSG00000206989 | -2.216253445 | 0.00028571 | 0.002025442 | SNORD63 |  |
| ENSG00000280404 | -2.930635308 | 0.0002859 | 0.002025442 | AC005086.4 | |
| ENSG00000180573 | 0.5899305 | 0.0002859 | 0.002025442 | HIST1H2AC | |
| ENSG00000169439 | -1.703464978 | 0.00028638 | 0.002027811 | SDC2 |  |
| ENSG00000130508 | 0.9554412 | 0.00029019 | 0.002053612 | PXDN |  |
| ENSG00000108854 | -0.742355319 | 0.00029031 | 0.002053612 | SMURF2 |  |
| ENSG00000131470 | -1.406855725 | 0.00029193 | 0.002063539 | PSMC3IP |  |
| ENSG00000010327 | -2.927053592 | 0.00029202 | 0.002063539 | STAB1 |  |
| ENSG00000116127 | -1.134407403 | 0.00029214 | 0.002063539 | ALMS1 |  |
| ENSG00000137404 | 1.268414914 | 0.00029257 | 0.002065512 | NRM |  |
| ENSG00000178977 | -0.579123481 | 0.0002927 | 0.002065512 | LINC00324 | |
| ENSG00000254931 | -2.802462538 | 0.00029336 | 0.002069127 | MTATP6P15 | |
| ENSG00000197355 | 1.256594203 | 0.00029599 | 0.002086645 | UAP1L1 |  |
| ENSG00000259921 | -2.03592512 | 0.00029702 | 0.002092937 | AC022819.1 | |
| ENSG00000141298 | -1.082335145 | 0.00029932 | 0.002108099 | SSH2 |  |
| ENSG00000178691 | -0.780527304 | 0.00030595 | 0.002153697 | SUZ12 |  |
| ENSG00000151327 | -1.203740545 | 0.00030648 | 0.00215638 | FAM177A1 |  |
| ENSG00000011566 | -1.362548727 | 0.00031299 | 0.002201164 | MAP4K3 |  |
| ENSG00000160445 | 1.226235259 | 0.00031345 | 0.002203267 | ZER1 |  |
| ENSG00000071462 | 0.854694648 | 0.00031627 | 0.002222012 | BUD23 |  |
| ENSG00000145536 | -2.771738019 | 0.00031665 | 0.002223655 | ADAMTS16 |  |
| ENSG00000117308 | 0.973458831 | 0.00031714 | 0.002225993 | GALE |  |
| ENSG00000108654 | -0.469300023 | 0.00031777 | 0.002229334 | DDX5 |  |
| ENSG00000249915 | 0.76682927 | 0.0003194 | 0.00223965 | PDCD6 |  |
| ENSG00000167768 | -2.91037291 | 0.00032137 | 0.002252431 | KRT1 |  |
| ENSG00000006125 | 0.579578035 | 0.00032386 | 0.00226875 | AP2B1 |  |
| ENSG00000102362 | -1.815976445 | 0.00032501 | 0.002275708 | SYTL4 |  |
| ENSG00000137807 | -0.537344469 | 0.00032582 | 0.002280284 | KIF23 |  |
| ENSG00000122783 | 1.750410278 | 0.00032683 | 0.002286231 | CYREN |  |
| ENSG00000171155 | 1.445000197 | 0.00032735 | 0.002288753 | C1GALT1C1 | |
| ENSG00000109066 | 1.084601843 | 0.00032808 | 0.002292717 | TMEM104 |  |
| ENSG00000100387 | 0.783332746 | 0.00033137 | 0.002314607 | RBX1 |  |
| ENSG00000100519 | 0.848366496 | 0.00033218 | 0.00231918 | PSMC6 |  |
| ENSG00000198858 | 1.362194338 | 0.00033429 | 0.002332739 | R3HDM4 |  |
| ENSG00000104805 | 0.736393534 | 0.00033476 | 0.002334883 | NUCB1 |  |
| ENSG00000143748 | -1.033400049 | 0.00033554 | 0.002339221 | NVL |  |
| ENSG00000100243 | 0.616845479 | 0.00033679 | 0.002346787 | CYB5R3 |  |
| ENSG00000117707 | -2.653992124 | 0.00033957 | 0.002365061 | PROX1 |  |
| ENSG00000063854 | -0.929898261 | 0.00034243 | 0.002383832 | HAGH |  |
| ENSG00000054118 | -0.579640108 | 0.00034314 | 0.002386623 | THRAP3 |  |
| ENSG00000085733 | -0.52288346 | 0.00034329 | 0.002386623 | CTTN |  |
| ENSG00000134575 | 1.293480849 | 0.00034333 | 0.002386623 | ACP2 |  |
| ENSG00000135842 | -0.626150021 | 0.00034457 | 0.002394112 | FAM129A |  |
| ENSG00000163634 | -1.033288989 | 0.00034492 | 0.002395403 | THOC7 |  |
| ENSG00000100401 | -0.553960116 | 0.00034654 | 0.002405475 | RANGAP1 |  |
| ENSG00000207714 | -1.191522066 | 0.00034721 | 0.00240896 | MIR584 |  |
| ENSG00000081923 | -0.662475891 | 0.00034832 | 0.002415468 | ATP8B1 |  |
| ENSG00000011485 | -0.645233519 | 0.00034866 | 0.002416692 | PPP5C |  |
| ENSG00000101126 | 0.809097637 | 0.00034955 | 0.002421723 | ADNP |  |
| ENSG00000183283 | 0.876822381 | 0.00035025 | 0.002425399 | DAZAP2 |  |
| ENSG00000157510 | -1.453779173 | 0.00035121 | 0.002430869 | AFAP1L1 |  |
| ENSG00000100731 | 0.789452238 | 0.00035743 | 0.002472733 | PCNX1 |  |
| ENSG00000117616 | -1.343945782 | 0.00035902 | 0.002482571 | RSRP1 |  |
| ENSG00000183856 | -0.631737095 | 0.00035992 | 0.002487586 | IQGAP3 |  |
| ENSG00000067225 | 0.492883992 | 0.00036074 | 0.002490861 | PKM |  |
| ENSG00000100664 | -0.558335386 | 0.00036074 | 0.002490861 | EIF5 |  |
| ENSG00000212163 | -2.065366755 | 0.00036262 | 0.002502668 | SNORD91A |  |
| ENSG00000168528 | 1.835384136 | 0.00036612 | 0.002525632 | SERINC2 |  |
| ENSG00000170445 | 0.535727775 | 0.00036652 | 0.00252717 | HARS |  |
| ENSG00000064313 | 1.065530395 | 0.00036706 | 0.002529658 | TAF2 |  |
| ENSG00000106605 | 1.015616625 | 0.00036734 | 0.002530361 | BLVRA |  |
| ENSG00000201289 | -2.418407195 | 0.0003687 | 0.002538571 | RN7SKP76 |  |
| ENSG00000104763 | 1.007283769 | 0.00037273 | 0.002565051 | ASAH1 |  |
| ENSG00000083168 | -0.835615511 | 0.00037386 | 0.00257162 | KAT6A |  |
| ENSG00000162409 | -2.424847343 | 0.00037434 | 0.002573675 | PRKAA2 |  |
| ENSG00000171960 | 1.256062612 | 0.00037483 | 0.002575836 | PPIH |  |
| ENSG00000174501 | -2.221299769 | 0.00037546 | 0.002578968 | ANKRD36C |  |
| ENSG00000111321 | 0.984342563 | 0.00037664 | 0.002585812 | LTBR |  |
| ENSG00000118046 | -1.012947485 | 0.00037709 | 0.002587683 | STK11 |  |
| ENSG00000135870 | -1.245956835 | 0.00037851 | 0.002596197 | RC3H1 |  |
| ENSG00000117362 | 0.798803301 | 0.00038 | 0.002604957 | APH1A |  |
| ENSG00000106628 | 0.772770419 | 0.00038015 | 0.002604957 | POLD2 |  |
| ENSG00000177885 | -0.804817021 | 0.00038187 | 0.002615528 | GRB2 |  |
| ENSG00000105701 | -0.726991609 | 0.0003852 | 0.002637036 | FKBP8 |  |
| ENSG00000162734 | 0.59180659 | 0.00038613 | 0.002641486 | PEA15 |  |
| ENSG00000199884 | -1.200846537 | 0.00038621 | 0.002641486 | RF00019 |  |
| ENSG00000107862 | 0.66440363 | 0.00038704 | 0.002645929 | GBF1 |  |
| ENSG00000136643 | -0.903786229 | 0.00038853 | 0.002654831 | RPS6KC1 |  |
| ENSG00000125648 | -1.608698432 | 0.00038903 | 0.002656985 | SLC25A23 |  |
| ENSG00000130164 | 0.492976436 | 0.00038944 | 0.002658502 | LDLR |  |
| ENSG00000082641 | 0.538379753 | 0.00038964 | 0.002658657 | NFE2L1 |  |
| ENSG00000198732 | -2.872902974 | 0.00039033 | 0.002662107 | SMOC1 |  |
| ENSG00000150093 | 0.481573884 | 0.00039423 | 0.002687401 | ITGB1 |  |
| ENSG00000126218 | -2.874481293 | 0.00039657 | 0.002701031 | F10 |  |
| ENSG00000207619 | -2.746971048 | 0.00039668 | 0.002701031 | MIR585 |  |
| ENSG00000234981 | -2.870510016 | 0.00039679 | 0.002701031 | AC244034.1 | |
| ENSG00000130309 | 0.732930985 | 0.00039857 | 0.002711907 | COLGALT1 |  |
| ENSG00000160214 | -0.78324596 | 0.00039956 | 0.002717343 | RRP1 |  |
| ENSG00000184347 | -1.322266225 | 0.00040045 | 0.002722085 | SLIT3 |  |
| ENSG00000075413 | -0.945854848 | 0.0004007 | 0.002722539 | MARK3 |  |
| ENSG00000124444 | 2.880415772 | 0.00040184 | 0.002728995 | ZNF576 |  |
| ENSG00000239437 | -2.871750611 | 0.00040205 | 0.002729136 | RN7SL752P | |
| ENSG00000095539 | -2.742822787 | 0.0004051 | 0.002748546 | SEMA4G |  |
| ENSG00000115540 | 1.46966232 | 0.00040679 | 0.002758737 | MOB4 |  |
| ENSG00000182979 | -1.240617133 | 0.00040741 | 0.002761613 | MTA1 |  |
| ENSG00000214071 | -2.865392375 | 0.00040855 | 0.00276809 | BX842568.1 | |
| ENSG00000172985 | -2.037444369 | 0.00040915 | 0.002770818 | SH3RF3 |  |
| ENSG00000182551 | -0.762027427 | 0.00040942 | 0.002770818 | ADI1 |  |
| ENSG00000128563 | -1.142377429 | 0.00040953 | 0.002770818 | PRKRIP1 |  |
| ENSG00000005893 | 0.773779818 | 0.00041082 | 0.002778212 | LAMP2 |  |
| ENSG00000073060 | 0.952509366 | 0.00041221 | 0.002786347 | SCARB1 |  |
| ENSG00000114978 | 0.70122249 | 0.00041381 | 0.002795851 | MOB1A |  |
| ENSG00000187955 | -2.861815259 | 0.00041507 | 0.002803045 | COL14A1 |  |
| ENSG00000104522 | -0.930098502 | 0.00041542 | 0.002804097 | TSTA3 |  |
| ENSG00000168497 | -2.745674616 | 0.00041744 | 0.002816419 | CAVIN2 |  |
| ENSG00000130560 | -0.960753289 | 0.0004208 | 0.002837752 | UBAC1 |  |
| ENSG00000137965 | 1.037353135 | 0.00042255 | 0.002848197 | IFI44 |  |
| ENSG00000116809 | -1.252132226 | 0.00042395 | 0.002856322 | ZBTB17 |  |
| ENSG00000073614 | -0.634749333 | 0.00042519 | 0.002863359 | KDM5A |  |
| ENSG00000166825 | 0.955045364 | 0.00042631 | 0.002869555 | ANPEP |  |
| ENSG00000179532 | -2.723169 | 0.00043078 | 0.00289826 | DNHD1 |  |
| ENSG00000196670 | -1.655938517 | 0.00043357 | 0.002915676 | ZFP62 |  |
| ENSG00000196418 | -1.34309329 | 0.00043462 | 0.002921384 | ZNF124 |  |
| ENSG00000149554 | 1.232509902 | 0.00043521 | 0.002924028 | CHEK1 |  |
| ENSG00000178573 | -2.613790754 | 0.00043566 | 0.002925681 | MAF |  |
| ENSG00000102804 | 0.946677483 | 0.00044576 | 0.002992103 | TSC22D1 |  |
| ENSG00000141338 | -2.84822214 | 0.00044874 | 0.003010685 | ABCA8 |  |
| ENSG00000116062 | 0.712592194 | 0.00045054 | 0.003021377 | MSH6 |  |
| ENSG00000139684 | 0.90567635 | 0.00045214 | 0.003030728 | ESD |  |
| ENSG00000078808 | -0.631693468 | 0.00045291 | 0.003034423 | SDF4 |  |
| ENSG00000133112 | -0.690595847 | 0.00045621 | 0.003055145 | TPT1 |  |
| ENSG00000266412 | 0.88611961 | 0.00045665 | 0.003056692 | NCOA4 |  |
| ENSG00000084734 | -2.850456508 | 0.00045861 | 0.003068357 | GCKR |  |
| ENSG00000163872 | -1.124281049 | 0.00046117 | 0.003084077 | YEATS2 |  |
| ENSG00000167110 | -0.756371472 | 0.00046169 | 0.003086092 | GOLGA2 |  |
| ENSG00000109133 | 0.676635797 | 0.00046289 | 0.003092729 | TMEM33 |  |
| ENSG00000184708 | -1.213792969 | 0.00046385 | 0.00309769 | EIF4ENIF1 | |
| ENSG00000273674 | -1.465906332 | 0.00046664 | 0.003114862 | AC021752.1 | |
| ENSG00000165119 | 0.485880973 | 0.00046816 | 0.003123593 | HNRNPK |  |
| ENSG00000168710 | 0.819025445 | 0.00046982 | 0.003133223 | AHCYL1 |  |
| ENSG00000107738 | 2.610831517 | 0.00047114 | 0.003140549 | VSIR |  |
| ENSG00000114857 | -0.955493805 | 0.00047344 | 0.003154417 | NKTR |  |
| ENSG00000130723 | -0.637484834 | 0.0004743 | 0.003157328 | PRRC2B |  |
| ENSG00000188612 | 0.60730113 | 0.00047431 | 0.003157328 | SUMO2 |  |
| ENSG00000078687 | -2.704895222 | 0.00047461 | 0.003157845 | TNRC6C |  |
| ENSG00000104859 | -1.111630202 | 0.00047893 | 0.003185124 | CLASRP |  |
| ENSG00000144746 | 0.848512389 | 0.00047921 | 0.003185549 | ARL6IP5 |  |
| ENSG00000143434 | 1.658962758 | 0.00048076 | 0.003194405 | SEMA6C |  |
| ENSG00000105518 | 1.291874455 | 0.0004881 | 0.003241064 | TMEM205 |  |
| ENSG00000152583 | -2.182924594 | 0.00048823 | 0.003241064 | SPARCL1 |  |
| ENSG00000072210 | 1.484323848 | 0.00050254 | 0.00333449 | ALDH3A2 |  |
| ENSG00000152556 | 0.835211498 | 0.00050342 | 0.003338765 | PFKM |  |
| ENSG00000165688 | 0.789658205 | 0.00050704 | 0.003361272 | PMPCA |  |
| ENSG00000228672 | 1.919965153 | 0.00051025 | 0.003381023 | PROB1 |  |
| ENSG00000257923 | -0.731696301 | 0.00051071 | 0.003381444 | CUX1 |  |
| ENSG00000152270 | -2.823944588 | 0.00051079 | 0.003381444 | PDE3B |  |
| ENSG00000138449 | -2.824508531 | 0.0005129 | 0.003393857 | SLC40A1 |  |
| ENSG00000226960 | -2.822791076 | 0.00051741 | 0.003422163 | MTCO1P21 |  |
| ENSG00000225136 | -2.559452801 | 0.00051821 | 0.003425846 | PGBD4P5 |  |
| ENSG00000128272 | 0.69709149 | 0.00051913 | 0.003430388 | ATF4 |  |
| ENSG00000201059 | -2.827240109 | 0.00051979 | 0.003433202 | RNA5SP336 | |
| ENSG00000138757 | -0.538064682 | 0.0005237 | 0.003457416 | G3BP2 |  |
| ENSG00000184208 | -2.822526376 | 0.0005292 | 0.003492134 | C22orf46 |  |
| ENSG00000100311 | -2.557212181 | 0.0005306 | 0.00349901 | PDGFB |  |
| ENSG00000038274 | 0.860479308 | 0.00053073 | 0.00349901 | MAT2B |  |
| ENSG00000144848 | -0.597863832 | 0.00053208 | 0.00350636 | ATG3 |  |
| ENSG00000183287 | -0.785873515 | 0.00053382 | 0.003516225 | CCBE1 |  |
| ENSG00000114573 | 1.054632556 | 0.0005352 | 0.003523696 | ATP6V1A |  |
| ENSG00000163249 | -1.383128451 | 0.00053672 | 0.003532076 | CCNYL1 |  |
| ENSG00000141582 | -1.738441866 | 0.0005392 | 0.00354585 | CBX4 |  |
| ENSG00000119638 | 0.972375553 | 0.0005393 | 0.00354585 | NEK9 |  |
| ENSG00000102393 | 1.422950307 | 0.00054005 | 0.003548144 | GLA |  |
| ENSG00000013523 | 1.866734453 | 0.00054014 | 0.003548144 | ANGEL1 |  |
| ENSG00000171365 | 1.453357656 | 0.00054263 | 0.003562873 | CLCN5 |  |
| ENSG00000140740 | 0.837932086 | 0.00054502 | 0.003576955 | UQCRC2 |  |
| ENSG00000163961 | -1.03749651 | 0.00054688 | 0.003587148 | RNF168 |  |
| ENSG00000147383 | 0.915799476 | 0.00054707 | 0.003587148 | NSDHL |  |
| ENSG00000099624 | 1.046640649 | 0.0005478 | 0.003590279 | ATP5F1D |  |
| ENSG00000140264 | -1.047311842 | 0.00055013 | 0.003603035 | SERF2 |  |
| ENSG00000153774 | -0.97615274 | 0.00055025 | 0.003603035 | CFDP1 |  |
| ENSG00000214286 | -2.806983317 | 0.00055297 | 0.003619245 | PDCL3P3 |  |
| ENSG00000201098 | 0.753274962 | 0.00055568 | 0.003635321 | RNY1 |  |
| ENSG00000244230 | -1.582384693 | 0.00055654 | 0.003639329 | RN7SL151P | |
| ENSG00000112651 | 1.292896321 | 0.00055749 | 0.003643847 | MRPL2 |  |
| ENSG00000278705 | 0.527247623 | 0.00055844 | 0.003648422 | HIST1H4B |  |
| ENSG00000115159 | 1.374740329 | 0.0005593 | 0.003652411 | GPD2 |  |
| ENSG00000162736 | 0.810969576 | 0.00056199 | 0.003667871 | NCSTN |  |
| ENSG00000122406 | -0.60163434 | 0.00056218 | 0.003667871 | RPL5 |  |
| ENSG00000112282 | 1.012923442 | 0.0005681 | 0.003704341 | MED23 |  |
| ENSG00000177106 | -0.802611381 | 0.00056828 | 0.003704341 | EPS8L2 |  |
| ENSG00000154262 | -2.559504174 | 0.00056873 | 0.003705616 | ABCA6 |  |
| ENSG00000130741 | 0.839644621 | 0.00056903 | 0.003705881 | EIF2S3 |  |
| ENSG00000034693 | 1.426290688 | 0.00057294 | 0.003729645 | PEX3 |  |
| ENSG00000223125 | -1.451071411 | 0.00057346 | 0.003731365 | RNU2-32P |  |
| ENSG00000145337 | -1.273850631 | 0.00057455 | 0.003736797 | PYURF |  |
| ENSG00000136379 | -2.453508798 | 0.00057575 | 0.003742883 | ABHD17C |  |
| ENSG00000163807 | -1.12123252 | 0.00057855 | 0.003759375 | KIAA1143 |  |
| ENSG00000273136 | -2.537202242 | 0.00058171 | 0.003778243 | NBPF26 |  |
| ENSG00000225947 | -2.800168492 | 0.00058452 | 0.003794784 | AL596448.1 | |
| ENSG00000275700 | -0.717256533 | 0.00058488 | 0.003795444 | AATF |  |
| ENSG00000129245 | 0.826211575 | 0.00058964 | 0.003824552 | FXR2 |  |
| ENSG00000114491 | 1.086096739 | 0.00059294 | 0.00384424 | UMPS |  |
| ENSG00000164733 | 0.435687417 | 0.00059647 | 0.003865424 | CTSB |  |
| ENSG00000148110 | 0.891509289 | 0.00059852 | 0.003875795 | MFSD14B |  |
| ENSG00000095485 | 1.231768676 | 0.00059861 | 0.003875795 | CWF19L1 |  |
| ENSG00000113924 | -1.948260187 | 0.00059893 | 0.003876158 | HGD |  |
| ENSG00000134057 | 0.489408443 | 0.00060667 | 0.003924451 | CCNB1 |  |
| ENSG00000283170 | -1.597586054 | 0.0006072 | 0.003926158 | MIR382 |  |
| ENSG00000199879 | -1.534255538 | 0.00061082 | 0.003947772 | RNU1-120P | |
| ENSG00000112992 | 1.1529412 | 0.00061302 | 0.003960232 | NNT |  |
| ENSG00000166396 | 2.570440646 | 0.0006141 | 0.003965432 | SERPINB7 |  |
| ENSG00000127948 | 0.75698532 | 0.00061492 | 0.003968961 | POR |  |
| ENSG00000244503 | -2.791864868 | 0.00061646 | 0.003977093 | AC108751.5 | |
| ENSG00000163660 | -1.041936413 | 0.00061937 | 0.003994133 | CCNL1 |  |
| ENSG00000173846 | -1.781907086 | 0.00062044 | 0.003999205 | PLK3 |  |
| ENSG00000169252 | 1.256594044 | 0.00062165 | 0.004005239 | ADRB2 |  |
| ENSG00000112337 | -2.783498803 | 0.00062501 | 0.004023831 | SLC17A2 |  |
| ENSG00000118246 | 1.28761788 | 0.00062509 | 0.004023831 | FASTKD2 |  |
| ENSG00000156398 | -1.852333128 | 0.00062687 | 0.00403345 | SFXN2 |  |
| ENSG00000071564 | -1.065140672 | 0.00062907 | 0.004045813 | TCF3 |  |
| ENSG00000134987 | 0.773703573 | 0.00063139 | 0.004058968 | WDR36 |  |
| ENSG00000129474 | 0.653953138 | 0.00063607 | 0.004085818 | AJUBA |  |
| ENSG00000170439 | -2.782842125 | 0.00063614 | 0.004085818 | METTL7B |  |
| ENSG00000116580 | -1.400896379 | 0.00063693 | 0.004089093 | GON4L |  |
| ENSG00000169241 | 0.985583574 | 0.00064163 | 0.004117444 | SLC50A1 |  |
| ENSG00000143379 | -1.021128257 | 0.00064479 | 0.004135427 | SETDB1 |  |
| ENSG00000196465 | -1.276832907 | 0.00064519 | 0.004135427 | MYL6B |  |
| ENSG00000115107 | 1.630642757 | 0.00064529 | 0.004135427 | STEAP3 |  |
| ENSG00000184838 | -2.528447001 | 0.00064565 | 0.004135883 | PRR16 |  |
| ENSG00000229774 | -1.692792084 | 0.00064605 | 0.004136603 | AC018866.1 | |
| ENSG00000001630 | 1.868445479 | 0.00065218 | 0.00417398 | CYP51A1 |  |
| ENSG00000269556 | 1.282439425 | 0.00065478 | 0.004188799 | TMEM185A |  |
| ENSG00000128524 | 1.526153065 | 0.00065777 | 0.004206073 | ATP6V1F |  |
| ENSG00000008018 | 0.503772614 | 0.00065807 | 0.004206114 | PSMB1 |  |
| ENSG00000166888 | 0.77781974 | 0.00065884 | 0.004208119 | STAT6 |  |
| ENSG00000174446 | -2.237235927 | 0.00065897 | 0.004208119 | SNAPC5 |  |
| ENSG00000103657 | 0.766252929 | 0.00066024 | 0.004214407 | HERC1 |  |
| ENSG00000200792 | -0.991899784 | 0.00067691 | 0.004318876 | SNORA80A |  |
| ENSG00000152133 | -1.034940017 | 0.00068084 | 0.004342062 | GPATCH11 |  |
| ENSG00000138162 | -0.933380238 | 0.00068172 | 0.004345757 | TACC2 |  |
| ENSG00000117597 | -0.966432996 | 0.00068648 | 0.004374136 | UTP25 |  |
| ENSG00000138430 | 1.195104237 | 0.00068797 | 0.004381703 | OLA1 |  |
| ENSG00000182199 | 0.705449468 | 0.00069208 | 0.004405922 | SHMT2 |  |
| ENSG00000168002 | 0.979844356 | 0.00069483 | 0.004421509 | POLR2G |  |
| ENSG00000147535 | 1.883547278 | 0.00069531 | 0.004422612 | PLPP5 |  |
| ENSG00000200176 | -1.266641159 | 0.00069671 | 0.004429581 | RNU1-19P |  |
| ENSG00000138085 | 0.97113874 | 0.00069754 | 0.00443288 | ATRAID |  |
| ENSG00000205336 | 1.02076681 | 0.0006997 | 0.004444665 | ADGRG1 |  |
| ENSG00000173409 | 1.620813736 | 0.00070129 | 0.004452835 | ARV1 |  |
| ENSG00000137409 | 0.726554766 | 0.00070257 | 0.004458998 | MTCH1 |  |
| ENSG00000135047 | 0.600033862 | 0.00070587 | 0.004477926 | CTSL |  |
| ENSG00000151304 | -1.035686786 | 0.00070818 | 0.004488702 | SRFBP1 |  |
| ENSG00000223247 | -1.255304456 | 0.00070819 | 0.004488702 | RNU2-64P |  |
| ENSG00000127241 | -2.759901046 | 0.0007108 | 0.004503293 | MASP1 |  |
| ENSG00000196476 | -1.350264136 | 0.00071633 | 0.004536366 | C20orf96 |  |
| ENSG00000199023 | -1.166200372 | 0.00071837 | 0.004545437 | MIR339 |  |
| ENSG00000179456 | -1.356032453 | 0.0007184 | 0.004545437 | ZBTB18 |  |
| ENSG00000197157 | 0.572321852 | 0.00072316 | 0.004573553 | SND1 |  |
| ENSG00000167695 | 1.257739126 | 0.00072509 | 0.004583759 | FAM57A |  |
| ENSG00000033627 | 0.787781056 | 0.00072662 | 0.004590917 | ATP6V0A1 |  |
| ENSG00000090487 | 1.222596707 | 0.00072686 | 0.004590917 | SPG21 |  |
| ENSG00000156983 | -1.099473608 | 0.00072938 | 0.00460487 | BRPF1 |  |
| ENSG00000142347 | -2.627710648 | 0.00073367 | 0.004629927 | MYO1F |  |
| ENSG00000119523 | 1.832451867 | 0.00073637 | 0.004644918 | ALG2 |  |
| ENSG00000125503 | -0.940751248 | 0.00073713 | 0.004647714 | PPP1R12C |  |
| ENSG00000124813 | 1.412746249 | 0.00073766 | 0.00464901 | RUNX2 |  |
| ENSG00000075711 | -0.735643251 | 0.0007383 | 0.004650999 | DLG1 |  |
| ENSG00000241469 | -2.228376983 | 0.00073862 | 0.004650999 | LINC00635 | |
| ENSG00000142208 | -0.711812268 | 0.00074342 | 0.004679167 | AKT1 |  |
| ENSG00000209482 | -0.607552196 | 0.00074446 | 0.00468365 | SNORD83A |  |
| ENSG00000132840 | -2.748211731 | 0.00075026 | 0.004716062 | BHMT2 |  |
| ENSG00000143774 | -0.973354823 | 0.00075026 | 0.004716062 | GUK1 |  |
| ENSG00000126803 | -1.759088539 | 0.00075252 | 0.004728188 | HSPA2 |  |
| ENSG00000140107 | -2.745144198 | 0.00075398 | 0.004735322 | SLC25A47 |  |
| ENSG00000159217 | -0.818284757 | 0.00075448 | 0.004736412 | IGF2BP1 |  |
| ENSG00000213719 | 0.466345142 | 0.00075502 | 0.004737723 | CLIC1 |  |
| ENSG00000101981 | -2.743633657 | 0.00075841 | 0.00475691 | F9 |  |
| ENSG00000156273 | -0.971988891 | 0.00076408 | 0.004790427 | BACH1 |  |
| ENSG00000164284 | -1.090704239 | 0.00076492 | 0.004793633 | GRPEL2 |  |
| ENSG00000269293 | 1.629241433 | 0.00076691 | 0.004803981 | ZSCAN16-AS1 | |
| ENSG00000169136 | -1.785125183 | 0.00077232 | 0.004835772 | ATF5 |  |
| ENSG00000157131 | -2.744855358 | 0.00077856 | 0.004872775 | C8A |  |
| ENSG00000110200 | -1.389837113 | 0.00077979 | 0.004878355 | ANAPC15 |  |
| ENSG00000179912 | -1.393823978 | 0.0007817 | 0.004888134 | R3HDM2 |  |
| ENSG00000156970 | -0.677090133 | 0.00078251 | 0.004891112 | BUB1B |  |
| ENSG00000132855 | -2.737821724 | 0.00078373 | 0.004896639 | ANGPTL3 |  |
| ENSG00000198826 | -0.997701429 | 0.00078639 | 0.004911119 | ARHGAP11A | |
| ENSG00000132471 | 0.905072968 | 0.00078701 | 0.004912884 | WBP2 |  |
| ENSG00000124357 | 1.069566247 | 0.00079234 | 0.004944015 | NAGK |  |
| ENSG00000125970 | -0.511303214 | 0.00079854 | 0.004980509 | RALY |  |
| ENSG00000183199 | -2.305067362 | 0.00080034 | 0.004989592 | HSP90AB3P | |
| ENSG00000230807 | -2.431843092 | 0.00080155 | 0.004993651 | AC099535.1 | |
| ENSG00000186818 | -2.735884727 | 0.00080168 | 0.004993651 | LILRB4 |  |
| ENSG00000103018 | 0.898333482 | 0.00080303 | 0.004998691 | CYB5B |  |
| ENSG00000138760 | 0.710550805 | 0.00080318 | 0.004998691 | SCARB2 |  |
| ENSG00000186350 | -1.199637184 | 0.00080603 | 0.005014271 | RXRA |  |
| ENSG00000144451 | -2.238130642 | 0.00080807 | 0.00502477 | SPAG16 |  |
| ENSG00000118402 | 1.883373623 | 0.00081314 | 0.005054124 | ELOVL4 |  |
| ENSG00000162851 | 1.502755277 | 0.00081543 | 0.005065853 | TFB2M |  |
| ENSG00000129151 | -2.730058144 | 0.00081573 | 0.005065853 | BBOX1 |  |
| ENSG00000091009 | -1.027148667 | 0.00081811 | 0.005078444 | RBM27 |  |
| ENSG00000198862 | 0.909417473 | 0.00081982 | 0.005086909 | LTN1 |  |
| ENSG00000112293 | -2.72779422 | 0.00082277 | 0.005103024 | GPLD1 |  |
| ENSG00000274997 | -0.478421099 | 0.00082416 | 0.00510946 | HIST1H2AH | |
| ENSG00000243562 | -1.259559038 | 0.00082572 | 0.005116919 | RN7SL838P | |
| ENSG00000188130 | -0.804042212 | 0.00083038 | 0.005143579 | MAPK12 |  |
| ENSG00000201900 | 2.279543133 | 0.00083496 | 0.00516976 | RNY1P13 |  |
| ENSG00000166411 | 1.186723041 | 0.00083666 | 0.005176489 | IDH3A |  |
| ENSG00000107341 | -0.827756426 | 0.00083677 | 0.005176489 | UBE2R2 |  |
| ENSG00000162384 | 0.972785165 | 0.00083802 | 0.00518205 | C1orf123 |  |
| ENSG00000237054 | -2.724262195 | 0.00084072 | 0.005196499 | PRMT5-AS1 | |
| ENSG00000201772 | -2.379192678 | 0.00084301 | 0.00520842 | SNORA5C |  |
| ENSG00000090061 | -1.475779892 | 0.00084926 | 0.005244814 | CCNK |  |
| ENSG00000070495 | -0.74845715 | 0.00085037 | 0.005249399 | JMJD6 |  |
| ENSG00000008256 | -0.829642699 | 0.00085239 | 0.005259651 | CYTH3 |  |
| ENSG00000133028 | -1.060196589 | 0.00085535 | 0.00527561 | SCO1 |  |
| ENSG00000101109 | -0.654028523 | 0.00086131 | 0.005310103 | STK4 |  |
| ENSG00000199121 | -1.177861601 | 0.00086329 | 0.00532006 | MIR26B |  |
| ENSG00000173715 | -1.519733668 | 0.00086997 | 0.005358956 | C11orf80 |  |
| ENSG00000076650 | -1.080402152 | 0.00087164 | 0.005366937 | GPATCH1 |  |
| ENSG00000112893 | 0.722594779 | 0.00087208 | 0.005367378 | MAN2A1 |  |
| ENSG00000100796 | -0.867139782 | 0.00087524 | 0.005384535 | PPP4R3A |  |
| ENSG00000107984 | 0.81372035 | 0.00087791 | 0.005396817 | DKK1 |  |
| ENSG00000182095 | -0.766606388 | 0.00087799 | 0.005396817 | TNRC18 |  |
| ENSG00000198887 | -0.908310373 | 0.00087927 | 0.005402398 | SMC5 |  |
| ENSG00000080189 | 1.168297296 | 0.00088011 | 0.005405289 | SLC35C2 |  |
| ENSG00000122958 | 0.869340075 | 0.00088147 | 0.005411339 | VPS26A |  |
| ENSG00000109572 | 1.050786612 | 0.00088508 | 0.005431192 | CLCN3 |  |
| ENSG00000173013 | -2.010909284 | 0.00088678 | 0.005439269 | CCDC96 |  |
| ENSG00000008517 | 1.115661595 | 0.00088954 | 0.005453907 | IL32 |  |
| ENSG00000277775 | 0.540993854 | 0.00089135 | 0.005462675 | HIST1H3F |  |
| ENSG00000106258 | -2.599403197 | 0.00089985 | 0.005510311 | CYP3A5 |  |
| ENSG00000162430 | 1.131982423 | 0.00089988 | 0.005510311 | SELENON |  |
| ENSG00000163792 | -2.709592102 | 0.00090558 | 0.00554284 | TCF23 |  |
| ENSG00000111843 | 0.812900842 | 0.00090716 | 0.005550134 | TMEM14C |  |
| ENSG00000086200 | 0.85093736 | 0.00090795 | 0.005552627 | IPO11 |  |
| ENSG00000121152 | -0.608108657 | 0.00091121 | 0.00557021 | NCAPH |  |
| ENSG00000175283 | 1.765526519 | 0.00092126 | 0.005629294 | DOLK |  |
| ENSG00000207116 | -1.200255577 | 0.00092714 | 0.005662814 | RNU6-31P |  |
| ENSG00000083444 | 0.764218149 | 0.00092848 | 0.005668613 | PLOD1 |  |
| ENSG00000163328 | 2.453187828 | 0.00092959 | 0.005672977 | GPR155 |  |
| ENSG00000249013 | -2.586308778 | 0.00093398 | 0.005697094 | FTH1P21 |  |
| ENSG00000239183 | -1.875860548 | 0.00093433 | 0.005697094 | SNORA84 |  |
| ENSG00000124155 | 0.66317857 | 0.00094222 | 0.005741047 | PIGT |  |
| ENSG00000108582 | 0.608951118 | 0.00094233 | 0.005741047 | CPD |  |
| ENSG00000123219 | 0.848032735 | 0.00094764 | 0.00577094 | CENPK |  |
| ENSG00000123130 | 0.6964472 | 0.00094864 | 0.00577459 | ACOT9 |  |
| ENSG00000222345 | -1.892665458 | 0.00095505 | 0.005811154 | RF00569 |  |
| ENSG00000155850 | 0.943249919 | 0.00095588 | 0.005813736 | SLC26A2 |  |
| ENSG00000185475 | 1.24181188 | 0.00095983 | 0.005835327 | TMEM179B |  |
| ENSG00000175899 | -2.697572522 | 0.00096257 | 0.005849525 | A2M |  |
| ENSG00000105339 | -1.225735425 | 0.00097147 | 0.00590115 | DENND3 |  |
| ENSG00000100325 | -0.822092712 | 0.00097521 | 0.005921358 | ASCC2 |  |
| ENSG00000163918 | 0.813861916 | 0.00098252 | 0.005963266 | RFC4 |  |
| ENSG00000112053 | -2.691932266 | 0.00098486 | 0.005974916 | SLC26A8 |  |
| ENSG00000124422 | 0.52286557 | 0.00098699 | 0.005985309 | USP22 |  |
| ENSG00000189057 | -1.134589245 | 0.00098751 | 0.005985963 | FAM111B |  |
| ENSG00000137474 | -2.690681448 | 0.00098904 | 0.00599276 | MYO7A |  |
| ENSG00000143575 | -0.955080541 | 0.00099177 | 0.006004728 | HAX1 |  |
| ENSG00000131043 | 1.064358657 | 0.00099185 | 0.006004728 | AAR2 |  |
| ENSG00000143374 | 0.977454286 | 0.00099241 | 0.006005585 | TARS2 |  |
| ENSG00000103353 | -0.991685972 | 0.00099413 | 0.006013496 | UBFD1 |  |
| ENSG00000199200 | 1.336647724 | 0.00099603 | 0.006021256 | RF00019 |  |
| ENSG00000106991 | 1.083936005 | 0.00099625 | 0.006021256 | ENG |  |
| ENSG00000109606 | 0.578903304 | 0.00100672 | 0.006082026 | DHX15 |  |
| ENSG00000102158 | 0.662318697 | 0.00101249 | 0.006114334 | MAGT1 |  |
| ENSG00000105723 | 1.230078547 | 0.00101707 | 0.006139389 | GSK3A |  |
| ENSG00000201801 | 0.936277406 | 0.00101928 | 0.00615017 | RNU5E-4P |  |
| ENSG00000185963 | -0.704260792 | 0.0010271 | 0.006194786 | BICD2 |  |
| ENSG00000198840 | -0.684201563 | 0.00103049 | 0.006212612 | MT-ND3 |  |
| ENSG00000129038 | -1.317718939 | 0.0010324 | 0.006221555 | LOXL1 |  |
| ENSG00000149782 | -0.77223426 | 0.00103366 | 0.006226515 | PLCB3 |  |
| ENSG00000100105 | 1.168469433 | 0.00103671 | 0.006242328 | PATZ1 |  |
| ENSG00000237412 | -2.678889942 | 0.001039 | 0.006253505 | PRSS56 |  |
| ENSG00000254858 | 1.326366452 | 0.00104219 | 0.006270054 | MPV17L2 |  |
| ENSG00000109184 | -1.173662357 | 0.00104716 | 0.006297325 | DCUN1D4 |  |
| ENSG00000182670 | -1.137807394 | 0.00104761 | 0.006297415 | TTC3 |  |
| ENSG00000164930 | 1.140335433 | 0.00105245 | 0.006320071 | FZD6 |  |
| ENSG00000129353 | 0.581315006 | 0.00105261 | 0.006320071 | SLC44A2 |  |
| ENSG00000212135 | -1.398847976 | 0.00105269 | 0.006320071 | SNORD67 |  |
| ENSG00000165732 | -0.53572984 | 0.00105341 | 0.006321756 | DDX21 |  |
| ENSG00000094914 | 0.921374965 | 0.00106428 | 0.006384347 | AAAS |  |
| ENSG00000076716 | -2.675510794 | 0.00107106 | 0.006420973 | GPC4 |  |
| ENSG00000166848 | -1.14238493 | 0.00107169 | 0.006420973 | TERF2IP |  |
| ENSG00000110660 | 1.100655313 | 0.00107172 | 0.006420973 | SLC35F2 |  |
| ENSG00000227001 | -1.529102709 | 0.00107848 | 0.006458787 | NBPF2P |  |
| ENSG00000278311 | -0.748583177 | 0.00108533 | 0.006497147 | GGNBP2 |  |
| ENSG00000036054 | -0.834348447 | 0.0010929 | 0.006539713 | TBC1D23 |  |
| ENSG00000039139 | -2.569379597 | 0.00109412 | 0.006544354 | DNAH5 |  |
| ENSG00000182326 | 1.245764016 | 0.00109619 | 0.006553991 | C1S |  |
| ENSG00000168137 | -0.60775511 | 0.00110221 | 0.006587282 | SETD5 |  |
| ENSG00000066468 | -2.336180872 | 0.00110294 | 0.006588906 | FGFR2 |  |
| ENSG00000100412 | 0.917632124 | 0.0011062 | 0.006605668 | ACO2 |  |
| ENSG00000162601 | -1.356954508 | 0.00111071 | 0.006629835 | MYSM1 |  |
| ENSG00000146112 | -0.752912283 | 0.00111345 | 0.006643461 | PPP1R18 |  |
| ENSG00000170385 | 0.984362153 | 0.00111609 | 0.006656462 | SLC30A1 |  |
| ENSG00000251791 | -0.54272786 | 0.00111855 | 0.006668392 | SCARNA6 |  |
| ENSG00000204387 | 1.173877184 | 0.00112893 | 0.006727458 | C6orf48 |  |
| ENSG00000244462 | 1.018869584 | 0.00113141 | 0.006739451 | RBM12 |  |
| ENSG00000079332 | 0.700328417 | 0.00113519 | 0.0067592 | SAR1A |  |
| ENSG00000219027 | -2.659782722 | 0.00114352 | 0.006806016 | RPS3AP2 |  |
| ENSG00000202515 | 0.944413824 | 0.00114504 | 0.006810502 | VTRNA1-3 |  |
| ENSG00000227881 | -2.65869797 | 0.00114548 | 0.006810502 | ASS1P5 |  |
| ENSG00000023287 | -0.883648574 | 0.00114569 | 0.006810502 | RB1CC1 |  |
| ENSG00000187021 | -0.603384966 | 0.00116165 | 0.006902519 | PNLIPRP1 |  |
| ENSG00000119314 | 0.661270321 | 0.00116395 | 0.006913338 | PTBP3 |  |
| ENSG00000117228 | -1.252598621 | 0.00116452 | 0.006913871 | GBP1 |  |
| ENSG00000216101 | 0.933647951 | 0.00116773 | 0.006929054 | MIR877 |  |
| ENSG00000104731 | -0.929130295 | 0.00116803 | 0.006929054 | KLHDC4 |  |
| ENSG00000008869 | 1.113952032 | 0.00116911 | 0.006932573 | HEATR5B |  |
| ENSG00000206659 | 1.154875861 | 0.00117459 | 0.006961519 | RF00019 |  |
| ENSG00000044459 | -1.14168747 | 0.00117508 | 0.006961519 | CNTLN |  |
| ENSG00000142156 | 0.769416269 | 0.00117543 | 0.006961519 | COL6A1 |  |
| ENSG00000163466 | 0.540238865 | 0.00117616 | 0.006962979 | ARPC2 |  |
| ENSG00000116991 | -1.175711843 | 0.00117969 | 0.00698098 | SIPA1L2 |  |
| ENSG00000251432 | 1.800379893 | 0.00118028 | 0.006981656 | AC108062.1 | |
| ENSG00000164163 | 0.906472042 | 0.00118484 | 0.007004469 | ABCE1 |  |
| ENSG00000122787 | -2.652645207 | 0.00118511 | 0.007004469 | AKR1D1 |  |
| ENSG00000147408 | -2.651666672 | 0.0011905 | 0.007033445 | CSGALNACT1 | |
| ENSG00000143947 | 0.674909243 | 0.00119319 | 0.007046441 | RPS27A |  |
| ENSG00000137478 | -1.24772186 | 0.00119396 | 0.007048121 | FCHSD2 |  |
| ENSG00000090060 | -0.73259846 | 0.00120513 | 0.007111139 | PAPOLA |  |
| ENSG00000197296 | 1.253163043 | 0.0012062 | 0.007114548 | FITM2 |  |
| ENSG00000171863 | 0.737091691 | 0.00121423 | 0.007158691 | RPS7 |  |
| ENSG00000184719 | 2.566251987 | 0.00121467 | 0.007158691 | RNLS |  |
| ENSG00000127884 | -0.648579488 | 0.00121626 | 0.007162664 | ECHS1 |  |
| ENSG00000023608 | -0.795241966 | 0.00121634 | 0.007162664 | SNAPC1 |  |
| ENSG00000112110 | 0.843770146 | 0.00121824 | 0.007170929 | MRPL18 |  |
| ENSG00000007047 | -1.159160016 | 0.00122129 | 0.00718326 | MARK4 |  |
| ENSG00000171302 | 0.780958445 | 0.00122133 | 0.00718326 | CANT1 |  |
| ENSG00000140526 | 0.563745075 | 0.0012362 | 0.007267746 | ABHD2 |  |
| ENSG00000122194 | -2.643417266 | 0.0012368 | 0.00726836 | PLG |  |
| ENSG00000138279 | 0.823934305 | 0.00123799 | 0.007272348 | ANXA7 |  |
| ENSG00000107562 | -2.643186781 | 0.00123911 | 0.007275643 | CXCL12 |  |
| ENSG00000180376 | -1.459418651 | 0.00123955 | 0.007275643 | CCDC66 |  |
| ENSG00000058091 | -1.574959133 | 0.00124144 | 0.00728373 | CDK14 |  |
| ENSG00000180628 | -0.734064035 | 0.00124501 | 0.007301711 | PCGF5 |  |
| ENSG00000130227 | 0.642199196 | 0.00124727 | 0.007312024 | XPO7 |  |
| ENSG00000117266 | -2.000503097 | 0.00125209 | 0.007337279 | CDK18 |  |
| ENSG00000102901 | -1.100947341 | 0.00125557 | 0.007354681 | CENPT |  |
| ENSG00000266088 | -2.639617442 | 0.00125871 | 0.007370083 | AC004585.1 | |
| ENSG00000073712 | -0.641508839 | 0.00126244 | 0.007388961 | FERMT2 |  |
| ENSG00000124214 | -0.5982057 | 0.00126566 | 0.007400701 | STAU1 |  |
| ENSG00000100024 | -2.638795139 | 0.0012665 | 0.007400701 | UPB1 |  |
| ENSG00000124134 | -2.638795139 | 0.0012665 | 0.007400701 | KCNS1 |  |
| ENSG00000239632 | -2.638795139 | 0.0012665 | 0.007400701 | MTND4LP3 |  |
| ENSG00000073584 | -2.43359238 | 0.00127078 | 0.007422723 | SMARCE1 |  |
| ENSG00000094880 | 0.767037055 | 0.0012732 | 0.007433848 | CDC23 |  |
| ENSG00000173085 | 1.13142408 | 0.00128272 | 0.007486402 | COQ2 |  |
| ENSG00000135940 | 0.795400529 | 0.0012879 | 0.007513591 | COX5B |  |
| ENSG00000197565 | 1.580593981 | 0.00128999 | 0.007522758 | COL4A6 |  |
| ENSG00000105677 | 1.341008618 | 0.00129312 | 0.007538005 | TMEM147 |  |
| ENSG00000033011 | 1.067167975 | 0.00130207 | 0.007587098 | ALG1 |  |
| ENSG00000153827 | -0.47943891 | 0.00130573 | 0.00760533 | TRIP12 |  |
| ENSG00000120158 | -1.431556017 | 0.00131189 | 0.007638179 | RCL1 |  |
| ENSG00000169446 | 1.445306972 | 0.00131767 | 0.007668695 | MMGT1 |  |
| ENSG00000110711 | -0.96990273 | 0.00132073 | 0.007683413 | AIP |  |
| ENSG00000168175 | 0.848220104 | 0.00132265 | 0.007691493 | MAPK1IP1L | |
| ENSG00000121064 | 0.810883186 | 0.00132679 | 0.007710951 | SCPEP1 |  |
| ENSG00000224032 | 0.95128943 | 0.00132706 | 0.007710951 | EPB41L4A-AS1 | |
| ENSG00000110906 | 0.807808594 | 0.0013293 | 0.007720849 | KCTD10 |  |
| ENSG00000121542 | 1.524531504 | 0.00133201 | 0.007730517 | SEC22A |  |
| ENSG00000112576 | 1.188280551 | 0.00133204 | 0.007730517 | CCND3 |  |
| ENSG00000138413 | 0.891442697 | 0.00133649 | 0.00775328 | IDH1 |  |
| ENSG00000119041 | -0.868709191 | 0.00133786 | 0.007758092 | GTF3C3 |  |
| ENSG00000138399 | 1.609093846 | 0.00134479 | 0.007795125 | FASTKD1 |  |
| ENSG00000103066 | 1.272889264 | 0.00134543 | 0.007795714 | PLA2G15 |  |
| ENSG00000174444 | -0.474332289 | 0.00135696 | 0.007859349 | RPL4 |  |
| ENSG00000166889 | -0.707377509 | 0.00136055 | 0.007877003 | PATL1 |  |
| ENSG00000143632 | 1.464319016 | 0.00137043 | 0.007931031 | ACTA1 |  |
| ENSG00000131149 | -1.094033105 | 0.00137437 | 0.007950642 | GSE1 |  |
| ENSG00000196890 | -1.512349752 | 0.00138129 | 0.007987462 | HIST3H2BB | |
| ENSG00000114770 | -1.295172155 | 0.00138379 | 0.007998761 | ABCC5 |  |
| ENSG00000177674 | 1.304682895 | 0.00138748 | 0.008016833 | AGTRAP |  |
| ENSG00000211448 | -1.27538462 | 0.0013881 | 0.008017229 | DIO2 |  |
| ENSG00000177917 | 0.960348627 | 0.0013897 | 0.008023286 | ARL6IP6 |  |
| ENSG00000244734 | -2.523580268 | 0.00139769 | 0.008066166 | HBB |  |
| ENSG00000099814 | -1.055191307 | 0.0014032 | 0.008094756 | CEP170B |  |
| ENSG00000170638 | -1.161740744 | 0.00142624 | 0.008224353 | TRABD |  |
| ENSG00000160439 | -2.069106706 | 0.00143781 | 0.008287755 | RDH13 |  |
| ENSG00000117143 | -0.539865028 | 0.00144103 | 0.008301958 | UAP1 |  |
| ENSG00000185909 | 2.242225672 | 0.00144142 | 0.008301958 | KLHDC8B |  |
| ENSG00000217733 | -2.610598612 | 0.00144576 | 0.008321084 | CCT7P1 |  |
| ENSG00000162924 | 1.385929909 | 0.00144589 | 0.008321084 | REL |  |
| ENSG00000169903 | -2.610084441 | 0.00145311 | 0.008359265 | TM4SF4 |  |
| ENSG00000160932 | 1.046994228 | 0.00145814 | 0.008384904 | LY6E |  |
| ENSG00000138107 | 0.661312468 | 0.00146186 | 0.008399699 | ACTR1A |  |
| ENSG00000144580 | 0.947002126 | 0.00146188 | 0.008399699 | CNOT9 |  |
| ENSG00000154511 | 1.989424888 | 0.00146549 | 0.008415276 | FAM69A |  |
| ENSG00000180182 | 0.682543506 | 0.00146576 | 0.008415276 | MED14 |  |
| ENSG00000105732 | 1.506640609 | 0.0014671 | 0.008419636 | ZNF574 |  |
| ENSG00000037749 | 0.958383344 | 0.00146827 | 0.008422991 | MFAP3 |  |
| ENSG00000163661 | 0.955929508 | 0.00146901 | 0.00842389 | PTX3 |  |
| ENSG00000110395 | 0.703689159 | 0.00147322 | 0.008444733 | CBL |  |
| ENSG00000178802 | 1.517529358 | 0.00147449 | 0.008448655 | MPI |  |
| ENSG00000120054 | -2.603856239 | 0.001476 | 0.008453953 | CPN1 |  |
| ENSG00000278463 | 0.718583233 | 0.00147935 | 0.008469741 | HIST1H2AB | |
| ENSG00000039319 | 0.882572091 | 0.00148339 | 0.008489534 | ZFYVE16 |  |
| ENSG00000198730 | -0.666035248 | 0.00148469 | 0.00849363 | CTR9 |  |
| ENSG00000173264 | 1.50181276 | 0.00148623 | 0.008499038 | GPR137 |  |
| ENSG00000202151 | 2.329372995 | 0.00148899 | 0.008511488 | RNY4P28 |  |
| ENSG00000179091 | 0.684808404 | 0.00148993 | 0.008511943 | CYC1 |  |
| ENSG00000105953 | 0.631435531 | 0.00149025 | 0.008511943 | OGDH |  |
| ENSG00000104756 | 1.233479272 | 0.00149715 | 0.008547947 | KCTD9 |  |
| ENSG00000119977 | 1.420502161 | 0.00150369 | 0.008581894 | TCTN3 |  |
| ENSG00000171488 | 1.409270166 | 0.00150488 | 0.008585292 | LRRC8C |  |
| ENSG00000112972 | 0.613488489 | 0.00151505 | 0.008639914 | HMGCS1 |  |
| ENSG00000164237 | 2.093999408 | 0.00152377 | 0.008685052 | CMBL |  |
| ENSG00000132912 | 0.702396246 | 0.00152434 | 0.008685052 | DCTN4 |  |
| ENSG00000110651 | 0.567097255 | 0.00152477 | 0.008685052 | CD81 |  |
| ENSG00000084731 | -0.899458745 | 0.00152546 | 0.008685587 | KIF3C |  |
| ENSG00000095637 | -2.594543715 | 0.00154513 | 0.008790648 | SORBS1 |  |
| ENSG00000113905 | -2.594543715 | 0.00154513 | 0.008790648 | HRG |  |
| ENSG00000048405 | 1.103909185 | 0.00154678 | 0.008795792 | ZNF800 |  |
| ENSG00000147604 | -0.487835546 | 0.00154726 | 0.008795792 | RPL7 |  |
| ENSG00000100554 | -0.748646204 | 0.00155107 | 0.008813983 | ATP6V1D |  |
| ENSG00000188850 | -2.592435491 | 0.00155988 | 0.008860609 | AC114947.1 | |
| ENSG00000130772 | 1.973294483 | 0.0015681 | 0.008903754 | MED18 |  |
| ENSG00000070087 | 0.793462448 | 0.00156905 | 0.008905681 | PFN2 |  |
| ENSG00000162413 | 1.057033249 | 0.00157121 | 0.008914435 | KLHL21 |  |
| ENSG00000087008 | 1.322290496 | 0.00157402 | 0.008926841 | ACOX3 |  |
| ENSG00000207996 | 1.015137579 | 0.00157954 | 0.008954629 | MIR301A |  |
| ENSG00000119953 | -0.819607296 | 0.00159297 | 0.009027224 | SMNDC1 |  |
| ENSG00000112378 | 1.200397598 | 0.00159383 | 0.009028552 | PERP |  |
| ENSG00000141506 | -2.58673607 | 0.00159908 | 0.00905477 | PIK3R5 |  |
| ENSG00000214128 | -2.587605556 | 0.00160637 | 0.009092484 | TMEM213 |  |
| ENSG00000165813 | -1.26248749 | 0.0016094 | 0.009106093 | CCDC186 |  |
| ENSG00000101199 | -0.754283351 | 0.00161285 | 0.009122007 | ARFGAP1 |  |
| ENSG00000205531 | -0.585295444 | 0.00161421 | 0.009126125 | NAP1L4 |  |
| ENSG00000127554 | -1.211922315 | 0.0016154 | 0.009127364 | GFER |  |
| ENSG00000108799 | -1.252414904 | 0.00161569 | 0.009127364 | EZH1 |  |
| ENSG00000134056 | 1.747908218 | 0.00162775 | 0.00919191 | MRPS36 |  |
| ENSG00000239306 | 1.051455871 | 0.00163458 | 0.009226841 | RBM14 |  |
| ENSG00000071243 | -1.239173745 | 0.00164614 | 0.009288459 | ING3 |  |
| ENSG00000114209 | -0.848372098 | 0.00165858 | 0.009355038 | PDCD10 |  |
| ENSG00000168569 | 1.7728397 | 0.00166283 | 0.00937375 | TMEM223 |  |
| ENSG00000177200 | -0.790710692 | 0.0016632 | 0.00937375 | CHD9 |  |
| ENSG00000115641 | 0.843893642 | 0.00166446 | 0.009377179 | FHL2 |  |
| ENSG00000146833 | -0.862940553 | 0.00166581 | 0.009381118 | TRIM4 |  |
| ENSG00000133422 | -0.957256329 | 0.00167218 | 0.009413369 | MORC2 |  |
| ENSG00000115944 | 0.946710906 | 0.00167623 | 0.00943246 | COX7A2L |  |
| ENSG00000111331 | 1.043022726 | 0.00168872 | 0.009497374 | OAS3 |  |
| ENSG00000019549 | 1.383314394 | 0.00168908 | 0.009497374 | SNAI2 |  |
| ENSG00000133119 | 0.892019708 | 0.00169441 | 0.009521446 | RFC3 |  |
| ENSG00000134248 | 1.430657601 | 0.00169468 | 0.009521446 | LAMTOR5 |  |
| ENSG00000237118 | -2.574178818 | 0.00169728 | 0.009532367 | CYP2F2P |  |
| ENSG00000164649 | -1.008091014 | 0.00169918 | 0.009533999 | CDCA7L |  |
| ENSG00000237651 | -2.032337744 | 0.00169954 | 0.009533999 | C2orf74 |  |
| ENSG00000069667 | -1.986754642 | 0.00169955 | 0.009533999 | RORA |  |
| ENSG00000205644 | -2.573862764 | 0.00170261 | 0.009547447 | AC025186.1 | |
| ENSG00000164180 | 1.046798085 | 0.00171561 | 0.009616586 | TMEM161B |  |
| ENSG00000083520 | -0.794671523 | 0.00172441 | 0.009662166 | DIS3 |  |
| ENSG00000100162 | 0.886245594 | 0.00173131 | 0.0096971 | CENPM |  |
| ENSG00000187193 | 0.73887873 | 0.00175024 | 0.009796873 | MT1X |  |
| ENSG00000278771 | -1.635634865 | 0.00175048 | 0.009796873 | RN7SL3 |  |
| ENSG00000076555 | -1.831055178 | 0.00175395 | 0.009809877 | ACACB |  |
| ENSG00000165915 | 0.744048589 | 0.00175416 | 0.009809877 | SLC39A13 |  |
| ENSG00000128422 | -0.85353508 | 0.00175609 | 0.009816855 | KRT17 |  |
| ENSG00000165630 | -1.435263232 | 0.00177913 | 0.009941775 | PRPF18 |  |
| ENSG00000177613 | 1.179115132 | 0.00178407 | 0.009965567 | CSTF2T |  |
| ENSG00000164466 | 0.75584361 | 0.00178827 | 0.009985166 | SFXN1 |  |
| ENSG00000259904 | -2.26453969 | 0.00178939 | 0.009987532 | ACTG1P15 |  |
