# Supplementary material for: PM2.5 promotes NSCLC carcinogenesis through translationally and transcriptionally activating DLAT-mediated glycolysis reprograming
Source: J Exp Clin Cancer Res. 2022 Jul 22;41:229. doi: 10.1186/s13046-022-02437-8 (PMC9308224; doi:10.1186/s13046-022-02437-8)
Supplement: Supplementary file 12 — Additional file 12: Table S4. Genes with significant change in translation efficiency (TE) in PM2.5-exposed BEAS-2B cells. [file 13046_2022_2437_MOESM12_ESM.docx]

| **Table S4. Genes with significant change in translation efficiency (TE) in PM2.5-exposed BEAS-2B cells** | | | | | | |
| --- | --- | --- | --- | --- | --- | --- |
| **Gene_id** | **TETE_control cells** | **TETE_PM2.5-exposed cells** | **log2FoldChange** | **P value** | **Padj** | **Gene name** |
| ENSG00000107738 | 0.00143905 | 0.04053553 | 4.815997959 | 3.31E-11 | 1.09E-09 | VSIR |
| ENSG00000184719 | 0.003219286 | 0.085636817 | 4.733418292 | 2.81E-06 | 3.49E-05 | RNLS |
| ENSG00000249264 | 1.181866626 | 31.42542413 | 4.732793075 | 6.53E-06 | 7.26E-05 | EEF1A1P9 |
| ENSG00000197557 | 0.002645253 | 0.068467983 | 4.693951671 | 9.84E-05 | 0.000785059 | TTC30A |
| ENSG00000167536 | 0.002158484 | 0.053264362 | 4.625079953 | 4.27E-06 | 5.02E-05 | DHRS13 |
| ENSG00000270605 | 0.023594144 | 0.473181494 | 4.325892904 | 0.000167121 | 0.001233661 | AL353622.1 |
| ENSG00000112182 | 0.02151053 | 0.428860713 | 4.317394161 | 0.000452111 | 0.002821374 | BACH2 |
| ENSG00000125148 | 0.034532557 | 0.677527632 | 4.29425071 | 0 | 0 | MT2A |
| ENSG00000006757 | 0.003395763 | 0.06627224 | 4.286597113 | 4.23E-05 | 0.000377671 | PNPLA4 |
| ENSG00000137819 | 0.003262241 | 0.058653977 | 4.168293515 | 9.85E-05 | 0.000785238 | PAQR5 |
| ENSG00000233521 | 0.00711981 | 0.123667265 | 4.118481109 | 4.02E-05 | 0.000360967 | LINC01638 |
| ENSG00000222009 | 0.002518668 | 0.043206518 | 4.100516111 | 0.000354181 | 0.00229597 | BTBD19 |
| ENSG00000173825 | 0.006428252 | 0.107796666 | 4.067742264 | 0.000692238 | 0.004023277 | TIGD3 |
| ENSG00000242516 | 0.007286451 | 0.122060619 | 4.066237684 | 0.00045072 | 0.002815537 | LINC00960 |
| ENSG00000099958 | 0.153973532 | 2.347173883 | 3.930170447 | 3.34E-05 | 0.000308188 | DERL3 |
| ENSG00000278619 | 0.00416494 | 0.05968853 | 3.841086231 | 0.000187399 | 0.001355883 | MRM1 |
| ENSG00000177854 | 0.002147429 | 0.030704454 | 3.837765675 | 0.001825758 | 0.008942866 | TMEM187 |
| ENSG00000131127 | 0.001986452 | 0.027971876 | 3.815710847 | 0.000323909 | 0.002135419 | ZNF141 |
| ENSG00000101017 | 0.00588349 | 0.080429924 | 3.772988228 | 6.46E-08 | 1.18E-06 | CD40 |
| ENSG00000203778 | 0.002110882 | 0.028475725 | 3.753814377 | 0.001268312 | 0.006613811 | FAM229B |
| ENSG00000021645 | 0.012764198 | 0.164311085 | 3.686255006 | 0.001692259 | 0.00839844 | NRXN3 |
| ENSG00000064205 | 0.008683763 | 0.111160831 | 3.678184306 | 5.05E-07 | 7.53E-06 | WISP2 |
| ENSG00000171984 | 0.014539701 | 0.184755678 | 3.667549196 | 1.16E-05 | 0.000121921 | SHLD1 |
| ENSG00000166396 | 0.004109999 | 0.049597183 | 3.59304808 | 2.27E-05 | 0.000219377 | SERPINB7 |
| ENSG00000186907 | 0.01056488 | 0.124075043 | 3.553864711 | 0.00045322 | 0.002822356 | RTN4RL2 |
| ENSG00000164039 | 0.008660897 | 0.098101901 | 3.501692704 | 3.03E-05 | 0.00028412 | BDH2 |
| ENSG00000118363 | 0.020290641 | 0.224226145 | 3.466068145 | 2.68E-12 | 1.03E-10 | SPCS2 |
| ENSG00000253716 | 0.008749908 | 0.095765898 | 3.45217228 | 0.000162759 | 0.001205901 | MINCR |
| ENSG00000198794 | 0.005268371 | 0.054164327 | 3.361914256 | 3.07E-08 | 6.02E-07 | SCAMP5 |
| ENSG00000119640 | 0.004882539 | 0.046358894 | 3.247142701 | 0.001585256 | 0.007942796 | ACYP1 |
| ENSG00000144120 | 0.004126302 | 0.038787887 | 3.232684736 | 2.32E-05 | 0.000223926 | TMEM177 |
| ENSG00000173928 | 0.014527469 | 0.135360123 | 3.219947505 | 0.001992898 | 0.009570529 | SWSAP1 |
| ENSG00000007255 | 0.012679013 | 0.114471743 | 3.174477219 | 4.28E-07 | 6.49E-06 | TRAPPC6A |
| ENSG00000255468 | 0.019997148 | 0.178864192 | 3.160998443 | 1.44E-05 | 0.000146659 | AP001107.9 |
| ENSG00000185909 | 0.002609422 | 0.022946653 | 3.136481401 | 2.44E-06 | 3.11E-05 | KLHDC8B |
| ENSG00000163328 | 0.013772754 | 0.11906741 | 3.111889569 | 0.00028903 | 0.001943841 | GPR155 |
| ENSG00000205485 | 0.009542598 | 0.081822987 | 3.1000522 | 0.000600045 | 0.003581626 | AC004980.1 |
| ENSG00000102317 | 0.035707014 | 0.298190451 | 3.061954673 | 0 | 0 | RBM3 |
| ENSG00000128394 | 0.009632026 | 0.076121251 | 2.982388045 | 0.000339049 | 0.002212188 | APOBEC3F |
| ENSG00000124444 | 0.004904102 | 0.03832444 | 2.966203883 | 1.40E-06 | 1.89E-05 | ZNF576 |
| ENSG00000173432 | 0.007571451 | 0.056293872 | 2.894336103 | 3.24E-05 | 0.000300887 | SAA1 |
| ENSG00000135211 | 0.019094494 | 0.139580508 | 2.869868924 | 5.38E-08 | 9.97E-07 | TMEM60 |
| ENSG00000147027 | 0.007822687 | 0.055906208 | 2.837272311 | 3.41E-06 | 4.12E-05 | TMEM47 |
| ENSG00000226380 | 0.066274125 | 0.463946213 | 2.807439939 | 0.001318363 | 0.006833323 | LINC-PINT |
| ENSG00000165949 | 0.003508369 | 0.023963255 | 2.771951347 | 0.000650084 | 0.003833867 | IFI27 |
| ENSG00000183621 | 0.007771366 | 0.052942081 | 2.768174767 | 0.000692077 | 0.004023277 | ZNF438 |
| ENSG00000181264 | 0.006666903 | 0.044692912 | 2.744957427 | 6.57E-05 | 0.000554778 | TMEM136 |
| ENSG00000276966 | 301.916937 | 2015.449801 | 2.738878254 | 0.000185439 | 0.001344122 | HIST1H4E |
| ENSG00000115268 | 0.04811663 | 0.318242473 | 2.725518876 | 0 | 0 | RPS15 |
| ENSG00000255198 | 0.089458588 | 0.584004361 | 2.706687257 | 0.00020781 | 0.001471679 | SNHG9 |
| ENSG00000108590 | 0.02194061 | 0.143185491 | 2.706209767 | 0.000152621 | 0.001141338 | MED31 |
| ENSG00000198918 | 0.010825836 | 0.07046985 | 2.702527715 | 8.44E-09 | 1.81E-07 | RPL39 |
| ENSG00000262814 | 0.016709414 | 0.10825871 | 2.695750046 | 0.000347329 | 0.002258859 | MRPL12 |
| ENSG00000187266 | 0.006666704 | 0.043164192 | 2.694789468 | 7.68E-05 | 0.000634196 | EPOR |
| ENSG00000138172 | 0.006129521 | 0.039230957 | 2.678146296 | 8.14E-08 | 1.44E-06 | CALHM2 |
| ENSG00000085871 | 0.01409184 | 0.089381273 | 2.665112589 | 0.000412619 | 0.002618337 | MGST2 |
| ENSG00000119328 | 0.007472004 | 0.046963751 | 2.651980456 | 4.14E-06 | 4.88E-05 | FAM206A |
| ENSG00000177352 | 0.003055262 | 0.019126929 | 2.646237231 | 0.000195252 | 0.001402572 | CCDC71 |
| ENSG00000145439 | 0.026355327 | 0.163637752 | 2.634339146 | 7.56E-14 | 3.65E-12 | CBR4 |
| ENSG00000185499 | 0.007587399 | 0.0468318 | 2.625811226 | 2.93E-09 | 6.87E-08 | MUC1 |
| ENSG00000104783 | 0.009044127 | 0.055457547 | 2.61633071 | 6.13E-10 | 1.64E-08 | KCNN4 |
| ENSG00000069943 | 0.022465186 | 0.136515639 | 2.603303309 | 4.78E-10 | 1.29E-08 | PIGB |
| ENSG00000269293 | 0.166312451 | 1.001372226 | 2.59001026 | 3.97E-05 | 0.000356708 | ZSCAN16-AS1 |
| ENSG00000267530 | 0.012910252 | 0.077414621 | 2.584088913 | 7.97E-06 | 8.66E-05 | LINC01836 |
| ENSG00000253522 | 0.014627241 | 0.08720818 | 2.575805809 | 9.02E-07 | 1.27E-05 | MIR3142HG |
| ENSG00000254162 | 0.111100011 | 0.661196188 | 2.573219443 | 0.000273472 | 0.001857913 | AC009812.3 |
| ENSG00000175482 | 0.026789041 | 0.1581863 | 2.561909795 | 0.00066435 | 0.003898884 | POLD4 |
| ENSG00000139508 | 0.009527117 | 0.054529617 | 2.516928368 | 5.46E-05 | 0.000468992 | SLC46A3 |
| ENSG00000130772 | 0.007044713 | 0.040248732 | 2.514330508 | 8.45E-05 | 0.000688274 | MED18 |
| ENSG00000110628 | 0.018118778 | 0.102217375 | 2.496082859 | 3.38E-10 | 9.44E-09 | SLC22A18 |
| ENSG00000090661 | 0.020287732 | 0.113627689 | 2.485634938 | 4.20E-09 | 9.56E-08 | CERS4 |
| ENSG00000082515 | 0.012107289 | 0.06624286 | 2.451889066 | 4.44E-08 | 8.41E-07 | MRPL22 |
| ENSG00000142166 | 0.013803815 | 0.074948735 | 2.440837085 | 0 | 0 | IFNAR1 |
| ENSG00000101464 | 0.024526477 | 0.132642211 | 2.435128045 | 3.55E-14 | 1.80E-12 | PIGU |
| ENSG00000263823 | 0.068399956 | 0.368828971 | 2.430884687 | 0.000125887 | 0.000969139 | AC009831.1 |
| ENSG00000152193 | 0.029183278 | 0.155198414 | 2.410899947 | 7.59E-11 | 2.33E-09 | RNF219 |
| ENSG00000077463 | 0.009888926 | 0.052558379 | 2.410035069 | 2.71E-05 | 0.000257419 | SIRT6 |
| ENSG00000096092 | 0.025578501 | 0.134575828 | 2.395415658 | 1.19E-07 | 2.03E-06 | TMEM14A |
| ENSG00000277459 | 0.047759359 | 0.250910858 | 2.393319531 | 0.001699559 | 0.008427712 | AP001527.2 |
| ENSG00000122873 | 0.02868377 | 0.145879194 | 2.346467588 | 1.95E-07 | 3.19E-06 | CISD1 |
| ENSG00000232112 | 0.013540889 | 0.068188323 | 2.332202263 | 0.000957583 | 0.005238823 | TMA7 |
| ENSG00000169715 | 0.018365902 | 0.091973837 | 2.324193773 | 2.21E-07 | 3.55E-06 | MT1E |
| ENSG00000164237 | 0.026038061 | 0.129132598 | 2.310159295 | 0.000231084 | 0.001613698 | CMBL |
| ENSG00000100344 | 0.010269391 | 0.050613655 | 2.301175982 | 0.001154698 | 0.006130351 | PNPLA3 |
| ENSG00000134056 | 0.007016366 | 0.034226095 | 2.286300863 | 0.00020373 | 0.001448762 | MRPS36 |
| ENSG00000143434 | 0.035975241 | 0.174119192 | 2.27499896 | 2.37E-09 | 5.62E-08 | SEMA6C |
| ENSG00000170860 | 0.085716744 | 0.412921574 | 2.268218836 | 4.12E-09 | 9.41E-08 | LSM3 |
| ENSG00000142227 | 0.031477954 | 0.150557496 | 2.257900872 | 2.01E-12 | 7.92E-11 | EMP3 |
| ENSG00000187118 | 0.014127066 | 0.067191493 | 2.249816705 | 0.000153217 | 0.001145083 | CMC1 |
| ENSG00000204315 | 0.010062641 | 0.047587157 | 2.24156323 | 0.000777468 | 0.004426633 | FKBPL |
| ENSG00000138744 | 0.015021211 | 0.071003764 | 2.240894344 | 0.000266574 | 0.001822373 | NAAA |
| ENSG00000101443 | 0.015545585 | 0.073088563 | 2.233140778 | 0.000274593 | 0.001864337 | WFDC2 |
| ENSG00000167565 | 0.022404816 | 0.104727267 | 2.224756323 | 4.56E-13 | 2.01E-11 | SERTAD3 |
| ENSG00000163923 | 0.009398594 | 0.043584947 | 2.213313139 | 0.000387131 | 0.002473568 | RPL39L |
| ENSG00000150779 | 0.037928314 | 0.175054996 | 2.206461091 | 3.39E-06 | 4.11E-05 | TIMM8B |
| ENSG00000003509 | 0.01537711 | 0.070460067 | 2.196021499 | 1.26E-05 | 0.000130233 | NDUFAF7 |
| ENSG00000008118 | 0.054264525 | 0.24790781 | 2.191722448 | 0.001356615 | 0.00699241 | CAMK1G |
| ENSG00000127419 | 0.011413363 | 0.051750567 | 2.180850697 | 1.44E-05 | 0.000146659 | TMEM175 |
| ENSG00000182544 | 0.018869558 | 0.083811526 | 2.151088048 | 3.48E-12 | 1.30E-10 | MFSD5 |
| ENSG00000154511 | 0.014077536 | 0.062510564 | 2.15070519 | 0.000105196 | 0.000829499 | FAM69A |
| ENSG00000198892 | 0.003651174 | 0.016109515 | 2.141480795 | 0.000318944 | 0.002107307 | SHISA4 |
| ENSG00000169599 | 0.020507135 | 0.08989975 | 2.132191168 | 4.30E-06 | 5.05E-05 | NFU1 |
| ENSG00000168872 | 0.021935591 | 0.096005164 | 2.129838408 | 6.35E-06 | 7.08E-05 | DDX19A |
| ENSG00000083844 | 0.009228568 | 0.040252514 | 2.124900209 | 2.05E-07 | 3.32E-06 | ZNF264 |
| ENSG00000125652 | 0.016888272 | 0.073382491 | 2.119414128 | 3.19E-05 | 0.000296849 | ALKBH7 |
| ENSG00000141026 | 0.006205978 | 0.026843061 | 2.112818811 | 0.000130876 | 0.000999231 | MED9 |
| ENSG00000100564 | 0.016810183 | 0.072671956 | 2.112063286 | 6.82E-05 | 0.000569137 | PIGH |
| ENSG00000088035 | 0.065724453 | 0.283618127 | 2.109447618 | 1.32E-06 | 1.79E-05 | ALG6 |
| ENSG00000124786 | 0.016993293 | 0.072753973 | 2.098060576 | 1.95E-08 | 3.96E-07 | SLC35B3 |
| ENSG00000284726 | 1.37212025 | 5.87146786 | 2.097314298 | 2.31E-06 | 2.97E-05 | AL109936.6 |
| ENSG00000198856 | 0.012434321 | 0.053051941 | 2.093077749 | 1.93E-05 | 0.000189134 | OSTC |
| ENSG00000114544 | 0.01530906 | 0.064882464 | 2.083442925 | 5.82E-13 | 2.50E-11 | SLC41A3 |
| ENSG00000204435 | 0.039734412 | 0.168213649 | 2.081833864 | 0.000987357 | 0.005365101 | CSNK2B |
| ENSG00000004455 | 0.01957035 | 0.0824621 | 2.075061609 | 0 | 0 | AK2 |
| ENSG00000008282 | 0.019637022 | 0.082463691 | 2.070182861 | 2.39E-10 | 6.81E-09 | SYPL1 |
| ENSG00000181458 | 0.05497108 | 0.229386695 | 2.061036973 | 0.000625726 | 0.0037139 | TMEM45A |
| ENSG00000255112 | 0.133316662 | 0.556271548 | 2.060932215 | 6.69E-10 | 1.78E-08 | CHMP1B |
| ENSG00000185803 | 0.018107917 | 0.075262438 | 2.05530944 | 4.91E-13 | 2.14E-11 | SLC52A2 |
| ENSG00000119523 | 0.007880617 | 0.032641136 | 2.050310863 | 5.19E-05 | 0.000449385 | ALG2 |
| ENSG00000114988 | 0.012683667 | 0.052379427 | 2.046028412 | 3.18E-09 | 7.40E-08 | LMAN2L |
| ENSG00000197208 | 0.035306849 | 0.145410256 | 2.042109073 | 0 | 0 | SLC22A4 |
| ENSG00000205133 | 0.012734737 | 0.052322566 | 2.038664147 | 0.001607358 | 0.008036788 | TRIQK |
| ENSG00000162976 | 0.016457406 | 0.067516622 | 2.036505742 | 1.08E-05 | 0.00011436 | PQLC3 |
| ENSG00000091073 | 0.004985406 | 0.020231871 | 2.020846842 | 5.87E-06 | 6.62E-05 | DTX2 |
| ENSG00000101574 | 0.009505503 | 0.038553254 | 2.020017848 | 8.88E-05 | 0.000716497 | METTL4 |
| ENSG00000147535 | 0.007577122 | 0.030714989 | 2.019220982 | 7.12E-06 | 7.86E-05 | PLPP5 |
| ENSG00000125995 | 0.022145743 | 0.089763104 | 2.019093184 | 3.85E-06 | 4.59E-05 | ROMO1 |
| ENSG00000151247 | 0.041097756 | 0.165914933 | 2.01331221 | 3.46E-12 | 1.30E-10 | EIF4E |
| ENSG00000143252 | 0.030050757 | 0.120978989 | 2.009283263 | 2.25E-10 | 6.45E-09 | SDHC |
| ENSG00000111335 | 0.007355548 | 0.029534039 | 2.005474026 | 0.000455833 | 0.002837156 | OAS2 |
| ENSG00000168569 | 0.020010056 | 0.080086871 | 2.000840547 | 0.000385764 | 0.002467455 | TMEM223 |
| ENSG00000118402 | 0.019841328 | 0.079398851 | 2.000609501 | 3.24E-05 | 0.000300887 | ELOVL4 |
| ENSG00000122783 | 0.016842373 | 0.067061039 | 1.993379399 | 2.56E-07 | 4.06E-06 | CYREN |
| ENSG00000184260 | 1.278724143 | 5.087445002 | 1.992236226 | 6.47E-06 | 7.19E-05 | HIST2H2AC |
| ENSG00000135404 | 0.021715996 | 0.086345794 | 1.991367741 | 1.55E-12 | 6.22E-11 | CD63 |
| ENSG00000120963 | 0.013129583 | 0.052177625 | 1.990610135 | 1.18E-07 | 2.02E-06 | ZNF706 |
| ENSG00000173409 | 0.017784461 | 0.070579026 | 1.988622246 | 0.000125304 | 0.000966508 | ARV1 |
| ENSG00000277161 | 0.007091969 | 0.028112641 | 1.986960841 | 0.000142927 | 0.001076201 | PIGW |
| ENSG00000131966 | 0.016888096 | 0.066768893 | 1.983169395 | 2.11E-05 | 0.000205217 | ACTR10 |
| ENSG00000163626 | 0.007628837 | 0.030108048 | 1.980614127 | 0.001152078 | 0.006121848 | COX18 |
| ENSG00000111775 | 0.141939832 | 0.559232389 | 1.978168416 | 1.49E-06 | 1.99E-05 | COX6A1 |
| ENSG00000115540 | 0.018891247 | 0.074367028 | 1.976945164 | 4.45E-05 | 0.000393764 | MOB4 |
| ENSG00000156411 | 0.021123744 | 0.082917001 | 1.972802391 | 3.77E-05 | 0.000340461 | ATP5MPL |
| ENSG00000120860 | 0.01290931 | 0.050643171 | 1.971955851 | 0.001538709 | 0.007760087 | WASHC3 |
| ENSG00000228672 | 0.009410064 | 0.036612206 | 1.960048285 | 0.000876884 | 0.004865959 | PROB1 |
| ENSG00000116815 | 0.008090328 | 0.031427916 | 1.957776477 | 0.000263306 | 0.001804908 | CD58 |
| ENSG00000137198 | 0.013522224 | 0.052295245 | 1.951347375 | 2.65E-05 | 0.000252185 | GMPR |
| ENSG00000196655 | 0.025110963 | 0.09706599 | 1.950648551 | 8.58E-05 | 0.000696728 | TRAPPC4 |
| ENSG00000143570 | 0.034895974 | 0.134851281 | 1.950236713 | 0.000274728 | 0.001864337 | SLC39A1 |
| ENSG00000108947 | 0.004456712 | 0.017210858 | 1.949267404 | 0.000684561 | 0.003990231 | EFNB3 |
| ENSG00000175283 | 0.02211855 | 0.085213357 | 1.945822759 | 2.20E-09 | 5.23E-08 | DOLK |
| ENSG00000214160 | 0.019917147 | 0.076561487 | 1.942607877 | 3.89E-15 | 2.27E-13 | ALG3 |
| ENSG00000143753 | 0.018465483 | 0.070502011 | 1.932833441 | 8.03E-13 | 3.33E-11 | DEGS1 |
| ENSG00000106028 | 0.021630367 | 0.082449465 | 1.930451989 | 9.93E-08 | 1.73E-06 | SSBP1 |
| ENSG00000150768 | 0.013336324 | 0.050718168 | 1.927141533 | 0 | 0 | DLAT |
| ENSG00000108518 | 0.024893066 | 0.094470383 | 1.924118172 | 1.21E-12 | 4.90E-11 | PFN1 |
| ENSG00000143553 | 0.022223207 | 0.084309678 | 1.923631236 | 0.00011874 | 0.000921789 | SNAPIN |
| ENSG00000128283 | 0.01363653 | 0.051394309 | 1.914132056 | 6.89E-08 | 1.24E-06 | CDC42EP1 |
| ENSG00000119878 | 0.034236869 | 0.127500132 | 1.896876078 | 7.91E-05 | 0.000650637 | CRIPT |
| ENSG00000163933 | 0.031867578 | 0.118334735 | 1.892712316 | 1.06E-05 | 0.000112896 | RFT1 |
| ENSG00000134058 | 0.012541164 | 0.046506606 | 1.890764429 | 3.34E-06 | 4.06E-05 | CDK7 |
| ENSG00000171155 | 0.033195966 | 0.123085072 | 1.890575949 | 2.09E-05 | 0.000203299 | C1GALT1C1 |
| ENSG00000134248 | 0.014489027 | 0.053538553 | 1.885617405 | 0.000643111 | 0.003796472 | LAMTOR5 |
| ENSG00000164342 | 0.02019158 | 0.074426481 | 1.882062223 | 0.000651559 | 0.003840686 | TLR3 |
| ENSG00000100300 | 0.008603851 | 0.031709764 | 1.881872676 | 3.70E-07 | 5.67E-06 | TSPO |
| ENSG00000128340 | 0.016216388 | 0.059482036 | 1.875001547 | 6.74E-10 | 1.79E-08 | RAC2 |
| ENSG00000052802 | 0.01514105 | 0.055493837 | 1.873862311 | 1.42E-07 | 2.39E-06 | MSMO1 |
| ENSG00000153551 | 0.010097909 | 0.037002607 | 1.873570348 | 0.000511587 | 0.003129112 | CMTM7 |
| ENSG00000006625 | 0.047144262 | 0.172104383 | 1.868129735 | 2.66E-06 | 3.33E-05 | GGCT |
| ENSG00000251432 | 0.168858893 | 0.614387929 | 1.863331707 | 0.000803721 | 0.004541706 | AC108062.1 |
| ENSG00000111711 | 0.01886162 | 0.068250594 | 1.855388 | 1.57E-08 | 3.23E-07 | GOLT1B |
| ENSG00000064545 | 0.02278238 | 0.082383073 | 1.854429458 | 1.48E-08 | 3.07E-07 | TMEM161A |
| ENSG00000168528 | 0.008582993 | 0.030951076 | 1.850436842 | 2.81E-06 | 3.49E-05 | SERINC2 |
| ENSG00000116521 | 0.015554931 | 0.055744057 | 1.841445998 | 3.30E-14 | 1.67E-12 | SCAMP3 |
| ENSG00000250510 | 0.012949229 | 0.046121948 | 1.832587262 | 0.000790199 | 0.004489502 | GPR162 |
| ENSG00000071967 | 0.014853546 | 0.052776263 | 1.829081863 | 4.44E-16 | 2.89E-14 | CYBRD1 |
| ENSG00000141526 | 0.017707636 | 0.062367782 | 1.816429286 | 0 | 0 | SLC16A3 |
| ENSG00000108691 | 0.023560617 | 0.082966334 | 1.816148739 | 4.53E-07 | 6.84E-06 | CCL2 |
| ENSG00000091947 | 0.024080856 | 0.084461478 | 1.810406832 | 2.29E-08 | 4.57E-07 | TMEM101 |
| ENSG00000100294 | 0.009411567 | 0.032941982 | 1.807420443 | 0.000187569 | 0.001356298 | MCAT |
| ENSG00000121542 | 0.026977257 | 0.094374772 | 1.806657594 | 2.66E-05 | 0.000253255 | SEC22A |
| ENSG00000176809 | 0.221617855 | 0.774984743 | 1.80609379 | 0.000147353 | 0.001105379 | LRRC37A3 |
| ENSG00000164252 | 0.013705489 | 0.047839004 | 1.803433493 | 9.45E-05 | 0.000757723 | AGGF1 |
| ENSG00000160326 | 0.011574234 | 0.040394569 | 1.803244625 | 9.09E-12 | 3.15E-10 | SLC2A6 |
| ENSG00000205544 | 0.182884155 | 0.637786498 | 1.802143472 | 0.001175784 | 0.006220326 | TMEM256 |
| ENSG00000068697 | 0.023512929 | 0.081906687 | 1.800526962 | 3.39E-11 | 1.11E-09 | LAPTM4A |
| ENSG00000180879 | 0.038941303 | 0.135375754 | 1.79759631 | 8.86E-08 | 1.56E-06 | SSR4 |
| ENSG00000105438 | 0.023626497 | 0.082019846 | 1.795567271 | 1.11E-16 | 7.67E-15 | KDELR1 |
| ENSG00000179820 | 0.053907617 | 0.186784199 | 1.792811363 | 0 | 0 | MYADM |
| ENSG00000178896 | 0.016469265 | 0.056961701 | 1.790216035 | 0.001413208 | 0.007237575 | EXOSC4 |
| ENSG00000116918 | 0.011344143 | 0.039219386 | 1.789619287 | 0.000599653 | 0.003581626 | TSNAX |
| ENSG00000162910 | 0.027247576 | 0.094089409 | 1.787904455 | 0.000793338 | 0.004497828 | MRPL55 |
| ENSG00000139722 | 0.041870879 | 0.143996204 | 1.782011681 | 4.12E-07 | 6.26E-06 | VPS37B |
| ENSG00000138061 | 0.015093267 | 0.051873838 | 1.78110205 | 1.05E-14 | 5.74E-13 | CYP1B1 |
| ENSG00000178802 | 0.013533716 | 0.046491271 | 1.780401809 | 4.08E-06 | 4.82E-05 | MPI |
| ENSG00000141349 | 0.011550308 | 0.039650432 | 1.779405213 | 1.34E-05 | 0.000137554 | G6PC3 |
| ENSG00000126709 | 0.017984473 | 0.061585496 | 1.775838742 | 0.000695377 | 0.00403762 | IFI6 |
| ENSG00000095261 | 0.014557092 | 0.049720971 | 1.77213229 | 1.30E-08 | 2.72E-07 | PSMD5 |
| ENSG00000132406 | 0.069287297 | 0.236425236 | 1.770721248 | 1.84E-05 | 0.000182126 | TMEM128 |
| ENSG00000139370 | 0.023670102 | 0.080535642 | 1.766561507 | 9.55E-10 | 2.44E-08 | SLC15A4 |
| ENSG00000197858 | 0.012243247 | 0.041573759 | 1.763686925 | 4.60E-14 | 2.29E-12 | GPAA1 |
| ENSG00000115828 | 0.039924843 | 0.135368199 | 1.761530206 | 9.17E-05 | 0.000737976 | QPCT |
| ENSG00000156171 | 0.011975824 | 0.040570551 | 1.760307921 | 0.002052264 | 0.009816418 | DRAM2 |
| ENSG00000170142 | 0.016605572 | 0.056216576 | 1.759328206 | 3.52E-06 | 4.25E-05 | UBE2E1 |
| ENSG00000128973 | 0.013781821 | 0.046564983 | 1.756478913 | 3.31E-05 | 0.000305164 | CLN6 |
| ENSG00000028839 | 0.020244629 | 0.068389868 | 1.756243397 | 0.000971607 | 0.005301079 | TBPL1 |
| ENSG00000159063 | 0.042959939 | 0.144685383 | 1.751855347 | 1.00E-09 | 2.55E-08 | ALG8 |
| ENSG00000167447 | 0.031573707 | 0.106005753 | 1.747346996 | 9.32E-11 | 2.83E-09 | SMG8 |
| ENSG00000213699 | 0.019495777 | 0.065429949 | 1.746789481 | 2.22E-16 | 1.49E-14 | SLC35F6 |
| ENSG00000176731 | 0.037338585 | 0.125114801 | 1.744513316 | 0.002050034 | 0.009809652 | C8orf59 |
| ENSG00000057252 | 0.048463633 | 0.162184613 | 1.74266249 | 0 | 0 | SOAT1 |
| ENSG00000171960 | 0.039661633 | 0.13211222 | 1.735947941 | 9.88E-05 | 0.000786443 | PPIH |
| ENSG00000213347 | 0.022778046 | 0.075804494 | 1.734639378 | 6.53E-05 | 0.000552622 | MXD3 |
| ENSG00000104408 | 0.025259255 | 0.084048088 | 1.73440289 | 4.49E-08 | 8.49E-07 | EIF3E |
| ENSG00000123374 | 0.021823826 | 0.072493381 | 1.731945195 | 3.83E-10 | 1.05E-08 | CDK2 |
| ENSG00000132423 | 0.025813239 | 0.08552029 | 1.728155561 | 0.001354317 | 0.006986555 | COQ3 |
| ENSG00000050130 | 0.022480801 | 0.074340718 | 1.725459197 | 1.16E-08 | 2.45E-07 | JKAMP |
| ENSG00000139133 | 0.03045993 | 0.100576069 | 1.723302523 | 0.00142822 | 0.007303799 | ALG10 |
| ENSG00000111897 | 0.044798554 | 0.147910165 | 1.723197139 | 1.25E-14 | 6.70E-13 | SERINC1 |
| ENSG00000109084 | 0.011659826 | 0.038440749 | 1.721090159 | 1.52E-05 | 0.000154221 | TMEM97 |
| ENSG00000181513 | 0.016489056 | 0.05419958 | 1.716772911 | 0.000950785 | 0.005211119 | ACBD4 |
| ENSG00000198858 | 0.009366833 | 0.030689104 | 1.712093256 | 1.16E-07 | 1.98E-06 | R3HDM4 |
| ENSG00000204392 | 0.013994436 | 0.045848066 | 1.712007516 | 0.000818774 | 0.004611445 | LSM2 |
| ENSG00000173264 | 0.00806042 | 0.02636908 | 1.709920233 | 0.000282814 | 0.001908442 | GPR137 |
| ENSG00000110108 | 0.019955893 | 0.065211172 | 1.708304285 | 3.93E-12 | 1.45E-10 | TMEM109 |
| ENSG00000112695 | 0.023984465 | 0.078368619 | 1.708175848 | 2.91E-05 | 0.000275255 | COX7A2 |
| ENSG00000102265 | 0.025164917 | 0.082210757 | 1.70791336 | 5.51E-06 | 6.26E-05 | TIMP1 |
| ENSG00000165629 | 0.040159821 | 0.131053308 | 1.706329022 | 8.02E-07 | 1.14E-05 | ATP5F1C |
| ENSG00000119431 | 0.01865445 | 0.060819146 | 1.705005769 | 0.000252763 | 0.001745541 | HDHD3 |
| ENSG00000177700 | 0.04823687 | 0.157145018 | 1.703888345 | 2.08E-07 | 3.35E-06 | POLR2L |
| ENSG00000169446 | 0.013262157 | 0.043185767 | 1.70324043 | 0.000126113 | 0.000969857 | MMGT1 |
| ENSG00000176903 | 0.013488316 | 0.043729864 | 1.696908662 | 5.22E-12 | 1.87E-10 | PNMA1 |
| ENSG00000138092 | 0.092204434 | 0.298762612 | 1.696091588 | 4.12E-12 | 1.51E-10 | CENPO |
| ENSG00000176340 | 0.055472921 | 0.179171778 | 1.691487799 | 3.27E-05 | 0.000302035 | COX8A |
| ENSG00000181830 | 0.010670939 | 0.034233915 | 1.681739161 | 4.25E-06 | 5.00E-05 | SLC35C1 |
| ENSG00000158158 | 0.017411471 | 0.055605606 | 1.675192268 | 0.000879054 | 0.00487575 | CNNM4 |
| ENSG00000105281 | 0.035619048 | 0.113614041 | 1.673420283 | 0 | 0 | SLC1A5 |
| ENSG00000117394 | 0.034793122 | 0.110573516 | 1.668131832 | 0 | 0 | SLC2A1 |
| ENSG00000085719 | 0.029003284 | 0.091451927 | 1.656797278 | 2.00E-15 | 1.21E-13 | CPNE3 |
| ENSG00000104341 | 0.011869149 | 0.037293505 | 1.65170788 | 0.000380006 | 0.002435809 | LAPTM4B |
| ENSG00000198015 | 0.01537366 | 0.048260084 | 1.650369722 | 0.000172686 | 0.001265417 | MRPL42 |
| ENSG00000105193 | 0.021339708 | 0.066951911 | 1.649584779 | 3.60E-06 | 4.33E-05 | RPS16 |
| ENSG00000177058 | 0.037749219 | 0.118244028 | 1.647248632 | 1.18E-08 | 2.48E-07 | SLC38A9 |
| ENSG00000162851 | 0.024783612 | 0.077461272 | 1.64408872 | 4.46E-05 | 0.000393937 | TFB2M |
| ENSG00000111801 | 0.018422276 | 0.057565604 | 1.643755694 | 0.001133894 | 0.006038562 | BTN3A3 |
| ENSG00000078668 | 0.024260751 | 0.075623403 | 1.640208581 | 1.50E-10 | 4.41E-09 | VDAC3 |
| ENSG00000184500 | 0.010407795 | 0.032422494 | 1.639330549 | 6.84E-08 | 1.24E-06 | PROS1 |
| ENSG00000177628 | 0.014772475 | 0.045977672 | 1.638021878 | 0.000265333 | 0.001816986 | GBA |
| ENSG00000083457 | 0.067481653 | 0.21000416 | 1.637850676 | 0.000338205 | 0.002207881 | ITGAE |
| ENSG00000137100 | 0.032752204 | 0.101832657 | 1.636536421 | 6.41E-08 | 1.17E-06 | DCTN3 |
| ENSG00000159208 | 0.029138049 | 0.090394521 | 1.633331039 | 0.00071867 | 0.004140194 | CIART |
| ENSG00000106615 | 0.012562825 | 0.038935926 | 1.631941006 | 8.77E-07 | 1.24E-05 | RHEB |
| ENSG00000137965 | 0.042975676 | 0.133024737 | 1.630102305 | 1.05E-05 | 0.00011153 | IFI44 |
| ENSG00000139624 | 0.02510815 | 0.077561168 | 1.627178799 | 3.88E-09 | 8.92E-08 | CERS5 |
| ENSG00000171488 | 0.027547927 | 0.085022436 | 1.625899819 | 1.84E-05 | 0.000182346 | LRRC8C |
| ENSG00000097046 | 0.007887371 | 0.024324252 | 1.624779127 | 0.000273376 | 0.001857913 | CDC7 |
| ENSG00000075188 | 0.027405663 | 0.08431713 | 1.62135171 | 1.31E-06 | 1.77E-05 | NUP37 |
| ENSG00000184898 | 0.02398208 | 0.073747151 | 1.620630508 | 0.000811236 | 0.004576861 | RBM43 |
| ENSG00000184967 | 0.023509159 | 0.072274165 | 1.620257134 | 7.55E-06 | 8.28E-05 | NOC4L |
| ENSG00000005022 | 0.013091858 | 0.040237405 | 1.619867356 | 1.67E-06 | 2.21E-05 | SLC25A5 |
| ENSG00000105518 | 0.087384598 | 0.268459493 | 1.619253501 | 0.000452554 | 0.002821374 | TMEM205 |
| ENSG00000109854 | 0.025596492 | 0.078555885 | 1.617773242 | 2.22E-05 | 0.000214989 | HTATIP2 |
| ENSG00000100263 | 0.011718386 | 0.035933327 | 1.616548691 | 7.70E-05 | 0.000635218 | RHBDD3 |
| ENSG00000188811 | 0.010156228 | 0.031138671 | 1.616342736 | 6.40E-05 | 0.000542775 | NHLRC3 |
| ENSG00000142444 | 0.027061547 | 0.082878574 | 1.61475488 | 0.000292334 | 0.001960581 | TIMM29 |
| ENSG00000117461 | 0.010375147 | 0.031673774 | 1.610156984 | 0.001675195 | 0.00833784 | PIK3R3 |
| ENSG00000138463 | 0.04510803 | 0.137572781 | 1.608738882 | 8.95E-13 | 3.69E-11 | DIRC2 |
| ENSG00000123989 | 0.013455946 | 0.040997329 | 1.607286103 | 1.48E-12 | 5.98E-11 | CHPF |
| ENSG00000224687 | 0.021709516 | 0.066121726 | 1.60679685 | 0.000727576 | 0.004180175 | RASAL2-AS1 |
| ENSG00000141499 | 0.029991989 | 0.091119893 | 1.603188861 | 8.36E-07 | 1.19E-05 | WRAP53 |
| ENSG00000137331 | 0.034296007 | 0.104160697 | 1.602698471 | 1.99E-08 | 4.03E-07 | IER3 |
| ENSG00000113732 | 0.013854508 | 0.041895442 | 1.5964378 | 1.38E-05 | 0.000141307 | ATP6V0E1 |
| ENSG00000006327 | 0.059281439 | 0.178521456 | 1.590445104 | 0 | 0 | TNFRSF12A |
| ENSG00000112378 | 0.010295439 | 0.030984556 | 1.589543981 | 1.38E-05 | 0.000141394 | PERP |
| ENSG00000105677 | 0.015216869 | 0.045580085 | 1.58273203 | 0.001664027 | 0.008295989 | TMEM147 |
| ENSG00000100258 | 0.027761235 | 0.083058223 | 1.581051229 | 0 | 0 | LMF2 |
| ENSG00000235173 | 0.013441949 | 0.040189611 | 1.580080302 | 4.29E-09 | 9.73E-08 | HGH1 |
| ENSG00000172269 | 0.026962567 | 0.080442402 | 1.576998324 | 1.87E-07 | 3.07E-06 | DPAGT1 |
| ENSG00000109099 | 0.03612076 | 0.107631246 | 1.575196812 | 6.74E-05 | 0.000563682 | PMP22 |
| ENSG00000234745 | 0.015269083 | 0.045445672 | 1.573529527 | 2.47E-06 | 3.14E-05 | HLA-B |
| ENSG00000178252 | 0.016838835 | 0.050074354 | 1.572279529 | 9.40E-06 | 0.000100605 | WDR6 |
| ENSG00000105402 | 0.039559184 | 0.117555746 | 1.571260466 | 5.55E-16 | 3.57E-14 | NAPA |
| ENSG00000102100 | 0.013639379 | 0.040323977 | 1.563859978 | 1.20E-05 | 0.000125695 | SLC35A2 |
| ENSG00000148834 | 0.028959367 | 0.085536239 | 1.562505703 | 0.000373409 | 0.002402486 | GSTO1 |
| ENSG00000226479 | 0.019487681 | 0.057381403 | 1.558020841 | 2.68E-06 | 3.34E-05 | TMEM185B |
| ENSG00000144476 | 0.134872448 | 0.397111687 | 1.557949158 | 0.001431742 | 0.007310701 | ACKR3 |
| ENSG00000176393 | 0.011694098 | 0.034429076 | 1.557846826 | 3.46E-06 | 4.18E-05 | RNPEP |
| ENSG00000152683 | 0.019349967 | 0.056907548 | 1.556288948 | 3.56E-09 | 8.23E-08 | SLC30A6 |
| ENSG00000173085 | 0.019108488 | 0.056194377 | 1.556212134 | 0.000589462 | 0.003530864 | COQ2 |
| ENSG00000146143 | 0.012447273 | 0.036509322 | 1.55243515 | 2.41E-05 | 0.000231511 | PRIM2 |
| ENSG00000134910 | 0.063945766 | 0.187367835 | 1.550952564 | 1.14E-14 | 6.17E-13 | STT3A |
| ENSG00000103512 | 0.003318932 | 0.009721844 | 1.550511142 | 0.000355271 | 0.002300555 | NOMO1 |
| ENSG00000129562 | 0.023148143 | 0.067803279 | 1.550458564 | 2.62E-06 | 3.28E-05 | DAD1 |
| ENSG00000185475 | 0.107987071 | 0.316270348 | 1.550299705 | 0.000173048 | 0.001267295 | TMEM179B |
| ENSG00000131069 | 0.02515352 | 0.073464221 | 1.546281468 | 1.69E-05 | 0.000168459 | ACSS2 |
| ENSG00000229833 | 1.19825497 | 3.499364636 | 1.546158078 | 0.000610616 | 0.003633178 | PET100 |
| ENSG00000254999 | 0.007404839 | 0.021606185 | 1.544904065 | 1.41E-05 | 0.000143692 | BRK1 |
| ENSG00000144659 | 0.018356178 | 0.053518082 | 1.543760705 | 3.58E-05 | 0.000326573 | SLC25A38 |
| ENSG00000113643 | 0.036170826 | 0.105436482 | 1.543475704 | 7.07E-09 | 1.54E-07 | RARS |
| ENSG00000115970 | 0.034834609 | 0.101403303 | 1.541511362 | 2.57E-06 | 3.23E-05 | THADA |
| ENSG00000167925 | 0.015956059 | 0.046427922 | 1.54088832 | 0.001188294 | 0.006267206 | GHDC |
| ENSG00000198131 | 0.013071463 | 0.037988269 | 1.539133323 | 0.000502518 | 0.003083051 | ZNF544 |
| ENSG00000103363 | 0.036759178 | 0.106780264 | 1.538468605 | 0.000105742 | 0.000832262 | ELOB |
| ENSG00000100342 | 0.015421297 | 0.044785235 | 1.538099044 | 5.29E-05 | 0.000456338 | APOL1 |
| ENSG00000163347 | 0.012486993 | 0.036214062 | 1.536123943 | 1.04E-05 | 0.000110391 | CLDN1 |
| ENSG00000140471 | 0.020676998 | 0.059938485 | 1.535455872 | 0.000698351 | 0.004047072 | LINS1 |
| ENSG00000136986 | 0.029097772 | 0.084217676 | 1.533214372 | 4.89E-06 | 5.63E-05 | DERL1 |
| ENSG00000155380 | 0.038848465 | 0.112365714 | 1.532272381 | 2.95E-12 | 1.12E-10 | SLC16A1 |
| ENSG00000160932 | 0.01074593 | 0.031075915 | 1.532006493 | 3.02E-05 | 0.000283488 | LY6E |
| ENSG00000105732 | 0.013938725 | 0.040304998 | 1.531860155 | 0.000111617 | 0.000873258 | ZNF574 |
| ENSG00000156381 | 0.004698917 | 0.013584689 | 1.531581202 | 0.000668724 | 0.003914999 | ANKRD9 |
| ENSG00000099624 | 0.011475451 | 0.033167421 | 1.531215962 | 0.000116259 | 0.00090604 | ATP5F1D |
| ENSG00000079459 | 0.016527768 | 0.047657112 | 1.527799643 | 5.11E-07 | 7.61E-06 | FDFT1 |
| ENSG00000133872 | 0.015806694 | 0.045561703 | 1.527286037 | 0.000795923 | 0.004508228 | SARAF |
| ENSG00000157593 | 0.008112777 | 0.02336944 | 1.526355421 | 1.57E-08 | 3.24E-07 | SLC35B2 |
| ENSG00000127423 | 0.015288308 | 0.044031637 | 1.5261117 | 0.000954672 | 0.005225274 | AUNIP |
| ENSG00000048544 | 0.007531494 | 0.021674614 | 1.524998426 | 0.001907266 | 0.009229324 | MRPS10 |
| ENSG00000138399 | 0.022604303 | 0.065007188 | 1.524001842 | 0.000263419 | 0.001804908 | FASTKD1 |
| ENSG00000065154 | 0.026897526 | 0.077321384 | 1.523394002 | 7.01E-07 | 1.01E-05 | OAT |
| ENSG00000104549 | 0.012736437 | 0.036542651 | 1.520619542 | 6.98E-09 | 1.52E-07 | SQLE |
| ENSG00000068079 | 0.017710434 | 0.05071202 | 1.51772822 | 0.001193434 | 0.006288344 | IFI35 |
| ENSG00000140612 | 0.025431362 | 0.072759666 | 1.516530209 | 3.01E-05 | 0.000282713 | SEC11A |
| ENSG00000179409 | 0.025495185 | 0.072934359 | 1.516373788 | 3.80E-11 | 1.23E-09 | GEMIN4 |
| ENSG00000138029 | 0.017086742 | 0.048768476 | 1.513071504 | 7.43E-06 | 8.16E-05 | HADHB |
| ENSG00000103978 | 0.027107041 | 0.07736469 | 1.513007619 | 3.48E-07 | 5.38E-06 | TMEM87A |
| ENSG00000276045 | 0.008475815 | 0.02417305 | 1.511975495 | 0.000657509 | 0.003870067 | ORAI1 |
| ENSG00000123297 | 0.03100067 | 0.088330463 | 1.510611658 | 0.001250354 | 0.006534896 | TSFM |
| ENSG00000140497 | 0.015985551 | 0.045402585 | 1.506005979 | 8.10E-15 | 4.49E-13 | SCAMP2 |
| ENSG00000102554 | 0.014030163 | 0.03983523 | 1.505513105 | 0.001327708 | 0.006858113 | KLF5 |
| ENSG00000112651 | 0.023934392 | 0.067933751 | 1.505043332 | 0.001487483 | 0.007534532 | MRPL2 |
| ENSG00000128951 | 0.022547363 | 0.063944579 | 1.50386331 | 5.42E-05 | 0.000465897 | DUT |
| ENSG00000196787 | 5.985739024 | 16.9521175 | 1.501864208 | 3.38E-06 | 4.11E-05 | HIST1H2AG |
| ENSG00000080822 | 0.018812805 | 0.053179411 | 1.499152853 | 5.01E-06 | 5.73E-05 | CLDND1 |
| ENSG00000101940 | 0.01160324 | 0.032761785 | 1.497486289 | 0.000114839 | 0.000896133 | WDR13 |
| ENSG00000115446 | 0.031178264 | 0.087986452 | 1.496740785 | 1.88E-06 | 2.46E-05 | UNC50 |
| ENSG00000152661 | 0.019020552 | 0.053660127 | 1.496291376 | 9.88E-05 | 0.000786443 | GJA1 |
| ENSG00000147416 | 0.024963985 | 0.070409694 | 1.495925826 | 0 | 0 | ATP6V1B2 |
| ENSG00000108666 | 0.013333976 | 0.037579372 | 1.494833879 | 0.000984759 | 0.005353403 | C17orf75 |
| ENSG00000105404 | 0.307731311 | 0.865887538 | 1.492508417 | 0.000825388 | 0.004635819 | RABAC1 |
| ENSG00000273611 | 0.023134358 | 0.065079264 | 1.492160888 | 0.000630963 | 0.00373976 | ZNHIT3 |
| ENSG00000125356 | 0.037278222 | 0.104861798 | 1.492084245 | 0.000610065 | 0.0036317 | NDUFA1 |
| ENSG00000106009 | 0.013217991 | 0.037158785 | 1.491200444 | 3.04E-07 | 4.79E-06 | BRAT1 |
| ENSG00000174938 | 0.023732127 | 0.066625116 | 1.489224722 | 6.24E-08 | 1.15E-06 | SEZ6L2 |
| ENSG00000170312 | 0.016801209 | 0.047134947 | 1.48823205 | 3.71E-05 | 0.000335784 | CDK1 |
| ENSG00000126062 | 0.017335077 | 0.048580676 | 1.486688324 | 1.90E-05 | 0.000187562 | TMEM115 |
| ENSG00000168496 | 0.014905488 | 0.041759379 | 1.486256596 | 3.92E-07 | 5.97E-06 | FEN1 |
| ENSG00000124702 | 0.015552614 | 0.04353538 | 1.485031255 | 1.17E-12 | 4.77E-11 | KLHDC3 |
| ENSG00000164815 | 0.029802068 | 0.083317841 | 1.48321303 | 9.06E-05 | 0.000729789 | ORC5 |
| ENSG00000125945 | 0.011550988 | 0.03229207 | 1.48316363 | 0.001591384 | 0.007966855 | ZNF436 |
| ENSG00000198818 | 0.02314201 | 0.064625039 | 1.481579041 | 0.000216635 | 0.001528774 | SFT2D1 |
| ENSG00000017483 | 0.074653469 | 0.208458519 | 1.481479132 | 0.001576066 | 0.00790663 | SLC38A5 |
| ENSG00000169710 | 0.011456277 | 0.031988851 | 1.48143093 | 2.13E-05 | 0.000206885 | FASN |
| ENSG00000169908 | 0.041966028 | 0.11717466 | 1.48136677 | 1.80E-05 | 0.000179013 | TM4SF1 |
| ENSG00000138430 | 0.008964456 | 0.025008987 | 1.480158632 | 0.000718896 | 0.004140194 | OLA1 |
| ENSG00000083099 | 0.011530561 | 0.032131781 | 1.478538202 | 0.00206615 | 0.009871057 | LYRM2 |
| ENSG00000141458 | 0.053042075 | 0.147572632 | 1.476216078 | 1.61E-09 | 3.96E-08 | NPC1 |
| ENSG00000099849 | 0.005455137 | 0.015169408 | 1.475477363 | 0.001210699 | 0.00636027 | RASSF7 |
| ENSG00000149554 | 0.016959067 | 0.047095765 | 1.473540576 | 3.73E-07 | 5.72E-06 | CHEK1 |
| ENSG00000183726 | 0.021577649 | 0.059916285 | 1.47341051 | 4.24E-08 | 8.09E-07 | TMEM50A |
| ENSG00000167900 | 0.014928099 | 0.041425922 | 1.472503347 | 2.93E-08 | 5.78E-07 | TK1 |
| ENSG00000100601 | 0.016023628 | 0.044436882 | 1.471556788 | 0.001249106 | 0.006533493 | ALKBH1 |
| ENSG00000176102 | 0.056387789 | 0.156347128 | 1.471298025 | 5.92E-07 | 8.69E-06 | CSTF3 |
| ENSG00000134824 | 0.0152974 | 0.042391394 | 1.47048492 | 9.28E-10 | 2.37E-08 | FADS2 |
| ENSG00000005448 | 0.026450172 | 0.073292577 | 1.470389994 | 5.44E-06 | 6.20E-05 | WDR54 |
| ENSG00000132432 | 0.034636785 | 0.095735514 | 1.466749172 | 0.000295015 | 0.001973137 | SEC61G |
| ENSG00000102854 | 0.025909828 | 0.071604468 | 1.466550151 | 3.44E-11 | 1.12E-09 | MSLN |
| ENSG00000084073 | 0.037068598 | 0.10233323 | 1.465005245 | 9.76E-12 | 3.37E-10 | ZMPSTE24 |
| ENSG00000106477 | 0.020843067 | 0.057425087 | 1.462113545 | 3.39E-05 | 0.000312227 | CEP41 |
| ENSG00000136930 | 0.027913709 | 0.076829021 | 1.460677519 | 2.15E-06 | 2.77E-05 | PSMB7 |
| ENSG00000187608 | 0.016668888 | 0.045806139 | 1.458383107 | 0.000271921 | 0.00185016 | ISG15 |
| ENSG00000135503 | 0.011811309 | 0.0323823 | 1.455036568 | 0.000171504 | 0.00125906 | ACVR1B |
| ENSG00000184584 | 0.016445304 | 0.044927435 | 1.449921048 | 3.79E-07 | 5.79E-06 | TMEM173 |
| ENSG00000034693 | 0.035467338 | 0.096727904 | 1.447441103 | 1.56E-05 | 0.00015748 | PEX3 |
| ENSG00000143198 | 0.043824958 | 0.1191418 | 1.442855034 | 7.16E-05 | 0.00059434 | MGST3 |
| ENSG00000196177 | 0.017360003 | 0.047172753 | 1.442186622 | 0.000159509 | 0.001186943 | ACADSB |
| ENSG00000106605 | 0.032736572 | 0.088877634 | 1.440917128 | 0.001422666 | 0.007279813 | BLVRA |
| ENSG00000128699 | 0.00978263 | 0.02652684 | 1.439158469 | 0.002009448 | 0.00963846 | ORMDL1 |
| ENSG00000198715 | 0.018084174 | 0.049035472 | 1.439098059 | 6.68E-06 | 7.41E-05 | GLMP |
| ENSG00000103148 | 0.013964562 | 0.037859946 | 1.43890204 | 6.75E-07 | 9.77E-06 | NPRL3 |
| ENSG00000102882 | 0.013192768 | 0.035713143 | 1.43670778 | 1.29E-09 | 3.23E-08 | MAPK3 |
| ENSG00000126453 | 0.010695968 | 0.028894917 | 1.433748682 | 0.000316467 | 0.002095551 | BCL2L12 |
| ENSG00000137404 | 0.01767349 | 0.047702922 | 1.432490726 | 2.91E-06 | 3.59E-05 | NRM |
| ENSG00000254858 | 0.016223301 | 0.043787492 | 1.43245147 | 3.65E-05 | 0.000331716 | MPV17L2 |
| ENSG00000133597 | 0.015333282 | 0.0413716 | 1.431974275 | 0.001073925 | 0.005772886 | ADCK2 |
| ENSG00000113161 | 0.026609104 | 0.071668161 | 1.429412384 | 5.67E-11 | 1.76E-09 | HMGCR |
| ENSG00000145414 | 0.043565793 | 0.117335666 | 1.429373886 | 4.89E-05 | 0.000426973 | NAF1 |
| ENSG00000105223 | 0.016462465 | 0.04429785 | 1.428056267 | 0 | 0 | PLD3 |
| ENSG00000213614 | 0.045480957 | 0.122362546 | 1.427827517 | 1.14E-07 | 1.95E-06 | HEXA |
| ENSG00000115514 | 0.041039385 | 0.110362046 | 1.427163086 | 0.001081396 | 0.005805265 | TXNDC9 |
| ENSG00000185339 | 0.021503912 | 0.057652176 | 1.422775912 | 0.000705493 | 0.004078633 | TCN2 |
| ENSG00000196455 | 0.025742434 | 0.06889749 | 1.420302965 | 1.99E-07 | 3.23E-06 | PIK3R4 |
| ENSG00000163870 | 0.022340893 | 0.059667219 | 1.417251686 | 7.38E-07 | 1.06E-05 | TPRA1 |
| ENSG00000140691 | 0.016172424 | 0.043153793 | 1.415951461 | 0.000801066 | 0.004530958 | ARMC5 |
| ENSG00000130204 | 0.011824733 | 0.031478983 | 1.412581278 | 9.13E-10 | 2.35E-08 | TOMM40 |
| ENSG00000106853 | 0.021423903 | 0.056954421 | 1.410586513 | 1.57E-06 | 2.09E-05 | PTGR1 |
| ENSG00000058804 | 0.018850802 | 0.050101864 | 1.41023835 | 1.11E-12 | 4.56E-11 | NDC1 |
| ENSG00000138600 | 0.055917076 | 0.148408882 | 1.408216622 | 2.50E-09 | 5.92E-08 | SPPL2A |
| ENSG00000134575 | 0.013570455 | 0.035998219 | 1.40745643 | 1.38E-06 | 1.85E-05 | ACP2 |
| ENSG00000072042 | 0.017360715 | 0.046034351 | 1.406884459 | 2.07E-07 | 3.34E-06 | RDH11 |
| ENSG00000116729 | 0.045401548 | 0.120318876 | 1.406049609 | 3.00E-15 | 1.76E-13 | WLS |
| ENSG00000162729 | 0.009516353 | 0.025115766 | 1.400112616 | 0.000317672 | 0.002101212 | IGSF8 |
| ENSG00000168003 | 0.035088765 | 0.092466277 | 1.397918138 | 2.66E-12 | 1.03E-10 | SLC3A2 |
| ENSG00000173653 | 0.019256706 | 0.050722099 | 1.397253472 | 0.000291368 | 0.00195519 | RCE1 |
| ENSG00000090432 | 0.0145523 | 0.038252686 | 1.394313886 | 1.77E-06 | 2.33E-05 | MUL1 |
| ENSG00000084754 | 0.025379856 | 0.066683654 | 1.393649266 | 4.46E-14 | 2.24E-12 | HADHA |
| ENSG00000125450 | 0.031626088 | 0.083093641 | 1.393622967 | 5.88E-15 | 3.32E-13 | NUP85 |
| ENSG00000159131 | 0.028241863 | 0.074172447 | 1.393048082 | 0 | 0 | GART |
| ENSG00000178927 | 0.007949044 | 0.020863785 | 1.39214759 | 4.75E-07 | 7.13E-06 | CYBC1 |
| ENSG00000167325 | 0.034555829 | 0.090654064 | 1.391442604 | 1.53E-13 | 7.10E-12 | RRM1 |
| ENSG00000181104 | 0.021253189 | 0.055716892 | 1.390435463 | 3.28E-12 | 1.24E-10 | F2R |
| ENSG00000164073 | 0.030130225 | 0.078807749 | 1.387126042 | 0.00010459 | 0.000825801 | MFSD8 |
| ENSG00000178719 | 0.017045516 | 0.044569009 | 1.386648645 | 2.54E-06 | 3.20E-05 | GRINA |
| ENSG00000064601 | 0.018639566 | 0.048722544 | 1.38622118 | 0 | 0 | CTSA |
| ENSG00000152700 | 0.021823352 | 0.056967869 | 1.384275741 | 6.76E-10 | 1.79E-08 | SAR1B |
| ENSG00000178982 | 0.029126786 | 0.076018794 | 1.384009607 | 0.000159894 | 0.001189071 | EIF3K |
| ENSG00000143476 | 0.022678761 | 0.059104395 | 1.381923599 | 5.90E-13 | 2.52E-11 | DTL |
| ENSG00000030582 | 0.019749049 | 0.051455495 | 1.381541998 | 1.04E-14 | 5.70E-13 | GRN |
| ENSG00000125304 | 0.057903714 | 0.150813706 | 1.381039766 | 1.84E-14 | 9.72E-13 | TM9SF2 |
| ENSG00000005812 | 0.013389098 | 0.034849916 | 1.380096383 | 0.002064357 | 0.009866413 | FBXL3 |
| ENSG00000214063 | 0.020910935 | 0.054194397 | 1.373886161 | 3.82E-12 | 1.42E-10 | TSPAN4 |
| ENSG00000115207 | 0.024129143 | 0.062404853 | 1.370881572 | 2.67E-09 | 6.29E-08 | GTF3C2 |
| ENSG00000183624 | 0.01280066 | 0.03304295 | 1.368124322 | 0.000102605 | 0.000814408 | HMCES |
| ENSG00000173638 | 0.025242368 | 0.065061827 | 1.365964087 | 2.66E-06 | 3.32E-05 | SLC19A1 |
| ENSG00000112514 | 0.024397846 | 0.062836335 | 1.364845266 | 0.000465822 | 0.002888862 | CUTA |
| ENSG00000010256 | 0.027739144 | 0.071427593 | 1.364558226 | 1.55E-06 | 2.06E-05 | UQCRC1 |
| ENSG00000130479 | 0.020836616 | 0.053592465 | 1.362909164 | 0.000740669 | 0.004245257 | MAP1S |
| ENSG00000139324 | 0.034648367 | 0.0890325 | 1.361544713 | 4.50E-13 | 2.00E-11 | TMTC3 |
| ENSG00000131981 | 0.021326106 | 0.054787311 | 1.361221241 | 9.67E-05 | 0.000772682 | LGALS3 |
| ENSG00000111640 | 0.013276344 | 0.034104599 | 1.361108329 | 3.62E-11 | 1.17E-09 | GAPDH |
| ENSG00000169299 | 0.024187627 | 0.062068077 | 1.359582211 | 4.01E-07 | 6.10E-06 | PGM2 |
| ENSG00000168002 | 0.02102393 | 0.05389711 | 1.358175525 | 0.001485457 | 0.007533793 | POLR2G |
| ENSG00000213853 | 0.013295316 | 0.034061562 | 1.357226577 | 0.000952582 | 0.005218586 | EMP2 |
| ENSG00000132591 | 0.014103627 | 0.036093413 | 1.355669317 | 1.34E-05 | 0.000137554 | ERAL1 |
| ENSG00000120333 | 0.029143248 | 0.07453519 | 1.35476007 | 0.001013462 | 0.005487113 | MRPS14 |
| ENSG00000147955 | 0.012366621 | 0.031616648 | 1.354233122 | 1.43E-09 | 3.55E-08 | SIGMAR1 |
| ENSG00000187522 | 0.027379495 | 0.069988271 | 1.354017303 | 0.001036764 | 0.00559313 | HSPA14 |
| ENSG00000163541 | 0.022671836 | 0.057940763 | 1.35367746 | 4.92E-05 | 0.000429096 | SUCLG1 |
| ENSG00000156535 | 0.02967632 | 0.075840727 | 1.353660586 | 5.62E-06 | 6.37E-05 | CD109 |
| ENSG00000023191 | 0.023760932 | 0.060708727 | 1.353312488 | 3.86E-13 | 1.73E-11 | RNH1 |
| ENSG00000278828 | 10.58855657 | 27.02246857 | 1.35165354 | 0.00018904 | 0.00136611 | HIST1H3H |
| ENSG00000067167 | 0.024249299 | 0.061858743 | 1.351034445 | 0 | 0 | TRAM1 |
| ENSG00000174021 | 0.240246066 | 0.612528465 | 1.350264082 | 0.000792591 | 0.004496275 | GNG5 |
| ENSG00000103066 | 0.027382292 | 0.069776492 | 1.349497857 | 2.60E-06 | 3.26E-05 | PLA2G15 |
| ENSG00000135926 | 0.015327373 | 0.039000798 | 1.347393242 | 1.31E-10 | 3.89E-09 | TMBIM1 |
| ENSG00000155463 | 0.022141123 | 0.056298134 | 1.346358743 | 4.79E-12 | 1.74E-10 | OXA1L |
| ENSG00000138074 | 0.031795783 | 0.080733064 | 1.344324222 | 6.50E-08 | 1.19E-06 | SLC5A6 |
| ENSG00000013297 | 0.014713821 | 0.037301371 | 1.342056686 | 2.87E-06 | 3.55E-05 | CLDN11 |
| ENSG00000186480 | 0.007301239 | 0.018360098 | 1.330360651 | 6.91E-05 | 0.000575252 | INSIG1 |
| ENSG00000160752 | 0.037200363 | 0.09333759 | 1.327141517 | 6.83E-08 | 1.24E-06 | FDPS |
| ENSG00000119977 | 0.010218758 | 0.025624843 | 1.326323376 | 0.000140964 | 0.00106476 | TCTN3 |
| ENSG00000112759 | 0.026313266 | 0.065973821 | 1.326103343 | 5.12E-11 | 1.62E-09 | SLC29A1 |
| ENSG00000126432 | 0.017685435 | 0.0443223 | 1.325471031 | 0.000916952 | 0.005051005 | PRDX5 |
| ENSG00000010278 | 0.042668484 | 0.106919557 | 1.325283012 | 1.42E-06 | 1.90E-05 | CD9 |
| ENSG00000159377 | 0.037197062 | 0.093178335 | 1.324805863 | 3.67E-05 | 0.000332751 | PSMB4 |
| ENSG00000120437 | 0.028037126 | 0.070127313 | 1.322637997 | 3.40E-06 | 4.12E-05 | ACAT2 |
| ENSG00000006451 | 0.008897464 | 0.022242854 | 1.321875842 | 0.000212773 | 0.001504169 | RALA |
| ENSG00000163702 | 0.01321096 | 0.033021885 | 1.321687155 | 0.000181176 | 0.001318789 | IL17RC |
| ENSG00000143771 | 0.022481421 | 0.056168515 | 1.321028467 | 6.88E-05 | 0.000573107 | CNIH4 |
| ENSG00000164951 | 0.01344857 | 0.033576347 | 1.319992517 | 3.99E-10 | 1.09E-08 | PDP1 |
| ENSG00000178467 | 0.02764905 | 0.068962965 | 1.318591879 | 2.18E-09 | 5.21E-08 | P4HTM |
| ENSG00000175782 | 0.018919208 | 0.047010405 | 1.313128404 | 3.77E-06 | 4.51E-05 | SLC35E3 |
| ENSG00000183155 | 0.031894833 | 0.079223753 | 1.312610326 | 3.39E-06 | 4.11E-05 | RABIF |
| ENSG00000137628 | 0.022943218 | 0.056894579 | 1.31022347 | 0.000114157 | 0.000891385 | DDX60 |
| ENSG00000086062 | 0.016415676 | 0.040699387 | 1.30993293 | 1.11E-16 | 7.67E-15 | B4GALT1 |
| ENSG00000240849 | 0.016990334 | 0.042113061 | 1.309553534 | 0.000385656 | 0.002467455 | TMEM189 |
| ENSG00000077721 | 0.019621884 | 0.048630973 | 1.309411887 | 0.000228071 | 0.001597296 | UBE2A |
| ENSG00000102007 | 0.014500365 | 0.035933474 | 1.309239204 | 2.03E-08 | 4.09E-07 | PLP2 |
| ENSG00000067704 | 0.016208637 | 0.040158025 | 1.308925512 | 6.20E-07 | 9.06E-06 | IARS2 |
| ENSG00000164040 | 0.032435603 | 0.080312827 | 1.308052173 | 1.28E-05 | 0.000132731 | PGRMC2 |
| ENSG00000171135 | 0.016770501 | 0.041520182 | 1.307886976 | 0.001280835 | 0.00667044 | JAGN1 |
| ENSG00000139641 | 0.036326897 | 0.089814602 | 1.305911899 | 0 | 0 | ESYT1 |
| ENSG00000147155 | 0.02183151 | 0.053951364 | 1.305247535 | 0.000134098 | 0.001020586 | EBP |
| ENSG00000106080 | 0.014262986 | 0.035212672 | 1.3038186 | 0.000922726 | 0.005075838 | FKBP14 |
| ENSG00000149761 | 0.235426988 | 0.581040837 | 1.303359849 | 6.38E-06 | 7.10E-05 | NUDT22 |
| ENSG00000174851 | 0.016200961 | 0.039702546 | 1.29315219 | 0.000547973 | 0.00332461 | YIF1A |
| ENSG00000166710 | 0.018592529 | 0.04547777 | 1.290438472 | 0.000118254 | 0.000919204 | B2M |
| ENSG00000063241 | 0.011395421 | 0.027863156 | 1.289904431 | 0.000854149 | 0.004766193 | ISOC2 |
| ENSG00000116221 | 0.011655941 | 0.028495434 | 1.289665325 | 0.000127761 | 0.000981054 | MRPL37 |
| ENSG00000138071 | 0.017139893 | 0.041882404 | 1.288986108 | 0 | 0 | ACTR2 |
| ENSG00000138459 | 0.021591533 | 0.052697535 | 1.287269775 | 3.09E-05 | 0.000288585 | SLC35A5 |
| ENSG00000166189 | 0.011996119 | 0.029269127 | 1.286811967 | 0.000497387 | 0.003059374 | HPS6 |
| ENSG00000115091 | 0.02443085 | 0.059608007 | 1.286802077 | 3.47E-10 | 9.62E-09 | ACTR3 |
| ENSG00000073578 | 0.014427824 | 0.035147985 | 1.284588317 | 3.42E-11 | 1.12E-09 | SDHA |
| ENSG00000172809 | 0.040684512 | 0.099081285 | 1.284132899 | 0.00020545 | 0.001458404 | RPL38 |
| ENSG00000008394 | 0.026757245 | 0.065156821 | 1.283986652 | 0.000427118 | 0.002687645 | MGST1 |
| ENSG00000173113 | 0.023007373 | 0.056017344 | 1.283777326 | 0.002026519 | 0.009708722 | TRMT112 |
| ENSG00000164169 | 0.042214684 | 0.102743949 | 1.283236589 | 3.44E-05 | 0.000314861 | PRMT9 |
| ENSG00000149084 | 0.026156559 | 0.063622534 | 1.282365079 | 0.000598426 | 0.003576577 | HSD17B12 |
| ENSG00000140391 | 0.010464789 | 0.025408378 | 1.279761132 | 0.000239362 | 0.001665698 | TSPAN3 |
| ENSG00000177674 | 0.020037897 | 0.04858939 | 1.277910237 | 0.000703205 | 0.004067358 | AGTRAP |
| ENSG00000117335 | 0.006333999 | 0.015353194 | 1.277350184 | 0.000153802 | 0.001148027 | CD46 |
| ENSG00000171311 | 0.016723316 | 0.040482133 | 1.275424328 | 0.001776297 | 0.008736184 | EXOSC1 |
| ENSG00000165672 | 0.044137542 | 0.106798953 | 1.274819318 | 5.72E-05 | 0.000490143 | PRDX3 |
| ENSG00000122692 | 0.023512896 | 0.056887229 | 1.274652554 | 6.31E-07 | 9.21E-06 | SMU1 |
| ENSG00000105723 | 0.012481372 | 0.030158279 | 1.272777587 | 3.27E-06 | 3.99E-05 | GSK3A |
| ENSG00000148484 | 0.024937055 | 0.060241717 | 1.272471803 | 7.94E-09 | 1.71E-07 | RSU1 |
| ENSG00000108946 | 0.023360173 | 0.056382751 | 1.271202919 | 0 | 0 | PRKAR1A |
| ENSG00000100462 | 0.057442497 | 0.138633533 | 1.271085905 | 5.96E-07 | 8.73E-06 | PRMT5 |
| ENSG00000138942 | 0.018239856 | 0.043933626 | 1.268231196 | 1.12E-05 | 0.000118395 | RNF185 |
| ENSG00000129255 | 0.035123767 | 0.08456194 | 1.26756088 | 6.62E-05 | 0.000557719 | MPDU1 |
| ENSG00000133318 | 0.023995946 | 0.057749503 | 1.267017811 | 1.13E-05 | 0.000119329 | RTN3 |
| ENSG00000117592 | 0.017513689 | 0.042134083 | 1.266504737 | 3.09E-05 | 0.000288585 | PRDX6 |
| ENSG00000115649 | 0.022138213 | 0.05316259 | 1.263872577 | 1.59E-07 | 2.65E-06 | CNPPD1 |
| ENSG00000177054 | 0.023858554 | 0.057245413 | 1.262653509 | 1.53E-05 | 0.000155417 | ZDHHC13 |
| ENSG00000136856 | 0.011782575 | 0.028259572 | 1.262084769 | 0.000914961 | 0.005042349 | SLC2A8 |
| ENSG00000177733 | 0.024675 | 0.059171433 | 1.261850732 | 1.06E-08 | 2.24E-07 | HNRNPA0 |
| ENSG00000137055 | 0.026925876 | 0.064517444 | 1.260696009 | 7.17E-06 | 7.90E-05 | PLAA |
| ENSG00000172270 | 0.043064901 | 0.10315983 | 1.260296877 | 8.33E-15 | 4.59E-13 | BSG |
| ENSG00000145495 | 0.052241969 | 0.12486457 | 1.257082998 | 8.55E-11 | 2.61E-09 | 6-Mar |
| ENSG00000136021 | 0.012926868 | 0.030876376 | 1.256130683 | 3.42E-07 | 5.31E-06 | SCYL2 |
| ENSG00000177613 | 0.017756535 | 0.042402622 | 1.255803425 | 1.58E-05 | 0.000158851 | CSTF2T |
| ENSG00000204387 | 0.062555838 | 0.149170599 | 1.253746772 | 0.000299848 | 0.001999815 | C6orf48 |
| ENSG00000157224 | 0.014716182 | 0.035065847 | 1.252663186 | 0.000932214 | 0.005121001 | CLDN12 |
| ENSG00000188229 | 0.014742335 | 0.0351211 | 1.252372973 | 4.52E-09 | 1.02E-07 | TUBB4B |
| ENSG00000240065 | 0.013688542 | 0.032596581 | 1.25175182 | 0.000838589 | 0.004694613 | PSMB9 |
| ENSG00000108784 | 0.024563482 | 0.058488488 | 1.251637604 | 3.90E-05 | 0.000351038 | NAGLU |
| ENSG00000172053 | 0.048955191 | 0.116525096 | 1.25110694 | 1.11E-16 | 7.67E-15 | QARS |
| ENSG00000075415 | 0.016418034 | 0.039068983 | 1.250742288 | 1.82E-05 | 0.00018067 | SLC25A3 |
| ENSG00000177917 | 0.025514818 | 0.060678075 | 1.249839969 | 4.66E-05 | 0.000409072 | ARL6IP6 |
| ENSG00000086475 | 0.007824396 | 0.018603733 | 1.24954086 | 0.000143226 | 0.001077779 | SEPHS1 |
| ENSG00000100162 | 0.036254748 | 0.086162731 | 1.24889403 | 0.000640313 | 0.003784864 | CENPM |
| ENSG00000214517 | 0.024555759 | 0.058331576 | 1.248215611 | 1.27E-09 | 3.20E-08 | PPME1 |
| ENSG00000204394 | 0.025677469 | 0.060882377 | 1.245521663 | 3.47E-10 | 9.62E-09 | VARS |
| ENSG00000125691 | 0.014770853 | 0.0350005 | 1.244622338 | 8.22E-05 | 0.00067227 | RPL23 |
| ENSG00000066322 | 0.021485522 | 0.05084708 | 1.242800103 | 1.81E-10 | 5.28E-09 | ELOVL1 |
| ENSG00000103642 | 0.024028828 | 0.056817256 | 1.241562871 | 4.40E-05 | 0.000389966 | LACTB |
| ENSG00000130733 | 0.023699542 | 0.05601021 | 1.240830645 | 0.000197344 | 0.001413375 | YIPF2 |
| ENSG00000134333 | 0.028327985 | 0.066850756 | 1.238715879 | 3.37E-08 | 6.56E-07 | LDHA |
| ENSG00000135956 | 0.012721027 | 0.030010205 | 1.238238039 | 7.87E-07 | 1.13E-05 | TMEM127 |
| ENSG00000221823 | 0.025518233 | 0.060172259 | 1.237570093 | 0.000411615 | 0.002613341 | PPP3R1 |
| ENSG00000100347 | 0.059902457 | 0.141184608 | 1.23689574 | 1.58E-05 | 0.000158851 | SAMM50 |
| ENSG00000138085 | 0.032877286 | 0.077384425 | 1.23495202 | 0.000662092 | 0.003891329 | ATRAID |
| ENSG00000139644 | 0.026535652 | 0.062370399 | 1.232929468 | 0 | 0 | TMBIM6 |
| ENSG00000167815 | 0.048576893 | 0.114103925 | 1.232006292 | 7.55E-05 | 0.000624205 | PRDX2 |
| ENSG00000132128 | 0.018071631 | 0.042421481 | 1.231068286 | 1.98E-06 | 2.58E-05 | LRRC41 |
| ENSG00000171241 | 0.0260053 | 0.061021704 | 1.230516794 | 4.65E-08 | 8.77E-07 | SHCBP1 |
| ENSG00000164144 | 0.025802372 | 0.060508839 | 1.229642191 | 0.001055515 | 0.005684088 | ARFIP1 |
| ENSG00000074696 | 0.028955436 | 0.067761378 | 1.226629019 | 7.15E-10 | 1.88E-08 | HACD3 |
| ENSG00000117318 | 0.042597564 | 0.099680391 | 1.226538813 | 0.001545972 | 0.007783434 | ID3 |
| ENSG00000143368 | 0.014182394 | 0.03317986 | 1.226206722 | 8.67E-12 | 3.01E-10 | SF3B4 |
| ENSG00000118855 | 0.02053933 | 0.048036877 | 1.225753192 | 0.000101892 | 0.000809279 | MFSD1 |
| ENSG00000118246 | 0.03748673 | 0.087565807 | 1.223987642 | 0.000378317 | 0.002428141 | FASTKD2 |
| ENSG00000115159 | 0.013078755 | 0.030546056 | 1.223760934 | 0.000252868 | 0.001745541 | GPD2 |
| ENSG00000136240 | 0.028236374 | 0.065941558 | 1.22363314 | 1.79E-06 | 2.35E-05 | KDELR2 |
| ENSG00000115520 | 0.035082458 | 0.081928188 | 1.223610051 | 0.00019843 | 0.001418619 | COQ10B |
| ENSG00000221983 | 0.022605758 | 0.052772778 | 1.223103646 | 0.000506882 | 0.003106655 | UBA52 |
| ENSG00000172893 | 0.018541438 | 0.043182768 | 1.219702614 | 8.66E-05 | 0.000701961 | DHCR7 |
| ENSG00000158402 | 0.02981673 | 0.069361537 | 1.218013828 | 0.000266014 | 0.001819576 | CDC25C |
| ENSG00000143819 | 0.03004178 | 0.069731968 | 1.214849902 | 1.94E-06 | 2.53E-05 | EPHX1 |
| ENSG00000143815 | 0.020691793 | 0.04800075 | 1.213998292 | 1.52E-07 | 2.56E-06 | LBR |
| ENSG00000165006 | 0.022969686 | 0.053282839 | 1.2139398 | 3.17E-06 | 3.87E-05 | UBAP1 |
| ENSG00000185825 | 0.033408025 | 0.077417406 | 1.212463279 | 2.45E-06 | 3.12E-05 | BCAP31 |
| ENSG00000109919 | 0.025794112 | 0.059755863 | 1.212038505 | 0.00017734 | 0.001294788 | MTCH2 |
| ENSG00000165782 | 0.012964031 | 0.030026887 | 1.211740593 | 0.000346338 | 0.002253632 | PIP4P1 |
| ENSG00000204592 | 0.020080828 | 0.046487887 | 1.21103607 | 1.16E-10 | 3.48E-09 | HLA-E |
| ENSG00000157637 | 0.012836912 | 0.029686689 | 1.209517999 | 7.52E-07 | 1.08E-05 | SLC38A10 |
| ENSG00000124641 | 0.02170285 | 0.050083945 | 1.206463664 | 0.001845612 | 0.00899979 | MED20 |
| ENSG00000101474 | 0.017447223 | 0.040225488 | 1.205112471 | 4.78E-10 | 1.29E-08 | APMAP |
| ENSG00000159111 | 0.011561904 | 0.026655147 | 1.205035139 | 0.000199097 | 0.001421699 | MRPL10 |
| ENSG00000167695 | 0.015353964 | 0.035383176 | 1.204452402 | 5.20E-06 | 5.95E-05 | FAM57A |
| ENSG00000135486 | 0.005911871 | 0.0136011 | 1.202036501 | 3.31E-07 | 5.16E-06 | HNRNPA1 |
| ENSG00000111481 | 0.021761457 | 0.050064119 | 1.202001827 | 2.39E-05 | 0.000229633 | COPZ1 |
| ENSG00000156642 | 0.027505645 | 0.063277323 | 1.201960836 | 1.19E-06 | 1.63E-05 | NPTN |
| ENSG00000132824 | 0.023512146 | 0.05406183 | 1.201204114 | 7.35E-12 | 2.59E-10 | SERINC3 |
| ENSG00000114902 | 0.018496467 | 0.042479156 | 1.199505395 | 0.001671043 | 0.008320619 | SPCS1 |
| ENSG00000175334 | 0.023867315 | 0.054802703 | 1.199210767 | 0.000206176 | 0.00146269 | BANF1 |
| ENSG00000137700 | 0.023680086 | 0.054349704 | 1.198597879 | 1.84E-06 | 2.41E-05 | SLC37A4 |
| ENSG00000105135 | 0.020313843 | 0.046530741 | 1.195720966 | 3.07E-07 | 4.81E-06 | ILVBL |
| ENSG00000087008 | 0.041515087 | 0.095013122 | 1.194491051 | 0.000960798 | 0.005251355 | ACOX3 |
| ENSG00000156261 | 0.032318909 | 0.073942624 | 1.194027751 | 4.24E-05 | 0.000377671 | CCT8 |
| ENSG00000136826 | 0.015471577 | 0.03539277 | 1.193834394 | 0.0018287 | 0.008946348 | KLF4 |
| ENSG00000125505 | 0.015903499 | 0.036338271 | 1.192145533 | 2.13E-10 | 6.19E-09 | MBOAT7 |
| ENSG00000149547 | 0.007969427 | 0.018193382 | 1.190865855 | 1.05E-07 | 1.81E-06 | EI24 |
| ENSG00000040531 | 0.031641125 | 0.07220363 | 1.190270464 | 0.00168812 | 0.008391185 | CTNS |
| ENSG00000155304 | 0.028602767 | 0.06521686 | 1.189090272 | 1.68E-08 | 3.44E-07 | HSPA13 |
| ENSG00000167113 | 0.017260342 | 0.039301252 | 1.187114255 | 0.000223965 | 0.00157312 | COQ4 |
| ENSG00000269556 | 0.028774687 | 0.065424039 | 1.185020623 | 4.40E-05 | 0.000389966 | TMEM185A |
| ENSG00000046604 | 0.016444119 | 0.03731942 | 1.182354851 | 2.99E-05 | 0.000281841 | DSG2 |
| ENSG00000115275 | 0.01171298 | 0.026563373 | 1.181330167 | 1.30E-05 | 0.000134271 | MOGS |
| ENSG00000140545 | 0.015956614 | 0.036116816 | 1.178516212 | 0.000308693 | 0.002051981 | MFGE8 |
| ENSG00000167118 | 0.023314587 | 0.052722959 | 1.177198465 | 0.000435254 | 0.002728848 | URM1 |
| ENSG00000084207 | 0.022031379 | 0.049797787 | 1.176521823 | 0.000558192 | 0.003372996 | GSTP1 |
| ENSG00000108479 | 0.015762968 | 0.035586088 | 1.17477409 | 0.001583747 | 0.007941848 | GALK1 |
| ENSG00000101079 | 0.020121619 | 0.045279299 | 1.170105235 | 5.01E-07 | 7.47E-06 | NDRG3 |
| ENSG00000104635 | 0.019779822 | 0.044475555 | 1.168983177 | 9.99E-16 | 6.26E-14 | SLC39A14 |
| ENSG00000111229 | 0.023885836 | 0.053646856 | 1.167338232 | 0.001451293 | 0.007394626 | ARPC3 |
| ENSG00000047932 | 0.034097045 | 0.076576402 | 1.167253164 | 5.86E-06 | 6.62E-05 | GOPC |
| ENSG00000134531 | 0.015182403 | 0.034006809 | 1.163423482 | 1.80E-06 | 2.36E-05 | EMP1 |
| ENSG00000063177 | 0.013265514 | 0.029687941 | 1.162196413 | 0.000906041 | 0.005000065 | RPL18 |
| ENSG00000125124 | 0.019988792 | 0.044708158 | 1.161346815 | 0.000254852 | 0.001755209 | BBS2 |
| ENSG00000105355 | 0.024236482 | 0.054122705 | 1.159053649 | 0 | 0 | PLIN3 |
| ENSG00000141367 | 0.043718905 | 0.097570497 | 1.158187708 | 0 | 0 | CLTC |
| ENSG00000163162 | 0.02140311 | 0.047748967 | 1.157649041 | 0.001758141 | 0.00866461 | RNF149 |
| ENSG00000100387 | 0.033408229 | 0.074467534 | 1.156408101 | 0.001944314 | 0.009378386 | RBX1 |
| ENSG00000163527 | 0.027683508 | 0.061705565 | 1.156373864 | 0 | 0 | STT3B |
| ENSG00000167779 | 0.015558803 | 0.034670429 | 1.155974657 | 0.001478027 | 0.007508778 | IGFBP6 |
| ENSG00000162384 | 0.038155409 | 0.085015326 | 1.155835358 | 0.000198825 | 0.001420599 | C1orf123 |
| ENSG00000213625 | 0.012866186 | 0.028592286 | 1.152041471 | 3.02E-05 | 0.00028332 | LEPROT |
| ENSG00000101146 | 0.028069084 | 0.062375443 | 1.151996192 | 0.000763222 | 0.004351705 | RAE1 |
| ENSG00000163918 | 0.034505439 | 0.076627703 | 1.151042262 | 0.001943171 | 0.009377563 | RFC4 |
| ENSG00000130726 | 0.018702577 | 0.041489476 | 1.149508401 | 2.72E-14 | 1.39E-12 | TRIM28 |
| ENSG00000213949 | 0.032350481 | 0.071715384 | 1.148495483 | 0.001002814 | 0.005441715 | ITGA1 |
| ENSG00000087088 | 0.029757028 | 0.065852061 | 1.145998169 | 0.000828073 | 0.004646562 | BAX |
| ENSG00000197296 | 0.021903879 | 0.048471919 | 1.145962843 | 8.83E-05 | 0.000713885 | FITM2 |
| ENSG00000165282 | 0.037781224 | 0.083458708 | 1.14339313 | 0.000102821 | 0.00081451 | PIGO |
| ENSG00000145741 | 0.030095403 | 0.066427905 | 1.142246266 | 0.001319982 | 0.006838767 | BTF3 |
| ENSG00000159403 | 0.022651572 | 0.049990716 | 1.142048987 | 0.000802212 | 0.004535307 | C1R |
| ENSG00000138175 | 0.027364078 | 0.060370036 | 1.141549393 | 0.000597813 | 0.003574693 | ARL3 |
| ENSG00000167085 | 0.014600559 | 0.032158649 | 1.139183183 | 7.87E-08 | 1.40E-06 | PHB |
| ENSG00000131435 | 0.01506455 | 0.033178977 | 1.139111824 | 0.000696149 | 0.004038489 | PDLIM4 |
| ENSG00000109501 | 0.03454109 | 0.07601814 | 1.138030128 | 1.76E-07 | 2.90E-06 | WFS1 |
| ENSG00000185896 | 0.030266016 | 0.066604376 | 1.137918172 | 9.99E-16 | 6.26E-14 | LAMP1 |
| ENSG00000071553 | 0.016146088 | 0.035494483 | 1.136410187 | 4.59E-11 | 1.47E-09 | ATP6AP1 |
| ENSG00000166986 | 0.047986861 | 0.10530549 | 1.133869305 | 1.72E-12 | 6.86E-11 | MARS |
| ENSG00000136235 | 0.03238155 | 0.071008309 | 1.132815783 | 8.58E-10 | 2.21E-08 | GPNMB |
| ENSG00000135624 | 0.036902746 | 0.080909977 | 1.132589423 | 6.66E-05 | 0.000559291 | CCT7 |
| ENSG00000012660 | 0.020336789 | 0.044565469 | 1.131834389 | 1.15E-07 | 1.98E-06 | ELOVL5 |
| ENSG00000055211 | 0.022997796 | 0.050329095 | 1.129897063 | 0.001451868 | 0.007394626 | GINM1 |
| ENSG00000144746 | 0.026597354 | 0.058160894 | 1.128766691 | 0.001454667 | 0.007405744 | ARL6IP5 |
| ENSG00000163661 | 0.022076888 | 0.048166082 | 1.125480722 | 0.001724917 | 0.008539368 | PTX3 |
| ENSG00000168394 | 0.016471716 | 0.035928833 | 1.125151203 | 4.22E-07 | 6.39E-06 | TAP1 |
| ENSG00000104299 | 0.036704727 | 0.080052862 | 1.124987108 | 0.001584794 | 0.007942796 | INTS9 |
| ENSG00000071462 | 0.022210446 | 0.048438155 | 1.12490556 | 0.000820848 | 0.004616788 | BUD23 |
| ENSG00000104660 | 0.015443518 | 0.033660139 | 1.124039682 | 0.00088763 | 0.004918526 | LEPROTL1 |
| ENSG00000089597 | 0.034783229 | 0.075809399 | 1.123984867 | 3.75E-07 | 5.73E-06 | GANAB |
| ENSG00000124762 | 0.02180946 | 0.0475233 | 1.123680944 | 8.10E-07 | 1.15E-05 | CDKN1A |
| ENSG00000164930 | 0.020811858 | 0.045346704 | 1.123591902 | 3.24E-05 | 0.000300887 | FZD6 |
| ENSG00000198743 | 0.036778652 | 0.080070956 | 1.122410438 | 3.42E-05 | 0.000313487 | SLC5A3 |
| ENSG00000116747 | 0.044299393 | 0.096427114 | 1.122151922 | 1.86E-08 | 3.79E-07 | TROVE2 |
| ENSG00000109107 | 0.030696384 | 0.066793878 | 1.121647172 | 0.000497651 | 0.003059433 | ALDOC |
| ENSG00000133119 | 0.02408514 | 0.052298521 | 1.118626819 | 3.42E-05 | 0.000313544 | RFC3 |
| ENSG00000139880 | 0.009626123 | 0.020898845 | 1.118396471 | 0.000146846 | 0.001102264 | CDH24 |
| ENSG00000120798 | 0.028137464 | 0.060987171 | 1.116013491 | 0.000370983 | 0.00238943 | NR2C1 |
| ENSG00000162341 | 0.038480615 | 0.083303656 | 1.114247961 | 9.85E-05 | 0.000785238 | TPCN2 |
| ENSG00000196305 | 0.037035445 | 0.080135899 | 1.113542023 | 0 | 0 | IARS |
| ENSG00000137509 | 0.0277871 | 0.060057877 | 1.111938168 | 6.63E-05 | 0.000557719 | PRCP |
| ENSG00000142751 | 0.014871491 | 0.032118328 | 1.110847462 | 0.000427788 | 0.002689047 | GPN2 |
| ENSG00000067560 | 0.022115203 | 0.047710754 | 1.109276 | 8.90E-06 | 9.59E-05 | RHOA |
| ENSG00000159720 | 0.048411071 | 0.104222354 | 1.106255816 | 4.83E-06 | 5.57E-05 | ATP6V0D1 |
| ENSG00000168209 | 0.031705724 | 0.068183683 | 1.104683194 | 7.00E-06 | 7.73E-05 | DDIT4 |
| ENSG00000112110 | 0.024709924 | 0.053123992 | 1.104272955 | 0.00038678 | 0.002472639 | MRPL18 |
| ENSG00000125037 | 0.027980921 | 0.060152183 | 1.104173634 | 0.001689967 | 0.008393991 | EMC3 |
| ENSG00000013588 | 0.033776645 | 0.072569486 | 1.103337032 | 2.45E-06 | 3.12E-05 | GPRC5A |
| ENSG00000206503 | 0.01862701 | 0.040009612 | 1.102950539 | 0.000225586 | 0.001583578 | HLA-A |
| ENSG00000136271 | 0.028849367 | 0.061947722 | 1.102511599 | 3.09E-06 | 3.80E-05 | DDX56 |
| ENSG00000112576 | 0.010232601 | 0.021972032 | 1.102495435 | 0.000135882 | 0.001031551 | CCND3 |
| ENSG00000104518 | 0.017195159 | 0.036918923 | 1.102358067 | 0.000184753 | 0.001341577 | GSDMD |
| ENSG00000090339 | 0.02416314 | 0.051814847 | 1.100557633 | 0.0007081 | 0.004091734 | ICAM1 |
| ENSG00000144959 | 0.019422192 | 0.041633764 | 1.100047962 | 1.86E-11 | 6.31E-10 | NCEH1 |
| ENSG00000148175 | 0.018195771 | 0.038955814 | 1.098235499 | 6.52E-08 | 1.19E-06 | STOM |
| ENSG00000277443 | 0.017924598 | 0.038365425 | 1.097865952 | 0.001671003 | 0.008320619 | MARCKS |
| ENSG00000090487 | 0.011794061 | 0.025235915 | 1.097417849 | 0.001594917 | 0.007981221 | SPG21 |
| ENSG00000038274 | 0.019123914 | 0.040871292 | 1.095710019 | 0.000230031 | 0.001609147 | MAT2B |
| ENSG00000047315 | 0.029335551 | 0.062689842 | 1.095581584 | 1.11E-16 | 7.67E-15 | POLR2B |
| ENSG00000144589 | 0.020383292 | 0.043527904 | 1.094553475 | 0.000189863 | 0.001370406 | STK11IP |
| ENSG00000001036 | 0.020394371 | 0.043548935 | 1.094466393 | 0.000206802 | 0.001466268 | FUCA2 |
| ENSG00000102316 | 0.047138036 | 0.10065181 | 1.094409559 | 7.84E-06 | 8.54E-05 | MAGED2 |
| ENSG00000100994 | 0.0216745 | 0.04625616 | 1.093646785 | 5.22E-15 | 2.97E-13 | PYGB |
| ENSG00000152234 | 0.029984598 | 0.063916952 | 1.091976981 | 4.75E-06 | 5.49E-05 | ATP5F1A |
| ENSG00000027001 | 0.035737411 | 0.076146903 | 1.091350239 | 0.002049968 | 0.009809652 | MIPEP |
| ENSG00000188917 | 0.022268515 | 0.047421831 | 1.090546027 | 0.002088368 | 0.009961376 | TRMT2B |
| ENSG00000189241 | 0.03881837 | 0.082603437 | 1.089462283 | 0.000165001 | 0.001219504 | TSPYL1 |
| ENSG00000239306 | 0.015756827 | 0.033489275 | 1.087722138 | 1.24E-06 | 1.69E-05 | RBM14 |
| ENSG00000155508 | 0.021473657 | 0.045628816 | 1.087377327 | 0.000836155 | 0.004687536 | CNOT8 |
| ENSG00000117399 | 0.012495037 | 0.02648871 | 1.084022406 | 8.65E-08 | 1.52E-06 | CDC20 |
| ENSG00000059573 | 0.023077149 | 0.048875136 | 1.082635741 | 5.85E-07 | 8.59E-06 | ALDH18A1 |
| ENSG00000156508 | 0.003203405 | 0.006779909 | 1.081659483 | 3.06E-05 | 0.000286188 | EEF1A1 |
| ENSG00000177485 | 0.02115233 | 0.044727852 | 1.080356891 | 0.000745347 | 0.004265968 | ZBTB33 |
| ENSG00000135919 | 0.017669186 | 0.037356749 | 1.080133327 | 1.33E-07 | 2.26E-06 | SERPINE2 |
| ENSG00000166881 | 0.017505542 | 0.036978917 | 1.078891225 | 0.00024411 | 0.001696778 | NEMP1 |
| ENSG00000145817 | 0.027948882 | 0.059036102 | 1.078806867 | 2.64E-05 | 0.000251221 | YIPF5 |
| ENSG00000119899 | 0.020405678 | 0.043086465 | 1.078264101 | 0.00148651 | 0.007534532 | SLC17A5 |
| ENSG00000105568 | 0.029330239 | 0.061928126 | 1.078205966 | 8.88E-15 | 4.88E-13 | PPP2R1A |
| ENSG00000146242 | 0.017892789 | 0.037761806 | 1.077549455 | 4.43E-06 | 5.18E-05 | TPBG |
| ENSG00000124357 | 0.020647585 | 0.043550908 | 1.076729727 | 0.000611465 | 0.003636431 | NAGK |
| ENSG00000103018 | 0.013069362 | 0.027501677 | 1.073330904 | 0.000508344 | 0.003114027 | CYB5B |
| ENSG00000014824 | 0.025457425 | 0.053566114 | 1.07323413 | 2.71E-06 | 3.37E-05 | SLC30A9 |
| ENSG00000134308 | 0.050913969 | 0.107122948 | 1.073134109 | 2.23E-07 | 3.58E-06 | YWHAQ |
| ENSG00000139112 | 0.030117803 | 0.063225361 | 1.069886814 | 0.000100873 | 0.000801715 | GABARAPL1 |
| ENSG00000140395 | 0.039278049 | 0.082440107 | 1.069623106 | 0.001442968 | 0.00735865 | WDR61 |
| ENSG00000109736 | 0.014401643 | 0.030223116 | 1.069419011 | 1.37E-05 | 0.000139959 | MFSD10 |
| ENSG00000149485 | 0.010991343 | 0.023064439 | 1.069302469 | 8.68E-05 | 0.000703118 | FADS1 |
| ENSG00000169188 | 0.013478407 | 0.028260766 | 1.068150576 | 0.001272704 | 0.006633839 | APEX2 |
| ENSG00000143537 | 0.015112892 | 0.031677207 | 1.067665384 | 1.60E-10 | 4.69E-09 | ADAM15 |
| ENSG00000143369 | 0.03075655 | 0.064394942 | 1.066053694 | 0.001111361 | 0.005944889 | ECM1 |
| ENSG00000181523 | 0.010009986 | 0.020948645 | 1.065416956 | 0.000212948 | 0.001504522 | SGSH |
| ENSG00000164244 | 0.02186014 | 0.045676815 | 1.063159391 | 0.000858984 | 0.004787441 | PRRC1 |
| ENSG00000137203 | 0.078767151 | 0.164496801 | 1.06239353 | 4.59E-06 | 5.34E-05 | TFAP2A |
| ENSG00000164180 | 0.03123485 | 0.065212436 | 1.06199049 | 4.04E-05 | 0.000362262 | TMEM161B |
| ENSG00000160445 | 0.02324925 | 0.048530858 | 1.061718176 | 0.001081029 | 0.005805265 | ZER1 |
| ENSG00000145860 | 0.01645755 | 0.034324039 | 1.060469742 | 4.48E-13 | 2.00E-11 | RNF145 |
| ENSG00000101310 | 0.023379449 | 0.048719766 | 1.059266275 | 1.59E-09 | 3.91E-08 | SEC23B |
| ENSG00000144744 | 0.04440129 | 0.092394298 | 1.057202219 | 1.47E-05 | 0.000149458 | UBA3 |
| ENSG00000164941 | 0.023646769 | 0.049182999 | 1.056516631 | 0.000696428 | 0.004038489 | INTS8 |
| ENSG00000112655 | 0.012568899 | 0.026136181 | 1.056190132 | 0.0004307 | 0.002704524 | PTK7 |
| ENSG00000073060 | 0.026352039 | 0.054769186 | 1.055449815 | 9.18E-07 | 1.29E-05 | SCARB1 |
| ENSG00000138821 | 0.016163384 | 0.033548063 | 1.053500162 | 0.000640219 | 0.003784864 | SLC39A8 |
| ENSG00000094914 | 0.027283434 | 0.056519277 | 1.050717745 | 9.64E-07 | 1.34E-05 | AAAS |
| ENSG00000077147 | 0.022819514 | 0.047239974 | 1.049740123 | 7.11E-09 | 1.55E-07 | TM9SF3 |
| ENSG00000135390 | 0.01677371 | 0.034699803 | 1.048725683 | 0.001901829 | 0.009210427 | ATP5MC2 |
| ENSG00000115365 | 0.014250414 | 0.029451725 | 1.047348364 | 0.000565632 | 0.003411094 | LANCL1 |
| ENSG00000101084 | 0.041648498 | 0.085927166 | 1.044849859 | 0.00116758 | 0.006182363 | RAB5IF |
| ENSG00000137575 | 0.011096314 | 0.022888743 | 1.04455771 | 0.000452209 | 0.002821374 | SDCBP |
| ENSG00000120438 | 0.014196443 | 0.029270866 | 1.04393591 | 4.64E-05 | 0.000407723 | TCP1 |
| ENSG00000130669 | 0.014197844 | 0.029253795 | 1.042951908 | 2.84E-05 | 0.000268837 | PAK4 |
| ENSG00000062716 | 0.025215703 | 0.051939833 | 1.042518956 | 8.80E-06 | 9.50E-05 | VMP1 |
| ENSG00000185721 | 0.020957384 | 0.043165914 | 1.042433877 | 0.00089803 | 0.004958131 | DRG1 |
| ENSG00000163453 | 0.035057045 | 0.072165005 | 1.041595001 | 0.001469895 | 0.007473776 | IGFBP7 |
| ENSG00000154473 | 0.015138729 | 0.031159061 | 1.041407723 | 3.32E-06 | 4.04E-05 | BUB3 |
| ENSG00000006118 | 0.01310699 | 0.026961698 | 1.040575015 | 1.95E-05 | 0.000190583 | TMEM132A |
| ENSG00000106771 | 0.034004831 | 0.069913302 | 1.03982727 | 6.67E-08 | 1.21E-06 | TMEM245 |
| ENSG00000231925 | 0.015807566 | 0.032408793 | 1.035770017 | 8.02E-05 | 0.000658462 | TAPBP |
| ENSG00000092203 | 0.015041868 | 0.030798019 | 1.033853845 | 8.78E-05 | 0.000710198 | TOX4 |
| ENSG00000126088 | 0.033250106 | 0.068078785 | 1.033846356 | 0.000869375 | 0.00483321 | UROD |
| ENSG00000092931 | 0.034732574 | 0.071074701 | 1.033046782 | 0.000334819 | 0.002192918 | MFSD11 |
| ENSG00000126247 | 0.031988349 | 0.065351726 | 1.030678802 | 3.39E-05 | 0.000312261 | CAPNS1 |
| ENSG00000012963 | 0.030292056 | 0.06182748 | 1.029308693 | 3.21E-09 | 7.47E-08 | UBR7 |
| ENSG00000033011 | 0.035976094 | 0.073425285 | 1.029238378 | 0.000892803 | 0.004936072 | ALG1 |
| ENSG00000102144 | 0.018990285 | 0.038719156 | 1.027785916 | 2.04E-06 | 2.63E-05 | PGK1 |
| ENSG00000099246 | 0.026400295 | 0.053774547 | 1.02636943 | 0.000227942 | 0.001597296 | RAB18 |
| ENSG00000069998 | 0.019023953 | 0.038730791 | 1.025663926 | 0.00128356 | 0.006678844 | HDHD5 |
| ENSG00000197746 | 0.035433043 | 0.072118917 | 1.02528237 | 1.64E-11 | 5.58E-10 | PSAP |
| ENSG00000165678 | 0.02267178 | 0.046126396 | 1.024694939 | 0.000292749 | 0.001962267 | GHITM |
| ENSG00000127564 | 0.014346849 | 0.029171827 | 1.023841891 | 0.000546985 | 0.003321966 | PKMYT1 |
| ENSG00000146701 | 0.024521092 | 0.049831752 | 1.023042098 | 0.001847532 | 0.009005504 | MDH2 |
| ENSG00000121073 | 0.029427261 | 0.059794672 | 1.022863689 | 0.000349589 | 0.002268651 | SLC35B1 |
| ENSG00000168036 | 0.042697004 | 0.08667204 | 1.021431832 | 6.01E-13 | 2.55E-11 | CTNNB1 |
| ENSG00000148248 | 0.018927368 | 0.038396796 | 1.020512144 | 0 | 0 | SURF4 |
| ENSG00000126773 | 0.024920916 | 0.050546457 | 1.020252876 | 0.000408516 | 0.00259778 | PCNX4 |
| ENSG00000134255 | 0.048095388 | 0.097525078 | 1.0198747 | 0.001164665 | 0.006175087 | CEPT1 |
| ENSG00000114491 | 0.026633487 | 0.053926823 | 1.017761711 | 0.000167382 | 0.001234828 | UMPS |
| ENSG00000062485 | 0.01190889 | 0.024108875 | 1.017525436 | 1.16E-08 | 2.45E-07 | CS |
| ENSG00000116260 | 0.023174775 | 0.046829468 | 1.014861316 | 2.22E-16 | 1.49E-14 | QSOX1 |
| ENSG00000182220 | 0.015665958 | 0.031576338 | 1.011210875 | 0.000250573 | 0.001731689 | ATP6AP2 |
| ENSG00000163520 | 0.024278811 | 0.048922067 | 1.010787579 | 1.22E-05 | 0.000126921 | FBLN2 |
| ENSG00000014641 | 0.055353658 | 0.111526936 | 1.010641639 | 0.001644934 | 0.008214422 | MDH1 |
| ENSG00000174915 | 0.037320343 | 0.075075915 | 1.008387893 | 3.61E-05 | 0.000328998 | PTDSS2 |
| ENSG00000115419 | 0.026127961 | 0.052534293 | 1.007664927 | 0.000874384 | 0.004856568 | GLS |
| ENSG00000132434 | 0.01863132 | 0.037370476 | 1.004169025 | 0.00103534 | 0.005587953 | LANCL2 |
| ENSG00000183283 | 0.008206339 | 0.01645708 | 1.00389782 | 3.78E-06 | 4.52E-05 | DAZAP2 |
| ENSG00000049860 | 0.024708506 | 0.049505426 | 1.00257888 | 0.000432929 | 0.002715685 | HEXB |
| ENSG00000185515 | 0.034954769 | 0.069901253 | 0.999829014 | 1.14E-06 | 1.57E-05 | BRCC3 |
| ENSG00000110955 | 0.027595338 | 0.055169669 | 0.999450778 | 1.28E-06 | 1.74E-05 | ATP5F1B |
| ENSG00000165283 | 0.020739382 | 0.041458202 | 0.99928464 | 0.000785407 | 0.004465496 | STOML2 |
| ENSG00000184292 | 0.013297287 | 0.0265728 | 0.998818301 | 0.000888112 | 0.004918526 | TACSTD2 |
| ENSG00000100105 | 0.016824331 | 0.03359719 | 0.997791462 | 0.000335396 | 0.002195505 | PATZ1 |
| ENSG00000158710 | 0.017303966 | 0.034554364 | 0.997765167 | 2.93E-06 | 3.61E-05 | TAGLN2 |
| ENSG00000058262 | 0.017442452 | 0.034805527 | 0.996713554 | 6.55E-15 | 3.68E-13 | SEC61A1 |
| ENSG00000100883 | 0.025126884 | 0.050107069 | 0.995782386 | 0.000130196 | 0.000996399 | SRP54 |
| ENSG00000064651 | 0.053966373 | 0.107479346 | 0.99392682 | 7.88E-07 | 1.13E-05 | SLC12A2 |
| ENSG00000130638 | 0.054197965 | 0.10789114 | 0.993265805 | 0.000578317 | 0.003473655 | ATXN10 |
| ENSG00000090674 | 0.03466417 | 0.068983928 | 0.99281508 | 0.000781781 | 0.004446982 | MCOLN1 |
| ENSG00000116922 | 0.032484773 | 0.064636424 | 0.992583757 | 0.00018257 | 0.00132813 | C1orf109 |
| ENSG00000169567 | 0.017355765 | 0.034520575 | 0.992041509 | 0.001139206 | 0.006061481 | HINT1 |
| ENSG00000095002 | 0.027055092 | 0.053786725 | 0.991349984 | 9.03E-09 | 1.94E-07 | MSH2 |
| ENSG00000141574 | 0.016233448 | 0.032250159 | 0.990336822 | 6.22E-05 | 0.000528915 | SECTM1 |
| ENSG00000101365 | 0.02162202 | 0.042918521 | 0.989099051 | 0.000138675 | 0.001049444 | IDH3B |
| ENSG00000161057 | 0.032848308 | 0.06517139 | 0.988419702 | 0.000419556 | 0.002652553 | PSMC2 |
| ENSG00000106991 | 0.014071501 | 0.027916946 | 0.988364914 | 0.000983745 | 0.005352733 | ENG |
| ENSG00000225697 | 0.028666969 | 0.056778191 | 0.985947526 | 0.001878554 | 0.009123432 | SLC26A6 |
| ENSG00000221914 | 0.019419725 | 0.03842822 | 0.984643389 | 0.000112968 | 0.000883249 | PPP2R2A |
| ENSG00000124207 | 0.034203931 | 0.067658569 | 0.984110513 | 1.23E-07 | 2.10E-06 | CSE1L |
| ENSG00000067829 | 0.018846239 | 0.037256819 | 0.983227859 | 0.000511325 | 0.003129103 | IDH3G |
| ENSG00000173812 | 0.01290801 | 0.025509331 | 0.982758451 | 0.00048189 | 0.002973177 | EIF1 |
| ENSG00000079332 | 0.015902165 | 0.031424837 | 0.982682013 | 7.40E-05 | 0.000612569 | SAR1A |
| ENSG00000131269 | 0.054269504 | 0.10720795 | 0.982198271 | 0.000573218 | 0.003449922 | ABCB7 |
| ENSG00000151135 | 0.016379892 | 0.032307667 | 0.979950741 | 0.001830503 | 0.008951523 | TMEM263 |
| ENSG00000149308 | 0.022728743 | 0.044819233 | 0.979600068 | 0.001882144 | 0.009133486 | NPAT |
| ENSG00000244462 | 0.015452988 | 0.030456946 | 0.978885413 | 0.000210163 | 0.001487471 | RBM12 |
| ENSG00000163399 | 0.051161147 | 0.100773615 | 0.977997444 | 2.20E-11 | 7.38E-10 | ATP1A1 |
| ENSG00000128245 | 0.040626262 | 0.079785567 | 0.973715174 | 0.000524112 | 0.00319921 | YWHAH |
| ENSG00000177105 | 0.015768955 | 0.030957667 | 0.973209729 | 0.000668167 | 0.003913645 | RHOG |
| ENSG00000204463 | 0.024770559 | 0.04858404 | 0.971856075 | 2.45E-05 | 0.000234754 | BAG6 |
| ENSG00000138442 | 0.035004072 | 0.068635041 | 0.971422568 | 5.07E-05 | 0.000440001 | WDR12 |
| ENSG00000006534 | 0.011324516 | 0.022131795 | 0.9666711 | 0.00033818 | 0.002207881 | ALDH3B1 |
| ENSG00000125166 | 0.0182278 | 0.035575533 | 0.964744913 | 1.38E-09 | 3.42E-08 | GOT2 |
| ENSG00000138279 | 0.020174033 | 0.039370879 | 0.964629391 | 0.000119625 | 0.000927459 | ANXA7 |
| ENSG00000080189 | 0.018766611 | 0.036549793 | 0.961695096 | 0.000146 | 0.0010966 | SLC35C2 |
| ENSG00000204628 | 0.042718128 | 0.083193172 | 0.961616684 | 1.64E-06 | 2.17E-05 | RACK1 |
| ENSG00000107798 | 0.027550581 | 0.053608112 | 0.960368569 | 2.61E-07 | 4.12E-06 | LIPA |
| ENSG00000063660 | 0.019900502 | 0.038720727 | 0.960301177 | 4.85E-05 | 0.000424272 | GPC1 |
| ENSG00000110911 | 0.030725319 | 0.059594548 | 0.955752367 | 8.56E-07 | 1.21E-05 | SLC11A2 |
| ENSG00000134759 | 0.040526749 | 0.078582798 | 0.955339104 | 4.54E-06 | 5.29E-05 | ELP2 |
| ENSG00000111530 | 0.031004209 | 0.060066507 | 0.954096671 | 1.11E-16 | 7.67E-15 | CAND1 |
| ENSG00000163257 | 0.014519675 | 0.028127065 | 0.953949826 | 0.000348136 | 0.002261664 | DCAF16 |
| ENSG00000198689 | 0.025601274 | 0.04958466 | 0.953678243 | 0.001013113 | 0.005487113 | SLC9A6 |
| ENSG00000155876 | 0.024929787 | 0.048219471 | 0.951745258 | 0.000547385 | 0.003322715 | RRAGA |
| ENSG00000138095 | 0.038848073 | 0.075108091 | 0.951125271 | 4.64E-13 | 2.04E-11 | LRPPRC |
| ENSG00000180340 | 0.040430516 | 0.078128196 | 0.950398677 | 0.001130806 | 0.006027458 | FZD2 |
| ENSG00000105011 | 0.023373906 | 0.045094751 | 0.948060691 | 9.97E-10 | 2.53E-08 | ASF1B |
| ENSG00000242372 | 0.040328857 | 0.077529599 | 0.942934695 | 0.000178428 | 0.001301943 | EIF6 |
| ENSG00000163507 | 0.032195944 | 0.061894006 | 0.942920754 | 2.28E-07 | 3.65E-06 | CIP2A |
| ENSG00000171298 | 0.019580805 | 0.037589667 | 0.940896049 | 0.001740115 | 0.008586329 | GAA |
| ENSG00000274523 | 0.014992886 | 0.028754389 | 0.939504102 | 0.00080936 | 0.0045693 | RCC1L |
| ENSG00000106105 | 0.034046944 | 0.065247288 | 0.938392645 | 4.59E-08 | 8.67E-07 | GARS |
| ENSG00000151348 | 0.021506604 | 0.04116003 | 0.936464309 | 2.72E-08 | 5.40E-07 | EXT2 |
| ENSG00000171566 | 0.033620371 | 0.064340252 | 0.936385949 | 6.62E-05 | 0.000557719 | PLRG1 |
| ENSG00000130821 | 0.008687557 | 0.016617795 | 0.93570657 | 7.14E-05 | 0.000592991 | SLC6A8 |
| ENSG00000100991 | 0.023699609 | 0.04532853 | 0.935556091 | 1.43E-09 | 3.54E-08 | TRPC4AP |
| ENSG00000101290 | 0.03427428 | 0.065541645 | 0.935285516 | 0.000594063 | 0.003555801 | CDS2 |
| ENSG00000161011 | 0.023475181 | 0.044866599 | 0.934505527 | 0 | 0 | SQSTM1 |
| ENSG00000122958 | 0.015970351 | 0.030491623 | 0.933016967 | 0.001937195 | 0.009355328 | VPS26A |
| ENSG00000022267 | 0.013388928 | 0.025559645 | 0.932827349 | 0.001248295 | 0.006532092 | FHL1 |
| ENSG00000132436 | 0.021690896 | 0.041370972 | 0.931529197 | 0.000500607 | 0.003076036 | FIGNL1 |
| ENSG00000213585 | 0.013283294 | 0.025313019 | 0.93026668 | 0.000104667 | 0.000825869 | VDAC1 |
| ENSG00000249915 | 0.028260661 | 0.053845964 | 0.930042985 | 2.49E-05 | 0.000238703 | PDCD6 |
| ENSG00000149923 | 0.026483326 | 0.050399953 | 0.92833808 | 0.001800135 | 0.008842576 | PPP4C |
| ENSG00000106348 | 0.010199364 | 0.019388841 | 0.92674732 | 0.001311274 | 0.006799514 | IMPDH1 |
| ENSG00000146828 | 0.040374569 | 0.076653119 | 0.924897642 | 4.89E-06 | 5.63E-05 | SLC12A9 |
| ENSG00000131043 | 0.020963544 | 0.039748712 | 0.92302552 | 9.84E-05 | 0.000785059 | AAR2 |
| ENSG00000089693 | 0.014985915 | 0.028407979 | 0.922689007 | 3.57E-05 | 0.000325988 | MLF2 |
| ENSG00000131475 | 0.057508327 | 0.108878376 | 0.920874681 | 0.000203569 | 0.001448469 | VPS25 |
| ENSG00000104419 | 0.030089413 | 0.056940757 | 0.920205701 | 0.00016838 | 0.001239908 | NDRG1 |
| ENSG00000078140 | 0.018344373 | 0.034687082 | 0.919060883 | 1.86E-05 | 0.00018386 | UBE2K |
| ENSG00000161091 | 0.016819492 | 0.031794384 | 0.918637815 | 0.001255444 | 0.00655524 | MFSD12 |
| ENSG00000157500 | 0.020943337 | 0.039578228 | 0.918215683 | 0.000444713 | 0.002779454 | APPL1 |
| ENSG00000003436 | 0.037685815 | 0.071165652 | 0.917159518 | 8.80E-06 | 9.50E-05 | TFPI |
| ENSG00000110660 | 0.027110958 | 0.05116987 | 0.916418449 | 0.000738904 | 0.004237157 | SLC35F2 |
| ENSG00000167986 | 0.024984929 | 0.047149283 | 0.916177711 | 1.01E-07 | 1.75E-06 | DDB1 |
| ENSG00000113048 | 0.01668286 | 0.031443574 | 0.914398576 | 0.0008506 | 0.00474859 | MRPS27 |
| ENSG00000160746 | 0.026020472 | 0.049007949 | 0.913368643 | 0.000622753 | 0.003699902 | ANO10 |
| ENSG00000130202 | 0.016476001 | 0.031016779 | 0.912682765 | 3.89E-05 | 0.000350462 | NECTIN2 |
| ENSG00000173692 | 0.032515024 | 0.060956658 | 0.906677299 | 7.89E-08 | 1.40E-06 | PSMD1 |
| ENSG00000117362 | 0.018381619 | 0.034367641 | 0.902787039 | 0.000564491 | 0.003407631 | APH1A |
| ENSG00000112282 | 0.033684705 | 0.062973575 | 0.902652906 | 0.000676423 | 0.003950452 | MED23 |
| ENSG00000101294 | 0.043063856 | 0.08050083 | 0.902526152 | 8.31E-11 | 2.54E-09 | HM13 |
| ENSG00000122729 | 0.019209613 | 0.035872975 | 0.901068925 | 6.87E-08 | 1.24E-06 | ACO1 |
| ENSG00000160789 | 0.03758437 | 0.070139509 | 0.900094504 | 3.63E-10 | 1.00E-08 | LMNA |
| ENSG00000123983 | 0.024681885 | 0.046038288 | 0.899381586 | 0.001296305 | 0.006735979 | ACSL3 |
| ENSG00000120053 | 0.027033729 | 0.050379218 | 0.898068169 | 0.000265718 | 0.00181859 | GOT1 |
| ENSG00000152952 | 0.028501316 | 0.053074894 | 0.897001067 | 2.64E-08 | 5.24E-07 | PLOD2 |
| ENSG00000145685 | 0.015667929 | 0.02914661 | 0.895513578 | 0.000326877 | 0.002149093 | LHFPL2 |
| ENSG00000107651 | 0.023430551 | 0.043528522 | 0.893570141 | 4.10E-06 | 4.84E-05 | SEC23IP |
| ENSG00000120253 | 0.034393382 | 0.063872535 | 0.893064724 | 0.000574784 | 0.003455887 | NUP43 |
| ENSG00000166313 | 0.02231732 | 0.041409492 | 0.891797754 | 0.00097354 | 0.005307981 | APBB1 |
| ENSG00000124145 | 0.020502382 | 0.038041223 | 0.891772068 | 3.96E-09 | 9.06E-08 | SDC4 |
| ENSG00000105976 | 0.029953974 | 0.055545459 | 0.89092156 | 0.000178882 | 0.001304464 | MET |
| ENSG00000113569 | 0.036744575 | 0.068049531 | 0.889053967 | 0.000352096 | 0.002283685 | NUP155 |
| ENSG00000179115 | 0.029529925 | 0.054686145 | 0.888997646 | 1.11E-05 | 0.000117484 | FARSA |
| ENSG00000106608 | 0.016567907 | 0.030674748 | 0.888660127 | 0.001761848 | 0.008679319 | URGCP |
| ENSG00000163346 | 0.030030429 | 0.055590075 | 0.888402271 | 6.46E-05 | 0.000547261 | PBXIP1 |
| ENSG00000116120 | 0.02580538 | 0.047696048 | 0.886197848 | 0.001112202 | 0.005946743 | FARSB |
| ENSG00000106628 | 0.020852416 | 0.038535295 | 0.885965902 | 0.00012849 | 0.000986016 | POLD2 |
| ENSG00000120885 | 0.056975853 | 0.105291456 | 0.885965848 | 0.001129929 | 0.006027308 | CLU |
| ENSG00000156471 | 0.037089445 | 0.068531382 | 0.885756108 | 6.87E-08 | 1.24E-06 | PTDSS1 |
| ENSG00000134001 | 0.031987649 | 0.059022065 | 0.883739456 | 0.00118118 | 0.006237898 | EIF2S1 |
| ENSG00000115415 | 0.023581931 | 0.043505237 | 0.883507212 | 1.39E-07 | 2.35E-06 | STAT1 |
| ENSG00000143149 | 0.037932952 | 0.069821097 | 0.880211357 | 2.72E-05 | 0.000258386 | ALDH9A1 |
| ENSG00000143374 | 0.031855389 | 0.05860178 | 0.879407005 | 0.000737389 | 0.004234526 | TARS2 |
| ENSG00000113194 | 0.015547568 | 0.028553627 | 0.876985086 | 0.000149685 | 0.001121469 | FAF2 |
| ENSG00000142230 | 0.01799166 | 0.033025902 | 0.876269648 | 3.86E-05 | 0.00034778 | SAE1 |
| ENSG00000099797 | 0.024289523 | 0.044583769 | 0.876184414 | 0.001611859 | 0.008055946 | TECR |
| ENSG00000142945 | 0.035392847 | 0.064942265 | 0.875699867 | 9.56E-06 | 0.000102227 | KIF2C |
| ENSG00000130714 | 0.038075243 | 0.069802233 | 0.874419948 | 4.37E-05 | 0.000388144 | POMT1 |
| ENSG00000168476 | 0.030326418 | 0.055534672 | 0.87281366 | 0.000452716 | 0.002821374 | REEP4 |
| ENSG00000104763 | 0.027636571 | 0.050422649 | 0.867493278 | 0.000522493 | 0.003190949 | ASAH1 |
| ENSG00000160285 | 0.013224608 | 0.024114372 | 0.86666824 | 9.54E-05 | 0.000764561 | LSS |
| ENSG00000168175 | 0.015999815 | 0.029169635 | 0.866412136 | 0.000746969 | 0.004269766 | MAPK1IP1L |
| ENSG00000110651 | 0.033359719 | 0.060749962 | 0.864776354 | 0.001156417 | 0.006136765 | CD81 |
| ENSG00000108679 | 0.025561565 | 0.046515168 | 0.863725052 | 2.65E-10 | 7.51E-09 | LGALS3BP |
| ENSG00000158864 | 0.039364071 | 0.071525388 | 0.861575986 | 0.001832991 | 0.008960047 | NDUFS2 |
| ENSG00000125257 | 0.042140917 | 0.076559096 | 0.861352073 | 0.000248004 | 0.001716078 | ABCC4 |
| ENSG00000187957 | 0.02373931 | 0.043117927 | 0.86100982 | 0.000824272 | 0.004631715 | DNER |
| ENSG00000205937 | 0.006781945 | 0.012307668 | 0.859786472 | 0.000170332 | 0.001251979 | RNPS1 |
| ENSG00000165688 | 0.032428968 | 0.058848279 | 0.859717081 | 0.000920694 | 0.005069298 | PMPCA |
| ENSG00000227500 | 0.018697845 | 0.033911122 | 0.858886511 | 0.001681205 | 0.008364291 | SCAMP4 |
| ENSG00000080815 | 0.033777859 | 0.061178061 | 0.856936497 | 1.19E-05 | 0.000124801 | PSEN1 |
| ENSG00000150687 | 0.013094916 | 0.02370166 | 0.855981303 | 3.42E-05 | 0.000313487 | PRSS23 |
| ENSG00000141076 | 0.035689923 | 0.064589218 | 0.855776556 | 5.46E-05 | 0.000468992 | UTP4 |
| ENSG00000143493 | 0.027743033 | 0.050149811 | 0.854118718 | 0.000391421 | 0.002498319 | INTS7 |
| ENSG00000164308 | 0.02852795 | 0.051431817 | 0.850285048 | 1.40E-10 | 4.17E-09 | ERAP2 |
| ENSG00000197324 | 0.012867764 | 0.023167122 | 0.848317475 | 0.001879805 | 0.009125821 | LRP10 |
| ENSG00000244038 | 0.022424222 | 0.040364389 | 0.8480251 | 2.82E-12 | 1.08E-10 | DDOST |
| ENSG00000144580 | 0.016838798 | 0.030226985 | 0.844047955 | 1.60E-05 | 0.000160371 | CNOT9 |
| ENSG00000068366 | 0.018596195 | 0.033275426 | 0.839449698 | 6.17E-06 | 6.92E-05 | ACSL4 |
| ENSG00000221968 | 0.0178406 | 0.031900058 | 0.838394939 | 0.000307801 | 0.002047183 | FADS3 |
| ENSG00000170043 | 0.040348766 | 0.072094516 | 0.837364981 | 0.001396616 | 0.007173987 | TRAPPC1 |
| ENSG00000013364 | 0.020250433 | 0.036166221 | 0.836690082 | 0.001082876 | 0.005810611 | MVP |
| ENSG00000166266 | 0.023334236 | 0.041627359 | 0.835083776 | 0.001539525 | 0.007760087 | CUL5 |
| ENSG00000066427 | 0.049224164 | 0.087625702 | 0.831987392 | 0.001565855 | 0.007865247 | ATXN3 |
| ENSG00000110107 | 0.032782727 | 0.058325543 | 0.83119197 | 5.10E-09 | 1.15E-07 | PRPF19 |
| ENSG00000086200 | 0.045892573 | 0.081628065 | 0.830804562 | 0.000703126 | 0.004067358 | IPO11 |
| ENSG00000154096 | 0.020232061 | 0.035980081 | 0.830555138 | 1.03E-07 | 1.77E-06 | THY1 |
| ENSG00000198824 | 0.026453161 | 0.046991301 | 0.828953601 | 0.00067196 | 0.003928936 | CHAMP1 |
| ENSG00000074695 | 0.023184064 | 0.041140092 | 0.827411522 | 0.000326608 | 0.002148504 | LMAN1 |
| ENSG00000127463 | 0.036001558 | 0.063658786 | 0.822300298 | 1.65E-06 | 2.18E-05 | EMC1 |
| ENSG00000140740 | 0.020649084 | 0.036424031 | 0.818812805 | 0.000230998 | 0.001613698 | UQCRC2 |
| ENSG00000116062 | 0.029654477 | 0.052136511 | 0.814044097 | 3.35E-05 | 0.000308765 | MSH6 |
| ENSG00000077942 | 0.017425731 | 0.030620833 | 0.813294353 | 0.000277416 | 0.001879395 | FBLN1 |
| ENSG00000149115 | 0.019932493 | 0.034933527 | 0.809490171 | 0.001536179 | 0.007751803 | TNKS1BP1 |
| ENSG00000177697 | 0.030394565 | 0.053121996 | 0.805495994 | 0.000819806 | 0.004613087 | CD151 |
| ENSG00000170266 | 0.037639477 | 0.065773829 | 0.805267071 | 5.81E-07 | 8.56E-06 | GLB1 |
| ENSG00000170876 | 0.023294757 | 0.04065941 | 0.803584002 | 1.99E-06 | 2.58E-05 | TMEM43 |
| ENSG00000148110 | 0.014520073 | 0.025313298 | 0.801846735 | 6.78E-05 | 0.000566535 | MFSD14B |
| ENSG00000118705 | 0.051131938 | 0.089092034 | 0.80107172 | 7.05E-07 | 1.02E-05 | RPN2 |
| ENSG00000091317 | 0.014570333 | 0.025359679 | 0.799502603 | 0.001023416 | 0.005531048 | CMTM6 |
| ENSG00000184007 | 0.037129794 | 0.064606457 | 0.799101044 | 3.65E-05 | 0.000331716 | PTP4A2 |
| ENSG00000101447 | 0.02081734 | 0.036143334 | 0.795943834 | 1.16E-05 | 0.0001217 | FAM83D |
| ENSG00000123130 | 0.048193948 | 0.083627847 | 0.795131449 | 0.001420293 | 0.007270762 | ACOT9 |
| ENSG00000136891 | 0.029693865 | 0.051484547 | 0.793974604 | 0.000247689 | 0.001716078 | TEX10 |
| ENSG00000105220 | 0.04646996 | 0.08043416 | 0.791509949 | 2.36E-09 | 5.60E-08 | GPI |
| ENSG00000170315 | 0.026046904 | 0.045082215 | 0.791446511 | 0.001094122 | 0.005863109 | UBB |
| ENSG00000129474 | 0.017031748 | 0.029455304 | 0.790300954 | 7.42E-07 | 1.07E-05 | AJUBA |
| ENSG00000149591 | 0.068897658 | 0.119110807 | 0.789777472 | 6.21E-05 | 0.000528331 | TAGLN |
| ENSG00000064313 | 0.023707306 | 0.040956236 | 0.788751388 | 0.002069934 | 0.009885208 | TAF2 |
| ENSG00000204262 | 0.048089715 | 0.083053989 | 0.7883211 | 6.65E-05 | 0.000558281 | COL5A2 |
| ENSG00000136492 | 0.027125306 | 0.046806467 | 0.78706847 | 0.001496631 | 0.007571303 | BRIP1 |
| ENSG00000093183 | 0.01443529 | 0.024883604 | 0.785595414 | 0.001130277 | 0.006027308 | SEC22C |
| ENSG00000120539 | 0.022789636 | 0.039241289 | 0.783994578 | 0.000979814 | 0.005334209 | MASTL |
| ENSG00000266412 | 0.015650608 | 0.026900394 | 0.781408652 | 0.000304429 | 0.002027 | NCOA4 |
| ENSG00000135446 | 0.024070039 | 0.041279803 | 0.778197604 | 0.000665689 | 0.003904831 | CDK4 |
| ENSG00000128335 | 0.025831714 | 0.044247936 | 0.776466811 | 0.000979152 | 0.005334209 | APOL2 |
| ENSG00000102158 | 0.037787733 | 0.064709617 | 0.776062154 | 0.000186921 | 0.001353236 | MAGT1 |
| ENSG00000178952 | 0.024134857 | 0.041296842 | 0.774913164 | 0.000568358 | 0.003425816 | TUFM |
| ENSG00000124155 | 0.016103667 | 0.027510465 | 0.772591233 | 3.98E-06 | 4.72E-05 | PIGT |
| ENSG00000125484 | 0.023489348 | 0.040050466 | 0.769812344 | 0.001451103 | 0.007394626 | GTF3C4 |
| ENSG00000197771 | 0.029151026 | 0.04962917 | 0.767641669 | 1.25E-05 | 0.000129592 | MCMBP |
| ENSG00000146918 | 0.023013982 | 0.039120607 | 0.765418132 | 3.67E-07 | 5.64E-06 | NCAPG2 |
| ENSG00000129103 | 0.016747282 | 0.028446982 | 0.764348632 | 0.001597867 | 0.007992659 | SUMF2 |
| ENSG00000136738 | 0.03549272 | 0.060229552 | 0.762948419 | 0.00033571 | 0.00219636 | STAM |
| ENSG00000163975 | 0.013709122 | 0.023245913 | 0.761840897 | 0.000202552 | 0.001442085 | MELTF |
| ENSG00000184254 | 0.057985065 | 0.098133612 | 0.759066011 | 0.001651184 | 0.008242213 | ALDH1A3 |
| ENSG00000068438 | 0.025469404 | 0.043054662 | 0.757404277 | 0.001817338 | 0.008912514 | FTSJ1 |
| ENSG00000141543 | 0.046833946 | 0.079041794 | 0.755061075 | 1.57E-05 | 0.000158438 | EIF4A3 |
| ENSG00000137845 | 0.029079052 | 0.049012285 | 0.75316319 | 7.33E-09 | 1.59E-07 | ADAM10 |
| ENSG00000170144 | 0.007569459 | 0.012721992 | 0.749062425 | 0.000461528 | 0.002869635 | HNRNPA3 |
| ENSG00000083444 | 0.025753268 | 0.043273874 | 0.748740758 | 0.000285043 | 0.001921324 | PLOD1 |
| ENSG00000123416 | 0.007245036 | 0.012161894 | 0.7473032 | 0.001436701 | 0.007329797 | TUBA1B |
| ENSG00000196230 | 0.011594178 | 0.01946253 | 0.747298707 | 2.39E-06 | 3.06E-05 | TUBB |
| ENSG00000204525 | 0.018264302 | 0.030590404 | 0.744052534 | 0.000120793 | 0.000935308 | HLA-C |
| ENSG00000128510 | 0.033762254 | 0.056521781 | 0.743395696 | 5.86E-05 | 0.000501148 | CPA4 |
| ENSG00000155561 | 0.034838145 | 0.058280618 | 0.742348353 | 0.000383362 | 0.002456011 | NUP205 |
| ENSG00000124486 | 0.033753177 | 0.056438996 | 0.741669027 | 0.001120867 | 0.005982437 | USP9X |
| ENSG00000197170 | 0.045564153 | 0.075950181 | 0.737154153 | 0.001493248 | 0.007560551 | PSMD12 |
| ENSG00000180957 | 0.051060717 | 0.085089828 | 0.736772881 | 0.000295027 | 0.001973137 | PITPNB |
| ENSG00000144136 | 0.026582198 | 0.044288343 | 0.736466599 | 3.01E-05 | 0.000282713 | SLC20A1 |
| ENSG00000104805 | 0.034431326 | 0.057339708 | 0.735812822 | 2.27E-07 | 3.65E-06 | NUCB1 |
| ENSG00000102226 | 0.052337204 | 0.086893645 | 0.731413807 | 5.28E-06 | 6.02E-05 | USP11 |
| ENSG00000101346 | 0.017816445 | 0.029554406 | 0.730163742 | 0.001688704 | 0.008391185 | POFUT1 |
| ENSG00000174840 | 0.027496032 | 0.045561772 | 0.728600415 | 0.001885814 | 0.009147604 | PDE12 |
| ENSG00000111321 | 0.01252617 | 0.020729986 | 0.726773765 | 0.000219307 | 0.001544011 | LTBR |
| ENSG00000133835 | 0.035338264 | 0.058292479 | 0.722078592 | 0.000391985 | 0.002500595 | HSD17B4 |
| ENSG00000164535 | 0.041539373 | 0.068496268 | 0.721545935 | 0.000129799 | 0.000994798 | DAGLB |
| ENSG00000104142 | 0.026178921 | 0.043142979 | 0.720720159 | 0.002007332 | 0.009632148 | VPS18 |
| ENSG00000135018 | 0.020814424 | 0.034204114 | 0.716586223 | 5.74E-07 | 8.46E-06 | UBQLN1 |
| ENSG00000092964 | 0.057766283 | 0.094918716 | 0.71646492 | 0.000570326 | 0.003434236 | DPYSL2 |
| ENSG00000143761 | 0.025979656 | 0.042659553 | 0.715486531 | 8.58E-05 | 0.000696728 | ARF1 |
| ENSG00000079246 | 0.037197852 | 0.06099515 | 0.713475215 | 2.25E-06 | 2.89E-05 | XRCC5 |
| ENSG00000170522 | 0.029872021 | 0.048945643 | 0.712385573 | 0.000780148 | 0.004439791 | ELOVL6 |
| ENSG00000166913 | 0.034062708 | 0.05574638 | 0.710684974 | 0.000317533 | 0.002101212 | YWHAB |
| ENSG00000130741 | 0.018701241 | 0.030544478 | 0.707777595 | 0.000174498 | 0.001276364 | EIF2S3 |
| ENSG00000047188 | 0.027654639 | 0.045109984 | 0.705925231 | 0.000200957 | 0.001433281 | YTHDC2 |
| ENSG00000129353 | 0.035191883 | 0.057309044 | 0.703520099 | 0.000497074 | 0.003059017 | SLC44A2 |
| ENSG00000116237 | 0.053425689 | 0.086868535 | 0.701300103 | 2.28E-08 | 4.56E-07 | ICMT |
| ENSG00000196776 | 0.012778878 | 0.020663909 | 0.693352049 | 6.92E-05 | 0.00057566 | CD47 |
| ENSG00000067182 | 0.016953903 | 0.027410641 | 0.693118611 | 1.28E-05 | 0.000131856 | TNFRSF1A |
| ENSG00000161800 | 0.043516792 | 0.070266666 | 0.691268259 | 4.52E-07 | 6.83E-06 | RACGAP1 |
| ENSG00000175221 | 0.019398973 | 0.031309207 | 0.690606685 | 0.001571606 | 0.007890838 | MED16 |
| ENSG00000072274 | 0.028686707 | 0.046273442 | 0.689802074 | 2.66E-05 | 0.000253255 | TFRC |
| ENSG00000106397 | 0.034802763 | 0.056055708 | 0.687659447 | 6.36E-07 | 9.26E-06 | PLOD3 |
| ENSG00000159202 | 0.016115528 | 0.025913681 | 0.685262462 | 1.22E-06 | 1.66E-05 | UBE2Z |
| ENSG00000110906 | 0.020915085 | 0.033571488 | 0.6826926 | 0.000253521 | 0.00174905 | KCTD10 |
| ENSG00000170348 | 0.016536076 | 0.026415688 | 0.675778054 | 4.40E-06 | 5.16E-05 | TMED10 |
| ENSG00000171302 | 0.013877005 | 0.022147686 | 0.674459792 | 0.000325526 | 0.00214373 | CANT1 |
| ENSG00000119314 | 0.019989444 | 0.031853831 | 0.672228581 | 0.000175753 | 0.001283981 | PTBP3 |
| ENSG00000173744 | 0.016020113 | 0.025525855 | 0.672074972 | 0.001092327 | 0.005856099 | AGFG1 |
| ENSG00000168397 | 0.04985188 | 0.079326288 | 0.67015111 | 0.001412768 | 0.007237575 | ATG4B |
| ENSG00000155850 | 0.02381153 | 0.037855849 | 0.668855877 | 0.001539756 | 0.007760087 | SLC26A2 |
| ENSG00000094880 | 0.029405715 | 0.046744506 | 0.668700263 | 0.000260177 | 0.001785747 | CDC23 |
| ENSG00000005893 | 0.018521471 | 0.029416545 | 0.667429142 | 5.00E-05 | 0.000435549 | LAMP2 |
| ENSG00000100934 | 0.021211415 | 0.033673575 | 0.666776032 | 2.95E-05 | 0.000278777 | SEC23A |
| ENSG00000152558 | 0.010289086 | 0.016312972 | 0.664904842 | 0.001482808 | 0.007523531 | TMEM123 |
| ENSG00000102900 | 0.028274644 | 0.044811607 | 0.66436359 | 0.000331809 | 0.002176762 | NUP93 |
| ENSG00000089280 | 0.028294172 | 0.044692909 | 0.659541052 | 1.99E-07 | 3.23E-06 | FUS |
| ENSG00000168159 | 0.016463174 | 0.025816128 | 0.649030152 | 0.001163613 | 0.006172228 | RNF187 |
| ENSG00000103257 | 0.017565098 | 0.027486111 | 0.645991196 | 0.001917968 | 0.009277381 | SLC7A5 |
| ENSG00000115310 | 0.032381037 | 0.050636496 | 0.645028387 | 5.30E-11 | 1.66E-09 | RTN4 |
| ENSG00000070961 | 0.028660532 | 0.044797497 | 0.64435277 | 1.25E-05 | 0.000129592 | ATP2B1 |
| ENSG00000197548 | 0.051632974 | 0.080517483 | 0.641009378 | 0.001495631 | 0.00756943 | ATG7 |
| ENSG00000127948 | 0.053444567 | 0.082821595 | 0.63196368 | 0.000790376 | 0.004489502 | POR |
| ENSG00000179091 | 0.017405885 | 0.026930552 | 0.629668637 | 0.000125786 | 0.000968982 | CYC1 |
| ENSG00000014216 | 0.023948834 | 0.036973412 | 0.626532804 | 5.07E-05 | 0.000440001 | CAPN1 |
| ENSG00000123146 | 0.075425394 | 0.116391667 | 0.625865547 | 0.000435777 | 0.002730704 | ADGRE5 |
| ENSG00000090006 | 0.019860101 | 0.030552543 | 0.62141953 | 0.000929376 | 0.005107744 | LTBP4 |
| ENSG00000108861 | 0.012188012 | 0.018677177 | 0.615813621 | 0.001878295 | 0.009123432 | DUSP3 |
| ENSG00000217128 | 0.036853016 | 0.056471488 | 0.615739951 | 0.000290803 | 0.001952486 | FNIP1 |
| ENSG00000138107 | 0.026353388 | 0.040366755 | 0.61517916 | 8.73E-06 | 9.44E-05 | ACTR1A |
| ENSG00000165915 | 0.024482757 | 0.037484929 | 0.614544659 | 0.001281841 | 0.006672786 | SLC39A13 |
| ENSG00000082898 | 0.026916001 | 0.041179185 | 0.613451216 | 3.69E-08 | 7.12E-07 | XPO1 |
| ENSG00000163466 | 0.019427598 | 0.029688565 | 0.611799824 | 0.001384431 | 0.00711804 | ARPC2 |
| ENSG00000164904 | 0.025680753 | 0.039164724 | 0.608867308 | 0.000798788 | 0.004520202 | ALDH7A1 |
| ENSG00000100243 | 0.023670464 | 0.036075564 | 0.607934004 | 1.19E-05 | 0.000124285 | CYB5R3 |
| ENSG00000075618 | 0.01869911 | 0.028375503 | 0.60167637 | 0.001988305 | 0.009552285 | FSCN1 |
| ENSG00000135677 | 0.018512593 | 0.028071914 | 0.600620428 | 0.000999925 | 0.005428487 | GNS |
| ENSG00000176390 | 0.60459848 | 0.914050201 | 0.59629605 | 0.000428045 | 0.002689259 | CRLF3 |
| ENSG00000171314 | 0.010980011 | 0.016576045 | 0.594220295 | 0.000578721 | 0.003474351 | PGAM1 |
| ENSG00000129245 | 0.026174017 | 0.039359326 | 0.588570195 | 0.000889654 | 0.004923188 | FXR2 |
| ENSG00000164062 | 0.029025422 | 0.043446353 | 0.581918007 | 0.000385742 | 0.002467455 | APEH |
| ENSG00000165119 | 0.021192056 | 0.031677723 | 0.579945083 | 0.00042308 | 0.002672025 | HNRNPK |
| ENSG00000138760 | 0.022024937 | 0.03283028 | 0.575889184 | 0.001110439 | 0.005944889 | SCARB2 |
| ENSG00000172354 | 0.02145608 | 0.031905797 | 0.572432086 | 0.00198236 | 0.009527528 | GNB2 |
| ENSG00000007168 | 0.030333673 | 0.044832586 | 0.563627494 | 0.00019363 | 0.001394254 | PAFAH1B1 |
| ENSG00000109606 | 0.030314609 | 0.044642456 | 0.558403156 | 0.000126142 | 0.000969857 | DHX15 |
| ENSG00000108582 | 0.035423553 | 0.052016694 | 0.55426577 | 0.000635954 | 0.003765311 | CPD |
| ENSG00000169679 | 0.029767258 | 0.043646193 | 0.552129477 | 0.000337618 | 0.002206442 | BUB1 |
| ENSG00000108828 | 0.016438563 | 0.023989488 | 0.545318196 | 0.001729922 | 0.008553583 | VAT1 |
| ENSG00000158623 | 0.048402573 | 0.070617556 | 0.544943158 | 0.001723684 | 0.008536779 | COPG2 |
| ENSG00000062725 | 0.039071788 | 0.056889594 | 0.542037507 | 0.000891271 | 0.004929868 | APPBP2 |
| ENSG00000136628 | 0.054599594 | 0.079378666 | 0.539861097 | 0.001804238 | 0.00885662 | EPRS |
| ENSG00000132912 | 0.025148834 | 0.036314615 | 0.530058751 | 0.000520228 | 0.003180346 | DCTN4 |
| ENSG00000113140 | 0.027369104 | 0.039487943 | 0.528864006 | 5.23E-12 | 1.87E-10 | SPARC |
| ENSG00000175166 | 0.034059055 | 0.049121166 | 0.528306403 | 0.00030276 | 0.002018119 | PSMD2 |
| ENSG00000108829 | 0.033909016 | 0.048898402 | 0.52811839 | 9.58E-05 | 0.000767354 | LRRC59 |
| ENSG00000160211 | 0.034555817 | 0.049789939 | 0.526925684 | 4.85E-07 | 7.26E-06 | G6PD |
| ENSG00000069329 | 0.024643257 | 0.035415477 | 0.523187002 | 0.000636648 | 0.003767567 | VPS35 |
| ENSG00000065150 | 0.047748018 | 0.068179633 | 0.513899986 | 5.46E-06 | 6.22E-05 | IPO5 |
| ENSG00000150093 | 0.020455401 | 0.0291117 | 0.509117287 | 0.000247459 | 0.001716078 | ITGB1 |
| ENSG00000197043 | 0.038736197 | 0.054914027 | 0.503492409 | 0.000423587 | 0.002673821 | ANXA6 |
| ENSG00000114978 | 0.020878923 | 0.029589016 | 0.503014428 | 0.001401435 | 0.007192597 | MOB1A |
| ENSG00000113580 | 0.035439145 | 0.049870234 | 0.492835157 | 0.001208094 | 0.006349356 | NR3C1 |
| ENSG00000143384 | 0.019154235 | 0.026846549 | 0.487073242 | 0.000137741 | 0.001043036 | MCL1 |
| ENSG00000184743 | 0.027831947 | 0.038926344 | 0.484005026 | 0.001702868 | 0.008440638 | ATL3 |
| ENSG00000075785 | 0.030554468 | 0.042585835 | 0.478990248 | 3.60E-05 | 0.000328072 | RAB7A |
| ENSG00000130175 | 0.074160363 | 0.102462829 | 0.466380421 | 8.47E-05 | 0.000689354 | PRKCSH |
| ENSG00000100030 | 0.024906969 | 0.034021576 | 0.449900491 | 0.001751947 | 0.008637621 | MAPK1 |
| ENSG00000215301 | 0.030321052 | 0.040283883 | 0.409882947 | 0.000478821 | 0.002957277 | DDX3X |
| ENSG00000164733 | 0.026359855 | 0.034408711 | 0.384431414 | 0.000470738 | 0.002913345 | CTSB |
| ENSG00000104738 | 0.058025398 | 0.072000369 | 0.311319795 | 0.00079766 | 0.004515941 | MCM4 |
| ENSG00000044115 | 0.034663452 | 0.027497926 | -0.334092529 | 3.25E-05 | 0.000300932 | CTNNA1 |
| ENSG00000137807 | 0.04838165 | 0.037101272 | -0.382991309 | 0.001890276 | 0.00916555 | KIF23 |
| ENSG00000177469 | 0.057365731 | 0.042526772 | -0.431817818 | 0.000140252 | 0.001060711 | CAVIN1 |
| ENSG00000170027 | 0.070398175 | 0.0521762 | -0.432146148 | 0.000116452 | 0.000906951 | YWHAG |
| ENSG00000198727 | 0.024007424 | 0.017407194 | -0.463796928 | 7.87E-05 | 0.000647831 | MT-CYB |
| ENSG00000108654 | 0.065785837 | 0.046672198 | -0.495213608 | 0.001509641 | 0.007628601 | DDX5 |
| ENSG00000170017 | 0.038949686 | 0.027533789 | -0.500408582 | 2.52E-06 | 3.18E-05 | ALCAM |
| ENSG00000136937 | 0.078756215 | 0.05500111 | -0.517933048 | 0.000792691 | 0.004496275 | NCBP1 |
| ENSG00000167996 | 0.03284878 | 0.022895477 | -0.520777154 | 0.00027527 | 0.001866963 | FTH1 |
| ENSG00000197451 | 0.043285304 | 0.030029905 | -0.527477409 | 0.001153346 | 0.006125877 | HNRNPAB |
| ENSG00000198888 | 0.034520664 | 0.023934545 | -0.52836585 | 8.13E-06 | 8.82E-05 | MT-ND1 |
| ENSG00000083642 | 0.104898336 | 0.072080744 | -0.541305976 | 0.00025675 | 0.00176626 | PDS5B |
| ENSG00000100644 | 0.07391326 | 0.050726073 | -0.543105745 | 4.72E-07 | 7.10E-06 | HIF1A |
| ENSG00000169592 | 0.148839988 | 0.100627595 | -0.564736188 | 0.000195586 | 0.001404134 | INO80E |
| ENSG00000048828 | 0.030577145 | 0.020621744 | -0.56828735 | 0.001204322 | 0.006332299 | FAM120A |
| ENSG00000205531 | 0.042770273 | 0.028462701 | -0.587535857 | 0.000501782 | 0.003080855 | NAP1L4 |
| ENSG00000124214 | 0.031037479 | 0.020653901 | -0.587597132 | 2.99E-06 | 3.68E-05 | STAU1 |
| ENSG00000092820 | 0.053525214 | 0.035584778 | -0.588958421 | 7.08E-07 | 1.02E-05 | EZR |
| ENSG00000114302 | 0.04841685 | 0.032187935 | -0.58898922 | 0.001242414 | 0.006504148 | PRKAR2A |
| ENSG00000119318 | 0.024739786 | 0.016382833 | -0.594648164 | 3.43E-05 | 0.000313774 | RAD23B |
| ENSG00000147862 | 0.08953468 | 0.059221813 | -0.596317928 | 1.57E-05 | 0.000158438 | NFIB |
| ENSG00000164754 | 0.049297093 | 0.03255444 | -0.598648236 | 0.001558584 | 0.007838551 | RAD21 |
| ENSG00000177479 | 0.062548014 | 0.041262728 | -0.600124886 | 0.001146793 | 0.006099155 | ARIH2 |
| ENSG00000100811 | 0.067421385 | 0.044229154 | -0.608208635 | 6.69E-07 | 9.72E-06 | YY1 |
| ENSG00000145386 | 0.056463464 | 0.036970949 | -0.610925565 | 2.12E-05 | 0.000205815 | CCNA2 |
| ENSG00000198668 | 0.052152183 | 0.034132647 | -0.611575346 | 0.001193527 | 0.006288344 | CALM1 |
| ENSG00000100401 | 0.03785312 | 0.024616933 | -0.620761224 | 6.75E-05 | 0.000564784 | RANGAP1 |
| ENSG00000114353 | 0.045699998 | 0.029706713 | -0.621405117 | 1.40E-13 | 6.53E-12 | GNAI2 |
| ENSG00000129351 | 0.041843521 | 0.027182826 | -0.622308797 | 0.001945089 | 0.009378386 | ILF3 |
| ENSG00000138757 | 0.057720085 | 0.037435498 | -0.624666496 | 1.78E-09 | 4.30E-08 | G3BP2 |
| ENSG00000204560 | 0.067516954 | 0.043780226 | -0.624970413 | 0.00058292 | 0.00349781 | DHX16 |
| ENSG00000137770 | 0.052605085 | 0.034050376 | -0.627531514 | 0.000934296 | 0.005130095 | CTDSPL2 |
| ENSG00000131437 | 0.06722624 | 0.04318766 | -0.638405294 | 0.002027372 | 0.009708941 | KIF3A |
| ENSG00000022840 | 0.026579017 | 0.017068383 | -0.638961332 | 0.000701168 | 0.004059483 | RNF10 |
| ENSG00000154380 | 0.062173083 | 0.039798702 | -0.643568769 | 2.01E-05 | 0.000195778 | ENAH |
| ENSG00000102119 | 0.068903144 | 0.043990231 | -0.647386637 | 0.000113163 | 0.000884202 | EMD |
| ENSG00000174780 | 0.053284312 | 0.033969813 | -0.649457574 | 4.32E-05 | 0.000383924 | SRP72 |
| ENSG00000009307 | 0.048309933 | 0.030762278 | -0.651157502 | 5.00E-15 | 2.87E-13 | CSDE1 |
| ENSG00000257103 | 0.029119705 | 0.018483178 | -0.655782922 | 0.001950872 | 0.009402497 | LSM14A |
| ENSG00000165934 | 0.061936072 | 0.039311312 | -0.655835384 | 1.45E-05 | 0.000147172 | CPSF2 |
| ENSG00000100664 | 0.040624728 | 0.025775539 | -0.656355535 | 0.000355853 | 0.002303084 | EIF5 |
| ENSG00000114062 | 0.06723609 | 0.042557589 | -0.65981941 | 0.001974829 | 0.009495128 | UBE3A |
| ENSG00000196923 | 0.040975148 | 0.025927462 | -0.660268185 | 0.000247647 | 0.001716078 | PDLIM7 |
| ENSG00000180628 | 0.044694707 | 0.028231812 | -0.662782254 | 0.000184972 | 0.001342359 | PCGF5 |
| ENSG00000073712 | 0.059385569 | 0.037442786 | -0.665424584 | 1.70E-05 | 0.000169365 | FERMT2 |
| ENSG00000099956 | 0.073608469 | 0.046307224 | -0.668634489 | 0.000601404 | 0.003587242 | SMARCB1 |
| ENSG00000113013 | 0.054336343 | 0.034105658 | -0.67190637 | 0.000130964 | 0.000999264 | HSPA9 |
| ENSG00000173039 | 0.058919955 | 0.036836732 | -0.677611234 | 0.001733915 | 0.008566279 | RELA |
| ENSG00000173020 | 0.025206655 | 0.015727673 | -0.680499422 | 0.000849877 | 0.004746758 | GRK2 |
| ENSG00000160633 | 0.125051565 | 0.077966076 | -0.681604687 | 6.07E-08 | 1.12E-06 | SAFB |
| ENSG00000070756 | 0.029975174 | 0.018635517 | -0.685713281 | 5.95E-08 | 1.10E-06 | PABPC1 |
| ENSG00000130703 | 0.059897066 | 0.037121789 | -0.6902191 | 0.001379229 | 0.007099842 | OSBPL2 |
| ENSG00000198363 | 0.066982389 | 0.041505906 | -0.690465189 | 7.66E-07 | 1.10E-05 | ASPH |
| ENSG00000143799 | 0.051279987 | 0.031667974 | -0.695371317 | 4.19E-08 | 8.01E-07 | PARP1 |
| ENSG00000153914 | 0.031624945 | 0.01951634 | -0.696380422 | 5.25E-06 | 6.01E-05 | SREK1 |
| ENSG00000163848 | 0.061917483 | 0.038082554 | -0.701216591 | 0.001690991 | 0.008395611 | ZNF148 |
| ENSG00000162434 | 0.066111556 | 0.040524775 | -0.706098303 | 0.000359026 | 0.002321124 | JAK1 |
| ENSG00000162923 | 0.042886659 | 0.026254649 | -0.707956014 | 7.60E-06 | 8.32E-05 | WDR26 |
| ENSG00000055917 | 0.040038601 | 0.024419779 | -0.71334143 | 2.08E-05 | 0.000202728 | PUM2 |
| ENSG00000138398 | 0.063493986 | 0.038549286 | -0.719915835 | 1.26E-05 | 0.000130533 | PPIG |
| ENSG00000140718 | 0.163041741 | 0.098984316 | -0.719969514 | 0.001014432 | 0.005489896 | FTO |
| ENSG00000172262 | 0.060464559 | 0.036679376 | -0.721120655 | 0.001459289 | 0.00742613 | ZNF131 |
| ENSG00000121152 | 0.079808918 | 0.04837552 | -0.722272795 | 9.32E-07 | 1.30E-05 | NCAPH |
| ENSG00000100353 | 0.055048952 | 0.033236371 | -0.727952236 | 0.000845289 | 0.00472333 | EIF3D |
| ENSG00000083799 | 0.041288676 | 0.024905523 | -0.729280453 | 0.000984392 | 0.005353403 | CYLD |
| ENSG00000176619 | 0.03460993 | 0.020864696 | -0.730122097 | 0.000845019 | 0.00472333 | LMNB2 |
| ENSG00000136824 | 0.068256758 | 0.041129295 | -0.73080555 | 5.00E-06 | 5.73E-05 | SMC2 |
| ENSG00000278311 | 0.050160007 | 0.03013396 | -0.735147308 | 0.00169843 | 0.008425584 | GGNBP2 |
| ENSG00000036054 | 0.073007549 | 0.043794094 | -0.737309317 | 0.001201741 | 0.00632426 | TBC1D23 |
| ENSG00000110321 | 0.028919422 | 0.017338229 | -0.738082178 | 3.27E-13 | 1.49E-11 | EIF4G2 |
| ENSG00000171206 | 0.026583815 | 0.015933298 | -0.738503291 | 5.36E-05 | 0.000461686 | TRIM8 |
| ENSG00000138434 | 0.041237337 | 0.024659263 | -0.741821479 | 4.89E-05 | 0.000426973 | ITPRID2 |
| ENSG00000028528 | 0.02875228 | 0.017169398 | -0.743836907 | 0.000136278 | 0.00103391 | SNX1 |
| ENSG00000103496 | 0.103847173 | 0.061836445 | -0.747932668 | 4.69E-07 | 7.06E-06 | STX4 |
| ENSG00000145725 | 0.056122731 | 0.033330808 | -0.751728903 | 1.45E-05 | 0.000147172 | PPIP5K2 |
| ENSG00000108953 | 0.085646992 | 0.050752088 | -0.75493541 | 0.00072688 | 0.00417817 | YWHAE |
| ENSG00000148468 | 0.040324003 | 0.02387911 | -0.755889799 | 0.00035667 | 0.002307133 | FAM171A1 |
| ENSG00000075711 | 0.048950189 | 0.028967587 | -0.756874893 | 0.000185191 | 0.001343135 | DLG1 |
| ENSG00000140750 | 0.057874013 | 0.034242441 | -0.757130112 | 0.001896783 | 0.009189694 | ARHGAP17 |
| ENSG00000112983 | 0.081551563 | 0.048159782 | -0.759883675 | 9.09E-08 | 1.59E-06 | BRD8 |
| ENSG00000131504 | 0.057160973 | 0.033751359 | -0.760084875 | 0.001411931 | 0.007237198 | DIAPH1 |
| ENSG00000163946 | 0.068462912 | 0.040412902 | -0.760506709 | 0.000121011 | 0.000936391 | FAM208A |
| ENSG00000101577 | 0.037392249 | 0.022071014 | -0.760586344 | 0.000888402 | 0.004918526 | LPIN2 |
| ENSG00000213341 | 0.050179759 | 0.029613822 | -0.760834837 | 0.000970025 | 0.005294849 | CHUK |
| ENSG00000100106 | 0.032458545 | 0.019146746 | -0.761499125 | 0.000968733 | 0.005290196 | TRIOBP |
| ENSG00000160014 | 0.034432499 | 0.0202292 | -0.767331663 | 8.89E-08 | 1.56E-06 | CALM3 |
| ENSG00000198899 | 0.005214351 | 0.003062362 | -0.767842922 | 0.000280694 | 0.001899463 | MT-ATP6 |
| ENSG00000170561 | 0.076068739 | 0.044652469 | -0.768563754 | 0.00091071 | 0.005023524 | IRX2 |
| ENSG00000149187 | 0.033332945 | 0.019545446 | -0.770116304 | 0.000200691 | 0.001432228 | CELF1 |
| ENSG00000011485 | 0.083152451 | 0.048689953 | -0.772134677 | 4.84E-07 | 7.25E-06 | PPP5C |
| ENSG00000127022 | 0.109700352 | 0.064230517 | -0.772237345 | 2.53E-07 | 4.03E-06 | CANX |
| ENSG00000104522 | 0.07551374 | 0.044209917 | -0.77236913 | 0.001925908 | 0.009312038 | TSTA3 |
| ENSG00000119541 | 0.104839332 | 0.06131181 | -0.773943169 | 0.000258216 | 0.001774312 | VPS4B |
| ENSG00000112715 | 0.047822765 | 0.027951038 | -0.774795703 | 0.001117479 | 0.00596965 | VEGFA |
| ENSG00000179335 | 0.059130154 | 0.034541618 | -0.775558377 | 0.000235153 | 0.001639254 | CLK3 |
| ENSG00000111615 | 0.060089987 | 0.035083898 | -0.776315555 | 0.000556903 | 0.003368588 | KRR1 |
| ENSG00000149657 | 0.04392078 | 0.025636681 | -0.776694159 | 0.001591157 | 0.007966855 | LSM14B |
| ENSG00000160949 | 0.034443946 | 0.020013438 | -0.783281401 | 0.000680816 | 0.00397225 | TONSL |
| ENSG00000172775 | 0.060827239 | 0.035338448 | -0.783478822 | 0.000819131 | 0.004611445 | FAM192A |
| ENSG00000153560 | 0.029170286 | 0.016945432 | -0.783603122 | 0.000143742 | 0.00108099 | UBP1 |
| ENSG00000113282 | 0.077145934 | 0.044787607 | -0.784490542 | 7.52E-13 | 3.15E-11 | CLINT1 |
| ENSG00000065485 | 0.114252005 | 0.066252307 | -0.786176875 | 0.000973755 | 0.005307981 | PDIA5 |
| ENSG00000198730 | 0.086049015 | 0.049804503 | -0.788882499 | 1.31E-06 | 1.78E-05 | CTR9 |
| ENSG00000127870 | 0.053287169 | 0.030811834 | -0.790303631 | 3.08E-05 | 0.000287956 | RNF6 |
| ENSG00000102910 | 0.077518502 | 0.044731656 | -0.793244522 | 0.000526562 | 0.003209278 | LONP2 |
| ENSG00000125944 | 0.043072066 | 0.024827434 | -0.794817374 | 3.26E-05 | 0.000301396 | HNRNPR |
| ENSG00000115053 | 0.274341695 | 0.157723271 | -0.79857837 | 0.000229103 | 0.00160359 | NCL |
| ENSG00000142731 | 0.027087844 | 0.015559109 | -0.799886119 | 0.001825296 | 0.008942866 | PLK4 |
| ENSG00000029993 | 0.052982659 | 0.030327464 | -0.804895383 | 0.001409061 | 0.007228651 | HMGB3 |
| ENSG00000197712 | 0.082540916 | 0.04724024 | -0.805093152 | 9.54E-08 | 1.66E-06 | FAM114A1 |
| ENSG00000101199 | 0.030250533 | 0.017308326 | -0.805494363 | 8.07E-05 | 0.000661632 | ARFGAP1 |
| ENSG00000198886 | 0.024331676 | 0.013916377 | -0.806052081 | 3.83E-06 | 4.58E-05 | MT-ND4 |
| ENSG00000089351 | 0.058915664 | 0.033643974 | -0.808303129 | 0.000488895 | 0.003013307 | GRAMD1A |
| ENSG00000179218 | 0.14397395 | 0.082186069 | -0.808842032 | 0 | 0 | CALR |
| ENSG00000137831 | 0.0540693 | 0.030815087 | -0.811172842 | 2.44E-08 | 4.86E-07 | UACA |
| ENSG00000187555 | 0.042490903 | 0.024208535 | -0.811638242 | 0.000343494 | 0.002238763 | USP7 |
| ENSG00000154845 | 0.061531446 | 0.034991212 | -0.81433128 | 2.61E-09 | 6.16E-08 | PPP4R1 |
| ENSG00000107897 | 0.068727735 | 0.039043497 | -0.815810115 | 5.38E-05 | 0.000462725 | ACBD5 |
| ENSG00000102081 | 0.061203642 | 0.034732951 | -0.817312526 | 5.47E-07 | 8.11E-06 | FMR1 |
| ENSG00000153827 | 0.043083716 | 0.024438591 | -0.817981568 | 1.85E-05 | 0.000182654 | TRIP12 |
| ENSG00000112763 | 0.062117611 | 0.035137948 | -0.821972403 | 0.000272024 | 0.00185016 | BTN2A1 |
| ENSG00000179262 | 0.081064739 | 0.045848769 | -0.822191512 | 0.000173632 | 0.001270803 | RAD23A |
| ENSG00000163781 | 0.033792828 | 0.019100405 | -0.823113836 | 0.001131466 | 0.006028301 | TOPBP1 |
| ENSG00000148498 | 0.070404273 | 0.039770893 | -0.823950036 | 0.000169419 | 0.001246032 | PARD3 |
| ENSG00000072110 | 0.092670002 | 0.052300837 | -0.825268367 | 0.000427421 | 0.002688146 | ACTN1 |
| ENSG00000198231 | 0.047577531 | 0.026828753 | -0.826500373 | 1.67E-09 | 4.07E-08 | DDX42 |
| ENSG00000113163 | 0.077610457 | 0.043720279 | -0.827948446 | 1.05E-06 | 1.46E-05 | COL4A3BP |
| ENSG00000163811 | 0.102301779 | 0.057579172 | -0.829212294 | 2.36E-06 | 3.02E-05 | WDR43 |
| ENSG00000144224 | 0.076385773 | 0.042922156 | -0.831581422 | 5.30E-08 | 9.85E-07 | UBXN4 |
| ENSG00000070495 | 0.091486123 | 0.051402658 | -0.831709956 | 1.59E-05 | 0.000159621 | JMJD6 |
| ENSG00000124783 | 0.110337723 | 0.061968681 | -0.832314952 | 9.85E-07 | 1.37E-05 | SSR1 |
| ENSG00000119335 | 0.029974183 | 0.016826286 | -0.833003686 | 0.000424767 | 0.002678461 | SET |
| ENSG00000173848 | 0.032381458 | 0.018174736 | -0.833233504 | 0.000117199 | 0.00091218 | NET1 |
| ENSG00000107341 | 0.055605477 | 0.03118489 | -0.834379795 | 0.001827489 | 0.008946348 | UBE2R2 |
| ENSG00000056097 | 0.070698371 | 0.039610454 | -0.835795726 | 3.11E-07 | 4.87E-06 | ZFR |
| ENSG00000100941 | 0.061048367 | 0.034193949 | -0.836211649 | 2.55E-07 | 4.05E-06 | PNN |
| ENSG00000112118 | 0.103744518 | 0.0579589 | -0.839932977 | 0 | 0 | MCM3 |
| ENSG00000141568 | 0.025979336 | 0.014510767 | -0.840240777 | 0.001705288 | 0.008449148 | FOXK2 |
| ENSG00000139697 | 0.140948822 | 0.078641127 | -0.841815506 | 1.78E-07 | 2.95E-06 | SBNO1 |
| ENSG00000135052 | 0.044783962 | 0.024982545 | -0.842061696 | 4.69E-06 | 5.43E-05 | GOLM1 |
| ENSG00000164134 | 0.083233321 | 0.046426971 | -0.842198042 | 1.89E-07 | 3.10E-06 | NAA15 |
| ENSG00000110075 | 0.145254694 | 0.080867937 | -0.844945078 | 0.00119387 | 0.006288344 | PPP6R3 |
| ENSG00000054118 | 0.05527686 | 0.030709878 | -0.847972863 | 4.46E-05 | 0.000393937 | THRAP3 |
| ENSG00000108406 | 0.049828345 | 0.027680661 | -0.848088246 | 5.16E-05 | 0.00044751 | DHX40 |
| ENSG00000109917 | 0.079784017 | 0.044260193 | -0.850090006 | 0.001959828 | 0.009438096 | ZPR1 |
| ENSG00000160741 | 0.041344156 | 0.022933614 | -0.850219707 | 0.000108607 | 0.000852478 | CRTC2 |
| ENSG00000143337 | 0.066554656 | 0.036864675 | -0.85230055 | 5.13E-05 | 0.000445291 | TOR1AIP1 |
| ENSG00000130826 | 0.098432247 | 0.054305526 | -0.858032028 | 0.000315965 | 0.002093549 | DKC1 |
| ENSG00000132002 | 0.043001753 | 0.02370666 | -0.859103088 | 4.41E-06 | 5.17E-05 | DNAJB1 |
| ENSG00000117020 | 0.070152682 | 0.038649181 | -0.860060441 | 0.00209712 | 0.00999519 | AKT3 |
| ENSG00000115694 | 0.023625998 | 0.013013062 | -0.860414849 | 0.000410977 | 0.002610672 | STK25 |
| ENSG00000039560 | 0.046002706 | 0.025313552 | -0.861808755 | 5.95E-06 | 6.69E-05 | RAI14 |
| ENSG00000060069 | 0.03791084 | 0.020859819 | -0.861883769 | 0.000594921 | 0.003559168 | CTDP1 |
| ENSG00000105486 | 0.085016655 | 0.046716969 | -0.863798816 | 1.17E-06 | 1.60E-05 | LIG1 |
| ENSG00000123562 | 0.04496108 | 0.024699212 | -0.864211683 | 0.000530798 | 0.003233457 | MORF4L2 |
| ENSG00000141179 | 0.071884157 | 0.039474422 | -0.864755712 | 0.001031626 | 0.005570412 | PCTP |
| ENSG00000278053 | 0.060435669 | 0.03317564 | -0.865275983 | 0.00034778 | 0.002260572 | DDX52 |
| ENSG00000135842 | 0.061171166 | 0.033570954 | -0.865638213 | 0.000741539 | 0.004248219 | FAM129A |
| ENSG00000153561 | 0.043182534 | 0.023696231 | -0.865790279 | 0.001786841 | 0.008784451 | RMND5A |
| ENSG00000106299 | 0.067275099 | 0.036836115 | -0.868951698 | 0.000217373 | 0.00153129 | WASL |
| ENSG00000091127 | 0.100160701 | 0.054832291 | -0.869218896 | 4.18E-05 | 0.000373552 | PUS7 |
| ENSG00000125827 | 0.049815323 | 0.027266755 | -0.869446555 | 7.61E-05 | 0.000628805 | TMX4 |
| ENSG00000083520 | 0.072993123 | 0.03993386 | -0.870148009 | 1.60E-06 | 2.13E-05 | DIS3 |
| ENSG00000117751 | 0.034262989 | 0.018739398 | -0.870576422 | 0.000233816 | 0.001631825 | PPP1R8 |
| ENSG00000111364 | 0.052067249 | 0.028475801 | -0.870639733 | 0.001688443 | 0.008391185 | DDX55 |
| ENSG00000090889 | 0.069103826 | 0.037786484 | -0.870895296 | 2.31E-06 | 2.97E-05 | KIF4A |
| ENSG00000008130 | 0.03222953 | 0.017620392 | -0.871137137 | 3.70E-05 | 0.000335167 | NADK |
| ENSG00000137942 | 0.059222604 | 0.032341482 | -0.872762108 | 0.000760096 | 0.004335939 | FNBP1L |
| ENSG00000178691 | 0.059158245 | 0.032295479 | -0.873247065 | 2.45E-05 | 0.000234754 | SUZ12 |
| ENSG00000085733 | 0.040055898 | 0.021860786 | -0.873669403 | 6.57E-08 | 1.20E-06 | CTTN |
| ENSG00000177885 | 0.034554252 | 0.018833582 | -0.875555883 | 0.000172598 | 0.001265417 | GRB2 |
| ENSG00000159256 | 0.094315509 | 0.051400783 | -0.875704691 | 6.36E-06 | 7.08E-05 | MORC3 |
| ENSG00000141644 | 0.028601468 | 0.015582639 | -0.876149605 | 0.000548306 | 0.003324952 | MBD1 |
| ENSG00000198586 | 0.049535145 | 0.026932586 | -0.87909975 | 0.00019445 | 0.001398481 | TLK1 |
| ENSG00000153187 | 0.075826137 | 0.04122635 | -0.879128481 | 0 | 0 | HNRNPU |
| ENSG00000102038 | 0.069421978 | 0.037716139 | -0.88021046 | 0.00138158 | 0.007108903 | SMARCA1 |
| ENSG00000204130 | 0.066737201 | 0.036199293 | -0.882529685 | 0.001179431 | 0.006231396 | RUFY2 |
| ENSG00000090060 | 0.02494561 | 0.013526456 | -0.883001983 | 3.64E-08 | 7.05E-07 | PAPOLA |
| ENSG00000143164 | 0.05619538 | 0.030417634 | -0.885543583 | 0.000100277 | 0.000797508 | DCAF6 |
| ENSG00000198648 | 0.073479423 | 0.039744665 | -0.886579093 | 0.001557805 | 0.007837909 | STK39 |
| ENSG00000055130 | 0.072439484 | 0.039178484 | -0.886714695 | 1.65E-09 | 4.03E-08 | CUL1 |
| ENSG00000275700 | 0.102328992 | 0.055296685 | -0.887950052 | 0.000392629 | 0.002503377 | AATF |
| ENSG00000130254 | 0.237138157 | 0.127806911 | -0.891761968 | 4.94E-14 | 2.43E-12 | SAFB2 |
| ENSG00000160214 | 0.088956457 | 0.047890883 | -0.893348288 | 0.001241868 | 0.00650412 | RRP1 |
| ENSG00000168813 | 0.056076313 | 0.030157144 | -0.894891694 | 0.002073608 | 0.009898821 | ZNF507 |
| ENSG00000115993 | 0.053084851 | 0.02854453 | -0.89508592 | 0.000469901 | 0.002909661 | TRAK2 |
| ENSG00000165732 | 0.05624406 | 0.030215131 | -0.89642955 | 3.69E-08 | 7.12E-07 | DDX21 |
| ENSG00000146909 | 0.075818812 | 0.040697127 | -0.897628911 | 8.08E-06 | 8.77E-05 | NOM1 |
| ENSG00000198826 | 0.019046939 | 0.010220913 | -0.898035001 | 4.01E-05 | 0.000359969 | ARHGAP11A |
| ENSG00000077097 | 0.056002542 | 0.029994807 | -0.900779569 | 3.57E-09 | 8.25E-08 | TOP2B |
| ENSG00000187954 | 0.014630579 | 0.007822042 | -0.903369681 | 0.002005438 | 0.009626905 | CYHR1 |
| ENSG00000164045 | 0.053183377 | 0.028421887 | -0.903973037 | 0.000182843 | 0.001329317 | CDC25A |
| ENSG00000151726 | 0.08818063 | 0.047039267 | -0.906596196 | 0.001302986 | 0.00675986 | ACSL1 |
| ENSG00000180104 | 0.072343681 | 0.038568395 | -0.907447889 | 1.94E-09 | 4.66E-08 | EXOC3 |
| ENSG00000101057 | 0.042114392 | 0.022442898 | -0.908054364 | 1.53E-12 | 6.17E-11 | MYBL2 |
| ENSG00000075413 | 0.038620287 | 0.020571281 | -0.908727266 | 4.43E-06 | 5.18E-05 | MARK3 |
| ENSG00000133612 | 0.027746034 | 0.014772285 | -0.909388622 | 0.000315991 | 0.002093549 | AGAP3 |
| ENSG00000125351 | 0.099816327 | 0.053120078 | -0.910018537 | 0.001967564 | 0.009467768 | UPF3B |
| ENSG00000255717 | 19.35072366 | 10.2897991 | -0.911172705 | 0.000720672 | 0.004146448 | SNHG1 |
| ENSG00000113810 | 0.068220028 | 0.036099461 | -0.918218045 | 4.04E-06 | 4.79E-05 | SMC4 |
| ENSG00000176444 | 0.117601106 | 0.062214255 | -0.918584547 | 7.67E-06 | 8.38E-05 | CLK2 |
| ENSG00000137177 | 0.061074264 | 0.032285937 | -0.919658674 | 0.002061524 | 0.009856792 | KIF13A |
| ENSG00000123136 | 0.091864392 | 0.048538944 | -0.920363041 | 0.001843724 | 0.008994231 | DDX39A |
| ENSG00000112531 | 0.068778727 | 0.036327699 | -0.920892435 | 2.93E-11 | 9.75E-10 | QKI |
| ENSG00000107815 | 0.07287646 | 0.03848628 | -0.921108643 | 0.002087289 | 0.009960176 | TWNK |
| ENSG00000128578 | 0.04052924 | 0.021377862 | -0.922845575 | 0.001251005 | 0.006534896 | STRIP2 |
| ENSG00000145388 | 0.09296596 | 0.04894288 | -0.925603562 | 0.000483945 | 0.002984325 | METTL14 |
| ENSG00000023287 | 0.061989192 | 0.032583862 | -0.92785911 | 5.90E-06 | 6.65E-05 | RB1CC1 |
| ENSG00000176915 | 0.06952282 | 0.036529848 | -0.928410855 | 4.42E-13 | 1.97E-11 | ANKLE2 |
| ENSG00000090372 | 0.08137778 | 0.042745961 | -0.928846825 | 2.18E-13 | 1.00E-11 | STRN4 |
| ENSG00000144029 | 0.155861349 | 0.081612397 | -0.933402989 | 0.000629575 | 0.003734901 | MRPS5 |
| ENSG00000150403 | 0.078892667 | 0.04117309 | -0.938189495 | 3.63E-06 | 4.36E-05 | TMCO3 |
| ENSG00000050405 | 0.074496149 | 0.038862451 | -0.938788947 | 5.82E-10 | 1.56E-08 | LIMA1 |
| ENSG00000137275 | 0.05986652 | 0.031180456 | -0.941107381 | 8.25E-05 | 0.000674749 | RIPK1 |
| ENSG00000131263 | 0.036521489 | 0.019010277 | -0.941966049 | 9.20E-05 | 0.00073915 | RLIM |
| ENSG00000131747 | 0.064241553 | 0.033419179 | -0.942830473 | 5.19E-07 | 7.71E-06 | TOP2A |
| ENSG00000196365 | 0.077101614 | 0.040079596 | -0.943893098 | 2.44E-07 | 3.90E-06 | LONP1 |
| ENSG00000005889 | 0.055584297 | 0.028889861 | -0.944114083 | 0.001861014 | 0.009060198 | ZFX |
| ENSG00000071189 | 0.045970971 | 0.023862504 | -0.945977729 | 0.00121946 | 0.006403496 | SNX13 |
| ENSG00000178913 | 0.074007467 | 0.038385439 | -0.9471117 | 0.001729414 | 0.008553583 | TAF7 |
| ENSG00000179295 | 0.030218745 | 0.015662239 | -0.948153282 | 0.000330755 | 0.002172219 | PTPN11 |
| ENSG00000055483 | 0.046291981 | 0.023971726 | -0.949428502 | 0.001807016 | 0.008865511 | USP36 |
| ENSG00000132561 | 0.148600834 | 0.076915274 | -0.950100188 | 0.001804467 | 0.00885662 | MATN2 |
| ENSG00000102786 | 0.049433022 | 0.025558365 | -0.951679596 | 0.000253838 | 0.001750234 | INTS6 |
| ENSG00000154832 | 0.031068859 | 0.01605724 | -0.952245288 | 8.42E-05 | 0.000686096 | CXXC1 |
| ENSG00000167657 | 0.060464516 | 0.031212626 | -0.953958976 | 0.000838103 | 0.004694613 | DAPK3 |
| ENSG00000141002 | 0.120896136 | 0.062233442 | -0.958006185 | 0.000641747 | 0.003790277 | TCF25 |
| ENSG00000127054 | 0.072995201 | 0.03756591 | -0.958377562 | 1.56E-08 | 3.22E-07 | INTS11 |
| ENSG00000142002 | 0.039195001 | 0.020170195 | -0.958444657 | 0.001356083 | 0.00699241 | DPP9 |
| ENSG00000122417 | 0.156741351 | 0.080566778 | -0.960128868 | 3.09E-05 | 0.000288585 | ODF2L |
| ENSG00000134779 | 0.047952143 | 0.024636288 | -0.960810409 | 5.44E-11 | 1.70E-09 | TPGS2 |
| ENSG00000100109 | 0.080979254 | 0.041603059 | -0.960862746 | 0.000179505 | 0.001308214 | TFIP11 |
| ENSG00000125733 | 0.077169979 | 0.03962953 | -0.961463873 | 1.15E-14 | 6.20E-13 | TRIP10 |
| ENSG00000146463 | 0.048005159 | 0.024625225 | -0.963052525 | 0.000600164 | 0.003581626 | ZMYM4 |
| ENSG00000145868 | 0.096839088 | 0.049660683 | -0.963485406 | 1.16E-05 | 0.0001217 | FBXO38 |
| ENSG00000170759 | 0.085964781 | 0.04408158 | -0.963569801 | 0 | 0 | KIF5B |
| ENSG00000156273 | 0.064316135 | 0.032976616 | -0.963737376 | 8.32E-05 | 0.000679417 | BACH1 |
| ENSG00000126457 | 0.080362501 | 0.041169779 | -0.964936771 | 2.95E-06 | 3.63E-05 | PRMT1 |
| ENSG00000120910 | 0.067308889 | 0.034438077 | -0.966792435 | 0.001974642 | 0.009495128 | PPP3CC |
| ENSG00000101109 | 0.069572285 | 0.035551738 | -0.968592626 | 0.000366904 | 0.002366238 | STK4 |
| ENSG00000185104 | 0.099348293 | 0.050742788 | -0.969292401 | 1.33E-06 | 1.79E-05 | FAF1 |
| ENSG00000174371 | 0.052907847 | 0.026997934 | -0.970632714 | 4.31E-05 | 0.000383282 | EXO1 |
| ENSG00000118260 | 0.061814799 | 0.031536813 | -0.970915398 | 0.001177722 | 0.006227839 | CREB1 |
| ENSG00000187514 | 0.079928367 | 0.040757103 | -0.971656106 | 0.000360039 | 0.002326423 | PTMA |
| ENSG00000168234 | 0.107303832 | 0.054701032 | -0.972061652 | 1.93E-05 | 0.000189427 | TTC39C |
| ENSG00000167257 | 0.170911776 | 0.087120793 | -0.972162818 | 0.001736234 | 0.008570696 | RNF214 |
| ENSG00000102189 | 0.11835499 | 0.06020257 | -0.975223548 | 9.30E-06 | 9.97E-05 | EEA1 |
| ENSG00000010810 | 0.060678754 | 0.03082985 | -0.976863617 | 0.000738772 | 0.004237157 | FYN |
| ENSG00000156976 | 0.049432034 | 0.025100647 | -0.977721709 | 0.000102767 | 0.00081451 | EIF4A2 |
| ENSG00000104131 | 0.115146785 | 0.058414076 | -0.979086164 | 1.91E-05 | 0.000187667 | EIF3J |
| ENSG00000134684 | 0.047567982 | 0.024098444 | -0.981050847 | 3.45E-07 | 5.35E-06 | YARS |
| ENSG00000164741 | 0.095944823 | 0.048590426 | -0.981532871 | 0.000280052 | 0.001896187 | DLC1 |
| ENSG00000123064 | 0.062600177 | 0.031690373 | -0.982122073 | 0.000110276 | 0.000864449 | DDX54 |
| ENSG00000132485 | 0.061591719 | 0.031152193 | -0.983402667 | 0.000413766 | 0.002622844 | ZRANB2 |
| ENSG00000142192 | 0.069359401 | 0.03506025 | -0.984255153 | 5.96E-09 | 1.32E-07 | APP |
| ENSG00000100813 | 0.068668691 | 0.034702647 | -0.98460675 | 6.31E-08 | 1.16E-06 | ACIN1 |
| ENSG00000135801 | 0.042871441 | 0.021639731 | -0.986334323 | 0.001735211 | 0.008569162 | TAF5L |
| ENSG00000163535 | 0.033218226 | 0.016748435 | -0.987948787 | 0.000979898 | 0.005334209 | SGO2 |
| ENSG00000146833 | 0.095272298 | 0.047844843 | -0.99369335 | 6.20E-05 | 0.000527952 | TRIM4 |
| ENSG00000173011 | 0.066863604 | 0.033536771 | -0.995477325 | 7.11E-05 | 0.000590781 | TADA2B |
| ENSG00000125503 | 0.031035865 | 0.015558022 | -0.996277647 | 6.76E-05 | 0.000565038 | PPP1R12C |
| ENSG00000198836 | 0.06776399 | 0.033944003 | -0.9973621 | 7.96E-08 | 1.41E-06 | OPA1 |
| ENSG00000182272 | 0.052534711 | 0.026314836 | -0.997394542 | 0.000589603 | 0.003530864 | B4GALNT4 |
| ENSG00000131725 | 0.098923653 | 0.049493293 | -0.999082491 | 0.000269276 | 0.001837709 | WDR44 |
| ENSG00000166012 | 0.109970578 | 0.05493505 | -1.001318776 | 0.00025479 | 0.001755209 | TAF1D |
| ENSG00000068784 | 0.113787358 | 0.056744947 | -1.003776459 | 0.000464659 | 0.002884628 | SRBD1 |
| ENSG00000114867 | 0.093917663 | 0.046833213 | -1.003864486 | 9.59E-06 | 0.000102467 | EIF4G1 |
| ENSG00000136643 | 0.099744272 | 0.049734156 | -1.003996994 | 1.52E-07 | 2.56E-06 | RPS6KC1 |
| ENSG00000165244 | 0.039344173 | 0.019602606 | -1.005104498 | 0.000194657 | 0.001399131 | ZNF367 |
| ENSG00000105698 | 0.030489707 | 0.015188533 | -1.005339767 | 9.48E-06 | 0.000101452 | USF2 |
| ENSG00000137574 | 0.109344453 | 0.054469089 | -1.005370387 | 6.36E-06 | 7.08E-05 | TGS1 |
| ENSG00000153147 | 0.127674655 | 0.063478879 | -1.008123591 | 6.65E-07 | 9.67E-06 | SMARCA5 |
| ENSG00000129292 | 0.060161404 | 0.029832139 | -1.01197079 | 3.78E-05 | 0.000341467 | PHF20L1 |
| ENSG00000124831 | 0.049544946 | 0.024554286 | -1.012763032 | 0.000348904 | 0.002265425 | LRRFIP1 |
| ENSG00000155660 | 0.075431036 | 0.037362852 | -1.013553651 | 3.62E-13 | 1.63E-11 | PDIA4 |
| ENSG00000198786 | 0.14857794 | 0.073567349 | -1.01408242 | 3.14E-05 | 0.000292856 | MT-ND5 |
| ENSG00000137601 | 0.117833856 | 0.05829785 | -1.015239525 | 4.03E-05 | 0.000361171 | NEK1 |
| ENSG00000164902 | 0.062843809 | 0.031081633 | -1.015708342 | 9.81E-06 | 0.000104681 | PHAX |
| ENSG00000112941 | 0.029503849 | 0.014579839 | -1.016928408 | 6.08E-05 | 0.000518558 | TENT4A |
| ENSG00000070540 | 0.075456492 | 0.037274227 | -1.017466598 | 0.001749654 | 0.008629857 | WIPI1 |
| ENSG00000117616 | 0.044601777 | 0.022029295 | -1.017677861 | 0.001182394 | 0.006241566 | RSRP1 |
| ENSG00000060138 | 0.045002389 | 0.022224232 | -1.017868041 | 1.64E-05 | 0.000164387 | YBX3 |
| ENSG00000119906 | 0.022976196 | 0.011339634 | -1.018765895 | 0.000425632 | 0.002682054 | SLF2 |
| ENSG00000100796 | 0.077615733 | 0.038305659 | -1.018791581 | 9.65E-05 | 0.000771877 | PPP4R3A |
| ENSG00000166938 | 0.059226036 | 0.029216939 | -1.019426471 | 6.10E-05 | 0.000520378 | DIS3L |
| ENSG00000177732 | 0.058611029 | 0.028890814 | -1.020561331 | 1.87E-08 | 3.81E-07 | SOX12 |
| ENSG00000181163 | 0.05202931 | 0.025632904 | -1.021327623 | 0.001772209 | 0.008719644 | NPM1 |
| ENSG00000116584 | 0.037454828 | 0.018434544 | -1.02273996 | 2.23E-05 | 0.000216084 | ARHGEF2 |
| ENSG00000072786 | 0.096349994 | 0.047413584 | -1.02298411 | 5.38E-05 | 0.000462798 | STK10 |
| ENSG00000011426 | 0.064810575 | 0.031862144 | -1.02438588 | 0 | 0 | ANLN |
| ENSG00000084234 | 0.051554645 | 0.025306017 | -1.026621975 | 0 | 0 | APLP2 |
| ENSG00000180354 | 0.054657772 | 0.0267971 | -1.028349785 | 0.00027698 | 0.001877503 | MTURN |
| ENSG00000138182 | 0.087765006 | 0.043006293 | -1.029098027 | 0.002011398 | 0.009643965 | KIF20B |
| ENSG00000186834 | 0.141443018 | 0.06930632 | -1.029162153 | 2.92E-07 | 4.61E-06 | HEXIM1 |
| ENSG00000106554 | 0.047513667 | 0.023276119 | -1.029492056 | 0.001056631 | 0.005687552 | CHCHD3 |
| ENSG00000100592 | 0.106391151 | 0.05209986 | -1.030026763 | 0.001850576 | 0.009016686 | DAAM1 |
| ENSG00000143401 | 0.122807534 | 0.060103708 | -1.030873165 | 3.98E-06 | 4.72E-05 | ANP32E |
| ENSG00000179364 | 0.039590568 | 0.019376134 | -1.030876016 | 0.000396298 | 0.002524091 | PACS2 |
| ENSG00000090273 | 0.143417901 | 0.070152011 | -1.031668743 | 6.20E-05 | 0.000527952 | NUDC |
| ENSG00000260032 | 0.006681624 | 0.003268032 | -1.031776757 | 0.000322584 | 0.002130189 | NORAD |
| ENSG00000152409 | 0.095212063 | 0.046541489 | -1.032627008 | 1.56E-05 | 0.000158088 | JMY |
| ENSG00000131023 | 0.055838929 | 0.027290496 | -1.032872685 | 0.000143968 | 0.001082009 | LATS1 |
| ENSG00000185246 | 0.052539265 | 0.025673972 | -1.0330895 | 0.000197103 | 0.001412493 | PRPF39 |
| ENSG00000130770 | 0.172089649 | 0.084035981 | -1.034081256 | 0.000758551 | 0.004329179 | ATP5IF1 |
| ENSG00000104067 | 0.069675961 | 0.034003013 | -1.034998388 | 0.000366988 | 0.002366238 | TJP1 |
| ENSG00000185963 | 0.080555662 | 0.03924995 | -1.037295182 | 2.84E-05 | 0.000269142 | BICD2 |
| ENSG00000143228 | 0.120999336 | 0.058952299 | -1.037379138 | 0.000217324 | 0.00153129 | NUF2 |
| ENSG00000087152 | 0.032580817 | 0.015868759 | -1.037833476 | 0.000164729 | 0.001218248 | ATXN7L3 |
| ENSG00000134597 | 0.092038456 | 0.044798973 | -1.038771112 | 0.001324111 | 0.006847445 | RBMX2 |
| ENSG00000188641 | 0.148575893 | 0.072108351 | -1.042961793 | 3.65E-05 | 0.000331708 | DPYD |
| ENSG00000138162 | 0.105397658 | 0.051139302 | -1.043338432 | 1.21E-06 | 1.66E-05 | TACC2 |
| ENSG00000198356 | 0.06272453 | 0.03043387 | -1.043351964 | 1.93E-06 | 2.53E-05 | ASNA1 |
| ENSG00000108588 | 0.132596585 | 0.064327272 | -1.043541208 | 3.51E-08 | 6.82E-07 | CCDC47 |
| ENSG00000168066 | 0.058024414 | 0.028119985 | -1.045064196 | 1.91E-07 | 3.12E-06 | SF1 |
| ENSG00000162889 | 0.065372116 | 0.031670722 | -1.045525653 | 1.73E-06 | 2.28E-05 | MAPKAPK2 |
| ENSG00000067334 | 0.099626662 | 0.048064809 | -1.051550894 | 0.000809365 | 0.0045693 | DNTTIP2 |
| ENSG00000103540 | 0.030791835 | 0.014846693 | -1.052406286 | 0.000520585 | 0.003180912 | CCP110 |
| ENSG00000136436 | 0.090199416 | 0.043427135 | -1.054521311 | 1.75E-09 | 4.26E-08 | CALCOCO2 |
| ENSG00000146112 | 0.039446309 | 0.018986016 | -1.054953104 | 1.36E-06 | 1.83E-05 | PPP1R18 |
| ENSG00000108395 | 0.067465568 | 0.032469501 | -1.055066186 | 0 | 0 | TRIM37 |
| ENSG00000142208 | 0.03249822 | 0.015624117 | -1.056585988 | 1.93E-05 | 0.000189427 | AKT1 |
| ENSG00000163961 | 0.091627424 | 0.04399476 | -1.05844774 | 0.00013424 | 0.001021019 | RNF168 |
| ENSG00000086061 | 0.061596126 | 0.029533465 | -1.060489 | 5.27E-05 | 0.000455525 | DNAJA1 |
| ENSG00000131323 | 0.029010681 | 0.013908011 | -1.060668027 | 0.000468908 | 0.002905009 | TRAF3 |
| ENSG00000140350 | 0.102323313 | 0.04894589 | -1.063875248 | 0.000478616 | 0.002957277 | ANP32A |
| ENSG00000152465 | 0.046350957 | 0.02215369 | -1.065052115 | 8.90E-05 | 0.000717944 | NMT2 |
| ENSG00000198171 | 0.063353993 | 0.030267468 | -1.065667545 | 4.46E-05 | 0.000393937 | DDRGK1 |
| ENSG00000175029 | 0.033458633 | 0.015979391 | -1.066166065 | 9.23E-07 | 1.29E-05 | CTBP2 |
| ENSG00000031823 | 0.092214659 | 0.044039391 | -1.066201579 | 4.94E-07 | 7.37E-06 | RANBP3 |
| ENSG00000118503 | 0.04521549 | 0.021581006 | -1.067054995 | 3.00E-05 | 0.000282343 | TNFAIP3 |
| ENSG00000104731 | 0.088035875 | 0.041971866 | -1.068668934 | 6.23E-05 | 0.000529404 | KLHDC4 |
| ENSG00000215908 | 0.016208933 | 0.007720277 | -1.070064635 | 0.00106048 | 0.005705713 | CROCCP2 |
| ENSG00000111011 | 0.098987803 | 0.047018843 | -1.074011729 | 1.96E-05 | 0.000191547 | RSRC2 |
| ENSG00000129038 | 0.023274889 | 0.011054456 | -1.074146263 | 0.000331173 | 0.002173773 | LOXL1 |
| ENSG00000113300 | 0.053299089 | 0.025283393 | -1.075920802 | 3.62E-11 | 1.17E-09 | CNOT6 |
| ENSG00000100325 | 0.069181125 | 0.032815695 | -1.075992482 | 5.82E-07 | 8.56E-06 | ASCC2 |
| ENSG00000115904 | 0.035354533 | 0.016753733 | -1.077412569 | 0.001066099 | 0.00573338 | SOS1 |
| ENSG00000184428 | 0.096646764 | 0.045788163 | -1.077746749 | 3.83E-08 | 7.38E-07 | TOP1MT |
| ENSG00000065978 | 0.014527043 | 0.006878115 | -1.078655887 | 2.25E-05 | 0.000217945 | YBX1 |
| ENSG00000139746 | 0.074233879 | 0.035138913 | -1.079008179 | 6.83E-06 | 7.56E-05 | RBM26 |
| ENSG00000119041 | 0.107958778 | 0.051065234 | -1.080067219 | 6.66E-09 | 1.46E-07 | GTF3C3 |
| ENSG00000123485 | 0.069810433 | 0.033018023 | -1.080188937 | 8.99E-14 | 4.27E-12 | HJURP |
| ENSG00000100425 | 0.056246573 | 0.026590569 | -1.080850575 | 3.50E-07 | 5.40E-06 | BRD1 |
| ENSG00000198055 | 0.033768455 | 0.015962227 | -1.081014206 | 2.87E-08 | 5.65E-07 | GRK6 |
| ENSG00000186575 | 0.088224544 | 0.041671072 | -1.082133855 | 3.83E-07 | 5.85E-06 | NF2 |
| ENSG00000221978 | 0.035077355 | 0.01656144 | -1.082711787 | 9.71E-09 | 2.07E-07 | CCNL2 |
| ENSG00000067066 | 0.084295549 | 0.03976274 | -1.084039276 | 1.92E-12 | 7.56E-11 | SP100 |
| ENSG00000102580 | 0.075479636 | 0.035502495 | -1.088167049 | 2.54E-10 | 7.22E-09 | DNAJC3 |
| ENSG00000153015 | 0.0695637 | 0.032676449 | -1.090083458 | 0.001339982 | 0.006918544 | CWC27 |
| ENSG00000168172 | 0.106080261 | 0.049806861 | -1.09073984 | 2.86E-06 | 3.54E-05 | HOOK3 |
| ENSG00000169398 | 0.077137182 | 0.036190428 | -1.091818267 | 1.22E-06 | 1.67E-05 | PTK2 |
| ENSG00000144747 | 0.161051037 | 0.075537983 | -1.092243783 | 3.97E-12 | 1.46E-10 | TMF1 |
| ENSG00000041880 | 0.055245169 | 0.025909181 | -1.092384877 | 0.000389664 | 0.002488428 | PARP3 |
| ENSG00000119408 | 0.089850272 | 0.042124289 | -1.092870551 | 3.83E-08 | 7.38E-07 | NEK6 |
| ENSG00000123737 | 0.169767235 | 0.079585094 | -1.092987894 | 0.000203948 | 0.001449453 | EXOSC9 |
| ENSG00000108819 | 0.103130958 | 0.048261615 | -1.095529356 | 1.64E-07 | 2.74E-06 | PPP1R9B |
| ENSG00000078674 | 0.138577317 | 0.064847976 | -1.095557689 | 0 | 0 | PCM1 |
| ENSG00000164151 | 0.041814746 | 0.019558371 | -1.096225602 | 0.000223679 | 0.001572025 | ICE1 |
| ENSG00000177606 | 0.100945246 | 0.047169628 | -1.097642849 | 3.65E-09 | 8.41E-08 | JUN |
| ENSG00000104859 | 0.067901666 | 0.031678564 | -1.09994006 | 1.77E-06 | 2.33E-05 | CLASRP |
| ENSG00000115355 | 0.084138256 | 0.03924126 | -1.100390526 | 0.000464945 | 0.002884912 | CCDC88A |
| ENSG00000071564 | 0.021615647 | 0.01007577 | -1.101185896 | 8.32E-05 | 0.000679417 | TCF3 |
| ENSG00000143748 | 0.179566703 | 0.083684805 | -1.101482265 | 0.000110209 | 0.000864449 | NVL |
| ENSG00000163660 | 0.03852698 | 0.017953765 | -1.101582734 | 1.36E-07 | 2.31E-06 | CCNL1 |
| ENSG00000023330 | 0.087431462 | 0.040718018 | -1.102485203 | 1.53E-05 | 0.000154691 | ALAS1 |
| ENSG00000204569 | 0.051237974 | 0.023856697 | -1.102819123 | 0.000282214 | 0.001905707 | PPP1R10 |
| ENSG00000107949 | 0.148337629 | 0.069065849 | -1.1028402 | 0.001365425 | 0.007031791 | BCCIP |
| ENSG00000124201 | 0.054496615 | 0.025369508 | -1.103071075 | 0.000822318 | 0.004622894 | ZNFX1 |
| ENSG00000189060 | 0.021120051 | 0.009825091 | -1.104070572 | 1.24E-08 | 2.59E-07 | H1F0 |
| ENSG00000167110 | 0.049744339 | 0.023133501 | -1.104548728 | 4.99E-06 | 5.73E-05 | GOLGA2 |
| ENSG00000147065 | 0.062640978 | 0.029123021 | -1.104946724 | 5.85E-06 | 6.62E-05 | MSN |
| ENSG00000154920 | 0.059554115 | 0.027646186 | -1.107120709 | 0.00139098 | 0.007148093 | EME1 |
| ENSG00000087087 | 0.090470852 | 0.041972751 | -1.108000035 | 3.40E-07 | 5.29E-06 | SRRT |
| ENSG00000083535 | 0.134373606 | 0.062332672 | -1.108189333 | 0.000872221 | 0.004846793 | PIBF1 |
| ENSG00000044574 | 0.070235319 | 0.032528618 | -1.110487181 | 6.91E-09 | 1.51E-07 | HSPA5 |
| ENSG00000025293 | 0.064030796 | 0.029635815 | -1.111424243 | 2.32E-10 | 6.62E-09 | PHF20 |
| ENSG00000104979 | 0.152784428 | 0.070696745 | -1.111781822 | 0.000576903 | 0.003466893 | C19orf53 |
| ENSG00000078061 | 0.054616495 | 0.025270384 | -1.111889182 | 1.86E-09 | 4.48E-08 | ARAF |
| ENSG00000116871 | 0.035413647 | 0.016385156 | -1.111915962 | 1.11E-09 | 2.80E-08 | MAP7D1 |
| ENSG00000102974 | 0.058750993 | 0.027146792 | -1.113831503 | 3.54E-11 | 1.15E-09 | CTCF |
| ENSG00000214944 | 0.06808018 | 0.031451831 | -1.114090856 | 3.67E-05 | 0.000332751 | ARHGEF28 |
| ENSG00000056586 | 0.039562462 | 0.018237952 | -1.117188455 | 0.000452828 | 0.002821374 | RC3H2 |
| ENSG00000160075 | 0.055405337 | 0.025511974 | -1.118850443 | 6.63E-05 | 0.000557719 | SSU72 |
| ENSG00000127616 | 0.179395817 | 0.082589435 | -1.119117101 | 3.80E-06 | 4.54E-05 | SMARCA4 |
| ENSG00000075292 | 0.087179398 | 0.040085287 | -1.120914442 | 5.09E-11 | 1.61E-09 | ZNF638 |
| ENSG00000100239 | 0.042358314 | 0.019465426 | -1.12173126 | 2.19E-06 | 2.82E-05 | PPP6R2 |
| ENSG00000156787 | 0.075402572 | 0.034647079 | -1.121880015 | 8.04E-05 | 0.000659631 | TBC1D31 |
| ENSG00000198887 | 0.092925444 | 0.042683047 | -1.122410517 | 0.001321876 | 0.00684092 | SMC5 |
| ENSG00000100266 | 0.138705333 | 0.06370348 | -1.12257916 | 2.00E-14 | 1.05E-12 | PACSIN2 |
| ENSG00000171067 | 0.046285362 | 0.021257434 | -1.122588562 | 0.000210572 | 0.001489485 | C11orf24 |
| ENSG00000150990 | 0.075376931 | 0.034575483 | -1.124373651 | 0.000936456 | 0.005139609 | DHX37 |
| ENSG00000135541 | 0.081075764 | 0.037168455 | -1.125191999 | 1.34E-05 | 0.000138117 | AHI1 |
| ENSG00000176845 | 0.04510771 | 0.020663801 | -1.126268357 | 0.00157333 | 0.007896198 | METRNL |
| ENSG00000204516 | 0.018983228 | 0.008691882 | -1.126984754 | 0.001292933 | 0.006721797 | MICB |
| ENSG00000080986 | 0.139310241 | 0.063666477 | -1.129695469 | 0.001504784 | 0.007609347 | NDC80 |
| ENSG00000086102 | 0.047304636 | 0.021612591 | -1.130109514 | 1.75E-05 | 0.000173955 | NFX1 |
| ENSG00000140299 | 0.071745635 | 0.032758137 | -1.131037737 | 2.39E-06 | 3.05E-05 | BNIP2 |
| ENSG00000101335 | 0.075957774 | 0.034660138 | -1.131920215 | 8.60E-07 | 1.21E-05 | MYL9 |
| ENSG00000148158 | 0.059496181 | 0.02714246 | -1.132245579 | 0.001171934 | 0.006202688 | SNX30 |
| ENSG00000165209 | 0.059634531 | 0.027197135 | -1.132693265 | 0.000197905 | 0.001416551 | STRBP |
| ENSG00000092439 | 0.06337583 | 0.02888779 | -1.133472914 | 1.92E-05 | 0.000188392 | TRPM7 |
| ENSG00000115761 | 0.144382 | 0.065784385 | -1.13407382 | 5.26E-08 | 9.79E-07 | NOL10 |
| ENSG00000111961 | 0.09195935 | 0.041876699 | -1.134848537 | 0.000913149 | 0.005034672 | SASH1 |
| ENSG00000171862 | 0.018900614 | 0.008606655 | -1.134908592 | 0.000377061 | 0.002423385 | PTEN |
| ENSG00000138709 | 0.071560524 | 0.032582552 | -1.135064334 | 0.00036601 | 0.002362462 | LARP1B |
| ENSG00000137501 | 0.047306773 | 0.021534475 | -1.135398602 | 0.000150048 | 0.001123491 | SYTL2 |
| ENSG00000147140 | 0.040902977 | 0.018577845 | -1.138622702 | 0 | 0 | NONO |
| ENSG00000152492 | 0.040517395 | 0.018400869 | -1.138767501 | 4.98E-08 | 9.31E-07 | CCDC50 |
| ENSG00000197372 | 0.06797038 | 0.030864279 | -1.138968086 | 0.00177053 | 0.008714951 | ZNF675 |
| ENSG00000156232 | 0.059376314 | 0.026950176 | -1.139592872 | 0.000189579 | 0.001369183 | WHAMM |
| ENSG00000173715 | 0.095342949 | 0.043266355 | -1.139880662 | 0.000864204 | 0.00480891 | C11orf80 |
| ENSG00000140829 | 0.08783146 | 0.039760126 | -1.143415459 | 0.000216796 | 0.001529014 | DHX38 |
| ENSG00000101189 | 0.03283624 | 0.014839526 | -1.145843885 | 0.001020992 | 0.005520428 | MRGBP |
| ENSG00000176783 | 0.099725201 | 0.045053958 | -1.146304279 | 7.81E-06 | 8.52E-05 | RUFY1 |
| ENSG00000148660 | 0.040869036 | 0.018436876 | -1.148414029 | 0.000308986 | 0.002052795 | CAMK2G |
| ENSG00000121621 | 0.116268236 | 0.05241373 | -1.149440335 | 5.88E-06 | 6.63E-05 | KIF18A |
| ENSG00000172071 | 0.120555193 | 0.054256937 | -1.151814295 | 1.19E-05 | 0.000124891 | EIF2AK3 |
| ENSG00000104957 | 0.070742045 | 0.031828844 | -1.152233167 | 0.000545192 | 0.003312752 | CCDC130 |
| ENSG00000189042 | 0.393813946 | 0.177098883 | -1.15295909 | 0.000134598 | 0.001023097 | ZNF567 |
| ENSG00000144036 | 0.102343124 | 0.046002269 | -1.153637232 | 0.000417987 | 0.002644024 | EXOC6B |
| ENSG00000187742 | 0.031352601 | 0.014073102 | -1.15564475 | 0.000738397 | 0.004237157 | SECISBP2 |
| ENSG00000169371 | 0.141997699 | 0.06373235 | -1.155769798 | 0.001358312 | 0.006998159 | SNUPN |
| ENSG00000164823 | 0.02967756 | 0.013314536 | -1.15637036 | 0.000491626 | 0.003027381 | OSGIN2 |
| ENSG00000197694 | 0.158086037 | 0.070862357 | -1.15761859 | 0.000141657 | 0.001067975 | SPTAN1 |
| ENSG00000133114 | 0.093438923 | 0.041865666 | -1.158256074 | 3.68E-06 | 4.42E-05 | GPALPP1 |
| ENSG00000131013 | 0.126481766 | 0.056658207 | -1.158572547 | 1.74E-07 | 2.89E-06 | PPIL4 |
| ENSG00000103353 | 0.044333147 | 0.019827176 | -1.160906599 | 0.000132153 | 0.00100642 | UBFD1 |
| ENSG00000163605 | 0.137701159 | 0.061581415 | -1.160973789 | 0.000323819 | 0.002135419 | PPP4R2 |
| ENSG00000257923 | 0.083348929 | 0.037262661 | -1.161432955 | 0.000694967 | 0.004037187 | CUX1 |
| ENSG00000006652 | 0.064924642 | 0.028999029 | -1.162761551 | 0.001666521 | 0.00830498 | IFRD1 |
| ENSG00000104980 | 0.11672166 | 0.052120374 | -1.163152956 | 3.04E-07 | 4.79E-06 | TIMM44 |
| ENSG00000077044 | 0.048955434 | 0.021796149 | -1.167395749 | 0.001325459 | 0.006849438 | DGKD |
| ENSG00000134480 | 0.212892359 | 0.094697681 | -1.16872317 | 0.000130257 | 0.000996399 | CCNH |
| ENSG00000041802 | 0.103367121 | 0.045976474 | -1.168809634 | 3.99E-12 | 1.47E-10 | LSG1 |
| ENSG00000162852 | 0.085854412 | 0.03818421 | -1.168916108 | 0.00011778 | 0.000916108 | CNST |
| ENSG00000152133 | 0.137160903 | 0.060999977 | -1.168988705 | 0.000118468 | 0.000920267 | GPATCH11 |
| ENSG00000054523 | 0.081752612 | 0.036353561 | -1.169168132 | 0.001959447 | 0.009438096 | KIF1B |
| ENSG00000151532 | 0.07981322 | 0.035410015 | -1.172470266 | 3.65E-07 | 5.61E-06 | VTI1A |
| ENSG00000146007 | 0.177961795 | 0.078891104 | -1.173633027 | 7.92E-06 | 8.61E-05 | ZMAT2 |
| ENSG00000106443 | 0.129956505 | 0.05756001 | -1.174890105 | 1.28E-10 | 3.82E-09 | PHF14 |
| ENSG00000122483 | 0.104533824 | 0.046240015 | -1.176756057 | 0.000162718 | 0.001205901 | CCDC18 |
| ENSG00000089737 | 0.076917281 | 0.034022514 | -1.176818035 | 0 | 0 | DDX24 |
| ENSG00000179456 | 0.045114239 | 0.019949079 | -1.177260695 | 6.58E-05 | 0.000555488 | ZBTB18 |
| ENSG00000159217 | 0.083026079 | 0.036683705 | -1.178425193 | 0.000700161 | 0.004055603 | IGF2BP1 |
| ENSG00000137656 | 0.061601512 | 0.027186279 | -1.180087048 | 0.000588008 | 0.003524823 | BUD13 |
| ENSG00000196465 | 0.048188269 | 0.021261305 | -1.180451849 | 0.00150986 | 0.007628601 | MYL6B |
| ENSG00000116514 | 0.030459447 | 0.013414384 | -1.183108919 | 0.001321132 | 0.00684092 | RNF19B |
| ENSG00000185024 | 0.043021709 | 0.01894024 | -1.183610257 | 2.96E-05 | 0.000278777 | BRF1 |
| ENSG00000138593 | 0.033844941 | 0.01489235 | -1.184368818 | 0.000287855 | 0.001937024 | SECISBP2L |
| ENSG00000105176 | 0.220698279 | 0.097054869 | -1.185202879 | 0.001654365 | 0.00825338 | URI1 |
| ENSG00000070778 | 0.028100583 | 0.012357137 | -1.18525548 | 0.000771377 | 0.004394034 | PTPN21 |
| ENSG00000146457 | 0.042534611 | 0.018689158 | -1.186435702 | 0.000659449 | 0.003879585 | WTAP |
| ENSG00000116731 | 0.095039828 | 0.041525388 | -1.194538583 | 0.001147646 | 0.006100993 | PRDM2 |
| ENSG00000138386 | 0.019757979 | 0.008631712 | -1.194716735 | 0.00070984 | 0.00409982 | NAB1 |
| ENSG00000054282 | 0.147220643 | 0.064212767 | -1.197047898 | 0.000222789 | 0.001566692 | SDCCAG8 |
| ENSG00000129484 | 0.079538178 | 0.034689033 | -1.197167873 | 2.55E-05 | 0.000244074 | PARP2 |
| ENSG00000261609 | 0.192597234 | 0.083946199 | -1.198050072 | 0.000415398 | 0.002630414 | GAN |
| ENSG00000154222 | 0.044689237 | 0.01947534 | -1.198278925 | 9.24E-08 | 1.62E-06 | CC2D1B |
| ENSG00000163807 | 0.046040384 | 0.020008265 | -1.202303779 | 2.45E-07 | 3.90E-06 | KIAA1143 |
| ENSG00000113360 | 0.104586431 | 0.045431689 | -1.202924844 | 6.89E-06 | 7.61E-05 | DROSHA |
| ENSG00000111142 | 0.158759755 | 0.068954509 | -1.203128446 | 3.31E-05 | 0.000305164 | METAP2 |
| ENSG00000182195 | 0.200118133 | 0.086904961 | -1.203341459 | 1.92E-11 | 6.48E-10 | LDOC1 |
| ENSG00000130396 | 0.065509777 | 0.028424379 | -1.204581407 | 0.000817004 | 0.004603783 | AFDN |
| ENSG00000117569 | 0.101822581 | 0.044170914 | -1.204888945 | 3.13E-05 | 0.000291776 | PTBP2 |
| ENSG00000115216 | 0.116360136 | 0.050400792 | -1.207078576 | 0 | 0 | NRBP1 |
| ENSG00000151849 | 0.088678108 | 0.038362858 | -1.208867782 | 5.54E-06 | 6.29E-05 | CENPJ |
| ENSG00000127554 | 0.080359786 | 0.034734234 | -1.210115451 | 0.000506443 | 0.003105548 | GFER |
| ENSG00000148843 | 0.062312733 | 0.026887246 | -1.212605011 | 0.001054907 | 0.00568336 | PDCD11 |
| ENSG00000197312 | 0.057870922 | 0.024968166 | -1.2127488 | 0.001482637 | 0.007523531 | DDI2 |
| ENSG00000129250 | 0.049743145 | 0.021455248 | -1.213167124 | 3.97E-08 | 7.63E-07 | KIF1C |
| ENSG00000075213 | 0.076612017 | 0.033021271 | -1.214175064 | 5.51E-06 | 6.26E-05 | SEMA3A |
| ENSG00000079313 | 0.053733897 | 0.023138423 | -1.215541934 | 0.000859071 | 0.004787441 | REXO1 |
| ENSG00000154945 | 0.050238371 | 0.02160816 | -1.217213432 | 0.000333423 | 0.002186159 | ANKRD40 |
| ENSG00000156802 | 0.165040631 | 0.070939717 | -1.218155765 | 0 | 0 | ATAD2 |
| ENSG00000148229 | 0.078485645 | 0.033729445 | -1.218420216 | 0.000439242 | 0.002746687 | POLE3 |
| ENSG00000143514 | 0.083935197 | 0.036055207 | -1.219068282 | 0.000130813 | 0.000999231 | TP53BP2 |
| ENSG00000106462 | 0.070189739 | 0.030138354 | -1.219659532 | 2.42E-06 | 3.09E-05 | EZH2 |
| ENSG00000100614 | 0.071091041 | 0.030402811 | -1.22546305 | 5.29E-06 | 6.04E-05 | PPM1A |
| ENSG00000145901 | 0.059501619 | 0.02543714 | -1.225992474 | 0 | 0 | TNIP1 |
| ENSG00000163808 | 0.136712549 | 0.058422501 | -1.226549664 | 5.52E-12 | 1.96E-10 | KIF15 |
| ENSG00000198399 | 0.083446878 | 0.035619982 | -1.228171271 | 9.28E-06 | 9.96E-05 | ITSN2 |
| ENSG00000127511 | 0.053733692 | 0.02293602 | -1.228211915 | 9.36E-05 | 0.000750486 | SIN3B |
| ENSG00000165406 | 0.032372691 | 0.013809148 | -1.229152954 | 0.000643578 | 0.003797362 | 8-Mar |
| ENSG00000073792 | 0.055417858 | 0.023623746 | -1.230113222 | 1.93E-05 | 0.000189427 | IGF2BP2 |
| ENSG00000087074 | 0.067561896 | 0.028720274 | -1.234140322 | 0 | 0 | PPP1R15A |
| ENSG00000104177 | 0.064939371 | 0.027594197 | -1.234728509 | 0.00092862 | 0.005105925 | MYEF2 |
| ENSG00000071859 | 0.105065796 | 0.044560832 | -1.237445013 | 3.21E-05 | 0.000298384 | FAM50A |
| ENSG00000161526 | 0.106848658 | 0.045313125 | -1.237567903 | 9.64E-09 | 2.05E-07 | SAP30BP |
| ENSG00000143549 | 0.053150469 | 0.022536192 | -1.237838633 | 5.07E-08 | 9.45E-07 | TPM3 |
| ENSG00000116285 | 0.039832161 | 0.01688452 | -1.238232613 | 8.02E-08 | 1.42E-06 | ERRFI1 |
| ENSG00000166140 | 0.03613097 | 0.015313075 | -1.238472004 | 0.000939991 | 0.005156656 | ZFYVE19 |
| ENSG00000117226 | 0.064921525 | 0.027503642 | -1.239074229 | 0.000369357 | 0.002380236 | GBP3 |
| ENSG00000159086 | 0.132787125 | 0.056249061 | -1.239214356 | 5.75E-09 | 1.28E-07 | PAXBP1 |
| ENSG00000049246 | 0.065355843 | 0.027648677 | -1.241105762 | 1.21E-05 | 0.000126482 | PER3 |
| ENSG00000119596 | 0.091175153 | 0.038508744 | -1.24345466 | 0.000108501 | 0.000852205 | YLPM1 |
| ENSG00000153317 | 0.056947083 | 0.024045625 | -1.243847519 | 0.000184067 | 0.001337402 | ASAP1 |
| ENSG00000120647 | 0.175611336 | 0.07414124 | -1.244037834 | 1.09E-06 | 1.51E-05 | CCDC77 |
| ENSG00000135905 | 0.114859657 | 0.048371712 | -1.247636646 | 3.14E-06 | 3.85E-05 | DOCK10 |
| ENSG00000145675 | 0.0831389 | 0.034966241 | -1.24956096 | 0.00016032 | 0.001191502 | PIK3R1 |
| ENSG00000165650 | 0.069596586 | 0.029247825 | -1.250687183 | 9.26E-09 | 1.98E-07 | PDZD8 |
| ENSG00000004478 | 0.094739041 | 0.039809015 | -1.250863903 | 4.93E-09 | 1.11E-07 | FKBP4 |
| ENSG00000150991 | 0.08020202 | 0.033674553 | -1.251979784 | 1.36E-09 | 3.38E-08 | UBC |
| ENSG00000076650 | 0.189412228 | 0.079511007 | -1.252302965 | 7.39E-08 | 1.32E-06 | GPATCH1 |
| ENSG00000140319 | 0.068310868 | 0.02863908 | -1.254130009 | 0.001482098 | 0.007523531 | SRP14 |
| ENSG00000134697 | 0.097572069 | 0.040890952 | -1.254686568 | 8.00E-10 | 2.08E-08 | GNL2 |
| ENSG00000113649 | 0.096367621 | 0.040363715 | -1.255489545 | 0 | 0 | TCERG1 |
| ENSG00000071054 | 0.086159458 | 0.036087707 | -1.255501691 | 8.38E-07 | 1.19E-05 | MAP4K4 |
| ENSG00000109184 | 0.039973985 | 0.016714331 | -1.257975792 | 1.41E-05 | 0.000143804 | DCUN1D4 |
| ENSG00000163444 | 0.018567503 | 0.007762417 | -1.258201985 | 0.000719401 | 0.004141117 | TMEM183A |
| ENSG00000070814 | 0.07019101 | 0.029315199 | -1.259637397 | 0.000122975 | 0.00095037 | TCOF1 |
| ENSG00000159842 | 0.060359434 | 0.025204495 | -1.259898242 | 3.25E-05 | 0.000301189 | ABR |
| ENSG00000205726 | 0.084239111 | 0.03517162 | -1.260078428 | 7.41E-06 | 8.15E-05 | ITSN1 |
| ENSG00000174891 | 0.218242818 | 0.09103872 | -1.261382005 | 1.77E-09 | 4.30E-08 | RSRC1 |
| ENSG00000107672 | 0.127887308 | 0.053302677 | -1.2625932 | 0.000175197 | 0.001280694 | NSMCE4A |
| ENSG00000051341 | 0.045608825 | 0.019001865 | -1.263171945 | 0.000550548 | 0.003336866 | POLQ |
| ENSG00000164284 | 0.060279767 | 0.025112871 | -1.263246873 | 4.84E-06 | 5.58E-05 | GRPEL2 |
| ENSG00000070047 | 0.061869239 | 0.025755961 | -1.264315911 | 1.22E-05 | 0.000127417 | PHRF1 |
| ENSG00000078618 | 0.099771085 | 0.041530561 | -1.264448395 | 0 | 0 | NRDC |
| ENSG00000196914 | 0.056867755 | 0.023670514 | -1.2645198 | 4.79E-05 | 0.000419783 | ARHGEF12 |
| ENSG00000171681 | 0.167406621 | 0.069649703 | -1.265167488 | 1.13E-06 | 1.56E-05 | ATF7IP |
| ENSG00000110696 | 0.065368084 | 0.027195752 | -1.265205076 | 0.000840497 | 0.004703108 | C11orf58 |
| ENSG00000133858 | 0.05070997 | 0.021076325 | -1.266646124 | 3.86E-06 | 4.60E-05 | ZFC3H1 |
| ENSG00000180329 | 0.078723119 | 0.032698431 | -1.267565957 | 0.000713664 | 0.004119927 | CCDC43 |
| ENSG00000092201 | 0.100057339 | 0.041550709 | -1.267881984 | 0 | 0 | SUPT16H |
| ENSG00000116560 | 0.09969218 | 0.04138893 | -1.268235387 | 1.12E-14 | 6.07E-13 | SFPQ |
| ENSG00000089094 | 0.08289817 | 0.034397583 | -1.269033083 | 8.08E-05 | 0.000662039 | KDM2B |
| ENSG00000078403 | 0.095424853 | 0.039584525 | -1.269428509 | 5.70E-09 | 1.27E-07 | MLLT10 |
| ENSG00000116171 | 0.076778417 | 0.031845429 | -1.269614486 | 0.000318227 | 0.002103728 | SCP2 |
| ENSG00000103126 | 0.043067347 | 0.017836116 | -1.271792936 | 7.23E-06 | 7.96E-05 | AXIN1 |
| ENSG00000100888 | 0.093396217 | 0.03862307 | -1.273901256 | 0.001904709 | 0.00922066 | CHD8 |
| ENSG00000133028 | 0.076862352 | 0.031772248 | -1.27450995 | 4.40E-09 | 9.97E-08 | SCO1 |
| ENSG00000066136 | 0.091351327 | 0.037719693 | -1.276107735 | 2.00E-09 | 4.78E-08 | NFYC |
| ENSG00000127418 | 0.027055669 | 0.011138642 | -1.280357569 | 0.000171177 | 0.001257428 | FGFRL1 |
| ENSG00000196235 | 0.155798226 | 0.064137951 | -1.28042863 | 6.82E-10 | 1.80E-08 | SUPT5H |
| ENSG00000118482 | 0.069465457 | 0.028578404 | -1.281370389 | 0.000310162 | 0.002059468 | PHF3 |
| ENSG00000117360 | 0.089658523 | 0.036835294 | -1.28335197 | 3.54E-13 | 1.60E-11 | PRPF3 |
| ENSG00000075856 | 0.093691245 | 0.038481076 | -1.28376511 | 8.86E-07 | 1.25E-05 | SART3 |
| ENSG00000111885 | 0.107794422 | 0.044245066 | -1.284694045 | 0.00138454 | 0.00711804 | MAN1A1 |
| ENSG00000163565 | 0.103907388 | 0.042636341 | -1.285142701 | 1.44E-15 | 8.90E-14 | IFI16 |
| ENSG00000006453 | 0.134550355 | 0.055201714 | -1.285361229 | 0.000299835 | 0.001999815 | BAIAP2L1 |
| ENSG00000151327 | 0.258208979 | 0.105920385 | -1.2855589 | 0.001009637 | 0.005471331 | FAM177A1 |
| ENSG00000105401 | 0.115900949 | 0.047538026 | -1.285738482 | 3.45E-11 | 1.12E-09 | CDC37 |
| ENSG00000163161 | 0.080427887 | 0.03296143 | -1.286916994 | 4.94E-12 | 1.77E-10 | ERCC3 |
| ENSG00000104904 | 0.029198709 | 0.011964111 | -1.287191309 | 0.001167263 | 0.006182363 | OAZ1 |
| ENSG00000019995 | 0.199286329 | 0.081647712 | -1.287358377 | 4.04E-08 | 7.74E-07 | ZRANB1 |
| ENSG00000004534 | 0.096339236 | 0.039456997 | -1.287842308 | 1.89E-15 | 1.15E-13 | RBM6 |
| ENSG00000196642 | 0.083161792 | 0.034030255 | -1.289102883 | 4.74E-13 | 2.08E-11 | RABL6 |
| ENSG00000115539 | 0.110154878 | 0.04505999 | -1.289614496 | 0.000240987 | 0.001676039 | PDCL3 |
| ENSG00000125844 | 0.068948849 | 0.028191219 | -1.290280631 | 1.44E-08 | 2.99E-07 | RRBP1 |
| ENSG00000167671 | 0.090080823 | 0.036804128 | -1.291352426 | 7.53E-11 | 2.32E-09 | UBXN6 |
| ENSG00000064961 | 0.065729775 | 0.026850622 | -1.291591529 | 4.60E-06 | 5.35E-05 | HMG20B |
| ENSG00000143793 | 0.098788297 | 0.040341967 | -1.292058695 | 0.00071459 | 0.004123293 | C1orf35 |
| ENSG00000182979 | 0.041826579 | 0.017051887 | -1.294488615 | 0.001460699 | 0.007430166 | MTA1 |
| ENSG00000163029 | 0.105350076 | 0.042944506 | -1.294645889 | 1.11E-10 | 3.33E-09 | SMC6 |
| ENSG00000088325 | 0.08739468 | 0.03560653 | -1.295403603 | 0 | 0 | TPX2 |
| ENSG00000128829 | 0.076150948 | 0.030960649 | -1.298426277 | 6.79E-09 | 1.49E-07 | EIF2AK4 |
| ENSG00000092969 | 0.132871921 | 0.05399895 | -1.299033004 | 1.98E-07 | 3.22E-06 | TGFB2 |
| ENSG00000155158 | 0.082943144 | 0.033692238 | -1.29970646 | 0.000959437 | 0.00524658 | TTC39B |
| ENSG00000113645 | 0.050393068 | 0.020461021 | -1.300347157 | 1.27E-05 | 0.000131051 | WWC1 |
| ENSG00000168944 | 0.080470842 | 0.032646885 | -1.301520795 | 1.78E-09 | 4.30E-08 | CEP120 |
| ENSG00000155903 | 0.046370082 | 0.018800083 | -1.302455246 | 2.19E-05 | 0.000212139 | RASA2 |
| ENSG00000067900 | 0.06499466 | 0.026347107 | -1.302676634 | 1.08E-09 | 2.72E-08 | ROCK1 |
| ENSG00000103550 | 0.106582996 | 0.043027629 | -1.308642034 | 2.19E-05 | 0.000212247 | KNOP1 |
| ENSG00000198742 | 0.039446263 | 0.015919643 | -1.309080646 | 0.001859749 | 0.009057708 | SMURF1 |
| ENSG00000112787 | 0.016795783 | 0.006773688 | -1.310085689 | 0.00101533 | 0.005492283 | FBRSL1 |
| ENSG00000151718 | 0.090417432 | 0.036412553 | -1.312165051 | 1.29E-05 | 0.000132805 | WWC2 |
| ENSG00000122034 | 0.032667387 | 0.013154781 | -1.312263891 | 0.00046206 | 0.002871458 | GTF3A |
| ENSG00000196141 | 0.061187441 | 0.024633877 | -1.312591829 | 0 | 0 | SPATS2L |
| ENSG00000182606 | 0.051895938 | 0.020885481 | -1.313121206 | 0.000105471 | 0.000831122 | TRAK1 |
| ENSG00000151304 | 0.185024535 | 0.074353613 | -1.315241847 | 4.08E-07 | 6.20E-06 | SRFBP1 |
| ENSG00000099875 | 0.033186685 | 0.013331637 | -1.315750544 | 0.000261115 | 0.001791164 | MKNK2 |
| ENSG00000005302 | 0.045714519 | 0.018331702 | -1.318311725 | 0.000640515 | 0.003784864 | MSL3 |
| ENSG00000123240 | 0.069500752 | 0.02783317 | -1.320223383 | 8.56E-10 | 2.21E-08 | OPTN |
| ENSG00000169641 | 0.077693014 | 0.031092366 | -1.321224493 | 0.000324337 | 0.00213707 | LUZP1 |
| ENSG00000122335 | 0.080890376 | 0.032335891 | -1.322831705 | 4.25E-05 | 0.000378945 | SERAC1 |
| ENSG00000141580 | 0.034839102 | 0.013922063 | -1.323334365 | 0 | 0 | WDR45B |
| ENSG00000136811 | 0.091622632 | 0.036602823 | -1.323749092 | 7.54E-10 | 1.97E-08 | ODF2 |
| ENSG00000105127 | 0.093434732 | 0.037280392 | -1.325541895 | 2.57E-09 | 6.07E-08 | AKAP8 |
| ENSG00000104332 | 0.055750572 | 0.022244324 | -1.325549355 | 0.000111415 | 0.000872245 | SFRP1 |
| ENSG00000072609 | 0.047496554 | 0.018941011 | -1.326309474 | 1.62E-06 | 2.15E-05 | CHFR |
| ENSG00000108669 | 0.04880394 | 0.019456116 | -1.326773903 | 3.55E-12 | 1.33E-10 | CYTH1 |
| ENSG00000119772 | 0.053704159 | 0.021389251 | -1.32814784 | 0.001111148 | 0.005944889 | DNMT3A |
| ENSG00000171530 | 0.140095376 | 0.055776783 | -1.32867272 | 0.000653278 | 0.003848931 | TBCA |
| ENSG00000171132 | 0.095376557 | 0.037909411 | -1.331078656 | 0.001474473 | 0.007493889 | PRKCE |
| ENSG00000167491 | 0.062784229 | 0.02495057 | -1.331329411 | 5.22E-09 | 1.17E-07 | GATAD2A |
| ENSG00000107960 | 0.051547425 | 0.020479316 | -1.331732841 | 0.001927688 | 0.009313157 | STN1 |
| ENSG00000122966 | 0.112313862 | 0.044617255 | -1.331862354 | 0.000744358 | 0.004262333 | CIT |
| ENSG00000184708 | 0.106807409 | 0.042345171 | -1.334742378 | 8.17E-07 | 1.16E-05 | EIF4ENIF1 |
| ENSG00000183431 | 0.127442802 | 0.050493908 | -1.335668649 | 1.71E-13 | 7.90E-12 | SF3A3 |
| ENSG00000044459 | 0.10312407 | 0.040856865 | -1.335730702 | 1.33E-06 | 1.79E-05 | CNTLN |
| ENSG00000170638 | 0.052924658 | 0.020950164 | -1.336978519 | 0.000297005 | 0.001984158 | TRABD |
| ENSG00000171467 | 0.044279227 | 0.017523183 | -1.337365148 | 0.000304875 | 0.002028842 | ZNF318 |
| ENSG00000065883 | 0.079434665 | 0.031389552 | -1.339484286 | 2.62E-06 | 3.28E-05 | CDK13 |
| ENSG00000048342 | 0.077673797 | 0.030683057 | -1.339985789 | 0.00189464 | 0.00918301 | CC2D2A |
| ENSG00000103995 | 0.182521759 | 0.072073964 | -1.34051836 | 4.51E-10 | 1.23E-08 | CEP152 |
| ENSG00000164031 | 0.058412424 | 0.023053947 | -1.341261478 | 3.00E-08 | 5.90E-07 | DNAJB14 |
| ENSG00000159128 | 0.022141723 | 0.008708659 | -1.346245084 | 0.001268123 | 0.006613811 | IFNGR2 |
| ENSG00000117724 | 0.126677355 | 0.049779405 | -1.34753776 | 4.68E-08 | 8.81E-07 | CENPF |
| ENSG00000131051 | 0.075471946 | 0.029654189 | -1.34770458 | 4.18E-12 | 1.53E-10 | RBM39 |
| ENSG00000133069 | 0.073753723 | 0.028964192 | -1.348445437 | 0.001025969 | 0.005542354 | TMCC2 |
| ENSG00000080608 | 0.133035498 | 0.052159939 | -1.350797172 | 2.22E-05 | 0.000214989 | PUM3 |
| ENSG00000122545 | 0.126327835 | 0.049521476 | -1.351046323 | 2.26E-10 | 6.47E-09 | 7-Sep |
| ENSG00000095066 | 0.065029062 | 0.025477228 | -1.351876289 | 0.00011926 | 0.000925226 | HOOK2 |
| ENSG00000196476 | 0.12745591 | 0.049904049 | -1.352769476 | 4.51E-05 | 0.000397622 | C20orf96 |
| ENSG00000138778 | 0.107189463 | 0.041952057 | -1.353349637 | 5.32E-11 | 1.66E-09 | CENPE |
| ENSG00000133422 | 0.099065426 | 0.038760535 | -1.353793171 | 0.001054802 | 0.00568336 | MORC2 |
| ENSG00000180992 | 0.065335591 | 0.025479804 | -1.358514899 | 0.000827196 | 0.004643804 | MRPL14 |
| ENSG00000154240 | 0.19687864 | 0.076693472 | -1.360130906 | 4.89E-08 | 9.18E-07 | CEP112 |
| ENSG00000203875 | 1.080852332 | 0.421013092 | -1.36023243 | 0.00014044 | 0.001061462 | SNHG5 |
| ENSG00000123104 | 0.094024623 | 0.036616661 | -1.360538353 | 0.001962 | 0.009444775 | ITPR2 |
| ENSG00000177030 | 0.019360521 | 0.007537262 | -1.361005422 | 0.000201947 | 0.001438635 | DEAF1 |
| ENSG00000164402 | 0.031234126 | 0.012148761 | -1.362313992 | 2.22E-10 | 6.40E-09 | 8-Sep |
| ENSG00000148842 | 0.068276412 | 0.026543917 | -1.363005972 | 0.001220227 | 0.006404727 | CNNM2 |
| ENSG00000164934 | 0.204849504 | 0.079542405 | -1.364768316 | 7.81E-05 | 0.000643646 | DCAF13 |
| ENSG00000105298 | 0.061181905 | 0.023748844 | -1.365247755 | 0.000155172 | 0.001156819 | CACTIN |
| ENSG00000163939 | 0.082255816 | 0.031901999 | -1.366470839 | 2.35E-06 | 3.01E-05 | PBRM1 |
| ENSG00000101745 | 0.105492551 | 0.040834466 | -1.369281854 | 1.71E-07 | 2.84E-06 | ANKRD12 |
| ENSG00000143344 | 0.06012968 | 0.023264214 | -1.369964829 | 0.000415323 | 0.002630414 | RGL1 |
| ENSG00000167674 | 0.122291415 | 0.047295122 | -1.370559842 | 1.23E-11 | 4.21E-10 | HDGFL2 |
| ENSG00000117597 | 0.109850122 | 0.042456241 | -1.371487934 | 2.79E-09 | 6.56E-08 | UTP25 |
| ENSG00000107404 | 0.028638598 | 0.011051671 | -1.373696401 | 4.90E-10 | 1.32E-08 | DVL1 |
| ENSG00000104714 | 0.114894257 | 0.044322874 | -1.374183361 | 0.000161935 | 0.001202021 | ERICH1 |
| ENSG00000196182 | 0.028825222 | 0.011117997 | -1.374434811 | 0.00053144 | 0.003235732 | STK40 |
| ENSG00000011275 | 0.074046706 | 0.028548564 | -1.375017376 | 7.27E-10 | 1.91E-08 | RNF216 |
| ENSG00000066697 | 0.044625175 | 0.017202631 | -1.37522859 | 0.000160471 | 0.001191885 | MSANTD3 |
| ENSG00000065000 | 0.096583727 | 0.037231208 | -1.375267724 | 6.88E-08 | 1.24E-06 | AP3D1 |
| ENSG00000100764 | 0.039100602 | 0.015072483 | -1.375273713 | 2.01E-07 | 3.26E-06 | PSMC1 |
| ENSG00000051825 | 0.066970899 | 0.025813407 | -1.375413781 | 1.64E-05 | 0.000163913 | MPHOSPH9 |
| ENSG00000141447 | 0.08642212 | 0.033267033 | -1.377307401 | 4.29E-08 | 8.17E-07 | OSBPL1A |
| ENSG00000140990 | 0.067601843 | 0.026014596 | -1.377741278 | 2.40E-05 | 0.000230512 | NDUFB10 |
| ENSG00000116809 | 0.062645912 | 0.024070483 | -1.379955243 | 2.25E-07 | 3.61E-06 | ZBTB17 |
| ENSG00000084731 | 0.082069801 | 0.031470335 | -1.382858948 | 0.000838365 | 0.004694613 | KIF3C |
| ENSG00000164877 | 0.029059247 | 0.011136989 | -1.383638036 | 0.000396805 | 0.002525985 | MICALL2 |
| ENSG00000184205 | 0.132025746 | 0.050593082 | -1.383807259 | 1.18E-08 | 2.48E-07 | TSPYL2 |
| ENSG00000132680 | 0.045207675 | 0.017315114 | -1.384535842 | 1.94E-09 | 4.66E-08 | KHDC4 |
| ENSG00000133302 | 0.033493376 | 0.012825843 | -1.384822137 | 0.000662795 | 0.003892906 | SLF1 |
| ENSG00000125651 | 0.131516454 | 0.05035387 | -1.385068742 | 0 | 0 | GTF2F1 |
| ENSG00000179981 | 0.04588537 | 0.017558863 | -1.385834846 | 0.001290063 | 0.006709776 | TSHZ1 |
| ENSG00000165494 | 0.087109671 | 0.033329263 | -1.386043482 | 6.72E-07 | 9.75E-06 | PCF11 |
| ENSG00000143971 | 0.035221267 | 0.013466699 | -1.387050522 | 0.00071721 | 0.004136428 | ETAA1 |
| ENSG00000171988 | 0.086150859 | 0.032889357 | -1.389244362 | 9.33E-06 | 1.00E-04 | JMJD1C |
| ENSG00000139433 | 0.021191335 | 0.008067894 | -1.393210459 | 0.000115597 | 0.000901465 | GLTP |
| ENSG00000011566 | 0.050711126 | 0.019300919 | -1.393632771 | 1.05E-07 | 1.81E-06 | MAP4K3 |
| ENSG00000166598 | 0.257637952 | 0.097993046 | -1.394593861 | 5.01E-08 | 9.36E-07 | HSP90B1 |
| ENSG00000141298 | 0.070218196 | 0.026644638 | -1.397999711 | 0.000293348 | 0.001964091 | SSH2 |
| ENSG00000020922 | 0.071373412 | 0.027031503 | -1.400745039 | 5.68E-09 | 1.27E-07 | MRE11 |
| ENSG00000136997 | 0.161072941 | 0.060975635 | -1.40140936 | 3.06E-11 | 1.01E-09 | MYC |
| ENSG00000112701 | 0.11136579 | 0.042151059 | -1.401665351 | 5.00E-13 | 2.17E-11 | SENP6 |
| ENSG00000185453 | 0.030004437 | 0.011352426 | -1.402175274 | 0.001186765 | 0.00626189 | ZSWIM9 |
| ENSG00000106344 | 0.109559147 | 0.041438383 | -1.402670326 | 4.40E-08 | 8.37E-07 | RBM28 |
| ENSG00000109920 | 0.054945169 | 0.020777879 | -1.402944224 | 1.33E-05 | 0.000137282 | FNBP4 |
| ENSG00000167258 | 0.081036144 | 0.030620541 | -1.404065754 | 0.001546331 | 0.007783434 | CDK12 |
| ENSG00000163166 | 0.243157892 | 0.091843526 | -1.404643474 | 7.77E-13 | 3.24E-11 | IWS1 |
| ENSG00000149136 | 0.099696749 | 0.037639955 | -1.405281563 | 2.22E-16 | 1.49E-14 | SSRP1 |
| ENSG00000099814 | 0.025326777 | 0.009549552 | -1.407158529 | 0.000462973 | 0.002875646 | CEP170B |
| ENSG00000011454 | 0.145229623 | 0.054715236 | -1.408321221 | 7.65E-07 | 1.10E-05 | RABGAP1 |
| ENSG00000125633 | 0.121087743 | 0.045600487 | -1.408931696 | 2.96E-07 | 4.67E-06 | CCDC93 |
| ENSG00000187164 | 0.068113234 | 0.025628168 | -1.410204755 | 3.09E-09 | 7.22E-08 | SHTN1 |
| ENSG00000152433 | 0.119833895 | 0.045067979 | -1.410861388 | 0.002023905 | 0.009700061 | ZNF547 |
| ENSG00000148337 | 0.047185741 | 0.017742936 | -1.411106166 | 5.90E-09 | 1.31E-07 | CIZ1 |
| ENSG00000101004 | 0.105094564 | 0.039496523 | -1.411890497 | 0.000142741 | 0.00107548 | NINL |
| ENSG00000111707 | 0.075648784 | 0.028428167 | -1.411995787 | 3.03E-10 | 8.54E-09 | SUDS3 |
| ENSG00000133030 | 0.059241517 | 0.022212482 | -1.415238001 | 3.50E-06 | 4.23E-05 | MPRIP |
| ENSG00000038219 | 0.17713385 | 0.06628458 | -1.418094739 | 0.001116544 | 0.005967306 | BOD1L1 |
| ENSG00000153922 | 0.097310708 | 0.036397839 | -1.418745753 | 1.42E-07 | 2.40E-06 | CHD1 |
| ENSG00000187239 | 0.064019443 | 0.023912615 | -1.420738248 | 3.19E-07 | 4.97E-06 | FNBP1 |
| ENSG00000115306 | 0.116220215 | 0.043397918 | -1.421163279 | 0.000569407 | 0.003430422 | SPTBN1 |
| ENSG00000182670 | 0.02666001 | 0.009953578 | -1.421390256 | 5.05E-05 | 0.000439312 | TTC3 |
| ENSG00000137710 | 0.059364884 | 0.022127501 | -1.423769263 | 0 | 0 | RDX |
| ENSG00000169607 | 0.072531165 | 0.027017806 | -1.42469051 | 1.22E-15 | 7.61E-14 | CKAP2L |
| ENSG00000108055 | 0.143308552 | 0.053360591 | -1.425278167 | 3.13E-12 | 1.19E-10 | SMC3 |
| ENSG00000258890 | 0.104879479 | 0.038989915 | -1.427559508 | 6.11E-06 | 6.87E-05 | CEP95 |
| ENSG00000035928 | 0.068473311 | 0.025448729 | -1.427948166 | 0 | 0 | RFC1 |
| ENSG00000173281 | 0.078840385 | 0.029250125 | -1.430492038 | 0.000196576 | 0.001409558 | PPP1R3B |
| ENSG00000108592 | 0.135827927 | 0.050352793 | -1.431636423 | 0 | 0 | FTSJ3 |
| ENSG00000135387 | 0.086010386 | 0.031874944 | -1.432088078 | 0 | 0 | CAPRIN1 |
| ENSG00000087206 | 0.116978962 | 0.043351187 | -1.432105683 | 5.64E-11 | 1.76E-09 | UIMC1 |
| ENSG00000134318 | 0.106487673 | 0.039459612 | -1.432237757 | 1.86E-12 | 7.36E-11 | ROCK2 |
| ENSG00000148339 | 0.061235964 | 0.022683885 | -1.432711469 | 0.000326233 | 0.002147209 | SLC25A25 |
| ENSG00000143740 | 0.05730035 | 0.021214784 | -1.433473931 | 5.00E-05 | 0.000435309 | SNAP47 |
| ENSG00000065882 | 0.038227185 | 0.014152577 | -1.433534206 | 4.24E-09 | 9.64E-08 | TBC1D1 |
| ENSG00000092531 | 0.178321152 | 0.066017573 | -1.43355584 | 5.28E-05 | 0.000455853 | SNAP23 |
| ENSG00000134453 | 0.084958289 | 0.031421365 | -1.435008876 | 1.21E-08 | 2.54E-07 | RBM17 |
| ENSG00000166025 | 0.082123819 | 0.030358695 | -1.435690946 | 0.000292927 | 0.001962369 | AMOTL1 |
| ENSG00000100345 | 0.13567537 | 0.050148254 | -1.435887453 | 1.12E-05 | 0.000118657 | MYH9 |
| ENSG00000204574 | 0.086806253 | 0.032019359 | -1.438854549 | 7.42E-12 | 2.60E-10 | ABCF1 |
| ENSG00000196323 | 0.038442669 | 0.014170692 | -1.439798245 | 5.32E-08 | 9.87E-07 | ZBTB44 |
| ENSG00000084112 | 0.028103764 | 0.010355772 | -1.440328311 | 0.000670311 | 0.003922378 | SSH1 |
| ENSG00000174243 | 0.104926528 | 0.038653176 | -1.44072059 | 0 | 0 | DDX23 |
| ENSG00000103942 | 0.158045173 | 0.058173663 | -1.441898935 | 0.000815137 | 0.004595418 | HOMER2 |
| ENSG00000100722 | 0.135609269 | 0.049804422 | -1.445110033 | 5.60E-07 | 8.28E-06 | ZC3H14 |
| ENSG00000135365 | 0.06176969 | 0.022648085 | -1.447510041 | 0.000378179 | 0.002428141 | PHF21A |
| ENSG00000093167 | 0.087067447 | 0.031886359 | -1.449194022 | 1.60E-09 | 3.95E-08 | LRRFIP2 |
| ENSG00000134982 | 0.076315046 | 0.027934712 | -1.449908568 | 3.46E-07 | 5.37E-06 | APC |
| ENSG00000065809 | 0.095615306 | 0.034978627 | -1.450767929 | 1.78E-08 | 3.64E-07 | FAM107B |
| ENSG00000127328 | 0.04129871 | 0.015106458 | -1.450931262 | 0.000573971 | 0.003452728 | RAB3IP |
| ENSG00000144824 | 0.088420768 | 0.032315888 | -1.452141626 | 1.67E-15 | 1.02E-13 | PHLDB2 |
| ENSG00000047644 | 0.043839504 | 0.016011924 | -1.45308477 | 0.000354942 | 0.002299667 | WWC3 |
| ENSG00000125354 | 0.105562032 | 0.03852278 | -1.454307319 | 1.85E-07 | 3.04E-06 | 6-Sep |
| ENSG00000186350 | 0.033755809 | 0.012306704 | -1.455691386 | 3.16E-06 | 3.87E-05 | RXRA |
| ENSG00000182150 | 0.046028737 | 0.016771642 | -1.456510902 | 0.001637769 | 0.008182042 | ERCC6L2 |
| ENSG00000185697 | 0.052382326 | 0.019076373 | -1.457293235 | 0 | 0 | MYBL1 |
| ENSG00000143379 | 0.12665438 | 0.046113267 | -1.457643184 | 0.000684943 | 0.003990525 | SETDB1 |
| ENSG00000099940 | 0.069116959 | 0.02516081 | -1.457861397 | 4.70E-10 | 1.27E-08 | SNAP29 |
| ENSG00000136940 | 0.116853753 | 0.042516613 | -1.458605489 | 1.46E-06 | 1.94E-05 | PDCL |
| ENSG00000225190 | 0.018426605 | 0.006700975 | -1.459347249 | 0.001654794 | 0.00825338 | PLEKHM1 |
| ENSG00000163872 | 0.063501334 | 0.023080389 | -1.460119349 | 0.000426511 | 0.002685235 | YEATS2 |
| ENSG00000102763 | 0.082875872 | 0.030095791 | -1.461390409 | 0.000988305 | 0.005367824 | VWA8 |
| ENSG00000140386 | 0.132477577 | 0.04795806 | -1.465902974 | 0.000282251 | 0.001905707 | SCAPER |
| ENSG00000184640 | 0.066419379 | 0.024012256 | -1.46783331 | 0 | 0 | 9-Sep |
| ENSG00000125843 | 0.027383587 | 0.009882313 | -1.470390807 | 0.001241317 | 0.006504067 | AP5S1 |
| ENSG00000029153 | 0.019385547 | 0.0069869 | -1.472257053 | 0.000234737 | 0.001637301 | ARNTL2 |
| ENSG00000175470 | 0.106290901 | 0.038304823 | -1.472420155 | 8.10E-15 | 4.49E-13 | PPP2R2D |
| ENSG00000080503 | 0.205396139 | 0.07401541 | -1.472511491 | 4.44E-16 | 2.89E-14 | SMARCA2 |
| ENSG00000116717 | 0.076396258 | 0.027497299 | -1.474212074 | 7.65E-06 | 8.36E-05 | GADD45A |
| ENSG00000113758 | 0.048123945 | 0.017317683 | -1.474509016 | 1.42E-11 | 4.84E-10 | DBN1 |
| ENSG00000245937 | 0.07182967 | 0.025837485 | -1.475114234 | 0.000270029 | 0.001840758 | LINC01184 |
| ENSG00000077458 | 0.051855218 | 0.018650027 | -1.475311412 | 0.00182868 | 0.008946348 | FAM76B |
| ENSG00000091986 | 0.096939247 | 0.034864401 | -1.475326193 | 3.13E-10 | 8.78E-09 | CCDC80 |
| ENSG00000112130 | 0.121882828 | 0.043774409 | -1.477335255 | 2.83E-05 | 0.000267855 | RNF8 |
| ENSG00000007392 | 0.110848197 | 0.039676911 | -1.482213684 | 4.41E-05 | 0.000391022 | LUC7L |
| ENSG00000106665 | 0.05406434 | 0.0193508 | -1.482284109 | 7.81E-05 | 0.000643646 | CLIP2 |
| ENSG00000132424 | 0.088125382 | 0.031453997 | -1.486314227 | 0 | 0 | PNISR |
| ENSG00000149231 | 0.086097112 | 0.030702404 | -1.487613221 | 8.39E-14 | 4.01E-12 | CCDC82 |
| ENSG00000168439 | 0.08595493 | 0.030636984 | -1.488306126 | 0 | 0 | STIP1 |
| ENSG00000165669 | 0.085660242 | 0.03042992 | -1.493135221 | 0.000259877 | 0.001784703 | FAM204A |
| ENSG00000162244 | 0.006206773 | 0.002202278 | -1.494846856 | 0.000342539 | 0.00223375 | RPL29 |
| ENSG00000133026 | 0.135695278 | 0.048079767 | -1.496868708 | 3.41E-05 | 0.000313043 | MYH10 |
| ENSG00000181751 | 0.035221098 | 0.012478337 | -1.497014233 | 0.001431184 | 0.007310701 | C5orf30 |
| ENSG00000162869 | 0.141282932 | 0.049961481 | -1.49969904 | 1.29E-06 | 1.75E-05 | PPP1R21 |
| ENSG00000204120 | 0.123150097 | 0.043509419 | -1.501018106 | 1.93E-07 | 3.15E-06 | GIGYF2 |
| ENSG00000166024 | 0.03927845 | 0.013870677 | -1.50169985 | 0.000268725 | 0.00183499 | R3HCC1L |
| ENSG00000138592 | 0.124640806 | 0.043941215 | -1.504129816 | 3.65E-05 | 0.000331708 | USP8 |
| ENSG00000108799 | 0.054600898 | 0.019233834 | -1.505278304 | 8.18E-05 | 0.000669417 | EZH1 |
| ENSG00000111450 | 0.098858538 | 0.034786057 | -1.50685642 | 0 | 0 | STX2 |
| ENSG00000116754 | 0.121725948 | 0.042829146 | -1.506971913 | 0 | 0 | SRSF11 |
| ENSG00000132740 | 0.043834781 | 0.015415684 | -1.507677171 | 0.00019443 | 0.001398481 | IGHMBP2 |
| ENSG00000065548 | 0.092296014 | 0.032452491 | -1.50793913 | 1.46E-06 | 1.94E-05 | ZC3H15 |
| ENSG00000107581 | 0.268839408 | 0.094460185 | -1.508966363 | 0 | 0 | EIF3A |
| ENSG00000100815 | 0.153549228 | 0.053869456 | -1.511161857 | 3.04E-10 | 8.55E-09 | TRIP11 |
| ENSG00000116161 | 0.047874581 | 0.016793909 | -1.511321779 | 3.97E-05 | 0.000356708 | CACYBP |
| ENSG00000105705 | 0.059702575 | 0.020929461 | -1.512257986 | 9.20E-07 | 1.29E-05 | SUGP1 |
| ENSG00000112511 | 0.030029225 | 0.010519913 | -1.513244407 | 1.54E-08 | 3.18E-07 | PHF1 |
| ENSG00000116539 | 0.068365744 | 0.023936269 | -1.514075316 | 0.00125096 | 0.006534896 | ASH1L |
| ENSG00000166123 | 0.038392732 | 0.013440762 | -1.514218327 | 3.16E-06 | 3.87E-05 | GPT2 |
| ENSG00000163681 | 0.065478869 | 0.022921855 | -1.514305576 | 0.001119777 | 0.005979269 | SLMAP |
| ENSG00000072518 | 0.063520531 | 0.022216842 | -1.51556924 | 6.65E-05 | 0.000558281 | MARK2 |
| ENSG00000140575 | 0.085505529 | 0.029905632 | -1.515600521 | 1.80E-10 | 5.27E-09 | IQGAP1 |
| ENSG00000139437 | 0.139839093 | 0.048845099 | -1.517482009 | 1.98E-06 | 2.57E-05 | TCHP |
| ENSG00000062194 | 0.101793301 | 0.035475189 | -1.52076035 | 6.18E-11 | 1.91E-09 | GPBP1 |
| ENSG00000109072 | 0.569418332 | 0.198416151 | -1.520959478 | 0.000794664 | 0.004503219 | VTN |
| ENSG00000164463 | 0.159759092 | 0.05566361 | -1.52109166 | 0.000471901 | 0.002919037 | CREBRF |
| ENSG00000228409 | 0.157132645 | 0.0547307 | -1.521560734 | 0.001837498 | 0.008978429 | CCT6P1 |
| ENSG00000232593 | 0.082383039 | 0.028666414 | -1.522985894 | 0.000423017 | 0.002672025 | KANTR |
| ENSG00000080824 | 0.117152393 | 0.040640478 | -1.527397131 | 8.47E-13 | 3.50E-11 | HSP90AA1 |
| ENSG00000167670 | 0.117795707 | 0.040801217 | -1.529602886 | 0 | 0 | CHAF1A |
| ENSG00000011243 | 0.118723226 | 0.041109557 | -1.530056488 | 2.17E-08 | 4.36E-07 | AKAP8L |
| ENSG00000055609 | 0.079412515 | 0.027495712 | -1.530159745 | 0.000371321 | 0.002390332 | KMT2C |
| ENSG00000084093 | 0.102525285 | 0.035480716 | -1.530872735 | 0 | 0 | REST |
| ENSG00000135870 | 0.08916098 | 0.030848215 | -1.531225477 | 6.51E-09 | 1.43E-07 | RC3H1 |
| ENSG00000106346 | 0.042687631 | 0.014761723 | -1.53195692 | 0.000345685 | 0.002250602 | USP42 |
| ENSG00000131351 | 0.078175661 | 0.027008807 | -1.533289588 | 7.42E-06 | 8.15E-05 | HAUS8 |
| ENSG00000083937 | 0.120690617 | 0.041538412 | -1.538795533 | 8.52E-06 | 9.22E-05 | CHMP2B |
| ENSG00000166900 | 0.081893076 | 0.028178043 | -1.539170068 | 0 | 0 | STX3 |
| ENSG00000119685 | 0.088998421 | 0.030593324 | -1.540562866 | 0.001487241 | 0.007534532 | TTLL5 |
| ENSG00000079134 | 0.096943636 | 0.033295335 | -1.541826113 | 2.58E-07 | 4.09E-06 | THOC1 |
| ENSG00000115816 | 0.196597417 | 0.067505829 | -1.542160382 | 2.13E-08 | 4.28E-07 | CEBPZ |
| ENSG00000131711 | 0.279132961 | 0.095842237 | -1.54221901 | 1.04E-07 | 1.80E-06 | MAP1B |
| ENSG00000126581 | 0.133842824 | 0.045869237 | -1.544940976 | 2.30E-12 | 8.97E-11 | BECN1 |
| ENSG00000162385 | 0.179680362 | 0.061548064 | -1.545647348 | 0.000557704 | 0.003371739 | MAGOH |
| ENSG00000151276 | 0.084362631 | 0.028894722 | -1.54579811 | 0.000501904 | 0.003080855 | MAGI1 |
| ENSG00000142599 | 0.071942285 | 0.024639857 | -1.545846071 | 1.21E-05 | 0.000126221 | RERE |
| ENSG00000156983 | 0.066282551 | 0.022637507 | -1.549914013 | 0.000135204 | 0.001027055 | BRPF1 |
| ENSG00000272886 | 0.047087779 | 0.016063946 | -1.551526323 | 0.000105754 | 0.000832262 | DCP1A |
| ENSG00000130695 | 0.070556309 | 0.024060174 | -1.55212804 | 0.000280864 | 0.001899543 | CEP85 |
| ENSG00000160551 | 0.074030573 | 0.025228347 | -1.553075529 | 5.25E-05 | 0.000453859 | TAOK1 |
| ENSG00000169062 | 0.027202173 | 0.009267073 | -1.553536225 | 3.31E-07 | 5.16E-06 | UPF3A |
| ENSG00000125817 | 0.053741646 | 0.018255407 | -1.557716664 | 0 | 0 | CENPB |
| ENSG00000104129 | 0.062446531 | 0.02120603 | -1.55814689 | 0.000526475 | 0.003209278 | DNAJC17 |
| ENSG00000084652 | 0.104380177 | 0.035443507 | -1.558254494 | 1.37E-14 | 7.27E-13 | TXLNA |
| ENSG00000141577 | 0.049658056 | 0.016842988 | -1.559879678 | 1.93E-11 | 6.51E-10 | CEP131 |
| ENSG00000147526 | 0.088821339 | 0.030090441 | -1.561601073 | 6.72E-09 | 1.47E-07 | TACC1 |
| ENSG00000105058 | 0.085580549 | 0.028976126 | -1.562418226 | 2.15E-06 | 2.77E-05 | FAM32A |
| ENSG00000077380 | 0.135440934 | 0.04585705 | -1.562448366 | 6.03E-14 | 2.93E-12 | DYNC1I2 |
| ENSG00000198732 | 0.237542297 | 0.080340449 | -1.563985985 | 0.000867188 | 0.004823283 | SMOC1 |
| ENSG00000122965 | 0.119320057 | 0.040353467 | -1.564072023 | 1.14E-08 | 2.40E-07 | RBM19 |
| ENSG00000118900 | 0.054828054 | 0.018534797 | -1.564677997 | 2.61E-05 | 0.000249383 | UBN1 |
| ENSG00000046651 | 0.07665787 | 0.025892225 | -1.565914986 | 4.30E-05 | 0.000382598 | OFD1 |
| ENSG00000130816 | 0.118363147 | 0.039938936 | -1.567352169 | 1.97E-10 | 5.72E-09 | DNMT1 |
| ENSG00000198087 | 0.118477729 | 0.039961552 | -1.567931378 | 0 | 0 | CD2AP |
| ENSG00000140743 | 0.183519626 | 0.06188277 | -1.568324672 | 1.11E-16 | 7.67E-15 | CDR2 |
| ENSG00000058091 | 0.039879038 | 0.013446834 | -1.568364122 | 5.89E-05 | 0.000503688 | CDK14 |
| ENSG00000126870 | 0.086037674 | 0.028992655 | -1.569281072 | 2.25E-08 | 4.52E-07 | WDR60 |
| ENSG00000061936 | 0.106368112 | 0.0358383 | -1.569491598 | 6.85E-13 | 2.88E-11 | SFSWAP |
| ENSG00000109466 | 0.046191968 | 0.015527644 | -1.572803049 | 0.000560002 | 0.003382231 | KLHL2 |
| ENSG00000136111 | 0.03532219 | 0.011867277 | -1.573585816 | 0.001410513 | 0.007233015 | TBC1D4 |
| ENSG00000185619 | 0.072901816 | 0.024471339 | -1.574861703 | 0 | 0 | PCGF3 |
| ENSG00000127483 | 0.080342886 | 0.02696514 | -1.575074746 | 0 | 0 | HP1BP3 |
| ENSG00000109118 | 0.071918018 | 0.024128084 | -1.575639889 | 2.82E-06 | 3.49E-05 | PHF12 |
| ENSG00000066651 | 0.074275185 | 0.024909857 | -1.576163538 | 0.000542079 | 0.003295503 | TRMT11 |
| ENSG00000068400 | 0.098175155 | 0.032907911 | -1.576923531 | 5.27E-13 | 2.27E-11 | GRIPAP1 |
| ENSG00000154310 | 0.121640084 | 0.040720556 | -1.578789538 | 2.17E-06 | 2.79E-05 | TNIK |
| ENSG00000153046 | 0.173774747 | 0.058162264 | -1.5790631 | 3.00E-15 | 1.76E-13 | CDYL |
| ENSG00000136935 | 0.085697007 | 0.028562629 | -1.58511603 | 7.50E-10 | 1.96E-08 | GOLGA1 |
| ENSG00000180376 | 0.105162584 | 0.035028483 | -1.586021085 | 6.20E-06 | 6.95E-05 | CCDC66 |
| ENSG00000105323 | 0.070386759 | 0.023419472 | -1.587595514 | 6.09E-11 | 1.89E-09 | HNRNPUL1 |
| ENSG00000181555 | 0.096067615 | 0.031927266 | -1.589261142 | 9.90E-06 | 0.000105577 | SETD2 |
| ENSG00000108312 | 0.151530136 | 0.05034106 | -1.589797249 | 0 | 0 | UBTF |
| ENSG00000163516 | 0.044635317 | 0.014808068 | -1.591802277 | 0.000166424 | 0.001229269 | ANKZF1 |
| ENSG00000137478 | 0.081564364 | 0.027007833 | -1.594561087 | 7.47E-06 | 8.19E-05 | FCHSD2 |
| ENSG00000121289 | 0.099599839 | 0.032977694 | -1.594652882 | 2.24E-12 | 8.73E-11 | CEP89 |
| ENSG00000052126 | 0.146399079 | 0.048393491 | -1.597021569 | 2.11E-14 | 1.10E-12 | PLEKHA5 |
| ENSG00000185989 | 0.120382544 | 0.039715011 | -1.599869887 | 7.84E-06 | 8.54E-05 | RASA3 |
| ENSG00000083896 | 0.141930927 | 0.046754334 | -1.602016964 | 1.11E-16 | 7.67E-15 | YTHDC1 |
| ENSG00000163249 | 0.069430023 | 0.022862839 | -1.602555068 | 1.80E-07 | 2.98E-06 | CCNYL1 |
| ENSG00000007047 | 0.060138876 | 0.019753337 | -1.606201554 | 4.86E-07 | 7.27E-06 | MARK4 |
| ENSG00000157827 | 0.106198085 | 0.034879729 | -1.606296996 | 2.76E-11 | 9.20E-10 | FMNL2 |
| ENSG00000130559 | 0.062318267 | 0.020449986 | -1.607555269 | 6.73E-06 | 7.45E-05 | CAMSAP1 |
| ENSG00000198467 | 0.18399486 | 0.060378297 | -1.607563492 | 1.28E-07 | 2.17E-06 | TPM2 |
| ENSG00000155313 | 0.08631164 | 0.028320422 | -1.607712354 | 5.55E-15 | 3.15E-13 | USP25 |
| ENSG00000065613 | 0.159820892 | 0.052367061 | -1.60972448 | 0 | 0 | SLK |
| ENSG00000105556 | 0.157530765 | 0.051537222 | -1.611946937 | 0.000692095 | 0.004023277 | MIER2 |
| ENSG00000163714 | 0.079008503 | 0.025841181 | -1.612335899 | 0 | 0 | U2SURP |
| ENSG00000091527 | 0.089758824 | 0.029352003 | -1.61259481 | 3.85E-11 | 1.24E-09 | CDV3 |
| ENSG00000160293 | 0.099751901 | 0.032615375 | -1.612792127 | 3.04E-09 | 7.12E-08 | VAV2 |
| ENSG00000115677 | 0.083915658 | 0.027410558 | -1.614208327 | 2.23E-14 | 1.15E-12 | HDLBP |
| ENSG00000163510 | 0.164315333 | 0.053630701 | -1.615336093 | 5.87E-12 | 2.08E-10 | CWC22 |
| ENSG00000167460 | 0.071648462 | 0.023376515 | -1.615875867 | 0 | 0 | TPM4 |
| ENSG00000148143 | 0.167414164 | 0.054609094 | -1.616208459 | 9.35E-05 | 0.000750486 | ZNF462 |
| ENSG00000102030 | 0.121607662 | 0.039626122 | -1.617710458 | 3.59E-07 | 5.53E-06 | NAA10 |
| ENSG00000011007 | 0.052498467 | 0.017098686 | -1.618389863 | 3.90E-09 | 8.95E-08 | ELOA |
| ENSG00000135945 | 0.081712487 | 0.026587805 | -1.619791858 | 1.81E-06 | 2.37E-05 | REV1 |
| ENSG00000138587 | 0.333441067 | 0.108401941 | -1.621041216 | 0.000201721 | 0.001437872 | MNS1 |
| ENSG00000131470 | 0.112519241 | 0.036530336 | -1.623004785 | 3.21E-08 | 6.27E-07 | PSMC3IP |
| ENSG00000136861 | 0.138411921 | 0.044815045 | -1.626913161 | 1.43E-10 | 4.24E-09 | CDK5RAP2 |
| ENSG00000173230 | 0.150770706 | 0.048807543 | -1.627180112 | 2.82E-06 | 3.49E-05 | GOLGB1 |
| ENSG00000101040 | 0.084459031 | 0.027339871 | -1.62724524 | 5.06E-11 | 1.61E-09 | ZMYND8 |
| ENSG00000118007 | 0.12404283 | 0.040152443 | -1.627278689 | 7.87E-08 | 1.40E-06 | STAG1 |
| ENSG00000173599 | 0.10276448 | 0.033261063 | -1.627435514 | 0.000255457 | 0.001758372 | PC |
| ENSG00000096070 | 0.063240965 | 0.020450194 | -1.628744814 | 2.63E-05 | 0.000250721 | BRPF3 |
| ENSG00000248323 | 0.209061943 | 0.067579974 | -1.629262759 | 0.000107424 | 0.000844297 | LUCAT1 |
| ENSG00000100023 | 0.085288211 | 0.027553416 | -1.630115133 | 4.62E-05 | 0.000406571 | PPIL2 |
| ENSG00000141013 | 0.228794086 | 0.073830159 | -1.631767588 | 8.03E-05 | 0.000659403 | GAS8 |
| ENSG00000110200 | 0.250930058 | 0.080929404 | -1.632549414 | 0.000155763 | 0.001159787 | ANAPC15 |
| ENSG00000108349 | 0.093672651 | 0.030152572 | -1.635346844 | 7.24E-14 | 3.51E-12 | CASC3 |
| ENSG00000173905 | 0.178338009 | 0.057339099 | -1.637023088 | 0 | 0 | GOLIM4 |
| ENSG00000204396 | 0.177211909 | 0.056945346 | -1.637825711 | 0.00173173 | 0.008559002 | VWA7 |
| ENSG00000179912 | 0.064661504 | 0.020738916 | -1.640566585 | 2.28E-08 | 4.56E-07 | R3HDM2 |
| ENSG00000198707 | 0.08518259 | 0.02731422 | -1.640906392 | 6.19E-06 | 6.93E-05 | CEP290 |
| ENSG00000144283 | 0.086364977 | 0.027681036 | -1.641548433 | 1.11E-16 | 7.67E-15 | PKP4 |
| ENSG00000133131 | 0.085722055 | 0.027459457 | -1.642363326 | 0.001007033 | 0.005459678 | MORC4 |
| ENSG00000165813 | 0.110733659 | 0.035459374 | -1.642854838 | 8.58E-05 | 0.000696728 | CCDC186 |
| ENSG00000089048 | 0.143264636 | 0.045808607 | -1.644991922 | 1.62E-12 | 6.46E-11 | ESF1 |
| ENSG00000107890 | 0.180744373 | 0.05774512 | -1.646179806 | 6.19E-07 | 9.05E-06 | ANKRD26 |
| ENSG00000089022 | 0.062218715 | 0.019869408 | -1.646799715 | 7.41E-08 | 1.32E-06 | MAPKAPK5 |
| ENSG00000127329 | 0.071214189 | 0.022695535 | -1.649756257 | 0.000554837 | 0.003359474 | PTPRB |
| ENSG00000164114 | 0.060288956 | 0.019200809 | -1.650726643 | 0.000863312 | 0.004806172 | MAP9 |
| ENSG00000182827 | 0.08874718 | 0.028206847 | -1.653655868 | 3.27E-13 | 1.49E-11 | ACBD3 |
| ENSG00000166164 | 0.040285625 | 0.01278883 | -1.655380894 | 1.50E-08 | 3.11E-07 | BRD7 |
| ENSG00000170004 | 0.098766326 | 0.031328989 | -1.656521015 | 4.91E-08 | 9.20E-07 | CHD3 |
| ENSG00000163950 | 0.132701375 | 0.042069876 | -1.657323856 | 9.22E-07 | 1.29E-05 | SLBP |
| ENSG00000008083 | 0.096739305 | 0.030584621 | -1.661295777 | 0.000534461 | 0.003252477 | JARID2 |
| ENSG00000197535 | 0.084362989 | 0.026653088 | -1.662307519 | 9.48E-07 | 1.32E-05 | MYO5A |
| ENSG00000065328 | 0.194359707 | 0.061354108 | -1.663497309 | 1.67E-08 | 3.44E-07 | MCM10 |
| ENSG00000118200 | 0.112776679 | 0.035518754 | -1.666815881 | 4.67E-11 | 1.49E-09 | CAMSAP2 |
| ENSG00000161981 | 0.12066264 | 0.037989539 | -1.667304939 | 9.59E-05 | 0.00076761 | SNRNP25 |
| ENSG00000090615 | 0.118212219 | 0.037218041 | -1.667305135 | 0 | 0 | GOLGA3 |
| ENSG00000173473 | 0.12405676 | 0.039057407 | -1.667332293 | 3.10E-14 | 1.58E-12 | SMARCC1 |
| ENSG00000101161 | 0.18522635 | 0.058282834 | -1.668146403 | 2.66E-15 | 1.59E-13 | PRPF6 |
| ENSG00000130749 | 0.136818931 | 0.042784444 | -1.677109601 | 5.68E-07 | 8.39E-06 | ZC3H4 |
| ENSG00000033030 | 0.120555916 | 0.037628881 | -1.679790171 | 3.84E-14 | 1.93E-12 | ZCCHC8 |
| ENSG00000054267 | 0.130965249 | 0.040876839 | -1.67982852 | 0 | 0 | ARID4B |
| ENSG00000197345 | 0.186934687 | 0.058312223 | -1.680664081 | 1.62E-07 | 2.71E-06 | MRPL21 |
| ENSG00000196418 | 0.474281441 | 0.147937008 | -1.680760413 | 4.72E-07 | 7.10E-06 | ZNF124 |
| ENSG00000186566 | 0.095269839 | 0.029710821 | -1.681031097 | 2.47E-05 | 0.000237204 | GPATCH8 |
| ENSG00000104375 | 0.179793297 | 0.056045709 | -1.681663397 | 3.05E-07 | 4.79E-06 | STK3 |
| ENSG00000198920 | 0.110748236 | 0.034515172 | -1.681981137 | 3.38E-09 | 7.83E-08 | KIAA0753 |
| ENSG00000198000 | 0.136715915 | 0.04256571 | -1.683417611 | 1.11E-16 | 7.67E-15 | NOL8 |
| ENSG00000125648 | 0.031871853 | 0.009921773 | -1.683613024 | 8.87E-05 | 0.000716448 | SLC25A23 |
| ENSG00000132275 | 0.065883701 | 0.020493054 | -1.684786637 | 1.59E-05 | 0.000160008 | RRP8 |
| ENSG00000166813 | 0.120797208 | 0.037479792 | -1.688402251 | 1.53E-07 | 2.56E-06 | KIF7 |
| ENSG00000196628 | 0.092450115 | 0.028678451 | -1.688708003 | 0.000436447 | 0.00273348 | TCF4 |
| ENSG00000109881 | 0.391665055 | 0.121482451 | -1.688872489 | 1.99E-08 | 4.02E-07 | CCDC34 |
| ENSG00000148296 | 0.088487449 | 0.027440349 | -1.689173999 | 9.26E-09 | 1.98E-07 | SURF6 |
| ENSG00000170633 | 0.166179216 | 0.05147841 | -1.690700562 | 4.71E-12 | 1.71E-10 | RNF34 |
| ENSG00000183258 | 0.125582615 | 0.038782087 | -1.69517441 | 0 | 0 | DDX41 |
| ENSG00000015171 | 0.082490421 | 0.025472165 | -1.695304997 | 5.55E-16 | 3.57E-14 | ZMYND11 |
| ENSG00000161267 | 0.067483364 | 0.020831286 | -1.69577999 | 9.07E-06 | 9.76E-05 | BDH1 |
| ENSG00000156650 | 0.140933894 | 0.043501941 | -1.695866938 | 0.000436749 | 0.002733943 | KAT6B |
| ENSG00000155827 | 0.145628667 | 0.044917669 | -1.696939413 | 5.52E-08 | 1.02E-06 | RNF20 |
| ENSG00000113141 | 0.10112359 | 0.031155156 | -1.698576729 | 2.83E-08 | 5.59E-07 | IK |
| ENSG00000128872 | 0.071322237 | 0.021955234 | -1.699787044 | 9.72E-07 | 1.35E-05 | TMOD2 |
| ENSG00000100014 | 0.014903145 | 0.004585677 | -1.700410113 | 0.000491682 | 0.003027381 | SPECC1L |
| ENSG00000125482 | 0.103699767 | 0.031899942 | -1.700786933 | 2.00E-05 | 0.000195559 | TTF1 |
| ENSG00000084072 | 0.05728786 | 0.017606999 | -1.702080429 | 1.68E-07 | 2.80E-06 | PPIE |
| ENSG00000178105 | 0.211717408 | 0.064970144 | -1.704291085 | 5.75E-09 | 1.28E-07 | DDX10 |
| ENSG00000083223 | 0.192837282 | 0.059111656 | -1.705869458 | 1.45E-06 | 1.94E-05 | TUT7 |
| ENSG00000167515 | 0.0983953 | 0.030140727 | -1.706875191 | 4.89E-05 | 0.000426973 | TRAPPC2L |
| ENSG00000127914 | 0.140756373 | 0.043098899 | -1.707477308 | 4.00E-06 | 4.74E-05 | AKAP9 |
| ENSG00000028310 | 0.095492133 | 0.029229581 | -1.707952752 | 0 | 0 | BRD9 |
| ENSG00000072571 | 0.136356001 | 0.041731801 | -1.708159093 | 0 | 0 | HMMR |
| ENSG00000188321 | 0.021687832 | 0.006631959 | -1.70937878 | 0.000478781 | 0.002957277 | ZNF559 |
| ENSG00000153574 | 0.030968951 | 0.009464682 | -1.710196622 | 0.000718489 | 0.004140194 | RPIA |
| ENSG00000101350 | 0.074908463 | 0.022882949 | -1.710855742 | 1.85E-11 | 6.29E-10 | KIF3B |
| ENSG00000131016 | 0.301291415 | 0.09194889 | -1.712255512 | 3.26E-10 | 9.12E-09 | AKAP12 |
| ENSG00000235162 | 0.209510339 | 0.063830618 | -1.714700915 | 2.29E-07 | 3.66E-06 | C12orf75 |
| ENSG00000145555 | 0.130845768 | 0.039857727 | -1.714935919 | 1.98E-06 | 2.57E-05 | MYO10 |
| ENSG00000108771 | 0.024778267 | 0.007546403 | -1.715214165 | 0.000653635 | 0.003849149 | DHX58 |
| ENSG00000099901 | 0.062947876 | 0.019120163 | -1.719062896 | 8.93E-06 | 9.62E-05 | RANBP1 |
| ENSG00000144161 | 0.086592699 | 0.026196626 | -1.724864356 | 8.33E-05 | 0.000679604 | ZC3H8 |
| ENSG00000059769 | 0.211504064 | 0.063909793 | -1.726576479 | 2.70E-06 | 3.36E-05 | DNAJC25 |
| ENSG00000120370 | 0.169635295 | 0.051159639 | -1.729358386 | 6.62E-05 | 0.000557719 | GORAB |
| ENSG00000060339 | 0.168138052 | 0.05063181 | -1.731530313 | 0 | 0 | CCAR1 |
| ENSG00000048991 | 0.03973892 | 0.011955929 | -1.732826384 | 5.32E-05 | 0.000458819 | R3HDM1 |
| ENSG00000087365 | 0.282417601 | 0.084913477 | -1.733764552 | 0 | 0 | SF3B2 |
| ENSG00000100281 | 0.126736864 | 0.038082734 | -1.734627265 | 0 | 0 | HMGXB4 |
| ENSG00000118412 | 0.079054903 | 0.023753884 | -1.734691538 | 3.48E-05 | 0.00031826 | CASP8AP2 |
| ENSG00000011260 | 0.140864872 | 0.042296635 | -1.735697081 | 5.54E-09 | 1.24E-07 | UTP18 |
| ENSG00000114770 | 0.050993982 | 0.015309596 | -1.735890769 | 0.00010311 | 0.000816259 | ABCC5 |
| ENSG00000147010 | 0.107168331 | 0.032133621 | -1.73772318 | 1.24E-09 | 3.13E-08 | SH3KBP1 |
| ENSG00000132670 | 0.082091415 | 0.024582783 | -1.739583096 | 0 | 0 | PTPRA |
| ENSG00000122257 | 0.090087477 | 0.026977223 | -1.739584713 | 2.80E-13 | 1.28E-11 | RBBP6 |
| ENSG00000048649 | 0.220014472 | 0.065883867 | -1.739601277 | 7.19E-09 | 1.56E-07 | RSF1 |
| ENSG00000065534 | 0.114136387 | 0.034101003 | -1.742872745 | 6.68E-05 | 0.000560313 | MYLK |
| ENSG00000166197 | 0.102699568 | 0.030681946 | -1.742968241 | 0 | 0 | NOLC1 |
| ENSG00000156876 | 0.063708199 | 0.018989417 | -1.746283411 | 4.69E-06 | 5.43E-05 | SASS6 |
| ENSG00000099331 | 0.086955772 | 0.025912527 | -1.746632065 | 7.92E-07 | 1.13E-05 | MYO9B |
| ENSG00000114473 | 0.156250098 | 0.046540707 | -1.747292076 | 0.000120708 | 0.000935254 | IQCG |
| ENSG00000096401 | 0.155053287 | 0.046160556 | -1.748031611 | 0 | 0 | CDC5L |
| ENSG00000204209 | 0.121946664 | 0.036165272 | -1.753573381 | 0 | 0 | DAXX |
| ENSG00000168386 | 0.138340787 | 0.040967073 | -1.755689829 | 2.54E-06 | 3.20E-05 | FILIP1L |
| ENSG00000175467 | 0.213094881 | 0.063041615 | -1.75711954 | 0 | 0 | SART1 |
| ENSG00000152457 | 0.051384773 | 0.015200457 | -1.757226249 | 0.001303065 | 0.00675986 | DCLRE1C |
| ENSG00000129347 | 0.328492747 | 0.097163458 | -1.75737578 | 0 | 0 | KRI1 |
| ENSG00000196562 | 0.100851441 | 0.029753917 | -1.761080174 | 0.000127119 | 0.000976745 | SULF2 |
| ENSG00000124228 | 0.18467317 | 0.054470589 | -1.761424917 | 0 | 0 | DDX27 |
| ENSG00000176788 | 0.060228771 | 0.017761793 | -1.761675629 | 0 | 0 | BASP1 |
| ENSG00000105048 | 0.065905218 | 0.019426353 | -1.7623776 | 4.83E-06 | 5.57E-05 | TNNT1 |
| ENSG00000120158 | 0.112343237 | 0.033086361 | -1.763604753 | 8.05E-07 | 1.15E-05 | RCL1 |
| ENSG00000184863 | 0.114093864 | 0.033562565 | -1.765296316 | 2.54E-07 | 4.04E-06 | RBM33 |
| ENSG00000099381 | 0.069787212 | 0.020503978 | -1.767058869 | 1.10E-06 | 1.51E-05 | SETD1A |
| ENSG00000115241 | 0.161613436 | 0.047460992 | -1.767732982 | 1.33E-15 | 8.26E-14 | PPM1G |
| ENSG00000183955 | 0.15321847 | 0.044921559 | -1.770110331 | 1.45E-12 | 5.88E-11 | KMT5A |
| ENSG00000005339 | 0.101258321 | 0.029663169 | -1.771295808 | 0.000257241 | 0.001768625 | CREBBP |
| ENSG00000116991 | 0.133150006 | 0.038947834 | -1.773437484 | 8.92E-05 | 0.000718843 | SIPA1L2 |
| ENSG00000140939 | 0.028118867 | 0.008222608 | -1.77387041 | 0.00122717 | 0.006438357 | NOL3 |
| ENSG00000066455 | 0.132810607 | 0.038806583 | -1.774997049 | 2.83E-12 | 1.09E-10 | GOLGA5 |
| ENSG00000106052 | 0.102917116 | 0.030032459 | -1.776888442 | 3.72E-12 | 1.39E-10 | TAX1BP1 |
| ENSG00000107262 | 0.060675091 | 0.017650209 | -1.781419134 | 0.000137298 | 0.001040987 | BAG1 |
| ENSG00000101361 | 0.150718118 | 0.043734264 | -1.785016936 | 2.89E-11 | 9.61E-10 | NOP56 |
| ENSG00000198783 | 0.102868879 | 0.029808373 | -1.787017047 | 0.001429883 | 0.007307411 | ZNF830 |
| ENSG00000147649 | 0.101063207 | 0.029221956 | -1.790133221 | 0 | 0 | MTDH |
| ENSG00000135899 | 0.205731266 | 0.059322922 | -1.794099505 | 1.32E-09 | 3.30E-08 | SP110 |
| ENSG00000139718 | 0.110375481 | 0.031819424 | -1.794440116 | 0.00032857 | 0.002159048 | SETD1B |
| ENSG00000205464 | 0.032605657 | 0.009395887 | -1.795021006 | 0.000290576 | 0.00195247 | ATP6AP1L |
| ENSG00000125386 | 0.089152845 | 0.025561597 | -1.802302859 | 0.000141291 | 0.001065886 | FAM193A |
| ENSG00000181350 | 0.032192289 | 0.009227031 | -1.802776757 | 0.000286647 | 0.00192997 | LRRC75A |
| ENSG00000198301 | 0.124550749 | 0.035687036 | -1.803261719 | 2.78E-15 | 1.64E-13 | SDAD1 |
| ENSG00000135931 | 0.219501735 | 0.062874642 | -1.803682151 | 3.39E-10 | 9.45E-09 | ARMC9 |
| ENSG00000152404 | 0.169094315 | 0.048405012 | -1.804599803 | 3.20E-08 | 6.25E-07 | CWF19L2 |
| ENSG00000171444 | 0.088068698 | 0.025194299 | -1.805532001 | 5.91E-05 | 0.000505157 | MCC |
| ENSG00000186522 | 0.102159281 | 0.029214817 | -1.806048095 | 0 | 0 | 10-Sep |
| ENSG00000023892 | 0.081562618 | 0.023314413 | -1.806686006 | 0.000125674 | 0.000968738 | DEF6 |
| ENSG00000081041 | 0.016892552 | 0.004826595 | -1.807309781 | 0.001083984 | 0.005813966 | CXCL2 |
| ENSG00000173575 | 0.093521851 | 0.026720339 | -1.807365176 | 5.61E-08 | 1.03E-06 | CHD2 |
| ENSG00000081026 | 0.095088953 | 0.027134067 | -1.809172463 | 5.29E-05 | 0.000456338 | MAGI3 |
| ENSG00000196498 | 0.071033382 | 0.020256605 | -1.810104795 | 5.06E-05 | 0.000440001 | NCOR2 |
| ENSG00000117133 | 0.096087856 | 0.02737421 | -1.811536804 | 1.19E-06 | 1.63E-05 | RPF1 |
| ENSG00000234741 | 3.915853134 | 1.114622715 | -1.812771197 | 3.26E-09 | 7.56E-08 | GAS5 |
| ENSG00000069275 | 0.143138719 | 0.040727862 | -1.813325991 | 0 | 0 | NUCKS1 |
| ENSG00000272779 | 0.056854228 | 0.016146193 | -1.816073618 | 2.69E-06 | 3.35E-05 | AC245060.4 |
| ENSG00000198160 | 0.184355452 | 0.052256005 | -1.818821352 | 0 | 0 | MIER1 |
| ENSG00000074356 | 0.113824757 | 0.032262341 | -1.818891361 | 2.10E-14 | 1.10E-12 | NCBP3 |
| ENSG00000115289 | 0.056434039 | 0.015977993 | -1.820479399 | 0.00033597 | 0.002196868 | PCGF1 |
| ENSG00000143970 | 0.092170142 | 0.026069653 | -1.821928088 | 6.35E-06 | 7.08E-05 | ASXL2 |
| ENSG00000135966 | 0.065578358 | 0.018486243 | -1.826767714 | 5.54E-07 | 8.21E-06 | TGFBRAP1 |
| ENSG00000054654 | 0.099256997 | 0.027965848 | -1.827502717 | 0.000244914 | 0.001700399 | SYNE2 |
| ENSG00000106244 | 0.094422245 | 0.026583457 | -1.828598064 | 0 | 0 | PDAP1 |
| ENSG00000101421 | 0.118300601 | 0.033301466 | -1.828799794 | 2.55E-15 | 1.54E-13 | CHMP4B |
| ENSG00000107104 | 0.098347568 | 0.027676487 | -1.829228546 | 0.000688229 | 0.004006083 | KANK1 |
| ENSG00000171219 | 0.053742588 | 0.015113982 | -1.830181963 | 0.000322836 | 0.002130684 | CDC42BPG |
| ENSG00000108091 | 0.154190105 | 0.043311681 | -1.831882131 | 0 | 0 | CCDC6 |
| ENSG00000108848 | 0.111521791 | 0.031298991 | -1.83313759 | 0 | 0 | LUC7L3 |
| ENSG00000142864 | 0.065813424 | 0.018456564 | -1.834247836 | 2.66E-15 | 1.59E-13 | SERBP1 |
| ENSG00000181090 | 0.098803024 | 0.027686869 | -1.835353295 | 0 | 0 | EHMT1 |
| ENSG00000116580 | 0.133073268 | 0.037256469 | -1.836657926 | 0.000922395 | 0.005075838 | GON4L |
| ENSG00000248527 | 0.002167072 | 0.000605118 | -1.840459341 | 0.000244724 | 0.001700062 | MTATP6P1 |
| ENSG00000131626 | 0.115645872 | 0.032228732 | -1.843294452 | 3.17E-08 | 6.20E-07 | PPFIA1 |
| ENSG00000248333 | 0.039429665 | 0.010985708 | -1.843653591 | 1.11E-10 | 3.33E-09 | CDK11B |
| ENSG00000197582 | 0.282476551 | 0.078630008 | -1.844979199 | 0.000791095 | 0.004491462 | GPX1P1 |
| ENSG00000102241 | 0.14173531 | 0.039450442 | -1.845085863 | 6.86E-10 | 1.81E-08 | HTATSF1 |
| ENSG00000126001 | 0.089228587 | 0.024826336 | -1.845634628 | 1.21E-07 | 2.07E-06 | CEP250 |
| ENSG00000196670 | 0.051275849 | 0.01424362 | -1.847963613 | 2.02E-06 | 2.62E-05 | ZFP62 |
| ENSG00000138835 | 0.052645371 | 0.014581714 | -1.852146409 | 1.90E-05 | 0.000187667 | RGS3 |
| ENSG00000155275 | 0.071660132 | 0.019769824 | -1.857870684 | 0.000192273 | 0.001385311 | TRMT44 |
| ENSG00000188177 | 0.138319302 | 0.038153868 | -1.858101257 | 7.56E-07 | 1.09E-05 | ZC3H6 |
| ENSG00000136153 | 0.071904993 | 0.019824186 | -1.858830342 | 4.75E-14 | 2.35E-12 | LMO7 |
| ENSG00000102043 | 0.702792547 | 0.193620353 | -1.859868278 | 0.000875433 | 0.00486015 | MTMR8 |
| ENSG00000100916 | 0.22644666 | 0.062369162 | -1.860266485 | 6.12E-06 | 6.87E-05 | BRMS1L |
| ENSG00000134884 | 0.039523187 | 0.01087671 | -1.861457056 | 0 | 0 | ARGLU1 |
| ENSG00000168404 | 0.139873771 | 0.038439095 | -1.863479166 | 1.64E-05 | 0.000163913 | MLKL |
| ENSG00000058272 | 0.199403778 | 0.054762076 | -1.864443691 | 0 | 0 | PPP1R12A |
| ENSG00000266088 | 0.496869753 | 0.136377002 | -1.865267342 | 0.000895287 | 0.004945256 | AC004585.1 |
| ENSG00000198176 | 0.078579059 | 0.021518468 | -1.868569531 | 0 | 0 | TFDP1 |
| ENSG00000128191 | 0.085310122 | 0.023303838 | -1.872149372 | 7.98E-07 | 1.14E-05 | DGCR8 |
| ENSG00000157510 | 0.068230185 | 0.018601195 | -1.875014856 | 1.00E-07 | 1.74E-06 | AFAP1L1 |
| ENSG00000265241 | 0.110398239 | 0.030093259 | -1.875204878 | 1.45E-06 | 1.94E-05 | RBM8A |
| ENSG00000160208 | 0.136875234 | 0.037296449 | -1.875751254 | 2.91E-12 | 1.11E-10 | RRP1B |
| ENSG00000086712 | 0.138215175 | 0.037650742 | -1.876165818 | 5.94E-13 | 2.53E-11 | TXLNG |
| ENSG00000008710 | 0.015057406 | 0.004091663 | -1.879713887 | 0.001875807 | 0.009119992 | PKD1 |
| ENSG00000130956 | 0.130276545 | 0.035395812 | -1.879926802 | 5.10E-11 | 1.61E-09 | HABP4 |
| ENSG00000107863 | 0.020366354 | 0.005519807 | -1.883497887 | 0.000124324 | 0.000959559 | ARHGAP21 |
| ENSG00000135837 | 0.112595881 | 0.030510473 | -1.883777608 | 4.23E-06 | 4.99E-05 | CEP350 |
| ENSG00000188343 | 0.086668107 | 0.02348296 | -1.883886954 | 1.99E-06 | 2.58E-05 | FAM92A |
| ENSG00000263001 | 0.233507754 | 0.063144535 | -1.886740685 | 1.81E-07 | 2.99E-06 | GTF2I |
| ENSG00000160818 | 0.160631983 | 0.043407976 | -1.88772713 | 2.25E-11 | 7.53E-10 | GPATCH4 |
| ENSG00000135250 | 0.209896822 | 0.056677592 | -1.888829954 | 0 | 0 | SRPK2 |
| ENSG00000122786 | 0.129528059 | 0.03494613 | -1.890060053 | 0 | 0 | CALD1 |
| ENSG00000112739 | 0.11070716 | 0.029833715 | -1.891733009 | 2.00E-15 | 1.21E-13 | PRPF4B |
| ENSG00000141858 | 0.102159819 | 0.027467705 | -1.895019576 | 6.22E-06 | 6.96E-05 | SAMD1 |
| ENSG00000164985 | 0.105566458 | 0.02831698 | -1.898412183 | 0 | 0 | PSIP1 |
| ENSG00000008311 | 0.155229958 | 0.041632099 | -1.898638813 | 0.000304095 | 0.002025896 | AASS |
| ENSG00000029725 | 0.100781518 | 0.02701481 | -1.89940863 | 0 | 0 | RABEP1 |
| ENSG00000082805 | 0.125975996 | 0.033744104 | -1.900441524 | 2.56E-06 | 3.22E-05 | ERC1 |
| ENSG00000140259 | 0.196861557 | 0.052626838 | -1.903310797 | 6.07E-13 | 2.57E-11 | MFAP1 |
| ENSG00000070081 | 0.201387785 | 0.053762027 | -1.905316755 | 0 | 0 | NUCB2 |
| ENSG00000171316 | 0.133157348 | 0.035457894 | -1.908953309 | 7.78E-06 | 8.49E-05 | CHD7 |
| ENSG00000133789 | 0.17790985 | 0.047345571 | -1.909845001 | 0 | 0 | SWAP70 |
| ENSG00000196504 | 0.158192915 | 0.042027616 | -1.912275446 | 0 | 0 | PRPF40A |
| ENSG00000165630 | 0.322589746 | 0.08549071 | -1.915861019 | 0.000410063 | 0.002606242 | PRPF18 |
| ENSG00000200534 | 4.377105713 | 1.159430298 | -1.916561135 | 0.001167215 | 0.006182363 | SNORA33 |
| ENSG00000143674 | 0.085347191 | 0.022592668 | -1.917489045 | 0.000270386 | 0.001842146 | MAP3K21 |
| ENSG00000147133 | 0.078420767 | 0.02074142 | -1.91872109 | 8.15E-10 | 2.11E-08 | TAF1 |
| ENSG00000117480 | 0.268233029 | 0.070931061 | -1.91899746 | 0.001943359 | 0.009377563 | FAAH |
| ENSG00000160299 | 0.13206794 | 0.034920578 | -1.919130941 | 2.51E-06 | 3.18E-05 | PCNT |
| ENSG00000154767 | 0.174202348 | 0.046055549 | -1.919317192 | 2.13E-06 | 2.75E-05 | XPC |
| ENSG00000135521 | 0.382965918 | 0.101212638 | -1.919826567 | 4.53E-05 | 0.000399162 | LTV1 |
| ENSG00000081386 | 0.056806674 | 0.01498777 | -1.92227465 | 0.000281613 | 0.001903542 | ZNF510 |
| ENSG00000102362 | 0.144887267 | 0.038155435 | -1.924970314 | 0.000424061 | 0.002675412 | SYTL4 |
| ENSG00000101882 | 0.196454587 | 0.051647673 | -1.927420592 | 0.000190026 | 0.001370765 | NKAP |
| ENSG00000072501 | 0.149362038 | 0.039174391 | -1.930830767 | 4.82E-12 | 1.74E-10 | SMC1A |
| ENSG00000085224 | 0.126250229 | 0.033104057 | -1.931206061 | 5.31E-11 | 1.66E-09 | ATRX |
| ENSG00000197903 | 0.050459245 | 0.013223378 | -1.932027864 | 0.000842976 | 0.004714786 | HIST1H2BK |
| ENSG00000273559 | 0.171604438 | 0.044899598 | -1.934312417 | 6.80E-11 | 2.10E-09 | CWC25 |
| ENSG00000143079 | 0.107885968 | 0.028219289 | -1.934753685 | 1.65E-06 | 2.19E-05 | CTTNBP2NL |
| ENSG00000144118 | 0.12616451 | 0.03299688 | -1.934904606 | 0 | 0 | RALB |
| ENSG00000126653 | 0.134159783 | 0.034945913 | -1.940756633 | 6.22E-10 | 1.66E-08 | NSRP1 |
| ENSG00000141582 | 0.040008828 | 0.0103877 | -1.945442083 | 1.62E-07 | 2.71E-06 | CBX4 |
| ENSG00000196535 | 0.119459306 | 0.030956734 | -1.948194054 | 0.000205075 | 0.001456604 | MYO18A |
| ENSG00000105894 | 0.154982206 | 0.040133444 | -1.949225703 | 0.001280741 | 0.00667044 | PTN |
| ENSG00000161647 | 0.101188477 | 0.026179125 | -1.950556255 | 1.35E-06 | 1.82E-05 | MPP3 |
| ENSG00000187109 | 0.119453527 | 0.030879144 | -1.951744801 | 7.43E-12 | 2.60E-10 | NAP1L1 |
| ENSG00000162601 | 0.061107668 | 0.015785382 | -1.952764276 | 4.37E-06 | 5.13E-05 | MYSM1 |
| ENSG00000100503 | 0.141438636 | 0.036482639 | -1.954894277 | 0 | 0 | NIN |
| ENSG00000109111 | 0.14651028 | 0.037788232 | -1.954992975 | 4.63E-10 | 1.26E-08 | SUPT6H |
| ENSG00000119698 | 0.02840045 | 0.007316492 | -1.956689786 | 0.000334508 | 0.002192074 | PPP4R4 |
| ENSG00000084463 | 0.06662207 | 0.017161174 | -1.956851952 | 0 | 0 | WBP11 |
| ENSG00000162645 | 0.062215079 | 0.01597941 | -1.961050199 | 7.24E-05 | 0.000600163 | GBP2 |
| ENSG00000075151 | 0.119427387 | 0.030618519 | -1.963657301 | 1.26E-11 | 4.31E-10 | EIF4G3 |
| ENSG00000038382 | 0.123165438 | 0.031440376 | -1.969907094 | 1.58E-05 | 0.000158851 | TRIO |
| ENSG00000204256 | 0.055003257 | 0.013969017 | -1.977286531 | 3.33E-16 | 2.21E-14 | BRD2 |
| ENSG00000257511 | 0.275622209 | 0.069729313 | -1.982854969 | 0.00039415 | 0.002511743 | AC084824.1 |
| ENSG00000136560 | 0.165143644 | 0.041764178 | -1.98338349 | 5.10E-13 | 2.20E-11 | TANK |
| ENSG00000109576 | 0.047814717 | 0.012080483 | -1.984776565 | 0.00015229 | 0.001139568 | AADAT |
| ENSG00000048392 | 0.013254847 | 0.003344682 | -1.986578917 | 0.000466274 | 0.002890175 | RRM2B |
| ENSG00000139613 | 0.147152837 | 0.037099941 | -1.987826571 | 4.79E-05 | 0.000419783 | SMARCC2 |
| ENSG00000182923 | 0.115409814 | 0.029093586 | -1.98799287 | 2.19E-10 | 6.34E-09 | CEP63 |
| ENSG00000205683 | 0.173682149 | 0.043724637 | -1.989931166 | 0.000155458 | 0.001158232 | DPF3 |
| ENSG00000209042 | 29.71618006 | 7.48018132 | -1.990103526 | 0.001559882 | 0.007841796 | SNORD12C |
| ENSG00000151914 | 0.099315303 | 0.02497997 | -1.9912443 | 3.60E-05 | 0.000328402 | DST |
| ENSG00000179119 | 0.107599803 | 0.027050673 | -1.991939055 | 6.87E-08 | 1.24E-06 | SPTY2D1 |
| ENSG00000149503 | 0.207460234 | 0.052144078 | -1.992259496 | 3.81E-10 | 1.05E-08 | INCENP |
| ENSG00000160310 | 0.079959775 | 0.020093636 | -1.9925358 | 0 | 0 | PRMT2 |
| ENSG00000135094 | 0.497244581 | 0.124863581 | -1.993602903 | 0.000769456 | 0.00438517 | SDS |
| ENSG00000188596 | 0.352218241 | 0.088414703 | -1.994111414 | 0.00068828 | 0.004006083 | CFAP54 |
| ENSG00000204104 | 0.148237465 | 0.037127362 | -1.997355407 | 0 | 0 | TRAF3IP1 |
| ENSG00000173846 | 0.102459822 | 0.025596775 | -2.001024311 | 3.62E-08 | 7.02E-07 | PLK3 |
| ENSG00000112419 | 0.308136979 | 0.076587115 | -2.00839823 | 0 | 0 | PHACTR2 |
| ENSG00000031003 | 0.121417477 | 0.03008563 | -2.012829633 | 0 | 0 | FAM13B |
| ENSG00000009780 | 0.075375748 | 0.018660854 | -2.014085398 | 3.40E-05 | 0.000312261 | FAM76A |
| ENSG00000214941 | 0.054191046 | 0.013415427 | -2.014161565 | 0.000290638 | 0.00195247 | ZSWIM7 |
| ENSG00000104611 | 0.131453997 | 0.032520535 | -2.015135136 | 1.01E-08 | 2.14E-07 | SH2D4A |
| ENSG00000140479 | 0.575198487 | 0.142152225 | -2.016623204 | 0.000811465 | 0.004576861 | PCSK6 |
| ENSG00000188529 | 0.139515568 | 0.034421148 | -2.019058977 | 0 | 0 | SRSF10 |
| ENSG00000171634 | 0.227697138 | 0.056128017 | -2.020323173 | 3.32E-06 | 4.04E-05 | BPTF |
| ENSG00000114107 | 0.047390827 | 0.011680298 | -2.020530824 | 0.000541019 | 0.003290722 | CEP70 |
| ENSG00000162545 | 0.124338104 | 0.030555624 | -2.024758647 | 0.000180681 | 0.001315987 | CAMK2N1 |
| ENSG00000197442 | 0.116519942 | 0.028552961 | -2.028864625 | 0.000747076 | 0.004269766 | MAP3K5 |
| ENSG00000134371 | 0.153643108 | 0.037341352 | -2.040736977 | 0 | 0 | CDC73 |
| ENSG00000163512 | 0.106080014 | 0.02577185 | -2.041284892 | 3.34E-12 | 1.26E-10 | AZI2 |
| ENSG00000172985 | 0.034263953 | 0.008301478 | -2.045251381 | 0.001766222 | 0.008697304 | SH3RF3 |
| ENSG00000154781 | 0.28141961 | 0.067428682 | -2.061288555 | 0 | 0 | CCDC174 |
| ENSG00000125398 | 0.071363311 | 0.017083882 | -2.062546754 | 1.46E-10 | 4.30E-09 | SOX9 |
| ENSG00000175550 | 0.187543793 | 0.044882057 | -2.063016821 | 1.04E-11 | 3.60E-10 | DRAP1 |
| ENSG00000134874 | 0.104774077 | 0.025056342 | -2.064034098 | 0 | 0 | DZIP1 |
| ENSG00000207547 | 166.6866807 | 39.82254993 | -2.06548132 | 0.00028638 | 0.001929253 | MIR25 |
| ENSG00000137817 | 0.059466884 | 0.014196792 | -2.066521479 | 2.69E-13 | 1.23E-11 | PARP6 |
| ENSG00000101132 | 0.699831194 | 0.166987059 | -2.06727067 | 0.000553574 | 0.003353517 | PFDN4 |
| ENSG00000144395 | 0.076166696 | 0.018158727 | -2.068497208 | 9.34E-07 | 1.30E-05 | CCDC150 |
| ENSG00000140044 | 0.078275111 | 0.018659105 | -2.068673873 | 4.47E-08 | 8.47E-07 | JDP2 |
| ENSG00000197321 | 0.06626677 | 0.015794257 | -2.068885517 | 6.61E-05 | 0.000557719 | SVIL |
| ENSG00000158417 | 0.1684246 | 0.040060303 | -2.071857617 | 0 | 0 | EIF5B |
| ENSG00000169136 | 0.058340146 | 0.013857618 | -2.073809687 | 2.48E-08 | 4.93E-07 | ATF5 |
| ENSG00000115504 | 0.231524508 | 0.054947121 | -2.07504911 | 2.48E-14 | 1.27E-12 | EHBP1 |
| ENSG00000212232 | 2.525275523 | 0.598025038 | -2.07816301 | 1.07E-09 | 2.70E-08 | SNORD17 |
| ENSG00000128487 | 0.178561922 | 0.042076203 | -2.08534804 | 0 | 0 | SPECC1 |
| ENSG00000198604 | 0.119199637 | 0.027926211 | -2.093688073 | 0 | 0 | BAZ1A |
| ENSG00000145220 | 0.285184741 | 0.066717062 | -2.095769125 | 7.53E-08 | 1.34E-06 | LYAR |
| ENSG00000151693 | 0.200535618 | 0.046680612 | -2.102963131 | 8.23E-06 | 8.92E-05 | ASAP2 |
| ENSG00000129518 | 0.36502888 | 0.084739923 | -2.106896892 | 2.88E-10 | 8.14E-09 | EAPP |
| ENSG00000124177 | 0.125460896 | 0.02911784 | -2.107262526 | 0.000130183 | 0.000996399 | CHD6 |
| ENSG00000208772 | 50.58855487 | 11.7326993 | -2.108276061 | 0.000680654 | 0.00397225 | SNORD94 |
| ENSG00000143365 | 0.720125464 | 0.166494156 | -2.112776747 | 0.000746173 | 0.004268662 | RORC |
| ENSG00000189337 | 0.04403679 | 0.010152097 | -2.116931522 | 1.72E-07 | 2.86E-06 | KAZN |
| ENSG00000040275 | 0.153802469 | 0.035314366 | -2.122751566 | 4.70E-14 | 2.33E-12 | SPDL1 |
| ENSG00000128908 | 0.128328734 | 0.029446108 | -2.123695403 | 6.50E-09 | 1.43E-07 | INO80 |
| ENSG00000196338 | 0.269892095 | 0.061903901 | -2.124280484 | 0.001348658 | 0.006960348 | NLGN3 |
| ENSG00000121769 | 0.117392584 | 0.026895495 | -2.125904821 | 0.000148606 | 0.001114083 | FABP3 |
| ENSG00000156398 | 0.091428376 | 0.020908248 | -2.128569791 | 4.48E-06 | 5.23E-05 | SFXN2 |
| ENSG00000126803 | 0.147991736 | 0.033835479 | -2.128907885 | 4.82E-08 | 9.05E-07 | HSPA2 |
| ENSG00000123636 | 0.166055947 | 0.037950907 | -2.129463125 | 4.81E-11 | 1.53E-09 | BAZ2B |
| ENSG00000166477 | 0.465311484 | 0.106213464 | -2.13123014 | 0 | 0 | LEO1 |
| ENSG00000027075 | 0.112134151 | 0.025556378 | -2.133470457 | 9.17E-05 | 0.000737976 | PRKCH |
| ENSG00000255974 | 0.734339133 | 0.167242227 | -2.134507325 | 0.001299988 | 0.006749721 | CYP2A6 |
| ENSG00000090061 | 0.116574315 | 0.026538107 | -2.13511257 | 0.001196556 | 0.006299731 | CCNK |
| ENSG00000032219 | 0.181026987 | 0.041163913 | -2.136752754 | 3.22E-11 | 1.06E-09 | ARID4A |
| ENSG00000087269 | 0.36326242 | 0.082524929 | -2.138110221 | 0 | 0 | NOP14 |
| ENSG00000166831 | 0.07024331 | 0.015918604 | -2.141647036 | 0.002096635 | 0.00999519 | RBPMS2 |
| ENSG00000198900 | 0.158666463 | 0.03595219 | -2.141845654 | 0 | 0 | TOP1 |
| ENSG00000164104 | 0.148201552 | 0.03350459 | -2.145129898 | 1.09E-10 | 3.30E-09 | HMGB2 |
| ENSG00000105821 | 0.168790834 | 0.038108568 | -2.147049239 | 0 | 0 | DNAJC2 |
| ENSG00000189001 | 0.70031161 | 0.157960194 | -2.14843596 | 0.001727533 | 0.008548801 | SBSN |
| ENSG00000170417 | 0.10933476 | 0.024636279 | -2.149895894 | 0.000102683 | 0.00081449 | TMEM182 |
| ENSG00000092853 | 0.169102149 | 0.038102108 | -2.149952267 | 2.32E-05 | 0.000223772 | CLSPN |
| ENSG00000144674 | 0.200982285 | 0.045151202 | -2.15423205 | 0 | 0 | GOLGA4 |
| ENSG00000167522 | 0.113085657 | 0.025385111 | -2.155361507 | 1.02E-05 | 0.000108511 | ANKRD11 |
| ENSG00000119707 | 0.325079139 | 0.072596199 | -2.16282506 | 0 | 0 | RBM25 |
| ENSG00000146904 | 0.642825383 | 0.143252009 | -2.165871529 | 0.001261192 | 0.006582393 | EPHA1 |
| ENSG00000117266 | 0.040744735 | 0.009063196 | -2.168521849 | 5.17E-05 | 0.000448004 | CDK18 |
| ENSG00000230359 | 0.244394479 | 0.054108565 | -2.1752828 | 0.000247859 | 0.001716078 | TPI1P2 |
| ENSG00000139116 | 0.103472653 | 0.022819511 | -2.180909759 | 6.90E-08 | 1.24E-06 | KIF21A |
| ENSG00000152782 | 0.26244896 | 0.05778524 | -2.183263951 | 1.63E-05 | 0.000163637 | PANK1 |
| ENSG00000067715 | 0.114228982 | 0.025106301 | -2.185807327 | 0.000887989 | 0.004918526 | SYT1 |
| ENSG00000138449 | 0.692912841 | 0.152104676 | -2.187609385 | 0.000753222 | 0.004300804 | SLC40A1 |
| ENSG00000153487 | 0.126841653 | 0.027835109 | -2.188050959 | 1.08E-13 | 5.14E-12 | ING1 |
| ENSG00000137815 | 0.148279809 | 0.032452027 | -2.191941642 | 0 | 0 | RTF1 |
| ENSG00000109670 | 0.129598679 | 0.028313159 | -2.194506383 | 1.53E-13 | 7.10E-12 | FBXW7 |
| ENSG00000106012 | 0.098472716 | 0.021426531 | -2.200325787 | 1.19E-08 | 2.50E-07 | IQCE |
| ENSG00000064042 | 0.146587218 | 0.031768583 | -2.206086644 | 0 | 0 | LIMCH1 |
| ENSG00000169981 | 0.046829702 | 0.010148686 | -2.206130833 | 5.61E-05 | 0.000480947 | ZNF35 |
| ENSG00000089177 | 0.149607197 | 0.032375807 | -2.208191506 | 6.59E-13 | 2.78E-11 | KIF16B |
| ENSG00000117395 | 0.240030166 | 0.05184116 | -2.211045823 | 2.79E-12 | 1.07E-10 | EBNA1BP2 |
| ENSG00000103264 | 0.040882673 | 0.008828536 | -2.211243462 | 5.42E-06 | 6.18E-05 | FBXO31 |
| ENSG00000096696 | 0.197316677 | 0.042415419 | -2.217852179 | 5.64E-07 | 8.32E-06 | DSP |
| ENSG00000078487 | 0.089708354 | 0.019269287 | -2.218939188 | 0.000682345 | 0.00397924 | ZCWPW1 |
| ENSG00000153113 | 0.285943475 | 0.061232731 | -2.223355059 | 0 | 0 | CAST |
| ENSG00000166402 | 0.042344134 | 0.009032825 | -2.228912992 | 0.001296781 | 0.006735979 | TUB |
| ENSG00000145734 | 0.10230696 | 0.021816336 | -2.229423577 | 4.04E-08 | 7.74E-07 | BDP1 |
| ENSG00000155592 | 0.030876692 | 0.00657532 | -2.231385094 | 0.000631016 | 0.00373976 | ZKSCAN2 |
| ENSG00000174799 | 0.160641742 | 0.034097576 | -2.236105736 | 0 | 0 | CEP135 |
| ENSG00000107537 | 0.088348159 | 0.018751961 | -2.236158622 | 3.90E-06 | 4.64E-05 | PHYH |
| ENSG00000141027 | 0.115237792 | 0.024432662 | -2.237730985 | 4.29E-07 | 6.49E-06 | NCOR1 |
| ENSG00000254064 | 0.36047837 | 0.076355892 | -2.239101298 | 0.001435946 | 0.007329056 | AC105206.2 |
| ENSG00000101773 | 0.091810624 | 0.019389159 | -2.243410859 | 0 | 0 | RBBP8 |
| ENSG00000197114 | 0.074978855 | 0.015834482 | -2.243414159 | 0.00028394 | 0.001914963 | ZGPAT |
| ENSG00000171951 | 0.168279748 | 0.035536073 | -2.243505407 | 6.22E-05 | 0.000528915 | SCG2 |
| ENSG00000138758 | 0.158582115 | 0.033449861 | -2.245157942 | 0 | 0 | 11-Sep |
| ENSG00000135090 | 0.208506738 | 0.043818381 | -2.250485912 | 4.28E-12 | 1.56E-10 | TAOK3 |
| ENSG00000117523 | 0.139064574 | 0.02921977 | -2.250738224 | 1.57E-07 | 2.63E-06 | PRRC2C |
| ENSG00000047410 | 0.250064968 | 0.052540244 | -2.250808159 | 4.51E-10 | 1.23E-08 | TPR |
| ENSG00000241935 | 0.266038797 | 0.055824595 | -2.252663863 | 0.00016775 | 0.001236024 | HOGA1 |
| ENSG00000130779 | 0.160272707 | 0.033603237 | -2.253856671 | 0 | 0 | CLIP1 |
| ENSG00000165525 | 0.17710997 | 0.037101084 | -2.255112168 | 7.98E-14 | 3.84E-12 | NEMF |
| ENSG00000139132 | 0.197086179 | 0.041230912 | -2.257028323 | 0.000659935 | 0.003880548 | FGD4 |
| ENSG00000123095 | 0.098632934 | 0.0205254 | -2.264659079 | 0.000748703 | 0.00427703 | BHLHE41 |
| ENSG00000222365 | 65.6744554 | 13.59153307 | -2.272624137 | 0.001565169 | 0.007865088 | SNORD12B |
| ENSG00000126777 | 0.134985097 | 0.027848397 | -2.277133943 | 0 | 0 | KTN1 |
| ENSG00000204138 | 0.160378071 | 0.033067108 | -2.278008103 | 0 | 0 | PHACTR4 |
| ENSG00000151461 | 0.168466338 | 0.03472516 | -2.278407107 | 0 | 0 | UPF2 |
| ENSG00000136936 | 0.186201333 | 0.03831987 | -2.280698813 | 7.18E-08 | 1.29E-06 | XPA |
| ENSG00000138380 | 0.062853355 | 0.01291986 | -2.282399311 | 0.000696534 | 0.004038489 | CARF |
| ENSG00000137877 | 0.142652622 | 0.029260102 | -2.285499548 | 0.000103964 | 0.00082248 | SPTBN5 |
| ENSG00000100629 | 0.200672447 | 0.040718567 | -2.301083837 | 9.05E-09 | 1.94E-07 | CEP128 |
| ENSG00000181982 | 0.112940541 | 0.022906539 | -2.301732033 | 0.000313191 | 0.002077288 | CCDC149 |
| ENSG00000138134 | 0.035882936 | 0.007209129 | -2.315401139 | 0.000431042 | 0.002705261 | STAMBPL1 |
| ENSG00000119242 | 0.071906057 | 0.014442857 | -2.315757142 | 0 | 0 | CCDC92 |
| ENSG00000120051 | 0.14570505 | 0.029213309 | -2.318353215 | 0.000153515 | 0.001146594 | CFAP58 |
| ENSG00000167987 | 0.019679839 | 0.003935302 | -2.322172062 | 2.62E-06 | 3.28E-05 | VPS37C |
| ENSG00000121851 | 0.305439915 | 0.061041569 | -2.323024666 | 3.69E-05 | 0.000334445 | POLR3GL |
| ENSG00000166348 | 0.119991422 | 0.023951927 | -2.32471761 | 1.90E-05 | 0.000187667 | USP54 |
| ENSG00000101104 | 0.068598523 | 0.013679943 | -2.326115316 | 7.55E-05 | 0.000624033 | PABPC1L |
| ENSG00000101343 | 0.19725655 | 0.039297589 | -2.327560497 | 0 | 0 | CRNKL1 |
| ENSG00000088038 | 0.182120906 | 0.036238472 | -2.329302519 | 1.64E-09 | 4.01E-08 | CNOT3 |
| ENSG00000123200 | 0.232213079 | 0.046182353 | -2.330035643 | 0 | 0 | ZC3H13 |
| ENSG00000104218 | 0.326956355 | 0.064715626 | -2.336912065 | 0 | 0 | CSPP1 |
| ENSG00000142494 | 0.126559115 | 0.025012926 | -2.339065703 | 7.96E-05 | 0.000654647 | SLC47A1 |
| ENSG00000167711 | 0.806274793 | 0.158633388 | -2.345575169 | 0.000207494 | 0.001470311 | SERPINF2 |
| ENSG00000158691 | 0.08744387 | 0.017097052 | -2.354609664 | 8.47E-08 | 1.50E-06 | ZSCAN12 |
| ENSG00000125352 | 0.133563504 | 0.026074695 | -2.356803579 | 1.58E-05 | 0.000159287 | RNF113A |
| ENSG00000017797 | 0.129923022 | 0.025343845 | -2.357949799 | 0 | 0 | RALBP1 |
| ENSG00000188906 | 0.19830302 | 0.038375345 | -2.369455022 | 0.00024932 | 0.00172402 | LRRK2 |
| ENSG00000230606 | 0.193585455 | 0.037403515 | -2.371724822 | 0.000226165 | 0.00158672 | AC092683.1 |
| ENSG00000109762 | 0.102139464 | 0.019612526 | -2.380693144 | 1.88E-06 | 2.45E-05 | SNX25 |
| ENSG00000251348 | 0.102487421 | 0.019594139 | -2.386952794 | 0.00067288 | 0.003931675 | HSPD1P11 |
| ENSG00000140416 | 0.384010679 | 0.073399629 | -2.387301754 | 6.66E-16 | 4.24E-14 | TPM1 |
| ENSG00000103061 | 0.149179941 | 0.028408152 | -2.392676676 | 2.50E-06 | 3.17E-05 | SLC7A6OS |
| ENSG00000150281 | 0.066969404 | 0.012729404 | -2.395337318 | 2.95E-05 | 0.000278777 | CTF1 |
| ENSG00000282826 | 0.026669294 | 0.00505976 | -2.398038625 | 0.00108129 | 0.005805265 | FRG1CP |
| ENSG00000134046 | 0.19135525 | 0.036239447 | -2.400620652 | 1.27E-13 | 5.97E-12 | MBD2 |
| ENSG00000067533 | 0.244302496 | 0.046038406 | -2.407758803 | 2.22E-16 | 1.49E-14 | RRP15 |
| ENSG00000272333 | 0.065168488 | 0.01221408 | -2.415629356 | 4.35E-08 | 8.28E-07 | KMT2B |
| ENSG00000187955 | 0.177490954 | 0.033233073 | -2.417053897 | 1.81E-05 | 0.000180078 | COL14A1 |
| ENSG00000119397 | 0.184093822 | 0.034360864 | -2.421602998 | 9.21E-10 | 2.36E-08 | CNTRL |
| ENSG00000175048 | 0.042569112 | 0.007936531 | -2.423226574 | 0.000565611 | 0.003411094 | ZDHHC14 |
| ENSG00000164167 | 0.241150801 | 0.04495305 | -2.423444691 | 1.98E-07 | 3.22E-06 | LSM6 |
| ENSG00000169554 | 0.116299239 | 0.021665015 | -2.424402502 | 0 | 0 | ZEB2 |
| ENSG00000168724 | 0.161053613 | 0.029629733 | -2.442423476 | 0 | 0 | DNAJC21 |
| ENSG00000151466 | 0.156130217 | 0.028716043 | -2.442820912 | 1.74E-08 | 3.55E-07 | SCLT1 |
| ENSG00000101298 | 0.020446277 | 0.00375427 | -2.445233912 | 6.86E-05 | 0.000572001 | SNPH |
| ENSG00000144891 | 0.565970464 | 0.103812358 | -2.446748565 | 0.00015399 | 0.001148718 | AGTR1 |
| ENSG00000168495 | 0.196318191 | 0.035962663 | -2.448622097 | 0 | 0 | POLR3D |
| ENSG00000114446 | 0.316103527 | 0.057857362 | -2.449824684 | 7.22E-15 | 4.04E-13 | IFT57 |
| ENSG00000244405 | 0.101102441 | 0.018419543 | -2.456508638 | 1.08E-07 | 1.86E-06 | ETV5 |
| ENSG00000009954 | 0.189007482 | 0.03425294 | -2.464143594 | 0 | 0 | BAZ1B |
| ENSG00000141867 | 0.239283635 | 0.043251606 | -2.467896115 | 5.11E-15 | 2.92E-13 | BRD4 |
| ENSG00000141622 | 0.092751431 | 0.016683854 | -2.474916951 | 0.00182286 | 0.008935953 | RNF165 |
| ENSG00000089682 | 0.099320414 | 0.017837957 | -2.47713992 | 7.19E-05 | 0.000595903 | RBM41 |
| ENSG00000233045 | 0.627145103 | 0.111659349 | -2.489695227 | 8.61E-05 | 0.00069879 | AC097523.1 |
| ENSG00000165632 | 0.109595186 | 0.01930857 | -2.504871226 | 5.76E-14 | 2.82E-12 | TAF3 |
| ENSG00000164609 | 0.196660976 | 0.034625325 | -2.505811191 | 0 | 0 | SLU7 |
| ENSG00000187951 | 0.013706184 | 0.002409608 | -2.507956479 | 0.000672085 | 0.003928936 | AC091057.1 |
| ENSG00000138180 | 0.423936297 | 0.074253727 | -2.513312154 | 0 | 0 | CEP55 |
| ENSG00000242689 | 0.130011168 | 0.022726574 | -2.516183424 | 0.000215436 | 0.001521211 | CNTF |
| ENSG00000236609 | 0.05386542 | 0.009405391 | -2.517799629 | 6.65E-06 | 7.39E-05 | ZNF853 |
| ENSG00000111275 | 0.184730127 | 0.03220902 | -2.519882509 | 1.88E-07 | 3.09E-06 | ALDH2 |
| ENSG00000099822 | 0.079223639 | 0.013696672 | -2.532105533 | 1.91E-05 | 0.000188368 | HCN2 |
| ENSG00000170949 | 0.136099587 | 0.023478322 | -2.535261505 | 9.94E-10 | 2.53E-08 | ZNF160 |
| ENSG00000155100 | 0.236049662 | 0.040548162 | -2.541381985 | 0 | 0 | OTUD6B |
| ENSG00000174720 | 0.17000086 | 0.029122264 | -2.545347632 | 1.07E-10 | 3.24E-09 | LARP7 |
| ENSG00000225921 | 0.286295777 | 0.048861771 | -2.550728316 | 7.77E-16 | 4.92E-14 | NOL7 |
| ENSG00000222489 | 4.283185461 | 0.727608708 | -2.557449432 | 0.000360882 | 0.002330618 | SNORA79B |
| ENSG00000174446 | 0.036013761 | 0.006089952 | -2.564045588 | 3.04E-05 | 0.000285094 | SNAPC5 |
| ENSG00000131773 | 0.137087436 | 0.023154247 | -2.565747611 | 9.43E-08 | 1.65E-06 | KHDRBS3 |
| ENSG00000221539 | 29.93053024 | 5.010555047 | -2.578575505 | 4.93E-06 | 5.66E-05 | SNORD99 |
| ENSG00000129680 | 0.239910672 | 0.039791461 | -2.591966568 | 0 | 0 | MAP7D3 |
| ENSG00000117533 | 0.027773997 | 0.004582791 | -2.599436273 | 0.000121774 | 0.000941694 | VAMP4 |
| ENSG00000264290 | 0.83852558 | 0.137910157 | -2.604126086 | 0.000425783 | 0.002682054 | AC104564.3 |
| ENSG00000171094 | 0.426784248 | 0.069995153 | -2.608180009 | 0.000128655 | 0.000986659 | ALK |
| ENSG00000140367 | 0.093951596 | 0.01540583 | -2.608441227 | 0 | 0 | UBE2Q2 |
| ENSG00000125977 | 0.372206498 | 0.060958581 | -2.610202014 | 0 | 0 | EIF2S2 |
| ENSG00000124383 | 0.201959408 | 0.033011345 | -2.613031542 | 0 | 0 | MPHOSPH10 |
| ENSG00000069667 | 0.266191889 | 0.043436103 | -2.615500021 | 2.62E-05 | 0.000250082 | RORA |
| ENSG00000118181 | 0.248858737 | 0.040430976 | -2.621794086 | 8.37E-14 | 4.01E-12 | RPS25 |
| ENSG00000169925 | 0.152249751 | 0.024715886 | -2.622929311 | 4.32E-10 | 1.18E-08 | BRD3 |
| ENSG00000148773 | 0.150644949 | 0.024416541 | -2.625221561 | 1.03E-08 | 2.18E-07 | MKI67 |
| ENSG00000200913 | 18.8843507 | 3.059711736 | -2.625723537 | 0.000509059 | 0.003116823 | SNORD46 |
| ENSG00000240370 | 0.155407575 | 0.025156049 | -2.627079586 | 3.66E-05 | 0.000331979 | RPL13P5 |
| ENSG00000115457 | 0.121652655 | 0.019672987 | -2.628479893 | 0.000608982 | 0.003627049 | IGFBP2 |
| ENSG00000267216 | 0.157474369 | 0.025380862 | -2.633304049 | 1.33E-05 | 0.000137149 | AC020915.1 |
| ENSG00000135315 | 0.285813877 | 0.044635729 | -2.67880508 | 6.66E-16 | 4.24E-14 | CEP162 |
| ENSG00000126218 | 0.479504762 | 0.074809787 | -2.680246207 | 4.17E-05 | 0.000373179 | F10 |
| ENSG00000188559 | 0.097923774 | 0.015194099 | -2.688148082 | 1.09E-06 | 1.51E-05 | RALGAPA2 |
| ENSG00000168280 | 0.251136111 | 0.038957844 | -2.688483739 | 5.79E-05 | 0.000496009 | KIF5C |
| ENSG00000137776 | 0.315382547 | 0.048782964 | -2.692653504 | 0 | 0 | SLTM |
| ENSG00000166004 | 0.17736272 | 0.027381765 | -2.695415447 | 0.000663008 | 0.003892906 | CEP295 |
| ENSG00000133226 | 0.180426018 | 0.027446595 | -2.716708329 | 0 | 0 | SRRM1 |
| ENSG00000160439 | 0.079616147 | 0.012083064 | -2.720074682 | 3.11E-06 | 3.81E-05 | RDH13 |
| ENSG00000189056 | 1.070119505 | 0.161284259 | -2.730094373 | 0.000248029 | 0.001716078 | RELN |
| ENSG00000106633 | 1.839465267 | 0.275875318 | -2.73719814 | 0.001178281 | 0.006228057 | GCK |
| ENSG00000165733 | 0.261348448 | 0.039101998 | -2.740660345 | 0 | 0 | BMS1 |
| ENSG00000236130 | 1.157608877 | 0.172711108 | -2.74471511 | 0.000859632 | 0.004787902 | PTCSC2 |
| ENSG00000136819 | 0.249055465 | 0.037067439 | -2.748242714 | 4.44E-15 | 2.57E-13 | C9orf78 |
| ENSG00000147408 | 0.203955051 | 0.030216943 | -2.754821604 | 4.00E-06 | 4.74E-05 | CSGALNACT1 |
| ENSG00000148798 | 1.35914897 | 0.198985497 | -2.771968405 | 2.24E-10 | 6.44E-09 | INA |
| ENSG00000076555 | 0.147620796 | 0.021606318 | -2.772370841 | 0.000198066 | 0.001416862 | ACACB |
| ENSG00000169031 | 1.380677443 | 0.201985285 | -2.773054212 | 0.000953464 | 0.005221043 | COL4A3 |
| ENSG00000147124 | 0.03017833 | 0.004388535 | -2.781701778 | 0.001842679 | 0.008992784 | ZNF41 |
| ENSG00000185761 | 0.240577625 | 0.034663421 | -2.795016505 | 0.000438215 | 0.002741695 | ADAMTSL5 |
| ENSG00000207445 | 63.42275538 | 9.129178768 | -2.796443565 | 1.58E-05 | 0.000158851 | SNORD15B |
| ENSG00000179104 | 0.475900726 | 0.068453463 | -2.797465222 | 0.00022126 | 0.001556849 | TMTC2 |
| ENSG00000116761 | 0.114919407 | 0.016483363 | -2.801539924 | 0.001876329 | 0.009119992 | CTH |
| ENSG00000114670 | 0.108265932 | 0.01549401 | -2.804796868 | 2.47E-06 | 3.13E-05 | NEK11 |
| ENSG00000230453 | 0.53908985 | 0.07686552 | -2.81011725 | 2.11E-07 | 3.40E-06 | ANKRD18B |
| ENSG00000185345 | 1.776808212 | 0.251456713 | -2.82090599 | 0.000726381 | 0.004177299 | PRKN |
| ENSG00000162520 | 0.072944884 | 0.010251158 | -2.83101984 | 0.00034545 | 0.002250292 | SYNC |
| ENSG00000163001 | 0.152918488 | 0.021026712 | -2.862467679 | 3.33E-16 | 2.21E-14 | CFAP36 |
| ENSG00000158321 | 0.525800392 | 0.072077611 | -2.866892116 | 0.000524666 | 0.003200966 | AUTS2 |
| ENSG00000162849 | 1.389147759 | 0.188456248 | -2.881898528 | 0.001869636 | 0.009094809 | KIF26B |
| ENSG00000223396 | 0.30307994 | 0.041040755 | -2.884569176 | 0.001037725 | 0.005595806 | RPS10P7 |
| ENSG00000100311 | 0.034475598 | 0.004657383 | -2.8879841 | 0.000169276 | 0.001245746 | PDGFB |
| ENSG00000255529 | 0.011481714 | 0.001549466 | -2.889494554 | 0.000556307 | 0.003366677 | POLR2M |
| ENSG00000201772 | 1.346553297 | 0.179068462 | -2.91068816 | 0.000632458 | 0.003746456 | SNORA5C |
| ENSG00000182578 | 0.903653186 | 0.119611873 | -2.917408587 | 6.70E-05 | 0.000561161 | CSF1R |
| ENSG00000058335 | 0.279703553 | 0.036735175 | -2.928664516 | 4.55E-06 | 5.30E-05 | RASGRF1 |
| ENSG00000121691 | 0.306195337 | 0.039863634 | -2.941307182 | 7.31E-10 | 1.92E-08 | CAT |
| ENSG00000176771 | 0.132078685 | 0.017181368 | -2.942480811 | 1.25E-07 | 2.12E-06 | NCKAP5 |
| ENSG00000270231 | 0.011287209 | 0.001465393 | -2.945329685 | 0.001839286 | 0.008983517 | NBPF8 |
| ENSG00000234545 | 0.10155369 | 0.013161053 | -2.947895875 | 4.67E-09 | 1.05E-07 | FAM133B |
| ENSG00000207280 | 436.7930108 | 54.38281211 | -3.005727112 | 0.000192052 | 0.001384546 | SNORD20 |
| ENSG00000204103 | 0.162244259 | 0.019990256 | -3.020798602 | 5.60E-08 | 1.03E-06 | MAFB |
| ENSG00000132386 | 0.100518916 | 0.012353049 | -3.024527903 | 7.63E-06 | 8.35E-05 | SERPINF1 |
| ENSG00000183044 | 0.45689739 | 0.055754035 | -3.034722073 | 0.000461392 | 0.002869635 | ABAT |
| ENSG00000201457 | 35.62422636 | 4.342526861 | -3.036252006 | 1.68E-08 | 3.44E-07 | SNORA55 |
| ENSG00000074181 | 0.420697666 | 0.051257107 | -3.03695986 | 2.05E-05 | 0.000199856 | NOTCH3 |
| ENSG00000184838 | 0.195054477 | 0.023728954 | -3.03915669 | 0.000268336 | 0.001833375 | PRR16 |
| ENSG00000164610 | 0.140075349 | 0.017008417 | -3.041882267 | 1.16E-13 | 5.49E-12 | RP9 |
| ENSG00000173208 | 1.795692411 | 0.217436778 | -3.04587236 | 0.000894986 | 0.004945256 | ABCD2 |
| ENSG00000266852 | 0.755796843 | 0.090801898 | -3.057204132 | 6.49E-05 | 0.000548938 | MIR4482 |
| ENSG00000064199 | 0.165554967 | 0.019875078 | -3.058277877 | 0.001428568 | 0.007303799 | SPA17 |
| ENSG00000129596 | 0.805747502 | 0.09643749 | -3.062661799 | 8.41E-09 | 1.81E-07 | CDO1 |
| ENSG00000006459 | 0.125122654 | 0.01494431 | -3.065674863 | 0.000237129 | 0.001652069 | KDM7A |
| ENSG00000110888 | 0.036353483 | 0.004310481 | -3.076172727 | 3.37E-08 | 6.56E-07 | CAPRIN2 |
| ENSG00000005075 | 0.005830384 | 0.000688748 | -3.081542745 | 0.000961184 | 0.005251355 | POLR2J |
| ENSG00000091428 | 1.481611503 | 0.174394347 | -3.086742024 | 0.000123483 | 0.000953683 | RAPGEF4 |
| ENSG00000275131 | 0.041655368 | 0.004891035 | -3.090290863 | 0.000226588 | 0.001588756 | AC241952.1 |
| ENSG00000145703 | 1.65509764 | 0.193687921 | -3.09511044 | 4.64E-05 | 0.000407573 | IQGAP2 |
| ENSG00000162409 | 0.142294153 | 0.016616812 | -3.098160873 | 3.06E-05 | 0.000286188 | PRKAA2 |
| ENSG00000070729 | 1.74684219 | 0.203102512 | -3.104469291 | 0.00058714 | 0.003521374 | CNGB1 |
| ENSG00000204991 | 0.042521834 | 0.004893429 | -3.119286114 | 1.18E-05 | 0.000123612 | SPIRE2 |
| ENSG00000023839 | 0.29245481 | 0.033033966 | -3.146191607 | 1.14E-06 | 1.57E-05 | ABCC2 |
| ENSG00000165275 | 0.041204322 | 0.004636118 | -3.15180646 | 4.21E-05 | 0.000375933 | TRMT10B |
| ENSG00000112293 | 1.266875294 | 0.142154084 | -3.155747069 | 2.52E-05 | 0.000241468 | GPLD1 |
| ENSG00000204524 | 0.0791196 | 0.008849154 | -3.160423761 | 0.001545219 | 0.007783434 | ZNF805 |
| ENSG00000214530 | 0.14504876 | 0.016195792 | -3.162847005 | 9.21E-10 | 2.36E-08 | STARD10 |
| ENSG00000197779 | 0.064071545 | 0.007047671 | -3.184465223 | 0.000481742 | 0.002973177 | ZNF81 |
| ENSG00000237437 | 1.566240076 | 0.171800082 | -3.188502738 | 3.85E-06 | 4.59E-05 | ASS1P12 |
| ENSG00000174501 | 0.107235685 | 0.01168316 | -3.198282663 | 0.001516178 | 0.007654091 | ANKRD36C |
| ENSG00000185485 | 0.028489343 | 0.003091177 | -3.20419418 | 3.26E-05 | 0.000301396 | SDHAP1 |
| ENSG00000069535 | 3.074211333 | 0.333053177 | -3.206391898 | 0.000196421 | 0.001409283 | MAOB |
| ENSG00000200087 | 45.27143624 | 4.87082493 | -3.216363042 | 2.15E-14 | 1.12E-12 | SNORA73B |
| ENSG00000226210 | 0.017601847 | 0.001883651 | -3.224123002 | 0.001841945 | 0.008992784 | WASH8P |
| ENSG00000178445 | 0.505044582 | 0.053872021 | -3.22880265 | 1.95E-06 | 2.54E-05 | GLDC |
| ENSG00000133739 | 0.092929595 | 0.009891795 | -3.23183393 | 0.001322104 | 0.00684092 | LRRCC1 |
| ENSG00000006468 | 0.459658589 | 0.047793026 | -3.265690677 | 0.000625601 | 0.0037139 | ETV1 |
| ENSG00000130208 | 2.241566464 | 0.232476846 | -3.269348334 | 0.000603716 | 0.003599247 | APOC1 |
| ENSG00000140465 | 0.261865479 | 0.027119289 | -3.271434612 | 6.71E-06 | 7.44E-05 | CYP1A1 |
| ENSG00000212135 | 38.02874668 | 3.932705876 | -3.273496195 | 1.08E-05 | 0.000114875 | SNORD67 |
| ENSG00000219451 | 1.161812112 | 0.119997479 | -3.27530077 | 0.000110529 | 0.000865869 | RPL23P8 |
| ENSG00000081803 | 0.693065036 | 0.071158043 | -3.283892005 | 0.000239044 | 0.001664451 | CADPS2 |
| ENSG00000073350 | 0.045299112 | 0.004637589 | -3.288036015 | 0.000373808 | 0.002403768 | LLGL2 |
| ENSG00000162614 | 0.587724245 | 0.060059398 | -3.290677502 | 0 | 0 | NEXN |
| ENSG00000238363 | 308.1337916 | 31.39115772 | -3.295126762 | 0.000164724 | 0.001218248 | SNORA13 |
| ENSG00000151150 | 1.767825798 | 0.179440366 | -3.300399743 | 0.000378581 | 0.002428141 | ANK3 |
| ENSG00000182985 | 1.852465232 | 0.187617293 | -3.30358175 | 7.01E-05 | 0.000583335 | CADM1 |
| ENSG00000135338 | 0.096163997 | 0.009733165 | -3.304515973 | 9.25E-05 | 0.000743145 | LCA5 |
| ENSG00000132330 | 0.152747854 | 0.015432044 | -3.307151018 | 0.000137568 | 0.001042382 | SCLY |
| ENSG00000157890 | 0.43852381 | 0.043889648 | -3.320702561 | 0.00066617 | 0.003905753 | MEGF11 |
| ENSG00000183199 | 0.234149997 | 0.023406023 | -3.322481284 | 3.67E-07 | 5.64E-06 | HSP90AB3P |
| ENSG00000178401 | 0.063745298 | 0.006324306 | -3.333339787 | 0.001510916 | 0.00763073 | DNAJC22 |
| ENSG00000207973 | 13.62163273 | 1.347099618 | -3.337971193 | 8.56E-07 | 1.21E-05 | MIR589 |
| ENSG00000164989 | 0.287403434 | 0.028419703 | -3.338113895 | 2.73E-08 | 5.40E-07 | CCDC171 |
| ENSG00000145241 | 0.054198282 | 0.005323878 | -3.347697776 | 1.05E-10 | 3.17E-09 | CENPC |
| ENSG00000235408 | 96.73194334 | 9.463113346 | -3.353605572 | 5.94E-12 | 2.10E-10 | SNORA71B |
| ENSG00000143507 | 0.096767548 | 0.009381374 | -3.366652196 | 3.49E-06 | 4.21E-05 | DUSP10 |
| ENSG00000170873 | 0.317793369 | 0.030724069 | -3.370647835 | 3.14E-06 | 3.85E-05 | MTSS1 |
| ENSG00000143847 | 0.068816695 | 0.006590369 | -3.384327397 | 0.000296511 | 0.00198196 | PPFIA4 |
| ENSG00000207757 | 581.787277 | 55.36155547 | -3.393535363 | 0.000666616 | 0.00390646 | MIR93 |
| ENSG00000110693 | 0.964592786 | 0.090804174 | -3.409089506 | 4.76E-06 | 5.50E-05 | SOX6 |
| ENSG00000184208 | 0.021815134 | 0.002045591 | -3.414739793 | 5.40E-07 | 8.02E-06 | C22orf46 |
| ENSG00000196405 | 0.10725389 | 0.009979554 | -3.425910806 | 1.47E-14 | 7.76E-13 | EVL |
| ENSG00000057704 | 0.070989106 | 0.006545579 | -3.439004864 | 2.37E-05 | 0.000228163 | TMCC3 |
| ENSG00000112679 | 0.023311847 | 0.00211835 | -3.460050209 | 5.80E-05 | 0.000496009 | DUSP22 |
| ENSG00000105327 | 0.039815975 | 0.003616795 | -3.460563635 | 0.000944257 | 0.005177699 | BBC3 |
| ENSG00000213071 | 0.958256432 | 0.085949522 | -3.478850255 | 4.76E-06 | 5.50E-05 | LPAL2 |
| ENSG00000120833 | 0.034757009 | 0.003117445 | -3.478867818 | 0.000269885 | 0.001840758 | SOCS2 |
| ENSG00000259726 | 1.529750403 | 0.136894021 | -3.482164938 | 6.71E-05 | 0.000561898 | CSPG4P11 |
| ENSG00000139344 | 0.524487364 | 0.04689883 | -3.483284186 | 6.24E-09 | 1.38E-07 | AMDHD1 |
| ENSG00000136379 | 0.038673332 | 0.003446206 | -3.488258045 | 6.11E-06 | 6.87E-05 | ABHD17C |
| ENSG00000256238 | 1.869894061 | 0.165627616 | -3.496941391 | 0.000172293 | 0.00126408 | SUPT16HP1 |
| ENSG00000264049 | 6.743546246 | 0.589881564 | -3.51501024 | 0.000131711 | 0.001003688 | MIR4737 |
| ENSG00000237054 | 2.519164549 | 0.218714418 | -3.525825129 | 0.000131548 | 0.001003085 | PRMT5-AS1 |
| ENSG00000284906 | 0.091285566 | 0.007869962 | -3.535958218 | 0.001792059 | 0.008806503 | AC091057.6 |
| ENSG00000176024 | 0.10953991 | 0.00943094 | -3.537911295 | 1.39E-05 | 0.000142237 | ZNF613 |
| ENSG00000224083 | 3.480719502 | 0.298104778 | -3.545494155 | 0.001927564 | 0.009313157 | MTCO1P11 |
| ENSG00000128833 | 0.091641916 | 0.007833428 | -3.548291981 | 1.25E-06 | 1.70E-05 | MYO5C |
| ENSG00000178038 | 0.046273319 | 0.003832932 | -3.593660251 | 0.000604703 | 0.003603346 | ALS2CL |
| ENSG00000118514 | 1.580062166 | 0.130599202 | -3.596763338 | 4.61E-06 | 5.35E-05 | ALDH8A1 |
| ENSG00000134899 | 0.086368136 | 0.007105963 | -3.603397069 | 0.001401338 | 0.007192597 | ERCC5 |
| ENSG00000137474 | 1.38483498 | 0.113714092 | -3.606231116 | 5.81E-06 | 6.58E-05 | MYO7A |
| ENSG00000095637 | 0.631698901 | 0.051399362 | -3.619414717 | 3.15E-07 | 4.92E-06 | SORBS1 |
| ENSG00000137561 | 2.666828916 | 0.21616538 | -3.624917881 | 3.57E-06 | 4.30E-05 | TTPA |
| ENSG00000278932 | 84.36870459 | 6.796256094 | -3.633895829 | 0.00186459 | 0.009073934 | CR381653.1 |
| ENSG00000100024 | 1.894691362 | 0.150863258 | -3.650649469 | 1.01E-05 | 0.000107734 | UPB1 |
| ENSG00000123977 | 0.201380028 | 0.01591742 | -3.66124215 | 0.001324504 | 0.006847445 | DAW1 |
| ENSG00000170439 | 1.830864443 | 0.144378531 | -3.664596845 | 1.22E-05 | 0.000127099 | METTL7B |
| ENSG00000099937 | 2.104535091 | 0.165002882 | -3.672938435 | 1.15E-05 | 0.000120757 | SERPIND1 |
| ENSG00000183060 | 0.028161761 | 0.002203854 | -3.675636905 | 0.000298323 | 0.001991858 | LYSMD4 |
| ENSG00000245848 | 3.831951747 | 0.295736288 | -3.695696213 | 0.000616819 | 0.003666459 | CEBPA |
| ENSG00000259921 | 10.74447574 | 0.821561995 | -3.709081835 | 0.000415707 | 0.00263099 | AC022819.1 |
| ENSG00000215883 | 0.105835697 | 0.007993309 | -3.726889638 | 2.13E-09 | 5.10E-08 | CYB5RL |
| ENSG00000168306 | 0.603858601 | 0.045301048 | -3.736594434 | 7.26E-05 | 0.000601468 | ACOX2 |
| ENSG00000153157 | 0.161206584 | 0.011904923 | -3.759280436 | 0.001006774 | 0.005459678 | SYCP2L |
| ENSG00000273136 | 0.038646779 | 0.002842067 | -3.765335922 | 1.90E-05 | 0.000187667 | NBPF26 |
| ENSG00000109063 | 0.516949025 | 0.037622428 | -3.780357161 | 1.85E-10 | 5.39E-09 | MYH3 |
| ENSG00000168702 | 1.672893274 | 0.12132607 | -3.785383924 | 0.000167736 | 0.001236024 | LRP1B |
| ENSG00000196912 | 0.167341813 | 0.012084785 | -3.79153425 | 2.99E-06 | 3.68E-05 | ANKRD36B |
| ENSG00000112167 | 0.019945724 | 0.001437995 | -3.793949328 | 0.00120274 | 0.006326747 | SAYSD1 |
| ENSG00000121101 | 0.438194207 | 0.031214558 | -3.811279464 | 0.001229427 | 0.006447389 | TEX14 |
| ENSG00000207523 | 1008.093134 | 71.78102572 | -3.811882581 | 9.08E-06 | 9.76E-05 | SNORA66 |
| ENSG00000025423 | 0.614169775 | 0.043682936 | -3.813495801 | 4.20E-09 | 9.56E-08 | HSD17B6 |
| ENSG00000130707 | 0.29232098 | 0.020326278 | -3.846135387 | 8.04E-13 | 3.33E-11 | ASS1 |
| ENSG00000109758 | 3.326939684 | 0.231028979 | -3.84804998 | 0.000106481 | 0.000837435 | HGFAC |
| ENSG00000188856 | 0.01634367 | 0.001133311 | -3.850115912 | 0.000104273 | 0.000823841 | RPSAP47 |
| ENSG00000120915 | 2.406236888 | 0.164262254 | -3.872705775 | 2.50E-05 | 0.00023913 | EPHX2 |
| ENSG00000141338 | 3.169775902 | 0.214904516 | -3.882613145 | 1.96E-05 | 0.000191753 | ABCA8 |
| ENSG00000102931 | 0.856530995 | 0.057676502 | -3.892449878 | 3.92E-06 | 4.66E-05 | ARL2BP |
| ENSG00000224699 | 0.13298216 | 0.00895016 | -3.893175422 | 4.69E-05 | 0.00041174 | LAMTOR5-AS1 |
| ENSG00000213398 | 0.085987198 | 0.005736233 | -3.905946395 | 1.70E-06 | 2.25E-05 | LCAT |
| ENSG00000084734 | 2.181440626 | 0.143814966 | -3.922995481 | 1.10E-05 | 0.000116062 | GCKR |
| ENSG00000141965 | 0.076395887 | 0.004892104 | -3.964967992 | 0.001106384 | 0.005926176 | FEM1A |
| ENSG00000154262 | 2.666085953 | 0.169029614 | -3.979375361 | 0.000405466 | 0.002579753 | ABCA6 |
| ENSG00000229980 | 1.423005577 | 0.087455309 | -4.024251538 | 8.39E-05 | 0.000683727 | TOB1-AS1 |
| ENSG00000143195 | 0.189995823 | 0.011651268 | -4.027408803 | 0.000311435 | 0.002066782 | ILDR2 |
| ENSG00000147573 | 4.487936786 | 0.271730416 | -4.045804391 | 0.001136029 | 0.006047255 | TRIM55 |
| ENSG00000199805 | 10.21890103 | 0.611276753 | -4.06327054 | 4.59E-05 | 0.000404774 | RNU1-134P |
| ENSG00000175711 | 0.059219178 | 0.003524237 | -4.070681666 | 0.000141092 | 0.001065056 | B3GNTL1 |
| ENSG00000140538 | 4.291616773 | 0.254942556 | -4.073277136 | 2.52E-06 | 3.18E-05 | NTRK3 |
| ENSG00000178573 | 0.869435958 | 0.05152187 | -4.076822891 | 4.60E-05 | 0.000405052 | MAF |
| ENSG00000237118 | 1.509368054 | 0.088202325 | -4.096984157 | 8.80E-08 | 1.55E-06 | CYP2F2P |
| ENSG00000234585 | 0.165903277 | 0.009689308 | -4.097804981 | 8.71E-05 | 0.00070565 | CCT6P3 |
| ENSG00000100918 | 0.033675839 | 0.001959214 | -4.103366919 | 0.000413101 | 0.002620009 | REC8 |
| ENSG00000203814 | 120.9360971 | 7.025605606 | -4.105478525 | 1.74E-09 | 4.25E-08 | HIST2H2BF |
| ENSG00000172264 | 0.221182283 | 0.012776841 | -4.113632733 | 0.000162529 | 0.001205682 | MACROD2 |
| ENSG00000114541 | 0.131720273 | 0.007558254 | -4.123280613 | 0.00010419 | 0.000823728 | FRMD4B |
| ENSG00000073584 | 0.071195834 | 0.004035281 | -4.141051639 | 1.01E-06 | 1.40E-05 | SMARCE1 |
| ENSG00000109944 | 0.051753894 | 0.002883328 | -4.165860751 | 4.18E-05 | 0.00037382 | JHY |
| ENSG00000135423 | 2.932968405 | 0.160006559 | -4.19615858 | 2.65E-07 | 4.19E-06 | GLS2 |
| ENSG00000039139 | 0.140692666 | 0.007591092 | -4.212095914 | 6.48E-05 | 0.000548567 | DNAH5 |
| ENSG00000167588 | 0.651043588 | 0.034967864 | -4.218652559 | 0.000230644 | 0.001612498 | GPD1 |
| ENSG00000149131 | 6.181599666 | 0.330079496 | -4.227094797 | 2.74E-05 | 0.000259547 | SERPING1 |
| ENSG00000206073 | 0.116113073 | 0.006192863 | -4.22877998 | 0.0001629 | 0.001206199 | SERPINB4 |
| ENSG00000182568 | 0.124589897 | 0.006625117 | -4.233097375 | 1.66E-05 | 0.000166019 | SATB1 |
| ENSG00000117425 | 0.678229051 | 0.035533508 | -4.254520534 | 2.95E-10 | 8.33E-09 | PTCH2 |
| ENSG00000228634 | 8.331993685 | 0.425714364 | -4.290704074 | 0.001234703 | 0.006472233 | AL136115.1 |
| ENSG00000172425 | 4.95238338 | 0.251479984 | -4.299607523 | 0.000378608 | 0.002428141 | TTC36 |
| ENSG00000188338 | 4.036192609 | 0.201659045 | -4.323005001 | 7.29E-12 | 2.57E-10 | SLC38A3 |
| ENSG00000143867 | 0.028879242 | 0.001437441 | -4.328458437 | 3.90E-05 | 0.000351038 | OSR1 |
| ENSG00000143416 | 0.829418811 | 0.040951547 | -4.340110902 | 1.40E-06 | 1.88E-05 | SELENBP1 |
| ENSG00000225031 | 0.146089062 | 0.006914035 | -4.401176371 | 0.000859152 | 0.004787441 | EIF4BP7 |
| ENSG00000215182 | 5.367329754 | 0.249769382 | -4.425535988 | 4.81E-05 | 0.000420746 | MUC5AC |
| ENSG00000257956 | 4.944686833 | 0.228177102 | -4.437653224 | 0.000271971 | 0.00185016 | NOP56P3 |
| ENSG00000221994 | 0.243023623 | 0.011154331 | -4.445420671 | 4.43E-05 | 0.000392132 | ZNF630 |
| ENSG00000142347 | 0.686783866 | 0.031424902 | -4.44987601 | 1.83E-05 | 0.00018176 | MYO1F |
| ENSG00000207870 | 2242.921224 | 101.039169 | -4.472392367 | 3.53E-07 | 5.45E-06 | MIR221 |
| ENSG00000230454 | 0.453696499 | 0.020165467 | -4.49176878 | 0.000185873 | 0.001346458 | U73166.1 |
| ENSG00000204323 | 1.075290445 | 0.046022499 | -4.546243256 | 8.59E-08 | 1.51E-06 | SMIM5 |
| ENSG00000003989 | 0.908176885 | 0.038648538 | -4.554487568 | 9.92E-08 | 1.73E-06 | SLC7A2 |
| ENSG00000124134 | 4.695526238 | 0.199787304 | -4.55475004 | 1.10E-05 | 0.000116241 | KCNS1 |
| ENSG00000113924 | 32.0618357 | 1.340319123 | -4.580208585 | 0.00083199 | 0.004666365 | HGD |
| ENSG00000164509 | 0.110060465 | 0.004523316 | -4.604771646 | 1.25E-05 | 0.000129592 | IL31RA |
| ENSG00000125740 | 0.422723911 | 0.01732166 | -4.609066607 | 1.67E-10 | 4.91E-09 | FOSB |
| ENSG00000114923 | 0.064363272 | 0.002630541 | -4.612806354 | 7.33E-09 | 1.59E-07 | SLC4A3 |
| ENSG00000197249 | 0.991788552 | 0.040532834 | -4.612869603 | 0 | 0 | SERPINA1 |
| ENSG00000095539 | 0.220050543 | 0.008641128 | -4.670471415 | 8.79E-05 | 0.000710522 | SEMA4G |
| ENSG00000110169 | 1.632164883 | 0.061868375 | -4.721440854 | 2.14E-12 | 8.38E-11 | HPX |
| ENSG00000131781 | 0.57785581 | 0.021573271 | -4.74339273 | 2.22E-16 | 1.49E-14 | FMO5 |
| ENSG00000182890 | 1.816659466 | 0.067685845 | -4.74629004 | 8.09E-10 | 2.10E-08 | GLUD2 |
| ENSG00000062282 | 0.355174568 | 0.012148412 | -4.869688593 | 9.06E-13 | 3.72E-11 | DGAT2 |
| ENSG00000106258 | 0.547716076 | 0.017716679 | -4.950248096 | 9.03E-06 | 9.72E-05 | CYP3A5 |
| ENSG00000143409 | 2.476591445 | 0.078382919 | -4.981672789 | 0 | 0 | MINDY1 |
| ENSG00000145536 | 0.120980586 | 0.00368432 | -5.037233214 | 1.48E-08 | 3.07E-07 | ADAMTS16 |
| ENSG00000132837 | 4.09242398 | 0.124434724 | -5.039494586 | 4.94E-12 | 1.77E-10 | DMGDH |
| ENSG00000133135 | 2.364722415 | 0.070865732 | -5.060438859 | 6.35E-05 | 0.000539148 | RNF128 |
| ENSG00000151322 | 8.593965884 | 0.254936576 | -5.075113769 | 1.20E-05 | 0.000125126 | NPAS3 |
| ENSG00000225091 | 11.53791981 | 0.340182154 | -5.083931873 | 8.86E-11 | 2.70E-09 | SNORA71A |
| ENSG00000199266 | 209.407835 | 5.708099647 | -5.197161091 | 5.56E-14 | 2.73E-12 | SNORA60 |
| ENSG00000179532 | 0.051663982 | 0.001296589 | -5.31636518 | 4.05E-06 | 4.79E-05 | DNHD1 |
| ENSG00000106025 | 10.03217165 | 0.251468348 | -5.318113307 | 6.60E-07 | 9.61E-06 | TSPAN12 |
| ENSG00000226953 | 2.602370873 | 0.064835009 | -5.326909726 | 4.81E-13 | 2.10E-11 | AC010890.1 |
| ENSG00000107796 | 1.159682106 | 0.028728365 | -5.335109675 | 4.67E-06 | 5.42E-05 | ACTA2 |
| ENSG00000117594 | 10.93732486 | 0.254930597 | -5.423011565 | 8.91E-07 | 1.25E-05 | HSD11B1 |
| ENSG00000078687 | 0.39921218 | 0.008965592 | -5.476613011 | 3.93E-10 | 1.08E-08 | TNRC6C |
| ENSG00000123453 | 1.080879321 | 0.0230921 | -5.548662294 | 0 | 0 | SARDH |
| ENSG00000138207 | 22.42211188 | 0.432381428 | -5.6964738 | 1.36E-09 | 3.39E-08 | RBP4 |
| ENSG00000255823 | 833.1024016 | 16.06014086 | -5.696937385 | 5.09E-10 | 1.37E-08 | MTRNR2L8 |
| ENSG00000144908 | 2.066918 | 0.039500907 | -5.70945155 | 4.44E-16 | 2.89E-14 | ALDH1L1 |
| ENSG00000118520 | 5.441316928 | 0.096999877 | -5.809829133 | 1.56E-07 | 2.61E-06 | ARG1 |
| ENSG00000207181 | 6.896581161 | 0.114369943 | -5.91410149 | 8.97E-07 | 1.26E-05 | SNORA14B |
| ENSG00000047457 | 9.785857215 | 0.153857927 | -5.99102755 | 0.000130831 | 0.000999231 | CP |
| ENSG00000165795 | 1.384823389 | 0.018575444 | -6.220161469 | 4.44E-16 | 2.89E-14 | NDRG2 |
| ENSG00000151655 | 12.74303729 | 0.160385098 | -6.312025275 | 1.35E-05 | 0.000138132 | ITIH2 |
| ENSG00000164879 | 10.20264715 | 0.127633275 | -6.320795206 | 4.16E-05 | 0.000372188 | CA3 |
| ENSG00000175697 | 0.694985501 | 0.008332363 | -6.382113324 | 2.52E-06 | 3.18E-05 | GPR156 |
| ENSG00000128918 | 28.48269609 | 0.324278969 | -6.456706444 | 2.05E-08 | 4.13E-07 | ALDH1A2 |
| ENSG00000130649 | 13.30183377 | 0.14725369 | -6.497177553 | 7.64E-12 | 2.66E-10 | CYP2E1 |
| ENSG00000187045 | 2.652677956 | 0.02703978 | -6.616222295 | 1.56E-09 | 3.84E-08 | TMPRSS6 |
| ENSG00000240520 | 11.82416996 | 0.115796561 | -6.674002694 | 1.43E-05 | 0.000145828 | UOX |
| ENSG00000106804 | 0.69415902 | 0.00650775 | -6.736963467 | 1.11E-16 | 7.67E-15 | C5 |
| ENSG00000238961 | 15.39525414 | 0.139819097 | -6.782780455 | 1.65E-05 | 0.000165082 | SNORA47 |
| ENSG00000151790 | 12.78077063 | 0.114114631 | -6.807347245 | 1.42E-06 | 1.91E-05 | TDO2 |
| ENSG00000021826 | 7.248763749 | 0.064397511 | -6.81458623 | 0 | 0 | CPS1 |
| ENSG00000081051 | 4.437163259 | 0.038413699 | -6.851872926 | 1.84E-12 | 7.30E-11 | AFP |
| ENSG00000147231 | 19.08140125 | 0.07984974 | -7.900663692 | 0 | 0 | CXorf57 |
| ENSG00000124713 | 4.909601098 | 0.017414301 | -8.139189445 | 0 | 0 | GNMT |
| ENSG00000163631 | 356.896443 | 0.651180369 | -9.0982326 | 4.00E-15 | 2.32E-13 | ALB |
|  |  |  |  |  |  |  |
