# Supplementary material for: PM2.5 promotes NSCLC carcinogenesis through translationally and transcriptionally activating DLAT-mediated glycolysis reprograming
Source: J Exp Clin Cancer Res. 2022 Jul 22;41:229. doi: 10.1186/s13046-022-02437-8 (PMC9308224; doi:10.1186/s13046-022-02437-8)
Supplement: Supplementary file 13 — Additional file 13: Table S5. Enrichment results of differentially expressed mRNAs by KEGG analysis. [file 13046_2022_2437_MOESM13_ESM.docx]

| **Table S5. Enrichment results of differentially expressed mRNAs by KEGG analysis** | | | | | |
| --- | --- | --- | --- | --- | --- |
| **KEGG ID** | **Description** | **GeneRatio** | **BgRatio** | **P value** | **Padj** |
| hsa05219 | Bladder cancer | 7/179 | 40/5830 | 0.000181 | 0.043222 |
| hsa05418 | Fluid shear stress and atherosclerosis | 12/179 | 125/5830 | 0.000415 | 0.046419 |
| hsa04064 | NF-kappa B signaling pathway | 9/179 | 78/5830 | 0.000583 | 0.046419 |
| hsa04978 | Mineral absorption | 6/179 | 38/5830 | 0.000939 | 0.056076 |
| hsa04913 | Ovarian steroidogenesis | 5/179 | 33/5830 | 0.003045 | 0.145566 |
| hsa00140 | Steroid hormone biosynthesis | 4/179 | 24/5830 | 0.005643 | 0.177246 |
| hsa01521 | EGFR tyrosine kinase inhibitor resistance | 7/179 | 73/5830 | 0.006755 | 0.177246 |
| hsa05200 | Pathways in cancer | 24/179 | 460/5830 | 0.006762 | 0.177246 |
| hsa04550 | Signaling pathways regulating pluripotency of stem cells | 9/179 | 113/5830 | 0.007621 | 0.177246 |
| hsa00980 | Metabolism of xenobiotics by cytochrome P450 | 5/179 | 42/5830 | 0.008741 | 0.177246 |
| hsa05165 | Human papillomavirus infection | 17/179 | 295/5830 | 0.008745 | 0.177246 |
| hsa04060 | Cytokine-cytokine receptor interaction | 13/179 | 202/5830 | 0.008899 | 0.177246 |
| hsa00410 | beta-Alanine metabolism | 4/179 | 28/5830 | 0.009885 | 0.181729 |
| hsa05206 | MicroRNAs in cancer | 11/179 | 162/5830 | 0.010753 | 0.183512 |
| hsa04390 | Hippo signaling pathway | 10/179 | 143/5830 | 0.012164 | 0.183512 |
| hsa05323 | Rheumatoid arthritis | 6/179 | 63/5830 | 0.012285 | 0.183512 |
| hsa05412 | Arrhythmogenic right ventricular cardiomyopathy (ARVC) | 6/179 | 64/5830 | 0.013221 | 0.185867 |
| hsa05204 | Chemical carcinogenesis | 5/179 | 49/5830 | 0.016504 | 0.219139 |
| hsa05202 | Transcriptional misregulation in cancer | 10/179 | 155/5830 | 0.020405 | 0.256672 |
| hsa00340 | Histidine metabolism | 3/179 | 20/5830 | 0.02209 | 0.263199 |
| hsa02010 | ABC transporters | 4/179 | 36/5830 | 0.023532 | 0.263199 |
| hsa00010 | Glycolysis / Gluconeogenesis | 5/179 | 54/5830 | 0.024228 | 0.263199 |
| hsa04380 | Osteoclast differentiation | 7/179 | 96/5830 | 0.027529 | 0.280156 |
| hsa04216 | Ferroptosis | 4/179 | 38/5830 | 0.028133 | 0.280156 |
| hsa04622 | RIG-I-like receptor signaling pathway | 5/179 | 57/5830 | 0.02982 | 0.285076 |
| hsa04350 | TGF-beta signaling pathway | 6/179 | 79/5830 | 0.033719 | 0.298477 |
| hsa04657 | IL-17 signaling pathway | 6/179 | 79/5830 | 0.033719 | 0.298477 |
| hsa00030 | Pentose phosphate pathway | 3/179 | 26/5830 | 0.044086 | 0.370478 |
| hsa00330 | Arginine and proline metabolism | 4/179 | 44/5830 | 0.044953 | 0.370478 |
