# Supplementary material for: PM2.5 promotes NSCLC carcinogenesis through translationally and transcriptionally activating DLAT-mediated glycolysis reprograming
Source: J Exp Clin Cancer Res. 2022 Jul 22;41:229. doi: 10.1186/s13046-022-02437-8 (PMC9308224; doi:10.1186/s13046-022-02437-8)
Supplement: Supplementary file 14 — Additional file 14: Table S6. Enrichment results of differentially translated genes by KEGG analysis. [file 13046_2022_2437_MOESM14_ESM.docx]

| **Table S6. Enrichment results of differentially translated genes by KEGG analysis** | | | | | |
| --- | --- | --- | --- | --- | --- |
| **KEGG ID** | **Description** | **GeneRatio** | **BgRatio** | **P value** | **Padj** |
| hsa03040 | Spliceosome | 56/1472 | 123/6126 | 1.13E-07 | 3.51E-05 |
| hsa00020 | Citrate cycle (TCA cycle) | 20/1472 | 29/6126 | 3.73E-07 | 5.78E-05 |
| hsa03013 | RNA transport | 64/1472 | 153/6126 | 6.58E-07 | 6.21E-05 |
| hsa01200 | Carbon metabolism | 49/1472 | 108/6126 | 8.01E-07 | 6.21E-05 |
| hsa04141 | Protein processing in endoplasmic reticulum | 61/1472 | 153/6126 | 7.87E-06 | 0.000488 |
| hsa05322 | Systemic lupus erythematosus | 41/1472 | 95/6126 | 2.79E-05 | 0.00139 |
| hsa00310 | Lysine degradation | 28/1472 | 57/6126 | 3.14E-05 | 0.00139 |
| hsa00100 | Steroid biosynthesis | 12/1472 | 18/6126 | 0.0001502 | 0.0052268 |
| hsa05203 | Viral carcinogenesis | 65/1472 | 180/6126 | 0.0001517 | 0.0052268 |
| hsa00190 | Oxidative phosphorylation | 48/1472 | 124/6126 | 0.0001692 | 0.0052446 |
| hsa01230 | Biosynthesis of amino acids | 30/1472 | 68/6126 | 0.0002029 | 0.005717 |
| hsa05110 | Vibrio cholerae infection | 22/1472 | 45/6126 | 0.00024 | 0.0061998 |
| hsa04610 | Complement and coagulation cascades | 30/1472 | 69/6126 | 0.0002777 | 0.0066215 |
| hsa05130 | Pathogenic Escherichia coli infection | 23/1472 | 51/6126 | 0.0007542 | 0.0167004 |
| hsa00620 | Pyruvate metabolism | 17/1472 | 34/6126 | 0.0008784 | 0.0181545 |
| hsa04142 | Lysosome | 44/1472 | 119/6126 | 0.000977 | 0.0182555 |
| hsa05230 | Central carbon metabolism in cancer | 26/1472 | 61/6126 | 0.0010011 | 0.0182555 |
| hsa04110 | Cell cycle | 44/1472 | 120/6126 | 0.0011915 | 0.0205205 |
| hsa00010 | Glycolysis / Gluconeogenesis | 25/1472 | 59/6126 | 0.001377 | 0.022467 |
| hsa01210 | 2-Oxocarboxylic acid metabolism | 10/1472 | 17/6126 | 0.0022236 | 0.0344655 |
| hsa05100 | Bacterial invasion of epithelial cells | 27/1472 | 69/6126 | 0.0036085 | 0.0495449 |
| hsa05206 | MicroRNAs in cancer | 73/1472 | 229/6126 | 0.0036398 | 0.0495449 |
| hsa04979 | Cholesterol metabolism | 20/1472 | 47/6126 | 0.0038061 | 0.0495449 |
| hsa03050 | Proteasome | 18/1472 | 41/6126 | 0.0039651 | 0.0495449 |
| hsa04216 | Ferroptosis | 17/1472 | 38/6126 | 0.0039956 | 0.0495449 |
| hsa04530 | Tight junction | 51/1472 | 152/6126 | 0.0045861 | 0.0546809 |
| hsa03010 | Ribosome | 44/1472 | 129/6126 | 0.0058276 | 0.0669096 |
| hsa01040 | Biosynthesis of unsaturated fatty acids | 11/1472 | 22/6126 | 0.0071929 | 0.0783035 |
| hsa05323 | Rheumatoid arthritis | 23/1472 | 59/6126 | 0.0073252 | 0.0783035 |
| hsa00630 | Glyoxylate and dicarboxylate metabolism | 13/1472 | 28/6126 | 0.0078198 | 0.080805 |
| hsa05205 | Proteoglycans in cancer | 60/1472 | 189/6126 | 0.0087312 | 0.0873118 |
| hsa00220 | Arginine biosynthesis | 10/1472 | 20/6126 | 0.0102986 | 0.0997681 |
| hsa03060 | Protein export | 11/1472 | 23/6126 | 0.0108406 | 0.1018363 |
| hsa04145 | Phagosome | 38/1472 | 114/6126 | 0.0147974 | 0.1349173 |
| hsa00052 | Galactose metabolism | 12/1472 | 27/6126 | 0.0156796 | 0.1388766 |
| hsa05120 | Epithelial cell signaling in Helicobacter pylori infection | 22/1472 | 60/6126 | 0.018861 | 0.1624144 |
| hsa01212 | Fatty acid metabolism | 18/1472 | 47/6126 | 0.0202311 | 0.1695034 |
| hsa05418 | Fluid shear stress and atherosclerosis | 40/1472 | 124/6126 | 0.022085 | 0.1801671 |
| hsa05010 | Alzheimer disease | 50/1472 | 162/6126 | 0.0266468 | 0.2118078 |
| hsa05034 | Alcoholism | 48/1472 | 156/6126 | 0.0310276 | 0.2356255 |
| hsa05016 | Huntington disease | 55/1472 | 182/6126 | 0.0311634 | 0.2356255 |
| hsa05210 | Colorectal cancer | 28/1472 | 84/6126 | 0.0331783 | 0.2411542 |
| hsa05216 | Thyroid cancer | 14/1472 | 36/6126 | 0.0334504 | 0.2411542 |
| hsa00410 | beta-Alanine metabolism | 12/1472 | 30/6126 | 0.0380074 | 0.2677792 |
| hsa05219 | Bladder cancer | 15/1472 | 40/6126 | 0.0392437 | 0.2703456 |
| hsa03430 | Mismatch repair | 9/1472 | 21/6126 | 0.0443877 | 0.2991345 |
| hsa04217 | Necroptosis | 41/1472 | 134/6126 | 0.0474887 | 0.3132233 |
| hsa03420 | Nucleotide excision repair | 15/1472 | 41/6126 | 0.0485819 | 0.3137579 |
