# Supplementary material for: PM2.5 promotes NSCLC carcinogenesis through translationally and transcriptionally activating DLAT-mediated glycolysis reprograming
Source: J Exp Clin Cancer Res. 2022 Jul 22;41:229. doi: 10.1186/s13046-022-02437-8 (PMC9308224; doi:10.1186/s13046-022-02437-8)
Supplement: Supplementary file 15 — Additional file 15: Table S7. Enrichment results of genes with significant translation efficiency (TE) changes by KEGG analysis. [file 13046_2022_2437_MOESM15_ESM.docx]

| **Table S7. Enrichment results of genes with significant translation efficiency (TE) changes by KEGG analysis** | | | | | |
| --- | --- | --- | --- | --- | --- |
| **KEGG ID** | **Description** | **GeneRatio** | **BgRatio** | **P value** | **Padj** |
| hsa00010 | **Glycolysis / Gluconeogenesis** | 27/1483 | 42/4390 | 4.79E-05 | **0.01399777** |
| hsa03013 | RNA transport | 66/1483 | 139/4390 | 0.000471 | 0.058202501 |
| hsa00100 | Steroid biosynthesis | 11/1483 | 14/4390 | 0.000772 | 0.058202501 |
| hsa00620 | Pyruvate metabolism | 19/1483 | 30/4390 | 0.000854 | 0.058202501 |
| hsa01040 | Biosynthesis of unsaturated fatty acids | 14/1483 | 20/4390 | 0.000997 | 0.058202501 |
| hsa00310 | Lysine degradation | 28/1483 | 51/4390 | 0.001467 | 0.071389324 |
| hsa00340 | Histidine metabolism | 10/1483 | 13/4390 | 0.001824 | 0.076082208 |
| hsa00052 | Galactose metabolism | 14/1483 | 22/4390 | 0.003926 | 0.143308982 |
| hsa04216 | Ferroptosis | 19/1483 | 34/4390 | 0.006464 | 0.194828146 |
| hsa01212 | Fatty acid metabolism | 22/1483 | 41/4390 | 0.006672 | 0.194828146 |
| hsa01200 | Carbon metabolism | 43/1483 | 93/4390 | 0.007939 | 0.210757091 |
| hsa00220 | Arginine biosynthesis | 10/1483 | 15/4390 | 0.009338 | 0.227229237 |
| hsa03040 | Spliceosome | 52/1483 | 118/4390 | 0.011861 | 0.266418859 |
| hsa00563 | Glycosylphosphatidylinositol (GPI)-anchor biosynthesis | 13/1483 | 22/4390 | 0.012899 | 0.269044586 |
| hsa04141 | Protein processing in endoplasmic reticulum | 62/1483 | 146/4390 | 0.016181 | 0.295698334 |
| hsa04142 | Lysosome | 47/1483 | 107/4390 | 0.017352 | 0.295698334 |
| hsa01210 | 2-Oxocarboxylic acid metabolism | 10/1483 | 16/4390 | 0.017438 | 0.295698334 |
| hsa00980 | Metabolism of xenobiotics by cytochrome P450 | 14/1483 | 25/4390 | 0.018228 | 0.295698334 |
| hsa00983 | Drug metabolism - other enzymes | 21/1483 | 42/4390 | 0.021261 | 0.305129495 |
| hsa04530 | Tight junction | 50/1483 | 116/4390 | 0.021426 | 0.305129495 |
| hsa05230 | Central carbon metabolism in cancer | 25/1483 | 52/4390 | 0.022303 | 0.305129495 |
| hsa00982 | Drug metabolism - cytochrome P450 | 12/1483 | 21/4390 | 0.023345 | 0.305129495 |
| hsa05204 | Chemical carcinogenesis | 15/1483 | 28/4390 | 0.024034 | 0.305129495 |
| hsa00071 | Fatty acid degradation | 16/1483 | 31/4390 | 0.030161 | 0.352282926 |
| hsa04610 | Complement and coagulation cascades | 16/1483 | 31/4390 | 0.030161 | 0.352282926 |
| hsa00410 | beta-Alanine metabolism | 12/1483 | 22/4390 | 0.035881 | 0.375535162 |
| hsa03060 | Protein export | 12/1483 | 22/4390 | 0.035881 | 0.375535162 |
| hsa04110 | Cell cycle | 48/1483 | 114/4390 | 0.037186 | 0.375535162 |
| hsa04964 | Proximal tubule bicarbonate reclamation | 8/1483 | 13/4390 | 0.037296 | 0.375535162 |
| hsa00020 | Citrate cycle (TCA cycle) | 14/1483 | 27/4390 | 0.039688 | 0.386296581 |
| hsa01230 | Biosynthesis of amino acids | 25/1483 | 55/4390 | 0.046841 | 0.441210654 |
| hsa05110 | Vibrio cholerae infection | 19/1483 | 40/4390 | 0.049382 | 0.450614491 |
