# Supplementary material for: PM2.5 promotes NSCLC carcinogenesis through translationally and transcriptionally activating DLAT-mediated glycolysis reprograming
Source: J Exp Clin Cancer Res. 2022 Jul 22;41:229. doi: 10.1186/s13046-022-02437-8 (PMC9308224; doi:10.1186/s13046-022-02437-8)
Supplement: Supplementary file 16 — Additional file 16: Table S8. Results of gene set enrichment analysis (GSEA) on differentially expressed genes in translatome. [file 13046_2022_2437_MOESM16_ESM.docx]

| **Table S8. Results of gene set enrichment analysis (GSEA) on differentially expressed genes in translatome** | | | | | |
| --- | --- | --- | --- | --- | --- |
| **NAME** | **SIZE** | **ES** | **NES** | **NOM p-val** | **FDR q-val** |
| NOD-LIKE RECEPTOR SIGNALING PATHWAY(HSA04621) | 163 | 0.388483 | 1.153364 | 0 | 0.297248 |
| AUTOPHAGY - ANIMAL(HSA04140) | 127 | 0.393295 | 1.124688 | 0 | 0.337199 |
| KAPOSI SARCOMA-ASSOCIATED HERPESVIRUS INFECTION(HSA05167) | 177 | 0.406984 | 1.198841 | 0 | 0.233737 |
| ALCOHOLISM(HSA05034) | 179 | 0.410929 | 1.261102 | 0 | 0.15845 |
| GLYCOSAMINOGLYCAN BIOSYNTHESIS - HEPARAN SULFATE HEPARIN(HSA00534) | 24 | 0.413759 | 1.133296 | 0 | 0.322897 |
| CELLULAR SENESCENCE(HSA04218) | 148 | 0.422648 | 1.229946 | 0 | 0.203869 |
| SMALL CELL LUNG CANCER(HSA05222) | 91 | 0.437721 | 1.214134 | 0 | 0.215904 |
| HEPATITIS B(HSA05161) | 141 | 0.439778 | 1.187677 | 0 | 0.248252 |
| METABOLIC PATHWAYS(HSA01100) | 1270 | 0.44093 | 1.206925 | 0 | 0.223652 |
| NECROPTOSIS(HSA04217) | 160 | 0.44173 | 1.296099 | 0 | 0.148962 |
| HIF-1 SIGNALING PATHWAY(HSA04066) | 98 | 0.443914 | 1.175297 | 0 | 0.273283 |
| SYNAPTIC VESICLE CYCLE(HSA04721) | 59 | 0.44561 | 1.215143 | 0 | 0.218648 |
| HUNTINGTON DISEASE(HSA05016) | 190 | 0.445881 | 1.275728 | 0 | 0.152273 |
| MTOR SIGNALING PATHWAY(HSA04150) | 144 | 0.448386 | 1.270215 | 0 | 0.153376 |
| HEPATITIS C(HSA05160) | 128 | 0.448872 | 1.299513 | 0 | 0.142855 |
| GLYCEROPHOSPHOLIPID METABOLISM(HSA00564) | 96 | 0.458983 | 1.261841 | 0 | 0.16087 |
| UBIQUITIN MEDIATED PROTEOLYSIS(HSA04120) | 136 | 0.46068 | 1.212503 | 0 | 0.212311 |
| LEGIONELLOSIS(HSA05134) | 51 | 0.474088 | 1.327142 | 0 | 0.127748 |
| HEMATOPOIETIC CELL LINEAGE(HSA04640) | 83 | 0.475569 | 1.2778 | 0 | 0.153376 |
| SYSTEMIC LUPUS ERYTHEMATOSUS(HSA05322) | 116 | 0.485493 | 1.330433 | 0 | 0.123519 |
| GLYCOLYSIS GLUCONEOGENESIS(HSA00010) | 66 | 0.491739 | 1.214405 | 0 | 0.218085 |
| PROTEIN PROCESSING IN ENDOPLASMIC RETICULUM(HSA04141) | 158 | 0.493426 | 1.249987 | 0 | 0.172359 |
| P53 SIGNALING PATHWAY(HSA04115) | 72 | 0.499954 | 1.215756 | 0 | 0.220235 |
| ALZHEIMER DISEASE(HSA05010) | 169 | 0.503275 | 1.291682 | 0 | 0.149861 |
| CENTRAL CARBON METABOLISM IN CANCER(HSA05230) | 64 | 0.515641 | 1.283085 | 0 | 0.155811 |
| GLUTATHIONE METABOLISM(HSA00480) | 54 | 0.516443 | 1.265 | 0 | 0.155902 |
| PARKINSON DISEASE(HSA05012) | 141 | 0.517058 | 1.327681 | 0 | 0.122145 |
| CARBON METABOLISM(HSA01200) | 114 | 0.521328 | 1.273949 | 0 | 0.152482 |
| PHAGOSOME(HSA04145) | 126 | 0.530649 | 1.30405 | 0 | 0.143402 |
| ECM-RECEPTOR INTERACTION(HSA04512) | 80 | 0.536212 | 1.318068 | 0 | 0.129807 |
| EPITHELIAL CELL SIGNALING IN HELICOBACTER PYLORI INFECTION(HSA05120) | 64 | 0.542278 | 1.377897 | 0 | 0.10893 |
| MANNOSE TYPE O-GLYCAN BIOSYNTHESIS(HSA00515) | 23 | 0.550091 | 1.195982 | 0 | 0.236988 |
| BLADDER CANCER(HSA05219) | 41 | 0.550354 | 1.443558 | 0 | 0.099347 |
| OXIDATIVE PHOSPHORYLATION(HSA00190) | 130 | 0.554836 | 1.335215 | 0 | 0.130331 |
| ETHER LIPID METABOLISM(HSA00565) | 47 | 0.560197 | 1.372816 | 0 | 0.107476 |
| PYRUVATE METABOLISM(HSA00620) | 37 | 0.571528 | 1.206465 | 0 | 0.220791 |
| RHEUMATOID ARTHRITIS(HSA05323) | 69 | 0.586926 | 1.445768 | 0 | 0.102764 |
| PROTEIN EXPORT(HSA03060) | 23 | 0.629725 | 1.249869 | 0 | 0.169799 |
| FERROPTOSIS(HSA04216) | 40 | 0.64952 | 1.36837 | 0 | 0.10865 |
| OTHER TYPES OF O-GLYCAN BIOSYNTHESIS(HSA00514) | 22 | 0.657408 | 1.405035 | 0 | 0.117658 |
| COLLECTING DUCT ACID SECRETION(HSA04966) | 25 | 0.662074 | 1.441247 | 0 | 0.091456 |
| AMINO SUGAR AND NUCLEOTIDE SUGAR METABOLISM(HSA00520) | 46 | 0.663353 | 1.34628 | 0 | 0.125774 |
| GALACTOSE METABOLISM(HSA00052) | 29 | 0.675079 | 1.381898 | 0 | 0.112996 |
| GLYCOSPHINGOLIPID BIOSYNTHESIS - GLOBO AND ISOGLOBO SERIES(HSA00603) | 15 | 0.67601 | 1.46421 | 0 | 0.119851 |
| TERPENOID BACKBONE BIOSYNTHESIS(HSA00900) | 22 | 0.676636 | 1.220936 | 0 | 0.215098 |
| VIBRIO CHOLERAE INFECTION(HSA05110) | 47 | 0.693598 | 1.367051 | 0 | 0.108942 |
| FATTY ACID METABOLISM(HSA01212) | 47 | 0.71012 | 1.423245 | 0 | 0.087406 |
| LYSOSOME(HSA04142) | 121 | 0.713544 | 1.434907 | 0 | 0.081592 |
| FATTY ACID ELONGATION(HSA00062) | 30 | 0.72082 | 1.393532 | 0 | 0.113186 |
| 2-OXOCARBOXYLIC ACID METABOLISM(HSA01210) | 18 | 0.730337 | 1.332477 | 0 | 0.12677 |
| AMINOACYL-TRNA BIOSYNTHESIS(HSA00970) | 41 | 0.75142 | 1.270786 | 0 | 0.155975 |
| GLYCOSYLPHOSPHATIDYLINOSITOL (GPI)-ANCHOR BIOSYNTHESIS(HSA00563) | 25 | 0.757697 | 1.267944 | 0 | 0.153165 |
| CITRATE CYCLE (TCA CYCLE)(HSA00020) | 30 | 0.758344 | 1.302396 | 0 | 0.141974 |
| SPHINGOLIPID METABOLISM(HSA00600) | 45 | 0.770517 | 1.471588 | 0 | 0.149156 |
| STEROID BIOSYNTHESIS(HSA00100) | 18 | 0.777271 | 1.400971 | 0 | 0.111689 |
| N-GLYCAN BIOSYNTHESIS(HSA00510) | 49 | 0.778354 | 1.449849 | 0 | 0.119686 |
| OTHER GLYCAN DEGRADATION(HSA00511) | 16 | 0.805284 | 1.441154 | 0 | 0.085819 |
| BIOSYNTHESIS OF UNSATURATED FATTY ACIDS(HSA01040) | 22 | 0.820236 | 1.344932 | 0 | 0.122086 |
