# Supplementary material for: PM2.5 promotes NSCLC carcinogenesis through translationally and transcriptionally activating DLAT-mediated glycolysis reprograming
Source: J Exp Clin Cancer Res. 2022 Jul 22;41:229. doi: 10.1186/s13046-022-02437-8 (PMC9308224; doi:10.1186/s13046-022-02437-8)
Supplement: Supplementary file 17 — Additional file 17: Table S9. Characteristics of NSCLC patients recruited for analysis of SUVmax values of PCT-CT and DLAT IHC staining. [file 13046_2022_2437_MOESM17_ESM.docx]

| **Table S9. Characteristics of NSCLC patients with PET-CT scanning** | | | | | |
| --- | --- | --- | --- | --- | --- |
| **Study ID** | **Sex** | **Age** | **TNM Stage** | **Stage** | **SUVmax (PET/CT)** |
| PET-CT.1 | Male | 52 | pT2aN1M0 | IIA | 12.28 |
| PET-CT.2 | Male | 64 | pT2aN0M0 | IB | 6.26 |
| PET-CT.3 | Male | 66 | pT2aN0M0 | IB | 2.5 |
| PET-CT.4 | Female | 65 | pT2aN0M0 | IB | 6.11 |
| PET-CT.5 | Male | 64 | pT2aN1M0 | IIB | 13.6 |
| PET-CT.6 | Male | 72 | pT2aN3M1a | IV | 6.4 |
| PET-CT.7 | Male | 66 | pT2aN2M0 | IIIA | 9.9 |
| PET-CT.8 | Male | 73 | pT2aN0M0 | IB | 22.2 |
| PET-CT.9 | Male | 69 | pT1cN0M0 | IA3 | 4.2 |
| PET-CT.10 | Male | 55 | pT4N0M0 | IIIA | 11.2 |
| PET-CT.11 | Female | 71 | pT2aN0M0 | IB | 16 |
| PET-CT.12 | Male | 50 | pT2bN0M0 | IIA | 9.3 |
| PET-CT.13 | Male | 68 | PT2aN1M0 | Iib | 6.5 |
| PET-CT.14 | Male | 79 | pT1cN0M0 | IA3 | 8.7 |
| PET-CT.15 | Female | 39 | pT2aN0M0 | Ib | 2.1 |
