# Supplementary material for: PM2.5 promotes NSCLC carcinogenesis through translationally and transcriptionally activating DLAT-mediated glycolysis reprograming
Source: J Exp Clin Cancer Res. 2022 Jul 22;41:229. doi: 10.1186/s13046-022-02437-8 (PMC9308224; doi:10.1186/s13046-022-02437-8)
Supplement: Supplementary file 18 — Additional file 18: Table S10. Primers and antibodies used in this study. [file 13046_2022_2437_MOESM18_ESM.docx]

| **Table S10.** **Primers and antibodies used in this study** | |
| --- | --- |
| **Oligo names** | **Sequences (5 ́-3 ́)** |
| Primers for DLAT (human) | Forward: GCAGGACTCATCACACCTATTGT  Reverse: GTAGTTTACCCTCTCTTGCTTTGG |
| Primers for Dlat (rat) | Forward: CTATCCCGTTCACATGCAGATT  Reverse: TATGGTGGCCTTGTCGGTC |
| Primers for Sp1 (human) | Forward: AGGTCAGTTGGCAGACTCTACAGC  Reverse: GCATTGGGGCTAAGGTGATTG |
| Primers for Sp1 (rat) | Forward: GCCCTTATTACCACCAATATGGTAG  Reverse: GTCTAATCTCAGAAACCATTGCCA |
| Primers for eIF4E (human) | Forward: GGTATTGAGCCTATGTGGGAAG  Reverse: TCGTCTCTGCTGTTTGTTCAATG |
| Primers for eIF4E (rat) | Forward: GAACAAGCAGCAGAGACGAAGT  Reverse: TTCTCCAATAAGGCACAGCAGT |
| Primers for site 1 in DLAT promoter | Forward: TTAACCCGCGTCTCTGC  Reverse: GGAAAGTGGTAGGGTTCTAGG |
| Primers for site 2 in DLAT promoter | Forward: TCGGCCCAGACTCTCAGG  Reverse: CAAGGGCACGACCGAGG |
| Primers for site 3 in DLAT promoter | Forward: CCGCAATTAACCCGCGTC  Reverse: AGGCATCCGAAAGGGTGAG |
| Primers for ACTB | Forward: TCAAGATCATTGCTCCTCCTGAG  Reverse: ACATCTGCTGGAAGGTGGACA |
| si-DLAT#1 | CAGTGAATTGTCTTTTAGACAAC |
| si-DLAT#2 | TTGTCTTTTAGACAACTAGATTT |
| si-DLAT#3 | GTCTTTTAGACAACTAGATTTGT |
| si-eIF4E#1 | TGGCGCTGTTGTTAATGTT |
| si-eIF4E#2 | AGAGCTAAAGGTGATAAGA |
| si-eIF4E#3 | AGGACGATGGCTAATTACA |
| si-NC | Ribobio, # siN0000001-1 |
| **Antibodies** | **Source** |
| DLAT antibody for Western blot (1:1000) | SIGMA (R38114) |
| eIF4E antibody for Western blot (1:1000) | CST (2067) |
| Sp1 antibody for Western blot (1:1000) | CST (9389) |
| DLAT antibody for IHC (1:5000) | SIGMA (R38114) |
| Ki67 antibody for IHC (1:2000) | Proteintech (27309-1-AP) |
| Caspase-3 antibody for IHC (1:2000) | Proteintech (66470-2-Ig) |
| β-actin antibody (1:2000) | CST (4970) |
| Peroxidase-Conjugated Goat anti-Rabbit IgG (H+L) (1:10000) | ZSGB-BIO (ZB-5301) |
